# 10th Congress of International Society of Systemic Auto-Inflammatory Diseases (ISSAID): Genoa, Italy. 31 March - 3 April 2019

**DOI:** 10.1186/s12969-019-0313-x

**Published:** 2019-05-16

## Oral presentations

### Molecular determinants of innate immunity

#### O01 New MVK mutant mouse avatars of mevalonate kinase deficiency mimic the underlying defect in protein prenylation in patients

##### Marcia A. Munoz^1^, Oliver P. Skinner^1^, Julie Jurczyluk^1^, Kristen Perry^1^, Robert Brink^2^, David Zahra^2^, Rob J. Arts^3^, Anna Simon^3^, Michael J. Rogers^1^

###### ^1^Bone Biology; ^2^Immunology, Garvan Institute of Medical Research, Sydney, Australia; ^3^Medical Centre, Radboud University, Nijmegen, Netherlands

####### **Correspondence:** Marcia A. Munoz

**Introduction:** Mevalonate Kinase Deficiency (MKD) is a periodic fever syndrome characterised by recurrent bouts of high fever and systemic inflammation. MKD is caused by recessive, hypomorphic mutations in the mevalonate kinase gene (*MVK*) encoding a key enzyme in the mevalonate pathway. This pathway is responsible for cholesterol synthesis and the production of isoprenoid lipid tags required for post-translational prenylation of proteins. It is believed that inflammation in MKD is triggered by shortage of isoprenoid lipids and defective prenylation of small GTPases. We have found that protein prenylation is indeed compromised in PBMCs from patients and this defect distinguishes MKD from other periodic fever syndromes. However, the link between protein prenylation and inflammation in MKD is still far from understood, largely due to the lack of suitable genetic mouse models.

**Objectives:** To generate new *Mvk*mutant mouse models of MKD that mimic the human disease.

**Methods:** CRISPR/Cas9 gene editing was used to generate different heterozygous mouse lines with hypomorphic mutations in exon 11 of the*Mvk*gene: a V377I substitution (the most frequent mutation in MKD), and 8, 13 or 91 base pair deletions. These lines were then crossed to generate homozygous *Mvk*^*V377I/V377I*^or *Mvk*^*V377I/deI*^compound heterozygous mice. To assess the effect of the mutations on the mevalonate pathway, we measured the accumulation of unprenylated Rab GTPases and Rap1A using an *in vitro*prenylation assay and western blotting. For quantification of inflammatory cytokines in serum, as well as in culture supernatants from LPS-stimulated PBMCs and bone marrow macrophages, we used ELISA and multiplex cytokine bead arrays.

**Results:** Homozygous mice carrying complete loss of function deletion mutations were not viable and, as expected, wildtype and heterozygous *Mvk*mutant mice had normal protein prenylation. However, in a similar pattern to patient PBMCs, *Mvk*^*V377I/deI*^immune cells from blood, spleen and bone marrow had a dramatic accumulation of unprenylated Rab and Rap1A GTPases, with a much milder defect in *Mvk*^*V377I/V377I*^cells. This is consistent with reportedly less severe clinical disease associated with the homozygous V377I mutation. Furthermore, *Mvk*^*V377I/deI*^ mice had slightly elevated levels of inflammatory serum cytokines, including IL-6 and G-CSF, and cultures of PBMCs and bone marrow macrophages responded more robustly to LPS stimulation than cells from control mice. Interestingly, like patient-derived cell lines, the prenylation defect was dramatically enhanced in bone marrow cells from *Mvk*mutant mice after briefly culturing at higher temperature (39-40^o^C). Importantly, addition of the missing isoprenoid lipid geranylgeraniol could rescue the prenylation defect in primary cell cultures *in vitro*and in peritoneal macrophages *in vivo*.

**Conclusion:** To our knowledge, we have generated the first genetic mouse avatars of MKD. These mice, like MKD patients, have defective protein prenylation and an exaggerated inflammatory response. Furthermore, as with patient-derived cell lines, *Mvk*mutant mouse cells are temperature-sensitive, suggesting that elevations in body temperature (e.g. caused by mild infections) could quickly precipitate devastating defects in protein prenylation that lead to systemic inflammatory flares. These mice are exciting new tools to study the pathophysiology of MKD and can be used to develop new therapeutic approaches, such as supplementation with isoprenoid lipids.

**Disclosure of Interest**

None Declared

#### O02 Generation and analysis of mice carrying a novel heterozygous missense mutation of a proteasome subunit, PSMB9, in patients with autoinflammation and immunodeficiency

##### Hiroaki Hemmi^1^, Nobuo Kanazawa^2^, Noriko Kinjo^3^, Satoru Hamada^3^, Hidenori Ohnishi^4^, Tsunehiro Mizushima^5^, Akira Kinoshita^6^, Koh-Ichiro Yoshiura^6^, Tsuneyasu Kaisho^1^

###### ^1^Department of Immunology, Wakayama Medical University Institute of Advanced Medicine; ^2^Department of Dermatology, Wakayama Medical University, Wakayama; ^3^Department of Child Health and Welfare (Pediatrics), University of the Ryukyus Graduate School of Medicine, Nishihara; ^4^Department of Pediatrics, Gifu University Graduate School of Medicine, Gifu; ^5^Picobiology Institute, University of Hyogo Graduate School of Life Science, Kamigori; ^6^Department of Human Genetics, Nagasaki University Atomic Bomb Disease Institute, Nagasaki, Japan

####### **Correspondence:** Tsuneyasu Kaisho

**Introduction:** The proteasome is a large protein complex involved in degradation of unnecessary or useless proteins. Homozygous, compound heterozygous or digenic mutations of proteasome subunits cause autoinflammatory diseases, termed proteasome-associated autoinflammatory syndromes (PRAAS). De novo, heterozygous missense mutation in the proteasome subunit *PSMB9* (encodes β1i) gene (hereafter, *PSMB9 X* mutation), was commonly found in two unrelated patients showing PRAAS-like but distinct manifestations, on which two other posters are presented in this meeting. The *PSMB9 X* mutation is novel and causes a substitution of an amino acid conserved among multiple species.

**Objectives:** It is unclear whether and how the *PSMB9 X* mutation contributes to the manifestations of the patients. In order to clarify this issue, we have generated and analyzed mutant mice carrying the mutation.

**Methods:** The mice carrying the *Psmb9 X* mutation were generated with CRISPR/Cas9 technology. Homozygous *Psmb9 X* mutant mice died within 6 months old. Therefore, the heterozygous *Psmb9 X* mutant mice were mainly analyzed. Not only biochemical but also immunological analyses including histological and flow cytometry analyses were performed.

**Results:** Heterozygous *Psmb9 X* mutant mice appeared healthy at glance. The β1i subunit becomes mature after processing by the proteasome, but this maturation process was impaired in splenocytes of the heterozygous *Psmb9 X* mutant mice. In the mutant mice, thymus was small and the cortico-medullary junction was unclear. All thymocytes such as CD4^+^, CD8^+^, double positive and double negative cells were decreased. In the spleen, B cells as well as CD4^+^ and CD8^+^ T cells were decreased. Furthermore, serum levels of immunoglobulins, including IgM, IgGs and IgA, were severely decreased. Dendritic cells (DCs) and all DC subsets were also decreased, although the conventional DC1 subset, defined as CD8α^+^CD11b^-^ cells, was most severely decreased. Meanwhile, CD11b^+^ cells consisting mainly of neutrophils and monocytes were increased in the bone marrow. These phenotype of the heterozygous *Psmb9 X* mutant mice were not identical to, but overlapping significantly with the manifestations of the two patients.

**Conclusion:** We here generated mutant mice carrying a novel heterozygous missense mutation in the proteasome subunit *Psmb9* gene, which was identified in two unrelated PRAAS-like patients. Multiple defects in both innate and adaptive immune cells were observed in the heterozygous *Psmb9 X* mutant mice and some, although not all, defects were also observed in the two patients. These results indicate that the heterozygous *Psmb9 X* mutation can be the cause of the PRAAS-like phenotypes in the two patients. The findings that the mutation causes not only autoinflammation but also combined immunodeficiency prompt us to propose a novel category of autoinflammatory diseases distinct from PRAAS as “proteasome-associated autoinflammation and immunodeficiency disease (PRAID)”. The mutant mice are unique and quite useful for clarifying how the proteasome dysfunction leads to various manifestations of PRAID.

**Disclosure of Interest**

None Declared

### Mechanisms of inflammasome activation

#### O03 Cofilin-1 is an essential redox sensor for NLRP3 inflammasome activation

##### Wonyong Lee, Yong Hwan Park, Daniel L. Kastner, Jae Jin Chae

###### NHGRI, Bethesda, United States

####### **Correspondence:** Wonyong Lee

**Introduction:** NLRP3 has a pivotal role in nucleating the inflammasome, a cytoplasmic multiprotein complex that mediates the maturation of the proinflammatory cytokine interleukin-1β (IL-1β) by activating caspase-1. Mutations in the gene encoding NLRP3 cause a spectrum of autoinflammatory diseases, the cryopyrin-associated periodic syndromes (CAPS). The generation of reactive oxygen species (ROS) is one of the major NLRP3 inflammasome activating factors. However, the molecular basis of the relationship between change of cellular redox state and NLRP3 inflammasome activation has not been elucidated.

**Methods:** We utilized mouse bone marrow-derived macrophage (BMDM) to analyze interaction of cofilin-1 and NLRP3 by co-immunoprecipitation (co-IP). Mouse BMDMs were used to ectopically express wild-type (WT) or mutant cofilin-1 proteins, and to transfect siRNA for knockdown assay. Cofilin-1 knock-in (KI) mice (C39A or C39S) were generated by microinjection of sgRNA and Cas9 ribonucleoprotein (RNP) complex.

**Results:** To identify an ROS-mediated regulator for NLRP3 inflammasome activation, the immune complexes precipitated by NLRP3 specific antibody from BMDMs of WT or NLRP3-KO mice were analyzed by mass spectrometry. We found cofilin-1, the actin severing protein, as a negative regulator for the NLRP3 inflammasome. Cofilin-1 interacted with the nucleotide-binding domain (NBD) of NLRP3 and dissociated from NLRP3 when the cells were stimulated with known NLRP3 inflammasome activators, such as ATP or nigericin. The NLRP3 inflammasome activators generate ROS that leads to cofilin-1 oxidation, which is intramolecular disulfide bond formation between two cysteine residues at amino acids 39 and 80. This oxidation induces conformational change of cofilin-1 and dissociation from NLRP3, which results in the activation of the NLRP3 inflammasome. Indeed, the assembly of NLRP3 inflammasome components is impaired and the IL-1β release was significantly suppressed in BMDMs ectopically expressing oxidation-resistant mutant cofilin-1 (C39A or C80A). In addition, knockdown of cofilin-1 in LPS-primed BMDMs induced NLRP3 inflammasome activation without activator treatment. We also observed that the interaction of cofilin-1 with the CAPS-associated mutant NLRP3 proteins was substantially diminished relative to WT NLRP3, which resulted in constitutive activation of the NLRP3 inflammasome. To examine the role of cofilin as a redox sensor for NLRP3 inflammasome activation *in vivo*, we have generated KI mice expressing oxidation-resistant mutant cofilin-1 (C39A or C39S). Unexpectedly, the IL-1β release from the BMDMs of the KI mice was higher than WT BMDMs when the cells were stimulated with ATP after LPS priming. Consistently, IL-1β levels in the serum of KI mice after injection of lipopolysaccharide (LPS) intraperitoneally was significantly higher than WT mice. This inconsistent result may be due to the low level of mutant cofilin-1 in KI mice. Indeed, similarly to the result of cofilin-1 knockdown, LPS-primed KI BMDMs release substantial IL-1β without activators.

**Conclusion:** Taken together, these findings suggest that cofilin-1 is a key component in regulating the NLRP3 inflammasome in response to ROS. In addition, our data suggest cofilin-1 as a potential therapeutic target for the inflammatory conditions involving the NLRP3 inflammasome, including gout, type 2 diabetes mellitus, atherosclerosis, and Alzheimer’s disease.

**Disclosure of Interest**

None Declared

#### O04 Autoinflammatory mutation in NLRC4 reveals an LRR-LRR oligomerization interface

##### Fiona Moghaddas^1,2,3^, Ping Zeng^4^, Yuxia Zhang^5^, Heike Schuetzle^6^, Sebastian Brenner^6^, Sigrun Hofmann^6^, Reinhard Berner^6^, Yuanbo Zhao^5,7^, Bingtai Lu^5^, Xiaoyun Chen^5^, Li Zhang^5^, Suyun Cheng^4^, Stefan Winkler^6^, Kai Lehmberg^8^, Scott W. Canna^9^, Peter E. Czabotar^10,11^, Ian P. Wicks^2,11,12^, Dominic De Nardo^2,11^, Christian Hendrich^6,13,14^, Huasong Zeng^4^, Seth L. Masters^2,11^

###### ^1^Clinical Immunology and Allergy, The Royal Melbourne Hospital, Melbourne; ^2^Inflammation Division, The Walter and Eliza Hall Institute of Medical Research; ^3^Department of Medical Biology, The University of Melbourne, Parkville, Australia; ^4^Department of Rheumatology; ^5^Immunology Laboratory, Guangzhou Women and Children’s Medical Centre, Guangzhou, China; ^6^Department of Pediatrics, University Hospital and Faculty of Medicine Carl Gustav Carus, Dresden, Germany; ^7^Department of Chemical Biology, Guizhou Medical University, Guiyang, China; ^8^Division of Pediatric Stem Cell Transplantation and Immunology, University Medical Center Hamburg Eppendorf, Hamburg, Germany; ^9^Pediatric Rheumatology/RK Mellon Institute, Children’s Hospital of Pittsburgh ofUPMC, Pittsburgh, United States; ^10^Structural Biology Division, The Walter and Eliza Hall Institute of Medical Research, Parkville; ^11^Department of Medical Biology, The University of Melbourne, Melbourne; ^12^Rheumatology Department, The Royal Melbourne Hospital, Parkville, Australia; ^13^Department of Women’s & Children’s Health, nstitute of Translational Medicine, University of Liverpool; ^14^Department of Pediatrics Rheumatology, Alder Hey Children's NHS Foundation Trust Hospital, Liverpool, United Kingdom

####### **Correspondence:** Fiona Moghaddas

**Introduction:** Monogenic autoinflammatory disorders are characterised by dysregulation of the innate immune system. A significant number of this broadening group of disorders are caused by gain-of-function mutations in inflammasome forming proteins, such as NLRC4. A number of mutations in *NLRC4* have been described, leading to a spectrum of NLRC4-associated autoinflammatory disorders (NLRC4-AID).

**Objectives:** We studied two patients with early onset macrophage activation syndrome caused by the same *de novo* mutation in *NLRC4* (c.G1965C, p.W655C). Unlike other mutations in *NLRC4* described to date, p.W655 is located within the leucine rich repeat (LRR) domain. For this reason, we investigated mechanisms by which this mutation contributes to the pathogenesis of autoinflammatory disease.

**Methods:** Next generation and Sanger sequencing techniques were used for genetic analysis. ELISA was performed to quantify serum cytokine levels. *In vitro*, inflammasome complex formation was quantified using flow cytometric analysis of Apoptosis-associated Speck-like protein containing a Caspase recruitment domain (ASC) specks.Monocyte-like cell lines were generated by genetic deletion of *NLRC4* from THP-1 cells using CRISPR/Cas9 techniques followed by lentiviral transduction of wild type (WT) or mutant *NLRC4* cDNA. Cell death and release of IL-1b/IL-18 were quantified using flow cytometry and ELISA respectively.

**Results:** Both reported patients succumbed to macrophage activation syndrome early in life, associated with increased IL-18 serum levels. The *NLRC4* mutation identified, c.G1965C/p.W655C, caused increased ASC speck formation *in vitro*. In THP-1 cells, introduction of c.G1965C/p.W655C NLRC4 resulted in increased cell death, IL-1b and IL-18 production. The enhanced response was independent of NLRP3 and caspase-8. ASC contributed to p.W655C NLRC4 mediated cytokine release, but not cell death. p.W655 is located at the interface between adjacent LRR domains in the oligomeric inflammasome structure. Mutation of p.W655 activates the NLRC4 inflammasome complex by engaging with two interfaces on the opposing LRR domain. One key set of residues (p.D1010, p.D1011, p.L1012 and p.I1015) participates in LRR-LRR oligomerization when it is triggered by NLRC4-AID mutations or T3SS effector (PrgI) stimulation of the NLRC4 inflammasome complex.

**Conclusion:** This is the first report of a mutation in the LRR domain of NLRC4 causing NLRC4-AID. c.G1965C/p.W655C NLRC4 increases inflammasome activation, leading to constitutive IL-18 production and increased IL-1b release upon priming, where ASC contributes to the cytokine response, but not to cell death. Data generated from various *NLRC4* mutations suggests that the tryptophan at p.W655 does not tolerate substitution, and provides evidence that the LRR-LRR interface has an important, previously unrecognized role in oligomerization of the NLRC4 inflammasome complex.

**Disclosure of Interest**

None Declared

### Multifactorial vs monogenic autoinflammatory diseases

#### O05 Canakinumab, on a reduced dose or a prolonged dose interval without concomitant corticosteroids and methotrexate, maintains efficacy in systemic juvenile idiopathic arthritis patients in clinical remission

##### Pierre Quartier^1^, Ekaterina Alexeeva^2^, Carine Wouters^3^, Inmaculada Calvo^4^, Tilmann Kallinich^5^, Bo Magnusson^6^, Nico Wulffraat^7^, Xiaoling Wei^8^, Alan Slade^9^, Ken Abrams^9^, Alberto Martini^10^

###### ^1^AP-HP, Institut des Maladies Génétiques (IMAGINE), and Université Paris-Descartes, Necker-Enfants Malades Hospital, Paris, France; ^2^National Medical Research Center of Children's Health and Sechenov First Moscow State Medical University of the Ministry of Health of the Russian Federation, Moscow, Russian Federation; ^3^Gasthuisberg University Hospital,Leuven, Belgium; ^4^Hospital Universitario La Fe, Valencia, Spain; ^5^Charité Berlin Campus Virchow, Berlin, Germany; ^6^Karolinska University Hospital, Stockholm, Sweden; ^7^University Medical Center Utrecht, Utrecht, Netherlands; ^8^China Novartis Institutes for Biomedical Research Co., Ltd, Beijing, China; ^9^Novartis Pharmaceuticals Corporation, East Hanover, United States; ^10^Universita di Genova Pediatria II, Genova, Italy

####### **Correspondence:** Ekaterina Alexeeva

**Introduction:** Treatment with canakinumab (CAN), a selective, human anti-IL-1β monoclonal antibody, has shown sustained therapeutic effect along with corticosteroid dose reduction/discontinuation in patients with systemic juvenile idiopathic arthritis (SJIA), in a long-term extension study (NCT00891046).^1^

**Objectives:** To evaluate the efficacy and safety results from a study evaluating 2 different canakinumab tapering regimens in SJIA patients who were in clinical remission (NCT02296424).

**Methods:** This Phase 3b/4 study had two parts. In Part I, 182 patients with inactive disease from the extension study^1^ (cohort 1) and CAN-naïve patients (cohort 2) with active disease were administered subcutaneous CAN 4 mg/kg q4w. Per protocol titration off corticosteroids and/or methotrexate was attempted during Part I. Eligible patients (inactive disease for 24 consecutive weeks and being corticosteroid- and methotrexate-free for at least 4 weeks) advanced to Part II. Patients were randomised to either a 3-step CAN dose reduction regimen (2mg/kg/q4w, followed by tapering to 1 mg/kg/q4w and then discontinuation) or dose interval prolongation regimen (4mg/kg q8w, followed by tapering to 4 mg/kg/q12w and then discontinuation); patients advanced to the next tapering step if inactive disease was maintained for 24 weeks. The primary objective was to evaluate if at least 40% of patients were able to maintain inactive disease status for at least 24 consecutive weeks on either 2mg/kg q4w or 4mg/kg q8w.

**Results:** In Part II, a total of 75 patients were randomised to a dose reduction (n=38) or dose interval prolongation (n=37) in CAN tapering regimen. The proportion of patients who maintained inactive disease for 24 consecutive weeks exceeded the predefined threshold of 40% for Step 1 of: the reduced CAN dose (71%; 2 mg/kg q4w) and prolonged dose interval (84%; 4 mg/kg q8w) treatment arms. A total of 68% (26/38) and 79% (30/37) of the dose reduction and interval prolongation arms, respectively were successful in Step 2, while only 33% (25/75) of patients successfully discontinued CAN and maintained inactive disease for 24 consecutive weeks. Adverse events (AEs) and serious AEs observed within the 2 treatment cohorts and across Parts I and II were similar without any specific pattern or relationship to patients’ disease status at baseline or treatment regimen. The most frequent AEs were common infections such as nasopharyngitis, upper respiratory tract infection, and pharyngitis followed by SJIA-related events such as rash, pyrexia and arthralgia. Clinical laboratory abnormalities were consistent with expected findings in patients with active SJIA and the known safety profile of CAN.

**Conclusion:** SJIA patients who are able to maintain inactive disease status on CAN monotherapy can successfully taper CAN by either reducing the dose or prolonging the dosing interval. However, only a minority of patients successfully discontinued CAN treatment for 24 weeks. The safety profile for both CAN titration regimens was similar and consistent with other CAN SJIA studies. No new safety signals were identified.

**Reference**

1. Brunner et al. *Arthritis Rheumatol*.2016; 68 (S10).

**Disclosure of Interest**

P. Quartier Consultant for: Abbvie, Lilly, Novimmune, Novartis and SOBI,Speaker Bureau of: AbbVie, Lilly, Novartis and SOBI, E. Alexeeva Grant / Research Support from: Roche, Abbott, Pfizer, Bristol-Myers Squibb, Centocor, Novartis, C. Wouters Consultant for: GSK, Roche, Pfizer , I. Calvo: None Declared, T. Kallinich Speaker Bureau of: Novartis, B. Magnusson: None Declared, N. Wulffraat Consultant for: Novartis, X. Wei Employee of: Novartis, A. Slade Conflict with: Novartis Pharmaceuticals CorporationShareholder of: Novartis Pharmaceuticals Corporation,Employee of: Novartis Pharmaceuticals Corporation, K. Abrams Conflict with: Novartis Pharmaceuticals CorporationShareholder of: Novartis Pharmaceuticals Corporation,Employee of: Novartis Pharmaceuticals Corporation, A. Martini: None Declared

#### O06 IL-18:CXCL9 ratio as a predictor of treatment response in patients with systemic juvenile idiopathic arthritis treated with canakinumab

##### Tanja Hinze^1^, Christoph Kessel^1^, Claas Hinze^1^, Julia Seibert^2^, Hermann Gram^2^, Dirk Foell^1^

###### ^1^Department of Pediatric Rheumatology and Immunology, Muenster University Hospital, Muenster, Germany; ^2^Novartis Pharma, Basel, Switzerland

####### **Correspondence:** Tanja Hinze

**Introduction:** Canakinumab, a monoclonal anti-interleukin (IL)-1β antibody, is highly effective for treating patients with systemic juvenile idiopathic arthritis (SJIA) but biomarkers predicting treatment response are desirable.

**Objectives:** The objective of this study was to analyze the association of various serum biomarkers with treatment outcomes.

**Methods:** Serum samples from 54 patients treated with canakinumab in an open-label long-term extension study were studied by Luminex at different time points during the study, including days 1 (baseline), 3, 15 and weeks 4, 8, 24, 48. Treatment outcomes included modified pediatric American College of Rheumatology (pACR) 30/50/70/90/100 responses within 15 days of treatment, clinically inactive disease (CID) according to the Wallace criteria within 15 days of treatment and sustained complete response, defined as pACR100or CID within 15 days of treatment plus no disease flare or macrophage activation syndrome (MAS) during the study. Data were analysed using non-parametric testing and receiver operating characteristic (ROC) analysis.

**Results:** Within 15 days of treatment with canakinumab, 79%/77%/68%/49%/34% of the patients reached a modified pACR 30/50/70/90/100 response and 34% CID. Within a median follow-up of 23 months, 12 of 54 (22%) patients had a sustained complete response and 5 (9%) had developed MAS. Biomarkers did not correlate significantly with age, duration of disease or active joint count. Most biomarkers were elevated when compared to healthy controls at baseline and some rapidly decreased within 15 days of therapy (IL-1RA, IL-6, IL-18 and S100A12). A pattern was apparent when comparing responders and non-responders; responders had higher IL-18 and IFN-γ levels and lower CXCL9 levels at baseline, most emphasized by the IL-18:CXCL9 and the IFN-γ:CXCL9 ratios (Table 1). As determined via ROC analyses, these ratios had good accuracy in predicting treatment responses relating to pACR30/50/70/90/100/CID responses at day 15 and sustained complete response (area under the curve [AUC] for IL-18:CXCL9 ratio 0.79/0.75/0.67/0.72/0.77/0.71 and 0.80, respectively; and for the IFN-γ:CXCL9 ratio 0.79/0.76/0.71/0.75/0.77/0.76 and 0.79). Higher baseline CXCL9 levels predicted future MAS during the course of the study (ROC analysis AUC = 0.77).

**Conclusion:** Several serum biomarkers were markedly elevated in patients with SJIA at baseline. A dysregulation of the IL-18-IFN-γ-CXCL9 axis is present in patients with SJIA, confirming findings from other investigators. However, our findings indicate that patients with a lower response to IL-18 and IFN-γ, as measured by CXCL9, an IFN-γ-induced chemokine, may have a better clinical response to canakinumab treatment. These findings will have to be confirmed in other cohorts.

**Disclosure of Interest**

T. Hinze Grant / Research Support from: Study was funded by Novartis Pharma , C. Kessel Grant / Research Support from: Study was funded by Novartis Pharma, C. Hinze Grant / Research Support from: Study was funded by Novartis Pharma, J. Seibert Employee of: Novartis Pharma, H. Gram Employee of: Novartis Pharma, D. Foell Grant / Research Support from: Study was funded by Novartis Pharma

**Table 1 (abstract O06).** Median (range) IFN-gamma: CXCL9 and IL-18:CXCL9 ratios at baseline

	IL-18:CXCL9 ratio at baseline			IFN-γ:CXCL9 ratio at baseline		
	Responders	Non-Responders	*P* value*	Responders	Non-Responders	*P* value*
pACR30 at day 15	2.54 (0.03-557.31)	0.34 (0.01-1.62)	0.004	5.27 (0.19-68.32)	0.82 (0.24-4.85)	0.003
pACR50 at day 15	2.37 (0.03-557.31)	0.36 (0.01-3.76)	0.010	5.23 (0.19-68.32)	1.35 (0.24-5.38)	0.007
pACR70 at day 15	2.54 (0.03-557.31)	0.57 (0.01-42.27)	0.052	5.27 (0.19-68.32)	1.56 (0.24-20.03)	0.015
pACR90 at day 15	3.69 (0.03-557.31)	0.57 (0.01-42.27)	0.006	7.51 (0.19-68.32)	1.88 (0.19-20.03)	0.002
pACR100 at day 15	3.99 (0.05-557.31)	0.57 (0.01-42.27)	0.001	8.35 (0.19-68.32)	1.97 (0.19-43.46)	0.002
CID at day 15	3.84 (0.03-557.31)	0.66 (0.01-42.81)	0.015	8.34 (0.62-68.32)	1.97 (0.19-41.52)	0.002
Sustained complete response	5.52 (0.03-557.31)	0.65 (0.01-277.69)	0.002	8.35 (0.62-68.32)	2.01 (0.19-43.46)	0.002

*Mann-Whitney U test

### Update of autoinflammatory diseases

#### O07 The NIH cohort study of DADA2 patients: novel insights into pathophysiology and treatment with TNF inhibitors

##### Qing Zhou^1,2^, Natalie Deuitch^1^, Dan Yang^3^, Natalia Sampaio Moura^1^, Xiaomin Yu^4^, Oskar Schnappauf^1^, Patrycja Hoffmann^1^, Deborah Stone^1^, Amanda Ombrello^1^, Manfred Boehm^3^, Daniel Kastner^1^, Ivona Aksentijevich^1^

###### ^1^NHGRI/NIH, Bethesda, United States; ^2^Zhejiang University, Hang Zhou, China; ^3^NHLBI/NIH; ^4^NIAID/NIH, Bethesda, United States

####### **Correspondence:** Qing Zhou

**Introduction:** Deficiency of Adenosine Deaminase 2 (DADA2) is a recessively inherited disorder caused by a loss of functional ADA2 protein. A broad spectrum of features, including systemic inflammation, cutaneous, neurologic, musculoskeletal, and immunological manifestations have now been associated with DADA2. Given the highly polymorphic nature of the *ADA2* and the complex clinical presentations of DADA2, large cohort studies are critical to advancing our knowledge of ADA2’s role in disease manifestation and pathogenesis.

**Objectives:** To expand on the genetics of patients with DADA2 and explore the pathophysiology and the underlying mechanisms of TNF inhibitor response in these patients.

**Methods:** We performed Sanger sequencing of the *ADA2* gene (previously known as *CECR1)* and measured ADA2 enzyme activity in serum samples of patients and family members. We used flow cytometry, intracellular cytokine staining, transcriptome analysis, immunohistochemistry and cell differentiation experiments to define an inflammatory signature in DADA2 patients and studied their response to TNF inhibitor treatment.

**Results:** We have identified 60 patients with DADA2 and detected 10 pathogenic variants, which had not previously been associated with DADA2 – p.R9W, p.R34W, p.W204C, p.E244A, p.D329N, p.L351Q, p.A357T, p.P425A, p.P435A, p.W501*.Patients tested for ADA2 enzymatic activity had significantly decreased protein activity. Symptoms were highly variable among patients, even in cases with identical genotypes.

We identified distinct inflammatory signatures. Patients had significantly higher subsets of CD14+ inflammatory monocytes than healthy controls. Phosphorylation of STAT1 was upregulated in patients CD4+ cells and monocytes following stimulation. Additionally, we observed increased gene expression of IP-10 in patients’ monocytes and macrophages. Furthermore, we observed strong NF-κB signaling in patients. Intracellular cytokine staining and qPCR for IL-1β, IL-6, and TNF cytokines were elevated in patients’ monocytes.

After TNF inhibitor treatment, IFN and NF-κB inflammatory signatures were normalized. Analyses of post-treatment skin, lung and brain biopsies showed minimal perivascular TNF and resolution of inflammatory myeloid cell infiltrates. Immunostaining of skin biopsies revealed intact blood vessels with normal endothelial layers after treatment. Together, the data provides evidence that TNF inhibition both reduces systemic inflammation and improves endothelial integrity in the small vessels.

ADA2 deficiency affects the differentiation of monocytes to anti-inflammatory M2 macrophages. Treatment with TNF-inhibitors rescued the impairment of M2 differentiation as demonstrated by improved cell morphology and a higher number of M2 macrophages.

**Conclusion:** We report 10 novel pathogenic variants in ADA2, which is valuable for future DADA2 molecular diagnostic. Most important, we showed the cellular mechanism underlying effective treatment with anti-TNF therapies. DADA2 vasculitis is strongly related to the presence of activated myeloid cells and is reversible.

**Disclosure of Interest**

None Declared

#### O08 Recommendation on colchicine dosing and definition of colchicine resistance/intolerance in the management of FMF

##### Seza Ozen^1^, Erdal Sag^1^, Eldad Ben-Chetrit^2^, Marco Gattorno^3^, Ahmet Gul^4^, Philip Hashkes^5^, Isabelle Kone-Paut^6^, Helen Lachmann^7^, Elena Tsitsamis^8^, Marinka Twilt^9^, Fabrizio de Benedetti^10^, Jasmin B. Kuemmerle-Deschner^11^

###### ^1^Pediatric Rheumatology, Hacettepe University, Ankara, Turkey; ^2^Rheumatology Unit, Hadassah-Hebrew University Hospital, Jerusalem, Israel; ^3^Clincal Pediatrics and Rheumatology, Gaslini Institute, Genova, Italy; ^4^Department of Internal Medicine, Division of Rheumatology, Istanbul University, Istanbul, Turkey; ^5^Pediatric Rheumatology Unit, Department of Pediatrics, Shaare Zedek Medical Center, Jerusalem, Israel; ^6^Service de rhumatologie pédiatrique, CHU de Bicêtre, Le Kremlin-Bicêtre, France; ^7^Royal Free Campus, National Amyloidosis Centre, London, United Kingdom; ^8^First Department of Pediatrics, Aghia Sophia Childrens Hospital, University of Athens Medical School, Athens, Greece; ^9^Rheumatology, Department of Pediatrics, Alberta Children’s Hospital, University of Calgary, Calgary, Canada; ^10^Division of Rheumatology, Ospedale Pediatrico Bambino Gesù, Rome, Italy; ^11^Department of Pediatrics, Division of Pediatric Rheumatology, University Hospital Tuebingen, Tuebingen, Germany

####### **Correspondence:** Jasmin B. Kuemmerle-Deschner

**Introduction:** FMF is the most common monogenic autoinflammatory disease and colchicine is the drug of choice for the treatment. However, about 5-10% of FMF patients do not respond to colchicine even when they are fully compliant. Anti-IL1 treatments are used for the patients who are resistant to colchicine. Treatment with IL-1 inhibitors have been shown to be effective in clinical trials and in several case series.

**Objectives:** The objective of this report is to produce evidence-based recommendations to define “colchicine resistance“, as well as compliance and intolerance, to guide rheumatologists and other health professionals in the treatment and follow-up of patients with colchicine-resistant FMF.

**Methods:** A consensus meeting with 12 experts followed a systemic literature review and Delphi questionnaire. The expert committee consisted of adult and pediatric rheumatologists with expertise in FMF. Parameters for colchicine resistance/intolerance/compliance derived from the literature were evaluated by a pre-meeting online questionnaire. All parameters were discussed with a nominal group technique during the meeting. Recommendations were accepted if more than 80% agreement was reached. If agreement was below 80% a second round of discussion was held.

**Results:** The systematic literature review yielded 264 articles. Of these, 38 were selected for expert review. After the literature review, Delphi survey, and round table discussion, recommendations that reached consensus levels were:

Colchicine is the drug of choice for the treatment of FMF and compliance is a critical issue. For the following statements, it is assumed that the patient is compliant with colchicine.

When utilizing colchicine to treat FMF, it is recommended to adjust the dose based on disease activity with the maximal dose in children depending on age (and weight).

The maximum recommended colchicine dose for the treatment of FMF is between 1-3 mg per day depending on age, limited by signs of toxicity and tolerability.

For a patient receiving the maximum tolerated dose of colchicine, resistance to colchicine is defined as ongoing disease activity (as reflected by either recurrent clinical attacks (average one or more attacks per month over a three-month period), or persistently elevated CRP or SAA in between attacks (depending on which is available locally)), in the absence of any other plausible explanation.

AA amyloidosis develops as a consequence of persistent inflammation, which may be a manifestation of colchicine resistance.

Colchicine intolerance, which generally manifests as GI symptoms (such as diarrhea and nausea), is common and can limit the ability to achieve or maintain the effective dose. Dose-limiting toxicity is rare and may include elevated LFT, leukopenia, azoospermia etc.

Active disease and intolerance to colchicine affect quality of life.

Various patient reported outcomes to be used to guide FMF disease management were outlined.

**Conclusion:** The suggested recommendations are intended to improve patient care in FMF, to make a personalized treatment plan.

**Disclosure of Interest**

S. Ozen Speaker Bureau of: Novartis, SOBI, Pfizer, E. Sag: None Declared, E. Ben-Chetrit: None Declared, M. Gattorno: None Declared, A. Gul: None Declared, P. Hashkes Grant / Research Support from: Novartis,Consultant for: Novartis, I. Kone-Paut: None Declared, H. Lachmann: None Declared, E. Tsitsamis: None Declared, M. Twilt: None Declared, F. de Benedetti: None Declared, J. Kuemmerle-Deschner Grant / Research Support from: Novartis, SOBI,Consultant for: Novartis, SOBI

### Oral communications – clinical

#### O09 The clinical spectrum of the deficiency of adenosine deaminase 2 (DADA2) continues to expand

##### Karyl Barron^1^, Amanda Ombrello^2^, Debra Stone^2^, Patrycja Hoffmann^2^, Tina Romeo^2^, Anne Jones^2^, Natalia Sampaio Moura^2^, Oskar Schnappauf^2^, Ivona Aksentijevich^2^, Jenna Bergerson^1^, Ariane Soldatos^3^, Camilo Toro^2^, Dan Kastner^2^

###### ^1^NIAID; ^2^NHGRI; ^3^NINDS, NIH, Bethesda, United States

####### **Correspondence:** Karyl Barron

**Introduction:** The original reports of the deficiency of adenosine deaminase 2 (DADA2) in 2014 emphasized early-onset lacunar strokes, livedoid rash, intermittent fevers and early-onset polyarteritis nodosa. Since then, there have been reports of antibody deficiency, pure red cell aplasia, and cytopenias observed in DADA2 patients.

**Objectives:** To document the range of clinical manifestations of DADA2.

**Methods:** 46 patients were enrolled in an IRB approved study at the NIH. Sequencing of *ADA2,* the gene encoding ADA2 (adenosine deaminase 2), was performed on all patients. All underwent extensive clinical, laboratory & radiologic evaluation.

**Results:** We evaluated 46 patients with DADA2 (24 F/22 M) including 6 sibling pairs & 2 families with 3 affected individuals. All patients had biallelic germline mutations in *ADA2*. Serum ADA2 enzyme activity levels were obtained in 32 patients and revealed absent to low levels compared to age-matched controls.

The 46^th^patient, seen 5 years after bone marrow transplantation for presumed GATA2 deficiency, was not included in our summary calculations.

32 patients (71%) reported a history of recurrent fevers. 6 patients (13%) had diffuse lymphadenopathy.

Skin involvement was seen in 38 patients (84%) including livedo in 34 (76%), cutaneous polyarteritis nodosa in 27 (60%), and Raynaud’s in 9 (20%).

22 patients (49%) had a history of at least one stroke.Brain MRI showed evidence of ischemic infarcts in l6/22 (73%), 5 had both ischemic & hemorrhagic strokes (23%) and 1 had a hemorrhagic stroke (5%). There were 55 strokes in the 22 patients, the majority occurring in the brain stem and cerebellum (38%) and deep brain nuclei (36%). The average age at the time of the first stroke was 5.6 years (range 5 months-20 years). Stroke patients had an average of 3 strokes (range of 1-11). 3 patients manifest severe sequelae of hemorrhagic strokes.

Abdominal ultrasound revealed hepatomegaly in 20 patients (44%) & splenomegaly in 26 (58%). Portal hypertension was observed in 7 patients (16%). Liver biopsies revealed hepatoportal sclerosis in 5 patients and focal nodular regenerative hyperplasia in 2. Abdominal MRA was abnormal in 7/13 patients, revealing arteritis and aneurysms.

Significant peripheral vasculopathy was seen in 4 patients, one requiring multiple amputations of gangrenous digits. Systemic hypertension was observed in 11 patients (24%).

Laboratory evaluation revealed hypogammaglobulinemia in 26 patients (58%). Immunoglobulin replacement was required in 10 patients. Lymphocyte phenotyping revealed arrested B cell class switching in 24/34 patients (71%) and decreased memory T cells in 11/34 (32%). Severe hematologic abnormalities, including anemia, leukopenia, lymphopenia and/or thrombocytopenia in 18 patients (40%), with 6 developing pancytopenia and 3 pure red cell aplasia. Immune mediated neutropenia was observed in 12 patients. ESR and C-reactive protein were elevated in 73% & 86%, respectively.

6 patients underwent bone marrow transplantation with 4 patients successfully engrafted (2 requiring a second transplant), and 2 recently transplanted.

**Conclusion:** The spectrum of DADA2 continues to expand to include ischemic and hemorrhagic strokes, cutaneous findings, portal and systemic hypertension, hematologic abnormalities, vascular pathology, immune deficiency and bone marrow failure. As the phenotypic presentation is likely to continue to expand, it is important to investigate any new complaints.

**Disclosure of Interest**

None Declared

#### O10 Serum S100A8/A9 (calprotectin) in familial mediterranean fever and carriers of MEFV mutations does not correlate with disease activity

##### Ruth Pepper^1^, Mathew Hutchinson^2^, Scott R. Henderson^3^, Sarah K. Todd^3^, Alan D. Salama^3^, Philip N. Hawkins^4^, Dorota Rowczenio^4^, Helen J. Lachmann^4^

###### ^1^Centre for Nephrology, UCL; ^2^Rheumatology, University College Hospital; ^3^Centre for Nephrology; ^4^National Amyloidosis Centre, UCL Division of Medicine and Royal Free Hospital NHS Foundation Trust, London, United Kingdom

####### **Correspondence:** Ruth Pepper

**Introduction:** Familial Mediterranean Fever (FMF) is caused by mutations in MEFV. The protein product pyrin is expressed in monocytes, neutrophils and eosinophils. Acute inflammatory attacks are accompanied by a dramatic hepatic acute phase response. S100A8/A9 is damage associated molecular pattern and a TLR4 ligand expressed in neutrophils, monocytes and early infiltrating macrophages.

**Objectives:** We aimed to investigate S100A8/A9 in 39 patients with FMF, 45 healthy carriers and 16 wild type controls.

**Methods:** All patients were genotyped. Patients and healthy controls (HC) serum S100A8/A9 levels, cell surface expression on monocytes and neutrophils as well as intracellular peripheral blood mononuclear cells (PBMC) expression were measured by flow cytometry (FACS). CD14 cells were isolated and following overnight incubation with or without LPS, S100A8/A9 was measured in the supernatants by ELISA. Patient and HC monocyte apoptosis was compared.

**Results:** Serum levels were measures in 84 samples from 31 patients with homozygous or compound mutations (median 9061ng/ml [range 500-38470], 79 samples from 39 symptomatic patients who were MEFV heterozygotes (median 9394ng/ml [range 1744-38119], 80 samples from 45 individuals with MEFV variants but without clinical features of FMF (median 10939ng/ml [range 2447->40000]. There was no difference in calprotectin concentrations between the different mutations and no correlation with levels of the hepatic acute phase response, CRP or SAA. All the groups described had significantly higher levels than healthy controls (n=16 median 2836ng/ml [range 1058-6175])(p<0.001). Minimal monocyte and neutrophil cell surface expression was detectable. Following LPS stimulation there was significantly more S100A8/A9 detected in the supernatants in patients than healthy control CD14. There was also a trend to an increased intracellular monocyte S100A8/A9 expression.

**Conclusion:** Patients with pyrin mutations both with and without clinical disease have greatly elevated serum S100A8/A9 levels without detectable cell surface expression in well-controlled disease with a trend to an increased monocyte intracellular expression. Upon monocyte stimulation with LPS, increasedS100A8/A9 is secreted. The exact mechanism by which these patients, especially those with mutations but no clinical disease, demonstrate sustained elevated serum S100A8/A9 remains to be elucidated but does not appear to result in a significant clinical sequelae.

**Disclosure of Interest**

None Declared

#### O11 PAPA syndrome: novelties from the Eurofever registry

##### Roberta Caorsi, Daniela Marotto, Antonella Insalaco, Angelo Marzano, Joost Frenkel, Graciela Espada, Immaculada Calvo Penades, Marijia Jelusic, Maria Cristina Maggio, Joost Swart, Esther Hoppenreijs, Ozgur Kasapcopur, Fabrizio De Benedetti, Marco Gattorno, The Pediatric Rheumatology International Trial Organization (PRINTO) and the Eurofever Project

###### Center for Autoinflammatory Diseases and Immunodeficiency, Istituto G. Gaslini, Genova, Italy

####### **Correspondence:** Roberta Caorsi

**Introduction:** PAPA syndrome is a very rare autoinflammatory condition. Few data are nowadays available about the clinical characteristic, the response to treatment and the outcome of this disease.

**Objectives:**To analyse the data of the PAPA patients enrolled to the Eurofever registry.

**Methods:** the data analysed in the study were extracted from the Eurofever registry, which is hosted in the PRINTO website (www.printo.it). The patients were included in the study in the presence of mutations in the PSTPIP1 gene or, in genetically negative patients, in the presence of at least two of the following clinical manifestation: recurrent pyogenic arthritis, pyoderma gangrenousm or skin abscess with negative cultural tests. Demographic data, clinical manifestations and response to treatment were analysed.

**Results:** In may 2018 baseline and clinical information were available of near 4000 patients in the Eurofever registry. Of the 36 patients classified as PAPA syndrome, 2 were excluded from the study. 34 PAPA patients, from 11 different centers, were analysed: the genotype was confirmatory in 29 patients, while in 5 was not available. 10 patients were of the same family, in 4 cases one parent was affected (2 included in the registry), while in other 8 patients the family history was negative. At the time of enrolment, 15 patients were in the paediatric age, while 19 were adults. The mutations detected in the PSTPIP1 gene were E250Q (13 pts), E250K (5 pts), A230T (3 pts), G258A (3 pts), E277D (2 pts), E257G (1 pt), G940A (1pts) and R365W (1 pts).

The disease course was recurrent in 24 patients, while the other 10 presented a chronic disease course with periodic recrudesces. Joint and skin involvement were present at disease onset in 24 and 9 patients respectively. In other 12 patients skin involvement appeared over time. 20 out of the 34 patients presented clinical manifestations not typical of PAPA syndrome (psoriasis, uveitis, osteolytic bone lesions, chronic renal failure, muscular abscesses, gastrointestinal symptoms anaemia and hepatosplenomegaly).

10 patients were treated with NSAID with partial and poor response in 6 and 4 patients respectively, while steroids caused a complete or partial control of disease manifestations in 6 and 10 patients respectively. Five patients were treated with methotrexate with partial response. Etanercept was used in 6 patient with complete response in 2 and partial in 4, adalimumab in 4 patients (1 partial and 1 complete responders, 2 failure) and anakinra in 9 patients (3 partial and 6 complete responders). 2 patients were treated with Canakinumab with complete response.

**Conclusion:** This study enlightens the phenotypic variability of PAPA syndrome. The unusual clinical manifestations and the lack of the clinical triad of the disease may be responsible for the under recognition of this disease. Between biologic drugs, IL-1 inhibitors were more effective in the analysed cohort of patients.

**Disclosure of Interest**

None Declared

#### O12 New classification criteria for recurrent autoinflammatory diseases applied to an independent cohort: experience from the JIR cohort database

##### Glory Dingulu^1^, Sophie Georgin-Lavialle^2^, Isabelle Koné-Paut^3^, Pascal Pillet^4^, Anne Pagnier^5^, Etienne Merlin^5^, Daniela Kaiser^6^, Alexandre Belot^7^, Michael Hofer^8^, Véronique Hentgen^9^

###### ^1^Centre Hospitalier Versailles, Le Chesnay; ^2^Service de Médecine Interne-CEREMAIA, Centre Hospitalier Universitaire Tenon, Paris; ^3^Service de Rhumatologie Pédiatrique-CEREMAIA, Centre Hospitalier Universitaire Le Kremlin Bicêtre, Le Kremlin Bicêtre; ^4^Service d’Accueil des Urgences Pédiatriques, Centre Hospitalier Universitaire Pellegrin, Bordeaux; ^5^Service de Pédiatrie Générale, Centre Hospitalier Universitaire Clermont Ferrand, Clermont Ferrand, France; ^6^Service de Pédiatrie Générale, Centre Hospitalier Cantonal Luzern, Luzern, Switzerland; ^7^Service de Néphrologie-Rhumatologie Pédiatrique, Centre Hospitalier Universitaire Mère-Enfant, Bron, France; ^8^Service de Rhumatologie Pédiatrique, Centre Hospitalier Universitaire Vaudois, Lausanne, Switzerland; ^9^Service de Pédiatrie Générale-CEREMAIA, Centre Hospitalier Versailles, Le Chesnay, France

####### **Correspondence:** Glory Dingulu

**Introduction:** New classification criteria for the inherited periodic fever syndromes Cryopyrin Associated Periodic Syndrom (CAPS), Tumour Necrosis Factor Receptor Associated Periodic Syndrom (TRAPS), Familial Mediterranean Fever (FMF), Mevalonate Kinase Deficiency (MKD), have recently been developed during a Consensus Conference held in Genoa in March 2017.

**Objectives:** The aim of our study was to compare these new classification criteria for monogenic recurrent fever syndromes with the diagnoses of clinicians in a real-life setting.For this purpose we used the JIRcohort database, an international platform gathering data of patients with pediatric inflammatory disease.

**Methods:** Patients with recurrent fever syndrome and complete clinical and genetic data were enrolled to the study from the auto-inflammatory module of the JIRcohort database. Patients genotype were characterized with HRF pathogenicity classification. A score from 0 to 2, 0 (no mutation), 1 (non-confirmatory genotype) and 2 (confirmatory genotype) was attributed to each gene screened in one patient. The new Genoa classification criteria were applied to all the patients and then compared to the diagnosis of the treating physician. The treating physician diagnosis was considered as standard reference. If the treating physician hesitated between two or more diagnoses, patients were redefined as Syndrome of Unexplained Recurrent Fever (SURF). SURF and PFAPA patients were pooled together. Finally, for each criteria sensitivity and specificity were determined before an analytical study, describing true positive, false positive and false positive patients.

**Results:** 455 patients were included:10 CAPS, 11 MKD, 27 TRAPS, 122 FMF and 285 SURF/PFAPA patients.

CAPS classification criteria showed a 60 % sensitivity and 98% specificity. 6 patients were true positive patients with confirmatory and non-confirmatory *NLRP3* genotype. 4 were false negative patients with non-confirmatory genotype or no mutation in NLRP3. 8 were false positive with no mutation in *NLRP3*.

TRAPS classification criteria showed a 100 % sensitivity and 98% specificity. 22 were true positive patients with confirmatory and non-confirmatory *TNFRSF1A* genotype. 5 patients were false positive with non-confirmatory genotype or no mutation in *TNFRSF1A*.

FMF classification criteria showed a 96 % sensitivity and 88% specificity. 117 patients were true positive with confirmatory, non-confirmatory and non mutated *MEFV* genotype. 5 were false negative with non-confirmatory and non mutated genotype. 37 werefalse positive patients with patients with non-confirmatory genotype or non mutated genotype or no *MEFV* screening.

MKD classification criteria showed a 64 % sensitivity and 66% specificity. 7 patients were true positive patients with confirmatory MVK genotype.4 were false negative patientswith non-confirmatory genotype or non mutated MVK genotype. 148 were false positive patients with non mutated MVK genotype.

**Conclusion:** This work is the first to study Genoa criteria, in real-life setting, in a cohort of patients seen with recurrent fever. Genoa criteria showed tremendous performance for patients with confirmatory genotype and helped classifying patients with non-confirmatory genotype.

Genoa classification criteria were less effective when patients did not display at least one genetic variant. Implementation of biological criteria in MKD might improve MKD criteria performance.

The major limit of our study is the lack of a proper gold standard when genotype is not confirmatory. Nevertheless our study shows that the new classification criteria are of a high risk of misclassification in patients displaying a recurrent fever syndrome without genetic test.

**Disclosure of Interest**

None Declared

#### O13 Impaired platelet functions in patients treated with colchicine

##### Özlem Çimen^1^, Selcan Demir^2^, Erdal Sağ^2^, Armağan Keskin^1^, Yelda Bilginer^2^, Şule Ünal Cangül^3^, Seza Özen^2^

###### ^1^Department of Pediatrics; ^2^Department of Pediatric Rheumatology; ^3^Department of Pediatric Hematology, Hacettepe University Medical Faculty, Ankara, Turkey

####### **Correspondence:** Selcan Demir

**Introduction:** Colchicine has been used in the treatment of Familial Mediterranean Fever (FMF) since 1972. Apart from the inhibiting mitosis in all cells, colchicine has an anti-inflammatory effect by inhibiting activation and migration of neutrophils. Colchicine is a safe drug at recommended doses, but it can cause rare side effects including hematological findings such as lymphopenia, thrombocytopenia and neutropenia.

**Objectives:** In this study we aimed to define the adverse effect of colchicine on platelet function and its clinical relevance.

**Methods:** A total of 220 FMF patients between June 2016-2017, followed at Hacettepe University Pediatric Rheumatology Department and were on colchicine treatment for at least one year, were included to the study.

**Results:** Among the selected 220 FMF patients, 100 of them (54% female) described hematological symptoms when questioned in detail. The mean age of these patients was 11.74 ± 4.86 years. The mean cumulative colchicine exposure was 5.7±3.8 years.

The most common referral symptom was frequent epistaxis (79%) followed by easy bruising (69%), and menstrual disorder including prolonged or heavy menstrual bleeding (21.8% among female patients). Among these 100 patients, 36 of them had prolonged bleeding time and impaired platelet aggregation test. Patients who had abnormal platelet function tests (the group with abnormal bleeding time) were receiving higher colchicine doses (median 0.05 vs 0.03 mg/kg/day; p:0.001) compared to the patients who had normal platelet function tests (bleeding time normal group) However there were no significant difference in terms of cumulative colchicine exposure (median 6.5 vs 4.5 years; p:0.07) and total platelet counts (median 288500 vs 279000/mm3; p:0.61). Patients with abnormal platelet function tests (bleeding time abnormal group) also had more epistaxis (47% vs 7%; p<0.001), bruising (51% vs 3%; p<0.001) and dysmenorrhea (among female patients 100% vs 26%; p<0.001) (Figure 1 and 2). Colchicine was not reduced in these patients and no life-threatening event was observed.

**Conclusion:** In our study, we have shown prolonged bleeding time for the first time in the literature. Colchicine may cause microtubule inhibition in platelets as well as in other cells and impair platelet function. Further prospective studies are needed to clarify the significance of this side effect.

**Disclosure of Interest**

None Declared

#### O14 Autoinflammatory disorders in patients with myelodysplastic syndrome: the role of distinctive karyotypes and somatic mutations

##### Mark Kacar^1^, Abdulla Watad^2^, Nicola Bragazzi^3^, Qiao Zhou^2^, Catherine Cargo^4^, Dennis McGonagle^2^, Sinisa Savic^2^

###### ^1^Department of Clinical Immunology and Allergy, St James University Hospital; ^2^LIRMM, University of Leeds, Leeds, United Kingdom; ^3^Department of Health Sciences, University of Genoa, Genoa, Italy; ^4^Department of Haematology, St James University Hospital, Leeds, United Kingdom

####### **Correspondence:** Mark Kacar

**Introduction:** A higher prevalence of autoimmune and autoinflammatory complications has been reported in patients with myelodysplastic syndrome (MDS). The exact cause of this remains to be elucidated.

**Objectives:** To determine the correlation between patients’ demographic, clinical and molecular (karyotype, somatic genetic mutation status) features of MDS with specific autoinflammatory complications and long-term outcome.

**Methods:** This was a retrospective study of 140 MDS patients referred to the Haematological Malignancy Diagnostic Service (HMDS) in Leeds, UK, between 2012-2018. As part of their diagnostic workup, all patients had karyotype assessment and targeted genetic sequencing performed. Patients’ medical records were examined to collect demographic and clinical information, and to identify patients with autoinflammatory complications. Patients were classified as having ‘non-specific autoinflammatory features’ if CRP was found to be elevated (>10.0 mg/L) on 5 or more separate occasions and this elevation could not be explained by infection, malignancy or autoimmunity. Chi-squared test, Student t-test, analysis of variance (ANOVA), univariate and multivariate logistic regression analyses were performed.

**Results:** The average age was 77.08±11 years (median 79 years), with a male (n=91, 65.0%) preponderance. The 72 patients who had non-specific autoinflammatory features (51%) tended to be younger (75.15±11.23 years *versus* 79.15±11.92, p=0.0395), and more frequently had arthritis (n=25, 34.7%, *versus* n=12, 17.6%, p=0.0225), arthralgia (n=32, 44.4%, *versus* n=18, 26.5%, p=0.0271), skin rash (n=22, 30.6%, *versus* n=10, 14.7%, p=0.0261), and pleuritis (16, 22.2%, *versus* n=3, 4.4%, p=0.0022). 26.4% of MDS patients had a well-defined diagnosis of autoinflammatory disorder, with neutrophilic dermatosis and polymyalgia rheumatic occurring most commonly. Mutations affecting the transcription factor pathway (NPM1, RUNX1, BCOR, WTI, TP53, MYD88) (OR 3.15 [95%CI 1.04-9.56], p=0.0426) and deletion of chromosome 5 (OR 3.37 [95%CI 1.01-11.22], p=0.0479) were associated with autoinflammatory complications in general. Stratifying the patients into a “well-defined” and “non-specific” autoinflammatory disease group showed that deletion of chromosome 7 was associated with well-defined conditions, whilst deletion of chromosome 5 was linked with a non-specific autoinflammatory status. Furthermore, a higher rate of acute leukaemia transformation was reported in MDS patients with autoinflammatory status (n=25, 34.7%, *versus* n=8, 11.8%, p=0.0002).

**Conclusion:** Autoinflammatory conditions were found to be more prevalent than expected in patients with MDS and were linked to a worse prognosis. Transcription factor pathway gene mutations and an abnormal karyotype were also associated with autoinflammation. Autoinflammatory features were associated with malignant transformation, hinting at the possibility that treatment of the autoinflammation might play a role in preventing disease progression. Further studies are required to replicate our findings and study the effect of anti-inflammatory therapy on disease progression.

**Disclosure of Interest**

None Declared

**Table 1 (abstract O14).** See text for description

	Predictor	OR [95%CI]	Statistical significance
Overall autoinflammation	Transcription factor pathway	3.15 [95%CI 1.04 to 9.56]	0.0426
	Deletion of chromosome 5	3.37 [95%CI 1.01 to 11.22]	0.0479
Well-defined autoinflammatory disease	Transcription factor pathway	4.50 [95%CI 1.04 to 19.47]	0.0441
	Deletion of chromosome 7	6.13 [95%CI 1.16 to 32.33]	0.0325
	Number of mutations	3.39 [95%CI 1.08 to 10.66]	0.0441
Unspecified inflammatory state	Deletionof chromosome 5	3.57 [95%CI 1.02 to 12.48]	0.0465

#### O15 A novel MEFV mutation associated with an autosomal dominant FMF complicated by AA amyloidosis in a large British kindred

##### Dorota Rowczenio, Taryn Youngstein, Hadija Trojer, Charalampia Papadopoulou, Tamer Rezk, Philip Hawkins, Helen Lachmann

###### National Amyloidosis Centre, UCL, London, United Kingdom

####### **Correspondence:** Dorota Rowczenio

**Introduction:** Hereditary systemic autoinflammatory diseases (SAIDs) are rare genetic disorders characterised by recurrent, spontaneously resolving episodes of fever and systemic inflammation that predominantly involves serosal surfaces. AA amyloidosis is the most serious complication of SAIDs and is associated with proteinuric renal dysfunction that progresses to end stage renal failure and premature death.

**Objectives:** To find a genetic cause in a large British family with a dominantly inherited autoinflammatory syndrome complicated by AA amyloidosis.

**Methods:** Initially the Next Generation Sequencing (NGS) targeting 20 autoinflammatory genes was performed in the index patient and his sister, both of whom have been diagnosed with AA amyloidosis. There was a clear autosomal dominant inheritance affecting three generations. Upon finding a genetic cause the DNA obtained from other affected family members were analysed by Sanger sequencing.

**Results:** The index case was diagnosed with sJIA aged 7 and developed end stage renal failure in his early twenties. He had haemodialysis for four years and underwent renal transplantation at the age of 32. He was referred to the National Amyloidosis Centre (NAC) with a suspicion of AA amyloid deposits in the transplanted kidneys. He reported suffering with fever accompanied by severe abdominal and chest pain, arthritis, erythema and night sweats from early life. Interestingly having been started on colchicine for post-transplant gout he felt significantly better. Subsequently his sister developed nephrotic syndrome due to AA amyloidosis. She describes similar symptoms throughout most of her life. Their parents were of white British origin from a non-consanguineous kindred. Their father had died aged 52 years from complications immediately following a cadaveric renal transplantation. The post-mortem examination of his renal biopsy revealed deposition of AA amyloid fibrils. The index case grandmother also suffered with cyclical episodic abdominal cramping and had a history of osteoarthritis. The index case’s two paternal cousins and two of their children described similar symptoms.

NGS revealed a single *MEFV* allele to be affected in both the index case and his sister, resulting in the p.P373L variant in exon 3. Subsequently this variant was confirmed by Sanger sequencing in all living affected members. The mutant allele was not identified in the unaffected cases. All symptomatic individuals were treated with colchicine which suppressed their FMF related inflammation. We sequenced *SAA1* gene, as homozygosity for the *SAA1.1* allele is a known susceptibility factor for AA amyloidosis, but all cases were heterozygous.

**Conclusion:** In the Northern Caucasian population FMF is extremely rare in comparison to the Mediterranean region. Typically FMF is an autosomal recessive disorder, nonetheless very rare cases of dominantly inherited disease have previously been reported, namely caused by the deletion of methionine residue at position 694 identified in British patients and three substitutions affecting threonine 577: p.T577N, p.T577S and p.T577A found in British, Turkish and Dutch patients respectively.

Here we report a novel *MEFV* variant p.P373L causing dominant FMF in three generations of a large British family and in three cases this was complicated by AA amyloidosis indicating a severe pathogenicity of this variant.

**Consent for publication has been obtained from patient**

Yes

**Disclosure of Interest**

None Declared

### Oral communications – Immunology

#### O16 PYRIN inflammasome dysregulation in FMF patients: implication for a fast diagnostic test

##### Thomas Henry, Flora Magnotti, Alexandre Belot, Yvan Jamilloux

###### Inserm U1111, CNRS UMR5308, Univ. Lyon, ENS Lyon, CIRI, Lyon, France

####### **Correspondence:** Thomas Henry

**Introduction:** Familial Mediterranean Fever (FMF) is usually associated with bi-allelic mutations in the *MEFV* gene. *MEFV* encodes Pyrin, an inflammasome sensor leading to IL-1β release and a fast cell death termed pyroptosis. The relationship between *MEFV* mutations and Pyrin inflammasome regulations is still poorly understood. Furthermore, due to the large number of *MEFV* variants and a substantial proportions of FMF patients presenting mutations in a single *MEFV* allele, the genetic confirmation of the FMF diagnosis is often unconclusive.

**Objectives:** The objective of the study were:

1. to understand at the molecular levels the Pyrin inflammasome dysregulation in monocytes from FMF patients
2. to assess whether monitoring Pyrin inflammasome activation could lead to a novel functional diagnostic test for FMF

**Methods:** Monocytes from healthy donors (HD) or FMF patients were isolated and treated with a kinase inhibitor targeting the PKC superfamily, which includes PKN1/2 known to regulate Pyrin inflammasome activation. IL-1β release andcell death kinetics were monitored.

In parallel, a human monocyte cell line was engineered to modelize FMF and HD monocytes and assess the role of Pyrin phosphorylation and inflammasome components in cell death and cytokine release.

**Results:** Dephosphorylation of Pyrin was sufficient to trigger Pyrin inflammasome activation in monocytes from FMF patients while no inflammasome activation was observed in monocytes from healthy controls.

Using a human monocyte cell line, we demonstrated that dephosphorylation of Pyrin was similar in cells expressing wild-type or mutated MEFV but that a mutated MEFV was necessary and sufficient to progress to active inflammasome upon PKC superfamily inhibition.

Finally, thanks to a cohort of FMF patients, we demonstrate that FMF patients can be efficiently and specifically diagnosed based on the response of their monocytes to PKC superfamily inhibitors.

**Conclusion:** Our results demonstrate that Pyrin dephosphorylation is sufficient to trigger inflammasome activation in monocytes from FMF patients but not from healthy donors. This indicates that in healthy donors, the progression to an active Pyrin inflammasome requires two independently-controlled steps and that the second mechanism of control is deficient in monocytes from FMF patients.

Our study also demonstrates that monitoring inflammasome activation upon PKC superfamily inhibition in monocytes can discriminate FMF patients from patients with other inflammatory conditions opening the way to a fast functional test to diagnose FMF.

**Disclosure of Interest**

None Declared

#### O17 Unraveling the molecular pathogenesis of proteasome-associated autoinflammatory syndromes

##### Frédéric Ebstein^1^, Anja Brehm^2^, Sébastien Küry^3^, Thomas Besnard^3^, Bertrand Isidor^3^, Stéphane Bézieau^3^, Pawel Stankiewicz^4^, Elke Krüger^1^

###### ^1^Institut für Medizinische Biochemie und Molekularbiologie (IMBM), Universitätsmedizin Greifswald, Greifswald; ^2^Institut für Biochemie, Charité Universitätsmedizin Berlin, Berlin, Germany; ^3^Service de Génétique Médicale, CHU de Nantes, Nantes, France; ^4^Dept of Molecular and Human Genetics, Baylor College of Medicine, Houston, United States

####### **Correspondence:** Frédéric Ebstein

**Introduction:** In most eukaryotic cells, the degradation of abnormal and/or regulatory proteins is ensured by large multi-subunit ATP-dependent proteases known as proteasomes which consist in two distinct sub-complexes, a 20S core particle and a 19S regulatory particle. Since the early 2010s, an increasing number of loss-of-function mutations have been identified in genes encoding proteasome subunits including *PSMB8*, *PSMB9*, *PSMB4*, *PSMA3* and *PSMD12* and/or the proteasome maturation protein POMP. Fascinatingly, depending on the subunit affected, such genomic alterations result in the development of two seemingly distinct phenotypes, namely: (i) systemic autoinflammation or (ii) cognitive impairment. Herein, mutations of the 20S core particle subunits (i.e. *PSMB8*, *PSMB9*, *PSMB4* and *PSMA3*) and/or POMP are typically associated with a group of autoinflammatory syndromes sharing the same constellation of clinical signs and frequently referred to as chronic atypical neutrophilic dermatosis with lipodystrophy and elevated temperature (CANDLE) and/or proteasome-associated autoinflammatory syndromes (PRAAS). By contrast, genetic disruption of the subunits of the 19S regulatory particle (i.e. *PSMD12*) leads to a syndromic form of intellectual disability (ID).

**Objectives:** Our aim in this study is to determine whether syndromic ID disorders caused by variants in genes encoding 19S proteasome subunits share similarities with CANDLE/PRAAS in their etiology and/or pathogenesis particularly regarding inflammation.

**Methods:** Peripheral blood mononuclear cells (PBMC) from subjects carrying 19S proteasome loss-of-function mutations diagnosed with syndromic ID as well as related control were collected and subjected to RNA isolation and protein extraction prior to quantitative PCR and western-blot analysis, respectively.

**Results:** Like CANDLE/PRAAS, syndromic ID disorders caused by genomic alterations in 19S proteasome genes were associated with perturbed proteasome function, as evidenced by increased levels of intracellular ubiquitin-protein conjugates. Most importantly, our data show that PBMC from patients with syndromic ID exhibit high transcription levels of various IFN-induced genes (ISG) including CXCL10, CXCL9, IFI44 and IFI44L. Interestingly, the strength of the IFN signatures in these patients correlated with the magnitude of the unfolded protein responses (UPR) initiated by proteasome dysfunction, suggesting a cause-and-effect relationship between endoplasmic reticulum (ER) stress and inflammation.

**Conclusion:** In this work, we provide evidence on the association of 19S proteasome dysfunction with the generation of autoinflammation in subjects diagnosed with syndromic ID disorders. From these data, we expect to convey a more integrated picture of the pathophysiology of syndromic ID and identify new therapeutic targets for the treatment of cognitive impairment.

**Disclosure of Interest**

None Declared

#### O18 A novel knock-in mouse model of CAPS that develops amyloidosis: therapeutic efficacy of proton pump inhibitors

##### Arinna Bertoni^1^, Sonia Carta^2^, Chiara Baldovini^3^, Federica Penco^1^, Enrica Balza^2^, Silvia Borghini^4^, Marco Di Duca^4^, Emanuela Ognio^5^, Paolo Nozza^3^, Francesca Schena^1^, Patrizia Castellani^2^, Claudia Pastorino^1^, Carola Perrone^1^, Laura Obici^6^, Alberto Martini^7^, Isabella Ceccherini^4^, Marco Gattorno^1^, Anna Rubartelli^2^, Sabrina Chiesa^1^

###### ^1^Centro Malattie Autoinfiammatorie ed Immunodeficienze, IRCCS Istituto G. Gaslini; ^2^Unità di Biologia Cellulare, IRCCS Ospedale Policlinico San Martino; ^3^Anatomia Patologica; ^4^Genetica Medica, IRCCS Istituto G. Gaslini; ^5^S.S Animal Facility, IRCCS Ospedale Policlinico San Martino, Genova; ^6^Centro per lo Studio e la Cura delle Amiloidosi Sistemiche, Fondazione IRCCS Policlinico San Matteo, Pavia; ^7^Direzione Scientifica, IRCCS Istituto G. Gaslini, Genova, Italy

####### **Correspondence:** Arinna Bertoni

**Introduction:** Cryopyrin associated periodic syndromes (CAPS) are a group of autoinflammatory diseases linked to gain-of-function mutations in the NLRP3 gene that cause uncontrolled IL-1β secretion. CINCA syndrome is the most severe CAPS disease characterized by central nervous system disabilities with a long-term risk of secondary amyloidosis.

Proton pump inhibitors (PPIs), commonly used as inhibitors of gastric acidproduction, also display anti-inflammatory properties, making them promising drugs in sepsis and in inflammatory disorders.

**Objectives:** To develop a novel NLRP3 knock-in (KI) mouse model of CAPSto evaluate amyloid deposition and to test alternative therapeutic approaches.

**Methods:** We generated KI mice by engineering N475K mutation associated with CAPS phenotype into mouse *Nlrp3* gene. KI and Wild Type (WT) mice received PPIs or PBS intraperitoneally and were analyzed for survival, inflammation, cytokine secretion, and amyloidosis development. Cytokines secretion from bone marrow derived dendritic cells (BMDCs) and peritoneal macrophages (PMs) was evaluated by ELISA. Hystological analysis of all organs was evaluated by hematoxylin and eosin staining. Amyloid deposition was quantified through Congo Red staining.

**Results:** Mutant NLRP3 KI mice displayed features that recapitulates the immunological and clinical phenotype of CAPS. These mice had systemic inflammation, with high levels of serum pro-inflammatory cytokines compared to WT controls. Hystological analysis revealed the presence of acute and chronic inflammatory cell infiltrates and amyloid deposits in spleen, liver and kidneys. As in CAPS monocytes , BMDCs and PM from KI mice showed a strong increase in IL-1β, IL-18, and IL-1α secretion and decreased levels in interleukin-1 receptor antagonist (IL-1Ra), the naturally occurring IL-1b inhibitor.

PPIs treatment of KI mice showed a clear clinical impact with improvement of inflammatory conditions and regression of amyloid deposits. Remarkably, BMDCs and PMs from PPI-treated mice presentedreduced secretion of pro-inflammatory cytokines and re-established the levels of IL-1RA.

**Conclusion:** NLRP3 KI mice display a CAPS phenotype with many characteristics of autoinflammation, including amyloidosis. The therapeutic effectiveness associated with lack oftoxicity indicates that PPIs could represent relevant adjuvants to the anti-IL-1 drugs in IL-1 driven diseases.

**Disclosure of Interest**

None Declared

#### O19 T cell defects in patients with ARPC1B germline mutations account for combined immunodeficiency

##### Immacolata Brigida^1^, Matteo Zoccolillo^1,4^, Maria Pia Cicalese^1,2,3^, Laurène Pfajfer^5-9^, Federica Barzaghi^2,4^, Serena Scala^1^, Carmen Oleaga-Quintas^10,11^, Jesus A. Alvarez^12^, Lucia Sereni^1^, Stefania Giannelli^1^, Claudia Sartirana^1^, Francesca Dionisio^1^, Luca Pavesi^13^, Marta Benavides-Nieto^14,15^, Luca Basso-Ricci^1^, Paola Capasso^1^, Benedetta Mazzi^16^, Jeremie Rosain^10,11,28^, Nufar Marcus^17^, Yu Nee Lee^18^, Raz Somech^18^, Massimo Degano^19^, Giuseppe Raiola^20^, Roberta Caorsi^21^, Paolo Picco^21^, Marcela Moncada Velez^12^, Joelle Khourieh^11,12^, Andrés Augusto Arias^12,29^, Aziz Bousfiha^22^, Thomas Issekutz^23^, Andrew Issekutz^23^, Bertrand Boisson^11,12,24^, Kerry Dobbs^25^, Anna Villa^1,26^, Angelo Lombardo^1,3^, Benedicte Neven^14^, Despina Moshous^14,15^, Jean-Laurent Casanova^11,12,14,24,27^, José Luis Franco^12^, Luigi D Notarangelo^25^, Cristina Scielzo^13^, Stefano Volpi^21,30^, Loïc Dupré^5-9^, Jacinta Bustamante^11,12,24,28^, Marco Gattorno^21,31‡^, and Alessandro Aiuti^1,2,3‡^

###### ^1^San Raffaele Telethon Institute for Gene Therapy, SR-TIGET; ^2^Pediatric Immunohematology, San Raffaele Scientific Institute, Milan; ^3^Vita-Salute San Raffaele University, Milan, Italy; ^4^Department of Systems Medicine, Tor Vergata University, Rome, Italy; ^5^INSERM, UMR1043, Centre de Physiopathologie de Toulouse Purpan, Toulouse, France; ^6^CNRS, UMR5282, Toulouse, France; ^7^Université Toulouse III Paul-Sabatier, Toulouse, France; ^8^Ludwig Boltzmann Institute for Rare and Undiagnosed Diseases, Vienna, Austria; ^9^CeMM Research Center for Molecular Medicine of the Austrian Academy of Sciences, Vienna, Austria; ^10^Laboratory of Human Genetics of Infectious Diseases, Necker Branch, INSERM U1163, 75015 Paris, France, EU; ^11^Paris Descartes University, Imagine Institute, 75015 Paris, France, EU; ^12^Group of Primary Immunodeficiencies, Department of Microbiology & Parasitology, School of Medicine, University of Antioquia UdeA, Medellin, Colombia; ^13^Division of Experimental Oncology, Unit of B-cell Neoplasia, San Raffaele Scientific Institute, Milan; ^14^Pediatric Hematology-Immunology Unit, Necker Hospital for Sick Children, AP-HP, 75015 Paris, France, EU; ^15^Genome Dynamics in the Immune System, Université Paris Descartes – Sorbonne Paris; ^16^Immunogenetics Laboratory, HLA & Chimerism, Dept. of Immunohematology & Blood Transfusion, IRCCS Ospedale San Raffaele, Milano, Italy; ^17^Kipper Institute for Allergy and Immunology, Schneider Children’s Medical Center of Israel, Petach Tikva, Israel, affiliated with Sackler School of Medicine, Tel Aviv University, Tel Aviv, Israel; ^18^Pediatric Department A and the Immunology Services, “Edmond and Lily Safra” Children's Hospital, Jeffrey Modell Foundation Center, Sheba Medical Center, Tel Hashomer affiliated with Sackler School of Medicine, Tel Aviv University, Tel Aviv, Israel; ^19^Division of Immunology, Transplantation, and Infectious Diseases, Biocrystallography Unit. San Raffaele Scientific Institute, Milan; ^20^Unità Operativa di Pediatria, Azienda Ospedaliera “Pugliese-Ciaccio” di Catanzaro; ^21^U.O. Clinica Pediatrica e Reumatologia, Istituto Giannina Gaslini, Genova, Italy; ^22^Clinical Immunology Unit, Department of Pediatrics, King Hassan II University, Ibn-Rochd Hospital, Casablanca, Morocco; ^23^Department of Pediatrics & Department of Microbiology-Immunology, Dalhousie University, Halifax, Nova Scotia, Canada; ^24^St. Giles Laboratory of Human Genetics of Infectious Diseases, Rockefeller Branch, The Rockefeller University, New York, NY 10065, USA; ^25^Laboratory of Clinical Immunology and Microbiology, National Institute of Allergy and Infectious Diseases, NIH, Bethesda, USA; ^26^Istituto di Ricerca Genetica e Biomedica (IRGB), Consiglio Nazionale delle Ricerche (CNR), Milan Unit, Milan, Italy; ^27^Howard Hughes Medical Institute, NY, USA; ^28^Center for the Study of Primary Immunodeficiencies, Assistance Publique-Hôpitaux de Paris AP-HP, Necker Hospital for Sick Children, Paris, France, EU; ^29^School of Microbiology, University of Antioquia UdeA, Medellin, Colombia; ^30^Università degli Studi di Genova, Genova, Italy; ^31^UOSD Centro Malattie e Autoinfiammatorie e Immunodeficienze, Istituto Giannina Gaslini, Genova, Italy

####### **Correspondence:** Alessandro Aiuti

**Introduction:** ARPC1B is a key factor for the assembly and maintenance of the ARP2/3 complex, involved in actin branching from an existing filament. Germline mutations in *ARPC1B* have been recently described in six unrelated patients, with clinical features of combined immunodeficiency, whose neutrophils and platelets but not T lymphocytes were studied.

**Objectives:** We hypothesized that ARPC1B-deficiency may also lead to cytoskeleton and functional defects in T cells

**Methods:** Next-generation sequencing in 6 patients; flow cytometry, proliferation and migration, confocal and electron microscopy) to characterize defects in T cells; lentiviral-mediated gene transfer for genetic correction of ARPC1B.

**Results:** We identified bi-allelic mutations in 6 unrelated patients with severe infections, autoimmune manifestations and platelet defects showing altered protein structure, and absent/low expression of the ARPC1B protein. Confocal microscopy showed altered expression of ARPC1B with actin. T cells displayed impaired TCR-mediated proliferation and SDF1-**α** directed migration. Gene transfer of ARPC1B in patient’s T cells using a lentiviral vector restored ARPC1B expression, leading to improved T-cell proliferation *in vitro*. In 2 patients normal ARPC1B levels in a fraction of lymphocytes were associated with *in vivo* somatic reversion and improved T cell migration *in vitro.* In one of the patient somatic revertant was present only in memory CD8+ T-cells, which showed improved in vitro T-cell migration.

**Conclusion:** Inherited ARPC1B deficiency alters T cell cytoskeletal dynamics and functions, contributing to the clinical features of combined immunodeficiency.

**Disclosure of Interest**

None Declared

#### O20 B cell defect in ADA2 deficiency patients

##### Francesca Schena^1^, Federica Penco^1^, Stefano Volpi^1,2^, Claudia Pastorino^1^, Roberta Caorsi^1^, Arinna Bertoni^1^, Francesca Kalli^3^, Daniela Fenoglio^3,4,5^, Annalisa Salis^6^, Ignazia Prigione^1^, Paola Bocca^1^, Francesca Antonini^7^, Antonella Insalaco^8^, Alice Grossi^9^, Gianluca Damonte^6^, Isabella Ceccherini^9^, Gilberto Filaci^3,4,5^, Alberto Martini^1,2^, Elisabetta Traggiai^10^, Marco Gattorno^1^

###### ^1^UOSD Centro Malattie Autoinfiammatorie e Immunodeficienze and Clinica Pediatrica e Reumatologia, IRCCS Istituto Giannina Gaslini; ^2^Università degli studi di Genova; ^3^Center of Excellence for Biomedical Research; ^4^Department of Internal Medicine, Clinical Immunology Unit, Università di Genova; ^5^Ospedale Policlinico San Martino; ^6^Department of Experimental Medicine and Center of Excellence for Biomedical Research, Università di Genova; ^7^Core Facilities Flow-Cytometry and Cell imaging Lab, Istituto Giannina Gaslini, Genova; ^8^Division of Rheumatology, Department of Pediatric Medicine, IRCCS, Bambino Gesù Children’s Hospital, Roma; ^9^Medical Genetics, IRCCS Istituto Giannina Gaslini, Genova, Italy; ^10^Novartis Institutes for Biomedical Research, Basel, Switzerland

####### **Correspondence:** Francesca Schena

**Introduction:** Adenosine Deaminase 2 Deficiency (DADA2) is an autoinflammatory disease characterized by systemic vasculopathy, strokes and mild immunodeficiency, mainly affecting B cell compartment. The defect is due to a loss of function mutation of *ADA2* gene, coding for Adenosine Deaminase 2, a protein which regulates the catabolism of extracellular adenosine.

**Objectives:** Hypogammaglobulinemia and recurrent infections are associated to DADA2. We therefore investigated phenotype and *in vitro* B and T cell responses in DADA2 patients to address if *ADA2* mutation affects B and T cell function and in particular we focused on B cell-T cell interaction.

**Methods:** 14 patients carrying loss of function mutations in *ADA2* were examined. They showed clinical history with livedo reticularis, fever, vasculitis and neurological symptoms. We analyzed peripheral B and T cell phenotype by flow cytometry, *in vitro* B-cell proliferation and differentiation to Immunoglobulin secreting cells in response to TLR9 agonist and T cell help. Moreover B cells isolated from DADA2 patients or HD have been cultured in co-culture with CD4+ T cells and *in vitro* B cell proliferation has been evaluated by CFSE dilution, whereas B cell differentiation and immunoglobulin secretion in response to TLR9 agonist and T cell help have been evaluated by ELISA and ELISPOT assay. Simultaneously cytokine production from Tfh cells has been analyzed.

**Results:** Flow-cytometric analysis of DADA2 peripheral blood showed a significant reduction of switch memory B cells and an increased frequency of CD21low B cells. Regarding T cell compartment memory CD4+ and CD8+ T cells are significantly reduced; interestingly we identified an expansion in frequency of circulating Tfh cells.

Then we investigated a role of ADA2 in B cells: we found that ADA2 is expressed in all B cell subsets, secreted but its enzymatic activity is strongly impaired in DADA2. We show that Naïve B cells from DADA2 patients are impaired in their proliferation, suggesting an intrinsic defect due to the mutation.

We also addressed the interaction between B and helper T cells; DADA2 CD4+ T cells showed an impairment in the IL21 production and a downregulation of CD40L, suggesting a functional defect of Tfh cells; then we found that proliferation and differentiation of patients’ B cells were not sustained from patients’ CD4+ T cells.

**Conclusion:** Our findings suggest that ADA2 mutations could lead to an intrinsic defect in B cell function and to a reduced T cell dependent B cell response.

**Disclosure of Interest**

None Declared

#### O21 Periodic Fever, Aphthous Stomatitis, Pharyngitis and Adenitis (PFAPA) syndrome and obstructive sleep apnea are distinct inflammatory disorders of oropharyngeal lymphoid tissue

##### Kalpana Manthiram^1^, Silvia Preite^2^, Fatma Dedeoglu^3^, Maranda Lawton^3^, Pamela Mudd^4^, Hemalatha Srinivasalu^4^, Greg Licameli^3^, Kathryn Edwards^5^, Pamela Schwartzberg^2^, Daniel Kastner^1^

###### ^1^National Human Genome Research Institute; ^2^National Institute of Allergy and Infectious Diseases, NIH, Bethesda; ^3^Boston Children’s Hospital, Harvard Medical School, Boston; ^4^Children’s National Medical Center, George Washington University School of Medicine, Washington; ^5^Vanderbilt University School of Medicine, Nashville, United States

####### **Correspondence:** Kalpana Manthiram

**Introduction:** Tonsillectomy is one of the most common surgical procedures in children and is performed typically for recurrent tonsillitis or obstructive sleep apnea (OSA), and less frequently for periodic fever, aphthous stomatitis pharyngitis, cervical adenitis (PFAPA) syndrome. The pathogenesis of OSA and PFAPA is unknown.

**Objectives:** In order to understand the pathogenesis of these two disorders, we studied the immunologic profile of tonsils removed from children with PFAPA and OSA in comparision with control tonsils.

**Methods:** After getting informed consent, we obtained tonsils from children undergoing tonsillectomy for (1) PFAPA (N=12) and (2) OSA (N=12), and from (3) children undergoing tonsillectomy during oropharyngeal anatomic correction surgery like pharyngeal flap preparation (“controls”, N=9).Mononuclear cells from the tonsils were separated by standard methods and cells were analyzed by flow cytometry and gene expression with Nanostring. Cytokine production by isolated tonsillar mononuclear cells was measured by flow cytometry following phorbol 12-myristate 13-acetate (PMA) and ionomycin stimulation for 4 hours *ex vivo*.

**Results:** Tonsils from patients with PFAPA, which were removed during asymptomatic intervals, had evidence of germinal center suppression with significantly fewer T follicular helper cells, more T follicular regulatory cells, fewer plasma cells and reduced IgG class switching compared to tonsils from either OSA patients or controls. Gene expression analysis revealed downregulation of pro-inflammatory genes in tonsillar myeloid cells from patients with PFAPA. However, upon stimulation with PMA and ionomycin, CD4+ T cells from tonsils of patients with PFAPA produced more IFNγ and IL-17 than that of controls, but less than those of patients with OSA.

On the other hand, tonsils from patients with OSA, had significantly larger germinal center areas by histology compared to those from patients with PFAPA and controls. Moreover, in tonsils from OSA patients, we found higher IFNγ production by CD4+ T, CD8+ T and NK cells, and greater IL-17 production by CD4+ T cells and NK cells upon stimulation with PMA and ionomycin when compared with tonsils from PFAPA patients and controls.

**Conclusion:** We show that tonsils from both patients with PFAPA and OSA exhibit evidence of immune dysregulation. During asymptomatic periods, PFAPA patients display myeloid cell and germinal center suppression in the tonsils, suggesting a compensatory response to inflammatory flares. However, upon stimulation, CD4+ T cells produce high levels of pro-inflammatory cytokines. We hypothesize that alternating periods of heightened immune activation and suppression lead to the periodicity of PFAPA. In comparison, tonsils from patients with OSA display relatively constant germinal center hypertrophy likely due to unchecked chronic T cell activation. Further studies are necessary to understand the genetic and environmental risk factors for T cell activation in both of these disorders and why PFAPA patients may have cycling of inflammatory periods in the tonsils while OSA patients have chronic tonsillar inflammation.

**Disclosure of Interest**

None Declared

#### O22 PFAPA syndrome: NK cells infiltrating tonsils support the crucial role of innate immunity in the pathogenesis

##### Sabrina Chiesa^1^, Roberta Caorsi^2^, Francesca Bellora^3^, Mariella Della Chiesa^3^, Ilaria Ingrosso^1^, Federica Penco^1^, ArinnaBertoni^1^, Claudia Pastorino^1^, Ignazia Prigione^1^, Alessia Omenetti^2^, Martina Finetti^2^, Silvia Borghini^4^, Angela Sementa^5^, Roberto D’Agostino^6^, LuciaSemino^6^, Alberto Martini^7^, Cristina Bottino^3^, Marco Gattorno^1^

###### ^1^Centro Malattie Autoinfiammatorie e Immunodeficienze; ^2^Clinica Pediatrica e Reumatologica, IIRCCS G. Gaslini; ^3^Medicina Sperimentale, University of Genoa; ^4^Laboratorio di Genetica Molecolare; ^5^Anatomia Patologica; ^6^UO Otorinolaringoiatria, IIRCCS G. Gaslini; ^7^Clinica Pediatrica e Reumatologica, University of Genoa, Genova, Italy

####### **Correspondence:** Sabrina Chiesa

**Introduction:** Periodic fever, aphthous stomatitis, pharyngitis, and cervical adenitis (PFAPA) syndrome is a more frequent cause of recurrent fever in children. The exact etiology of this pediatric disorder is still unknown. Elevated serum levels of IL-1β, IL-18, IL-6, and IFNγ suggest innate immunity dysregulation as the key pathomechanism of PFAPA attacks. Palatine tonsils are sites where innate immunity leads to the onset of adaptive immunity, mediated by B and T lymphocytes. Natural killer (NK) cells, the most important effectors of the innate lymphoid cells (ILCs), play a fundamental role in innate immune responses.

**Objectives:** Tonsillectomy is one of the therapeutic options for PFAPA patients. We tested whether specific infiltrating inflammatory cells in pediatric tonsils contribute to PFAPA pathogenesis.

**Methods:** Tonsils were collected from 2 groups of pediatric patients undergoing tonsillectomy: PFAPA patients (n=32) and children with bacterial tonsillitis (control group, CG) (n=25). Phenotypic analysis of subpopulations of tonsil cell suspensions and tissues was performedby flow cytometry and immunohistochemistry.

**Results:** During the asymptomatic phase of disease the number of monocytes did not differ between the PFAPA and control tonsils. We observed a considerable recruitment of NK cells in tonsils of PFAPA patients respect to CG. In particular, we detected a significant expansion of both CD56^bright^ CD16^-^ and CD56^dim^ CD16^+^ NK cell subsets in PFAPA samples compared to CG. A fine characterization of activating and inhibitory NK receptors suggested a crucial role of CD56^dim^ CD16^+^ cell subset in PFAPA disease. Specially, activating receptors, as natural cytotoxicity receptors (NCRs) and NKG2D, were higher in NK cells of PFAPA patients than in NK cells from CG. Remarkably, CD56^+^ NK cells from PFAPA tonsils werecytotoxic and contained much more perforine and granzyme than those from CG tonsils. NK cells from PFAPA tonsils exhibited increased IFN-g production, compared to NK from CG. Accordingly, plasmacytoid dendritic cells, the main source of type I interferon cytokines, were significantly increased in PFAPA tonsils. We detected a higher number of naïve and a significantly lower percentage of effector memory CD4^+^ and CD8^+^ T cells in PFAPA tonsils compared to CG. Additionally, PFAPA tonsils presented a significant decrease of both functional follicular helper T cells (CXCR5^+^ICOS^+^) and T regulatory cells (CD39^+^Foxp3^+^). Finally, CD19^+^CD38^+^ B cellswere reduced in this cohort of PFAPA tonsils.

**Conclusion:** These results suggest aninvolvement of NK cells in the pathogenesis of PFAPA and support the crucial role ofinnate immunity in the disease. Nonetheless, the abundant and activated CD56^+^NK cells might shape adaptive immunity that is impaired in the tonsils of PFAPA patients.

**Disclosure of Interest**

None Declared

### Novel AID pathways and genes

#### O23 Identification of rare coding variants in IL-1-related pathways in patients with adult-onset still’s disease

##### Giulio Cavalli^1,2^, Rosanne Van Deuren^2^, Peer Arts^2^, Marloes Steerhower^2^, Paolo Sfriso^3^, Paola Galozzi^3^, Serena Colafrancesco^4^, Roberta Priori^4^, Luca Cantarini^5^, Stefano Rodolfi^1^, Elena Baldissera^1^, Frank van der Veerdonk^2^, Lorenzo Dagna^1^, Alexander Hoischen^2^, Charles A. Dinarello^2,6^

###### ^1^Unit of Immunology, Rheumatology, Allergy and Rare Diseases (UniRAR), Vita-Salute San Raffaele University, Milan, Italy; ^2^Department of Medicine, Radboud University Medical Centre, Nijmegen, Netherlands; ^3^Rheumatology, University of Padua, Padua; ^4^Rheumatology, Sapienza University, Rome; ^5^Rheumatology, University of Siena, Siena; ^6^Department of Medicine, University of Colorado Denver, Aurora, CO, Italy

####### **Correspondence:** Giulio Cavalli

**Introduction:** Adult-onset Still's disease (AOSD) is a rare autoinflammatory disease characterized by fever, arthritis, and multi-organ involvement. Inflammation in AOSD is mediated by interleukin (IL)-1β, as confirmed by the dramatic clinical efficacy of selective blockers of this cytokine. The genetic predisposition to this rampant IL-1-driven inflammation remains nevertheless elusive. Previous studies failed to identify associations between polymorphisms in the genes encoding IL-1 and AOSD, thus pointing at more complex genetic mechanisms. This ‘missing heritability’ cannot be adequately investigated with traditional techniques for genetic partitioning, such as GWAS, which only assess common variants and polymorphisms. Studies focusing on highly penetrant rare variants or different types of mutations (i.e. small copy-number variations; insertions/deletions) are warranted.

**Objectives:** We hypothesized that genetically determined changes in IL-1-related pathways resulting in excessive IL-1β activity lead to the development of autoinflammation in AOSD. Scope of this study was to unravel the combined mutational variation of a network of IL-1-related receptors, pathways, counter-regulators, and cellular processes possibly involved in the pathogenesis of AOSD and IL-1-mediated inflammation in general.

**Methods:** We collected clinical, demographic, and genetic data from a large cohort of 76 AOSD patients and developed an innovative platform based on molecular inversion probes (MIP) technology for performing highly multiplexed targeted-resequencing. This allows efficient sequencing of the coding sequence of 48 genes related to the IL-1-pathway, and allows studying rare and common variants in one assay. We have also screened 500 healthy controls, and 1000s of samples with other disorders using the same assay.

**Results:** We identified rare and unique (i.e. private variants) in the IL1 pathway in several individuals with AOSD. Whether any these are involved in a strong predisposition to AOSD is currently followed-up. Rare genetic variants have been identified in six IL-1-pathway ‘clusters’:

1. Deregulated activation of the inflammasome and release of IL‑1β and IL-18.
2. IL-1 family receptors and intracellular signaling mediators.
3. Other pro-inflammatory cytokines and receptors.
4. Regulatory molecules, including IL-1Ra or IL-37.
5. Cellular processes regulating production of IL-1 and IL-18 (i.e. autophagy).
6. Production of ROS, which function as markers of cellular damage and trigger inflammation.

**Conclusion:** Unraveling the genetic bases of inflammation in AOSD deepens our understanding of the human innate immunome. Of note, this study platform may serve for the genetic analysis of other IL-1-mediated conditions, including gout and other autoinflammatory diseases, whose genetic predisposition remains elusive. Equally important, the identification of pathways amenable to targeting with small molecules or biologics may translate into remarkable clinical implications.

**Disclosure of Interest**

None Declared

#### O24 Trisomy 8-associated autoinflammatory disease (TRIAD) is characterized by increased monocyte activation

##### Kalpana Manthiram^1^, Alina Dulau-Florea^2^, Deborah Bruns^3^, Amanda Ombrello^1^, Karyl Barron^4^, Tina Romeo^1^, Anne Jones^1^, Karin Weiss^1^, Sandro Perazzio^5^, Isabelle Kone-Paut^6^, Nora Al-Mutiari^7^, Troy Torgerson^5^, Daniel Kastner^1^

###### ^1^National Human Genome Research Institute; ^2^Department of Laboratory Medicine, NIH, Bethesda; ^3^Department of Counseling, Quantitative Methods, and Special Education, Southern Illinois University Carbondale, Carbondale; ^4^National Institute of Allergy and Infectious Diseases, NIH, Bethesda; ^5^Department of Pediatrics, University of Washington School of Medicine, Seattle, United States; ^6^Bicêtre University Hospital, APHP, CEREMAIA, University of Paris Sud, Paris, France; ^7^Department of Pediatrics, Sabah Hospital, Kuwait City, Kuwait

####### **Correspondence:** Kalpana Manthiram

**Introduction:** Many adults with acquired trisomy 8 and myelodysplasia have been reported to have Behçet’s-like inflammatory disease. A few patients with constitutional trisomy 8 mosaicism and ulcerative disease have also been reported. The spectrum of phenotypic abnormalities, inflammatory profiles, and treatment responses of patients with constitutional trisomy 8 are not well-characterized.

**Objectives:** We analyzed the clinical and immunologic features of a cohort of patients with trisomy 8 mosaicism in order to understand the pathogenesis of the disorder.

**Methods:** Whole blood gene expression was analyzed with the Nanostring Human Immunology panel. Copy number of two genes on the p and q arms of chromosome 8 were determined by digital droplet PCR in sorted CD3+ T cells, CD19+ B cells, and CD14+ monocytes from peripheral blood to determine the percentage of mosaicism in different cell populations. Platelet electron microscopy was performed in a clinical laboratory.

**Results:** Eleven patients with trisomy 8 mosaicism ranging in age from 5 to 37 years were recruited. Eight-two percent had recurrent fever, 82% had oral ulcerations, and 72% had severe oral ulcerations larger than 1 cm or lasting more than one week. Five patients reported genital, esophageal, or colonic ulcers. Nine patients had cognitive or motor delay. Two patients had symptoms of a bleeding diathesis with subdural hematoma, petechiae, and/or menorrhagia with normal platelet counts; the platelets of these patients had fewer dense granules by electron microscopy. Patients had elevated peripheral absolute monocyte count (average 970 cells/uL), but none had chronic myelomonocytic leukemia, acute myeloid leukemia or myelodysplasia. Whole blood gene expression revealed upregulation of IL-1-, TLR- and NF-ĸB-related genes. CD14+ monocytes had a significantly higher percentage of cells with trisomy 8 compared to CD3+ T cells and CD19+ B cells (chromosome 8 copy number was 2.8 in CD14+ cells vs. 2.5 in CD19+ [p=0.01] and 2.3 in CD3+ cells [p =0.001]). Four out of 5 patients had partial improvement on daily colchicine, 3 out of 6 reported improvement with intermittent or daily anakinra, 2 out of 3 had improvement on TNFα inhibitors (etanercept or infliximab), and one patient improved following hematopoietic stem cell transplant.

**Conclusion:** We report the spectrum of clinical and immunologic manifestations in patients with trisomy 8 mosaicism, a disease we name trisomy 8-associated autoinflammatory disease (TRIAD). Patients with TRIAD present with (1) recurrent fever and/or severe mucosal ulcerations, (2) bleeding diatheses, and (3) developmental delay. Patient monocytes are elevated in number and have higher percentage of trisomy 8, indicating that an extra copy of chromosome 8 may confer a survival advantage preferentially to monocytes. In addition, activation pathways in monocytes are upregulated, and patients have symptom improvement in response to blockade of TNFα and IL-1, prominent cytokines associated with myeloid cell activation. Like monocytes, megakaryocytes and platelets develop from a common myeloid progenitor suggesting that cell development in the myeloid lineage may be affected. Further studies are underway to better characterize the inflammatory and survival pathways in myeloid cells with trisomy 8.

**Disclosure of Interest**

None Declared

### Deficiencies in key regulatory signals

#### O25 A combined immunodeficiency with severe infections, inflammation and allergy caused by ARPC1B deficiency

##### Stefano Volpi^1,2^, Maria Pia Cicalese^3^, Paul Tuijnenburg^4,5^, Anton T. J. Tool^6^, Eloy Cuadrado^7^, Hamid Ahanchian^8^, Raed Alzyoud^9^, Zeynep C. Akdemir^10^, Federica Barzaghi^11^, Alexander Blank^12^, Bertrand Boisson^13^, Cristina Bottino^14^, Roberta Caorsi^15^, PaoloPicco^15^, Jean-Laurent Casanova^13,16^, Sabrina Chiesa^17^, Ivan Kingyue Chinn^18^, Gregor Dückers^19^, Anselm Enders^20^, Hans Christian Erichsen^21^, LisaR. Forbes^18^, Tomasz Gambin^22,23^, Marco Gattorno^24^, Ehsan G. Karimiani^25^, Silvia Giliani^26^, Michael S. Gold^27^, Marwan Abu-Halaweh^28^, Eva-Maria Jacobsen^12^, Machiel H. Jansen^29,30^, Jovanka R. King^27^, Ronald M. Laxer^31^, James R. Lupski^22,32^, Emily Mace^18^, Stefania Marcenaro^33^, Reza Maroofian^34^, Alexander B. Meijer^35^, Tim Niehues^19^, Luigi D. Notarangelo^36^, Jordan Orange^18^, Ulrich Pannicke^37^, Chris Pearson^38^, Patrick J. Quinn^27^, Ansgar Schulz^12^, Filiz Seeborg^18^, Asbjørg Stray-Pedersen^39^, Hasan Tawamie^40^, Ester M. M. van Leeuwen^30^, Alessandro Aiuti^11^, Rae Yeung^31,41^, Klaus Schwarz^37,42^, Taco W. Kuijpers^29,43^

###### ^1^Clinica Pediatrica e Reumatologia, Centro per le malattie Autoinfiammatorie e Immunodeficienze, Istituto Giannina Gaslini; ^2^DINOGMI, Università degli Studi di Genova, Genova; ^3^Pediatric Immunohematology, San Raffaele Hospital and San Raffaele Telethon Institute for Gene Therapy (SR-TIGET), Milan, Italy; ^4^Department of Pediatric Immunology, Rheumatology and Infectious diseases, Emma Children’s Hospital, Amsterdam UMC, University of Amsterdam; ^5^Department of Experimental Immunology, Amsterdam Infection & Immunity Institute; ^6^Department of Blood Cell Research; ^7^Department of Immunopathology, Sanquin Research and Landsteiner Laboratory AMC, University of Amsterdam, Amsterdam, Netherlands; ^8^Department of Allergy and immunology, School of medicine, Mashhad university of Medical Sciences, Mashhad, Iran, Islamic Republic Of; ^9^Immunology, Allergy and Rheumatology section- Bone Marrow Transplantation for Primary Immunodeficiency Disorders, Queen Rania Children's Hospital, Amman, Jordan; ^10^Baylor-Hopkins Center for Mendelian Genomics of the Department of Molecular and Human Genetics, Baylor College of Medicine, Huston, United States; ^11^Pediatric Immunohematology, San Raffaele Hospital and San Raffaele Telethon Institute for Gene Therapy (SR-TIGET), Milan, Italy; ^12^Department of Pediatrics, University Medical Center Ulm, Ulm, Germany; ^13^St Giles Laboratory of Human Genetics of Infectious Diseases, Rockefeller Branch, The Rockefeller University, New York, United States; ^14^Department of Experimental Medicine (DIMES), University of Genoa; ^15^Centro per le Malattie Infiammatorie e Immunodeficienze, Clinica Pediatrica e Reumatologia, Istituto Giannina Gaslini, Genova, Italy; ^16^Pediatric Hematology-Immunology and Rheumatology Unit, Necker Hospital for Sick Children, APHP, Paris, France; ^17^Clinica Pediatrica e Reumatologia, Centro per le malattie Autoinfiammatorie e Immunodeficienze Istituto Giannina Gaslini, Genova, Italy; ^18^Department of Pediatrics, Section of Allergy, Immunology, and Rheumatology & Center for Human Immunobiology, Texas Children's Hospital, Houston, United States; ^19^Center for Child and Adolescent Medicine, Helios-Clinic, Krefeld, Germany; ^20^Department of Immunology and Infectious Disease, John Curtin School of Medical Research and Centre for Personalised Immunology, Australian National University, Canberra, Australia; ^21^Section of Paediatric Medicine and Transplantation, Division of Paediatric and Adolescent Medicine, Oslo University Hospital, oslo, Norway; ^22^Baylor-Hopkins Center for Mendelian Genomics of the Department of Molecular and Human Genetics, Baylor College of Medicine, Houston, United States; ^23^Institute of computer science, Warsaw University of Technology, Warsaw, Poland; ^24^. Clinica Pediatrica e Reumatologia, Centro per le malattie Autoinfiammatorie e Immunodeficienze Istituto Giannina Gaslini, Genova, Italy; ^25^Molecular and Clinical Sciences Institute, St. George’s, University of London, Cranmer Terrace, London, United Kingdom; ^26^Medical Genetics Unit and “A. Nocivelli” Institute for Molecular Medicine, Spedali Civili Hospital, Department of Molecular and Translational Medicine, University of Brescia, Brescia, Italy; ^27^Discipline of Pediatrics, School of Medicine, University of Adelaide and Department of Allergy and Clinical Immunology, Women's and Children's Health Network, Adelaide, Australia; ^28^Department of Biotechnology and Genetics Engineering in Philadelphia University, Jordan, United States; ^29^Emma Children’s Hospital, Amsterdam UMC, University of Amsterdam, Department of Pediatric Immunology, Rheumatology and Infectious diseases; ^30^Amsterdam UMC, University of Amsterdam, Department of Experimental Immunology, Amsterdam Infection & Immunity Institute, Amsterdam, Netherlands; ^31^Division of Rheumatology, Department of Paediatrics and Department of Medicine, University of Toronto, The Hospital for Sick Children, Toronto, Canada; ^32^Department of Pediatrics, Baylor College of Medicine, Houston, United States; ^33^Istituto Giannina Gaslini, Genova, Italy; ^34^Medical Research, RILD Welcome Wolfson Centre, Exeter Medical School, Royal Devon and Exeter NHS Foundation Trust, Exeter and Genetics and Molecular Cell Sciences Research Centre, St George's University of London, London, United Kingdom; ^35^Department of Plasma proteins, Sanquin Research and Landsteiner Laboratory AMC, University of Amsterdam, Amsterdam, Netherlands; ^36^Laboratory of Clinical Immunology and Microbiology, National Institute of Allergy and Infectious Diseases, Bethesda, United States; ^37^Institute for Transfusion Medicine, University Ulm, Ulm, Germany; ^38^Department of General Medicine, Women's and Children's Health Network, Adelaide, Australia; ^39^Norwegian National Unit for Newborn Screening, Division of Pediatric and Adolescent Medicine, Oslo University Hospital, Oslo, Norway; ^40^The Institute of Human genetics of Leipzig, Leipzig, Germany; ^41^Departments of Paediatrics, Immunology, Institute of Medical Science, University of Toronto, Cell Biology Program, The Hospital for Sick Children, Toronto, Canada; ^42^Institute for Clinical Transfusion Medicine and Immunogenetics Ulm, German Red Cross Blood Service Baden-Wuerttemberg – Hessen, Ulm, Germany; ^43^Department of Blood Cell Research, Sanquin Research and Landsteiner Laboratory AMC, University of Amsterdam, Amsterdam, Netherlands

####### **Correspondence:** Stefano Volpi

**Introduction:** Genetic defects in regulatory proteins of the cytoskeleton are known to cause different syndromes, mostly dominated by hematologic and immune phenotypes. Recently has been reported a novel syndrome of combined immunodeficiency, allergy and autoinflammation caused by mutations in the ARPC1B gene, one of the seven subunits of the ARP2/3 complex, which regulates actin polymerization.

**Methods:** We report the natural history, clinical manifestations, genetics, and immunohematological findings in 14 patients from 12 families with ARPC1B deficiency.

**Results:** Although consanguinity was not revealed by clinical history in five families, all cases carried homozygous mutations in ARPC1B. The mutations resulted in undetectable or reduced protein expression. Early-onset gastrointestinal bleeding and skin rash were common findings (7/14 and 9/14, respectively), whereas bacterial (12/14) and viral (10/14) infections (including warts, molluscum and CMV infections), vasculitis (9/14) and growth failure (10/14) became prominent features later in life. The majority of children developed severe allergy in the presence of extensive eczema (8/14) and a universal increase in IgA and IgE levels. Moderate thrombocytopenia was present in the majority of patients while overt bleeding tendency was absent after infancy. Immunophenotyping showed a B-cell lymphocytosis and abnormalities in T- and NK-lymphocyte subsets. In vitro T- and B-lymphocyte proliferation and in vivo response to vaccination were normal. Most noticeable was the strongly reduced regulatory T-cell function in those patients tested.

**Conclusion:** In conclusion, our cohort delineates the spectrum of clinical, hematological and immunological manifestations of subjects with ARPC1B deficiency. The disease appears progressive in most cases and challenging to manage clinically with prophylactic measures and immunosuppression alone.

**Disclosure of Interest**

None Declared

#### O26 ARPC1B-deficiency causes defective cell migration, loss of actin polymerization and hyperresponsiveness in zebrafish and patient-derived cells

##### Gabriella Leung^1,2^, Aleixo M. Muise^2,3^

###### ^1^Cell Biology; ^2^Gastroenterology, Hepatology and Nutrition; ^3^Inflammatory Bowel Disease Centre, Hospital for Sick Children, Toronto, ON, Canada

####### **Correspondence:** Gabriella Leung

**Introduction:** ARPC1B-deficiency is a rare paediatric genetic disorder identified in 2017 with autoinflammatory components. Primary symptoms include cutaneous vasculitis, increased susceptibility to infection, microthrombocytopenia, and colitis. ARPC1B is a component of the Arp2/3 complex which regulates branched actin polymerization. Its expression is limited to the haematopoietic compartment, however its function in macrophages and B cells has not been characterised.

**Objectives:** To determine the effect of ARPC1B-deficiency on macrophage migration and B cell activation.

**Methods:** Mutant Arpc1b-deficient zebrafish were generated by targeting CRISPR-Cas9 to exon 4 of *arpc1b* in AB zebrafish embryos, and crossing F1 mutants to *mpeg1*-GFP^+^ fish. Four days post-fertilization, tails were cut transversely just distal to the notochord, and GFP^+^ cells imaged over an 8 hr period. Cells were tracked individually and migration behaviour quantified using Volocity. EBV-transformed lymphoblasts (LCLs) were derived from three previously characterised ARPC1B patients. Patient 1 has a null mutation (c.387_388insCT, L90fs). Patients 2 and 3 are brothers with two SNPs (c.434C>T, A105V; c832G>A, A238T). LCLs were also derived from the parents of Patient 2 and 3, heterozygous for both mutations. Cell phenotype was assessed by immunoblotting, immunofluorescence, and flow cytometry. Calcium flux was analysed by flow cytometry using ratiometric dye Fura Red-AM and stimulating LCLs with anti-IgG. The VCA domain of WASP binds and activates the Arp2/3 complex and was used to stimulate pyrene-actin polymerization activity. Protein interaction between ARPC1B vs. ARPC1A to VCA and WASP were also tested by immunoprecipitation.

**Results:** ARPC1B-deficient zebrafish were significantly smaller, and monocytes migrated slower compared to WT fish in response to injury. In the patient LCLs, loss of ARPC1B protein, compensatory upregulation of isoform ARPC1A, and loss of total F-actin were confirmed. Patient 1 LCLs were unusually adherent in the absence of stimulation, suggesting constitutive activation. To measure activation, LCLs were stimulated with anti-IgG to measure calcium flux; Patient 1 had a statistically significant higher calcium flux peak compared to control. To address whether Arp2/3 function was compromised, LCL lysates were stimulated with (activating domain) VCA and actin assembly was measured. Although both ARPC1B and ARPC1A isoforms were found to interact with VCA and full-length WASP in immunoprecipitation experiments, VCA-induced actin polymerization in cell lysates was completely abolished in Patients 1-3. Patient 1 was treated with an allogeneic HSCT in January 2018.

**Conclusion:** Loss of ARPC1B leads to impaired cell migration, loss of Arp2/3 function, and hyperactivation in B cells, which together likely contribute to the dysfunctional immune phenotype. HSCT may represent a curative option for patients with severe ARPC1B-deficiency.

**Disclosure of Interest**

None Declared

### Nucleic acid sensing and interferon

#### O27 Heterozygous mutations in COPA are associated with enhanced type I interferon signalling

##### Marie-Louise Frémond^1^, Alice Lepelley^1^, Carolina Uggenti^2^, Maria José Martin-Niclos^1^, Marine Depp^2^, Vincent Bondet^3^, Darragh Duffy^3^, Gillian I. Rice^4^, Mary Brennan^5^, Caroline Thumerelle^6^, Siham Boulisfane^6^, Marie Legendre^7^, Serge Amselem^7^, Thierry Molina^8^, Nadia Nathan^9^, Yanick J. Crow^2^

###### ^1^Laboratory of Neurogenetics and Neuroinflammation, Imagine Institute, Paris, France; ^2^Centre for Genomic and Experimental Medicine, Institute of Genetics and Molecular Medicine, Edinburgh, United Kingdom; ^3^Immunobiology of Dendritic Cells, Institut Pasteur, Paris, France; ^4^Division of Evolution and Genomic Sciences, Manchester Academic Health Science Centre, Manchester; ^5^Department of Paediatric Rheumatology, Royal Hospital for Sick Children, Edinburgh, United Kingdom; ^6^Pediatrics Department, CHRU de Lille, Lille; ^7^Genetic Department and Inserm UMR S933, Trousseau Hospital-APHP and Sorbonne Université; ^8^Pathology Department, Necker Hospital-APHP; ^9^Inserm UMR S933 and Pediatric Pulmonology department and Reference Centre for Rare Lung Diseases, RespiRare, Trousseau Hospital-APHP and Sorbonne Université, Paris, France

####### **Correspondence:** Marie-Louise Frémond

**Introduction:** Heterozygous mutations in *COPA*, encoding coatomer protein subunit alpha, cause an autosomal dominant inflammatory syndrome associating lung, joint and renal disease, showing some overlap with STING-associated vasculopathy with onset in infancy (SAVI). Mutations were originally described to cause endoplasmic reticulum (ER) stress and priming of a T helper 17 response. More recently, increased transcription of interferon (IFN)-stimulated genes (ISGs) was reported in blood circulating cells of affected individuals. However, the precise pathophysiology of this disease remains unclear.

**Objectives:** To better decipher the mechanism of COPA syndrome.

**Methods:** We studied 8 patients from 3 unrelated families, each segregating a heterozygous mutation in *COPA.* We assessed type I IFN status by IFNa ultra-sensitive digital quantification in plasma, STAT1 phosphorylation and RNA expression of ISGs in whole blood from patients. *In vitro* assays also were performed in HEK293T and THP-1 cells to study IFN signalling in the context of COPA mutations.

**Results:** We observed commonalities in the lung pathology between COPA and SAVI, as well as an IFN signature, raised levels of IFNa protein in the serum and phosphorylation of STAT1 in patient T cells. In a cellular model of HEK293T, phosphorylation of IRF3 and increased ISG expression were observed in cells co-transfected with wild type STING and mutant COPA plasmids. In THP-1 cells, short hairpin RNA knockdown of COPA induced IFN signalling that was abrogated in the absence of STING.

**Conclusion:** Our data suggest that mutations in *COPA* lead to constitutive activation of type I IFN signalling through STING. Based on these results, one patient has been treated with the JAK1/2 inhibitor ruxolitinib for the last 12 months. How COPA interacts with ER-resident STING remains to be investigated.

**References**

Watkin et al, Nat Genet 2015;47:654-60.

Volpi et al, Clin Immunol 2018;187:33-36.

**Disclosure of Interest**

None Declared

#### O28 Sting-associated vasculopathy in mice requires adaptive immunity but not cGAS or type I interferon

##### Hella Luksch^1^, Angela Rösen-Wolff^1^, Alexander Gerbaulet^2^, W. Alexander Stinson^3^, Brock G. Bennion^3^, Gowri Kalugotla^4^, Wei Qian^3^, Catherine A. Miner^4^, Jonathan Miner^5^

###### ^1^Department of Pediatrics, University Clinic Carl Gustav Carus, TU Dresden; ^2^Department of Immunology, Faculty of Medicine,TU Dresden, Dresden, Germany; ^3^Department of Pathology and Immunology; ^4^Department of Medicine, Washington University School of Medicine; ^5^Departments of Pathology and Immunology, Medicine and Molecular Microbiology, Washington University School of Medicine, Saint Louis, St. Louis, United States

####### **Correspondence:** Angela Rösen-Wolff

**Introduction:** It is assumed that monogenic interferonopathies are mediated by type I interferon (IFN) inducing autoinflammation. For instance, it has been shown that a gain-of-function mutation in STING (STING N153S) up-regulates type I IFN-stimulated genes (ISGs) and results inperivascular inflammatory lung disease in mice. The corresponding mutation in humans also causes lung disease. It is thought that signaling via the cGAS-STING pathway and subsequent activation of type I IFN, IFN regulatory factors (IRF) 3/7, and ISGs are involved.

**Objectives:** We decided to characterize the role of cGAS, IRF3/7, the type I IFN receptor (IFNAR1), and adaptive immunity in the spontaneous inflammatory lung disease in STING N153S mice.

**Methods:** Hence, we crossed STING N153S mice to animals lacking cGAS, IRF3, IRF7, IFNAR1, adaptive immunity, alph/beta T cells, and mature B cells. As read out we evaluated the mice for development of spontaneous inflammatory lung disease.In addition we generated bone marrow chimeric mice and examined severity of the lung disease and survival of the transplanted animals for 322 days.

**Results:** We found that spontaneous inflammatory lung disease in STING N153S mice developed independently of cGAS, IRF3, IRF7, and type I IFN signaling. Bone marrow transplantation experiments revealed that certain aspects of STING N153S-associated disease are intrinsic to the hematopoietic system. In additon, we discovered that Rag1-/- STING N153S mice have histologically normal lungs without perivascular infiltrates.Tcr beta-/- STING N153S animals developed a mild lung phenotype.

**Conclusion:** Spontaneous inflammatory lung disease in STING N153S mice develops independently of cGAS and type I IFN signaling.STING N153S depends on adaptive immunity to induce inflammatory lung disease in mice.

**Disclosure of Interest**

None Declared

### New frontiers in the treatment of SAID

#### O29 Preclinical studies of gene therapy for deficiency of adenosine deaminase type 2 (DADA2)

##### Ying Hong, Marina S. Casimir, Barbara Jensen, Benjamin Houghton, Adrian Thrasher, Paul Brogan, Despina Eleftheriou

###### Infection, immunity and inflammation, UCL Great Ormond Street Institute of Child Health, London, United Kingdom

####### **Correspondence:** Ying Hong

**Introduction:** Deficiency of adenosine deaminase type 2 (DADA2) is an autosomal recessive autoinflammatory disease and vasculitis, caused by loss-of-function mutations in *ADA2*. Treatment with anti-TNF-α is effective for the autoinflammation and vasculitis of DADA2, but may add to the burden of immunosuppression, is expensive, and is required for life. This treatment may also not effectively treat bone marrow failure or the associated immunodeficiency. Since haematopoietic stem cell transplantation (HSCT) may be curative, but is toxic, we are currently exploring gene therapy for DADA2.

**Objectives:** (i) develop specific self-inactivating (SIN) lentivirus vectors encoding *ADA2* cDNA; (ii) explore the efficacy of gene transfer using this vector in monocyte derived macrophages (MDM) from DADA2 patients; and in an *ADA2* knockout (KO) monocyte cell-line (THP-1) model generated using CRISPR/CAS9.

**Methods:** We generated an *ADA2*-SIN lentivirus under the control of the elongation-1a-factor (EFS) promoter, that we then used to transduce *ADA2* KO THP-1/ wild type (WT) control THP-1 cells; or patient and healthy control-derived MDM, at different multiplicity of infection, and examined the following:

(i) ADA2 protein expression.
(ii) ADA2 enzyme activity
(iii) M1/M2 macrophage immunophenotype. MDM and THP-1 differentiated macrophages were incubated with IL-4, IL-10 and IL-13 to obtain M2 polarized macrophages or with IFN-γ/LPS to induce polarization to M1. Immunophenotyping was assessed in transduced or non-transduced M1 and M2 polarised macrophages using qPCR, flow cytometry, and ELISA-cytokine quantification in culture supernatants.
(iv) macrophage induced endothelial activation. Transduced or non-transduced MDM from DADA2 patients and healthy controls were also cultured with endothelial cells, and CD62E expression (marker of endothelial activation) was examined with flow cytometry

**Results:** In the THP-1 KO cell line we observed:

(i) **full restoration of ADA2 protein expression and ADA2 enzyme activity** in transduced *ADA2* KO THP-1 cells compared to non-transduced cells;
(ii) **amelioration of M1 proinflammatory molecule gene expression:** polarized M1 cells derived from transduced *ADA2* KO THP-1 cells exhibited a similar gene expression profile for proinflammatory cytokines (TNF-α, CXCL-10, STAT-1, and IL-1β) to that observed in M1 derived from WT control THP-cells; in comparison, non-transduced *ADA2* KO THP-1 cells exhibited significant up-regulation of gene expression for these proinflammatory cytokines (p<0.0001).

Using DADA2 patient derived cells (n=3) we also established:

(iii) **full restoration of ADA2 protein expression and enzyme activity:** transduction of DADA2 MDM led to full restoration of ADA2 protein expression and enzyme activity to levels observed in healthy controls (p=0.0001);
(iv) **amelioration of M1 proinflammatory gene expression in patient cells:** transduced DADA2 MDM were restored to levels seen in healthy control MDM cells for proinflammatory cytokine gene expression (p=0.0001); cytokine production at protein level (p=0.01); and reduced M2 apoptosis (p=0.0001).
(v) **prevention of endothelial activation:** transduction of DADA2 MDM significantly prevented endothelial activation in co-culture experiments, compared with non-transduced DADA2 MDM, that continued to induce significant endothelial activation (p=0.0002).

**Conclusion:** We have used a gene therapeutic approach to successfully demonstrate rescue of the immunophenotype of DADA2 using a THP-1 *ADA2* KO cell line model; and primary cells from DADA2 patients, thus providing proof of principle that gene therapy might work in patients with DADA2. Next steps now include: 1. gene correction in CD34 cells from DADA2 patients; and 2. safety studies in animal models.

**Disclosure of Interest**

None Declared

#### O30 Experience with and management of HLH-like toxicities following chimeric antigen receptor T-cell therapy for treatment of relapsed/refractory pre-B ALL

##### Amanda K. Ombrello^1^, Bonnie Yates^2^, Haneen Shalabi^2^, Terry J. Fry^3^, Nirali N. Shah^2^

###### ^1^NHGRI; ^2^NCI, NIH, Bethesda; ^3^Children's Hospital of Colorado, Denver, United States

####### **Correspondence:** Amanda K. Ombrello

**Introduction:** Chimeric antigen receptor T-cell (CAR-T) therapy is a highly effective form of adoptive cell immunotherapy combining antigen specific targeting capabilities with T-cell based cytotoxicity. Particularly effective against B-cell antigens (CD19/CD22), CAR-T cell activation leads to a systemic inflammatory response called cytokine release syndrome (CRS) that can further evolve into hemophagocytic histiocytosis (HLH) symptomatology.

**Objectives:** To evaluate the presentation, incidence and management of HLH toxicities in children/young adults with relapsed/refractory pre-B acute lymphoblastic leukemia (pre-B ALL) treated on a phase I study of anti-CD22 CAR-T cell therapy (Clinicaltrials.gov NCT02315612).

**Methods:** Using modified diagnostic criteria to define CAR-T cell related HLH, it was established in those with ferritin of > 100,000 ng/mL and at least 1 of the following: > grade 3* AST/ALT elevation or hyperbilirubinemia; > grade 3* oliguria or increase in serum creatinine; > grade 3* pulmonary edema; or hemophagocytosis in the bone marrow. Serial inflammatory markers (ferritin, CRP) and serum cytokines were prospectively monitored from CAR-T cell infusion through day +28 (+/-4) and retrospectively analyzed comparing peak values in those who did/did not develop HLH. Treatments included supportive care, glucocorticoids +/- anakinra.

*Grading as per Common Terminology Criteria for Adverse Events, v4.03

**Results:** In 52 subjects, 46 experienced CRS, of whom 37 (80.4%) achieved complete remission and 18 (39.1%) developed HLH. Median ferritin in those with/without HLH was 206740 vs. 22758 (ng/mL) (Table 1). Clinical manifestations included: > grade 3* creatinine (n=2); > grade 3* AST/ALT elevation or hyperbilirubinemia (n=23, including 6 without HLH), > grade 3* pulmonary edema (n=5, including 2 without HLH); and hemophagocytosis in the bone marrow (n=9). Cytokine profiling demonstrated significantly higher levels of IFN-y, IL-1B, IL-6, IL-10, TNFa and MIP-1a (Table 1). Limited paired samples of sIL-2R showed statistically significant increase from baseline (median level 1254) to HLH presentation (median 9310), with 6/9 having substantial increase (peak 123700). All had resolution of HLH symptoms, except 1 who died from gram-negative rod sepsis complications prior to resolution. Three had asymptomatic lab abnormalities that self-resolved (median 14 days) without intervention. Anakinra monotherapy (median 5 mg/kg/day) was used in 3 and the remainder (n=3) received anakinra + steroids. Both regimens resolved HLH. There was no statistically significant difference in underlying leukemia burden or efficacy following CAR-T cell therapy in those who did/did not develop HLH. Notably, use of anakinra +/- steroids did not diminish therapeutic efficacy of CAR-T cells.

**Conclusion:** Ferritin and cytokine profiling revealed HLH patients had a different inflammatory response independent of disease burden. Anakinra +/- steroids was effective and did not impede CAR-T cell expansion. Further analysis to identify HLH-predictive parameters and optimizing interventions to treat/prevent these complications are ongoing.

**Disclosure of Interest**

None Declared

**Table 1 (abstract O30).** See text for description

Inflammatory Marker	No HLH, median (25-75% IQR)	HLH, median (25-75% IQR)	p value (1- tailed)
Ferritin*	22758 (3554-52686)	206740 (171968-420273)	<0.0001
Cytokines* (pg/mL)			
IFNy	352.2 (196.7-1041)	2800 (1838-2900)	<0.0001
IL-1B	0.77 (0.45-2.09)	3.51 (1.02-48.95)	0.001
IL-6	41.58 (18.83-214.5)	904.5 (264.1-1480)	<0.0001
IL-10	55.94 (22.02-154)	338.7 (128.1-567.4)	0.0001
TNFa	12.77 (9.17-23.62)	27.1 (16.2-43.91)	0.002
MIP-1a	105.7(67.12-180.1)	223.8 (157-422.2)	0.0001

*For ferritin, n=19 (No HLH) and n=18 (HLH) due to initial lack of ferritin monitoring. All other cytokines, n=27 (No HLH) and n=18 (HLH)

### Novel targets and therapies in autoinflammation

#### O31 Novel NLRP3 targeted therapy in caps

##### Laela M. Booshehri^1^, Matthew McGeough^1^, Ben Keer^1^, Milos Lazic^2^, Christopher McBride^2^, Davide Povero^2^, James Veal^2^, Gretchen Bain^2^, Hal M. Hoffman^1^

###### ^1^Pediatrics, University of California San Diego, La Jolla; ^2^Jecure Therapeutics, San Diego, United States

####### **Correspondence:** Hal M. Hoffman

**Introduction:** Cryopyrin-associated periodic syndrome (CAPS) is an autoinflammatory disease characterized by a hyperactive inflammasome leading to the overproduction of interleukin-1b (IL-1b). Assembly of the NLRP3 inflammasome is central to the CAPS disease process resulting in subsequent activation and prolific release of inflammatory cytokines, which further propagate inflammation and disease. Current therapies for CAPS patients directly target IL-1b or IL-1 receptor to mitigate excess inflammation caused by NLRP3 activation. While there are a number of new compounds in pre-clinical development that target NLRP3 directly, there is little data on the efficacy of these compounds in CAPS versus healthy controls.

**Objectives:** To study the *ex vivo* efficacy of a novel NLRP3 selective small-molecule inhibitor compound 1 (C1) in monocytes from CAPS patients with multiple *Nlrp3* mutations as compared to healthy controls and in bone marrow derived dendritic cells from murine *Nlrp3* mutant CAPS models as compared to wild type mice, with the goal of identifying an effective NLRP3 specific inhibitor for CAPS patients.

**Methods:** Peripheral blood mononuclear cells from 7 CAPS patients with 6 different *NLRP3* mutations and 4 healthy donor controls were isolated by gradient centrifugation, and monocytes were allowed to adhere for 4 hours prior to treatment and stimulation. Samples were obtained under an approved Institutional Review Board protocol for human subjects. Bone marrow was isolated from MWS *Nlrp3*^*A350V/+*^ CreT, FCAS *Nlrp3*^*L351P/+*^ CreT, and NOMID *Nlrp3*^*D301N/+*^ CreT conditional knock-in mice and cells were cultured with GMCSF for 1 week and treated with tamoxifen 1 day prior to drug treatment to induce expression of the mutation. Cells were treated with C1 at varying concentrations prior to stimulation with LPS (mutants) or LPS and ATP (controls), and supernatants were collected after overnight incubation for analysis of IL-1b by ELISA. C1 was also orally administered to MWS *Nlrp3*^*A350V/+*^ CreT mice prior to *in vivo* tamoxifen administration to determine *in vivo* efficacy and pharmacodynamics. Whole body and spleen weights were measured and blood was obtained for complete blood counts in treated and untreated mice. Animal studies were performed in accordance with the University of California San Diego and IACUC policies and procedures.

**Results:** Robust inhibition of IL-1b release from C1 treated cells was shown with comparable activity across multiple human *NLRP3* mutations and murine *Nlrp3* mutant models. Compound efficacy and pharmacodynamics were also shown to be similar between CAPS mutant cells and cells from respective healthy donors or wild-type controls. Daily oral administration of C1 was well tolerated and demonstrated efficacy in preventing weight loss, splenomegaly, and neutrophilia in MWS *Nlrp3*^*A350V/+*^ CreT mice.

**Conclusion:** The novel NLRP3 inhibitor C1 was shown to significantly reduce LPS induced IL-1b release in cells from CAPS patients and murine *Nlrp3* mutant models indicating direct and effective inhibition of mutant NLRP3. C1 and other emerging NLRP3 inhibitors present an additional avenue for future CAPS patient therapy by directly targeting the inflammasome. Similar efficacy of C1 on both normal and mutant NLRP3 likewise indicate potential applications in other inflammatory diseases beyond CAPS.

**Disclosure of Interest**

None Declared

#### O32 Preclinical efficacy of NLRP3 small molecule inflammasome inhibitors: implications for future treatment of autoinflammatory syndromes

##### Angela Abad-Perez^1^, Stefan Frischbutter^1,2^, Niklas A. Mahnke^1^, Jens v. Kries^3^, Marc Nazaré^4^, Marcus Maurer^1^, Jörg Scheffel^1,2^, Karoline Krause^1,2^

###### ^1^Department of Dermatology, Venereology and Allergology; ^2^Autoinflammation Reference Center Charité (ARC2), Charité Universitätsmedizin Berlin; ^3^Screening Unit Cell Biology and High Content Screen; ^4^Medicinal Chemistry, Leibniz-Forschungsinstitut für Molekulare Pharmakologie (FMP), Berlin, Germany

####### **Correspondence:** Angela Abad-Perez

**Introduction:** Systemic autoinflammatory diseases (SAIDs) are characterized by abnormally increased inflammation affecting different organs. They are mediated predominantly by the cells and molecules of the innate immune system. Inflammasome activation represents the critical pathogenic mechanism shared by most SAIDs. Current treatment strategies are limited to downstream cytokine blockade. Specific inflammasome inhibitors are not available so far.

**Objectives:** To address this unmet medical need, we performed a high content screening of more than 60.000 small molecules from the compound collection of the …Leibniz-Forschungsinstitut für Molekulare Pharmakologie (FMP)”, which includes the ChemBioNet and LOPAC^®1280^ libraries as well as donations of academic chemists, FDA approved drugs (Selleck library) and a natural product collection from AnalytiCon Discovery.

**Methods:** For the primary screen, we used a fluorescent murine inflammasome reporter cell line to detect ASC speck formation, a marker of inflammasome activation. Compounds were selected based on their inhibitory capacity on ASC speck formation, as observed by automated fluorescence microscopy, and IL-1ß release after activation with canonical NLPR3-inflammasome inducers ATP and nigericin. The 10 most potent and druggable hit compounds were tested in peripheral blood mononuclear cells (PBMCs) obtained from venous blood of patients with Schnitzler syndrome (N=8), familial Mediterranean fever, FMF (N=8) and from unmatched healthy donors (N=18). *In vitro* effect on cellular IL-1ß release was measured by ELISA.

**Results:** Selected compounds proved to efficiently inhibit the secretion of IL-1ß in PBMCs from both patients and healthy controls in a dose dependent manner, validating their inhibitory capacities in human and murine cellular assays. Among these compounds were known anti-inflammatory drugs such as auranofin and a VEGFR2 tyrosine kinase inhibitor. The median inhibitory capacity upon stimulation with lipopolysaccharide (LPS) and ATP at 10 μM ranged between 50% to 80% with IC50s in the low μM region. A similar inhibitory profile could be observed for the previously reported inflammasome inhibitor MCC950, which was included in our assays as a reference substance. Moreover, compounds had similar efficacy in inflammasome inhibition PBMCs obtained from patients and healthy controls.

**Conclusion:** Based on our results in murine and human cells *in vitro*, small molecule inflammasome inhibitors my complement current treatment options for SAIDs in the future.

**Disclosure of Interest**

None Declared

### Oral communications – new diseases

#### O33 Biallelic loss of function mutations in sharpin cause autoinflammation

##### Hirotsugu Oda^1^, David Beck^1^, Kalpana Manthiram^1^, Hye Sun Kuehn^1^, Natalia Sampaio Moura^1^, Rao Anand^2^, Mariana Kaplan^1^, Douglas Kuhns^3^, Wanxia Li Tsai^1^, Hiroyuki Yoshitomi^4^, Junya Toguchida^4^, Gustavo Gutierrez-Cruz^1^, Jeremy Davis^1^, Massimo Gadina^1^, Jennifer Stoddard^1^, Kazuhiro Iwai^4^, Sergio Rosenzweig^1^, Luigi Notarangelo^1^, Daniel L. Kastner^1^, Ivona Aksentijevich^1^

###### ^1^NIH, Bethesda, United States; ^2^Manipal Hospital, Bangalore, India; ^3^NIH, Frederick, United States; ^4^Kyoto University, Kyoto, Japan

####### **Correspondence:** Hirotsugu Oda

**Introduction:** The linear ubiquitination chain assembly complex (LUBAC) consists of HOIP, HOIL1 and SHARPIN and mediates linear ubiquitination. LUBAC is essential for NF-κB signaling and thus proper innate and adaptive immunity. Patients with HOIP and HOIL1 deficiencies have been reported to have immunodeficiency, autoinflammation and amylopectinosis. Although mice deficient in *Sharpin* have severe TNF-dependent skin inflammation due to enhanced apoptosis and necroptosis of the keratinocytes, the role of SHARPIN in human diseases is unknown.

**Objectives:** We aimed to investigate a 14 year-old boy from a consanguineous family in India with polyarthritis, parotitis, hepatosplenomegaly and colitis associated with anorectal fistula, but without skin manifestations or any history of severe infections. The patient’s symptoms dramatically improved on anti-TNF therapy.

**Methods:** We performed whole exome sequencing to identify the genetic cause of the patient's phenotypes.

**Results:** We identified a homozygous frameshift mutation in *SHARPIN* in our proband (c.220dupC). Patient derived dermal fibroblasts have no detectable SHARPIN protein with markedly reduced HOIP, and HOIL1 suggesting destabilization of the LUBAC complex. These cells also displayed impaired canonical NF-κB activity, as exemplified by induction of IκBα phosphorylation and nuclear translocation of p65. However, in contrast to HOIP and HOIL1 deficiencies, the patient’s monocytes did not show hyperresponsiveness to IL-1β stimulation.SHARPIN deficient patient fibroblasts demonstrated enhanced apoptosis induced by FAS stimulation as compared to control cells, which parallels the enhanced apoptosis observed in the *Sharpin* deficient mice. We knocked out *SHARPIN* in a human immortalized osteoblast cell line (hFOB1.19) and interestingly, despite attenuated NF-κB activity, these cells secrete higher amounts of IL-6 more rapidly after IL-1β stimulation than control cells.

**Conclusion:** We identified the first case of human SHARPIN deficiency in a patient with autoinflammation. Molecular consequences of the SHARPIN deficiency are currently being investigated in comparison to other LUBAC deficiencies.

**Consent for publication has been obtained from patient**

Yes

**Disclosure of Interest**

None Declared

#### O34 A loss-of-function mutation in USP43, a deubiquitinase gene, is linked to an interferon-mediated autoinflammatory disorder with proteasome defects

##### Hongying Wang^1^, Qing Zhou^1^, Anna Kozlova^2^, Vasili Burlakov^2^, Daniel Kastner^1^, Ivona Aksentijevich^1^, Anna Shcherbina^2^

###### ^1^Inflammatory Disease Section, National Human Genome Research Institute (NHGRI) / NIH, Bethesda, United States; ^2^National Research and Practical Center of Pediatric Hematology, Oncology and Immunology, Moscow, Russian Federation

####### **Correspondence:** Hongying Wang

**Introduction:** Deubiquitinase enzymes (DUBs) function in the removal of poly-ubiquitin chains from substrate proteins to regulate their activity and degradation by the ubiquitin-proteasome system (UPS). Deficiency of DUB activity may lead to excessive immune signaling as has been observed in patients with haploinsufficiency of A20 (HA20) and OTULIN deficiency. Through whole exome sequencing analysis (WES), we identified a novel homozygous missense mutation (c. 2509G>A; p. E837K) in a deubiquitinase encoding gene *USP43*, in a Russian patient of Tatar ancestry. The patient presented with early-onset recurrent fevers, rash, subcutaneous skin nodules, lipodystrophy, and prominent arthritis, and this phenotype was suggestive of the CANDLE syndrome.

**Objectives:** USP43 is a poorly characterized ubiquitin specific protease (USP).We aimed to study the disease-causing mechanism underlying this novel mutation, E837K, as well as the biological function and targets of USP43.

**Methods:** We performed WES in the patient’s family and RNA sequencing in whole blood samples. We generated a fibroblast cell line and EBV-transformed B cells from the patient’s primary cells. We used USP43 knockdown and CRISPR/Cas9 knockout in 293T cells to study the effects of the USP43 depletion. A series of USP43 truncated mutants were generated to study the effect of protein domains on its DUB activity. Immunoprecipitation and immunoblot, luciferase assays, serum and plasma cytokine profiling, immunofluorescence, real-time PCR, and flow cytometry were used to investigate abnormalities in patient-derived cells.

**Results:** We found that the novel USP43 mutation leads to an upregulation in interferon signaling and causes an impairment in the proteasome-mediated protein degradation pathway. The patient’s EBV-transformed B cells had increased phospho-STAT levels in response to interferon stimulation and spontaneously produced a significantly higher level of IL-6. This cellular phenotype was rescued by transfection with wild-type USP43, which suggests that this mutation is loss-of-function.The patient’s primary cells showed decreased proteasome activity and excessive accumulation of K48-ubiquinated proteins following stimulation with a proteasome inhibitor MG132. Similarly, transient knockout of USP43 in 293T cells led to increased levels of ubiquitinated proteins. Reintroducing wildtype USP43 to EBV-transformed patient B cells markedly decreased the expression of ubiquitinated proteins. These data suggest that the mutant USP43/E837K protein loses the ability to remove K48-ubiquitin chains from target proteins. Overexpression of a series of truncated USP43 mutants with K-48 Ub chains in 293T cells confirmed that the C-terminal domain is required for the DUB function of USP43. In addition, USP43 mutants possessing the E837K mutation lost the ability to clear accumulated K48-Ub chains. Interferon-stimulated patient’s EBV cells and fibroblasts showed decreased protein levels of PSMB8.*PSMB8* encodes the catalytic subunit of the immunoproteasome. Co-transfection of USP43/E837K mutant with PSMB8 in 293T cells reduced the expression of PSMB8 precursor protein compared to cells transfected with USP43 wild type.

**Conclusion:** Our data suggest that the loss-of-function mutation in USP43 decreases immunoproteasome activity and causes an upregulation in type I interferon signaling, similar to what is observed in patients with CANDLE. Treatment with a JAK inhibitor has been very effective in controlling the disease activity in this patient. To our knowledge, this is the first report of a human disease caused by mutation in USP43.

**Disclosure of Interest**

None Declared

#### O35 A novel autoinflammatory disease characterized by neonatal-onset cytopenia with autoinflammation, rash, and hemophagocytosis (NOCARH) due to aberrant CDC42 function

##### Michael T. Lam^1,2,3^, Simona Coppola^4^, Oliver H. Krumbach^5^, Giusi Prencipe^6^, Antonella Insalaco^6^, Cristina Cifaldi^7,8^, Immacolata Brigida^9^, Serena Scala^9^, Marcello Niceta^10^, Andrea Ciolfi^10^, Alexandre F. Carisey^1,2^, Mohammad Akbarzadeh^5^, Andrea Finocchi^7,8^, Franco Locatelli^11^, Caterina Cancrini^7,8^, Alessandro Aiuti^9,12,13^, Mohammad R. Ahmadian^5^, Jordan S. Orange^2^, Fabrizio De Benedetti^6^, Marco Tartaglia^10^

###### ^1^Department of Pediatrics, Baylor College of Medicine, Houston; ^2^Department of Pediatrics, Columbia University, Irving Medical Center, New York; ^3^Translational Biology and Molecular Medicine Graduate Program and Medical Scientist Training Program, Baylor College of Medicine, Houston, United States; ^4^National Center for Rare Diseases, Istituto Superiore di Sanità, Rome, Italy; ^5^Institute of Biochemistry and Molecular Biology II, Medical Faculty of the Heinrich-Heine University, Düsseldorf, Germany; ^6^Division of Rheumatology; ^7^Department of Pediatrics, Ospedale Pediatrico Bambino Gesù, IRCCS; ^8^Department of Systems Medicine, University of Rome Tor Vergata, Rome; ^9^San Raffaele Telethon Institute for Gene Therapy (SR-TIGET), IRCCS San Raffaele Scientific Institute, Milan; ^10^Genetics and Rare Diseases Research Division; ^11^Department of Pediatric Hematology and Oncology, Ospedale Pediatrico Bambino Gesù, IRCCS, Rome; ^12^Pediatric Immunohematology, San Raffaele Scientific Institute; ^13^Vita Salute, San Raffaele University, Milan, Italy

####### **Correspondence:** Antonella Insalaco

**Introduction:** Despite continuous advances in the identification of novel causative genes, several patients with a clinical autoinflammatory phenotype remain unclassifiable.

**Objectives:** to describe a novel hematological and autoinflammatory disorder in three unrelated patients caused by a *de novo* missense mutation of *CDC42*

**Methods:** Whole exome sequencing was used to identify the novel variant. The functional impact of altered CDC42 function on hematopoiesis and inflammation was assessed through patient peripheral blood and bone marrow analyses, protein behavior and immune and non-immune cell functioning through *in vitro* biochemical and functional assays and *in vivo C. elegans* modeling.

**Results:** Patients shared the same *de novo* missense mutation of *CDC42* (NM_001791, Chr1:22417990, c.556C>T, p.R186C).Disease features included neonatal-onset cytopenia with dyshematopoiesis, autoinflammation, rash, and hemophagocytosis (collectively termed NOCARH syndrome) (Table). An altered hematopoietic compartment (prevalence of early differentiation elements and substantially decreased clonogenic progenitors) was demonstrated. Complementary assays documented the unique consequences of this mutation on CDC42 localization and function, and its disruptive effect on cell behavior and developmental processes, possibly linked to actin dysregulation. Increased secretion of IL-1β, and particularly of IL-18, was observed via *ex vivo* spontaneous release from unstimulated bone marrow mononuclear cells and by high levels in bone marrow supernatants and plasma. IFNγ was alsoincreased and correlated to CXCL9 levels which were strictly related to ferritin levels. Treatment with anakinra and emapalumab, a monoclonal antibody to IFNγ, was identified as critical in the survival of one patient, who underwent successful hematopoietic stem cell transplantation.

**Conclusion:** The p.R186C amino acid substitution in CDC42 underlies a novel, unique syndrome where CDC42 functional dysregulation has pleiotropic effects, causing hematopoietic disturbance, hyperinflammation, and immune impairment. Early recognition and control of HLH, through neutralization of IFNγ, followed by hematopoietic stem cell transplantion, appear to be crucial to survival.

**Disclosure of Interest**

None Declared

**Table 1 (abstract O35).** See text for description

Outcome and status	Patient 1	Patient 2	Patient 3
	Alive, 6 yrs	Dead, 6 mos	Dead, 1.5yrs
Fever	+	+	+
Skin rash	+	+	+
Hepato-Splenomegaly	+	+	+
Hemophagocytic lymphohistiocytosis	+	+	+
Gastrointestinal symptoms	+	+	+
Cytopenia	+	+	+
Acute phase response	+	+	+
Bone marrow dysplasia	+	+	+

#### O36 PSMB10, The last immunoproteasome gene missing for PRAAS (Proteasome-Associated Autoinflammatory Syndrome)

##### Guillaume Sarrabay^1,2^, Déborah Méchin^1,2^, Aicha Salhi^3^, Guilaine Boursier^1^, Cécile Rittore^1^, Yanick Crow^4^, Gillian Rice^5^, Tu-Ahn Tran^2,6,7^, Renaud Cezar^7^, Darragh Duffy^8^, Vincent Bondet^8^, Lakhtar Boudehane^9^, Sylvie Grandemange^1,2^, Florence Apparailly^2^, Isabelle Touitou^1,2^

###### ^1^Department of Medical Genetics, Rare diseases and Personalized medicine, Rare and Autoinflammatory diseases unit; ^2^IRMB, INSERM, CHU Montpellier, Univ Montpellier, Montpellier, France; ^3^Dermatology department, Alger medicine University, Alger, Algeria; ^4^Laboratory of Neurogenetics and Neuroinflammation, Institut Imagine, Paris Descartes University, Paris, France; ^5^Division of Evolution and Genomic Sciences, School of Biological Sciences, Faculty of Biology, Medicine and Health, University of Manchester, Manchester Academic Health Science Centre, Manchester, United Kingdom; ^6^Paediatrics department, University Hospital Nimes; ^7^Immunology department, CHU Nîmes, Univ Montpellier, Nîmes; ^8^ICD Unit, Inserm U1223, Institut Pasteur, Paris, France; ^9^Paediatrician office, Liberal, Sétif, Algeria

####### **Correspondence:** Guillaume Sarrabay

**Introduction:** PRAAS defines a clinical spectrum encompassing JMP (joint contractures, muscle atrophy, microcytic anemia and panniculitis-induced childhood-onset lipodystrophy syndrome), NNS (Nakajo-Nishimura syndrome) and CANDLE (chronic atypical neutrophilic dermatosis with lipodystrophy and elevated temperature). PRAAS is caused by autosomal recessive, autosomal dominant, or digenic mutations in several genes encoding for either constitutive proteasome subunits (*PSMB4*, *PSMA3*), immunoproteasome subunits (*PSMB8*, *PSMB9)* or chaperone protein (*POMP*). We describe here a 3-year-old female patient with clinical features evocative of PRAAS in whom no mutations have been found using our 62 gene panel sequencing that includes known PRAAS genes.

**Objectives:** The aim of the study was to identify the molecular cause responsible for the PRAAS phenotype in this patient.

**Methods:** We performed a trio-based whole exome sequencing (WES) in the patient and her parents. Functional assays were conducted to confirm the pathogenic effect of the mutated candidate gene. They included: enzymatic protease activity in the patient’s peripheral blood monocyte cells (PBMC) and in transfected HEK293T cells, interferon (IFN) signature and IFNα dosage, multiplexed cytokines measurement from patient’s serum, and protein maturation assays by WB (western-blot) analyses in transfected wild-type and mutant HEK293T cells.

**Results:** The patient is a 3-year-old female patient of Algerian descent, born to related parents. She developed a cutaneous rash on the 7^th^ day of life and became febrile at the age of one year. The rash was polymorphic, annular shaped and predominantly periorbital She failed to thrive and has long-lasting hepatosplenomegaly. She has an emaciated face, a distinctive nose, and long and gracile fingers. She had elevated acute phase reactants, microcytic anemia and hypertriglyceridemia. She exhibited partial response to steroid and methotrexate treatment and relapsed when the doses were lowered. WES revealed a homozygous missense mutation in the candidate gene *PSMB10*, located in the N-terminal part of protein which is cleaved in the mature form. This variant is absent from the GnomAD cohort, and predicted to be pathogenic according to *in silico* bioinformatic tools. The patient had a positive interferon signature and elevated IFNα protein in the serum on the one occasion tested. Cytokines multiplexed measurement showed raised IL-6, TNFα, MIG and MCP-3 in the patient’s serum whereas IL-1β level was similar to healthy pediatric controls. WB assays in HEK293T cells showed defective cleavage of the mutant protein upon IFNγ induction compared to the wild-type protein. Patient’s PBMC and mutant HEK293T cells showed an alteration in trypsin-like proteasome activity.

**Conclusion:** We report here a patient with clinical and biological criteria consistent with PRAAS, with a homozygous *PSMB10* mutation. This is the third and last immunoproteasome subunit involved in this disease, and this new gene responsible for PRAAS expands the number of genes involved in this spectrum.

**Consent for publication has been obtained from patient**

Yes

**Disclosure of Interest**

None Declared

#### O37 WNT6 mutation causes an early onset granulomatosus intestinal disease with recurrent hemophagocytic lymphohistiocytosis (HLH)

##### Claudia Bracaglia^1^, Daniela Knafelz^2^, Fiammetta Bracci^2^, Antonella Insalaco^1^, Giulia Marucci^1^, Manuela Pardeo^1^, Giusi Prencipe^1^, Ivan Caiello^1^, Antonia Pascarella^1^, Marcello Niceta^3^, Francesca Pantaleoni^3^, Andrea Ciolfi^3^, Bronislava Papadatou^2^, Marco Tartaglia^3^, Giuliano Torre^2^, Fabrizio De Benedetti^1^

###### ^1^Division of Rheumatology; ^2^Hepatology, Gastroenterology and Nutrition Unit; ^3^Genetics and Rare Diseases Research Division, IRCCS Ospedale Pediatrico Bambino Gesù, Rome, Italy

####### **Correspondence:** Claudia Bracaglia

**Introduction:** Use of NGS in patients with unclassifiable disease lies a possible approach to the identification of novel disease causing genes.

**Objectives:** We report a patient with an early onset inflammatory bowel disease with granulomatous lesions and recurrent HLH episodes carrying a missense mutation in the *WNT6* gene.

**Methods:** A trio based Whole Exome Sequencing (WES) approach was used. Cytokine levels were measured by multiplex assay and by specific ELISAs.

**Results:** Ten years old Caucasian boy affected by early onset pan-colitis from 9 months of age. Since the disease onset the patient is on glucocorticoid treatment with amino acidic enteral nutrition and oligo antigenic diet. Because of recurrent disease relapses at any attempt of glucocorticoid withdrawal, azathioprine and cyclosporine treatments were also added. At 2 years of age he received total colectomy with ileostomy. Because of insufficient disease control, treatment with a TNF-inhibitor (infliximab) was started with apparent improvement of intestinal symptoms.However, persistent granulomatous inflammatory disease of the distal portion of the ileus-rectal anastomosis persisted. Moreover, the patient presented recurrent HLH episodes that required high dose of glucocorticoid and cyclosporine-A treatment. Except one HLH episode related to a varicella zoster infection, the other HLH events were most likely triggered by his underlying inflammatory condition. During the HLH episodes levels of IL-18 were moderately elevated (10.880 pg/ml) the IFN-gamma induced chemokine CXCL9 was markedly high (21.871 pg/mL) and remained markedly elevated also during clinical and laboratory HLH remission (3.121 pg/ml and 9.929 pg/ml respectively). Considering the early disease onset, primary immunodeficiency and early intestinal bowel disease onset were genetically ruled out as well as chronic granulomatosis diseases through extensive NGS panels. WES revealed carriage of a private (MAF: 1/125568, TOPMED), predicted pathogenic (CADD: 31), homozygous variant of WNT6 (c.793G>C; p.(Asp265His); NM_006522.3). The patient is now partially controlled on low dose of oral glucocorticoid (0.1 mg/kg), cyclosporine-A (5mg/kg) and antimicrobic treatment.

**Conclusion:** WNT signalling has been primarily described as a regulatory pathway in ontogeny and homeostatic processes. Schaale at al. demonstrated that WNT6 is expressed in granulomatous lesions in the lung of *Mycobacterium tuberculosis*–infected mice. Moreover, they found that the transcription factor c-Myc is significantly induced in murine macrophages by WNT6. This identifies WNT6 as a novel factor driving macrophage polarization toward an M2-like phenotype, suggesting a role for WNT6 in macrophage differentiation. Our case suggests defective function of WNT6 might be involved in the development of a granulomatous disease. WNT6 role in macrophage differentiation and polarization might also be important in the activation of the IFN-gamma pathway and in recurrent HLH episodes.

**Reference**

K. Schaale et al. Wnt6 Is Expressed in Granulomatous Lesions of Mycobacterium tuberculosis–Infected Mice and Is Involved in Macrophage Differentiation and Proliferation. *J Immunol 2013; 191:5182-5195.*

**Consent for publication has been obtained from patient**

Yes

**Disclosure of Interest**

C. Bracaglia: None Declared, D. Knafelz: None Declared, F. Bracci: None Declared, A. Insalaco: None Declared, G. Marucci: None Declared, M. Pardeo: None Declared, G. Prencipe: None Declared, I. Caiello: None Declared, A. Pascarella: None Declared, M. Niceta: None Declared, F. Pantaleoni: None Declared, A. Ciolfi: None Declared, B. Papadatou: None Declared, M. Tartaglia: None Declared, G. Torre: None Declared, F. De Benedetti Grant / Research Support from: Novartis, Novimmune, Hoffmann- La Roche, SOBI, AbbVie, Pfizer

#### O38 NFIL3 mutations alter immune homeostasis and sensitize for arthritis pathology

##### Stephanie Humblet-Baron^1^, Susan Schlenner^2^, Emanuela Pasciuto^2^, Vasiliki Lagou^1^, Oliver Burton^2^, Teresa Prezzemolo^1^, Steffie Junius^1^, Carlos Roca^1^, Cyril Seillet^3^, Cynthia Louis^3^, James Dooley^1^, Kylie Luong^3^, Erika Van Nieuwenhove^1^, Ian P Wicks^3^, Gabrielle Belz^3^, Adrian Liston^1^, Carine Wouters^4^

###### ^1^Immunology and Microbiology, Center for Brain and disease research, KU Leuven-VIB; ^2^Immunology and Microbiology, Center for Brain and disease research, KU Leuven - VIB, LEUVEN, Belgium; ^3^Walter and Eliza Hall Institute of Medical Research, Melbourne, Australia; ^4^KU Leuven and University Hospitals Leuven, LEUVEN, Belgium

####### **Correspondence:** Stephanie Humblet-Baron

**Introduction:** Juvenile idiopathic arthritis (JIA) is the most common of the childhood rheumatic diseases. JIA is characterized as juvenile-onset persistent arthritis with no defined cause. A high degree of clinical heterogeneity is observed within the JIA group of diseases, thought to reflect a diversity in genetic and environmental factors and mechanistic drivers. JIA shows similarities to adult autoimmune diseases,but also has similarities to autoinflammatory diseases, such as genetic associations to innate inflammatory pathways and response to IL-1β blockade. The recent success in identifying monogenic causes of autoinflammatory diseases suggests that monogenic causes may also underlie a subset of JIA patients.

**Objectives:** *NFIL3* is a key immunological transcription factor, with knockout mice studies identifying functional roles in multiple immune cell types. Despite the importance of NFIL3, little is known about its function in humans.

**Methods:** Here we characterized a kindred of two monozygotic twin girls with juvenile idiopathic arthritis at the genetic and immunological level, using whole exome sequencing, single cell sequencing and flow cytometry. Parallel studies were performed in a mouse model.

**Results:** The patients inherited a novel p.M170I in *NFIL3* from each of the parents. The mutant form of NFIL3 demonstrated reduced stability *in vitro*. The potential contribution of this mutation to arthritis susceptibility was demonstrated through a pre-clinical model, where Nfil3-deficient mice upregulated IL-1β production, with more severe arthritis symptoms upon disease induction. Single cell sequencing of patient blood quantified the transcriptional dysfunctions present across the peripheral immune system, converging on IL-1β as a pivotal cytokine.

**Conclusion:** NFIL3 mutation can sensitize for arthritis development, in mice and humans, and rewires the innate immune system for IL-1β over-production.

**Disclosure of Interest**

None Declared

#### O39 EROS/CYBC1 mutations: a novel cause of chronic granulomatous disease and more

##### David C. Thomas^1^, Louis M. Charbonnier^2^, Andrea Schejtman^3^, Hasan Aldhekri^4^, Eve Coomber^5^, Elizabeth Dufficy^6^, Anne Beenken^1^, James Lee^1^, Simon Clare^7^, Anneliese Speak^7^, Adrian Thrasher^8^, Giorgia Santilli^8^, Hamoud Al-Mousa^9^, Fowzan Alkuraya^10^, Talal Chatila^11^, Kenneth Smith^1^

###### ^1^Department of Medicine, University of Cambridge, Cambridge, United Kingdom; ^2^Paediatrics, Harvard, Boston, United States; ^3^University College London, London, United Kingdom; ^4^Department of Paediatrics,King Faisal Specialist Hospital and Research Center , Riyadh, Saudi Arabia; ^5^Wellcome Trust Sanger Institute, Cambridge; ^6^Medicine, University of Cambridge, Camridge; ^7^WTSI, Cambridge; ^8^Institute of Child Health, UCL, London, United Kingdom; ^9^Paediatrics; ^10^Genetics, KFSH, Riyadh, Saudi Arabia; ^11^Paediatrics, Harvard University, Boston, United States

####### **Correspondence:** David C. Thomas

**Introduction:** The multi-subunit phagocyte nicotinamide adenine dinucle- otide phosphate oxidase generates reactive oxygen species and is crucial for host defence. Deficiencies in individual subunits (gp91phox, p22phox, p47phox, p67phox, and p40phox) cause chronic granulomatous disease (CGD), but some patients with CGD do not have mutations in these genes. We recently found that Eros, a hitherto undescribed protein, is essential for the generation of reactive oxygen species because it is necessary for protein (but not mRNA) expression of the gp91phox-p22phox heterodimer, which is almost absent in Eros-deficient mice. Eros-/- animals succumb quickly following infection with Salmonella typhimurium or Listeria monocytogenes. Eros is highly conserved and has a human orthologue CYBC1 (alias C17ORF62), hereafter referred to asCYBC1 gene and essential for reactive oxygen species (EROS) protein.

**Objectives:** We asked:

1. Whether the gene fulfilled the same function in humans.
2. Whether mutaions in juman EROS/CYBC1/C17ORF62 could cause a human disease

**Methods:** We performed CRISPR-mediated deletion of CYBC1/EROS in PLB-985 cells and identified 2 clones with 8 bp and 1 bp deletions, respectively. Neither clone expressed detectable EROS protein. We also identified a patient with a homozygous EROS/CYBC1/C17ORF62 who was subsequently diagnosed with chronic granulomatous disease secondary to this mutation.

**Results:** We show that the function of *CYBC1/EROS* is conserved in human cells. Knockout of EROS in cell lines or primary human ips derived macrophages results in abswnt gp91phox-p22phox expression and aboloishges the phagocyte respiratory burst.We also describe a case of CGD secondary to a homozygous *CYBC1/EROS*mutation that abolishes EROS protein expression. This work demonstrates the fundamental importance of *CYBC1/EROS*in human immunity and describes a novel, 6^th^cause of CGD.

However, EROS also regulates the expression of other proteins. Eros-/- macrophages also express very low levels of P2X7, a ligand gated ion channel that functions as a danger receptor by binding extracellular ATP released from damaged or dying cells. and driving activation of the NLRP3 inflammasome. We show that Eros co-immunoprecipiates with P2X7 and that P2X7 driven calcium flux and inflammasome activation are markedly abnormal in Eros-/- cells. Eros also affects T cell biology, underlining key roles beyond NADPH oxidase activation.

**Conclusion:** This work demonstrates the fundamental importance of *CYBC1/EROS*in human immunity and describes a novel, 6^th^cause of CGD as well as highlighting role of EROS that are independent of the geneartion of reactive oxygen species.

**Disclosure of Interest**

None Declared

#### O40 Cold-induced urticarial autoinflammatory syndrome related to factor XII activation

##### Jörg Scheffel^1^, Niklas Mahnke^1^, Zonne Hofman^2^, Steven de Maat^2^, Jim Wu^1^, Hanna Bonnekoh^1^, Reuben Pengelly^3^, Sarah Ennis^3^, John Holloway^3^, Martin Church^1^, Marcus Maurer^1^, Coen Maas^2^, Karoline Krause^1^

###### ^1^Charite - Universitaetsmedizin Berlin, Berlin, Germany; ^2^University Medical Center Utrecht, Utrecht, Netherlands; ^3^University of Southampton, Southampton, United Kingdom

####### **Correspondence:** Karoline Krause

**Introduction:** Early onset cold-induced urticarial rash with systemic inflammatory symptoms are hallmarks of hereditary autoinflammatory diseases caused by gene mutations of the innate immune pathway, e.g. nucleotide receptor protein 3 (NLRP3). However, in many cases genetic tests are negative, suggesting the existence of unrecognized genetic variants.

**Methods:** We studied eight members of a four-generation family, four of whom were affected. Genetic analysis involved exome sequencing followed by targeted Sanger sequencing. Functional analyses included immunoblotting, mononuclear cell stimulation and immunohistochemistry. We generated recombinant protein variants and assessed cytokines and proteins in plasma and skin.

**Results:** Affected patients had cold-induced urticarial rash, arthralgia, chills, headache and malaise associated with an autosomal-dominant inheritance. Genetic studies identified a novel deleterious variant in gene *F12* (*T859A*, resulting in p.*W268R*) which encodes coagulation factor XII (FXII). Occurrence of the mutation segregated with disease status. Immunoblotting for FXII exhibited a distinct 50kDa band that was also present in recombinant W268R-mutated proteins suggesting unusual fragmentation and spontaneous activation of FXII. Furthermore, we observed contact system activation with reduced plasma prekallikrein and profound cleavage of high molecular weight kininogen, representing bradykinin production. Skin and blood neutrophils were found to be a prominent source of FXII. Interleukin-1ß (IL-1ß) was upregulated in lesional skin and in mononuclear cells of healthy donors exposed to recombinant proteins. In accordance with these findings, treatment with icatibant (bradykinin-B2-antagonist) or anakinra (interleukin-1-antagonist) reduced disease activity in patients.

**Conclusion:** We identified a novel autoinflammatory syndrome characterized by a substitution in the *F12* gene resulting in activation of the contact system and cytokine-mediated inflammation.

**Disclosure of Interest**

None Declared

## Poster presentations – Monday 1 April

### Guided poster tour 1A

#### PT1A01 Multi-omics analysis of ADA2 deficiency in Japanese cohort

##### Hiroshi Nihira^1^, Kazushi Izawa^1^, Takahiro Yasumi^1^, Moeko Ito^2^, Sachiko Iwaki-Egawa^2^, Yoji Sasahara^3^, Hirokazu Kanegane^4^, Tadateru Yasu^5^, Tomohiro Kubota^6^, Syuji Takei^6^, Dai Keino^7^, Etsuro Nanishi^8^, Hidetoshi Takada^9^, Shoichi Ohga^8^, Syunsuke Kajikawa^10^, Makio Takahashi^11^, Naoko Nakano^12^, Osamu Ohara^13^, Toshio Heike^14^, Junko Takita^1^, Ryuta Nishikomori^1^

###### ^1^Pediatrics, Kyoto University, Kyoto; ^2^Life Sciences, Hokkaido University of Science, Sapporo; ^3^Pediatrics, Tohoku University, Sendai; ^4^Pediatrics, Tokyo Medical and Dental University, Tokyo; ^5^Pediatrics, Nagasaki Medical Center, Omura; ^6^Pediatrics, Kagoshima University, Kagoshima; ^7^Pediatrics, St. Marianna University, Kawasaki; ^8^Pediatrics, Kyusyu University, Fukuoka; ^9^Pediatrics, Tsukuba University, Tsukuba; ^10^Neurology, Kyoto University, Kyoto; ^11^Neurology, Osaka Red Cross Hospital, Osaka; ^12^Pediatrics, Ehime University, Toon; ^13^Applied Genomics, Kazusa DNA Research Institute, Kisarazu; ^14^Pediatrics, Hyogo Prefectural Amagasaki General Medical Center, Amagasaki, Japan

####### **Correspondence:** Hiroshi Nihira

**Introduction:** Adenosine deaminase type 2 deficiency (DADA2) is caused by recessive loss-of-function variants in *ADA2*. Most of DADA2 patients reveal systemic vasculopathy consistent with polyarteritis nodosa and large phenotypic variability has been reported [1, 2, 3]. However, pathogenesis of DADA2 remains unclear.

**Objectives:** The objective of this study is to reveal clinical and genetic characteristics of Japanese DADA2 patients, and to gain insight into the pathogenesis of DADA2 by multi-omics analysis.

**Methods:** We performed the genetic analysis of the *ADA2* gene and measured ADA2 activity of the patients from 2016 to 2018 in Japan. Multi-omics analysis had been done in 4 out of 8 DADA2 patients and 4 healthy donors using their peripheral blood mononuclear cells (PBMCs). The samples were taken before and after introduction of anti-TNFα agents (meaning acute and remission phase) in the patients.

**Results:** We found 8 DADA2 patients. In this cohort, central neurological manifestations were present in 5 (63%), including asymptomatic small lacunar infarction. Seven subjects (88%) had livedo racemose, but there was no digital ulcer or necrosis. Low levels of IgG and IgM were revealed in 3 (37.5%) and 5 (62.5%) patients respectively, but there was no recurrent infectious episode in this case series. There were two (25.0%) who revealed pure red cell aplasia (PRCA); one revealed only anemia without any inflammation, the other revealed anemia transiently and recovered from it spontaneously but he revealed chronic inflammation afterwards. All 8 patients received anti-TNFα agent and all except one with CsA-dependent PRCA were well controlled.

We identified 6 previously described and 4 novel variants in *ADA2*, which included two that we reported before [3]. Overexpression of *ADA2* variant constructs in HEK 293 cells showed that some variants had comparable protein expression levels to wild-type in cell lysate but most of them were not secreted and all the variants had low or absent ADA2 enzyme activities.

In multi-omics analysis, differentially expressed (DE) genes were analyzed at the mRNA and protein levels. We found 64 and 58 genes that were differentially expressed in acute phase vs control and remission phase vs control in common at the transcriptome and proteome levels respectively. Gene ontology analysis of these datasets revealed constitutive up-regulation of type 1 and type 2 interferon pathway. Some genes were common to both datasets.

**Conclusion:** We have found 8 DADA2 patients in Japan and identified some novel disease-causing variants. Using multi-omics analysis, we also have found differentially expressed genes in DADA2 patients. Some genes were consistently up-regulated even in the remission period. This may provide further insights into the pathogenesis of DADA2.

References

[1] Zhou Q., *et al*. N Engl J Med, 2014.

[2] Navon Elkan P., *et al*. N Engl J Med, 2014.

[3] Meyts I., Aksentijevich I. J Clin Immunol, 2018.

[4] Nihira H., *et al*. Scand J Rheumatol, 2017.

**Disclosure of Interest**

None Declared

#### PT1A02 The clinical and immunological profiles of haploinsufficiency of A20 in Japan

##### Hidenori Ohnishi^1^, Tomonori Kadowaki^1^, Norio Kawamoto^1^, Tomohiro Hori^1^, Kenichi Nishimura^2^, Chie Kobayashi^3^, Tomonari Shigemura^4^, Shohei Ogata^5^, Yuzaburo Inoue^6^, Tomoki Kawai^7^, Eitaro Hiejima^7^, Kazushi Izawa^7^, Tadashi Matsubayashi^8^, Kazuaki Matsumoto^9^, Masatoshi Takagi^9^, Kohsuke Imai^9^, Ryuta Nishikomori^7^, Shuichi Ito^2^, Toshio Heike^7^, Osamu Ohara^10^, Tomohiro Morio^11^, Hirokazu Kanegane^12^, Toshiyuki Fukao^1^

###### ^1^Pediatrics, Gifu University Graduate School of Medicine, Gifu; ^2^Pediatrics, Yokohama City University, Kanagawa; ^3^Child Health, Faculty of Medicine, University of Tsukuba, Ibaraki; ^4^Pediatrics, Shinshu University School of Medicine, Matsumoto; ^5^Pediatrics, Kitasato University Hospital, Kanagawa; ^6^Allergy and Rheumatology, Chiba Children’s Hospital, Chiba; ^7^Pediatrics, Kyoto University Hospital, Kyoto; ^8^Pediatrics, Seirei Hamamatsu General Hospital, Shizuoka; ^9^Community Pediatrics, Perinatal and Maternal Medicine, Graduate School of Medical and Dental Sciences, Tokyo Medical and Dental University, Tokyo; ^10^Applied Genomics, Kazusa DNA Research Institute, Chiba; ^11^Pediatrics and Developmental Biology; ^12^Child Health and Development, Graduate School of Medical and Dental Sciences, Tokyo Medical and Dental University, Tokyo, Japan

####### **Correspondence:** Hidenori Ohnishi

**Introduction:** A20, encoded by the *TNFAIP3*gene, is a negative regulator of the tumor necrosis factor (TNF)-nuclear factor (NF)-κB signaling pathway. Recently, the haploinsufficiency of A20 (HA20) caused by heterozygous mutations in the *TNFAIP3*gene was identified to cause early onset autoinflammatory disease resembling Behçet’s disease.

**Objectives:** In this study, we performed a multicenter survey investigating HA20 patients found in Japan and characterized immunological profile.

**Methods:** We summarized the detailed clinical manifestations, genetic analyses and several immunological parameters of Japanese patients with HA20. Serum cytokine levels and the production levels of IL-1β and TNF-α from the peripheral blood mononuclear cells (PBMCs) were measured using ELISA. Multicolor flowcytometry analysis was performed. To detect A20 protein expression, immunoblot analysis was performed for PHA blast derived from the patients and A20 transfected HEK293T cells. The NF-κB reporter gene activity was analyzed using the dual-luciferase reporter assay system.

**Results:** A total 32 patients from 10 independent families were enrolled in this study. Age of onset was 0 to 20 years. Three mutations in the *TNFAIP3*gene were previously reported; however, seven were novel. All these mutations were evaluated to be functionally pathogenic by several *in vitro*assays. The production levels of proinflammatory cytokines such as TNF-α and IL-1β from PBMCs were increased. In the detailed analysis of lymphocyte subsets including T, B and NK cells, regulatory T cells (Treg) were increased in all analyzed patients. The increase of double-negative T (DNT) and T helper 17 cell (Th17) cells were observed in 7 and 3 out of 18 analyzed patients, respectively. In addition, follicular helper T cells (Tfh) were significantly increased especially in younger patients. Memory B cells were decreased in most of analyzed patients. Intriguingly, in the complications of HA20 patients, 9 out of 32 of them had not only autoinflammatory phenotypes but also several autoimmune disorders including psoriatic arthritis, Hashimoto’s thyroiditisand autoimmune lymphoproliferative syndromewere observed. The immune dysregulation derived from the defect of A20 may cause the increase of the autoimmune related T cell subsets and the secondary Treg expansion as well as the phonotype of the previously reported A20 knockout mice.

**Conclusion:** Our study revealed unexpected variation in phenotypes of HA20 including autoimmunity. In the analysis of lymphocyte subsets for HA20 patients, the characteristic findings such as the increase of Treg and the decrease of memory B cells were observed. The increase of DNT, Th17 and Tfh cells may be involved in onset of several autoimmune disorders.

**Disclosure of Interest**

None Declared

#### PT1A03 Use of SIGLEC1/CD169 as a biomarker for monogenic interferonopathies

##### Banu Orak^1,2^, Axel Panzer^3^, Manuela Theophil^3^, Elke Krüger^4^, Frédéric Ebstein^4^, Barbara Zieba^4^, Nadine Unterwalder^5^, Christian Meisel^5^, Tilmann Kallinich^2^

###### ^1^Center for chronically sick children, Charité University Medicine Berlin; ^2^Department of Pediatrics, Division of Pneumology, Immunology with intensive Medicine, Charité University Medicine Berlin; ^3^Pediatric Neurology, DRK Klinikum Berlin-Westend, Berlin; ^4^Institute of Medical Biochemistry and Molecular Biology, University Medicine Greifswald, Greifswald; ^5^Department of Immunology, Labor Berlin GmbH, Berlin, Germany

####### **Correspondence:** Banu Orak

**Introduction:** Monogenic Interferonopathies represent a rare group of inflammatory diseases with difficulties in early diagnosis. Expression of SIGLEC1, also known as CD169, on monocytes is the second highest interferon stimulated gene (ISG) in systemic lupus erythematodes (SLE).A correlation of SIGLEC1 expression with ISG in SLE is well established. Furthermore, SIGLEC1 seems to estimate disease activity more accurately than anti-dsDNA antibodies.

**Objectives:** To show the relevance of SIGLEC1 as a diagnostic marker for detection of Interferonopathies.

**Methods:** Eight patients with genetically confirmed monogenic Interferonopathies were included. Clinical data, classical inflammatory markers and blood count were obtained by patients file. SIGLEC1 expression was measured by flow cytometry with a highly standardized quantitative assaywith a reference range in healthy controls less than 2500 SIGLEC1 molecules/monocyte.In order to quantify the antigen expression by every single cell QuantiBRITE™ PE tubes were applied. Additionally, transcriptional level of SIGLEC1, IFI44L, IFI27, ISG15 and RSAD2 as type I Interferon stimulated genes were assessed by real-time PCR.

**Results:** All patients showed homozygous mutations. Three patients displayed TREX-1, two patients IFIH-1, two patients SAMDH1 and one patient RNASE2HB mutations. Mean age of patients was 12 years (min. 6 months, max. 49 years, SD+/- 17 years). Six of eight patients showed neurological symptoms consistent with Aicardi-Goutières-Syndrome like neurological development retardation and microcephaly. Five patients showed abnormalities on brain MRI, like periventricular calcifications or corpus callosum thinning. Two patients (homozygous for IFIH-1 mutation) were diagnosed with Singleton-Merten-Syndrome presenting abnormal ossification of extremities and dental anomalies.One patient with homozygous TREX1 mutation presented with postnatal glaucoma, microcephaly, developed sensorimotor polyneuropathia and suffered from recurrent fever with persistent chilblain lesions.

All eight patients (100%) showed elevated results for SIGLEC1 expression (mean molcules/monocyte +/- SD: 10272 +/- 3746) without having high levels of standard inflammatory markers. In six patients elevated SIGLEC1 expression showed dysregulation of the type 1 interferon pathway prior to genetic testing. In three patients with unclear disease phenotype measuring expression of SIGLEC1 contributed to establish the right diagnosis. On transcriptional level SIGLEC1 and the other ISGs were also elevated in comparison to healthy controls.

**Conclusion:** In all patients with monogenic Interferonopathies like Aicardi-Goutières-Syndrome high expression of SIGLEC1 was observed, either before diagnosis was established or during disease course.

Therefore, SIGLEC1 qualifies as an easy accessible and cheap diagnostic marker with short turnaround time to screen patients with suspected Interferonopathy.

**Disclosure of Interest**

None Declared

#### PT1A04 Screening of patients with idiopathic polyarteritis nodosa, granulomatosis with polyangiitis, and microscopic polyangiitis for deficiency of adenosine deaminase 2

##### Oskar Schnappauf^1^, Monique Stoffels^2^, Ivona Aksentijevich^1^, Amanda Ombrello^1^, Natalia Sampaio Moura^1^, Karyl Barron^1^, Daniel Kastner^1^, Peter Grayson^3^, Peter Merkel^4^, on behalf of Vasculitis Clinical Research Consortium

###### ^1^National Human Genome Research Institute (NHGRI), National Institutes of Health (NIH); ^2^National Human Genome Research Institute (NHGRI), National Institutes of Health; ^3^National Institute of Arthritis and Musculoskeletal and Skin Diseases (NIAMS), National Institutes of Health (NIH), Bethesda; ^4^Division of Rheumatology and the Department of Biostatistics, Epidemiology, and Informatics (DBEI), University of Pennsylvania, Philadelphia, United States

####### **Correspondence:** Oskar Schnappauf

**Introduction:** Deficiency of adenosine deaminase 2 (DADA2) is the first described monogenic vasculitis. Patients usually present in childhood, but age of onset, disease severity, and organ involvement of DADA2-associated vasculitis is highly variable. Clinical manifestations of DADA2 overlap with the typical features of necrotizing vasculopathies such as polyarteritis nodosa (PAN), granulomatosis with polyangiitis (GPA), and microscopic polyangiitis (MPA).

**Objectives:** This study aimed to test the prevalence of DADA2 in patients with presumed idiopathic PAN, GPA or MPA.

**Methods:** Patients (n=117) with idiopathic PAN, all of whom tested negative for hepatitis B virus infection, and patients (n=1107) with GPA or MPA were screened for mutations in *ADA2*. To further assess the pathogenicity of identified variants on a functional level, ADA2 activity was determined on available serum samples of patients with PAN.

**Results:** Nine of 118 patients with PAN (7.6%) were identified as having rare missense variants in *ADA2* with a minor allele frequency of < 0.005. Four patients (3.4%) were homozygous or compound heterozygous for variants in *ADA2*. Of the seven distinct variants present in these four patients, G47A, G47W, R169Q, E328K, F355L, and G383S had previously been reported as causative for DADA2. The remaining variant, P106S, is a rare variant predicted to be damaging to protein function by *in silico* algorithms. Five additional patients were carriers for the monoallelic variants R34W, T65M, M309I, V349I, and Y453C. R34W and Y453C were reported in DADA2 before, while the three remaining variants are of unknown clinical significance. None of the patients with GPA or MPA were biallelic for rare missense variants in the *ADA2* gene but 32 individuals (2.9%) were carriers for monoallelic rare missense variants.

Serum samples of patients with PAN were available on the individuals with the G383S/G383S and E328K/F355L genotypes and showed markedly reduced ADA2 enzyme activity, comparable to levels seen in patients with DADA2. ADA2 activity of three of the four available serum samples on monoallelic carriers was not reduced, confirming the non-pathogenicity of T65M, M309I, and V349I. The serum sample on the individual carrying the pathogenic variant Y453C showed ADA2 activity in the range of carriers. ADA2 enzyme activity testing of the remaining serum samples revealed one additional individual with strongly reduced ADA2 activity levels as well as five individuals with enzymatic activity in the range of carriers. Sanger sequencing of the *ADA2* gene in these individuals did not identify any pathogenic variants and indicates the presence of cryptic mutations undetectable by conventional sequencing. In summary, for five out of 118 patients with PAN the diagnosis of DADA2 can be applied.

**Conclusion:** This is the first study to report biallelic pathogenic variants in *ADA2* in patients with adult-onset, idiopathic PAN, and demonstrates that DADA2 specifically accounts for a subset of patients with idiopathic PAN but not GPA or MPA. Given the potential efficacy of TNF-inhibitors in DADA2, that anti-TNF treatment is not the conventional therapy in PAN, and the consequences for other family members, these findings suggest that *ADA2* testing and/or ADA2 activity testing should be considered in patients with HBV-negative idiopathic PAN, especially in patients with an early onset of this potentially life-threatening disease.

**Disclosure of Interest**

None Declared

#### PT1A05 Diagnosis and long term management of type I interferonopathies in a pediatric rheumatology center

##### Stefano Volpi^1,2^, Elettra Santori^3^, Margherita Ricci^1^, Paolo Picco^1^, Alessandra Tesser^4^, Gillian I. Rice^5^, Roberta Caorsi^1^, Alice Grossi^6^, Isabella Ceccherini^6^, Alberto Martini^2^, Yanick J. Crow^7,8^, Alberto Magnasco^9^, Alberto Tommasini^4^, Fabio Candotti^3^, Marco Gattorno^1^

###### ^1^Centro per le Malattie Infiammatorie e Immunodeficienze, Clinica Pediatrica e Reumatologia, Istituto Giannina Gaslini; ^2^DINOGMI, Università degli Studi di Genova, Genova, Italy; ^3^Allergy and Immunology, Lausanne University Hospital, Lausanne, Switzerland; ^4^IRCCS Buro Garofalo, Trieste, Italy; ^5^Genetic Medicine, Manchester Academic Health Science Centre, University of Manchester, Manchester, United Kingdom; ^6^UOC Genetica Medica e UOSD Genetica e Genomica delle Malattie Rare, Istituto Giannina Gaslini, Genova, Italy; ^7^Centre for Genomic and Experimental Medicine, MRC Institute of Genetics and Molecular Medicine, University of Edinburgh, Edinburgh, United Kingdom; ^8^Institute Imagine, University Paris Decartes, Paris, France; ^9^Nephrology, Dialysis, Transplantation Unit, Istituto Giannina Gaslini, Genova, Italy

####### **Correspondence:** Stefano Volpi

**Introduction:** in recent years several genetic diseases linked to pathologic activation of type 1 interferon (IFN) pathway have been described

**Objectives:** To identify type I interferonopathies in a cohort of children with early-onset rheumatic disease and to report the long-term efficacy and side effect of pathway-specific treatment with a Janus Kinase (JAK) inhibitor

**Methods:** Patients were selected based on the presence of any of the following: i) early onset (infancy or before puberty) inflammatory SLE-like symptoms; ii) vasculopathy (skin ulcers, chilblains, strokes) iii) panniculitis with or without lipodystrophy iv) persistent or recurrent systemic inflammation with or without lung involvement v) polyarthritis with lung involvement. Type 1 IFN activation was assessed by quantitative PCR, measuring the expression of 6 type 1 interferon-related genes (IFI27, IFI44L, IFIT1, ISG15, RSAD2, SIGLEC1) in peripheral blood. In selected patients, molecular analysis was performed using Sanger sequencing, a NGS panel of 41 inflammatory-related genes or whole exome sequencing. Off label therapy with the JAK inhibitor Ruxolitinib was considered in patients with a definitive genetic diagnosis and incomplete disease control by standard treatment

**Results:** We screened 324 patients evaluated in the rheumatology unit of our Institute. 132 out of 324 patients had a positive type 1 IFN signature. Based on the clinical presentation and the result of the IFN signature we further analyzed a subset of patients for an underline genetic defect. In 6 patients we identified pathogenic mutations affecting *TMEM173* (p.V155M in one patient and p.R281Q in a second patient), *DNASE2* (p.D121V), *DNASE1L3* (c.289_290delAC in 2 patients) and *COPA* (p.R233H) genes were identified. Therapy with Ruxolitinib was started in 4 patients and allowed to partially control disease manifestations and reduce or stop steroids, however, we observed the following limitation: the patient with *DNASE1L3* mutations and a severe kidney involvement at therapy onset, despite the control of his systemic symptoms progressed to kidney failure; one patient with SAVI and a severe lung involvement experienced severe recurrent viral lung infections requiring intensive care and extracorporeal membrane oxygenation (ECMO) in one episode; the patient with *DNASE2* mutationsexperienced a Herpes Zoster infection controlled with antiviral therapy and decreasing Ruxolitinib dosage.

**Conclusion:** Patients with type 1 interferonopathies might present with symptoms overlapping pediatric rheumatic diseases. The combination of type 1 interferon pathway activation assessment and NGS is an effective strategy for the diagnosis of this heterogeneous class of diseases and can guide the choice for targeted therapies. JAK inhibitors represent a therapeutic resource in controlling these difficult-to-treat patients, however further studies are needed to assess their efficacy and long-term safety

**Disclosure of Interest**

None Declared

#### PT1A06 Results of international Delphi survey for the diagnosis, investigation and management of deficiency of ADA2

##### Taryn A.-B. Youngstein^1^, Eugene P. Chambers^2^, on behalf of DADA2 Foundation, Helen J. Lachmann^1^, DADA2 Delphi Study Participants

###### ^1^National Amyloidosis Centre, UCL Division of Medicine, London, United Kingdom; ^2^Vanderbilt University Medical Centre, Vanderbilt University, Nashville, United States

####### **Correspondence:** Taryn A.-B. Youngstein

**Introduction:** DADA2 is phenotypically heterogenous in its presentation, even within families with the same mutation, and the absence of a murine ortholog has placed emphasis on the study of known cases of the disease.

**Objectives:** We conducted an E-Delphi study to explore consensus on the diagnosis, investigations and management of those with suspected disease.

**Methods:** A Delphi method over two rounds of consensus building was used. Invitation email to participate in the study was sent to all published authors on DADA2 and physicians and scientists known to the DADA2 Foundation, a patient advocacy group supporting research into DADA2.Consensus was defined as > 80% agreement.

**Results:** 69 respondents contributed to Round 1 and 53 respondents in Round 2, 75% of respondents were paediatricians, the rest adult physicians, a nurse specialist and basic scientists.

Consensus was reached in 1. Suggestive diagnostic features; Livedoid Rash, Stroke, Fever, Digital Gangrene, 2. The combined use of ADA2 gene sequencing and ADA2 enzyme activity levels to confirm diagnosis. 3. Use of anti-TNF therapy in acute and chronic presentations, 4. Indefinite use of anti-TNF until more information is available, and 5. The avoidance of anticoagulation.

A wide variety in practice was identified in the use of baseline and follow up investigations including imaging.

**Conclusion:** There is consensus that rash, stroke, fever, digital gangrene are highly suggestive of DADA2 but there may be a separate haematological phenotype.Gene sequencing and ADA2 activity levels in combination are the diagnostic gold standard but not generally available.There is not yet consensus on the baseline screening investigations, such as neuroimaging, for these cases. Follow-up investigations are currently guided by clinical course and practice remains highly variable.

92% of treating physicians believe that anti-TNF therapy is the correct treatment approach currently, and its use should be indefinite until more information is available.

The authors recommend the creation of a detailed international case registry and a standardised series of baseline and follow-up investigations during this accelerated learning phase about this recently described disease.

**Disclosure of Interest**

None Declared

#### PT1A07 Somatic mutations in the NLRP3-inflammasome gene in late adulthood-onset chronic urticaria

##### Eman Assrawi^1^, Camille Louvrier^1^, Fawwaz Awad^1^, Clemence Lepelletier^2^, JD Bouaziz^2^, William Piterboth^1^, Florence Moinet^3^, Philippe Moguelet^4^, Claire Jumeau^1^, Laetitia Cobret^1^, Elma El-Khouri^1^, Philippe Duquesnoy^1^, Marie Legendre^1^, Sophie Georgin-Lavialle^5^, Gilles Grateau^5^, Sonia Athina Karabina^1^, Serge Amselem^1^, Irina Giurgea^1^

###### ^1^Sorbonne Université, inserm UMRS 993; ^2^Hôpital Saint-Louis , Service de Dermatologie, Paris; ^3^Centre Hospitalier Universitaire de Martinique, Service de médecine interne, Martinique; ^4^Hôpital Tenon , Anatomie et cytologie pathologiques; ^5^Hôpital Tenon, Service de médecine interne, Paris, France

####### **Correspondence:** Eman Assrawi

**Introduction:** Chronic urticaria is a common dermatological disorder and one of the most prominent symptoms of Cryopyrin-Associated Periodic Syndrome (CAPS), a systemic autoinflammatory condition. Besides urticaria, CAPS cardinal symptoms are fever, arthralgia and deafness; however, absence of pathognomonic symptoms makes this diagnosis challenging. *NLRP3*, the disease-causing gene, encodes the cryopyrin, which upon activation initiates NLRP3 inflammasome assembly and proinflammatory cytokine secretion. Familial cases of CAPS are due to heterozygous germ-line *NLRP3* mutations; however, sporadic cases, more often identified in children, are related to *de novo* or somatic *NLRP3* mutations.

**Objectives:** We aimed to establish the etiological diagnosis of two elderly unrelated patients presenting with idiopathic chronic urticaria for two decades.

**Methods:** Molecular study of patients’ DNA was performed using a NGS panel targeting genes involved in autoinflammatory disorders. Functional analyses of the identified *NLRP3* variants were performed using two cellular models, HEK293T cells (stably expressing ASC-GFP and pro-caspase1-FLAG) to study ASC speck formation, and THP1 cells to assess IL1β secretion.

**Results:**

In two sporadic unrelated patients, we identified two mosaic *NLRP3* mutations: a novel in-frame deletion (c.926_934del, p.Gly309_Phe311del) and a recurrent CAPS mutation (c.1705G>A, p.Glu569Lys). In whole blood DNA, the mosaicism level was of 17.2% in the first patient and of 11% in the second one. The patients, who are about 70 years old, presented for two decades with late onset chronic idiopathic urticaria, occasionally associated with fever, arthralgia and myalgia. Deafness was diagnosed at the age of 50 years in the first patient, and of 70 years, after the identification of a *NLRP3* mutation, in the second one.

To assess the pathogenicity of the identified variants, we studied the activation of NLRP3 inflammasome after transient expression of NLRP3 wild-type (WT) or of NLRP3 carrying either the p.Gly309_Phe311del or the p.Glu569Lys mutation. Both mutations were found to significantly increase ASC speck formation andIL1β secretion as compared to NLRP3-WT.

The diagnosis of CAPS was therefore established on the bases of these molecular and functional data. Accordingly, complete remission was achieved with anti-interleukin 1 receptor antagonists in both patients.

Finally, we studied the mosaicism level in several cell types from both patients and showed a wide distribution profile of the mutant alleles, suggesting that, in both cases, the mutational event occurred early during embryogenesis.

**Conclusion:** In late adulthood-onset chronic urticaria, the search for autoinflammatory markers and for somatic *NLRP3* mutations may have important diagnostic and therapeutic issues. Importantly, despite the onset of the disease after 50 years old, *NLRP3* mutations are not restricted to myelomonocytic cells.

**Disclosure of Interest**

None Declared

#### PT1A08 Idiopathic recurrent pericarditis: clinical findings and treatment approach

##### Camilla Celani^1^, Silvia Federici^1^, Anna Tulone^1^, Brigitte Bader Meunier^2^, Virginia Messia^1^, Manuela Pardeo^1^, Claudia Bracaglia^1^, Pierre Quartier Dit Maire^2^, Fabrizio De Benedetti^1^, Antonella Insalaco^1^

###### ^1^Division of Rheumatology, IRCCS, Ospedale Pediatrico Bambino Gesù, Rome, Italy; ^2^Unité d’Immunologie-Hématologie et Rhumatologie pédiatrique, Hôpital Necker-Enfants, Paris, France

####### **Correspondence:** Camilla Celani

**Introduction:** Recurrent pericarditis affects 15-30% of patients with acute pericarditis. The etiology is poorly understood, with about 80% being idiopathic. Several treatment options are available for recurrences, including NSAIDs, colchicine, glucocorticoides and IL-1 inhibitors (i.e. Anakinra). Standardized guidelines for the management of these patients are still lacking

**Objectives:** To analyze clinical findings and treatment approach in a cohort of pediatric patients with recurrent pericarditis

**Methods:** Patients with at least two episodes of idiopathic pericarditis, followed at two Pediatric Rheumatology centers between 2006 and 2018, were included

**Results:** A total of 42 patients (18 males ) were included. Mean age at disease onset was 11.8 years (range 4-17). Chest pain and fever were the presenting symptoms in all patients. In 47% pleural effusion was detected. Laboratory tests showed increased white blood cell count (mean 14.509/mm3), C-reactive protein (mean 18.01 mg/dl) and erythrocyte sedimentation rate (mean 39 mm/h) in all patients. The first episode was variably treated: 18/42 (43%) received NSAIDs alone, 5/42 (11.9%), colchicine alone or associated to NSAIDs and 3/42 patients (7%) received antibiotics alone. 16/42 (38%), not responsive to NSAIDs or colchicine, received glucocorticoides. Patients who received glucocorticoids at the first episode relapsed earlier (median time of 2.1 months range 10 days-5 months), than patients treated with NSAIDs ( 6.6 months range 10 days -24 months) or with colchicine (5 months range 10 days-5 months) (p<0.05). In our study, initial treatment of the first episode did not affect the number of subsequent flares. To evaluate treatment strategy at relapses, we divided our study population in two groups: Group 1 (20 pts) in which recurrence was treated with NSAIDs, colchicine or glucocorticoid (alone or combined); group 2 (22 patients) in which anakinra was started. Among patients belonging group 2, 9 received anakinra at first relapse, 7 at the second, 2 at the third and 2 at the fourth. Anakinra treatment was followed by a prompt resolution of symptoms and inflammatory signs within 2 days. During daily treatment with full dose anakinra, no relapses were reported over a median of 13.3 months (range 5-24 months). In 13 out of 22 patients, anakinra was gradually tapered reducing the days of administration during the week. Four of these patients relapsed. The mean time from the start of anakinra to tapering was 17±4 months (range 14-23 months) in the 4 patients who experienced a relapse versus 14±4 months (range 7-21 months) in patients who did not flare, with no statistical difference. Among the 22 patients belonging to group 2 anakinra was finally discontinued in 11 after a mean time of 23.4 months (range 12-36). Among these, 8 relapsed after anakinra withdrawal (including 2 of the 4 patients already relapsed during tapering). Only 3 patients didn’t present any relapse (up to 20.3 months of follow-up). All patients who relapsed responded quickly to the reintroduction of anakinra

**Conclusion:** Our study confirms the lack of a standardized treatment approach in patients with recurrent pericarditis. Patients treated with glucocorticoid at first episode relapse before than those treated with other drugs. Anakinra is an effective treatment; however, tapering/discontinuation of the drug lead to relapses in several cases. Further experience on larger population is needed to define the best treatment duration and approach to withdrawal of IL-1 inhibitor

**Disclosure of Interest**

None Declared

### Guided poster tour 1B

#### PT1B01 Monocytes proteomic profile of patients with different autoinflammatory diseases: a new approach to characterize these diseases

##### Federica Penco^1^, Andrea Petretto^2^, Chiara Lavarello^2^, Ilaria Gueli^3^, Arinna Bertoni^1^, Alessia Omenetti^3^, Claudia Pastorino^1^, Marco Gattorno^1^

###### ^1^Centro Malattie Autoinfiammatorie ed Immunodeficienze; ^2^Laboratorio Core Facilities - Proteomica e Metabolomica Clinica; ^3^Clinica Pediatrica e Reumatologica, Istituto Giannina Gaslini, Genova, Italy

####### **Correspondence:** Federica Penco

**Introduction:** Autoinflammatory diseases are a group of inherited diseases characterized by early onset and systemic inflammation, often manifesting with unexplained fevers.These pathologies are usually caused by mutations in genes involved in the regulation of innate immune response with a consequent inflammatory phenotype. The most common genetically defined periodic fevers are Familial Mediterranean Fever (FMF), Cryopyrin-associated periodic syndromes (CAPS), TNF receptor-associated periodic syndrome (TRAPS) and mevalonate kinase deficiency/hyperimmunoglobulin D syndrome (MKD/HIDS). Some patients show clinical features similar to autoinflammatory diseases but no genetic mutation has been found.

**Objectives:** Our aim is to evaluate the differences in the expression of proteins or pathway in monocytes, and plasma metabolites in patients with autoinflammatory diseases compared with healthy subjects to clusterize and better understand the mechanisms underlying different genetically defined disorders and try to characterize the genetically undefined pathologies.

**Methods:** Monocytes, purified from peripheral blood and incubated for 4 hours with or without LPS, were collected from 5 patients for each pathology (FMF, CAPS, TRAPS and MKD) and healthy donors. The samples have been processed by iST protocol. Each digested sample was analyzed by high-resolution liquid chromatography and tandem mass spectrometry (LC-MS/MS) based on Orbitrap technology. The quantification strategy is a label-free approach (LFQ) available in MaxQuant suite.

**Results:** Here we identified a median of about 5000 proteins from the monocyte samples of each 4000 is quantified by LFQ approach. PCA analysis and Person’s correlation show good reproducibility of data and a good separation between the different groups. The data were then submitted to an appropriate statistic. The T-Tests highlighted the differentially expressed proteins and through the use of Cytoscape with the ClueGo app we obtained the differently regulated pathways in the different conditions. It has also been constructed, starting from significative proteins, a network, related to disease using the information of String Disease db. This was done to highlight the proteins associated with the disease as well as to reveal new possible biomarkers. We observed that the expression of proteins is differently enriched according to the different conditions. For each autoinflammatory disease, a list of significantly modulated proteins was obtained: some of which are already known to be related to the disorders, while others have not yet been described. In FMF, MEFV, RhoA and some related proteins were significantly up-regulated together with genes linked to the interferon pathway. In TRAPS relevant proteins turn up related to the maintenance of Golgi and cellular trafficking. The bioinformatics analysis allows us to better understand the functional interaction between these monocytes proteins and map which are involved in the diseases. Proteins thus analyzed were then contextualized in dominant pathways for each pathology through Cytoescape network analysis.

**Conclusion:** Here, we addressed how a high-resolution proteomics approach could be used to better understand the biology of autoinflammatory diseases. The characterization of a broad spectrum of proteins and their interaction network will allow us to identify new biomarkers for the different pathologies and better comprehend and recognize the genetically undefined disorders.

**Disclosure of Interest**

None Declared

#### PT1B02 Prulipotent stem cell derived myeloid cell lines for dissecting the mechanism of autoinflammation

##### Megumu Saito

###### Center for iPS Cell Research and Application, Kyoto University, Kyoto, Japan

**Introduction:** Autoinflammatory disorders (AIDs) is defined as an disorders associated with dysregulation of innate immune systems. In typical case, patients with AIDs show various inflammatory symptoms, such as periodic fever, skin rash and sterile serositis. Human induced pluripotent stem cell (iPSC) models of AIDs are supposed to be feasible and useful, because 1) most of typical AIDs are monogenic, 2) responsible cells are usually innate immune cells which can be robustly differentiated from PSCs, and 3) improvement of therapeutic or diagnostic approach is still needed in many AIDs. We therefore focused on establishing PSC models of AIDs, and applied these models to identification of genetic and biological background of patients’ pathophysiology.

**Results:** We established iPSCs from patients with AIDs such as CINCA/NOMID, Nakajo-Nishimura syndrome (NNS), and Blau syndrome (BS). We also corrected the disease-causing mutations by genome editing technology in selected clones. iPSCs were then differentiated into hematopoietic cells, especially into monocytic lineage cells. To stably obtain functional monocytic cells, monocytic progenitor cells derived from iPSCs were immortalized. The immortalized monocytic cell lines (MLs) were further differentiated into mature macrophages and then used for functional assays. The monocytic cells from AID-iPSCs showed increased secretion of proinflammatory cytokines such as IL-6, TNF and IL-1β. In case of CINCA/NOMID, iPSCs established from a NLRP3-mutation negative patient were useful for the identification of somatic NLRC4 mutation of the patient. The AID-iPSCs were also used for dissecting underlying disease mechanism, and suitable for high-throughput phenotypic screening.

**Conclusion:** We applied iPSC technology for studying autonflammatory disorders. When combined with other novel technologies such as next generation sequencing and genome editing, iPSC-based phenotypic dissection was useful for diagnosis of the patients and understanding the disease mechanism.

**Disclosure of Interest**

None Declared

#### PT1B03 Loss of protein prenylation in human monocytes promotes the formation of an NLRP3-dependent inflammasome in a model of mevalonate kinase deficiency

##### Oliver Skinner^1^, Julie Jurczyluk^1^,Paul Baker^2^, Seth Masters^2^, Avril Robertson^3^, Kate Schroder^4^, Sam Mehr^5^, Marcia Munoz^1^, Michael Rogers^1^

###### ^1^Bone Biology Division, Garvan Institute of Medical Research, Darlinghurst, Sydney; ^2^Inflammation Division, Walter and Eliza Hall Institute of Medical Research, Melbourne; ^3^School of Chemistry and Molecular Biosciences; ^4^Institute of Molecular Bioscience, University of Queensland, Brisbane; ^5^Dept of Allergy/Immunology, Royal Children's Hospital, Melbourne, Australia

####### **Correspondence:** Oliver Skinner

**Introduction:** Mevalonate kinase deficiency (MKD) is an autoinflammatory disease caused by mutations in an enzyme of the mevalonate pathway, leading to loss of isoprenoid lipids necessary for protein prenylation. Defective prenylation in MKD is thought to trigger inflammasome activation, IL-1β release and recurrent episodes of systemic inflammation. However, how loss of prenylation causes inflammasome activation remains controversial. Recent studies have suggested that lack of Rho or K-Ras prenylation leads to assembly of the Pyrin inflammasome in macrophages, whereas others have shown that disruption of the mevalonate pathway in monocytes triggers IL-1β release via NLRP3 inflammasome activation.

**Objectives:** To determine which type of inflammasome is activated in human monocytes upon loss of protein prenylation in MKD.

**Methods:** THP-1 human monocytic cells were treated for 24 hours with 5μM simvastatin (SIM) to pharmacologically block the mevalonate pathway, followed by stimulation with LPS or Pam3CSK4 (TLR4 and TLR2 agonists, respectively) to induce an inflammatory response. IL-1β and IL-18 release was quantified by ELISA whilst caspase-1 enzyme activity, ASC immunostaining and permeability to propidium iodide were used as measures of inflammasome formation and pyroptosis. To assess the contribution of Pyrin or NLRP3, we used inducible CRISPR/Cas9 knockout THP-1 cells, as well as the small molecule inhibitor of NLRP3, MCC950. Finally, inflammasome activation was examined in peripheral blood mononuclear cells (PBMCs) from an MKD patient (bearing V377I/H20N mutations in *MVK*) and heterozygous parents.

**Results:** SIM treatment of THP-1 cells caused a clear accumulation of unprenylated Rab and Rap1A proteins, without affecting cell viability. Furthermore, LPS or Pam3CSK4 stimulation of SIM-treated cells resulted in a 4-fold increase in IL-1β and IL-18 release and significantly higher ASC-containing speck formation, caspase-1 activity and pyroptosis. Importantly, all these effects were reversed to normal levels by restoring protein prenylation using geranylgeraniol (the missing lipid metabolite necessary for protein prenylation). In support of a predominant role for NLRP3, MCC950 completely inhibited the stimulatory effect of SIM on LPS-induced ASC speck formation, caspase-1 activation, IL-1β release and pyroptosis. All of these were also abolished by knocking out NLRP3, whereas deletion of Pyrin had no effect. Similarly, LPS stimulation of MKD patient-derived PBMCs resulted in much higher IL-1β release compared to heterozygous parents, which was completely blocked by NLRP3 inhibition with MCC950.

**Conclusion:** We clearly demonstrate that lack of prenylation in human monocytic cells, using statin treatment (a pharmacologic model of MKD) or using authentic cells from an MKD patient, leads to enhanced formation of an NLRP3-dependent, Pyrin-independent, inflammasome upon TLR2/4 stimulation. In contrast to reports that lack of prenylation activates the Pyrin inflammasome in macrophages, these findings indicate a prominent additional role for NLRP3 in the pathogenesis of MKD and demonstrate that targeting the Pyrin inflammasome in isolation may not be sufficient to resolve all the pathology associated with MKD. Rather, approaches to overcome the metabolic defect in the mevalonate pathway and restore normal protein prenylation could be more effective at preventing broader inflammasome activation.

**Disclosure of Interest**

None Declared

#### PT1B04 Performance of targeted NGS for routine diagnosis of autoinflammatory diseases

##### Guilaine Boursier^1^, Cécile Rittore^1^, Déborah Méchin^2^, Muriel Gutierrez^1^, Florian Milhavet^1^, Guillaume Sarrabay^2^, Isabelle Touitou^2^

###### ^1^Department of Medical Genetics, Rare diseases and personalized medicine, Rare and autoinflammatory diseases unit, CHU Montpellier, Univ Montpellier; ^2^IRMB, INSERM, Univ Montpellier, Department of Medical Genetics, Rare diseases and personalized medicine, Rare and autoinflammatory diseases unit, CHU Montpellier, Montpellier, France

####### **Correspondence:** Guilaine Boursier

**Introduction:** Monogenic systemic autoinflammatory diseases (SAIDs) are characterized by mutations in genes coding for proteins involved in innate immunity. Since the discovery of the first gene *MEFV* (OMIM 608107) responsible for familial Mediterranean fever, more than 30 new conditions have been identified, notably through high-throughput sequencing approaches. Currently, next generation sequencing (NGS) allows the simultaneous investigation of multiple genes at a manageable cost. We present here our 4 years of experience of targeted NGS as a routine diagnostic for SAIDs.

**Objectives:** To evaluate the performance of a panel of 49 genes targeting well-defined autoinflammatory diseases and clinical concordance after retro-phenotyping.

**Methods:** DNAs from 577 patients clinically suspected for SAIDs (age 3 months to 79 years) were sequenced by NGS between September 2014 and December 2018. The libraries were prepared using Nextera (Illumina) or SureSelect (Agilent) Target Enrichment Capture custom kits. Sequencing reactions were performed on MiSeq or NextSeq500 equipment (Illumina). The mutations were classified according to a 5-class scale provided by the Infevers database or according to the American College of Medical Genetics and Genomics (ACMG) guidelines. Epidemiological data, clinical symptoms and biological markers were collected on a form provided with all genetic diagnosis requests.

**Results:** Almost a half of patients (261/577) had at least one mutation. Mutations that were probably pathogenic (class 4) or clearly pathogenic (class 5) accounted for one third of the mutations and were detected in only 30/49 genes. We identified 87% (100/115) missense variants and 11% (13/115) truncating mutations including whole gene deletions. The genes in which we identified most of the pathogenic mutations were *MEFV, MVK, PSMB8, ADA2, NLRP3* and *RNASEH2B* then *NOD2, PSTPIP1* and *SLC29A3*. The most recurrent pathogenic variants were *MVK*:p.(Val377Ile), *MEFV*:p.(Met694Val) and *RNASEH2B*:p.(Ala177Thr). The yield of conclusive genetic diagnosis (one class 4 or 5 mutation including mosaicism in a dominant condition or two non-allelic mutations in a recessive condition) using this NGS strategy was 8% over this 4-year period. We observed a rather good, though incomplete clinical concordance in patients with a genetic diagnosis.

**Conclusion:** The simultaneous investigation of multiple genes using targeted NGS is a successful routine diagnostic for SAIDs and is of particular interest for the detection of low-level mosaic mutations and copy number variations. However, our targeted NGS approach has resulted in genetic confirmation in a relatively small proportion of patients. One of the reasons may be related to the stricter definition we have used compared to previous reports. On the other hand, SAID genes or molecular mechanisms that are still unknown are likely to be found. Finally, a better clinical filter for ordering genetic tests through a consultation with experts could be encouraged.

**Disclosure of Interest**

None Declared

#### PT1B05 Late-onset TRAPS with low-grade mosaicism in TNFRSF1A

##### Barend P. Kant^1^, Marco J. Koudijs^1^, Ruben van‘t Slot^2^, Joyce van Kuik^3^, Lisanne M. Sikkema^1^, Joost Frenkel^4^, Anna Simon^5^, Mariëlle E. van Gijn^1^

###### ^1^Department of Genetics; ^2^Center for Molecular Medicine; ^3^Department of Pathology; ^4^Department of Pediatrics, University Medical Center Utrecht, Utrecht; ^5^Department of Internal Medicine, Radboudumc Expertisecenter for Immunodeficiency and Autoinflammation, Radboud University Medical Center, Nijmegen, Netherlands

####### **Correspondence:** Barend P. Kant

**Introduction:** The diagnosis of patients with systemic autoinflammatory diseases (SAID) is difficult which can result in delayed treatment and irreversible organ damage. In several patients, low grade mosaicism of an autosomal dominant form of SAID has been detected. In NLRP3, even mosaic mutations with allele frequency <5% in whole blood can result in severe disease. In recent years, high-grade mosaic mutations have been detected in the TNFRSF1A gene in patients with late-onset TRAPS. With a new screening assay, we investigate whether low-grade mosaic mutations in TNFRSF1A might contribute to autoinflammatory disease.

**Objectives:** To examine the presence of low-grade mosaicism in a patient with clinically suspected late-onset TRAPS.

**Methods:** DNA was extracted from whole blood, FACS sorted hematopoietic cells and non hematopoietic cells. Mosaic mutation screening was performed using a single molecule molecular inversion probe (smMIP) assay. Positive results were confirmed with droplet digital (dd)PCR technology.

**Results:** Our patient is a 70 year old male with a history of episodes of fever with migratory erythematous rash and myalgia since his 30s. Also pleuritis, cervical lymphadenopathy and eye involvement were recorded and there was a single episode with abdominal pain and vomiting. On average, the episodes lasted for 2 weeks and occurred every 5 weeks. Family history was negative. Complete remission of the symptoms was achieved after starting treatment with anakinra.

We previously screened the TNFRSF1A gene by Sanger sequencing and next generation sequencing (NGS) with negative results. With our smMIP assay, we detected a c.269C>A p.(Thr90Asn) likely pathogenic missense mutation with 1,3% allele frequency in whole blood. This result was confirmed by ddPCR. The mutation was also present in sorted granulocytes, monocytes, T and B cells, but not in non-hematopoietic cells. This distribution is different from late-onset CAPS patients who were found to have myeloid restricted mosaic mutations in NLRP3.

**Conclusion:** We report the first low-grade mosaic variant in TNFRSF1A. Based on clinical symptoms, favorable response to anakinra and genetic findings, we diagnosed our patient with mosaic TRAPS. Our data suggest that even mutations present in a very small number of cells can cause systemic autoinflammatory disease.

**Consent for publication has been obtained from patient**

Yes

**Disclosure of Interest**

None Declared

#### PT1B06 Heterozygous TNFAIP3 mutation as the cause of an interferon-mediated neuroinflammatory disorder

##### Ciara M. Mulhern^1^, Ying Hong^1^, Ebun Omoyinmi^1^, Dara McCreary^1^, Marina Casimir^1^, Cheryl Hemingway^2^, Felice D’Arco^2^, Paul Brogan^1^, Despina Eleftheriou^1^

###### ^1^Infection, Inflammation and Rheumatology, Great Ormond Street Institute of Child Health; ^2^Neurology Department, Great Ormond Street Hospital for Children, NHS Foundation Trust, London, United Kingdom

####### **Correspondence:** Ciara M. Mulhern

**Introduction:** Heterozygous loss-of-function mutations in *TNFAIP3* lead to haploinsufficiency of A20 (HA20) commonly manifesting with severe orogenital ulceration and ocular inflammation. Central nervous system (CNS) inflammation has been observed in patients with haploinsufficiency of HA20 but CNS involvement as the sole clinical manifestation of heterozygous TNFAIP3 mutation in humans has never been described however. Herein, we report on the case of an 8 year old female of non-consanguineous Pakistani-Indian descent who presented with left sided focal seizures and hemiparesis, acute uveitis and congitive decline. Magnetic resonance imaging (MRI) of her brain revealed contrast-enhancing T2 hypointense intracranial mass lesions, in the grey matter of the paracentral lobule and the thalamus on the left with surrounding oedema. Brain biopsy revealed necrotising granulomatous inflammation. Brain CT revealed intracerebral calcification. Extensive screening of infectious or malignant causes was negative.She failed to respond to multiple anti-inflammatory treatments but had an excellent radiological and clinical response to an oral JAK-STAT inhibitor suggesting this was likely an interferon (IFN) mediated inflammatory disease.

**Objectives:** To use next generation sequencing to identify the genetic cause of the progressive neuroinflammatory disorder in this case and characterise the mechanisms underpinning neuroinflammation

**Methods:** Whole exome sequencing (WES; illumina MiSeq) was carried out in all family members. Expression of phosphorylated- p65, IRF3, STAT1 and STAT3 in both patient and healthy control derived peripheral blood mononuclear cells was assessed by flow cytometry. Meso Scale Discovery (MSD) assays was used to quantify cytokines in patient serum. qPCR assays measured the expression levels of IFN stimulated gene expression. Co-immunoprecipitation analysis assessed the binding capacity of the mutated protein to Tank-binding kinase 1 (TBK1)

**Results:** WES revealed a heterozygous missense p.T647P mutation in *TNFAIP3* as the cause of the progressive neuroinflammatory disorder in this case. Patient-derived cells exhibited enhanced phosphorylation of the p65 transcription factor, and increased expression of NF-ƙB mediated proinflammatory cytokines. The mutated p.T647P A20 protein failed to control interferon-regulatory-factor-3 (IRF3) activation and interferon (IFN)-dependent gene transcription, in comparison to healthy control cells. We also show that the mutant A20 protein cannot efficiently bind to TBK1, in order to turn off IRF3 activation leading to an enhanced IFN signature in the patient. We believe that failure to regulate IFN-mediated immune responses, was the driver of the CNS inflammation observed. Furthermore, treatment with an oral JAK 1/2 inhibitor resulted in marked clinical improvement, complete radiological resolution of neuroinflammation, and normalisation of IFN-stimulated gene expression in whole blood. IFN mediated immune responses were also impaired in an additional disease control case of HA20 in a 4 year old heterozygote for the p.N98Tfs25 *TNFAIP3* variant.

**Conclusion:** We describe for the first time heterozygous p.T647P missense mutation in *TNFAIP3* as the cause ofprogressive neuroinflammation. The p.T647P mutated A20 proteinfailed to control IRF3 activation and IFN dependent transcription. Treatment with an oral JAK 1/2 inhibitor was highly effective. Our report now adds *TNFAIP3* mediated neuroinflammation to the ever-expanding group of monogenic interferonopathies with propensity to CNS involvement.

**Consent for publication has been obtained from patient**

Yes

**Disclosure of Interest**

None Declared

#### PT1B07 Systemic evaluation of genetic and biochemical testing for deficiency of ADA2 (DADA2) from the NIH patient cohort

##### Natalia Sampaio Moura, Oskar Schnappauf, Natalie Deuitch, Qing Zhou, Daniel Kastner, Ivona Aksentijevich

###### Inflammatory Disease Section, National Institutes of Health, Bethesda, United States

####### **Correspondence:** Natalia Sampaio Moura

**Introduction:** Deficiency of adenosine deaminase 2 (DADA2) is an autosomal recessive autoinflammatory disorder caused by loss-of-function mutations in the *ADA2* gene, which encodes the adenosine deaminase 2 (ADA2) enzyme. Patients with DADA2 can present with many different manifestations including early-onset lacunar stroke, recurrent fever, hepatosplenomegaly, livedo reticularis, immune dysregulation, and hematopoietic abnormalities. They also exhibit absent or significantly reduced ADA2 enzyme activity. Despite these conserved features, some individuals display discordance between clinical characteristics and genetic or enzyme activity testing.

**Objectives:** The goal of our retrospective study is to evaluate sensitivity of diagnostic testing for DADA2 in our CLIA-certified laboratory at the National Institutes of Health (NIH). We compared the diagnostic efficacy of the different types of assays, such as Sanger sequencing, ADA2 enzymatic analysis and multiplex ligation-dependent probe amplification (MLPA).

**Methods:** We reviewed all *ADA2* Sanger sequencing results of individuals tested between 2014 and 2018, including confirmatory testing in referral patients, and determined the diagnostic yield of this method in patients we suspected to have DADA2 based on clinical phenotype. We then analyzed if subsequent ADA2 enzyme assay and/or MLPA further increased our diagnostic yield.

**Results:** Out of 190 individuals with clinical characteristics suggestive of DADA2, 56 patients tested positive for biallelic mutations (29%). Eleven tested as carriers for a monoallelic pathogenic mutation (6%), and 123 (65%) were mutation-negative based on conventional Sanger sequencing. ADA2 enzyme activity assay was performed on 45 patients who had available serum samples. The assay corroborated the DADA2 diagnosis on 30 patients and identified 7 individuals with monoallelic mutations (detected via Sanger) from three different families as patients due to low enzyme activity. MLPA successfully identified a second pathogenic copy number variation (CNV) in all patients who displayed low ADA2 activity. Addition of ADA2 protein assay and MLPA increased our DADA2 diagnostic rate to 33%. Thus, we observed a 13.8% (4/29) relative increase in our diagnostic yield due to the incorporation of these testing modalities.

**Conclusion:** Our results suggest that the serum ADA2 enzyme assay should be considered first tier testing for patients with suspected DADA2, given that pathogenic mutations in *ADA2* are highly heterogeneous and not always detectable by Sanger sequencing alone. Sanger sequencing in combination with MLPA remain indispensable assays to support and confirm enzymatic testing when indeterminate or unclear, and to identify causal variants. Detection of mutations at the DNA level is important for genetic counseling. Targeted next-generation testing is another method able to identify a diverse scope of pathogenic mutations and has the potential to increase the diagnostic yield of genetic testing.

**Disclosure of Interest**

None Declared

### Monogenic autoinflammatory diseases (clinical)

#### P1001 Is there any difference between M694V heterozygote and non-exon 10 mutations on symptoms onset and response to colchicine treatment?

##### Hatice Adiguzel Dundar^1^, Serkan Turkucar^1^, Ceyhun Acari^1^, Ozge Altug Gucenmez^2^, Balahan Makay^2^, Erbil Unsal^1^

###### ^1^Department of Pediatrics, Pediatric Rheumatology Unit, Dokuz Eylul University Faculty of Medicine; ^2^Pediatric Rheumatology Unit, Dr. Behcet Uz Childrens’ Hospital, Izmir, Turkey

####### **Correspondence:** Hatice Adiguzel Dundar

**Introduction:** Familial Mediterranean fever (FMF) is the most common inherited autoinflammatory syndrome throughout the world. It is caused by mutations of the MEFV gene encoding a protein called pyrin. The most frequent genotype-phenotype correlation is in a certain part of exon 10, especially M694V mutation. There are also a group of patients with non-exon 10 mutations, who have a similar clinical spectrum of the disease.

**Objectives:** We aim to investigate the genotype-phenotype differences between M694V heterozygote mutations and non-exon 10 mutations.

**Methods:** Data charts of children (n=431) with FMF from Dokuz Eylul University childrens’ hospital and Dr.B.Uz childrens’ hospital were reviewed. Patients were divided into two groups with regard to having M694V heterozygote or non-exon 10 mutations. Genotype-phenotype features and response to treatment were compared.

**Results:** There were M694V heterozygote mutations in 128 (29.7%) patients and non-exon 10 mutations in 303 (70.3%) patients. The follow-up period was 54.5 (33-105) months. There was no difference between the age of symptoms onset, the age of diagnosis, and the diagnosis delay time. The family history in patients with M694V heterozygote mutation was statistically positive compared to non-exon 10 mutation group (p:0.000). The symptoms of joint involvement as arthritis were significantly higher in the M694V heterozygotegroup (p:0.026). Additionally, biological agent need due to colchicine unresponsiveness was statistically higher in M694V heterozygote group than group with non-exon 10 mutation (p:0.004) (Table 1).

**Conclusion:** There is a significant difference between children with M694V and non-exon 10 mutations, even when the M694V mutation is present in one allele only. Family history with FMF, musculoskeletal symptoms, and unresponsiveness to colchicine are main parameters.

**Disclosure of Interest**

None Declared

#### P1002 A case report of aicardi goutieres syndrome type 5 in two siblings mimicking juvenile idiopathic arthritis

##### Buthaina Al Adba^1^, Hajar Dauleh^2^

###### ^1^Paediatric Rheumatology; ^2^Paediatric, Sidra Medicine, Doha, Qatar

####### **Correspondence:** Buthaina Al Adba

**Introduction:** Aicardi-Goutières syndrome (AGS) is a genetically determined encephalopathy characterized by calcification of the basal ganglia and white matter, demyelination, and raised levels of lymphocytes and IFNα in the cerebrospinal fluid [1]. Neurological dysfunction becomes clinically apparent in infancy and manifests as progressive microcephaly, spasticity, dystonia, and psychomotor retardation. Expression of interferon-regulated genes (IGS) in peripheral blood is also upregulated, which is sustained over time [2]. The genes mutated in AGS have been defined to encode proteins implicated in the metabolism of nucleic acids TREX1, the RNASEH2 complex and SAMHD1,which may all function as cellular nucleases [3].

**Objectives:** Arthritis and progressive arthropathy with distal joint contractureshave been reported in SAMHD1 mutation along with neurological symptoms [4]. We are describing two siblings with SAMHD1 mutation who only presented with early onset polyarthritis and no systemic or CNS manifestations.

**Methods:** Chart review of clinical data includes IFN signature and molecular analysis.

**Results:** Two brothers of consanguineous parents with no significant family history of auto inflammatory or autoimmune disease, were born healthy, at term with normal growth parameters.Patient one is a six yearold boy who presented at age of four with one-year history of joint pain and morning stiffness. He had active arthritis in his both wrists, knees and elbows. Lab testing revealed normal blood work ESR, CRP, and negative ANA and RF. Interestingly HLAB27 was present.A diagnosis of JIA was made and he was started on naproxen, and had intra-articular in jectionsarticular injections twice. His arthritisHis arthritis was difficult to control and Etanercept andsubcutaneous Methotrexate were started. His arthritis improved significantly,with residual mild disease atboth wrist joints.His brother, patient two is a 3 year old boy who presented similarly at age of 18 months with morning stiffness and abnormal gait for one-month duration. He had active arthritis in both knees, hips and wrists. He had similar lab findings and positive HLAB27.He was treated with naproxen, a short course of oral steroid and Methotrexate. He responded faster than his brother, but continue to have mild arthritis atboth wrists. Due to difficult to treat, early arthritis in two consanguineous brothers, wesent genetic testing with whole exome sequencing (WES) to look for other causes of their arthritis.Genetic testing for both siblings showed Homozygous mutation at the SAMHD1 gene for the P.Arg 290His (CGT>CAT):c.869G >A.Both parents were heterozygous for the same mutation. The R290H variant in the SAMHD1 gene has been reported previously in association with AGS in an affected individual with multiple clinical features including CNS symptoms and arthritis [5].Peripheral blood analysis of type I interferon-related biomarkers demonstrated that both siblings have an interferon signature characteristic of Aicardi-Goutieres syndrome (Fig 1). Both brothers continued to have normal development with no CNS symptoms or rash and their eye exam showed no glaucoma, which was previously reported in AGS with the SAMHD1 mutation. Despite being asymptomatic from CNS disease we are considering brain MRI to look for inflammatory cerebral vasculopathy that has been reported previously [6].

**Conclusion:** We are reporting a spectrum of AGS with a SAMHD1 mutation that mimicks difficult to treat early onset polyarticular arthritis without any CNS or skin manifestations. This illustrates the need to consider mutation analysis of SAMHD1 in similar presentations as CNS and skin disease may evolve later. Finally, even though there has been partial response to treatments,we may consider changing therapy to Janus Kinase inhibitors to achieve complete remission of arthritis.

**Consent for publication has been obtained from patient**

Yes

**Disclosure of Interest**

None Declared

#### P1003 Phenotypic and genotypic characteristics and damage accrual of monogenic autoinflammatory diseases other than familial Mediterranean fever from the pediatric rheumatology Arab group (PRAG)

##### Sulaiman Al-Mayouf^1^, Abdulaziz Almutairi^1^, Safia Albrawi^2^, Abdulatif AlEnazi^3^, Basil Fatallah^4^, Abdulallh Alsonbul^1^, Mohammed Abu-shukair^5^, Raed Alzyoud^5^, Adel Alwahadneh^5^, Mabruka Zlenti^6^, Ebtisam Kawaja^6^, Khloud Khawaja^7^, Zakia Almusawi^8^, Wafa Madan^8^, Muna AlMutairi^9^, Nora Almuatiri^10^ and Pediatric Rheumatology Arab Group (PRAG)

###### ^1^King Faisal Specialist Hospital and Research Center, Riyadh, Saudi Arabia; ^2^Royal Hospital, Muscat, Oman; ^3^King Fahad Medical City, Riyadh, Saudi Arabia; ^4^AlJalial Children Hospital, Dubai, United Arab Emirates; ^5^Queen Rania Children Hospital, Amman, Jordan; ^6^Tripoli Children Hospital, Tripoli, Libya; ^7^Mafraq Hospital, Abu Dhabi, United Arab Emirates; ^8^Salmaniya Hospital, Bahrain, Bahrain; ^9^Al Adan Hospital; ^10^AlSabah Hospital, Kuwait, Kuwait

####### **Correspondence:** Sulaiman Al-Mayouf

**Introduction:** Monogenic autoinflammatory diseases (AIDs) are a group of rare hereditary recurrent multisystem inflammatory diseases.The available published data from Arab countries about monogenic AIDs other tahn Familial Mediterranean fever (FMF) is very limited.

**Objectives:** To report the phenotype, genotype and response to treatment in Arab children with monogenic AIDs other than FMF, with focus on accrual damage.

**Methods:** We retrospectively reviewed patients with clinical and/ or genetically proven monogenic AIDs other than FMF seen between 1990 and 2018 at 10 rheumatology clinics from seven Arab countries. Data were collected at the last follow-up visit comprising history of consanguinity, age at onset and diagnosis, follow-up duration, clinical and laboratory findings, as well as the damage accrual and death related to monogenic AIDs.

**Results:** Seventy (46 female) patients with monogenic AIDs were analyzed. Consanguinity rate among the enrolled patients was 74.6% and a family history of AIDs was present in 60%. The mean age at disease onset was 3.2±2 years and mean duration of follow-up was 7.5±4 years. The initial diagnosis was inaccurate in 47% and the mean diagnosis delay was 4±3 years. The most frequent monogenic AIDs were LACC1 associated monogenic disorders (monogenic JIA and monogenic Crohn’s) (23) followed by cryopyrin-associated periodic syndrome (CAPS) (12), tumor necrosis factor receptor associated periodic syndrome (TRAPS) (12), hyperimmunoglobulinemia D syndrome (HIDS) (9), Majeed’s syndrome (6) and eight patients with other monogenic AIDs. Musculoskeletal involvement was the main feature in LACC1 associated monogenic disorders while fever and mucocutaneous and gastrointestinal involvement were the most prevalent features in the other monogenic AIDs. Genetic testing was performed in 67 patients, 69% had genetically confirmed disease. Patients with mutation c.T850C p.C284R in exon 4 of *LACC1* had severe arthritic changes. Three CAPS patients with NLRP3 mutation had cognitive impairment and one with significant hearing and ocular damage. Two HIDS patients had homozygous p.V3771 mutation and other two patients with p.V3771/compound heterozygous MEFV: p.E148Q/p.P369S/p.R408G. Three different LPIN2 mutations were recorded for Majeed’s syndrome. Overall, growth failure was the most frequent (36%), followed by cognitive impairment (13%). There were three deaths due to infection.

**Conclusion:** The number of genetically confirmed patients with monogenic AIDs other than FMF are not uncommon among Arab children probably due to a high consanguinity rate. Diagnostic delay and high damage accrual emphasize the need for more awareness and early referral to specialized centers.

**Disclosure of Interest**

None Declared

#### P1004 Auto-inflammatory diseases: a retrospective single center study in Saudi Arabia

##### Abdulrahman A. Alrasheed^1^, Ashwag Al Harthi^2^, Banan Al Rewaithi^2^, Wafa Suwairi^2^, Jubran Al Qanatish^2^, Fayhan Al Roqi^2^

###### ^1^King Abdullah Specialized Children Hospital; ^2^Pediatric, King Abdullah Specialized Children Hospital, Riyadh, Saudi Arabia

####### **Correspondence:** Abdulrahman A. Alrasheed

**Introduction:** The auto-inflammatory conditions represent an emerging and over-expanding series of diseases unified by a dysregulation of innate immunity. The current increase in the prevalence of chronic inflammatory diseases makes this subject of interest. Early recognition and treatment with immunomodulator agents have the potential to improve the outcomes and the quality of life.

**Objectives:** The purpose of this review is to describe the clinical features, investigations, therapeutic modalities and outcome of auto-inflammatory syndromes seen at King Abdullah Specialized Children’s Hospital, Riyadh, Saudi Arabia.

**Methods:** We carried out a descriptive retrospective analysis of pediatric patients with auto-inflammatory diseases who were seen and managed at a single tertiary center, in Riyadh, Saudi Arabia, between 2004 and 2018. Multiple measures were investigated including time of disease onset, clinical features, investigations, treatment, and outcome.

**Results:** 67 patients (30 females and 37 males) with auto-inflammatory diseases were identified and formed the basis of the study (Table 1).

Autoinflammatory diseases include Systemic Juvenile Idiopathic Arthritis (sJIA) 46%, Chronic Recurrent Multifocal Osteomyelitis (CRMO) 15%, Familial Mediterranean fever (FMF) 7%, Hyperimmunoglobulin D syndrome (HIDS) 6%, Periodic fever, aphthous stomatitis, pharyngitis, cervical adenitis (PFAPA) 6%, Neonatal onset multisystemic inflammatory disease (NOMID) 3%, STING-associated vasculopathy with onset in infancy (SAVI) 3%, ), Aicardi-Goutières syndrome (AGS) 1%, Deficiency of Adenosine Deaminase 2 (DADA2) 5%, Deficiency of interleukin thirty-six receptor antagonist (DITRA) 1%, Blau syndrome 1% and Uncharacterized Auto-inflammatory Disease (UAD) 3%.

The mean age at initial presentation was 58.2 months. The median Erythrocyte Sedimentation Rate (ESR) and C - Reactive protein (CRP) were 72 mm/hr and 86 mg/dl, respectively. Treatment modalities that have been used include Disease-modifying antirheumatic drugs (DMARDs) and/ or biologic therapy including Anti TNF alpha, Anti IL-1 or Anti IL-6 and others (Table1).

**Conclusion:** We believe this cohort provides the common features of auto-inflammatory diseases in Saudi Children and the outcome after frequently used target therapies. Early recognition and treatment of auto-inflammatory diseases with immunomodulator agents have the great potential to alleviate the debilitating symptoms and to improve the quality of life.

**Disclosure of Interest**

None Declared

**Table 1 (abstract P1004).** See text for description

Disease	No. of patients	%	gene study	treatment and outcome
SoJIA	31	46%	Negative HLH gene done for patients with MAS	good response to Anakinra or Tocilizumab
CRMO	10	15%	Negative geneexcept 1 patient with CRMO and Familial hyperphosphatemic tumoral calcinosis who has homozygous mutation in GALNT3 gene	3 patients with mild CRMO responded to NSAIDs only. 2 patients required either pamidronate or Methotrexate. 4 patients required combination of Infliximab and Methotrexate. 1 patient who had unidentified auto- inflammatory disease responded partially to Abatacept but not to Anakinra, Adalimumab or Tocilizumab.
FMF	5	7%	Heterozygous mutation in MEFV gene (Met694Val)	Good response to Colchicine
PFAPA	5	8%	Negative periodic fever gene panel	4 patients received no treatments with less frequent attacks during follow up and one free of attacks after tonsillectomy
HIDS	4	6%	Mutation in MVK gene	3 patients treated with Anakinra showed good response 1 patient had infrequent attacks not on treatment
NOMID	2	3%	Negative gene	Good response to anti IL-1 therapy (Anakinra and Canakinumab)
Interferonopathies	2 SAVI 1 AGS	5%	Mutation in TMEM-173 gene (STING gene) for SAVI and RNASEH2A for AGS	1 SAVI patient treated with Ruxolitinib with significant improvement while the other died AGS treated with Ruxolitinib with good response
DADA2	3	5%	mutation in CECR1 gene	2 patients treated with adalimumab and GCSF with good response, and the 3rd patient recently diagnosed
DITRA	1	1%	mutation inIL36RN gene	Response was lost on etanercept, Anakinra and then Ustekinamab Currently on Adalimumab with no relapse so far
Blau syndrome + crohn’s disease	1	1%	mutations in NOD2/CARD15 gene	Treated with Azathioprine and steroid with partial response

#### P1005 Clinical and genetic characteristics of myalgia in Armenian children with familial Mediterranean fever

##### Gayane G. Amaryan^1^, Tamara F. Sarkisian^2^, Nune G. Mkrtchyan^3^, Marina M. Papazyan^4^, Artashes E. Tadevosyan^5^

###### ^1^National Pediatric Centre for Familial Mediterranean Fever of “Arabkir” Medical Centre - Institute of Child and Adolescent Health, Department of Pediatrics Yerevan State Medical University; ^2^Centre of Medical Genetics and Primary Health Care, Depatment of Medical Genetics Yerevan State Medical University; ^3^National Pediatric Centre for Familial Mediterranean Fever of “Arabkir” Medical Centre - Institute of Child and Adolescent Health, Department of Pediatrics Yerevan State Medical University; ^4^NationalPediatric Centre for Familial Mediterranean Fever, “Arabkir” Medical Centre - Institute of Child and Adolescent Health; ^5^Department of Public Health and Health Care Organization , Yerevan State Medical University, Yerevan, Armenia

####### **Correspondence:** Gayane G. Amaryan

**Introduction:** Familial Mediterranean Fever (FMF) as an ethnic disease is wide-spread in Armenia. In most cases FMF manifests in childhood. Myalgia is an essential feature of musculoskeletal symptoms (MSM) of FMF, which can be presented as acute muscle attacks - spontaneous myalgia (SM) and exercise-induced myalgia (EIM), as well as prolonged protracted febrile myalgia (PFM). Awareness of these symptoms are important for early diagnosis and differential diagnosis of FMF.

**Objectives:** to study the frequency of different types of myalgia in Armenian children with FMF and their correlation with *MEFV* genotype and disease severity.

**Methods:** A group of 715 children with FMF was observed at the National Pediatric Centre for FMF (438 boys, 277 girls, mean age 8.64±0.17). The diagnosis of FMF was based on the Tel-Hashomer criteria and detection of 12 *MEFV* mutations common for Armenians using Viennalab Diagnostics molecular-genetic assay. For statistical analysis standard statistical Epi-Info 2000 Program was performed. For comparison of two nominal variables in table “two by two” Yaet’s corrected for continuity chi-square test was used, significance level p<0.05

**Results:** Myalgia was observed in 37.5% (268 out of 715) of FMF patients and manifested mainly as SM and /or EIM in 34.7% (248 patients), as well as PFM in 2.7% (20 patients).

SM and EIM manifested as transient pains of the legs without fever, disappearing after resting or taking NSAIDs. The frequency of development of SM and EIM varied from 43.6% to 31.2% depending on the genotype and was relatively common in M694V and V726A heterozygotes compared to homozygotes(χ2= 3.41; p> 0.05). Risk of development of SM and EIM was associated with severity of FMF (χ2= 23.20, p <0.0001) and was higher in severe and moderate course of FMF compared to mild 5.6 times (χ2= 18.28; P <0.0001) and 1.9 times (χ2= 3.77; P <0.05) respectively.

We observed prolonged PFM in 2.7% of FMF patients with more severe phenotypes, which were characterized by 4 times higher risk of early onset (χ2= 5.94; p = 0.015) (mean age 3 years), as well as late diagnosis of FMF (9.42 ± 0.72) and the delayed start of colchicine therapy . This cohort of patients had also high frequency of severe FMF attacks with generalized febrile muscle pain during 1-3 weeks, prevalence of acute recurrent arthritis, erysipelas-like erythema. PFM manifestation usually was after 5-6 year of FMF onset. Because of PFM is considered also as vasculitis, steroid therapy was started along with colchicine. Development of PFM was associated with severe M694V-homozygous genotype (χ2 = 8.27; p <0.02) and was observed at 4.6% of M694V-homozygous patients.

**Conclusion:** The frequency of different types of myalgia in Armenian children with FMF is high (37.5%), especially acute muscle attacks - SM and/or EIM (34.7%). PFM is diagnosed in 2.7% patients.

The development of SM and EIM is associated with the severity of FMF and does not depend on the *MEFV* genotype. The risk of development of PFM, as a part of prolonged MSM, is associated with both: the *MEFV* genotype (mostly M694V- homozygous) and the severity of the disease.

This allows to consider PFM as a marker of severe course of FMF and early disease onset and the M694V homozygous genotype as a risk factor for the development of PFM.

*MEFV* mutation genetic screening is recommended for Armenian paediatric patients with different types of myalgia, especially with PFM, for early diagnosis of FMF, treatment and prevention of complications.

**Disclosure of Interest**

None Declared

#### P1006 The impact of aging on familial Mediterrinean fever patients

##### Okan Aydin^1^, Serdal Ugurlu^1^, Bugra Egeli^2^, Ece Soykut^2^, Deniz Demir^2^, Huri Ozdogan^1^

###### ^1^Division of Rheumatology, Department of Internal Medicine, Cerrahpasa Medical Faculty; ^2^University of Istanbul - Cerrahpasa, Istanbul, Turkey

####### **Correspondence:** Okan Aydin

**Introduction:** Familial Mediterranean Fever (FMF) is a monogenic autoinflammatory disorder with innate immune activation with an onset before age 20 in approximately 90% of the patients. There is scarce data on the effect of aging on FMF patients over 40 years of age

**Objectives:** This study aims to collect data on FMF patients who have survived over 40 years of age. Here we report our preliminary data on disease course and treatment status and comorbidities of our patients with FMF.

**Methods:** Among the FMF patients who have been followed in our FMF outpatient clinic with a pool of approximately 5000 patients, those who have aged 40 and over are being included to the study. As by today 180 patients are considered for evaluation. The files of patients were reviewed and a standard questionnaire was used to interview the patients. Here we report the results of 100 of these patients (56%) who were contacted for this purpose. These patients were questioned on their demographic characteristics, comorbid conditions, colchicine treatment details, and attack information. In order to see the trend of the change in the parameters assessed , the patients were divided into two groups based on their present age(Group 1: 40-50 years, Group 2: ≥50 years).

**Results:** A total of 100 (78 F, 22M) patients were evaluated. There were 61(46F, 15M) patients aged between 40-50 years and 39 (32F, 7M) over 50. The demographic characteristics and clinical features of these patients are given in Table 1.Besides 3, all patients were still on colchicine regularly. Ninety-six percent of the patients declared overall benefit from colchicine therapy, however 38% experienced a side effect related to this treatment.Over 88% of the patients reported decrease in severity and frequency of FMF attacks.The mean daily colchicine dose was lower in the age 50 and over group (1.7±0,77 mg versus 1,35 ±0,38 mg). There were no patients with AA amyloidosis in neither age group. The mean duration from the last attack increased from 15.3 ±19.7 months to 35.6± 52 months in the older patients. One or more additional disease was present in 75% of this patient group.Among the comorbidities hypertension was the most frequent, diagnosed in 25% of the patients, followed by hypothyroidism (16%), diabetes mellitus (10%) and cardiac disease (5% ). Sixty-five of the patients were recieving other medications in addition to colchicine.

**Conclusion:** According to our preliminary data the majority of the patients continue to take colchicine after age of 40. However the frequency of FMF attacks as well as daily colchicine dose decrease as the patients get older.With well designed trials stopping colchicine treatment may be considered in a subgroup of patients after 50 years of age. Approximately ¾ of the FMF population over 40 years of age has a comorbidity that neccecitates additional medications which underlines the need for special attention.

**Disclosure of Interest**

None Declared

**Table 1 (abstract P1006).** Clinical course and co-morbidities in two age groups over 40 years

n	Group 1* (n=61)	Group 2** (n=39)	p
Sex (F:M); current age (mean±SD) (yr)	(46 :15) ;45.5 ± 2.29	(32:7); 57.05 ± 6.81	0,43;<0.001
Mean duration since the last episode, (mean±SD, mo)	**15.3 ± 19.7 (1-60)**	**35.67 ± 52.05 (1-276)**	**0,012**
Number of patients on colchicine therapy, n (%)	59 (96.7)	38 (97.4)	NS
Mean colchicine dose, mg/day (current)	**1.7±0.76**	**1.41±0.45**	**0.03**
Number of patientswith decrease in attack severity, n (%)	54(88,5)	35 (89,7)	NS
Number of patients with decrease in attackfrequency, n (%)	57(93,4)	37 (94,8)	NS
Co-morbidities, n (%)			
Hypertension, n (%)	12(19.6)	13 (33.33)	NS
Hypotyroidism, n (%)	12(19,6)	4(10,2)	NS
Type 2 Diabetes Mellitus, n (%)	5(8.2)	5(12.82)	NS
Rheumatological diseases, n (%)	5(8,2)	3(7,7)	NS
Cardiac disease, n (%)	3(4,9)	2(5,1)	NS
Malignancies, n (%)	2(3,2)	2(5.13)	NS
Additionalmedications (number of patients, %)	36 (61)	29 (74.3)	NS

#### P1007 Comorbidities in familial Mediterranean fever

##### Ummusen Kaya Akca^1^, Banu Balci Peynircioglu^2^, Zehra S. Arici^1^, Edibe Avci^2^, Zulfiye Y. Akkaya Ulum^2^, Engin Yilmaz^2^, Yelda Bilginer^1^, Seza Ozen^1^

###### ^1^Pediatric Rheumatology; ^2^Medical Biology, Hacettepe University, Ankara, Turkey

####### **Correspondence:** Banu Balci Peynircioglu

**Introduction:** Familial Mediterranean Fever (FMF) is a periodic fever syndrome, characterized by recurrent episodes of fever and serosal inflammation accompanied with high acute phase reactants.The analysis of possible comorbidities is important to understand the impact of these conditions on clinical care and whether they share a common etiological pathway.

**Objectives:** We aimed to evaluate the comorbidities associated with FMF patients in a large genetically diagnosed cohort.

**Methods:** We retrospectively evaluated the medical records of FMF patients who were followed up at Department of Pediatric Rheumatology in Hacettepe University between 2000 and 2015. This study was approved by the Research Ethics Committee and was conducted in accordance with the Declaration of Helsinki. The diagnosis of FMF was made according to Tel Hashomer diagnosis criteria for patients who applied prior to April 2009 and to the Turkish FMF pediatric diagnosis criteria after April 2009. The FMF patients who had homozygous or compound heterozygous mutations were included in the study. Comorbidities associated with FMF were divided into three groups; associated with increased inflammation, associated with FMF and incidental.

**Results:** A total of 1999 patients were enrolled in the study. Of all 1999 FMF patients, 636 were children (31.8 %), 1029 were males (51.4%), with a mean age of 31.60±16.01 years. The mean follow up time was 4.50±3.99 years (median:3.84 range from 0.21-29.4 years). 880 of 1999 (44%) FMF patients had homozygous MEFV gene mutation, the most common mutation was M694V homozygous. The remaining were compound heterozygous. 656 patients (32.8%) had one or more than one comorbidity associated with FMF. Ankylosing spondylitis was the most common comorbidity associated with increased inflammation while the most common comorbidity in FMF related comorbidities was renal amyloidosis. The frequency of ankylosing spondylitis, henoch schonlein purpura, juvenile idiopathic arthritis, polyarteritis nodosa (PAN), multiple sclerosis (MS) and Behçet’s disease were increased in patients with FMF when compared to those in the literature. Systemic lupus erythematosus was observed less frequently in the patients with FMF than in the population. While the increase in the frequency of MS was 3.3 times, the frequency of PAN was increased 110 times.

**Conclusion:** This study shows that FMF is a hereditary disease associated with significant comorbidity. We also confirm that inflamatory and rheumatic diseases are more common in FMF.

**Disclosure of Interest**

None Declared

#### P1008 Bone metabolism in systemic autoinflammatory diseases (SAIDS): a case- control study from Padova cohort

##### Sara Bindoli^1^, Giulio Franceschet^2^, Paola Galozzi^1^, Martina Zaninotto^3^, Valentina Camozzi^2^, Paolo Sfriso^1^

###### ^1^Rheumatology Unit, Department of Medicine; ^2^Endocrinology Unit, Department of Medicine; ^3^Department of Laboratory Medicine, University of Padova, Padova, Italy

####### **Correspondence:** Sara Bindoli

**Introduction:** Systemic autoinflammatory diseases (SAIDs) represent a group of disorders characterized by recurrent fever attacks, polyserositis, skin, musculo-skeletal and articular manifestations. A dysregulation of the innate immune response in a genetic predisposed host leads to the development of SAIDs. In our study we included patients affected by Familial Mediterranean Fever (FMF), TNF Receptor Associated Periodic Syndrome (TRAPS) and Hyper-IgD Syndrome (HIDS). It is assessed that chronic inflammation, perpetuated by pro-inflammatory cytokines, may exert a role on bone metabolism leading, in the long run, at the onset of osteoporosis (OP). However, how OP may occur in the context of autoinflammatory diseases remains partially unknown.

**Objectives:** The aims of our study are focused on the assessment of bone metabolism in patients affected by SAIDs compared to healthy subjects and on the relationship between bone turnover markers (Receptor activator of nuclear factor kappa-Βligand, RANKL and osteoprotegerin, OPG) and serum inflammation markers (serum amyloid A, SAA).

**Methods:** 40 adults patients referring to the Rheumatology Unit of Padova University Hospital affected by FMF, TRAPS and HIDS and 40 healthy subjects were recruited between March and June 2018. Fasting blood samples were collected in order to determinate calcium (Ca), phosphorus (P), magnesium (Mg), 24-h urine calcium, 24-h urine phosphorus, albumin, parathyroid hormone (PTH), Vitamin D, creatinine, serum amyloid A (SAA), c-terminal telopeptide of type I collagen (CTX), bone alkaline phosphatase (b-ALP). Moreover, serum OPG and RANK-L were determined by a commercially available ELISA kit (*Pantec, Turin, Italy)*. Femur and lumbar dual-energy X-ray absorptiometry (DXA) was performed with the QDR Bone Densitometer Discovery (*Hologic Inc.,Waltham, MA*). Trabecular Bone Score (TBS) was calculated on DXA lumbar images, using iNsight Software (*version 1.8.0.0; Medimaps, Merignac, France*). The statistical analysis was performed using Mann-Whitney U Test.

**Results:** We did not observe a statistically significant difference between bone mineral density (BMD) and TBS of patients compared to controls (p=0.5037 and p=0.8031). Also, the values of phospho-calcic metabolism samples were not statistically different between patients and controls. As expected, SAA levels were significantly higher in patients (p= 0.0144), and interestingly also OPG levels were significantly higher if compared to the healthy subjects (p= 0.0018). For b-ALP, CTX and RANK-L no differences were observed between the two groups (p=0.1466, p=0.8861, and p= 0.7890 respectively).

**Conclusion:** Patients of our cohort affected by FMF, TRAPS and HIDS do not present an increased risk of OP compared to the healthy controls. Indeed, TBS and BMD are similar between the two groups highlighting a preserved bone quality in our patients. Interestingly, OPG levels are higher compared to controls and this could suggest a protective role and a bone re-balancing activity in a context of inflammation. Finally, a regulatory effect of serum amyloid A on bone homeostasis should be taken into account.

**Disclosure of Interest**

None Declared

#### P1009 Two cases of hyperzincaemia/hypercalprotectinaemia show novel phenotypic properties

##### Anikó Szabó^1^, Péter Blazsó^1^, Viktória Sümegi^2^, Tibor Kalmár^1^, Zoltán Maróti^1^, Csaba Bereczki^1^

###### ^1^Department of Paediatrics; ^2^Department of Rheumatology and Immunology, University of Szeged, Szeged, Hungary

####### **Correspondence:** Péter Blazsó

**Introduction:** Hyperzincaemia/hypercalprotectinaemia (Hz/Hc) is a recently discovered autoinflammatory disorder showing monogenic, autosomal dominant inheritance pattern. Until now the p.E250K and p.E257K missense mutations of PSTPIP1 gene have been found in the background. These result in the extreme overproduction of the pro-inflammatory cytokine IL-1β leading to the striking elevation of serum calprotectin and zinc through positive feedback loops. Uncontrolled inflammation elicited by the continuous activation of these danger signals end up in the classical constellation of systemic and cutaneous inflammation, hepatosplenomegaly, arthritis, pancytopenia, and failure to thrive.

**Objectives:** We aimed to characterize the clinical phenotypes accompanied the mutations of PSTPIP1 gene found in two unrelated Hungarian male patients. These data were compared to the clinical presentations of the already published cases and novel symptoms were demonstrated.

**Methods:** Clinical cases of both boys were investigated thoroughly. In order to clarify the diagnosis targeted exome sequencing panel (Illumina TruSight One) was applied in each case to pre-screen for possible mutations. Nucleotide variants in the probands and in their parents were confirmed by Sanger sequencing.

**Results:** A p.E250K mutation was detected in one patient and a p.E257K in the other. Both variants were *de novo* and heterozygous. p.E250K in the 2,5-year-old boy was associated with additional recurrent non-infectious diarrhea, macrocephaly and pericardial fluid on the top of the classical signs. p.E257K in the 7-year-old boy was joined with haemangiomatosis, mental retardation, autism and overweight besides the already known manifestations of Hz/Hc.

**Conclusion:** Atypical and potentially misleading clinical findings in Hz/Hc underline the importance of genetic testing in order to get to the proper diagnosis. The presented cases might extend our knowledge of the phenotypic traits observable in Hz/Hc.

**Consent for publication has been obtained from patient**

Yes

**Disclosure of Interest**

None Declared

#### P1010 Identification of novel NLRC4 and IL2RA variants in a family cohort with juvenile-onset arthritis and rash

##### Jessica L. Bloom^1^, Megan L. Curran^1^, Scott Canna^2^, Harold Hoffman^3^, Elena Hsieh^4^

###### ^1^Department of Pediatrics, University of Colorado, Aurora; ^2^Departments of Pediatrics and Immunology, University of Pittsburgh, Pittsburgh; ^3^Departments of Pediatrics and Medicine, University of California San Diego, San Diego; ^4^Departments of Pediatrics and Immunology and Microbiology, University of Colorado, Aurora, United States

####### **Correspondence:** Jessica L. Bloom

**Introduction:** Identification of genetic etiologies of autoinflammatory syndromes can inform targeted therapy to improve outcomes.

**Objectives:** We aimed to identify a genetic etiology for an underlying autoinflammatory syndrome in a 3-year-old boy presenting with failure to thrive, rash, and polyarthritis since infancy without significant fevers. The patient’s mother and maternal aunt also had similar symptoms since infancy.

**Methods:** We obtained a history, exam, routine laboratory evaluation, chromosomal microarray, immune phenotyping and functional assays, and genetic sequencing of familial autoinflammatory syndromes via INVITAE.

**Results:** The patient’s first examination showed small and large joint polyarthritis and maculopapular rash. Laboratory results included anemia, positive ANA, negative RF and anti-CCP, mildly raised ferritin, and very elevated platelet level, LDH, ESR, CRP and IgG. He had recurrent diarrhea and tested positive for Campylobacter. INVITAE’s autoinflammatory panel showed two heterozygous variants of unknown significance (VUS): *NLRC4*, exon 4*,* c.741_742insAlu (p.Leu247fs) and *IL2RA*, exon 2, c.76G>C (p.Asp26His)*.*

Genetic testing revealed the same two variants in the patient’s mother and aunt, while unaffected relatives had one or the other (*see Table 1)*. The patient and mother are HLA-B27+ and have the same 2.8 Mb duplication from 3q28 to 3q29 on chromosomal microarray including IL1RAP. Signal transducer and activator of transcription phosphorylation (pSTAT5) studies showed increased baseline pSTAT5 induction without IL-2 stimulation in patient compared to control.

The patient’s arthritis improved partially with naproxen and steroid joint injections. Given genetic results, subcutaneous anakinra was initiated at 10 mg/kg daily with significant improvement in arthritis, rash, and fatigue. Labs after one month showed resolved anemia, normal inflammatory markers, and high IL-18 (7,824 pg/mL, normal 89-540). He switched to 4.29 mg/kg of canakinumab monthly. After one month, he had mildly active arthritis, occasional fatigue, and stable labs apart from an increase of IL-18 to 15,329 pg/mL. The mother, who is poorly controlled off therapy, has elevated IL-18 (7,176 pg/mL)

**Conclusion:** We present a family cohort with juvenile-onset arthritis and rash, found to have elevated IL-18, VUS in both *NLRC4* and *IL2RA*, and a chromosomal duplication consistent with a heritable autoinflammatory syndrome. While it appears that both variants are required for symptomatology, his presentation is most consistent with an unrecognized gain of function *NLRC4* mutation despite lack of recurrent fevers or enterocolitis. Genetic evaluation led to targeted therapy and improved outcomes, with additional testing underway to further personalize therapy.

The patient's family consented for publication.

References

1. Canna SW, et al. An activating NLRC4 inflammasome mutation causes autoinflammation with recurrent macrophage activation syndrome. Nature Genetics. 2014;46(10):1140-6.

2. Romberg N, et al. Mutation of NLRC4 causes a syndrome of enterocolitis and autoinflammation. Nature Genetics. 2014;46(10):1135-9.

**Disclosure of Interest**

None Declared

**Table 1 (abstract P1010).** INVITAE Panel Results

Relation to Patient	Symptoms?	VUS
Patient*	Yes	NLRC4^1^, IL2RA^2^
Sister	No	NLRC4^1^
Mother*	Yes	NLRC4^1^, IL2RA^2^, ADAM17^3^
Father	No	-
Maternal Aunt	Yes	NLRC4^1^, IL2RA^2^
Maternal Half-Aunt	No	-
Maternal Grandfather	No	IL2RA^2^
Maternal Grandmother	No	NLRC4^4^

^1^c.741_742insAlu (p.Leu247fs), heterozygous, ExAC 0

^2^c.76G>C (p.Asp26His), heterozygous, ExAC 0.1%

^3^c.179T>C (p.Leu60Pro), heterozygous, ExAC 0

^4^c.741_742insAlu (p.Leu247fs), possibly mosaic, ExAC 0

*2.8 Mb duplication from 3q28 to 3q29 on chromosomal microarray (maternal aunt's is in process)

#### P1011 Discontinuation of colchicine therapy in children with familial Mediterranean fever

##### Yonatan Butbul^1^, Rawan Silman^1^, Shafe Fahoum^2^, Yackov Berkun^3^

###### ^1^Department of Pediatrics B, , Rappaport Children's Hospital, Rambam Medical Center, Haifa; ^2^Department of Pediatrics B, Rappaport Children’s Hospital, Rambam Medical Center, Haifa; ^3^Department of Pediatrics and FMF Clinic, Hadassah-Hebrew University Medical Center, Mount Scopus, Jerusalem, Israel

####### **Correspondence:** Yonatan Butbul

**Introduction:** Clinical phenotype of FMF exists in some carriers of MEFV mutation. These patients tend to have a mild disease. Prolonged colchicine free remission was reported in a small group of FMF patients.

**Objectives:** To describe and characterize a group of children with FMF in whom colchicine was discontinued.

**Methods:** The study cohort consisted of all children with FMF followed at 2 referral centers in Israel in whom colchicine was discontinued following prolonged attack free period.

Clinical presentation, mutations in MEFV gene and disease outcome of patients who successfully ceased colchicine therapy were compared with patients with relapse of FMF attacks.

We performed a retrospective study in two referral centers in Israel of 43patients with FMF with 1 or non-mutated MEFV allele who ceased colchicine therapy following prolonged attack free period. The phenotype of the patients was investigated in detail, and the MEFV mutations, laboratory findings, clinical picture and outcome of 30 (70%) patients that successfully ceased colchicine therapy were compared to 13 (30%) patients whofailed.

**Results:** 47 patients (55% males), mean age 6±3.2 years at the diagnosis, were enrolled in the study, of them 4patients were excluded due to poor follow up. Fever (93%), abdominal pain (79%), arthralgia (19%) and arthritis (12%) were the most common symptom at attack.The average period free of attacks before enrolment was 11.3±9.2 months. The average follow-up after ceasing colchicine was 5.1± 2.9 years. Thirteenpatients (30.2%) had anattack during follow up with most common symptomsof fever (92%) and abdominal pain (77%) and colchicine therapy was restarted within 10.1 months (1.1-36.4months). There were no differences between the groups of patients that were able to stop colchicine and the group that needed to renew therapy in demographic, genetic and most clinical parameters, including the age (13.4±3.9 vs 11.9±3.7p-0.26), level ofSAA at enrolment (4±3.6 vs 3.3±2.4p-0.7) and time of last attackprior to enrolment (12.6±9.6 vs 8.6±8.2 monthsp-0.08). Myalgia and arthritis were more common among children that required to renew therapy compared to the group that didn’t (31% vs 6.7% p-0.058 and 31% vs 3% p-0.024 respectively).

**Conclusion:** Cessation ofcolchicine therapy following prolonged remission in selected group of patients who are not homozygous for MEFV mutation could be considered. Patients with arthritis or arthralgia are more likely to have an attack after ceasing colchicine therapy.

**Disclosure of Interest**

None Declared

#### P1012 Long-term follow up of a mevalonate deficiency kinase patients cohort

##### Inmaculada Calvo Penades, Berta Lopez Montesinos, M. Isabel Gonzalez Fernandez, Miguel Marti Masanet, Elena Fernandez De La Puebla

###### Pediatric Rheumatology Unit, HUIP la Fe, Valencia, Spain

####### **Correspondence:** Inmaculada Calvo Penades

**Introduction:** The syndrome mevalonate kinase deficiency (MKD) is part of the syndromes of periodic fever. With typical clinical manifestations and good response to treatment with IL-1 blockade, but the long-term manifestations are little known

**Objectives:** To assess the clinical features, treatment and evolution of 11 patients diagnosed with Mevalonate Kinase Deficiency (MKD).

**Methods:** Baseline demographic and clinical characteristics of the patients were considered, including age at symptoms onset, age at diagnosis, time to follow up, clinical features, laboratory data, results of the MVK gene sequencing study, administered treatments and evolution.

**Results:** Overall, 11 patients. Gender: M/W 7:4. Onset age: 0.8 m (0-15 years). Age at diagnosis: 9.6 years (4m-15 years), time of follow up: 9.8 years (4m-11 years). Clinical features: Fever 93% of the patients, adenopathy 93%, abdominal pain 66%, oral aphtha 66%, diarrhea 60%, arthritis 60%, amygdalitis 60%, other symptoms: skin rash 34%, headache, 34%, hepatomegaly 27%, splenomegaly 20% and serositis 10%. Three patients presented atypical manifestations: intestinal obstruction (6 episods), intestinal invagination (4 episodis), orquitis (3 episodis) and chylothorax. All patients showed IgD levels > 100 U/mL (100-1500). MVK mutations: 6 patients homozygotes (2: V250I, 4: V377I) and 5 patients double heterozygotes: 4(I268T, V377I), 1(N205D, R388X). The genetic study of the whole family was performed in 7 families. Corticoid treatment used for 100% of the patients. Anakinra in 45% (parcial-complete response), Canakinumab 82% (complete response), Etanercept 9% (no response) and adalimumab 9% (hydradenitis). Long-term manifestations: 2 patients with cutaneous abscess and 1 patient with hydradenitis suppurativa.

**Conclusion:** In this cohort the high response to canakinumab treatment is shown and late clinical manifestations are described, to our knowledge, for the first time in literature.

**Disclosure of Interest**

None Declared

#### P1013 Novel assay to diagnose and monitor cryopyrin associated periodic syndromes (CAPS)

##### Fortunata Carbone^1,2^, Luca Cantarini^3^, Teresa Micillo^4^, Maria Alessio^5^, Alma Nunzia Olivieri^6^, Maria Francesca Gicchino^7^, Antonella Insalaco^8^, Maria Cristina Maggio^9^, Orso Maria Lucherini^3^, Roberto Scarpioni^10^, Matteo Piga^11^, Maria Maddalena Angioni^11^, Laura Obici^12^, Antonella Simpatico^3^, Pietro Leccese^13^, Rita Consolini^14^, Raffaele Manna^15^, Paolo Sfriso^16^, Sara Bindoli^16^, Paola Galozzi^16^, Ida Orlando^3^, Sabrina Chiesa^17^, Marco Gattorno^17^, Giuseppe Matarese^18,19^

###### ^1^Istituto per l'Endocrinologia e l'Oncologia Sperimentale-Consiglio Nazionale delle Ricerche (IEOS-CNR), Napoli; ^2^Unità di Neuroimmunologia, IRCCS Fondazione Santa Lucia, Roma; ^3^Research Centre of Systemic Autoinflammatory Diseases, Behçet's Disease Clinic and Rheumatology-Ophthalmology Collaborative Uveitis Centre, Department of Medical Sciences, Surgery and Neurosciences, University of Siena, Siena; ^4^Dipartimento di Biologia, Università di Napoli “Federico II”; ^5^Department of Translational Medical Sciences, Section of Pediatrics, Federico II University; ^6^Dipartimento della Donna, del Bambino e di Chirurgia Generale e Specialistica; ^7^Dipartimento della Donna, del Bambino e di Chirurgia Generale e Specialistica, Università degli Studi della Campania “Luigi Vanvitelli”, Napoli; ^8^Department of Pediatric Medicine, Division of Rheumatology, Bambino Gesù Children's Hospital, Roma; ^9^Ospedale dei Bambini “Di Cristina” , Palermo; ^10^Ospedale AUSL “Guglielmo da Saliceto” , Piacenza; ^11^Reumatologia, Policlinico Universitario, Cagliari; ^12^Centro per lo Studio e la Cura delle Amiloidosi, Fondazione IRRCS, Policlinico San Matteo, Pavia; ^13^Rheumatology Institute of Lucania (IReL), Rheumatology Department of Lucania, “San Carlo” Hospital of Potenza and “Madonna delle Grazie” Hospital of Matera, Potenza; ^14^Laboratory of Immunology, Division of Pediatrics, Department of Clinical and Experimental Medicine, University of Pisa, Pisa; ^15^Centro delle febbri periodiche e malattie rare, Policlinico Gemelli, Università Cattolica Roma, Roma; ^16^Unità di Reumatologia, DIMED, Policlinico Universitario, Padova; ^17^Dipartimento di Scienze Pediatriche Generali e Specialistiche, IRCCS Istituto Giannina Gaslini, Genova; ^18^Dipartimento di Medicina Molecolare e Biotecnologie Mediche, Università di Napoli Federico II; ^19^Istituto per l'Endocrinologia e l'Oncologia Sperimentale, Consiglio Nazionale delle Ricerche (IEOS-CNR), Napoli, Italy

####### **Correspondence:** Fortunata Carbone

**Introduction:** Cryopyrin associated periodic syndromes (CAPS) are rare autoinflammatory disorders associated with dominantly gain-of-function mutations in the NLRP3 gene that result in overactivation of the inflammasome, increased secretion of interleukin (IL)-1beta and IL-18, and systemic inflammation. It has been reported that oligomeric particles of the adaptor ASC (apoptosis-associated Speck-like protein with a caspase-recruitment domain) are released together with IL-1beta and active caspase-1 subunits after activation of the inflammosome complex and that patients with CAPS show an increased serum concentration of ASC^+^ particles.

**Objectives:** The diagnosis of CAPS is a critical factor due to both the lack of specific laboratory results and the sharing of similar clinical manifestations with other autoinflammatory diseases, our aim is to develop a simple assay to evaluate the levels of ASC particles in the serum of CAPS patients to provide novel biomarkers facilitating early disease diagnosis and able to monitor treatment responses.

**Methods:** We developed an ELISA for the quantification of ASC particles in serum and plasma of normal and pathological subjects. We analysed samples from CAPS patients and from patients with autoimmune disorders (Multiple Sclerosis (MS), Type 1 Diabetes (T1D) and juvenile idiopathic arthritis), to confirm that ASC presence in the serum is not due to other chronic inflammatory processes characterizing autoimmunity. In addition, we also evaluated the concentration of ASC in the sera of TNF receptor–associated periodic syndrome (TRAPS) patients to reinforce the concept of specificity of this biomarker in CAPS patients and not in individuals suffering from others inflammatory disorders.

**Results:** We observed that untreated CAPS patients are characterized by the presence of a significant higher amount of ASC particles when compared with healthy controls (HS) and with patients suffering from MS and T1D. This tendency was also evident in patients with arthritis and TRAPS even if the difference was not statistically significant due to the small number of samples. In addition there is a tendency through a reduction of ASC levels in CAPS patients after pharmacological treatment, which require future investigations.

**Conclusion:** These data suggest that ELISA quantitation of ASC protein could represent a novel and additional strategy for the diagnosis and monitoring of CAPS.

**Disclosure of Interest**

F. Carbone: None Declared, L. Cantarini: None Declared, T. Micillo: None Declared, M. Alessio: None Declared, A. N. Olivieri: None Declared, M. F. Gicchino: None Declared, A. Insalaco: None Declared, M. C. Maggio: None Declared, O. M. Lucherini: None Declared, R. Scarpioni: None Declared, M. Piga: None Declared, M. M. Angioni: None Declared, L. Obici: None Declared, A. Simpatico: None Declared, P. Leccese: None Declared, R. Consolini: None Declared, R. Manna: None Declared, P. Sfriso: None Declared, S. Bindoli: None Declared, P. Galozzi: None Declared, I. Orlando: None Declared, S. Chiesa: None Declared, M. Gattorno: None Declared, G. Matarese Grant / Research Support from: Giuseppe Matarese reports research grants from Merck-Serono, Biogen Idec, Novartis and IBSA.

#### P1014 An Italian family with FCAS

##### Maria Carrabba^1^, Marina Zarantonello^2^, Isabella Ceccherini^3^, Giovanna Fabio^1^

###### ^1^Internal Medicine - Rare Diseases Unit, Fondazione IRCCS Ca’ Granda Ospedale Maggiore Policlinico; ^2^Department of Clinical Sciences and Community Health , Università degli Studi, Milan; ^3^UOC Genetica Medica, UOS Diagnostica Molecolare e Malattie Ereditarie, Istituto Giannina Gaslini, Genoa, Italy

####### **Correspondence:** Maria Carrabba

**Introduction:** The prevalence of Cryopyrin-Associated Periodic Syndrome (CAPS) is estimated at about one per million. The Familial Cold Autoinflammatory Syndrome (FCAS) presents in about 95% of the patients by 6 months with cold-induced, urticaria-like rash, fever, and arthralgia. FCAS causes lifelong debilitating effects that restrict patients’ daily activities. Diagnostic delay related to lack of knowledge in Autoinflammatory Diseases is still an important problem.

**Objectives:** To report an Italian family with FCAS successfully treated with anti-IL1.

**Methods:** Three subjects, members of the same family, were screened by an experienced doctor. Clinical and laboratory variables, Brain-CT scan, X-ray, audiometry and lung function tests were performed. A targeted review of clinical feature for Autoinflammatory diseases and standardized questioning for CAPS-associated symptoms was conduct. Genetic testing for the NLRP3 mutation was performed.

**Results:** A 51 years old woman presented with a long history of maculopapular rash after cold exposure starting in early childhood associated with low-grade fever, higher during childhood. During cold months, she needed daily FANS because of fever, and topical steroids for skin rash, prescribed by dermatologists. The patient was in chronic therapy with local steroids because of recurrent ulcerative keratitis. Some corneal scars are present as outcomes. She experienced ocular manifestation on exposure to cold and summertime in contact with air conditioning. She has a 18-years daughter and a 15-years son with a long history of maculopapular rash after cold exposure starting in childhood associated with low-grade fever, fatigue, headache and arthralgia (more severe in the son) lasting less than 24hours. The two children had the same ocular manifestations of the mother and underwent local steroids therapy 4-5 times per year. They all carried the A439V mutation on NPLR3 gene exon 3. Patients were vaccinated (Pneumococcus, HBV, HAV) before starting treatment for anti-IL1 (canakinumab, a fully human anti-IL-1β monoclonal antibody, at a dose of 150 mg subcutaneous every 8 weeks). The Auto-Inflammatory Disease Activity Index (AIDAI, the simplified items scored 0–1 (CAPS range 0–155)) and the Autoinflammatory Disease Damage Index (ADDI) scores were assessed before every treatment. Before treatment, the AIDAI score calculated during a winter and summer month was respectively 59 and 5 for the mother, 30 and 3 for the daughter and 40 and 3 for the son. After 12-months of treatment, the AIDAI score is zero for the mother and the daughter as well as the ADDI score. During treatment mother experienced a urinary tract infection and the daughter two distinct infectious episodes: one of pharyngitis and one of diarrhoea. The son delayed his treatment because of a severe chronic sinusitis that needed surgical treatment. He just received his first dose last month.

**Conclusion:** The NLRP3 mutation A439V was first entered into the Infevers database in 2002 by Hal Hoffman. It is one of the most common mutations in multiplex families with CAPS and it is considered a CAPS disease-causing mutation, although the overall clinical phenotype is rather mild. Most of the patients reported with FCAS carrying A439V have ocular manifestations (conjunctivitis, keratitis, uveitis) that causes lifelong debilitating effects that restrict patients’ daily activities, as well as rash or low-grade fever or myalgia/arthralgia.FCAS-associated ocular manifestations need treatment with anti-IL1, which typically results in dramatic improvement in clinical and laboratory measures of inflammation, and is well tolerated. Unfortunately, ophthalmologists, dermatologist as well as other specialists do not recognise FCAS and do not appropriately treat this disease nor send patients to Autoinflammatory Diseases experienced Centres.

**Consent for publication has been obtained from patient**

Yes

**Disclosure of Interest**

None Declared

#### P1015 Scores assessment for clinical management of familial Mediterranean fever

##### Maria Carrabba^1^, Marina Zarantonello^2^, Giovanna Fabio^1^

###### ^1^Internal Medicine - Rare Diseases Unit, Fondazione IRCCS Ca’ Granda Ospedale Maggiore Policlinico; ^2^Department of Clinical Sciences and Community Health, Università degli Studi, Milan, Italy

####### **Correspondence:** Maria Carrabba

**Introduction:** Clinical spectrum of Familial Mediterranean fever (FMF) is heterogeneous, ranging between minimal activity of few affected sites and excellent response to colchicine and large number of frequent, intolerable, treatment-resistant attacks. Frequent and severe FMF attacks may result in serious complications. Severity scoring systems for FMF have been developed and validated in adults (1998 Pras et al., 2005 Mor et al.) to objectively quantify the disease severity for both therapeutic and prognostic purposes. In 2016 the ISSF score has been developed with a consensus-driven methodology by paediatricians and internists, with expertise in this disease, and validated in a large database comprising both children and adults with FMF. Recently, the ADDI score, which was developed based on consensus building, purposes to measure the chronic damage by Autoinflammatory Diseases.

**Objectives:** This study aims to assess the performance of the existing severity scores (Mor 2005 and ISSF 2016) for FMF in predicting the therapeutic outcome (colchicine dosage and residual attacks); and to assess the relation between the above scores and the ADDI score.

**Methods:** All the patients with FMF in charge since 2003 have been enrolled. Severity scores have been calculated at the diagnosis and ADDI has been calculated at the latest visit. ROC curves and statistical analysis have been performed.

**Results:** Forty-five consecutive patients affected by FMF (follow-up 1-41 years) with a median age of 39 years (range 9-89) have been evaluated. Ten patients were diagnosed for FMF in childhood and 35 in adulthood, with a median of diagnostic delay of 12 years (range 1-45). Eight patients are homozygous carries of one mutation on MEFV gene exon 10, twelve patients are heterozygous carriers of two mutations on MEFV gene exon 10, and twenty patients are heterozygous carriers of one mutation on MEFV gene exon 10.

All patients except four are under therapy with colchicine. Nine patients need more than 1mg per day of colchicine. Ten patients reported one or two mild FMF attacks during the year before the last visit. According to the severity score of Mor et al., 15 patients were stratified as mild disease, 20 as intermediate and 10 as severe. According to the ISSF score, 37 patients had a mild disease, eight intermediate and no one severe. Sixteen patients have a positive ADDI score (range 0-4). The ROC analysis shows that the score of Mor performed better than the ISSF (AUC 0.903vs0.661) to identify patients needing more than 1mg of colchicine per day. The ISSF has a good performance (AUC 0.694) to identify patients referring one or two mild attacks in the year before the last visit. The ROC curves analysis shows that both the Mor (AUC 0.677) and the ISSF (AUC 0.657) scores can identify patients with chronic damage (positive ADDI score). ROC analysis of Mor, ISSF and ADDI scores for FMF genetic pattern, showed that only ADDI has a good performance (AUC 0.811) to discriminate the FMF homozygous patients.

**Conclusion:** The score of Mor et al. can be useful for prediction of FMF patients needing higher colchicine dosage. The ISSF score seems to predict better patients with one or two mild attacks per year. The most of homozygous FMF patients have a positive ADDI score despite therapy.

**Disclosure of Interest**

None Declared

#### P1016 Single center experience with 402 familial Mediterranean fever patients

##### Ozlem Ozdemir Isik, Senem Tekeoglu, Duygu Temiz Karadag, Ayten Yazici, Ayse Cefle

###### Department of Internal Medicine Division of Rheumatology, Kocaeli University Faculty of Medicine, Kocaeli, Turkey

####### **Correspondence:** Ayse Cefle

**Introduction:** Familial Mediterranean Fever (FMF) is an autosomal recessive genetic disorder that causes recurrent episodes of fever, poliserositis, arthritis, skin eruptions.

**Objectives:** In this study, we aim to present clinical and demographic features of FMF patients followed in our clinic.

**Methods:** The clinical, demographic, genetic features and management of 402 FMF patients (fulfilling Tel-Hashomer Diagnostic Criteria) were analyzed.

**Results:** The mean age was 36,8±11,2 (10-71), the mean diagnosis age was 28±11,9 (3-66) years, and mean duration of disease was 189±125 months. Mean duration between disease onset and treatment was 93.6±104 months. 43% (174) of the patients had positive family history for FMF, 7% (29) had consanguineous marriage in family. 24% of the patients had appendectomy. Fever and abdominal pain both were initial symptoms in 72% of the patients, while 7% of them had chest pain, 4% had only fever, 15% had arthritis, 1% had erysipelas like erythema (ELE) as the first symptom of FMF.

Fever was observed 76% of the patients, abdominal pain in 86%, ELE in 13%, chest pain in 21%, and arthritis in 32%. The frequency of monoarthritis was 21%, oligoarthritis 10,5% and polyarthritis was identified 0,5% of the patients. Ankle arthritis was the most frequent(20%) one among the patients who had monoartritis. 16% of the patients had inflammatory back pain, while 11% of the patients were identified with sacroiliitis. 8 patients (2%) suffered from chronic kidney disease and 2 of them were on dialysis programme. Amyloidosis (diagnosed with biopsy) was identified among 14 patients (3,5%).

At least one mutation of MEFV gene was identified in 78% of the patients. No mutation could be identified in 8%. MEFV gene analyses was not performed in 14% of the patients. The most frequent mutation was M694V mutation and its allel frequency was found to be 54%. For V726A, M680I, E148Q, R761H and A744S allels, mutation frequency were in order of 11%, 7%, 7%, 2%, 1%. The frequency of compound heterozygositywas 38% and the most common genotype wasM694V/R202Q (11%).

There was a significant relationship between M694V mutation and arthritis, ELE, amyloidosis and sacroiliitis (p<0.001). Amyloidosis was more frequent in patients with M694 homozygous mutation. Mean age of disease onset was lower in patient who had M694V homozygous mutation than M694V heterozygous mutation (p<0.001).

**Conclusion:** The most common mutation in our patients is M694V mutation and it is significantly associated with arthritis, ELE, sacroiliitis, and amyloidosis. Tight control and regular treatment are important in FMF patients to protect from amyloidosis.

**Disclosure of Interest**

None Declared

#### P1017 Testicular ischemia in deficiency of adenosine deaminase 2 (DADA2)

##### Katherine Clarke, Cathy Campbell, Ebun Omoyinmi, Ying Hong, Muthana Al Obaidi, Neil Sebire, Paul Brogan

###### Paediatric Rheumatology, Great Ormond Street Hospital, London, United Kingdom

####### **Correspondence:** Katherine Clarke

**Introduction:** Deficiency of adenosine deaminase 2 (DADA 2) is a rare autosomal recessive autoinflammatory condition. Recognised features include vasculitis predominantly affecting medium sized vessels, livedoid skin rash, central and peripheral nervous system involvement, variable degrees of immunodeficiency, and marrow failure amongst other clinical presentations. We present the case of a six year old male with DADA 2 who presented with acute testicular ischaemia secondary to vasculitis, the first such description in DADA2.

**Objectives:** We wish to highlight the ever-expanding phenotype of DADA2, and to emphasise the ongoing controversies regarding management of this lifelong genetic disease. Improvements in our understanding of the pathogenesis will ultimately lead to better biomarkers, better treatments, and ultimately even cure using gene therapy based on the favourable clinical outcomes of patients undergoing allogeneic HSCT thus far.

**Methods:** A six year old male presented acute right-sided testicular pain. His history included transient infantile neutropenia, resolved hepatosplenomegaly, and longstanding livedo racemosa, leading to screening and confirmation of DADA2 caused by homozygous c.139G>C(p.G47R) mutation of *ADA2.* As his only clinical feature was that of mild livedo racemosa with normal laboratory parameters at diagnosis, he was being actively monitored prior to starting any treatment. At a routine clinic follow-up a 24 hour history of testicular pain was noted on systems review; he was afebrile, and his only physical signs were that of moderate livedo racemosa, and tenderness of the right testicle. Laboratory parameters revealed C-reactive protein 8mg/L (reference range [RR]<20mg/L); erythrocyte sedimentation rate 28 mm/hr (RR<10); and serum amyloid A 5mg/L (RR<10). Ultrasound-scan of the scrotum revealed significantly reduced perfusion of the right testes, without torsion; and surgical scrotal exploration confirmed testicular ischaemia without torsion. Histology demonstrated ischaemic seminiferous tubules with intervening haemorrhage and acute inflammatory cells, consistent with vasculitis of the testis as the cause. He was treated with high dose intravenous methyl-prednisolone followed by a weaning course of oral prednisolone, and subcutaneous adalimumab (anti-tumour necrosis factor alpha, anti-TNFα). Repeat ultrasound-scan 3 weeks later revealed good testicular perfusion, with a small area of focal infarction. At last follow-up (11 months post-event) he remained asymptomatic, on treatment with adalimumab.

**Results: Figure 1**: Livedo racemosa noted on both lower limbs.

**Figure 2:** Doppler study of both testes (transverse views) showing globally reduced (but not absent) perfusion in the right testes compared to the left.

**Figure 3:** Photomicrograph of testicular biopsy showing patchy areas of tubular necrosis with loss of cellular and nuclear detail and nuclear ‘smudging’ (Right side) with more normal, viable tubules (Left side); H&E original magnification x100

**Figure 4:** Acute phase markers before, during, and after acute testicular infarction in DADA2. Acute phase responses were completely normal prior to testicular infarction. C-reactive protein (CRP) and serum amyloid A (SAA) remained within the normal range throughout the episode and follow-up. Erythrocyte sedimentation rate (ESR) was normal, but was transiently modestly elevated on the day of tissue infarction (arrowed), remaining normal at follow-up.

**Conclusion:** The phenotype of DADA2 continues to expand, and we add testicular infarction to the features of DADA2. CRP and SAA cannot be relied on as reliable biomarkers to predict tissue ischaemia and hence who to target for anti-TNFα therapy in DADA2, since these remained steadfastly normal before, during, and after testicular infarction in this case

**Consent for publication has been obtained from patient**

Yes

**Disclosure of Interest**

None Declared

#### P1018 A novel duplication in the X-linked inhibitor of apoptosis protein gene leading to recurrent hemophagocytic lymphohistiocytosis that is responsive to interleukin-1 blockade

##### Dilan Dissanayake^1^, Rebecca Marsh^2^, Ahmed Naqvi^3^, Michael Jordan^2^, Rae Yeung^1^, Ronald Laxer^1^

###### ^1^Paediatric Rheumatology, The Hospital for Sick Children, Toronto, Canada; ^2^Bone Marrow Transplantation and Immune Deficiency, Cincinnati Children's Hospital, Cincinnati, United States; ^3^Paediatric Haematology/Oncology, The Hospital for Sick Children, Toronto, Canada

####### **Correspondence:** Dilan Dissanayake

**Introduction:** We present here a 9 year-old male with recurrent episodes of hemophagocytic lymphohistiocytosis (HLH), characterized by fever, cytopenias, transaminitis, hyperferritinemia, hypofibrinogenemia, and elevated levels of soluble CD25 and soluble CD163, in whom we investigated for an underlying genetic defect.

**Methods:** Peripheral blood samples were obtained from the patient and his family members for analysis using next generation sequencing by gene panels commonly associated with HLH and recurrent fever syndromes, followed by whole exome and whole genome sequencing.The patient’s blood was also assessed using an enzyme-linked immunosorbent assay for serum interleukin (IL)-18. Intracellular X-linked Inhibitor of Apoptosis Protein (XIAP) levels were measured by flow cytometric analysis of peripheral blood mononuclear cells (PBMC).The function of XIAP was assessed by a previously established assay that measures intracellular cytokine staining for tumor necrosis factor alpha (TNFa) production following stimulation of PBMC by muramyl dipeptide (MDP), the ligand for the Nucleotide-binding Oligomerization Domain-containing protein 2 (NOD2) receptor.

**Results:** The HLH and recurrent fever syndrome gene panels, as well as whole exome sequencing were initially unable to identify a contributory variant in this patient.However, we strongly suspected a primary form of HLH after having measured persistently elevated levels of serum IL-18 to 2524 pg/ml in between episodes of HLH.Furthermore, we discovered a family history of recurrent fevers in the maternal grandfather, which was suspicious for an X-linked condition, such as XIAP deficiency. While awaiting results of whole genome sequencing, we performed flow cytometry for XIAP, which demonstrated reduced protein levels in the patient’s T-lymphocyte, B-lymphocyte and natural killer cell populations.Subsequent results from whole genome sequencing demonstrated a large duplication spanning three exons within the XIAP gene, which was present in the patient, his asymptomatic mother, and his maternal grandfather. In addition to decreased protein expression, MDP stimulation of the patient’s PBMC confirmed a significant functional defect in XIAP function downstream of the NOD2 receptor.While the above testing was being completed, the patient was started on anakinra and successfully weaned off steroids, with no further recurrences of HLH to date.

**Conclusion:** This case describes the identification of a previously undescribed large duplication within the XIAP gene, which was initially not identified on gene panels and whole exome sequencing.This genetic variant is associated with reduced expression of XIAP in peripheral blood mononuclear cells, as well as decreased function downstream of the NOD2 receptor.Interestingly, while patients with XIAP deficiency are typically treated with hematopoietic stem cell transplantation, this patient has been successfully maintained on anakinra, suggesting an IL-1 dependent link between XIAP and recurrent HLH. We thereby illustrate the utility of combining genetic and molecular tools for the identification of contributors to unexplained autoinflammatory presentations, and present IL-1 blockade as a potential safe and effective alternative for some patients with XIAP deficiency.

**Consent for publication has been obtained from patient**

Yes

**Disclosure of Interest**

None Declared

#### P1019 10 year prognosis of patients diagnosed with familial Mediterranean fever

##### Serdal Ugurlu^1^, Bugra H. Egeli^2^, Asli E. Soykut^2^, Bilgesu Ergezen^2^

###### ^1^Division of Rheumatology, Department of Internal Medicine, Cerrahpasa Medical Faculty; ^2^University of Istanbul - Cerrahpasa, Istanbul, Turkey

####### **Correspondence:** Bugra H. Egeli

**Introduction:** In Familial Mediterranean Fever (FMF), other than amyloidosis factors affecting mortality are being debated. In our previous study, we did not observe any atherosclerotic plaque formation in carotid or femoral artery. We thought that the risk of atherosclerosis did not increase in patients diagnosed with FMF.

**Objectives:** The aim of this study was to assess the 10 year prognosis and comorbidity of patients diagnosed with FMF who have been treated in our rheumatology clinic.

**Methods:** The sample group is a subset of 2009 study. In 2009, the patients who already had myocardial infarction or cancer diagnosis were excluded. The patients were interviewed with polar questions of whether they were diagnosed with acute myocardial infarction (AMI), cerebrovascular events, cancer, diabetes, and hypertension.

**Results:** We studied 71 patients (37 males, 34 females; mean age: 49.66±6.91) with FMF, and 59 patients (24 males, 35 females) in healthy control (HC) group. The gender and age difference between two groups was not found significant.

During 10 year follow-up, 8% of FMF patients had either a cardiovascular or cerebrovascular event comparing to 5% in HC (p>0.05). 3% of FMF patients had a cancer diagnosis comparing to 3% in HC (p>0.05). Even though diabetes mellitus diagnosis rate was higher in FMF patients (15% to 10%), results were still not significant (p>0.05). Hypertension diagnosis was 5% higher in FMF group (p<0.05)

**Conclusion:** Even though there was a significant increase in hypertension, increased diabetes, cancer, and AMI/Stroke ratio was not found significant when compared to the HCs. Therefore, any cardiovascular and malignancy related comorbidities are not associated with FMF.

**Disclosure of Interest**

None Declared

**Table 1 (abstract P1019).** Prognostic Factors of FMF patients compared with Healthy Controls

	FMF 2018, n(%)	HC 2018, n(%)	p value
Female	34 (47.89)	35 (59)	0,193
Age	49±6.91	51±5.59	0,076
AMI/Stroke	6 (8.45)	3 (5.08)	0,45
Cancer	2 (2.82)	2 (3.39)	0,85
DM	9 (14.86)	6 (10.17)	0,198
Hypertension	25 (33.78)	10 (16.95)	0,019*
Total	71	59	

#### P1020 In a familial Mediterranean fever prevalent region, are familial Mediterranean fever and Behçet’s disease associated?

##### Ozgur Alparslan^1^, Bugra H. Egeli^2^, Yeltekin Demirel^3^, Serdal Ugurlu^4^

###### ^1^Gaziosmanpasa University, Tokat; ^2^university of Istanbul- Cerrahpasa, Istanbul; ^3^Sivas Cumhuriyet University, Sivas; ^4^Division of Rheumatology, Department of Internal Medicine, Cerrahpasa Medical Faculty, University of Istanbul- Cerrahpasa, Istanbul, Turkey

####### **Correspondence:** Bugra H. Egeli

**Introduction:** The co-existence of Familial Mediterranean Fever (FMF) and Behçet’s Disease (BD) has been questioned. There have been a variety of claims on a common pathogenesis.

**Objectives:** We intended to report the prevalence of Familial Mediterranean Fever (FMF) and Behçet’s disease (BD) and comorbidity ratio of these two diseases in Sivas, Turkey, a city where FMF is known to be very high.

**Methods:** Seventy-two primary schools in the center of Sivas participated in the study. A total of 14881 randomized sample children from 6th, 7th, and 8th grades, and also 985 of them with their parents (n: 978) were interviewed. During these interviews, the family tree up to second degree relatives was drawn. The presence of a diagnosis of FMF or BD was questioned. The ones who have a diagnosis were confirmed by contacting the medical centers. The ones who were suspected of a disease were further investigated at Sivas Cumhuriyet University Medical Faculty, Family Medicine Outpatient unit. For each disease a disease related history, physical examination, eye examination and pathergy test for BD were performed when needed.

**Results:** 985 students, 978 mothers, 953 fathers and 1876 relatives (4792 in total) were included in the study. Only 30 (0.6%) of the sample was diagnosed with FMF, and 3 (%0.06) was diagnosed with BD. One of them had concomitant FMF diagnosis.

**Conclusion:** The prevalence of FMF in Sivas is higher than Turkey’s prevalence; however, BD prevalence was found very low. According to these findings, it is not easy to conclude that these two diseases share a similar background of pathogenesis.

**Disclosure of Interest**

None Declared

**Table 1 (abstract P1020).** FMF symptoms within the last year

Symptoms	N	%
Abdominal Pain	20	66.7
Fever	23	76.7
Joint Pain	8	26.7
Chest Pain	10	33.3
Muscle Pain	7	23.3
Erysipelas like erythema	5	16.7

#### P1021 Does testing for SAA is more beneficial than CRP for the follow-up of patients with FMF?

##### Oguzhan Selvi, Serdal Ugurlu, Bilgesu Ergezen, Huri Ozdogan

###### Division of Rheumatology, Department of Internal Medicine, Cerrahpasa Medical Faculty, University of Istanbul - Cerrahpasa, Istanbul, Turkey

####### **Correspondence:** Bilgesu Ergezen

**Introduction:** In order to follow subclinical inflammation and adjust the therapy for an optimal disease control, clinicians seek for readily accessible, affordable and reproducible markers. C reactive protein (CRP) is widely used for this purpose. Some suggest that CRP measures are not conclusive in all cases, especially in initial stages of inflammation. It is suggested that Serum Amyloid A (SAA) may be more reliable and sensitive in predicting an ongoing inflammation.

**Objectives:** It is aimed to evaluate if SAA and CRP is correlated in M694V homozygous FMF cases and if one is superior to the other with regards to early sensitivity.

**Methods:** In order to evaluate and to compare the sensitivity of SAA and CRP, 234 measurements from 40 FMF patients with M694V homozygous mutation were obtained during a mean follow-up of 5 months.For the analysis, the folds of normal CRP and SAA values were used for correlation.Serum levels of the given markers were measured with nephelometric kits (normal CRP levels <5 mg/L and SAA levels <6,8 mg/L).

**Results:** All patients were on prophylactic colchicine. Among 40 patients 1 patient was being treated with tocilizumab, 2 patients with adalimumab, 19 patients with anti-IL-1 regimens. There were a total of 234 measurements of CRP and SAA from 40 patients.A similar significant correlation was found when we tested only the values obtained during 202 attack-free occasions (r = 0,863, p < 0,01). Both acute phase reactants were increased in 169 measurements, while in 15 CRP was high but SAA was normal and in 40 SAA was high however CRP was within normal limits. 13 patients has amyloidosis. A number of 86 CRP and SRR measures, these two parameters were correlated in patients with FMF Amyloidosis (r = 0,818, p < 0,01). The mean increase in CRP of the population was 2,82 ± 4,52 fold, whereas mean increase in SAA was 8,47 ± 15,15 fold of the normal.

**Conclusion:** According to these results, serial testing of SAA does not provide any additional advantages over CRP. Follow-up with CRP measures particularly in patients with amyloidosis might be adequate when it is considered that SAA is not superior to CRP. Readily accessible and affordable bio-marker CRP seems to be sufficient for follow-up of patients with FMF.

**Disclosure of Interest**

None Declared

#### P1022 The pregnancy outcomes in FMF patients who are exposed to IL-1 blockade with anakinra

##### Bilgesu Ergezen, Serdal Ugurlu, Huri Ozdogan

###### Division of Rheumatology, Department of Internal Medicine, Cerrahpasa Medical Faculty, University of Istanbul - Cerrahpasa, Istanbul, Turkey

####### **Correspondence:** Bilgesu Ergezen

**Introduction:** Some colchicine resistant and/or intolerant FMF cases as well as some patients who experience severe FMF episodes during pregnancy may require alternative therapies during their pregnancies. An Anti-IL-1 agent, Anakinra is being used for this purpose although is still under investigation of researchers.

**Objectives:** To assess the safety of anakinra in pregnant FMF patients and its effect on fetal and maternal outcomes.

**Methods:** Thirteen patients who were exposed to anakinra during their pregnancies were monitored closely for disease activity, side effects, fetal USG and pregnancy outcome.

**Results:** A total of 13 FMF cases followed in our clinic were exposed to Anakinra during the course of their pregnancies due to severe protracted febrile myalgia in 4, thrombocytopenia in 1 and amyloidosis in 1 and severe attacks during pregnancy in 6. One patient had a single injection of anakinra due to pericarditis at 2^nd^ GW and had a spontaneous abortus at 4^th^ GW. Among 13 patients, 2 are still pregnant and both at 8^th^ GW, one started Anakinra at 5^th^ GW, while the other concieved on Anakinra. One of our patients was reported previously by Lachman et al^1^. We represent detailed data of 9 pregnancies which we have conclusive data regarding the whole pregnancy as well as the follow up period after birth. The relation of the use of the drug and pregnancy are represented in Table 1.

**Conclusion:** Anakinra seems to be a very effective alternative in the treatment of protracted febrile myalgia non-responsive to colchicine and corticosteroid therapy, even in pregnant FMF patients who have active disease despite colchicine or intolerant and also can be given transiently during pregnancy and successfully stopped after delivery

**Reference**

1) Lachman HJ, et al. Anti IL-1 therapies and pregnancy outcome. Pediatric Rheumatology 2013, 11 (Suppl 1):A269

**Consent for publication has been obtained from patient**

Yes

**Disclosure of Interest**

None Declared

**Table 1 (abstract P1022).** See text for description

Case	Maternal Age at pregnancy	Anakinra Relation to pregnancy	USGs	Weeks at delivery	Gender of the baby/fetus	Mode of the delivery	1^st^ minute APGAR	Follow-up duration after birth (months)	Complications after birth
1	28	started at 12^th^ Gestational Week (GW) and used until birth	normal	Birth at 40^th^ GW	girl	vaginal	10	34 mo	No
2	31	Conceived on Anakinra (have been on Anakinra since 2012), discontinued at 29^th^ GW, reintroduced at 33^th^ GW due to symptom flare.	normal	Birth at 38^th^ GW	boy	C/S	6	48 mo	Methicillin-Sensitive Staphylococcus Aureus incision-site infection (treated with Tygecycline) in mother
3	24	started at 15^th^ GW and useduntil birth	normal	Birth at 38^th^ GW	boy	Vaginal	8	48 mo	thrombocyte count of the baby was low (23,000/mm^3^) (After three courses of IVIG, it was increased to 95,000/mm^3^ and a month later to 269,000/mm^3^)
4	32	started at 16^th^ GW, continued for 6 months following the birth at 31^th^ GW	normal	Birth at 31^th^ GW	2 girls	C/S	8 for both babies	42 mo	No
5	25	Started at 23^th^ GW used until birth at 37^th^ GW	normal	Birth at 37^th^ GW	girl	C/S	7	42 mo	Injection site reaction
6	29	Started at 32^nd^ GW, used until birth 40 th GW	normal	Birth at 40^th^ GW	Girl	C/S	?	37 mo	No
7	33	Started at 2013, conceived under Anakinra. Anakinra was stopped at the first month of pregnancy, she flared and it was reintroduced.	Normal	Birth at 38^th^ GW	Girl	C/S	8	42 mo	No
8	30	Started at 34th GW, used until still birth at 37^th^ GW	Intrauterine death at 37^th^ GW	Pregnancy terminated at 37^th^ GW	Boy	Normal	0	10 mo	No
9	Zeynep	Started at 6^th^ GW, still using after birth	Normal	Birth at 36th GW	Girl	C/S	9	9 mo	No

#### P1023 Clinical picture of 7 PAPA patients followed in a single pediatric rheumatologic center

##### Silvia Federici^1^, Camilla Celani^1^, Virginia Messia^1^, Giulia Marucci^1^, Christoph Kessel^2^, Fabrizio De Benedetti^1^, Antonella Insalaco^1^

###### ^1^Division of Rheumatology, IRCCS, Ospedale Pediatrico Bambino Gesu’, Rome, Italy; ^2^Department of pediatric Rheumathology & immunology, University Children’s Hospital, Muenster, Germany

####### **Correspondence:** Silvia Federici

**Introduction:** Pyogenic sterile arthritis, pyoderma and acne (PAPA) syndrome is an autosomal dominant inflammatory disorder caused by mutations in the *PSTPIP1* gene primarily affecting joints and skin. The E250K mutation cause the hyperzincaemia/hypercalprotectinemia syndrome termed *PSTPIP1*-associated-related proteinemia inflammatory (PAMI) syndrome in which a bone marrow involvement is reported

**Objectives:** To describe the clinical presentation of 7 PAPA patients followed at a single pediatric rheumatology center

**Methods:** For each patient clinical and laboratory data were collected from medical charts. *PSTPIP1* was sequenced through Sanger Sequencing or targeted resequencing using a customized panel and analyzed with the NextSeq® platform (Illumina)

**Results:** We describe 7 patients from 4 unrelated families with the E250K mutation in a mother and 2 siblings, the A230T variant in a father and his son and the R405C and D266N respectively in the last 2 unrelated patients. Disease onset occurred within the 7^th^year of life in all patients. Patients 3 and 4 (table) presented since the 1st year of life recurrent episodes of fever without any cutaneous or articular symptoms. In both patients inflammatory markers were elevated during fever episodes but persistently negative during well-being not requiring any therapy. The variants described in these patients were not previously reported. However their pathogenic role is supported by the detection of markedly high serum calprotectin levels (>10.000 microg/ml). The predominant feature of patients 1 and 2 was articular involvement with recurrent episodes of arthritis associated to acne. Patient 1 was initially treated with prednisone with good clinical response but relapse of arthritis at discontinuation followed bythe development of a sterile muscle abscess. An anti-TNF drug was started in both patients with complete clinical response. Patient 5 reported severe acne and psoriasis, and recurrent episodes of sterile arthritis. She presented a persistent elevation of acute phase reactants with severe anemia and leukopenia not resolving after splenectomy. His son (pts 6) presented with recurrent episodes of sterile arthritis, hepato-splenomegaly, anemia and neutropenia. Zinc and calprotectin serum levels resulted respectively 728 micromol/l and 2600 microg/ml. IL-1 inhibition determined a complete normalization of inflammatory parameters with no effects on anemia and neutropenia. In patient 6 zinchemia decreased to almost normal value after 4 months of therapy. Patient 7 presented at the age of 4 years a sterile lymphnode abscess. She also presented with splenomegaly and neutropenia with persistent elevation of acute phase reactants. Anakinra was proposed but not administered for poor compliance

**Conclusion:** The clinical picture of patients carrying *PSTPIP1* mutation may be heterogeneous. In our cohort TNF-inhibitors were successfully used in PAPA patients preventing new arthritis episodes and resolving cutaneous manifestation where present. In 2 patients the clinical picture was mild not requiring continuous treatment. One PAMI patient had a good response to IL-1 inhibition, which however, had no effect on hematological manifestations

**Consent for publication has been obtained from patient**

Yes

**Disclosure of Interest**

None Declared

**Table 1 (abstract P1023).** See text for description

Patients	Mutation	Clinical features	Laboratory features	Response to therapy
1	A230T	Recurrent fever, acne, cutaneous abscesses, pyogenic arthritis	←CRP,ESR,SAA	Anti-TNF
2	A230T	Acne, pyogenic arthritis	←CRP,ESR,SAA	Anti-TNF
3	R405C	Recurrent fever	← CRP,ESR,SAA, Zinc	/
4	D266N	Recurrent fever	←CRP,ESR,SAA, Zinc	/
5	E250K	Acne, psoriasis, pyogenic arthritis, hepatosplenomegaly	←CRP,ESR,SAA, Zinc, serum calprotectin	/
6	E250K	pyogenic arthritis,hepato- splenomegaly	←CRP,ESR,SAA, Zinc, serum calprotectin	IL-1 inhibition
7	E250K	Limphnode abscess, hepatosplenomegaly	←CRP,ESR,SAA	/

#### P1024 Clinical presentation, genetic analysis and IFN-score in patients with undefined interferonopathies

##### Silvia Federici^1^, Gianmarco Moneta^1^, Chiara Passarelli^1^, Claudia Bracaglia^1^, Luana Raffaele^1^, Fabrizio De Benedetti^2^, Antonella Insalaco^1^

###### ^1^Division of Rheumatology; ^2^Ospedale Pediatrico Bambino Gesù, Rome, Italy

####### **Correspondence:** Silvia Federici

**Introduction:** A group of genetic disorders with a disturbance of the homeostatic control of IFN-mediated immune responses, have been identified (type I interferonopathies). An increased expression of type I IFN regulated genes, IFN signature (IS), is reported

**Objectives:** To evaluate the correlation between clinical presentation, genetic analysis and IFN-score in 10 pts with undefined interpheronopathies

**Methods:** Patients with suspected interferonopathy based on the presence of typical clinical manifestations (neurological, muco-cutaneous symptoms), laboratory parameters (complement deficiency, low platelet count, presence of autoimmunity), instrumental abnormalities (cerebral calcification), were screened for the IFN-score. Defined IFN-mediated diseases were excluded. Patients with IFN-score above 10 underwent genetic screening by running a panel of 24 genes involved in interferonopathies

**Results:** 10 pts followed in a pediatric rheumatology center were included. 7/10 presented with recurrent fever (table). Pts 2, 3 and 7 displayed neurological manifestation, respectively epilepsy, epilepsy and mental retardation and progressive hemiplegia. To note epilepsy in pts 2 might be due to a bilateral intraventricular hemorrhage presented at birth. In pts 1,4 gastrointestinal manifestation resembling inflammatory bowel diseases were described while pts 5,7 and 10 suffered from recurrent abdominal pain, diarrhea and patient 10 from hypertransaminasemia. Half of the patients complained arthromyalgia; arthritis developed in pts 2. Cutaneous involvement presented in pts 1,3,6 respectively with widespread panniculitis of trunk and limbs, aspecific vasculitis and Schonlein Henoch purpura (HSP). Other cutaneous manifestation were urticarial rash (pts2) and an erythematous, desquamative confluent eczema (pts 4). Autoimmunity was detected in 2/10 pts. Pts 4 8 had an immunological defect with recurrent infections. The genetic analysis resulted negative in pts 1 and 7 and is ongoing in patients 5,6 and 8. Patients 2,3,4,9 and 10 carried one mutation in at least one IFN correlated gene not confirming the diagnosis. All patients presented an increased IS ranging from 14 to 172.

**Conclusion:** An elevated IFN-score represent a useful instrument in clinical practice to classify patients with suspected interferonopaties. It may be an important tool to select pts to be genetically screened with a defined panel of interferonopathies correlated genes. In pts in which the genetic analysis results negative, the presence of a positive IFN-score, may guide clinicians in the management of these patients and may support therapeutic decisions

**Disclosure of Interest**

None Declared

**Table 1 (abstract P1024).** See text for description

Pts	1	2	3	4	5	6	7	8	910
Systemic symptoms	Fever		Fever			Fever	Fever	Fever	FeverFever, Hemophagocytic lymphohistiocytosis
CNS Cerebral calcification		Epilepsy	Epilepsy, mental retardation		/			Hemiplegia	
Gastrointestinal	Epatosplenomegaly, aspecific IBD			IBD simil-RCU	Abdominal pain, diarrhoea		Abdominal pain	/	Hypertransaminasemia
Skin/osteoarticular	Arthro-myalgia, , panniculitis	Arthro-myalgia, arthritis, urticarial rash	Arthro-myalgia, vasculitis	Eczema	Arthro-myalgia	erythema polymorphe, HSP	Arthro-myalgia	/	9783 28 16
C3 (90-180 mg/dl)	144	127	81	133	53	137	96	203	
C4 (10-40 mg/dl)	34	24	6	23	20	54	17	38	
Immunodeficit autoimmunity		ANA 1:2.560 Anti ds-DNA 1:640	ANA 1:10.240 Anti dsDNA 1:1280	Immunodeficit ANA neg	Neg		Neg	Neg	Immuno Deficit /
WBC/ mmc^3^	5,2	4,4	9,8	3,9	4,6	8,1	6,4	4,4	3,51,7
Hb g/dl	10,4	12	12,9	10,2	13	11.1	14,1	12,1	16,5 9,7
PLT/mmc^3^	199	381	194	86	110	277	214	358	150 56
IS	14,2	171,5	54,73	60,09	27,8	17,2	53,9	40,6	*32,618,19*
Genetic analysis	Neg	*PSMB9:*p.Arg60Cys *OTULIN:*p.Gln115His	*CARD8:*p.Leu426Phe	*IFIH1*: p.Arg374His			Neg		*ACP5:DNASE2*: p.Arg269p.Ala45Gly Tr

#### P1025 Lesson from eurofever registry after the first ten years of enrollment

##### Martina Finetti, Ilaria Gueli, Joost Frenkel, Seza Ozen, HelenLachmann, Fabrizio De Benedetti, Isabelle Koné-Paut, Carine Wouters, Paul Brogan, Hermann Girschick, Benedicte Neven, Alberto Martini, Nicola Ruperto, Marco Gattorno, on behalf of the Paediatric Rheumatology International Trials Organisation (PRINTO) and the Eurofever Project

###### UOSD Centro Malattie Autoinfiammatorie e Immunodeficienze, on the behalf of the Paediatric Rheumatology International Trials Organisation (PRINTO) and the Eurofever Project, IRCCS Istituto Giannina Gaslini, Genoa, Italy

####### **Correspondence:** Martina Finetti

**Introduction:** In 2008 the Paediatric Rheumatology European Society (PReS) promoted an International Project for the study of Autoinflammatory Diseases (AIDs) named Eurofever, whose main purpose is to create a web-based registry for the collection of information in AIDs patients.

**Objectives:** To assess the impact of the Eurofever Registry on scientific community with particular interest in the geographical coverage, diagnostic delay, access to treatment and pubblications.

**Methods:** The data analyzed in the study were extracted from the Eurofever registry, which is hosted in the PRINTO website.

**Results:** Up to date 4175 patients have been enrolled from 62 countries (3843 of them with complete demographic data). Most of patients (72%) are resident in Western Europe, 8% in Central-Eastern Europe, 11% in Southern-Eastern Mediterranean, 2% in South America and 7% in other countries. Compared to the first Eurofever report (Toplak et al, 2012) we have observed an increase of enrolled patients from 1388 to 2651 in Western Europe, from 106 to 313 in Central-Eastern Europe, from 294 to 406 in Southern-Eastern Mediterranean. The median onset age is 4 years (range 1 month – 75 years), the median diagnosis age is 8 years (range 1 month – 78 years). The median diagnostic delay observed in 2012 was 7.3 years (range 0.3–76), from patients enrolled after 2012 it was 1.9 years (range 0-57). Comparing the mean diagnostic delay from 1980 to 2018, we have observed an encouraging constant reduction of period between AIDs onset e diagnosis (from a mean diagnostic delay value of 20 years for patients born before 1980, to a mean value of 1 year for patients born after 2011). Complete information on access to treatment were available in 2430 patients. DMARDs were used in 1031 (42%), biologics in 396 (16%) patients. According to the number of enrolled patients, biologics were used in 361/1782 (20%) of Western europeans, 17/342 (5%) of Central-Eastern europeans, 6/259 (2%) of Southern-Eastern Mediterranean patients. Regarding Eurofever impact on Scientific Community, during this first 10 years the Registry provided 12 papers with more than 800 citations. Detailed analysis of clinical features collected in Eurofever database allowed to perform studies with large cohort of patients, to purpose new classification criteria (Federici et al, 2015), to validate damage and activity score (Piram et al, 2014 and N Ter Haar et al, 2017) and to evaluated genotype/phenotype correlation (Papa et al, 2017).

**Conclusion:** In the last years we have observed an encouraging increase of involved Countries, with a greater number of patients coming from geographic area poorly represented in the first epidemiologic study of Toplak et al. Eurofever data analysis has confirmed an improvement of diagnostic ability during the last years, with a significant reduction of mean diagnostic delay. Long-term studies will help understand the efficacy and safety of different treatments used in these rare conditions.

**Disclosure of Interest**

M. Finetti: None Declared, I. Gueli: None Declared, J. Frenkel: None Declared, S. Ozen: None Declared, H. Lachmann: None Declared, F. De Benedetti: None Declared, I. Koné-Paut: None Declared, C. Wouters: None Declared, P. Brogan: None Declared, H. Girschick: None Declared, B. Neven: None Declared, A. Martini Grant / Research Support from: The Gaslini Hospital, which is the public Hospital where AM worked till 31/dec/2018as a full time public employee, has received contributions from the following industries:Abbvie, Ablynx, Aim Group, Amgen, Astrazeneca, Biogen, BMS, Boehringer, Celgene, Emd Serono, GSK, Janssen, Novartis, Pfizer, R-Pharm. This money has been reinvested for the research activities of the hospital in a fully independent manner without any commitment with third parties., N. Ruperto Grant / Research Support from: The Gaslini Hospital, where NR works as full-time public employee, has received contributions (> 10.000 USD each) from the following industries in the last 3 years: BMS, Eli-Lilly, GlaxoSmithKline, F Hoffmann-La Roche, Janssen, Novartis, Pfizer, Sobi. This funding has been reinvested for the research activities of the hospital in a fully independent manner, without any commitment with third parties.,Speaker Bureau of: NR has received honoraria for consultancies or speaker bureaus (< 10.000 USD each) from the following pharmaceutical companies in the past 3 years: Ablynx, AbbVie, Astrazeneca-Medimmune, Biogen, Boehringer, Bristol Myers and Squibb, Eli-Lilly, EMD Serono, Glaxo Smith and Kline, Hoffmann-La Roche, Janssen, Merck, Novartis, Pfizer, R-Pharma, SanofiServier, Sinergie, Sobi and Takeda., M. Gattorno Grant / Research Support from: MG has received unrestricted grants from Sobi and Novartis

#### P1026 Clinical study of Japanese patients with fever of unknown origin: investigation of mutations in 22 genes related to autoinflammatory diseases

##### Kyoko Fujimoto^1^, Yukiko Hidaka^1^, Yumi Yoshida^1^, Makiko Hayashi^1^, Takuma Koga^1^, Shinjiro Kaieda^1^, Satoshi Yamasaki^2^, Tomoaki Hoshino^1^, Hiroaki Ida^1^

###### ^1^Division of Respirology, Neurology and Rheumatology, Department of Medicine, Kurume University School of Medicine; ^2^Center for Rheumatology, Kurume University Medical Center, Kurume-shi, Fukuoka, Japan

####### **Correspondence:** Kyoko Fujimoto

**Introduction:** Autoinflammatory diseases cause systemic inflammation mainly by innate immune abnormality. It is important to have a diagnosis of autoinflammatory diseases in patients with fever of unknown origin. Examining gene mutations is valuable for the diagnosis of autoinflammatory diseases; however, the frequency of genetic mutations in patients with fever of unknown origin is not reported in Japan.

**Objectives:** We comprehensively analyzed genetic mutations related to autoinflammatory diseases and clinical features in patients with fever of unknown origin.

**Methods:** We analyzed mutations of 22 genes related to autoinflammatory diseases as follows: *TNFRSF1A, MEFV, NLRP3, MVK, NOD2, IL1RN, NLRP12, PSTPIP1, PSMB8, PSMB9, PSMA3, PSMB4, POMP, NLRC4, PLCG2, HMOX1, CECR1, COPA, TNFAIP3, FAM105B, RNF31, RBCK1,*in 84 patients with fever of unknown origin who introduced to our hospital from May 2017 to June 2018. Genetic analysis was performed by the next generation sequencer. Furthermore, we investigated precise clinical features in 53 cases.

**Results:** Fifteen genes were identified as having novel or rare variants. The most frequent variants were determined in *NLRP12* (8 cases, 6 sites) and *NLRP3* (8 cases, 4 sites). In addition, missense mutations including genetic polymorphisms were observed in *MEFV*(66.7%). In 53 cases in which clinical symptomatic investigations were possible, 40 cases (75.5%) had definite periodic fever. Among cases with periodic fever, joint symptoms were observed in 52.5%, and abdominal symptoms were in 35.0%. Colchicine treatment was effective in 37.5%. Sixty-five percent of patients with periodic fever had *MEFV*mutations, including genetic polymorphisms. Almost half of them had novel or rare mutations in autoinflammatory diseases related genes other than *MEFV*. The most frequent variants were determined in *NLRP3.*

**Conclusion:** Novel or rare variants in *NLRP12*and *NLRP3*were noted in patients with fever of unknown origin. Sixty-five percent of patients with periodic fever had *MEFV*mutations including genetic polymorphism.

**Disclosure of Interest**

None Declared

#### P1027 A case of adenosine deaminase 2 deficiency (DADA2) with an uncommon clinical presentation and response to IV IG

##### Francesca Garbarino^1^, Roberta Caorsi^2^, Stefano Volpi^2^, Alice Grossi^3^, Isabella Ceccherini^3^, Marco Gattorno^2^

###### ^1^Università Degli Studi Di Genova; ^2^Clinica Pediatrica Reumatologica, UOSD Malattie Autoinfiammatorie-Immunodeficienze; ^3^UOC Genetica Medica e UOSD Genetica e Genomica delle Malattie Rare, Ist. Giannina Gaslini, Genova, Italy

####### **Correspondence:** Francesca Garbarino

**Introduction:** DADA2 is an autoinflammatory disease with autosomal recessive inheritance characterized by a heterogeneous clinical phenotype ranging from multisystemic inflammation (fever, polyarteritis nodosa, cerebral stroke, livedo reticularis, gastro-intestinal involvement, peripheral neuropathy etc.) to immune-dysregulation and immunodeficiency (lymphoproliferation, recurrent infections, cytopenia etc.).

**Objectives:** To extend the clinical spectrum of DADA2 reporting a case of isolated nonspecific systemic inflammatory syndrome associated with slight signs of immune-dysregulation in a patient with a novel ADA2 mutation.

**Methods:** In a patient with nonspecific inflammatory phenotype associated to susceptibility to viral infections, Next Generation Sequencing (NGS) panel was performed; mutations detected were confirmed by direct sequencing (Sanger analysis). ADA2 enzymatic activity was analyzed in monocyte isolated from the patient and incubated with adenosine and an ADA1 inhibitor.

**Results:** The girl, adopted and of Asian origin, began to suffer from nonspecific systemic inflammatory symptoms (high persistent fever and arthralgias) at the age of 6 years. In past history recurrent respiratory infections and impaired immunological response to viruses (CMV related hepatitis, measles after vaccination) were reported. After few months the patient developed clinical and laboratory findings of HLH (Hemophagocytic Lympho-Histocytosis), confirmed on bone marrow samples; treatment with intravenous (IV) high dose (HD) steroids was started, with prompt response. During steroids tapering fever and systemic inflammation reappeared; anti-IL1 treatment (anakinra) was not effective. Immunologic assessment demonstrated mild hypogammaglobulinemia and moderate NK deficiency on lymphocyte subsets. HD IV Immunoglobulins (IG) (2 g/kg every month) allowed to achieve a complete control of the clinical picture; the frequency of administration was progressively reduced to every 4 months due to persistent wellbeing. At the age of 9, after switching IG to the substitutive dosage, the patient experienced an Herpes Zoster virus reactivation (requiring prolonged antiviral treatment), followed by the reappearance of the inflammatory phenotype complicated by HLH with neurological involvement (irritability and lethargy), responsive to HD steroids and IG. A later cerebral MRI evidenced a small gliotic area in left Centrum Ovale. After steroids suspension, monthly HD IV IG administrations maintained clinical remission. Further immunological studies confirmed a reduction of NK cells with normal function. Hereditary HLH, Autoimmune Lympho-Proliferative Syndrome (ALPS) and main primary immunodeficiencies were ruled out. Given the clinical picture, a large NGS diagnostic panel (courtesy by K. Botzug, Vienna) for autoinflammatory diseases and immunodeficiencies was performed revealing the homozygous LEU141PRO ADA2 mutation, confirmed by Sanger analysis. Being this mutation novel, an ADA2 enzymatic activity test was performed revealing a complete loss of ADA2 activity. The parents refused anti-TNF treatment and the patient is still on monthly HD IG with a complete wellbeing after 3 years of follow-up.

**Conclusion:** The current report enlarges the clinical spectrum associated with DADA2 to a persistent unspecific inflammatory syndrome, complicated by HLH. This case further emphasizes the possibility that NGS could unravel unusual phenotypes of already known inflammatory syndromes. Even if further reports are required, the response to high doses IG observed in the present case it is of interest. Even if anti-TNF is the treatment of choice HD IV IG could be a possible treatment in DADA2, especially during the acute phase.

**Consent for publication has been obtained from patient**

Yes

**Disclosure of Interest**

None Declared

#### P1028 Non-amyloid kidney diseases and autoinflammatory diseases: report of 20 cases and literature review

##### Charlotte Borocco^1,2^, Isabelle Kone-Paut^1,2^, Alexandre Belot^3^, Marine Desjonqueres^3^, Alexandre Karras^4^, Corinne Miceli-Richard^5^, Bruno Moulin^6^, Tim Ulinski^7^, Jean-Jacques Boffa^8^, David Buob^9^, Gilles Grateau^10,11^, Sophie Georgin-Lavialle^10,11^

###### ^1^Pediatric Rheumatology, Bicetre University Hospital; ^2^CeReMAIA, Le Kremlin-Bicêtre; ^3^Pediatric Rheumatology, Lyon University Hospital, Bron; ^4^Department of Nephrology, Georges Pompidou European Hospital; ^5^Department of Rheumatology, Cochin University Hospital, Paris; ^6^Department of Nephrology, Civil Hospital, Strasbourg; ^7^Pediatric Nephrology, Trousseau University Hospital; ^8^Department of Nephrology; ^9^Department of Pathology; ^10^Department of Internal Medicine, Tenon University Hospital; ^11^CeReMAIA, Paris, France

####### **Correspondence:** Sophie Georgin-Lavialle

**Introduction:** Autoinflammatory diseases (AID) are associated with abnormalities of innate immunity. Patients display recurrent fever and various signs including digestive disorders, cutaneous rashes and joint features. One of the most severe complication of AID is inflammatory amyloidosis (AA) including renal involvement, that can lead to kidney failure. However, other nephropathies exist and can be misdiagnosed.

**Methods:** This study was a retrospective, multicentric French study carried out by medical societies and national reference centers of AID. All patients with non-amyloid kidney diseases (NAKD) occurring at pediatric or adult age with a genetically proven or not AID were included.

**Results:** Twenty patients were included: 13 patients with familial Mediterranean fever (FMF), 3 with mevalonate kinase deficiency (MKD), one with TNF-receptor associated periodic syndrome (TRAPS) and 3 with unclassified AID (uAID). Among the 13 FMF patients, 8 displayed homozygous M694V *MEFV* mutation, the mean age at the occurrence of nephrological symptoms was 28.6 years old. Renal diagnosis were: Henoch-Schönlein purpura (HSP) nephritis (n=3), minimal change disease (n=3), IgA nephropathy (n=2), diabetic nephropathy (n=2), nephroangiosclerosis (n=2) and idiopathic nephrotic syndrome (n=1). Renal treatments were represented by angiotensin-converting enzyme inhibitors (ACEIs) (n=6), then colchicine (n=4; dose adjustment or reinstatement of treatment), corticosteroids (n=4), antihypertensive medication (n=4), dialysis (n=2), levamisol (n=1), kidney transplantation (n=1) and anakinra (n=1). After treatment, 5 patients were in renal remission, 2 patients had a transient proteinuria, 5 patients a persistent proteinuria, 2 patients had a renal insufficiency, and 1 patient was in remission after kidney transplantation.

Among the 3 MKD patients, the mean age at onset of nephrological symptoms was 17.7 years. Renal biopsies found: pauci immune extra capillary glomerulonephritis (n=1), HSP nephritis (n=1) and segmental focal hyalinosis lesions associated with nephrosclerosis (n=1). One patient received a kidney transplant with a relapse after it, one was in pre-transplant status, one was in remission under treatment. They all were treated with anti IL1 agent.

The TRAPS patient displayed rapidly progressive glomerulonephritis and was treated with steroids, etanercept and then anti IL1 agent.

The 3 uAID patients displayed: C3 depositional glomerulonephritis (n=1) an IgA nephropathy (n=2) at a median age of 36 years old.

In our literature review including our cases, we found 124 cases of FMF with NAKD biopsy proven, 4 MKD patients with NAKD, only 1 TRAPS patient and no cryopyrin associated periodic syndrome.

**Conclusion:** Non-amyloid nephropathies can occur in AID; the most frequent ones being IgA nephropathy and HSP nephropathy. Kidney biopsy is necessary in case of nephrological signs to optimize the treatment and to limit the evolution towards kidney failure.

**Disclosure of Interest**

None Declared

#### P1029 The transition from pediatrics to adulthood in auto-inflammatory diseases: comparison of 2 transition strategies among 72 patients

##### Sophie Georgin-Lavialle^1^, Pierre Quartier^2^, Brigitte Bader-Meunier^2^, Katia Stankovic Stojanovic^3^, Virginie Avellino^3^, Samuel Deshayes^3^, Isabelle Melki^4^, Alexandre Belot^5^, Isabelle Kone-Paut^6^, Gilles Grateau^7^, Véronique Hentgen^8^ on behalf of transition group project, CEREMAIA, FAI2R and Transition Working Group, National Reference Center for Autoinflammatory Diseases and AA Amyloidosis

###### ^1^Internal Medicine, Tenon hospital, AP-HP, Paris; ^2^Pediatric Rheumatology, AP-HP, Necker Hospital; ^3^Internal Medicine, Tenon Hospital; ^4^Pediatric Rheumatology, AP-HP, PARIS; ^5^Pediatric Rheumatology, HCL, Hopital Mère Enfant, LYON; ^6^Pediatric Rheumatology, AP-HP, Kremlin-Bicêtre hospital, Kremlin-Bicêtre; ^7^Internal Medicine, AP-HP, Tenon hospital, PARIS; ^8^General pediatry, CH Versailles André mignot, Versailles, France

####### **Correspondence:** Sophie Georgin-Lavialle

**Introduction:** Transition is the period during which the young patient will move from the pediatric team to the adult team for the follow-up of his or her chronic disease. It requires perfect coordination between the two medical teams but also the active involvement of the young person and his or her family. A specific transition program increases the chances of good health and well-being and reduces mortality in this age group. We experienced two different transition strategies from two different pediatric wards to a single adult ward specialized in auto-inflammatory diseases (AIDs) and compare the efficacy of the 2 strategies.

**Objectives:**To compare the effectiveness of the transition process in the two strategies, evaluated by the lost of sight patients avec a median 5 years of follow up.

**Methods:** Children from 2 pediatric centers were addressed for transition in a single adult ward. The first strategy concerned 53 patients that were to prepare since the age of 14 years old and at the age of 18, they were presented for transition thru a joint consultation with the pediatrician in the adult clinics. The second strategy concerned 19 patients who came directly from pediatrics without the pediatrician for an adult medical consultation; the pediatrician explained bye mail and/or phone the case and sent the patient with his medical file.

**Results:** Seventy-two young people (29 girls, 43 boys) passed the 10-year transition step between 2007 and 2017. The AIDs encountered were: familial Mediterranean fever (n=46), PFAPA syndrome, mevalonate kinase deficiency (n=6 of each), Still's disease (n=4), TRAPS syndrome (n=3) or another rare MAI (NRLP12 mutation, CAPS, or other rare) (1 to 2 cases each time). A total of 81% young patients came regularly (about once a year). Only 3 patients are currently out of sight (4%); none had a clearly identified single gene MAI requiring very regular follow-up. The young people interviewed are generally satisfied.

**Conclusion:** No difference was observed in the transition efficacy process when comparing the two transition strategies (joint consultation or not). Joint consultation is experienced as necessary, but our results show that this strategy is not mandatory for a successful transition. When asked, young people are eager to have written documentation about their illness and to have the telephone number and e-mail address of the doctor to contact in case of problems.

These positive results of the effectiveness of a transition program require close collaboration between the pediatrician and the adult physician regardless of the transition strategy chosen. The adult doctor must invest in taking care of young people who are very connected and in case of problems expect a quick response by email most often. Indeed, more than a joint consultation, the involvement of the adult physician seems essential to the successful transition from pediatrics to adult service.

**Disclosure of Interest**

None Declared

#### P1030 The longitudinal eurofever project: an update on enrollment

##### Ilaria Gueli, Martina Finetti, Fabrizio De Benedetti, Jordi Anton Lopez, Maria Alessio, Joost Frenkel, Luca Cantarini, Romina Gallizzi, Judith Sanchez Manubens, Marco Cattalini, Efimia Papadopoulou-Alataki, Rolando Cimaz, Donato Rigante, Alma Nunzia Olivieri, Pavla Dolezalova, Alberto Martini, Nicola Ruperto, Marco Gattorno, on behalf of the Paediatric Rheumatology International Trials Organisation (PRINTO) and the Eurofever Project

###### UOSD Centro Malattie Autoinfiammatorie e Immunodeficienze, on the behalf of the Paediatric Rheumatology International Trials Organisation (PRINTO) and the Eurofever Project, IRCCS Istituto Giannina Gaslini, Genoa, Italy

####### **Correspondence:** Ilaria Gueli

**Introduction:** In 2008 the Paediatric Rheumatology European Society (PReS) promoted an International Project for the study of Autoinflammatory Diseases (AIDs) named Eurofever, whose main purpose is to create a web-based registry for the collection of information in AIDs patients

**Objectives:** To implement the Registry with the new recently described AIDs and to increase the collection of longitudinal data

**Methods:** The data were extracted from the Eurofever registry, which is hosted in the PRINTO website (http://www. printo.it). From February 2015 we started the longitudinal collection of follow-up data with particular focus on treatment, modification of the clinical picture, onset of complication/adverse events. We have enrolled patients included in the registry up to 28 September 2018.

**Results:** Up to date 4175 patients have been enrolled (3843 of them with complete demographic information, 1903 M and 1940 F) from 62 countries. For 3356 (87%) patients also clinical data from onset to diagnosis, collected during the first visit performed at referred pediatric or adult center, are available. For each disease the number of enrolled patients is: FMF 1086 pts (951 with complete clinical data); TRAPS 273 pts (256 complete); CAPS 298 pts (279 complete); MKD 205 pts (190 complete); Blau’s disease 49 pts (26 complete); PAPA 42 pts (41 complete); NLRP-12 mediated periodic fever 13 pts (11 complete); DADA2 14 pts (9 complete); DIRA 3 pts (all complete); SAVI 3 pts (all complete); CANDLE 1 pt (complete) and Majeed 4 pts (all complete). Among multifactorial autoinflammatory diseases: PFAPA 676 pts (551 complete); CNO 581 pts (540 complete); Behcet 214 pts (186 complete), undefined periodic fever 368 pts (292 complete) and Schnitzler syndrome 13 pts (all complete). The median onset age is 4 years (range 1 month – 75 years), the median diagnosis age is 8 years (range 1 month – 78 years). Most of patients (3509, 91%) presented disease onset during pediatric age (<16 years), 334 (9%) during adult age (81 FMF, 31 CAPS, 53 TRAPS, 40 CRMO, 12 Schnitzler syndrome and 90 unknown fever). 405 of 3509 (12%) patients with pediatric onset received diagnosis during adult age. The median diagnostic delay is 5 years; diseases with longer diagnostic delay are: NLRP12 (24 years, range 4-76), CAPS (15 years, range 0-77), PAPA (14 years, range 2-57), TRAPS (12 years, range 0-77). 396 patients have been treated with at least one biologic drug, 1031 with DMARDs, 427 with systemic steroid and 686 with others drugs. The most frequent diseases treated with biologic drugs are: CAPS (38%), multifactorial diseases (22%), TRAPS (14%), MKD (11%), rare monogenic (8%: 1 CANDLE, 2 DIRA, 2 NLRP12, 3 Majeed, 8 DADA2 and 14 PAPA), and FMF (7%). Since February 2015, longitudinal visits have been inserted for 477 (12%) patients, with detailed data on treatment and safety.

**Conclusion:** The enrollment in Eurofever Registry is still ongoing. The analysis of data will improve our knowledge both on the natural history of the single disease and on the efficacy/safety of treatment commonly used in the clinical practice.

**Disclosure of Interest**

I. Gueli: None Declared, M. Finetti: None Declared, F. De Benedetti: None Declared, J. Anton Lopez: None Declared, M. Alessio: None Declared, J. Frenkel: None Declared, L. Cantarini: None Declared, R. Gallizzi: None Declared, J. Sanchez Manubens: None Declared, M. Cattalini: None Declared, E. Papadopoulou-Alataki: None Declared, R. Cimaz: None Declared, D. Rigante: None Declared, A. N. Olivieri: None Declared, P. Dolezalova: None Declared, A. Martini Grant / Research Support from: The Gaslini Hospital, which is the public Hospital where AM worked till 31/dec/2018as a full time public employee, has received contributions from the following industries:Abbvie, Ablynx, Aim Group, Amgen, Astrazeneca, Biogen, BMS, Boehringer, Celgene, Emd Serono, GSK, Janssen, Novartis, Pfizer, R-Pharm. This money has been reinvested for the research activities of the hospital in a fully independent manner without any commitment with third parties., N. Ruperto Grant / Research Support from: The Gaslini Hospital, where NR works as full-time public employee, has received contributions (> 10.000 USD each) from the following industries in the last 3 years: BMS, Eli-Lilly, GlaxoSmithKline, F Hoffmann-La Roche, Janssen, Novartis, Pfizer, Sobi. This funding has been reinvested for the research activities of the hospital in a fully independent manner, without any commitment with third parties.,Speaker Bureau of: NR has received honoraria for consultancies or speaker bureaus (< 10.000 USD each) from the following pharmaceutical companies in the past 3 years: Ablynx, AbbVie, Astrazeneca-Medimmune, Biogen, Boehringer, Bristol Myers and Squibb, Eli-Lilly, EMD Serono, Glaxo Smith and Kline, Hoffmann-La Roche, Janssen, Merck, Novartis, Pfizer, R-Pharma, SanofiServier, Sinergie, Sobi and Takeda., M. Gattorno Grant / Research Support from: MG has received unrestricted grants from Sobi and Novartis

#### P1031 Digital brachial index testing as a noninvasive tool in diagnosing peripheral vascular disease in DADA2

##### Patrycja M. Hoffmann^1^, Deborah L. Stone^1^, Karyl Barron^2^, Cornelia Cudrici^3^, Alessandra Brofferio^3^, Anne Jones^1^, Tina Romeo^1^, Ivona Aksentijevich^1^, Daniel L. Kastner^1^, Amanda K. Ombrello^1^

###### ^1^NHGRI; ^2^NIAID; ^3^NHLBI, National Institutes of Health, Bethesda, MD, United States

####### **Correspondence:** Patrycja M. Hoffmann

**Introduction:** Deficiency of adenosine deaminase 2 (DADA2), is an autosomal recessive disease caused by biallelic loss-of-function mutations in the *ADA2* gene. Deficiency of ADA2 is the first molecularly described monogenic vasculitis syndrome. In some cases, DADA2-associated peripheral vascular disease (PVD) can be severe enough to require amputation.

**Objectives:** To examine the results of upper and lower digital brachial index (DBI) and to compare upper and lower DBI tests to corresponding upper and/or lower extremity MRA tests in patients with DADA2 who have clinical features of PVD.

**Methods:** Three out of 45 patients with DADA2 seen at the NIH were found to have moderate to severe PVD. Complete histories were obtained and physical exams were performed. Upper and/or lower extremity DBI tests and MRA exams were performed.

**Results:** *28 yo male with DADA2 who had triphasic Raynaud’s syndrome and pustular-like lesions, bone resorption, and necrotic tissue of various digits, three of which eventually required partial amputation.* DBI of upper and lower extremities showed decreased waveform and pressure in 1^st^-5^th^ left upper digits, 1^st^, 3^rd^-5^th^ right upper digits, 2^nd^-5^th^ left lower digits and 2^nd^-5^th^ right lower digits. MRA findings showed a single patent common artery, supplying blood to 2^nd^-3^rd^ left upper digits, adequate blood flow in right upper 2^nd^ digit and little to no blood flow in right 1^st^, 3^rd^-5^th^ digits. Right and left lower 1^st^ digit blood flow was patent. There were numerous collaterals to all lower extremity digits. Thus DBI and angiography were in agreement. DBI results demonstrated a lack of waveform or pressure in digits that showed poor flow on angiography. Based on this data, DBI can be considered as an alternative to MRA if cost or renal insufficiency is a concern.

*26 yo female with DADA2 with polyarteritis nodosum and intermittent bilateral 1*^*st*^ *toe swelling and pain.* Right and left lower DBI showed no waveform or pressure in 1^st^-5^th^ digits. MRA of lower extremity was limited to medium vessel arteries which were patent bilaterally. Small vessels of the feet were not well visualized because of MRA limitations so digital blood flow could not be analyzed or compared to abnormal DBI results.

*39 yo female with DADA2 with multiple chronic ankle ulcerations and Raynoud’s syndrome.* Bilateral lower DBI waveform and pressure was normal in the 1^st^ digits bilaterally but there was decreased pressure and waveform in the 2^nd^-5^th^ digits bilaterally. MRA of lower extremity was limited to medium vessel arteries which were patent bilaterally. Small vessels were not well visualized because of MRA limitations so digital blood flow could not be analyzed or compared to abnormal DBI results.

**Conclusion:** The complexity of DADA2 increases the need for expanding diagnostic tests, some of which can be long and invasive. In the NIH cohort of 45 patients, 3 were found to have clinical findings of PVD. The upper and lower extremity DBI test helped diagnose patients with moderate to severe PVD. In our 2^nd^ and 3^rd^ patient, the MRA exam was limited to medium vessel blood flow testing and therefore blood flow in the small vessels of the feet were not well visualized.Based on the noninvasive less expensive DBI diagnostic testing, patients with DADA2 can be screened effectivey and in a timely manner and avoid exposure to contrast, especially in the setting of renal deficiency. The additional close monitoring can help expedite diagnosis and treatment to possibly avoid future amputation as was the case in Patient 1.

**Consent for publication has been obtained from patient**

Yes

**Disclosure of Interest**

None Declared

#### P1032 The expanding clinical and laboratory spectrum of PAPA syndrome: the NIH cohort

##### Patrycja Hoffmann^1^, Amanda K. Ombrello^1^, Deborah L. Stone^1^, Karyl Barron^2^, Anne Jones^1^, Tina Romeo^1^, Michele Nehrebecky^1^, Jae Chae^1^, Ivona Aksentijevich^1^, Daniel L. Kastner^1^

###### ^1^NHGRI; ^2^NIAID, National Institutes of Health, Bethesda, MD, United States

####### **Correspondence:** Patrycja Hoffmann

**Introduction:** The dominantly inherited PAPA syndrome is caused by mutations in *PSTPIP1*. It is one of the least understood of the known monogenic autoinflammatory diseases, both from a pathogenic and treatment perspective. Symptoms include arthritis, cystic acne, and pyoderma gangrenosum. Therapy includes corticosteroids, interleukin-1 receptor antagonists, and tumor necrosis factor inhibitors.

**Objectives:** To review noninfectious epiglottis and sterile osteomyelitis, rare complications not previously published in PAPA syndrome, and to compare LDH and aldolase levels in patients with PAPA syndrome vs patients with mutation-negative PAPA-like phenotype.

**Methods:** The NIH cohort of 20 patients with PAPA syndrome and 10 patients with PAPA-like phenotype were reviewed. Comprehensive evaluation was performed. MRI, CBC with differential, LDH, aldolase, CK, CRP and ESR were examined. Patients’ labs were drawn during times of flare and no flare. CK was drawn to ensure there was no inflammatory muscle involvement.

**Results:** One patient with PAPA syndrome developed severe sore throat. Clinical and radiographic evaluation showed piriform sinus swelling consistent with supraglottitis. CRP and ESR were elevated. WBC was normal. Treatment was started with IV clindamycin and ceftriaxone; doxycycline was added later. Severe sore throat continued and repeat radiographic findings showed ongoing swelling. Clindamycin was stopped.Methylprednisolone and a scheduled dose of golimumab were initiated with significant symptomatic improvement and normalization of radiographic findings, CRP and ESR.

A second patient with PAPA syndrome developed severe right wrist pain and swelling. MRI revealed distal right radial epiphyseal marrow abnormalities consistent with osteomyelitis without synovitis. ESR and CRP were elevated. WBC was normal. The patient was treated with clindamycin without symptomatic benefit or improvement in MRI findings. Clindamycin was discontinued. Methylprednisolone and anakinra were initiated which resulted in decreased pain and improvement in marrow inflammation on MRI. CRP and ESR improved.

Of the 20 patients with PAPA syndrome, LDH, aldolase, and CK levels were drawn. LDH was elevated in 19/20 patients. Aldolase was elevated in 19/19 patients. CK was elevated in 5/20 patients. Of the 10 patients with PAPA-like phenotype, LDH was elevated in 1/10 patients. Aldolase was elevated in 2/10. CK was elevated in 1/10. MRIs done on two patients with PAPA syndrome did not show muscle inflammation during a flare.

Upon further analysis of LDH isoenzymes I-V tested in 12/20 patients with PAPA syndrome, 12/12 had elevated LDH isoenzyme V, a skeletal muscle isoenzyme.

**Conclusion:** Our findings indicate an expanding clinical spectrum in PAPA syndrome that includes aseptic supraglottitis and non-bacterial osteomyelitis. Improvement in clinical symptoms, MRI findings, and acute phase reactants as well as a normal WBC on methylprednisolone support an inflammatory rather than infectious process.

We do not have a clear understanding as to the relevance of elevation in LDH and aldolase in patients with PAPA syndrome and overall normal LDH and aldolase in the PAPA-like phenotype during periods of flare and no flare. LDH isoenzyme V and aldolase are markers of skeletal muscle involvement. In looking at muscle MRIs and obtaining CK levels, we did not see any muscle inflammation. Therefore, in addition to pursuing further testing to rule out muscle damage, additional etiologies for elevation of LDH and aldolase should be considered.

**Disclosure of Interest**

None Declared

#### P1033 Pseudodominant inheritance of Behçet-like autoinflammatory disease associated with TNFAIP3 (A20) and MEFV mutations in a Turkish family with familial Mediterranean fever

##### Nobuyuki Horita^1^, Ahmet Gul^2^, Ivona Aksentijevich^1^, Daniel Kastner^1^, Elaine Remmers^1^

###### ^1^NIH, Bethesda, United States; ^2^Istanbul University, Istanbul, Turkey

####### **Correspondence:** Nobuyuki Horita

**Introduction:** Familial Mediterranean Fever (FMF) is an auto-inflammatory disorder that causes recurrent fevers and painful serosal inflammation in the abdomen and pleura as well as synovial inflammation in the joints. Missense M694V, V726A, M694I, M680I and other mutations in exon 10 of the MEFV gene are well known to cause FMF, usually with a recessive mode of inheritance. The MEFV gene encodes pyrin, a key component of the pyrin inflammasome. FMF-associated mutations in pyrin cause dysregulated activation of the inflammasome, leading to activation of caspase, which converts pro-interleukin-1 (IL-1) beta to its active cleaved form.

**Objectives:** We obtained a Turkish kindred with multiple cases with Familial Mediterranean Fever (FMF) and Behçet's disease (BD)-like manifestations. The index case and her two daughters, all with Behçet-like disease, were previously found to have a TNFAIP3 frameshift mutation. The high frequency of affecteds could be consistent with a dominantly inherited inflammatory disease in this family, although other individuals had clinical features consistent with recessively inherited FMF. We sequenced DNA from members of this family to determine whether the TNFAIP3 frameshift mutation and MEFV variants could explain this autoinflammatory disease pedigree.

**Methods:** Patients were clinically diagnosed to have FMF or BD. Sanger sequence targeting TNFAIP3 exon 5 and MEFV exon 10 was carried out.

**Results:** A maternal uncle of the index case and the mother of the index case had compound heterozygous FMF-associated MEFV exon 10 mutations, M680I and R761H. Two daughters of the maternal uncle also had compound heterozygous FMF-associated MEFV mutations, M680I and V726A. The index case and her two daughters had a TNFAIP3 exon 5 frameshift mutation (TNFAIP3 c.799delG, p.Pro268Leufs*19), which is consistent with their HA-20 diagnosis, and also carried a single allele (heterozygous) of the MEFV R761H mutation.

**Conclusion:** Segregation of BD-like manifestations in a single Turkish family with multiple FMF patients could be explained by co-inheritance of a heterozygous exon 10 MEFV variant and one TNFAIP3 mutation, and contribution of MEFV variants on the BD-like manifestations of HA20 requires further studies. 

**Consent for publication has been obtained from patient**

Yes

**Disclosure of Interest**

None Declared

#### P1034 Systematic review of biological treatment of deficiency of interleukin-36 receptor antagonist (DITRA) in children and adolescents

##### Toni Hospach^1^, Fabian Glowatzki^1^, Friederike Blankenburg^1^, Dennis Conzelmann^1^, Christian Stirnkorb^1^, Sandra Müllerschön^2^, Peter von den Driesch^2^, Lisa Koehler^3^, Meino Rohlfs^3^, Christoph Klein^3^, Fabian Hauck^3^

###### ^1^Olgahospital Stuttgart; ^2^Klinikum Stuttgart, Stuttgart; ^3^Dr. von Haunersches Kinderspital, Universität München, München, Germany

####### **Correspondence:** Toni Hospach

**Introduction:** Deficiency of interleukin-36 receptor antagonist (DITRA) is a life threatening autoinflammatory disease caused by autosomal recessive mutations of the IL36RN gene leading to recurrent episodes of generalized pustular psoriasis with systemic inflammation and fever. For this disease no standardized treatment guidelines do exist.

**Objectives:** To systematically review and analyze the data of biologically treated pediatric DITRA patients.

**Methods:** For systematic research we made a “pubmed” research using the term “Deficiency of interleukin-36 receptor antagonist”, “Deficiency of interleukin-36 antagonist”, “IL36RN mutation” and “DITRA” with age restriction to 18 years.

**Results:** Our literature research revealed 13 pediatric patients with DITRA and biolocial treatment. Ten patients were homozygous including six with the p.Leu27Pro, three with the p.Arg10 Argfs* and one with the p.Thr123Met mutation and four were compound heterozygous. We add an unreported DITRA patient with a compound heterozygous *IL36RN* p.Pro76Leu/pSer113Leu mutation. In total 29 flares in 14 patients were treated with biological agents- targeting IL-1/R, IL-17, IL-12/23 and TNF-α. Complete response was achieved in 15 (52%), partial in 4 (14 %), and no response in 10 (34 %) of the flares. Response rates were heterogeneous among the different agents. While complete/partial/no response with inhibition of TNF-alpha could be achieved in 6 (46%)/3 (23%)/4 (31%), the inhibition of IL-17 and of IL-12/23 led in each 4 flares to a 100 % complete response. IL-1/R inhibition led to complete/partial response in each 1 (13 %) and was not effective in 6 (75%) flares. Of note, the unreported patient was successfully treated with weekly dosed adalimumab.

**Conclusion:** DITRA is a rare disease that has to be considered in patients with generalized pustular psoriasiswith systemic inflammation and fever. It can be effectively treated with specific biological inhibition of TNF-alpha, IL-12/23 and IL- 17, while anti-IL-1/R treatment seems less effective. Weekly dosed adalimumab appears to be a novel treatment option for pediatric patients. Further reports and studies of biological treated pediatric DITRA patients are warranted for evaluation of optimal treatment.

**Disclosure of Interest**

None Declared

#### P1035 Familial Mediterranean fever in Slovakia – clinical and genetic characteristics of Slovak cohort

##### Milos Jesenak^1,2,3^, Lenka Kapustova^1^, Katarina Hrubiskova^4^, Tomas Dallos^5^, Peter Banovcin^1^

###### ^1^Centre for Periodic Fever Syndromes, Department of Pediatrics; ^2^Centre for Periodic Fever Syndromes, Department of Pulmonology and Phthisiology, Jessenius Faculty of Medicine, Comenius University in Bratislava; ^3^Department of Clinical Immunology and Allergology, University Teaching Hospital in Martin, Martin; ^4^Centre for Periodic Fever Syndrome, 5th Department of Internal Medicine, Comenius University in Bratislava, Faculty of Medicine, University Teaching Hospital; ^5^Department of Pediatrics, National Institute of Children’s Diseases, Comenius University in Bratislava, Faculty of Medicine, Bratislava, Slovakia

####### **Correspondence:** Milos Jesenak

**Introduction:** Familial Mediterranean fever (FMF) is considered to be a rare disease in the region of Central Europe (CE). True prevalence is still not known and awareness of FMF is very low.

**Objectives:** Since the data about FMF prevalence are missing, we aimed to find all the FMF cases from the whole area of Slovakia. Then we aimed to analyse the clinical and genetic characteristics of Slovakian FMF patients’ cohort.

**Methods:** We performed the questionnaire based survey in outpatient clinics for clinical immunology and rheumatology and hospital departments in Slovakia. We collected all the genetically-confirmed FMF patients and invite them for clinical examination in the National Centre for Periodic Fever Syndromes in Martin. They underwent complex clinical and laboratory examination and detailed history analysis.

**Results:** All together, we detected 53 FMF patients (males 23, 42%), aged 33.64±17.33 years (males 28.93±17.42 years, females 35.39±17.34 years). The age of first symptoms was 12.90±13.37 years and the age of FMF confirmation was 31.30±18.18 years, so the average diagnostic delay was almost 19 years. All the patients had recurrent abdominal pain (100%) which was accompanied by recurrent fever in 50 pts (94.3%). Other reported symptoms associated with FMF were: fatigue (77.4%), arthralgia/arthritis (66.0%), chest pain (56.6%), cervical lymphadenopathy (32.1%), tonsillitis (28.3%), headache (26.4%) and skin rash during flares (15.1%). Pleuritis was confirmed in 18.9%, pericarditis in 11.3% and ascited in 22.6%. 3 patients suffered from renal amyloidosis. 3 pts (5.7%) were homozygotes for pathogenic mutation, 10 (18.9%) compound heterozygotes, 28 (52.8%) with pseudo-AD inheritance and 12 (22.6%) patients carried mutations of unknown or possible benign origin. 79% pts were treated by colchicine (mean dose was 1.03±0.51 mg/day), 10 with anakinra (3 regular, 7 on demand) and 9 by canakinumab. 4 patients were intolerant to colchicine. Isolated elevation of serum amyloid A without concomitant elevation of C-reactive protein between the flares before the initiation of the treatment was observed in 34% pts. In 5 of them (9.4%), the IgD elevation was found. In 7 patients, the origin from FMF endemic regions was confirmed.

**Conclusion:** This the first complex report about the epidemiology of FMF in Central European Country with presumed low incidence. We confirmed much higher prevalence as was expected. The majority of our patients have pseudo-AD form of FMF. All the available therapeutic options were applied. It is necessary to raise the awareness of FMF and to shorten the diagnostic delay.

**Disclosure of Interest**

None Declared

#### P1036 Diagnostic criteria for proteasome-associated autoinflammatory syndromes (PRAASS) including Nakajo-Nishimura syndrome, JMP syndrome and CANDLE syndrome

##### Nobuo Kanazawa^1^, Hiroaki Ida^2^, Noriko Kinjo^3^, Tomoaki Ishikawa^4^, Ryuta Nishikomori^5^

###### ^1^Department of Dermatology, Wakayama Medical University, Wakayama; ^2^Department of Medicine, Division of Respirology, Neurology, and Rheumatology, Kurume University School of Medicine, Kurume; ^3^Department of Pediatrics, University of the Ryukyus Graduate School of Medicine, Nishihara; ^4^Department of Pediatrics, Nara Medical University, Kashihara; ^5^Department of Pediatrics, Kyoto University Graduate School of Medicine, Kyoto, Japan

####### **Correspondence:** Nobuo Kanazawa

**Introduction:** Nakajo-Nishimura syndrome (NNS) was described originally as “secondary osteoperiostosis with pernio” in Japanese by Nakajo in 1939 and by Nishimura in 1950, and then reported in English as “a syndrome with nodular erythema, elongated and thickened fingers, and emaciation” in 1985 and as “hereditary lipo-muscular atrophy with joint contracture, skin eruptions and hyper-gamma-globulinemia” in 1993. It was in 2011 that similar syndromes have first been reported from outside Japan, which included “joint contractures, muscular atrophy, microcytic anemia and panniculitis-induced lipodystrophy (JMP)” syndrome and “chronic atypical neutrophilic dermatitis with lipodystrophy and elevated temperature (CANDLE)” syndrome. As *PSMB8* mutations have commonly been identified in NNS and these two syndromes, they are now collectively called as “proteasome-associated autoinflammatory syndromes (PRAASs)”. Recently, their responsible genes are expanding to other proteasomal genes and, on the other hand, a similar but distinct proteasome-associated entity “proteasome-associated autoinflammation and immunodeficiency disease (PRAID)” has been proposed. In Japan, NNS is now registered as an officially-recognized intractable disease diagnosed by officially-approved criteria.

**Objectives:** To define the diagnostic criteria of PRAASs.

**Methods:** So far reported 30 NNS, 3 JMP and 21 CANDLE/PRAAS cases are reviewed and the temporal diagnostic criteria of NNS are verified for these cases.

**Results:** Among 8 points of 1) autosomal recessive inheritance (parental consanguinity or familial occurrence), 2) pernio-like purplish rash in hands and feet (appearing in winter since infancy), 3) haunting nodular erythema with infiltration and induration (sometimes circumscribed), 4) repetitive spiking fever (periodic, not necessarily), 5) Long clubbed fingers and toes with joint contractures, 6) progressive partial lipomuscular atrophy and emaciation (marked in the upper part of body), 7) hepatosplenomegaly, and 8) basal ganglia calcification, more than 5 are required for temporal clinical diagnosis of NNS. 80% of NNS, 100% of JMP and 67% of CANDLE/PRAAS cases meet these criteria, while most of NNS cases reported before 1990 and CANDLE/PRAAS cases without any description on pernio-like rash do not meet the criteria. If the point 7) is changed to hepatomegaly and 2 points of 9) microcytic anemia and 10) hyper-gamma-globulinemia are added and more than 6 of the final 10 points are required for the diagnosis, positivity of the new criteria reaches 93% of NNS, 100% of JMP and 76% of CANDLE/PRAAS cases. PRAID cases do not meet these criteria. Genetically, 11 cases of NNS, 3 cases of JMP and 11 cases of CANDLE/PRAAS cases have homozygous or compound heterozygous *PSMB8* mutations, while other digenic (*PSMB8* + *PSMA3*, *PSMB8* + *PSMB4*, *PSMB9* + *PSMB4*), compound heterozygous *PSMB4* or heterozygous *POMP* mutations are observed in 8 CANDLE/PRAAS patients. PRAID cases have distinct heterozygous *POMP* or *PSMB9* mutations.

**Conclusion:** We propose a new diagnostic criteria for PRAASs: clinically, at least 6 points are required among above-mentioned 10 points, and genetically, “definite” when disease-associated proteasomal gene mutation(s) are identified and “probable” even if such mutation(s) are not identified, but when other diseases are differentiated.

**Disclosure of Interest**

None Declared

#### P1037 Short term follow-up results of children with familial Mediterranean fever after cessation of colchicine: is it possible to quit?

##### Ayşe Tanatar, Hafize Emine Sönmez, Şerife Gül Karadağ, Mustafa Çakan, Nuray Aktay Ayaz

###### Pediatric Rheumatology, Health Sciences University Kanuni Sultan Süleyman Training and Research Hospital, Istanbul, Turkey

####### **Correspondence:** Şerife Gül Karadağ

**Introduction:** To define the characteristics of children with familial Mediterranean fever (FMF) whose colchicine treatment was discontinued and then to compare these features of the patients whose colchicine was restarted with the ones not restarted.

**Methods:** Sixty-four out of 1786 children with FMF whom colchicine was stopped by the physician or patients/parents own decision were enrolled. These patients were grouped into two as: group 1; children whose colchicine was re-started and group 2; children whose colchicine was not re-started. The demographic, clinical and genetic data were collected and compared between group1 and group 2.

**Results:** Colchicine was stopped in 59.4% (38/64) by the physician and 40.6% (26/64) of them had stopped colchicine by patients/parents will. Colchicine was ceased at a median of 10.6 (2.120.5) years of age, and attack- and inflammation-free periods of 18.2 (6-148) months. The median follow-up of 64 patients after colchicine cessation was 37.4 (6.4-154.7) months. It was re-started in seventeen patients due to attacks (n=11) or elevated acute phase reactants (n=6), while remaining 47 patients did not require colchicine. The age at cessation of the colchicine was lower (p= 0.04) and duration of colchicine treatment until its cessation was shorter (p= 0.007) in group 1 than group 2.

**Conclusion:** Even though the results of our study are not satisfactory enough to endorse the hypothesis that colchicine may be discontinued by close follow-up; older age and long duration of colchicine treatment before cessation may be two important features that should be considered in the future studies

**Disclosure of Interest**

None Declared

#### P1038 Periodic fever syndromes: a year follow-up of a tertiary pediatric rheumatology outpatient clinic in Turkey

##### Nuray Aktay Ayaz, Şerife G. Karadağ, Hafize Emine Sönmez, Ayşe Tanatar

###### Pediatric Rheumatology, Health Sciences University Kanuni Sultan Süleyman Training and Research Hospital, Istanbul, Turkey

####### **Correspondence:** Şerife G. Karadağ

**Introduction:** To define the frequency of patients who were suspected to have periodic fever syndromes (PFS) and their final diagnosis.

**Methods:** We prospectively evaluated the patients who were initially referred to our department with suspicion of PFS in a year period. These findings cover only ten months results as a preliminary study.

**Results:** A total of 2317 new patients (1142 male/1175 female) were seen. Among them, 724 patients were referred to evaluate for the presence of PFS. Finally, 553 patients were classified as having PFS. Of those, 444 patients were diagnosed with familial Mediterranean fever, 43 with periodic fever with aphthous stomatitis, pharyngitis, and adenitis, 2 with cryopyrin-associated periodic fever syndromes, and 1 with hyper-immunoglobulin D syndrome. Genetic analyses are still in progress in the remaining 63 patients. Most common MEFV variant was M694V in our patients. The rest of the patients who were suspected to have PFS were diagnosed as follows: gastrointestinal disorders (n= 161), infections (n=6), dysmenorrhea (n=2), immunodeficiency (n=1).

**Conclusion:** Diagnosing PFS requires a careful evaluation. As our study shows nearly one third of patients referred to our center were not accepted as PFS.A detailed evaluation of the patient’s signs and symptoms and also taking the recurrency of these features into account will help to exclude unnecessary referrals to pediatric rheumatology units. Although recommendations are present for rheumatologists, generating some clear-cut algorithms about PFS may provide a practical approach for general pediatricians while they are evaluating and referring these patients.

**Disclosure of Interest**

None Declared

#### P1039 Assessing French liberal pediatricians awareness and referral for reccurent fever syndromes: the fireville survey

##### Valerian Koskas^1^, Remy Assathiany^2^, Sylvie Hubinois^3^, Corinne Levy^4^, Marc Koskas^5^, Isabelle Koné-Paut^6^

###### ^1^Pediatric Rheumatology, APHP, University of Paris Sud, Le Kremlin Bicêtre; ^2^Pediatrics, Liberal Exercise, Issy les Moulineaux; ^3^AFPA (Association Française de Pédiatrie Ambulatoire), Saint Germain en Laye; ^4^Association Clinique et Thérapeutique Infantile du Val de Marne; ^5^Liberal Pediatrician, Saint Maur; ^6^Pediatric rheumatology, APHP, Bicetre Hospital, Le Kremlin Bicêtre, France

####### **Correspondence:** Valerian Koskas

**Introduction:** Patients with recurrent fevers almost always refer initially to their family (liberal) paediatrician, their general physician and/or the emergency hospital departments. If in most cases, benign recurrent viral infections are in causes, it may underlie rare well–defined disorders such as systemic auto inflammatory diseases (SAID). At present few is known on the degree of awareness of liberal paediatricians on SAID and nothing on how they deal with these patients in terms of further investigations, treatment and referral. FIREVILLE study tries to better understand why patients with SAID undergo complex medical journey before appropriate referral in our country.

**Objectives:** To survey, liberal paediatricians on theirs knowledge and practices regarding children with recurrent fevers

**Methods:** We took the facilities of the AFPA (French Association of Ambulatory Paediatrics) to email 1248 active liberal paediatricians between June and September 2018, to answer a Survey-Monkey type questionnaire. The survey, included 36 questions divided into 3 parts; i.e.Socio-demographic characteristics of paediatricians and their knowledge on recurrent fever syndromes (RFS), clinical case analysis, then more general evaluation of knowledge on and analysis of city-hospital networks.

**Results:**

360 paediatricians (28.8%) answered the survey after 4 email reminders. 77% were women aged 44 to 60 years with a completion rate of 79%. 22% had part time of hospital practice. The top 3 regions were: Paris ile de France: 24.9%, Auvergne-Rhône-Alpes: 18.3% and Provence-Alpes-Côte d'Azur f 9,9%. 69% of paediatricians considered their knowledge on HRF, and 83% on SAID. PFAPA was the best known (46%), on the basis of the frequency (92%) and the regularity (89%) of fever. Only 66% considered the duration of fever as an added diagnostic value. The clinical case was a PFAPA chart. 92% of paediatricians required whole blood cell count and 91.8% a CRP. 70% of paediatricians ruled out radiologic evaluations. They were 80.6% to suspect PFAPA and 14% to suspect FMF. 57% asked second opinion, in a paediatric rheumatology unit (46%) and in general paediatrics (35%). Youngest paediatricians chose more significantly a paediatric rheumatology unit (p = 0.009).Only 44% of responders knew a SAID referral centre in their area. Finally, 90% were interested in joined city-hospital care.

**Conclusion:** This is the first study surveying liberal paediatricians on their knowledge and practices with a child with supposed HRF. In spite of their thought insufficient knowledge, their answers were accurate in most cases, however the questionnaire revealed insufficient knowledge of the dedicated resources and network for SAID.

**Disclosure of Interest**

None Declared

#### P1040 Crimean Tatars is new target nationality for the familial Mediterranean fever

##### Olga Zhogova^1^, Natalya Lagunova^1^, Sergey Ivanovskiy^1^, Evgeny Suspitsin^2,3^, Mikhail Kostik^2^

###### ^1^V.I. Vernadskiy, Crimean Federal University, Simferopol; ^2^Saint-Petersburg State Pediatric Medical University; ^3^N.N. Petrov Institute of Oncology, Saint-Petersburg, Russian Federation

####### **Correspondence:** Mikhail Kostik

**Introduction:** Familial Mediterranean Fever (FMF) is a monogenic autoinflammatory disease with high prevalence in some nationalities, such as Jew, Armenians, Turkish, Arabians and other nationalities with Mediterranean origin. Before 2016 there were no data about FMF distribution in the Crimea region, but the first 15 new cases of FMF were diagnosed in the last 2 years.

**Objectives:** The aim of our study is the evaluation of the distribution of FMF in the Crimea region.

**Methods:** Our cohort consists of 13 children and 2 adults, among them were 2 parents and 6 kids from 1 family. All belong to Crimea Tatar nationality. This nationality is close to Turkish. The diagnosis of FMF was based on the Tel-Hashomer criteria and later was confirmed by MEFV gene sequence.

**Results:** 10 children with FMF have M694V heterozygous mutation, 3 have M694V homozygous mutations, and 2 adults (parents) have M694V homo (father) and heterozygous (mother) mutations. Ten children and 2 adults are treated with colchicin. 2 kids and 1 adult (all M694V/M694V) received canakinumab due to inefficacy and 1 child (M694V/N) due to intolerance of increased doses. Clinical characteristic of the studied population are in the table.

**Conclusion:** Crimean Tatars is a new nationality with an increased prevalence of FMF with typical high penetrance mutations. Further epidemiological studies required about MEFV alleles distribution in the healthy population and in the FMF patients.

**Disclosure of Interest**

None Declared

**Table 1 (abstract P1040).** See text for description

	The # of patients
Big criteria	
1. Typical recurrent seizures fever with serositis	15
2. Peritonitis	13
3. Pleurisy	1
4. Monoarthritis (hip, knee, ankle joints)	12
Small criteria	
1. Stomach	13
2. Thorax	1
3. Joint	10
4. Load pain in legs	9
5. A good response to colchicine therapy.	9
Supporting criteria	
1. The presence of FMF cases in the family history	11
2. Belonging to the relevant ethnic group	15
3. Age of onset of the disease d 20 years	15
4. heavy bedridden	14
5. spontaneous resolution attack	15
6. the presence of bright gaps	15
7. increase in laboratory markers of inflammation	14
8. episodes of proteinuria/hematuria	
9. unproductive laparotomy or removal of “white”appendix	
10. parental blood marriage	3

#### P1041 New variant in the IL1RN-gene associated with late onset and atypical presentation of DIRA – follow-up

##### Jasmin B. Kuemmerle-Deschner^1^, Kerstin Reicherter^2^, Susanne Schlipf^3^, Sandra Hansmann^1^, Anton Hospach^4^, Ilias Tsiflikas^5^, Xiao Liu^6^, Susanne Benseler^7^, Alexander Weber^6^

###### ^1^Department of Pediatrics, Division of Pediatric Rheumatology, University Hospital Tuebingen; ^2^CEGAT, Tuebingen; ^3^Kinderarztpraxis Dr. Lakner, Schwäbisch Gmünd; ^4^Zentrum für Pädiatrische Rheumatologie, Klinikum Stuttgart, Olgahospital, Stuttgart; ^5^Pediatric Radiology, Department of Radiology, University Hospital Tuebingen; ^6^Department of Immunology, University of Tuebingen, Tuebingen, Germany; ^7^Rheumatology, Department of Pediatrics, University of Calgary, Calgary, Canada

####### **Correspondence:** Jasmin B. Kuemmerle-Deschner

**Introduction:** Deficiency of the Interleukin-1 receptor antagonist (DIRA) is an autoinflammatory disease characterized by severe systemic inflammation with bone and skin involvement present in the first days of life. Here we report on diagnosis, treatment and follow up of a novel variant in the *IL1RN*-gene associated with late onset and atypical phenotype of DIRA.

**Objectives:** Here we report on diagnosis, treatment and follow up of a novel variant in the *IL1RN*-gene associated with late onset and atypical phenotype of DIRA.

**Methods:** A 3 year-old boy presented with recurrent monthly episodes of fever and fatigue, associated with lymphadenopathy, pericarditis, pleuritis, pancreatitis, and arthritis involving sacroiliac, hip, knee and ankle joints in the absence of any skin involvement. Symptoms had started at age one and had progressed over time to life-threatening episodes requiring intensive care therapy. Throughout, inflammatory parameters including ESR, CRP, SAA, S100A8/9, leukocytes and platelet counts were highly elevated. Treatment with colchicine and steroids improved symptoms but did not prevent flares. Typical immune deficiencies were ruled out; genetic testing for FMF, CAPS, TRAPS, HIDS and DITRA did not reveal variants in the associated genes.

**Results:** Whole exome sequencing detected a novel homozygous stop variant c.62C>G; p.Ser21* in the *ILRN*gene (NM_173842.2). Mother, father and brother were heterozygous for the same variant. In addition, three variants of unknown significance were identified in the patient's *PCGF5, CPA1*and *SPTA1*genes. Functional studies revealed only marginal secretion of IL-1RA in the patient’s unstimulated leukocytes and after stimulation with IL-1βand LPS, confirming the disease-causing nature of the variant.

IL-1 inhibition with anakinra at 2 mg/kg/d was started and resulted in complete resolution of clinical symptoms and signs of inflammation on MRI, in normal inflammatory markers, and dramatically improved energy levels. Intolerance to daily subcutaneous injections prompted a switch to canakinumab at 4 mg/kg/4 weeks. However, as per the patient’s and mother’s assessment of disease activity, canakinumab was inferior to anakinra. After four months a flare occurred, prompting re-start of anakinra and resulting in sustained and complete resolution of symptoms in an observation period of 14 months.

**Conclusion:** This is the first report of the novel c.62C>G; p.Ser21* variant in the *IL1RN*-gene primarily causing severe serositis in a homozygous carrier, while heterozygous family members were completely symptom-free. Skin disease, one of the most prominent features in other patients with DIRA was not observed in this patient, while IL-1 inhibition was likewise effective.

The different phenotype in the patient reported here, may be due to the selective loss of secreted IL1RN. Unlike one report of successful canakinumab treatment of one patient with late onset of DIRA [1], our patient did not respond favourably to canakinumab, but treatment with anakinra lead to sustained remission.

*1 Ulusoy et al. J Med Case Reports 2015; 9:145*

**Consent for publication has been obtained from patient**

Yes

**Disclosure of Interest**

J. Kuemmerle-Deschner Grant / Research Support from: Novartis, SOBI,Consultant for: Novartis, SOBI, K. Reicherter Employee of: CEGAT, S. Schlipf: None Declared, S. Hansmann: None Declared, A. Hospach Consultant for: Novartis, Chugai-Roche, SOBI, I. Tsiflikas: None Declared, X. Liu: None Declared, S. Benseler Consultant for: Novartis, SOBI, abbvie, A. Weber: None Declared

#### P1042 Efficacy of anti-IL-1 treatment in familial Mediterranean fever: a single-center experience

##### Tuba Kurt, Halide O. Basaran, Fatma Aydın, Nermin Uncu, Banu Celikel Acar

###### Pediatric Rheumatology, Ankara, Child Health Hematology and Oncology Education and Research Hospital, Ankara, Turkey

####### **Correspondence:** Tuba Kurt

**Introduction:** In 5%–10% of patients with familial Mediterranean fever (FMF), colchicine is not effective in preventing inflammatory attacks. Furthermore, another 5%–10% of patients are intolerant to effective doses of colchicine and experience serious side effects. In recent years, it has been shown that interleukin-1 (IL-1) plays a central role in the pathogenesis of FMF.Several reports have pointed out the efficacy of IL-1 blockade in colchicine resistant FMF.

**Objectives:** To review the patients followed with FMF who received anti-IL-1 treatment, in terms of outcome and side effects

**Methods: 18** FMF patients who were treated with anti-IL-1 treatment were retrospectively reviewed with regard to indication, effect on disease activity and acute phase response, adverse events and AIDAI score and patient global assessment. Colchicine resistance was defined as at least one attack per month for three consecutive months and elevated erythrocyte sedimentation rate or C-reactive protein in-between attacks despite taking adequate dose of colchicine.

**Results:** There were 18 patients with FMF (9 M/ 9 F) who were treated with Anakinra and Canakinumab for various indications (colchicine resistant recurrent febrile attacks in 16, colchicine related side effects in 1, subclinic inflamation in 1). 11 patients were treated with Anakinra while were 15 patients with Canakinumab. All patients except 5 had homozygous or compound heterozygous exon ten mutations. The mean age of onset of anti-IL-1 treatment was 15± 2 (11-18 years) years. The mean duration of the disease was 11.1 ± 4.34 years. All patients were taking adequate dose of colchicine for their age before treatment with a median dosage of 0,03 ±0,012 mg/kg/day before anti-IL-1 treatment (0.03–0.06 mg/kg/day). 11 patients were treated with anakinra with a median duration of 29,7 months (8- 60 months), but 6 of them switched to canakinumab because of noncompliance and side affects (2 headache, 1 urticerial rash), all responded.After the initiation of Anakinra treatment 6 patients became attack-free, 2 patients reported more than 50% decrease, and 3 patients no change in the frequency of the attacks.

15 patients were treated with Canakinumab but 2 (%13,3) of them swiched to anakinra because had increase in frequency of the attacks. All of patients complete respondend and any of them were no side effects.

A significant decrease was observed in the mean CRP (from 8.5± 8.7 mg/dLto 0.68 ± 0.75 mg/dL), WBC (from 10335 ± 3206 mm³ to 7047 mm³± 2108mm³) and ESR levels (from 44.35 ± 18.7 mm/h to 9.5 ± 7.6 mm/h), respectively (referance range for CRP: 0–0.3mg/dL).

AIDAI score decreased from 25±17,5 to 0,33±1 and mean pyscian’s global assessment 7,4±1,5to 1,25±1,1 respectively, under anti-IL-1 treatment treatment.

As for the adverse events, 1 patients (%9) had allergic reactions under Anakinra treatment (severe disseminated rash in 1 patient ) and 2 patients (%18,1) had headache which necessitated termination of treatment in 3 patients. There were no adverse events in the remaining all patients during the course of treatment.

**Conclusion:** Based on the results of our study, anti-IL-1 therapy seems to be a safe and effective alternative for patients with FMF who do not respond to or cannot tolerate colchicine, however approximately one fourth of the patients stop anakinra for insufficient response and injection site reaction. The treatment should be modified and decided for each patient on an individual basis.

**Disclosure of Interest**

None Declared

#### P1043 Erysipelas-like erythema as the primary gripe of FMF

##### Omer Kuru^1^, Ahmet Ki̇vanc Cengi̇z^2^

###### ^1^Physical Medicine and Rehabilitation, Division of Rheumatology, University of Heath Sciences, Istanbul Okmeydani Research and Training Hospital, Istanbul; ^2^Physical Medicine and Rehabilitation-Rheumatology, 19 Mayis University Faculty of Medicine, Samsun, Turkey

####### **Correspondence:** Omer Kuru

**Introduction:** Familial Mediterranean Fever (FMF) is a monogenic autoinflammatory disease characterized by recurrent polyserositis attacks, mainly involving peritoneum, pleura and synovium. Erysipelas-like erythema (ELE) is an aseptic neutrophilic dermatose defined as well-demarcated, warm, tender, erythematous and infiltrated plaques which resolves spontaneously in 2-3 days. ELE is a pathognomic cutaneous manifestation of FMF. The reported frequency of ELE is 5-30 % in different populations. It typically developes on the extensor surface of lower extremities especially on dorsum of ankles and feet. It is usually unilateral and associated with more severe FMF clinical phenotype and M694V homozygosity.

**Objectives:** Here we would like to present a 12 year old girl admitted to the emergency service with an erythematous, painful rash at the dorsum of her foot. This was her fourth admission in approximately 9 monthstime with a similar skin lesion at dorsum of the same foot. In her medical records previous diagnosis for the described lesion were insect bite, contact dermatitis and cellulitis. Oral and topical antibiotics, topical steroids were prescribed in her previous visits. She told that regardless of the medication used the lesions resolved spontaneously in 3-4 days time. She had also noticed that the lesions usully occured after her volleyball matches. Exercise and long time standing usually has caused pain in her legs and if she insists on the activity despite this pain, the lesions occured. She had no prominant abdominal or pleural pain. She didn’t have arthritis but had episodes of fever lasting 36-48 hours accompanying the skin lesions. Her family history was unremarkable except FMF in one of her cousins. Her laboratory tests revealed leukocytosis, elevated crp and fibrinogen levels. She did not have proteinuria.

**Methods:** Case report

**Results:** The recurrence of the typical lesion, spontaneous recovery in 3-4 days time, accompanying fever and positive family history reminds the diagnosis of FMF. Genetic analysis was performed and compound heterozygous genotype (M694V/V726A) was detected. Colchicine was prescribed. She is 14 years old now and did not have any typical serositis attack yet. Under colchicine treatment the ELE attacks did not disappear completely but their frequency and severity decreased prominently and fever did not accompany her ELE attacks any more.

**Conclusion:** Spontaneous recovery, recurrence of the attacks, accompanying fever and elevated acute phase reactants during the attacks points out FMF especially in inhabitants of the Mediterranean basin. Occurence of ELE as the first manifestation of FMF is rare but this possibility must also be kept in mind.

**Consent for publication has been obtained from patient**

Yes

**Disclosure of Interest**

None Declared

#### P1044 Analysis of new referrals to a specialist UK adult autoinflammatory disease service

##### Serdal Ugurlu^1^, Philip N. Hawkins^2^, Charalampia Papadopoulou^2^, Tamer Rezk^2^, Dorota Rowczenio^2^, Helen J. Lachmann^2^

###### ^1^Division of Rheumatology, Department of Internal Medicine, Cerrahpasa Medical Faculty, University of Istanbul - Cerrahpasa, Istanbul, Turkey; ^2^National Amyloidosis Centre, Centre for Amyloidosis and Acute Phase Proteins, Division of Medicine, Royal Free Campus, UCL, London, United Kingdom

####### **Correspondence:** Helen J. Lachmann

**Introduction:** Diagnosis of the SAIDs requires a high index of suspicion and previous series has suggested that there are often long diagnostic delays, particularly in TRAPS and MKD.

**Objectives:** To look at the case mixed referred to a single adult clinic in London specialising in assessment of potential SAIDS over the course of the year of 2017.

**Methods:** All new referrals were accepted for clinical assessment. At the first visit patients had a full history and examination, genetic testing – varying from single gene to a 20 gene panel depending on clinical features, and laboratory testing including fortnightly blood draws for serial analysis of the hepatic acute phase response proteins, CRP and SAA over a 3 month period.

Patients with a non suggestive history, non contributory genetic testing and no evidence of inflammation accompanying symptoms were felt not to have SAIDS and referred back to their local hospitals for further management. Other cases were diagnosed based on full clinical assessment, other investigations – for example ferritin in AOSD, genetic testing results, serial monitoring of CRP and SAA and therapeutic trials, for example colchicine in presume FMF and anti IL-1 therapies in CAPS and Schnitzler’s syndrome.

**Results:** 273 new patients were referred. Median age at referral was 37.4 years, the oldest patient was 84.3 years old and 59% were female. 174 (64%) were of northern European ancestry, 68 (25%) were eastern Mediterranean, west Asian or southern European ancestry, 19 (7%) were of south or east Asian ethnicity and 4% were of African or Afro-Caribbean ancestry. 76% of referrals were from hospital specialities. The referral source was: rheumatology 38%, general practitioner 24%, dermatology 8%, immunology 8%, gastroenterology 6%, infectious diseases 3%, clinical genetics 3%, nephrology 2%, haematology 2%, gynaecology 2%, emergency department 1%, respiratory 1%, other 2%.

After work up 135 (49.5%) were felt not to have a SAID as the cause of their symptoms. Of the remaining 138 patients who did have evidence of a SAID the diagnoses made were: FMF 33%, uncharacterised SAID 26%, CAPS 9%, AOSD 8%, recurrent idiopathic pericarditis 6%, Schnitzler’s Syndrome 5%, TRAPS 4%, variant PFAPA 4%, DADA2 1%, MKD 1%, CRMO 1%, Behcets 1%, Cattleman’s disease 1%.

The median interval between reported symptom onset and diagnosis were as follows: 16 yrs for FMF, 28.1 yrs for CAPS, 5.0 years for recurrent idiopathic pericarditis, 4.5 yrs for Schnitzler’s Syndrome, 5.7 yrs for TRAPS, 20.5 yrs for variant PFAPA,12.5 yrs for DADA2, 17 yrs for MKD and 2 years for CRMO**.**

**Conclusion:** This series suggests that recognition and diagnosis of the SAIDS remains a challenge. More than 1/3 of referrals were from rheumatology, referrals from primary care were almost exclusively from patients with a known family history of one the inherited syndromes. The wide variety of referring specialities reflects the diverse nature of SAIDS and the importance of almost all specialities considering the possibility of SAIDS. Only just over 50% referrals had evidence of diseases falling within the recognised SAID spectrum and 26% of these have currently uncharacterised disease with non diagnostic genetic testing. Of those in whom a diagnosis could be made there are significant diagnostic delays fortunately despite late initiation of treatment no patients had evidence of systemic AA amyloidosis.

**Disclosure of Interest**

None Declared

#### P1045 Application of autoinflammatory disease damage index (ADDI) to other autoinflammatory diseases in a tertiary referral hospital

##### Mireia Lopez, Estefania Moreno

###### Pediatric Rheumatology, Hospital Vall d’Hebron, Barcelona, Spain

####### **Correspondence:** Mireia Lopez

**Introduction:** Autoinflammatory diseases (AIDs) cause systemic inflammation that can be chronic and result in damage to multiple organs. Recently, the autoinflammatory disease damage index (ADDI) has been developed and validated to the four most common monogenic AIDS, cryopyrin-associated periodic syndrome (CAPS), familial Mediterranean Fever (FMF), mevalonate kinase deficiency (MKD) and tumor necrosis factor receptor-associated periodic fever syndrome (TRAPS). ADDI could be useful in other AIDs different than the four reported.

**Objectives:** The aim of the study is to asses the application of ADDI to patients with AIDs followed in our hospital with the four most common monogenic diseases and other AIDs, determine the reliability and point out encountered comments about the scoring.

**Methods:** All patients with AIDs followed in Transitional Care unit or Pediatric Rheumatology specialized in AIDs consult from Hospital Universitari Vall d’Hebron were identified. A cross- sectional, descriptive study was performed applying ADDI by two pediatric rheumatologists (EM, ML). Laboratory test including C-reactive protein (CRP) mg/dl, amyloid protein (AP) mg/L, erythrocyte sedimentation rate (ESR) mm/h and protein/creatinine rate (mg/g Cr) were performed at the moment ADDI was applied. Variables related with disease duration, current treatment and accumulated corticosteroids treatment was assessed. The continuous data are presented as mean and standard deviation (SD). Categorical variables are presented by percentages.

**Results:** A total of 41 patients with AIDs were included, of whom 61% were female, with a median age of 20 years (SD 11.9) at inclusion. Disease duration has a mean of 11 years (SD 8.2). AIDs included were 11 patients with FMF (26.8%), TRAPS n=4 (9.8%), MKDn=3 (7.3%), CAPS n= 2 (4,9%), Blau syndrome n= 7 (17.1%), SAVI syndrome n=3 (7.3%), CRMO n=4 (9.8%), PFAPA n=2 (4.9%), APLAID n=1 (2.4%), Stickler syndrome n=1 (2.4%), and 3 unknown AIDs with genetic test negative n=3 (7.3%). Current treatment is variable among patients, 6 (15.8%) are taking disease-modifying antirheumatic drugs (DMARDs), 9 (23.7%) Colchicine, 8 (21.1%) Anakinra, 13 anti-TNF therapy (34.2%), 1 (2.6%) Ruxolitinib and 1 (2.6%) Abatacept.Just 6 patients were receiving corticoids with mean prednisone dose of 7.5 mg/day.

ADDI mean score was 2.3 (SD 2.2) for all patients. Regarding the eight different items evaluated, musculoskeletal domain was the highest punctuated with a mean of 1.02 points, followed by ocular domain with 0.42 points. Laboratory test results were mean ESR 27.2 mm/h (SD 26.7), CRP 0.7 mg/dl (SD 1.3), AP 13.9 mg/L (SD 18.6). Proteinuria was present in 2 patients with mean 286.5 mg/g (SD 246.1). EM and ML applied ADDI in 5-10 minutes average.

**Conclusion:** ADDI is a feasible index suitable to measure damage in a single patient. Despite it was performed to the four most common AIDs it could be applied to other diseases. In our cohort mean ADDI was low with musculoskeletal item as the highest punctuated. This result could be explained by the correct control of the disease with the current treatment.Laboratory tests also support this finding. Despite good response to medication, it is difficult to reverse bone deformities or joint restriction. Nevertheless, some organ systems are not represented like respiratory, cardiovascular or cutaneous damage, important in some syndromes included in our cohort. Knowing the difficulties of conducting an unified index for all diseases, ADDI must be applied in longitudinal cohorts.

**Disclosure of Interest**

None Declared

#### P1046 Vasculitis in autoinflammatory diseases in a tertiary hospital

##### Rosa M. Alcobendas, Sara M. Loza, Pablo F. Fraga, Clara U. Gascon, Catarina Fervenza, Agustin Remesal

###### Pediatric Rheumatology, University Hospital La Paz, Madrid, Spain

####### **Correspondence:** Sara M. Loza

**Introduction:** Autoinflammatory diseases with vasculitis and interpheronopaties have been recently described and may present with variable clinical signs. Such entities are considered rare diseases and potentially life-threatening. Clinicians should be aware of the existence of autoinflammatory vasculitis to diagnose and treat them correctly.

**Objectives:** To describe the initial manifestations, laboratory findings and therapeutic approaches of patients diagnosed with autoinflammatory vasculitis during the last 5 years in a pediatric rheumatology unit of a tertiary hospital.

**Methods:** Retrospective chart review. Inclusion criteria were: children under 18 years diagnosedwith monogenic autoinflammatory vasculitis in a tertiary hospital.

**Results:** During the study period, 5 patients were identified (2 boys and 3 girls). The main clinical manifestations were severe cutaneous lesions (5/5), intermittent fever (4/5) and neurological involvement (4/5) (see table 1).The only patient without neurologic manifestations had relatives with psychomotor retardation and sensorineural symptoms of unknown etiology. All patients were diagnosed of with another rheumatology condition,with no response to conventional treatment.

**Conclusion:** Autoinflammatory vasculitis are extremely rare entities but potentially fatal. The early age of onset, the intense skin involvement as well as the presence of affected relatives may be suggestive data. Clinical suspicion is important in order to establish an early and adequate treatment.

**Disclosure of Interest**

None Declared

**Table 1 (abstract P1046).** Clinical characteristics of the patients

	Diagnosis and gene	Age of onset and clinical manifestations	Laboratory & inmunology	Previous diagnosis	Treatment & response
Patient 1	CANDLE (PSMB8)	Neonatal period: Fever, chondritis, aseptic meningitis, joint limitations, erythematous plaques,lipodystrophy, lymphadenopathy, splenomegaly and hepatomegaly	Anemia, ESR 122 mm/h, CRP 100 mg/L, AST 55 U/L, ALT 51 U/L,	CINCA	Anakinra (no) Tocilizumab (no) Baricitinib (yes)
Patient 2	ISG15 defficiency (ISG15)	6 months: Intermittent fever, severe cutaneous ulcerations skin, chorioretinitis, brain calcifications	CRP 75 mg/L, HLA B51 (+), ANA (-), ANCA (-), AST 80 U/L, ALT 65 U/L	Early onset Behçet disease	Corticosteroids (yes) Etanercept (partial) Anakinra (no) Adalimumab (no: ADA) Tocilizumab (partial)
Patient 3	FCL (TREX1)	6 years: Raynaud, amputation of phalanx, inflammatory arthritis and chilblains	Normal blood counts, Inmunology (-)	JIA	Etanercept (no) Tofacitinb (yes)
Patient 4	DADA2 (CECR1)	7 months: intermittent fever, livedo reticularis, paralysis of VI craneal nerve, Raynaud	CRP 16 mg/L, IgG 567 mg/dl, IgA 22 mg/dlIgM 32 mg/dl, Total lymphocytes 950/μL ANA and ANCA (-)	PAN	ASA Etanercept (yes)
Patient 5	DADA2 (CECR1)	5 years: Intermittent fever, livedo resticularis, paralysis of VI craneal nerve, painful nodular lesions, arthromyalgia, abdominalgia, Raynaud, peripheral neuropathy	ESR 27 mm/h; RCP 87.6 mg/L ANA and ANCA (-)	PAN	ASA Corticosteroids (yes) Cyclophosphamide (no) Etanercept (unknown) ---

CRP: C-reactive protein, ESR: erytrosedimentation rate, ADA: antidrug antibodies, FCL: familiar chilblain lupus, JIA: Juvenile Idiopathic Arthritis, PAN: polyarteritis nodosa, ANA: antinuclear antibodies, ANCA: antineutrophilic antibodies, ASA: acetylsalicylic acid

#### P1047 Diagnosis and treatment of periodic fevers: a single centre experience

##### Peter McNaughton^1,2^, Jane Peake^1,3^, Ben Whitehead^1,3^, Su Han Lum^2^, Kahn Preece^4^

###### ^1^Queensland Children's Hospital, Brisbane, Australia; ^2^Great North Children’s Hospital, Newcastle upon Tyne, United Kingdom; ^3^University of Queensland, Brisbane; ^4^John Hunter Hospital, Newcastle, Australia

####### **Correspondence:** Peter McNaughton

**Introduction:** Diagnosis of periodic fever syndromes is difficult due to atypical presentations and overlap in inflammatory symptoms. Treatment of suspected periodic fevers varies widely due to the lack of established clinical guidelines.The utility of genetic testing in identifying monogenic periodic fever syndromes is also unclear due to high frequency of variants of uncertain significance, somatic mutations and heterozygous mutations in genes associated with autosomal recessive conditions.

**Objectives:** This study aims to evaluate the diagnosis, treatment and use of genetic testing of patients diagnosed with periodic fever syndromes at a single tertiary paediatric hospital.

**Methods:** We retrospectively reviewed the clinical history of patients diagnosed with periodic fever syndromes at Queensland Children’s Hospital, Brisbane, Australia between November 2014 and June 2018.

**Results:** 43 patients were diagnosed based on their clinical presentation with periodic fever syndromes.10 patients were diagnosed with PFAPA, 9 with TRAPS, 6 with CAPS, 4 with MKD and 14 unspecified.Median age of onset of symptoms was 24m (range: birth-96m) and median age of diagnosis was 60m (9m-180m).Median time to diagnosis from onset of symptoms was 24m (0-149m).

Multiple medications were used in 15 patients.The medications used varied widely (prednisolone (22), anakinra (9), etanercept (5), tofacitinib (2), tocilizumab (2), cimetidine (2)).

Genetic testing of between 1-26 genes was performed in 26 patients (60%).1-3 genes were tested in 13 patients, targeted panels in 10 patients, SNP only in 1 patient.Genetic variants were identified in 9 patients (34% of those tested) however only 2 of these variants were clearly pathogenic (7.7% of those tested).

Clinical diagnosis and the Eurofever classification criteria were in agreement for patients diagnosed with CAPS (p=0.046) and TRAPS (p=0.025) but not for patients diagnosed with MKD (p=0.47).10 of the patients where clinical diagnosis and Eurofever classification criteria were in agreement had 2 diagnoses positive on the classification score.Two patients diagnosed with CAPS were exclusively positive for CAPS on the classification score and none of the patients diagnosed with TRAPS were exclusively positive for TRAPS.6 of the 7 TRAPS patients had a positive eurofever score for at least one PFS diagnosis.

**Conclusion:** As reported in previous studies there was a significant delay between onset of symptoms and diagnosis.This reflects an ongoing need to raise awareness of these conditions with primary care providers. The large number of patients treated with multiple medications and the broad range of medications used reflects the lack of established treatment protocols and varied response to treatments in this group of patients.

The clinical diagnosis and diagnostic score showed agreement for CAPS and TRAPs however many patients had a diagnostic score positive for more than one diagnosis meaning that a combination of clinical score and clinical judgement is required to make a diagnosis.

Consistent with previous studies many patients with heterozygous mutations in genes associated with periodic fevers were identified.The significance of these variants is not clear and the diagnostic yield from genetic testing in this cohort was low. Further improvements in availability of next generation sequencing and molecular understanding of autoinflammatory conditions will hopefully improve this yield and allow more confident diagnoses and targeted therapies.

**Disclosure of Interest**

None Declared

#### P1048 Multifocal osteomyelitis revealing a PSTPIP1- associated myeloid-related proteinemia inflammatory (PAMI) syndrome: case report and review of the literature

##### Manel Mejbri, Katerina Theodoropoulou, Michael Hofer

###### Femme-Mère-Enfant, Centre Hospitalier Universitaire Vaudois CHUV, Lausanne, Switzerland

####### **Correspondence:** Manel Mejbri

**Introduction:** PAMI syndrome is a recently described condition, previously known as Hyperzincemia/Hypercalprotectinemia(Hz/Hc) syndrome. It is a very rare auto-inflammatory disorder characterized by a chronic systemic inflammation, cutaneousand osteo-articular manifestations, hepatosplenomegaly, anemia and neutropenia. Increased blood levels of MRP 8/14(S100A8/A9 or calprotectin) and zinc distinguish this condition. Specific pathogenic mutations in PSTPIP1 gene (p.E250K andp.E257K) were identified as the genetic cause of this condition.

**Objectives:** Case report and review of the literature of PAMI syndrome

**Methods:** We report a case of 13 months age female referred to our unity for recurrent episodes of osteoarthritis.Physical examination showed hepatosplenomegaly. Blood work revealed a systemic inflammation, a microcytic anemia and neutropenia. A complete workup for metabolic disorders, oncologic processes and uncommon infections was negative.Because of history of recurrent osteoarthritis, a whole-body MRI was performed and confirmed a multifocal osteomyelitis.

Whole exome sequencing identified the missense p.E250K in the PSTPIP1 gene.

A literature search on PAMI syndrome was performed until the15 October 2018. Pubmed was screened using a combination of the following terms: Hyperzincemia, Hypercalprotectinemia, E250K mutation, PSTPIP1 mutation, PAPA with E250K mutation.

**Results:** We identified 20 cases of PAMI syndrome in the literature. PAMI syndrome is an early onset inflammatory diseasewith a median age of 2.4 years. Clinical manifestations include Osteo-articular manifestations (80%), skin lesions (71%), splenomegaly (89%), hepatomegaly (68%), lymphadenopathy (42%), growth failure (58%) and hemorragic diasthesis withrecurrent epistaxis and/or haematoma tendency in 5 patients. All cases had relevant abnormalities in hematologic parameters:mild to severe neutropenia and anemia (100%). Thrombocytopenia (42%). Systemic inflammation was confirmed in 94% using the monitoring of CRP, ESR or SAA. Zinc and MRP 8/14 blood concentrations were markedly elevated in all tested patients. Genetic analyses of PSTPIP1 gene revealed the two specific identified mutations (p.E250K and p.E257K) in all patients. Response to the treatment was variable with no consistently effective therapy. Most common therapeutic optionswere AINS, Corticosteroids (n=9), Anakinra (n=9), Anti-TNF (n=6) and Cyclosporine A (n=4).

**Conclusion:** PAMI syndrome is a rare auto inflammatory condition which should be considered in patients with undefined systemic inflammation and neutropenia, even without skin or osteo-articular manifestations. Zinc and serum MRP 14/8measurement may be helpful tools for the diagnostic orientation in these cases.

**Consent for publication has been obtained from patient**

Yes

**Disclosure of Interest**

None Declared

### Monogenic autoinflammatory diseases (basic science)

#### P1049 Investigation of inflammasome components in the process of cell migration in FMF patients

##### Tayfun H. Akbaba^1^, Z. Yeliz Akkaya-Ulum^1^, Selcan Demir^2^, Zeynep Tavukcuoglu^1^, Seza Ozen^2^, Banu Balci-Peynircioglu^1^

###### ^1^Medical Biology; ^2^Pediatric Nephrology and Rheumatology, Hacettepe University, Ankara, Turkey

####### **Correspondence:** Tayfun H. Akbaba

**Introduction:** Autoinflammatory diseases, periodic fever syndromes, are a group of diseases that develop recurrent inflammatory response in the absence of infection. Familial Mediterranean fever, which is the most common disease among autoinflammatory diseases, is caused by mutant pyrin production due to mutations in *MEFV* gene. Pyrin protein is thought to be involved in inflammatory pathways by means of protein-protein interactions. There are studies supporting the role of pyrin as a proinflammatory regulator in the literature and involved in pathways associated with cell migration.

**Objectives:** The aim of this study was to investigate the effect of pyrin inflammasome on cell migration in FMF patients, in CAPS patient as disease control and healthy controls.

**Methods:** Within the scope of the thesis; pyrin inflammasome was examined in mononuclear cells isolated from the blood samples of 11 FMF patients homozygous for M694V mutation, 1 CAPS patient with somatic mosaicism and 7 healthy individuals in the process of inflammatory cell migration. Lipopolysaccharide was used to induce the activation of inflammasome in the cells, while arachidonic acid was used for the inhibition of inflammasome. IL-1 beta secretion was analyzed by western blot as an indicator of inflammasome activation, and transwell filter assay was performed in order to examine the differences in cell migration.

**Results:** We demonstrated that the inhibition of inflammasome in the cells of FMF patients compared to the controls was less under the same application conditions. Following inflammasome activation and inhibition, filter experiments were performed and fetal bovine serum was used to induce inflammatory cell migration. In both inflammasome activation and suppression conditions; we observed a statistically significant increase in the ratio of cell migration of the mononuclear cells of the FMF patients compared to CAPS patient and control indivuals.

**Conclusion:** These findings support the idea of increased cell migration ratio in patients with FMF due to the more active pyrin inflammasome seen in patients. Although the involvement of other inflammasome components remains to be defined, this study has made significant contributions to illuminate the role of pyrin protein in inflammatory cell migration through the structure of inflammasome.

**Keywords:** FMF, pyrin inflammasome, cell migration.

This thesis was supported by Hacettepe University Scientific Research CoordinationUnit (Project Number: TYL-2018-17354)

**Disclosure of Interest**

None Declared

#### P1050 Cell migration defect in hyperimmunoglobulin D syndrome

##### Tayfun H. Akbaba^1^, Selcan Demir^2^, Z. Yeliz Akkaya-Ulum^1^, Seza Ozen^2^, Banu Balci-Peynircioglu^1^

###### ^1^Medical Biology; ^2^Pediatric Nephrology and Rheumatology, Hacettepe University, Ankara, Turkey

####### **Correspondence:** Tayfun H. Akbaba

**Introduction:** Hyper-IgD syndrome or Mevalonate Kinase Deficiency(HIDS/MKD, MIM #260920) is a ultra rare, autosomal recessive hereditary autoinflammatory syndrome caused by compound heterozygous or homozygous mutations in the mevalonate kinase(MVK) gene. It is characterized by recurrent febrile episodes with abdominal pain, lymphadenopathy and an increased serum immunoglobulin D (IgD) level.Anti-IL-1 and anti-TNF treatment are applied to alleviate the symptoms of the disease and to reduce the number of attacks.

**Objectives:** In this study, we aimed to analyze cell migration, an important process of inflammation,in HIDS patients and compare with FMF patients and controls in inflamasome activated and inhibited conditions.

**Methods:** Peripheral blood mononuclear cell isolation from HIDS patients, FMF patients and controls was performed. LPS was used to induce inflammasome in isolated cells while arachidonic acid was used for inhibition of inflammasome.After activation and inhibition of inflammasome, transwell filter experiments were performed with isolated cells from patients. As a result of the experiment, the migrating cells were stained with calcein-AM and analyzed by Image J programme.

**Results:** We did not observe cell migration in HIDS patients’ mononucleer cells under normal conditions, after LPS stimulation. We observed increased cell migration in FMF patients and normal rate in controls as a positive control of these experiments.Considering that 2 HIDS and 1 FMF patients involved in these experiments were receiving anti-IL-1 treatment, it was thought that the drug used by the patients had no effect in cell migration pattern that we observed.

**Conclusion:** Our preliminary data is the first study identified cell migration defect seen in HIDS. Due to the mutant protein produced as a result of MVK gene mutations, the deficiency in the function of the mevolanate kinase enzyme observed in HIDS leads to the depletion of geranylgeranyl pyrophosphate, a key substrate for protein prenylation.Considering the close relationship between protein prenylation and cytoskeleton elements related with cell migration, a possible decrease in prenylation may be a potential explanation of cell migration defect seen in HIDS patients.

**Keywords:** HIDS, cell migration defect, prenylation

This project was supported by Hacettepe University Scientific Research Coordination Unit (Project Number: TYL-2018-17354)

**Disclosure of Interest**

None Declared

#### P1051 Possible regulatory effects of miRNAs in the pathogenesis of systemic auto inflammatory diseases, from the perspective of familial Mediterranean fever

##### Z. Yeliz Akkaya-Ulum^1^, Zeynep Tavukcuoglu^1^, Ezgi Deniz Batu-Akal^2^, Tayfun Hilmi Akbaba^1^, Hafize Emine Sonmez^2^, Banu Balci-Peynircioglu^1^, Seza Ozen^2^

###### ^1^Medical Biology; ^2^Pediatric Nephrology and Rheumatology, Hacettepe University, Ankara, Turkey

####### **Correspondence:** Tayfun Hilmi Akbaba

**Introduction:** Systemic Auto Inflammatory Diseases (SAID) is a group of rare and hereditary periodic fever syndromes with recurrent inflammatory involvement developing in the absence of infection. Among same SAID patients, phenotypic heterogeneity is common, and modifier mechanisms such as epigenetic factors may be considered as one of the reason of these variations. MicroRNAs (miRNAs), a type of epigenetic mechanisms, are regulated in most of the biological processes like inflammation.

**Objectives:** This study aimed to explore the potential effect of miRNAs in the auto inflammation mechanism seen in SAID from the perspective of Familial Mediterranean fever (FMF) which is the most common auto inflammatory disorder.

**Methods:** The expression levels of miRNAs were analyzed by miRNA array, performed on whole blood RNA samples from healthy controls, homozygous FMF patients with severe phenotype, homozygous FMF patients with mild phenotype, carriers who displayed the disease phenotype, healthy carriers and other rare SAIDs, in pediatric term. The raw data was analyzed by Multi Experiment Viewer (MeV) and TAC programs. Then we performed pathway analyses using DAVID v6.8. and Panther analysistools. Candidate miRNAs shown to be related with inflammatory pathways by bioinformatics analysis, are further studied functionally for their possible effect on expression levels of inflammatory genes, caspase I activation and cell migration (transwell and wound healing experiments) in SW982 (synovial fibroblast) cell lines.

**Results:** The four patient groups were compared in between and miR-30e-3p, miR-374b-5p, miR-329-3p, miR-29c-3p, miR-25-5p were found to be significantly down regulated in the patient groups. The expression levels of these miRNAs were validated with qRT-PCR. All these miRNAs were found to be known regulators in TGF-beta and Toll-like receptor signaling pathway, apoptosis and actin cytoskeleton regulation by bioinformatics. After pre-miR transfection of miR-30e-3p, miR-374b-5p, miR-29c-3p; expression levels of inflammatory genes (IL-1β, IL-18, TNF-α, TGF-β) were decreased, caspase I activation and cell migration ratio were significantly decreased (p<0.05).

**Conclusion:** Functional results of this study showed that these miRNAs may possibly have an anti-inflammatory effect and can regulate the expression of genes found in inflammatory pathways. The investigation of potential target genes of these miRNAs by bioinformatics tools and 3’ UTR luciferase assay is underway. The results of this study will be informative for understanding and investigating the possible effect of miRNAs in other auto inflammatory diseases.

This project has been funded by E-RARE-3 project (INSAID, grant 003037603) and The Technical and Scientific Research Council of Turkey (TUBITAK), Grant number: TUBITAK 1001-SBAG 315S096.

Keywords: SAID, FMF, epigenetics, miRNA, inflammation.

**Disclosure of Interest**

None Declared

#### P1052 MIR-197 regulates inflammation in monocytes and synovial fibroblasts by targeting IL1R1

##### Yeliz Z. Akkaya Ulum^1^, Zeynep Tavukcuoglu^1^, Tayfun Hilmi Akbaba^1^, Engin Yilmaz^2^, Banu Balci-Peynircioglu^1^

###### ^1^Medical Biology, Hacettepe University, Ankara; ^2^Medical Biology, Acibadem Mehmet Ali Aydinlar University, Istanbul, Turkey

####### **Correspondence:** Yeliz Z. Akkaya Ulum

**Introduction:** Familial Mediterranean Fever (FMF); is an autosomal recessively inherited autoinflammatory disease. FMF is caused by the mutations in the Mediterranean Fever (MEFV) gene which encodes the pyrin protein. In many studies, pyrin has been implicated in important cellular processes like apoptosis, cytoskeleton dynamics, signal transduction, and inflammation. Recent studies have shown that epigenetic control mechanisms, particularly non-coding RNAs, may play a role in the pathogenesis of autoinflammation. microRNAs (miRNAs) are small non-coding RNAs that play critical roles in regulating host genome expression at the post-transcriptional level. Dysregulated miRNA expression patterns have been documented in a broad range of diseases including cancer, inflammatory, and autoimmune diseases.

**Objectives:** Phenotypic heterogeneity seen in FMF disease indicated that FMF is not a simple monogenic disease. Therefore it has been suggested that epigenetic factors can be one of the reason for the variations.

**Methods:** Previously we identified miR-20a-5p and miR-197-3p as significantly differentially expressed among healthy controls (-/-) and patients (M694V/M694V). The validation of differentially expressed miRNAs was done by quantitative reverse transcription polymerase chain reaction (qRT-PCR). miRNA target genes were determined in miRWalk, the database on predicted and validated miRNA targets. Then pathway analysis was performed in DAVID. For functional assays, SW982 (synovial fibroblast) and THP-1 (monocyte) cell lines were cultured and transfected with miR-197 mimic and scramble control. After transfection, cytokines expression levels (*MEFV, IL-1b, IL-18, TNF-α, TGF-β*), caspase I activity, apoptosis and cell migration rate were determined. Migration was analyzed in two ways by Transwell migration chamber and wound healing assays. For target gene studies, 3’UTR lusiferase activity assay was done.

**Results:** qRT-PCR analysis confirmed the results of the miRNA profiling for 2 miRNAs. From two of them; miR-20a showed induction and the other one; miR-197 showed reduction in the homozygote group compared to controls (Akkaya-Ulum et al., 2017).

For functional studies, after pre-miR-197 transfection, the expression levels of cytokines were decreased, cells showed less migration and caspase 1 activity. There was no effect in apoptosis. These results showed that miR-197 could have an anti-inflammatory effect by causing less migration and cytokine secretion. miR-197 was found to bind to the interleukin-1beta (IL-1β) receptor, type I (IL1R1), which is one of the key molecules of the inflammatory pathways.

**Conclusion:** These findings provide evidence that miR-197 may play role in FMF pathogenesis. Therefore, this study may contribute to understand the inflammatory process seen in FMF disease, as well as to development of the new drug targets and biomarkers in addition to the existing colchicine and similar treatments.

As miRNA-based therapeutics are promising approaches for treating autoinflammatory diseases, we are planning to continue to study on potential therapeutic usage of miR-197 in mouse model of FMF disease.

This study was supported by The Scientific and Technological Research Council of Turkey TUBITAK 1001-SBAG Project Number: 214S106 and Hacettepe University Scientific Research Projects Coordination Unit, Thesis Support, PhD Project Number: TDK-2017-16253 and BAP Comprehensive Project Number: 013D05101005 and Turkey Rheumatology Association.

**Keywords:** Familial Mediterranean Fever, inflammation, microRNA, miR-197, IL1R1.

**Reference**

Akkaya-Ulum YZ, Balci-Peynircioglu B, Karadag O, Eroglu FK, Kalyoncu U, Kiraz S, et al. Alteration of the microRNA expression profile in familial Mediterranean fever patients. Clin Exp Rheumatol. 2017 Nov-Dec;35 Suppl 108(6):90-94.

**Disclosure of Interest**

None Declared

#### P1053 Comparison of FMF patients with age of onset before 20 versus 40 years and over

##### Okan Aydin, Serdal Ugurlu, Huri Ozdogan

###### Division of Rheumatology, Department of Internal Medicine, Cerrahpasa Medical Faculty, University of Istanbul - Cerrahpasa, Istanbul, Turkey

####### **Correspondence:** Okan Aydin

**Introduction:** Familial Mediterranean fever (FMF) isa disease withan onset before 20 years of age in 90% of the patients.However late onset FMF defined as age of onset over 40 years is being recognised more frequently.

**Objectives:** To better define patients with FMF who had their first attack before age 40 and compare them with early onset patient group in Turkish population

**Methods:** The files of 2180 FMF patients followed in a single center between 2008-2017 who have fulfilled Tel-Hashomer criteria, were reviewed with regard to age of onset 40 years and over (index patients, Group 1).For control purposesfiles before and after the index patients were browsed and

first patients with an onset before age 20 years (Group 2) were included. The demographic, clinical and geneticcharacteristics are compared between these 2 subgroups.

**Results:** Patients with an onset after 40 years consisted 2.7% of our FMF population. 50 of the 59 patients with an onset 40 yearsor over were re-evaluated and compared with early onset group consisting of 100 patients (Table 1).The delay in diagnosis, and disease durationwere significantly longer and number of patients with M694V homozygosity and M694V allele frequencywere significantly more frequent among group 2. In general, phenotypes of both onset groups were similar, the only significant differences being the frequency of fever and myositis which were less common amonggroup 1. Also response to colchicine was more pronouncedingroup 1. One other interesting observation was the low incidence ofamyloidosis in a group with such a significant delay in diagnosis and thus treatment.

**Conclusion:** FMF should be included among the differential diagnosis of patients over 40 years of age with recurrent autoinflammatory manifestations. Less than 3% of FMF patients experience their first attacks after 40 years of age. The frequency of M694V is significantly less in the late onset group, pointing out a milder disease.

**Disclosure of Interest**

None Declared

**Table 1 (abstract P1053).** Demographic, clinical and genetic features of the study groups

	≥40 years n=50	≤20 years n= 100	p
Sex (F:M); present age (mean±SD) (yr)	32:18; **57.2±7.9**	62:38; **31.8±9.1**	NS; **<0.001**
Mean age at onset,(mean±sd) (yr)	**45.6±5.2**	**8.7±4.8**	**<0.001**
Mean age at diagnosis (mean ±sd) (yr)	**50.4±7.3**	**19.1±11.2**	**<0.001**
Delay in diagnosis (mean ±sd) (yr)	**4.8±5.5**	**10.4±11.8**	**<0.001**
Mean disease duration (mean ±sd) (yr)	**11.5±6.4**	**23.1±10.8**	**<0.001**
Abdominal pain, n (%)	44(88)	89(89.0)	NS
Chest pain, n (%)	7(14.0)	27 (27.0)	NS
Fever, n (%)	**30(60.0)**	**81(81.0)**	**0.005**
Arthritis, n (%)	12(24.0)	33(33.0)	0.25
Myalgia, n (%)	**1(2.0)**	**12(12.0)**	**0.04**
Amyloidosis, n (%)	1(2.0)	3(3.0)	NS
Positive family history, n (%)	33(68.7)	62 (65.2)	NS
Response to colchicine, n (%)	**37(82.2)**	**93 (94.8)**	**0.014**
M694VHomozygous, n (%)	**2(4.5)**	**23(25.8)**	**0.003**
N of M694Vallelles	**24( 48 )**	**82 (82)**	**0.014**
No mutation, n (%)	3(6.8)	2(2.2)	NS

#### P1054 What history of appendectomy will tell us about the course of familial Mediterranean fever in adulthood?

##### Erdal Bodakçi^1^, Nazife S. A. Bilge^1^, Nuh Ataş^2^, Berkan Armağan^3^, Hasan Satış^2^, Alper Sarı^3^, Hakan Babaoğlu^2^, Gözde K. Yardımcı^3^, Reyhan B. Salman^2^, Levent Kılıç^3^, Mehmet A. Öztürk^2^, Berna Göker^2^, Seminur Haznedaroğlu^2^, Umut Kalyoncu^3^, Abdurrahman Tufan^2^, Timuçin Kaşifoğlu^1^

###### ^1^Deparment of Internal Medicine, Division of Rheumatology, Eskişehir Osmangazi University Faculty of Medicine, Eskişehir; ^2^Deparment of Internal Medicine, Division of Rheumatology, Gazi University Faculty of Medicine; ^3^Deparment of Internal Medicine, Division of Rheumatology, Hacettepe University Faculty of Medicine, Ankara, Turkey

####### **Correspondence:** Erdal Bodakçi

**Introduction:** Peritonitis attacks of FMF usually requires emergency medical admissions and it’s hard to distinguish a typical attack from surgical causes of acute abdomen. Therefore, unrevealing abdominal surgery history, particularly appendectomy, is very common in patients with FMF. However, history of appendectomy might also give some clues about the disease course of FMF.

**Objectives:** to determine whether history of appendectomy is associated with disease course of FMF in adulthood.

**Methods:** All patients recruited from FMF in Central Anatolia (FiCA) cohort, comprising 970 (mean age 35.3±12 years, 61.5%female) adult subjects. All patients fulfilled Tel Hashomer criteria. Demographic data, FMF disease characteristics, co-morbid conditions, past medical history including surgeries, disease complications were meticulously questioned and laboratory features and genotype data (if available) were recruited from patient files. Disease severity and FMF associated damage were assessed with International Severity Scoring System (ISSF) for FMF and Autoinflammatory Disease Damage Index (ADDI), respectively.

**Results:** Appendectomy history was evident in 240 (24.7%) subjects.Peritonitis (4.4±6.7 vs 2.9±4.3 attacks/per year, p<0.001) and pleuritis (3.9±5.2 vs 2.8±4.4 attacks/per year, p=0.03) attacks were more frequent in appendectomy performed (AP) group than appendix intact (AI) group. However, there were no difference between AP and AI groups for the attack frequencies of musculoskeletal and skin components. Considering all types of attacks, AP group had more attacks (6.4±8.2 vs 4.3±6.6 attacks, p<0.001), despite they had used more higher doses of colchicine (1.43±0.6 mg/day vs 1.27±0.5 mg/day, p=0.002). ISSF scores were also higher in AP group 3.13±1.68 vs 2.73±1.48, p=0.001). Colchicine nonresponse were more prevalent in AP group as well (15.1% vs 6.7%, pearson X^2^=20.2, p<0.001). However, ADDI damage scores were similar between AP and AI groups.

**Conclusion:** Appendectomy history is common in FMF patients and associated with frequent serositis attacks in adulthood. These patients require more colchicine doses with a lower response rate. Hence, history of appendectomy would be a worthy clue for the management of FMF patients.

**Disclosure of Interest**

None Declared

#### P1055 Complement endorse the pathogenesis in autoinflammation

##### Juergen Brunner^1^, Wilfried Posch^2^, Doris Wilflingseder^2^

###### ^1^Pediatrics; ^2^Division of Hygiene and Medical Microbiology, Medical University Innsbruck, Innsbruck, Austria

####### **Correspondence:** Juergen Brunner

**Introduction:** The complement system represents a major part of the innate immune system, consisting of more than 30 different proteins in plasma and on cell surfaces and can be activated through three different pathways.Inflammasomes are also part of the innate immune system.A group of disorders in inflammasomes have been associated with autoinflammatory diseases (AIDs). Familial cold autoinflammatory syndrome (FCAS), Muckle-Wells syndrome (MWS) and chronic infantile neurological, cutaneous and articular syndrome/neonatal onset multisystem inflammatory disease (CINCA/NOMID) were originally described as three distinct diseases. After the identification of their common genetic origin, i.e. mutations in the NLRP3 gene on chromosome 1q44, they are perceived as a continuum of one disease entity and labelled cryopyrin-associated periodic syndromes (CAPS).

**Objectives:** Aim of this preliminary study in a patient with MWS was to find a correlation between the complement system and a disorder of autoinflammation.

**Methods:** PBMCs (peripheral blood mononuclear cells) were isolated from blood of a healthy donor and of an individual suffering from MWS by density gradient centrifugation using a Ficoll Paque Premium (GE Healthcare). After washing, PBMCs were incubated with anti-human CD14 Magnetic Beads (BD) to obtain CD14+ monocytes. These were stimulated by addition of cytokines (IL-4 and GM-CSF) for five days to generate immature moDCs (iDCs), which were used for cytokine ELISAs and flow cytometric analyses. IL-6 and IL-1β cytokine ELISAs were performed according to the manufacturer (Biolegend) following stimulation of cells using either LPS or differentially complement opsonized HIV-1. Phenotypical characterization of pathogen-exposed DCs was performed by analyzing characteristic surface markers (CD11c, DC-SIGN, CD86) by multi-color flow cytometry.

**Results:** IL-1β production of iDCs is higher in the patients cells than in the cells of the healthy donor. However, the most significant difference was shown in complement opsonized iDCs. DC-SIGN is higher expressed in complement opsonized iDCs in patient cells compared to cells of a healthy donor (37,12% v28,64%). DC-SIGN is also higher expressed in the iDCs of the MWS patient after stimulation with LPS.

**Conclusion:** The complement system may play an important role in the development of an proinflammatory milieu in patients with disorders of autoinflammation. The phenomenon shown in a patient with MWS has to be reproduced in more MWS patients a well as in patients with other disorder of autoinflammation.

**Disclosure of Interest**

None Declared

#### P1056 Generation of ADA2 genetic knockout in a myeloid cell line using CRISPR/CAS9 genome editing: an in vitro cell line model to study DADA2

##### Marina S. Casimir, Ying Hong, Paul Brogan, Despina Eleftheriou

###### Infection, Inflammation and Rheumatology, UCL Great Ormond Street Institute of Child Health, London, United Kingdom

####### **Correspondence:** Marina S. Casimir

**Introduction:** Deficiency of adenosine deaminase type 2 (DADA2) is an autosomal recessive genetic disease causing systemic inflammation and vasculitis resembling polyarteritis nodosa, caused by loss of function mutations in the *ADA2* gene. We are curently exploring an alternative treatment strategy of curing DADA2 using gene therapy. An important first step for this line of work was to develop a cell line model, with abrogation of ADA2 protein and enzyme expression and to determine whether the immunophenotype and characteristics of this cell line mimic the phenotype of primary cells derived from DADA2 patients.

**Objectives:** To develop an *ADA2* knockout (KO) monocyte cell line (THP-1) derived using Clustered Regularly-Interspersed Small Palindromic Repeats (CRISPR)/CAS9 and to confirm that this KO has comparable immunophenotype to monocytes from DADA2 patients (reduced ADA2 expression; M1/M2 skewing and pro-inflammatory cytokine production).

**Methods:** A plasmid delivered CRISPR/CAS9 system was used to generate the *ADA2* KO THP-1 cell line. ADA2 enzyme activity was assessed using a modified commercially available assay and ADA2 protein expression using immunoblotting. Cells were treated with PMA to induce differentiation into macrophages before polarisation into M1 through LPS/ INF-γ stimulation; and M2 polarisation through IL-4, IL-10 and IL-13 stimulation. Immunophenotyping was assessed using gene expression analysis by qPCR. We performed similar experiments in monocyte-derived macrophages (MDM) from 4 DADA2 patients using the same protocol as for the cell line. Cytokine production was evaluated using MSD electrochemiluminescence.

**Results:** We first confirmed effective knockout of *ADA2* at the genetic and protein level and a complete loss of ADA2 enzyme activity in culture supernatants when compared to scramble control THP-1 cells (p<0.01).M1 polarised *ADA2* KO THP1 cells exhibited increased activation of pro inflammatory markers such as TNF-α (p<0.001), CXCL-10 (p<0.001), STAT-1 (p<0.01) and IL-1β (p<0.001) when compared to control THP-1 cells. The M1/M2 ratio was reversed in the *ADA2* KO THP-1 cell line compared to the wild type THP-1 cells. In MDM cells from DADA2 patients, we observed similar induction of the pro-inflammatory pathway as indicated by increased TNF-α and IL-1β gene expression. Release of TNF-α in culture supernatants from M1 polarised cells was also enhanced for both the *ADA2* KO THP-1 cell line and in cells derived from DADA2 patients.

**Conclusion:** We have generated an effective *ADA2* genetic KO myeloid cell line using a CRISPR/Cas9 system and confirmed that these cells have a complete loss of ADA2 enzyme activity and a comparable immunophenotype to monocytes from DADA2 patients. This in vitro system can now be used to examine the possibility to reverse the defects associated with DADA2 using gene therapy strategies.

**Disclosure of Interest**

None Declared

#### P1057 Promising blood test for diagnosis and treatment options in patients with suspected chronic auto-inflammatory syndromes

##### Anne-Laure N. Chetaille^1^, Nathalie Pagé^2^, Marie-Pier Longchamps^2^, Louis Bessette^2^, Laetitia Michou^2^, Paul Fortin^2^, Philippe Tessier^2^, Martin Pelletier^2^

###### ^1^Rhumatologie Adulte et Pédiatrique; ^2^CHU de Québec-Université Laval, Québec, Canada

####### **Correspondence:** Anne-Laure N. Chetaille

**Introduction:** Diagnosis and treatments of Auto-Inflammatory Syndromes (AIS) patients are challenging as clinical symptoms are non-specific especially compared with Systemic Autoimmune Rheumatic Diseases (SARD) and detection rate of genetic mutations in patients with high suspicion for AIS is low. Even if a mutation is found, genotypes don’t correlate with phenotypes neither with the response to treatments. As a consequence, patients can be misdiagnosed, receive inappropriate treatment, leading to severe complications and substantial socio-economic costs.

**Objectives:** The cytokines abnormally secreted by each individual are unknown. We hypothesized that the secretion of cytokines by peripheral blood mononuclear cells (PBMC) could be an indicator of the disease and could guide the treatment to the most suitable anti-cytokine. Quantification of the secretome of leukocytes may guide diagnosis and treatment of AIS patients.

**Methods:** Plasma and peripheral blood mononuclear cells (PBMCs) were isolated from healthy controls, suspected AIS patients, and rheumatoid arthritis (RA) and systemic lupus erythematosus (SLE) patients from the CHU de Québec SARD Biobank Repository Database (SBRD). PBMCs were stimulated with well-known immune activators to trigger inflammasome activation, and cytokine levels were analyzed in the plasma and the supernatants by multiplex assays.

**Results:** Plasma cytokine levels were similar and did not allow to discriminate between suspected AIS patients, SARD patients or healthy volunteers. PBMCs, on the contrary, had a distinct profile of cytokine secretion between groups. PBMCs from AIS patients spontaneously secreted more IL-1a, IL‑12 and IL-18 than cells from SARD patients or healthy control subjects, as well as IFNg in response to NLRP3, AIM2 and pyrin inflammasome activation. PBMCs from some suspected AIS patients secreted excessive IL-1a or IL-1b in the absence of the antagonist IL‑1RA, suggesting blockers of IL-1 could be used as biotherapeutic approach. PBMCs from other suspected AIS patients secreted high levels of IFNg, IL-12 and IL‑18, pointing to the usefulness of treatments such as Janus kinase small molecule inhibitors, and may be the anti-IL‑12/IL-23 p40 antagonist and/or the novel anti-IL-18 in clinical development.

**Conclusion:** This study demonstrates that analysis of leukocytes’ secretome is reliably more sensitive than serum to reveal cytokine signatures and to predict biologic treatment options in patients with suspected chronic AIS. Quantification of leukocytes’ secretome allows personalized medicine by guiding diagnosis and treatment options of AIS patients.

**Disclosure of Interest**

None Declared

#### P1058 Specific dysbiosis associated with familial Mediterranean fever complicated or not with AA amyloidosis

##### Samuel Deshayes^1^, Soraya Fellahi^2^, Jean-Philippe Bastard^2^, Jean-Marie Launay^3^, Jacques Callebert^3^, Thibault Fraisse^4^, David Buob^5^, Jean-Jacques Boffa^6^, Irina Giurgea^7^, Charlotte Dupont^8^, Sarah Jegou^9^, Marjolène Straube^9^, Alexandre Karras^10^, Achille Aouba^1^, Gilles Grateau^4^, Harry Sokol^11^, Sophie Georgin-Lavialle^4^ and AA Amyloidosis Study Group

###### ^1^Internal Medicine, CHU Côte de Nacre, Caen; ^2^Biochemistry, Hôpital Tenon; ^3^Biochemistry, Hôpital Lariboisière; ^4^Internal Medicine; ^5^Anatomopathology; ^6^Nephrology, Hôpital Tenon; ^7^Medical Genetics, Hôpital Trousseau; ^8^Reproduction Biology, Hôpital Tenon; ^9^AP-HP Laboratoire des Biomolécules (LBM), UPMC Université Paris 06; ^10^Nephrology, Hôpital Européen Georges Pompidou; ^11^Gastroenterology, Hôpital Saint-Antoine, Paris, France

####### **Correspondence:** Samuel Deshayes

**Introduction:** Familial Mediterranean fever (FMF) can be complicated by inflammatory (AA) amyloidosis, but the reason why only some patients will develop amyloidosis is not completely understood.

**Objectives:** To assess gut microbiota composition and inflammatory markers in FMF patients complicated or not by AA amyloidosis.

**Methods:** We included 34 FMF patients without AA amyloidosis, 7 FMF patients with AA amyloidosis, 19 patients with AA amyloidosis from another origin, and 26 controls. The gut microbiota was studied by 16S ribosomal ribonucleic acid gene sequencing on an Illumina MiSeq platform. Associations between bacterial taxa and clinical phenotype were evaluated using the multivariate association with linear models (MaAsLin) statistical method. Blood dosages of interleukin (IL)-1β, IL-6 and tumor necrosis factor-α were carried out by enzyme-linked immunosorbent assay.

**Results:** Compared to healthy subjects, significant decreases in α-diversity were noted in FMF patients without amyloidosis and in patients with non-FMF-related AA amyloidosis. β-diversity analysis also showed significant differences between healthy controls and two same groups. After multivariate association testing with the MaAsLin statistical method to control for the effects of potential confounding factors (such as age, gender, body mass index and treatments), several operational taxonomic units belonging to the Clostridiales order were associated with FMF. Moreover, 2 operational taxonomic units belonging to the Clostridiales order were overrepresented in FMF-related AA amyloidosis compared to FMF patients without AA amyloidosis.

All studied groups had higher blood levels of IL-1β, IL-6 and tumor necrosis factor-α than controls.

**Conclusion:** FMF was associated with a gut microbiota dysbiosis characterized by a decreased α-diversity and a significant alteration in composition. Although these results are descriptive, they suggest that the gut microbiota might be involved in the clinical expression of FMF. In FMF patients, amyloidosis was independently associated with a specific alteration in the microbiota composition, suggesting that the gut microbiota may play a role in AA amyloidosis pathogenesis. These data need to be further consolidated in mechanistic and interventional studies.

**Disclosure of Interest**

None Declared

#### P1059 Proinflammatory cytokines induced by PBMCS from a patient with a NLRP1 variant showing severe corneal intraepithelial dyskeratosis

##### Troels Herlin^1^, Sofie E. Jørgensen^2^, Christian Høst^1^, Mette Christiansen^3^, Dorte A. Larsen^4^, Trine H. Mogensen^5^

###### ^1^Pediatrics, Aarhus University Hospital; ^2^Biomedicine, Aarhus University; ^3^Clinical Immunology; ^4^Ophthalmology; ^5^Infectious diseases, Aarhus University Hospital, Aarhus, Denmark

####### **Correspondence:** Troels Herlin

**Introduction:** Corneal intraepithelial dyskeratosis is an extremely rare autosomal dominant inherited disorder with the classical form known as hereditary benign intraepithelial dyskeratosis (HBID). Variants of the nucleotide-binding leucine-rich repeat containing purine domain 1, *NLRP1*gene, have recently been associated with autoinflammatory disorders with arthritis, vitiligo and dyskeratosis, including HBID.

**Objectives:** In a boy with severe HBID, with a variant p.A59P in the autoinhibitory PYD domain of NLRP1, to examine the level of NF-kB activation and proinflammatory cytokines in stimulated PBMCs.

**Methods:** \5-year-old boy with severe inflammation of the gingival mucosa with premature loss of several teeth, painful corneal and conjunctival inflammation of both eyes leading to marked photophobia, corneal opacification and loss of vision. He had mild eczema, but no dyskeratosis of the skin. He is the only child of non-consanguineous Danish parents. Grandfather’s brother and father’s grandfather both had hyperkeratotic dermatosis and swollen gingival mucosa but no eye inflammation.

*NLRP1*Sanger sequencing was performed. Peripheral blood mononuclear cells (PBMCs) from patient and 3 healthy controls were stimulated with NF-kB inducers (TNFa and LPS or left untreated. Phosphorylated IkBa, as a measure of NF-kB activation, was measured by Luminex technology. PBMCs were stimulated with different ligands (TNFa and LPS and muramyl dipeptide (MDP) or left untreated for 16 hrs. Proinflammatory cytokines (IL-1b, IL-6, IL-18, and TNFa) were measured in the supernatants using Mesoscale U-plex multiplex assays.

**Results:** Sanger sequencing identified a heterozygous variant (c.175G>C, p.A59P) in the autoinhibitory PYD domain of NLRP1. The variant has a high CADD score of 24.1 predicting a high likelihood of deleteriousness and is not present in the gnomAD database. The sequencing revealed other variants (p.L155H, p.V1063M and p.M1188V) with high minor allele frequencies, most likely benign. Family analysis showed that A59P and L155H were carried by the father and the grandfather’s brother but none by the mother.

Patient PBMCs demonstrated significantly increased levels of Ser32/36 phosphorylated IkBa in response to TNFa and LPS stimulation compared to controls, indicating increased NF-kB activation. In response to especially MDP, but also TNFa stimulation, patient PBMCs expressed extremely high levels of IL-1b and IL-6 and increased IL-18 levels in unstimulated PBMCs compared to controls. Clinically and biochemically the patient responded well to monthly treatment with subcutaneous canakinumab (5 mg/kg) with rapid improvement of the gingivitis, and ophthalmologically administration of diluted anakinra (2.5% solution) as 1 eye-droplet three times per day had a dramatic effect on ocular pain and inflammation.

**Conclusion:** We here report A59P NLRP1 as a novel defect causing inflammasome hyperactivation/autoinflammatory disease and a clinical picture including severe inflammation of the gingival mucosa and keratitis in a 5-year-old boy with a family history suggesting HBID. Patient PBMCs exhibited increased NF-kB activation and elevated levels of IL-1 and IL-6 in response to proinflammatory stimuli. Thus, our results enabled us to select relevant targeted therapy deploying IL-1binhibition with systemic canakinumab and topical anakinra.

**Consent for publication has been obtained from patient**

Yes

**Disclosure of Interest**

None Declared

#### P1060 Gain-of-function mutation of NOD2 impairs its ligand specific immune responses in BLAU syndrome patient-derived IPS cells

##### Naotomo Kambe^1^, Nhung T. M. Ly^1^, Megumu K. Saito^2^, Hiroyuki Okamoto^1^

###### ^1^Dermatology, Kansai Medical University, Hirakata, Osaka; ^2^Clinical Application, CiRA, Kyoto University, Kyoto, Japan

####### **Correspondence:** Naotomo Kambe

**Introduction:** NOD2 is crucial for innate immune response and mainly expressed in hematopoietic lineage cells, especially in monocytic cells. On the recognition of its ligand, muramyl dipeptide (MDP), NOD2 leads to activation of NF-κB pathway, causing upregulation of pro-inflammatory cytokines.

**Objectives:** Mutations of NOD2 have been associated with Blau syndrome, but the details regarding mechanisms associated mutant NOD2 leads to granuloma formation are still unclear. By using iPS cells derived from the patients, we tried to reveal the molecular mechanism of Blau syndrome.

**Methods:** The iPS cells with R334W mutation in NOD2 have been established from a Blau patient and we corrected the mutation of the iPS cells into wild type (WT) by using a CRISPR-Cas9 system. These isogenic iPS cells were differentiated into monocytic cell lineages, then transfected with the lentiviral vectors and maintained in the StemPro-34 medium with M-CSF and GM-CSF.

**Results:** IFNγ induced the same upregulation of NOD2 in iPS-derived monocytes with WT and those with R334W. Without MDP stimulation, pro-inflammatory cytokine production was only found in those with mutant NOD2, suggesting that Blau-associated NOD2 is gain-of-function mutation. However, after stimulating with MDP, the R334W mutant cells showed NF-κB pathway activation and cytokine secretions less than WT cells even with or without IFNγ treatment. On the other hand, both the cell groups showed the comparative immune response to TNFα and LPS treatment, unrelated to NOD2 mutation.

**Conclusion:** The response to MDP by mutant NOD2 was selectively impaired in Blau syndrome, that may incompletely lead to neutrophilic inflammation but compensatively induce granuloma formation in host defense.

**Disclosure of Interest**

None Declared

#### P1061 The role of DNA methylation for disease severity in patients with heterozygous mutations in the Mediterranean fever gene MEFV

##### Julie Krainer^1^, Walter Pulverer^1^, Dirk Foell^2^, Seza Özen^3^, Andreas Weinhäusel^1^

###### ^1^AIT - Austrian Institute Of Technology, Vienna, Austria; ^2^Pediatric Rheumatology & Immunology, University Children's Hospital, Muenster, Germany; ^3^Department of Pediatric Rheumatology, Hacettepe University, Ankara, Turkey

####### **Correspondence:** Julie Krainer

**Introduction:** Familial Mediterranean Fever (FMF) is the most common hereditary SAID, with a high prevalence in patients of eastern Mediterranean decent. It is known as an autosomal dominant disease with mutations in the MEFV gene that encodes for Pyrin, an important innate immunity regulator. However, some heterozygous individuals also show an FMF phenotype, which leads to the assumption that other modifying factors lead to a manifestation of the phenotype^1^. In recent years, DNA methylation has demonstrated suitable as biomarker and its potential for disease diagnosis by several studies. DNA methylation plays an important epigenetic regulatory effect on gene expression, and aberrances can result in pathological disease states.

**Objectives:** The main goal of this study was to evaluate the DNA methylation in patients carrying heterozygous mutations in the MEFV gene but show different phenotypes. We hypothesis that alterations in DNA methylation can add important and valuable information about the disease etiopathogenesis.

**Methods:** The study included 32 patients, 12 of the patients show a typical FMF phenotype and SNP analysis showed a heterozygous mutation, 9 patients showed similar heterozygous mutations but lack FMF characteristics and are healthy. We also included 12 control samples without any mutation in the MEFV gene.

We performed a genome wide DNA methylation analysis using Illumina’s EPIC BeadArray, interrogating over 850.000 CpG sites at single C resolution covering the first exon of >84% of all Genes^2^. The results were processed and normalized using the R package Champ^3^ and group comparisons were performed with limma^4^.

**Results:** In total four different group comparisons were calculated including Heterozygous Healthy vs. Heterozygous Disease, Heterozygous Healthy vs. Control, Heterozygous Disease vs. Control and Heterozygous (Disease + Healthy) vs. Control. Each comparison revealed over 30000 significant cpgs (p<0.05) where between 28 and 75 cpgs showed at least 15% differences in mean methylation between the groups. Eighty of the significant cpgs were present in every group comparison covering 62 unique genes. During hierarchical clustering the three clusters separated visibly, where two Heterozygous Healthy patients cluster within the control samples. A group comparison between the two Heterozygous groups revealed 71 differentially methylated sites (p<0.05, difference ≥ 15%), able to separate the two groups.

**Conclusion:** During our experiments we were able to detect differentially methylated sites separating Heterozygous Healthy and Heterozygous Disease patients. We hypothesize that the identified CpG sites can contribute to provide information to the etiopathogenesis of FMF in general.

**REFERENCES**

[1] Sönmez et al.. Familial Mediterranean fever. Current perspectives. In: Journal of Inflammation Research 9; 2016. S. 13–20. DOI: 10.2147/JIR.S91352.

[2] Pidsley et al. “Critical evaluation of the Illumina MethylationEPIC BeadChip microarray for whole-genome DNA methylation profiling.” Genome Biology 17 (1), S. 208; 2016. DOI: 10.1186/s13059-016-1066-1.

[3] Morris et al. “Champ: 450k chip analysis methylation pipeline.” Bioinformatics, 30(3), 428-30; 2014. DOI: 10.1093/bioinformatics/btt684.

[4] Ritchie ME et al. “limma powers differential expression analyses for RNA-sequencing and microarray studies.” Nucleic Acids Research, 43(7), e47; 2015. DOI: 10.1093/nar/gkv007

**Disclosure of Interest**

None Declared

#### P1062 Differential kinetic of actin polymerization in neutrophil from patients with familial Mediterranean fever

##### David Poghosyan^1^, Anush Martirosyan^1^, Nune Mkrtchyan^2,3^, Sona Margaryan^1^, Susanna Ghonyan^1^, Gayane Amaryan^2,3^, Gayane Manukyan^1^

###### ^1^Laboratory of Molecular and Cellular Immunology, Institute of Molecular Biology; ^2^Arabkir Medical Centre, Institute of Child and Adolescent Health, National Pediatric Centre for Familial Mediterranean Fever; ^3^Yerevan State Medical University, Yerevan, Armenia

####### **Correspondence:** Gayane Manukyan

**Introduction:** Seemingly unprovoked trafficking of neutrophils to the serosal/synovial membranes is a key event in acute inflammatory attacks of familial Mediterranean fever (FMF). Migratory events behind abnormal trafficking of the cells remain largely unknown.

**Objectives:** The aim of the present study is to analyze kinetic of actin polymerization and migration rate of neutrophils from colchicine naïve and colchicine receiving FMF patients.

**Methods:** FMF patients in acute attack (colchicine naïve A-FMF), FMF patients in remission period (receiving colchicine, R-FMF), and healthy controls (HD) were enrolled in the study. We have measured the amounts of F-actin in untreated and pre-treated with colchicine neutrophils induced by fMLP at 5s, 15s, 30s, 60s, 120s, and 180s time points *in vitro*. Actin polymerization was investigated by flow cytometry using FITC-Phalloidin. Neutrophils migration to fMLP was assessed using Transwell cell migration assay.

**Results:** Neutrophils from A-FMF displayed higher migration rate compared to R-FMF and HD (*P*< 0.05). R-FMF patients, who were receiving colchicine, displayed the lowest actin polymerization activity in neutrophils at all-time points. The cells from FMF patients in A-FMF stimulated with fMLP displayed a maximal F-actin polymerization earlier (at 5s) than in R-FMF (at 30s) and HD (at 15s) (*P*< 0.05). In opposite, plateau levels of F-actin content were reached earlier in HD neutrophils (after 1 min), while plateau levels in A-FMF and R-FMF cells were reached later (after 2 min). Colchicine-pretreated cells in all studied groups reached maximal F-actin polymerization later than in untreated with colchicine cells, namely at 15s in A-FMF and 30s in R-FMF and HD groups.

**Conclusion:** We have demonstrated actin dysfunctions in neutrophils from FMF patients which might cause defects in neutrophil functioning. The results suggest that colchicine may affect actin polymerization in the cells due to unknown mechanism.

**Disclosure of Interest**

None Declared

#### P1063 CARD15 mutations in an Indian cohort of Blau syndrome

##### Amit Rawat^1^, Deepti Suri^1^, Rajni Kumrah^1^, Sagar Bhattad^2^, Sandesh Guleria^1^, Vignesh Pandiarajan^1^, Ankur Jindal^1^, Anju Gupta^1^, Isabelle Ceccherini^3^, Marco Gattarno^4^, Surjit Singh^1^

###### ^1^Pediatrics, Postgraduate Institute of Medical Education and Research, Chandigarh; ^2^Pediatric Immunology and Rheumatology, Aster CMI Hospital, Bengaluru, India; ^3^Genetica Medica; ^4^U.O.C. Pediatria II - Reumatologia, Istituto Giannina Gaslini, Genoa, Italy, Genoa, Italy

####### **Correspondence:** Amit Rawat

**Introduction:** Blau syndrome is an autosomal dominant disorder resulting from genetic variants in the *CARD15* gene previously known as *NOD2*. It is characterized by a clinical triad of arthritis, uveitis and skin rash. Classic features also include skin lesion with non-caseating granulomas, boggy synovitis or tenosynovitis and camptodactyly. CARD15 gene comprises eleven exons encoding for a 1044 amino acid protein. The protein has N-terminal caspase recruitment domains (CARDs) linked to a nucleotide binding domain (NBD) and C- terminal leucine-rich repeats (LRRs).

**Objectives:** The objective of the study was to determine the underlying genetic variants in our cohort of patients with Blau syndrome and to establish a genotype-phenotype correlation if any.

**Methods:** Genetic variants in CARD15 were determined in 11 of our patients with Blau syndrome. Targeted next- generation sequencing was employed to detect these variants in six patients at the Istituto Giannina Gaslini, Genoa, Italy. In rest of the 5 patients, conventional Sanger sequencing was used. Since most of the variants have been detected in Exon 4, the exon4 was amplified by polymerase chain reaction. Four pairs of specific primers described in Resource of Asian Primary Immunodeficiency Diseases Database were used for amplification of the Exon 4 and the PCR amplifications generated were sequenced using conventional Sanger sequencing.

**Results:** Single nucleotide substitutions resulting in missense variants were detected in all of the eleven patients. Nine of the eleven patients had a recurrent, previously reported missense variant in Exon 4 which encodes for the nucleotide binding domain (NBD). This variant resulting from the substitution of Thymine for cytosine at c.1000; c.1000C>T causes the replacement of Arginine with Tryptophan at codon 344; p.R344W. This variant has been reported in the Infevers database. Three of the nine patients with this variant were related, an affected mother with her son and daughter. Rest of the six were from unrelated families. However, six of the nine patients were from the north Indian state of Haryana, and one was from the adjoining northern state of Punjab. The remaining two patients were from the eastern part of India. Two patients had another missense variant in the Exon 4; p.G299D with replacement of Glycine with Aspartic acid at codon 299. This variant has not been reported in the Infevers database or the Human Gene Mutation Database (HGMD), but it has been reported in the Exome Aggregation Consortium (ExAC).

**Conclusion:** The detection of a similar previously reported missense variant in 9 of the 11 patients might denote a founder mutation especially given the fact that seven of the nine patients with the p.R344W variant were from the same geographical locale in North India. This recurrent variant also provides an opportunity for development of an amplification-refractory mutation system for its detection without resorting to sequencing.

**Disclosure of Interest**

None Declared

#### P1064 Differential activation of the Pyrin inflammasome in monocytes and macrophages predicts the pathological significance of MEFV variants in familial Mediterranean fever (FMF) patients

##### Takeshi Shiba^1^, Takayuki Tanaka^1^, Hiroaki Ida^2^, Misa Watanabe^3^, Haruna Nakaseko^4^, Mitsujiro Osawa^5^, Hirofumi Shibata^1^, Kazushi Izawa^1^, Takahiro Yasumi^1^, Yuri Kawasaki^5^, Megumu K. Saito^5^, Junko Takita^1^, Toshio Heike^6^, Ryuta Nishikomori^1^

###### ^1^Department of Pediatrics, Kyoto University Graduate School of Medicine, Kyoto; ^2^Department of Respiratory, Neurology, and Rheumatology, Kurume University School of Medicine, Fukuoka; ^3^Department of Pediatrics, Toho University School of Medicine, Tokyo; ^4^Department of Infection and Immunology, Aichi Children’s Health and Medical Center, Aichi; ^5^Department for Clinical Application, Center for iPS cell Research and Application, Kyoto University, Kyoto; ^6^Department of Pediatrics, Hyogo Prefectural Amagasaki General Medical Center, Hyogo, Japan

####### **Correspondence:** Takeshi Shiba

**Introduction:** While numerous *MEFV* sequence variants have been reported, the impact of these variants on pyrin inflammasome functions remains unknown.

**Objectives:** To determine the effect of *MEFV* variants on IL-1β production by monocytes and monocyte-derived macrophages from Familial Mediterranean Fever (FMF) patients and to extend this approach to the functional evaluation of rare *MEFV* variants.

**Methods:** Freshly isolated monocytes and monocyte-derived macrophages from patients with typical FMF and healthy donors were analyzed for IL-1β secretion in response to the pyrin inflammasome activator Clostridium difficile toxin A (TcdA). Induced pluripotent stem cell (iPSC) clones derived from FMF patients and healthy donors were differentiated into macrophages and analyzed for pyrin inflammasome activation. Rare *MEFV* variants were expressed in iPSC-derived macrophages.

**Results:** IL-1β secretion by FMF monocytes in response to TcdA was comparable to that of healthy donors, and colchicine inhibited IL-1β secretion from healthy donors, but not from FMF monocytes. FMF macrophages secreted significantly higher levels of IL-1β than healthy donor macrophages, and their IL-1β secretion could be inhibited with colchicine. The characteristics observed in monocyte-derived macrophages were recapitulated in iPSC-derived macrophages. Finally, iPSC-derived macrophages transgenically expressing the N679H variant were functionally similar to those expressing the pathogenic *MEFV* variant M694I, while cells expressing the T577N variant had a similar phenotype as control cells.

**Conclusion:** While FMF monocytes carrying the typical *MEFV* mutations were distinct from healthy monocytes in their unresponsiveness to colchicine, FMF macrophages showed hyperactivation of the pyrin inflammasome and were sensitive to colchicine inhibition. Our novel approach may be useful for identifying *MEFV* variants that cause the typical FMF phenotype according to their capacity to induce pyrin inflammasome activation.

**Disclosure of Interest**

None Declared

#### P1065 Cross-talk between type I IFN pathway and TLR sensing in DNase2 mutated fibroblasts

##### Alessandra Tesser^1^, Elisa Piscianz^2^, Valentina Boz^2^, Giulia Piperno^3^, Federica Benvenuti^3^, Alberto Tommasini^1^

###### ^1^IRCCS Burlo Garofolo, Trieste, Italy; ^2^University of Trieste, Italy; ^3^ICGEB, Trieste, Italy

####### **Correspondence:** Alessandra Tesser

**Introduction:** Cytoplasmic sensing of nucleic acids via cGAS-TBK1 pathway leads to type I interferon (IFN) production. DNase2 deficiency impairs DNA digestion in phagosomes resulting in cGAS-dependent IFN hyper-production (1). However, other sensors like TLRs can stimulate this pathway, as recently shown in animal models (2).

**Objectives:** To investigate the interaction between cGAS and TLRs stimulation in DNAse2-deficient fibroblasts.

**Methods:** Fibroblasts from a subject with DNase2 deficiency and from healthy controls were treated with different Multiplicity Of Infection (MOI) of *E. coli* and concentration of bacterial LPS, 2’3’-cGAMP and Poly(I:C) for 1 hour. After stimulation, phosphorilated-TBK1 was measured by flow-cytometry with intracellular staining.

**Results:** Fibroblasts with DNase2 deficiency responded to an overload of bacteria with a greater activation of the IFN pathway compared to healthy controls. DNase2-mutated cells showed an enhanced IFN response even after stimulation with pure *E. coli* derived-LPS (Tab. 1). Moreover, the combined stimulation of the cGAS-STING pathway and on TLRs (cGAMP + LPS, cGAMP + Poly(I:C)) resulted in a synergic increase of TBK1 phosphorylation compared with any stimulus alone (Tab. 1).

**Conclusion:** The possibility that the dysregulated IFN pathway in DNase2-deficient fibroblasts may sensitise cells to an increased response to LPS is supported by others preliminary data (not shown) proving that intact STING is necessary for LPS-induced IFN production. Indeed, there are evidences that STING can be activated not only by 2’3’-cGAMP, but also by other molecules collecting the signals from distinct TLRs. Considering these results, we can assume that a dysfunctional IFN pathway could display a “permissive” role towards other stimuli acting on STING-TBK1 signaling. Ongoing experiments are being carried out to clarify the convergence of different TLRs stimuli on the cGAS-TBK1 axis.

1. Rodero MP, et al. Type I interferon-mediated autoinflammation due to DNase II deficiency. Nat Commun. 2017 Dec 19;8(1):2176.

2. Manfredo VS, et al. Translocation of a gut pathobiont drives autoimmunity in mice and humans. Science. 2018 Mar 09:Vol. 359, Issue 6380, pp. 1156-1161.

**Disclosure of Interest**

None Declared

**Table 1 (abstract P1065).** TBK1 phosphorylation assessed by flow-cytometry after infection with different MOI (20, 120, 400) of *E. coli*, single stimulation with LPS (0.5 ug/ml), cGAMP (20 ug/ml) and Poly(I:C) (20 ug/ml), and combined stimulation of cGAS/TBK1 and TLRs pathways (cGAMP + LPS; cGAMP + Poly(I:C); Poly(I:C) + LPS)

	pTBK1 (MFI) Healthy control fibros	pTBK1 (MFI) DNase2 mut fibros
NS	2.9	3
E.coli MOI 120	3.1	7
E.coli MOI 400	3.7	8
LPS	2.3	6.1
cGAMP	3.3	4.3
Poly(I:C)	3.4	5.6
cGAMP + LPS	4.5	7.5
cGAMP + Poly(I:C)	4.5	7.6
Poly(I:C) + LPS	2.8	4.4

NS: not stimulated; MFI: mean fluorescence intensity

#### P1066 Auto-inflammatory diseases (SAIDS) in Western Switzerland: a descriptive study through the JIRcohorte platform

##### Lorenzo Tosetti, Manel Mejbri, Aikaterini Theodoropoulou, Michael Hofer

###### Pediatric Immunology Allergology Rheumatology Unit, University Hospital Lausanne and University Hospital Geneva, Lausanne and Geneva, Switzerland

####### **Correspondence:** Lorenzo Tosetti

**Introduction:** SAIDs are a large and heterogeneous group of inflammatory conditions, including monogenetic and multifactorial diseases, associated with a dysregulation of innate immune system. Early diagnosis and treatment of these conditions are essential to prevent serious complications, in particular the development of amyloidosis. Biological treatments blocking the Interleukins (IL-) 1 and 6 can lead to rapid remission and are expected to improve long-term outcome in these patients. Five of them received an indication to be treated by these medications in Switzerland; therefore, we are interested to evaluate the number of patients who may receive these treatments.

**Objectives:** To describe and estimate the prevalence of FMF, MKD, TRAPS, CAPS and SoJIA in Switzerland.

**Methods:** This is a monocentric, prospective and descriptive cohort study, through the JIRcohorte platform. Patients with juvenile-onset sAIDs attending the pediatric rheumatology unit of Western Switzerland in the University Hospitals of Lausanne and Geneva were enrolled at the study until August 2018. AIDs included diagnosis of Systemic onset juvenile idiopathic arthritis (SoJIA), Familial Mediterranean fever (FMF), Cryopyrin-associated periodic syndromes (CAPS), Mevalonate kinase deficiency (MKD) and Tumor necrosis factor receptor-associated periodic syndrome (TRAPS). A projection for Switzerland was made using the population data extracted from the Swiss Federal Statistical Office in 2016.

**Results:** A total of 123 patients were enrolled, including 67 % females. The median age at last visit was 12.3 years[TA1] . Patients were distributed as follows: 61 patients with SoJIA, 35 with FMF, 15 with CAPS, 8 with MKD and 4 patients with TRAPS. Biologic agents (anti IL-1 or anti IL-6) were used for treatment in 55 % of our patients. The prevalence in our population is 1.4 per 100.000 for sAIDS and 1.5 per 100.000 for SoJIA. The total number of patients with sAIDs is estimated at 139 for monogenic fevers and 131 for SoJIA in Switzerland.

**Conclusion:** AIDs are rare but not negligible conditions in Switzerland. A national study through the JIRcohorte database is ongoing aiming to evaluate the number of pediatric and adult patients with sAIDs in Switzerland, in order to study clinical presentation, treatments and long term complications.

**Disclosure of Interest**

None Declared

#### P1067 Familial Mediterranean fever associated infertility and underlying factors

##### Nuh Atas^1^, Berkan Armagan^2^, Erdal Bodakci^3^, Timucin Kasifoglu^3^, Hasan Satis^1^, Alper Sari^2^, Nazife S. Y. Bilge^3^, Hakan Babaoglu^1^, Gozde K. Yardimci^2^, Reyhan B. Salman^1^, Levent Kilic^2^, Mehmet A. Ozturk^1^, Berna Goker^1^, Seminur Haznedaroglu^1^, Umut Kalyoncu^2^, Abdurrahman Tufan^1^

###### ^1^Department of Internal Medicine, Division of Rheumatology, Gazi University Faculty of Medicine; ^2^Department of Internal Medicine, Division of Rheumatology, Hacettepe University Faculty of Medicine, Ankara; ^3^Department of Internal Medicine, Division of Rheumatology, Eskisehir Osmangazi University Faculty of Medicine, Eskisehir, Turkey

####### **Correspondence:** Abdurrahman Tufan

**Introduction:** Familial Mediterranean Fever (FMF) is characterized by recurrent attacks of fever, serositis and arthritis, but some patients may experience long-term complications of disease such as infertility/subfertility.The published data about FMF associated infertility is still limited.

**Objectives:** The aim of this study is to investigate the frequency and to determine potential factors for FMF associated infertility /subfertility.

**Methods:** All patients recruited from FMF in Central Anatolia (FiCA) cohort, currently comprising 970 adult subjects. All patients fulfilled Tel Hashomer criteria and all were using colchicine for at least 1 year. Demographic data, FMF disease characteristics and genotype data (if available), disease complications, autoinflammatory damage index (ADDI), laboratory parameters and treatment features were recorded. For this study data on 582 patients (mean age 41±10.6, 65.8% female) who had been willing to have children were used.

**Results:** Proportions of FMF manifestations were fever 82.3%, peritonitis 90.5%, pleuritis 48.1%, arthritis 41.1% and skin rash 24.7%. MEFV mutations were available in 454 subjects and 79.5% of subjects were harboring M694V mutation (44.5% homozygous for M694V). Among all, 53 (9.1%) patients were colchicine resistant (crFMF). Infertility was present in 64 patients (14.6 % of females and 4 % of males). Multivariate analysis showed female sex (odds ratio, 8.19; 95% confidence interval [CI95%] 2.69-24.97; p<0.05), FMF disease onset <20 years (odds ratio, 9.9; [CI95% 2.06-47.82]; p<0.05), damage accrual (ADDI score) (odds ratio, 2.02; [CI95% 1.65-2.50]; p<0.05) and colchicine nonresponse (odds ratio, 2.07; 95% confidence interval, [CI95% 1.47-2.93]; P<0.05) are the independent predictors of infertility.

**Conclusion:** Damage accrual (ADDI), FMF disease onset <20 years, colchicine nonresponse and female sex were found to be the independent predictors of infertility. The value of effective therapeutic interventions must be determined to treat infertility in these patients.

**Disclosure of Interest**

None Declared

#### P1068 MEFV-mutant induced pluripotent stem-cells have reduced anti-inflammatory properties

##### Katharina Kessel^1,2^, Christoph Kessel^1^, Nadine Ludwig^3^, Toni Weinhage^1^, Dirk Foell^1^, Helmut Wittkowski^1^

###### ^1^Pediatric Rheumatology and Immunology, University Children’s Hospital; ^2^Department of Nuclear Medicine; ^3^Department of Anesthesiology, Intensive Care and Pain Medicine, University Hospital Münster, Muenster, Germany

####### **Correspondence:** Helmut Wittkowski

**Introduction:** Familial Mediterranean Fever (FMF) is a prototypic autoinflammatory disorder associated with MEFV pyrin-encoding gene mutations, characterized by unprovoked episodes of inflammation. On a molecular level, pyrin inflammasome activation with consecutive increased IL-1beta maturation is an important mechanism of inflammation in FMF.

**Objectives:** Experiments with human MEFV-mutated cells rely on FMF patient material, but patients are often already treated with an anti-inflammatory therapy, i. e. Colchicine. In order to systematically study functional consequences of MEFV pyrin-encoding gene mutations on a therapy-naïve background we aimed to generate human induced pluripotent stem cells (hiPSCs) from M694V homo- and heterozygous FMF-patients as well as healthy controls and differentiated those to functional human monocytes.

**Methods:** HiPSCs were generated from MEFV-mutant patients with clinical FMF and from healthy controls by reprogramming of peripheral blood-derived (PB) CD34+ HSCs. After confirmation of MEFV genotypes, HiPSCs were differentiated to monocytes using an embryoid body (EB)-based differentiation protocol. Terminally differentiated cells were phenotyped regarding extra- and intracellular marker expression as well as cell morphology and were subjected to different functional assays. Cells were stimulated with LPS, ATP and S100A12 and cytokine secretion into culture supernatants was quantified by multiplexed bead array assay. For confirmation of observed phenotypes, *ex vivo* purified primary monocytes from FMF-patients were likewise stimulated and analysed.

**Results:** Terminally differentiated hiPSC-derived monocytes from both FMF patients and HCs were larger in cell area (mm^2^) compared to primary human monocytes. On the level of 25F9, CD16a and CD68 expression hiPSC derived monocytes ranged between that detected on primary cells and *in vitro* differentiated primary monocyte derived macrophages. All cells expressed identical levels of CD14. Similar to our observations on primary monocytes from FMF patients and controls both CD14 and CD16 expression was lowest on p.M694V homozygous hiPSC derived monocytes. On the contrary intracellular S100A9 and A12 expression in both primary as well as hiPSC derived monocytes increased with p.M694V gene dose. Correspondingly, p.M694V homozygous hiPSC-derived monocytes revealed increased *S100A8* expression at base line and already spontaneous protein release in stimulation experiments. In contrast, *Il1rn* expression in p.M694V mutant hiPSC-derived monocytes was low compared to HCs. When induced by IFNa, *Il1rn* expression was reciprocal according to p.M694V gene dose. In similar lines, p.M694V mutant hiPSC-derived monocytes revealed strongly decreased IL-10 expression on both gene and protein level compared to healthy control cells.

**Conclusion:** We successfully generated mature monocytes from hiPSC derived from p.M694V hetero- and homozygous FMF-patients as well as healthy individuals. Apart from S100 proteins p.M694V mutant cells did not reveal a pronounced overexpression of inflammatory cytokines but rather demonstrated an intrinsic lack of anti-inflammatory counter-regulation on the level of IL-1Ra and IL-10 expression.

**Disclosure of Interest**

None Declared

### Monogenic autoinflammatory diseases (genetics)

#### P1069 Three novel cases of late-onset cryopyrin-associated periodic syndromes due to somatic NLRP3 mosaicism

##### Anna Mensa-Vilaró^1^, María Teresa Bosque^2^, Enrique Gómez de la Fuente^3^, Natalia Palmou^4^, Luis Martín-Penagos^5^, Concha Delgado^2^, Susana Plaza^1^, Rocío Lara^1^, María Carmen Antón^1^, Helios Martinez-Banaclocha^6^, Juan J. Martinez-García^6^, Jordi Yagüe^1,7,8^, Miguel Ángel González-Gay^4^, Pablo Pelegrin^6^, Juan I. Arostegui^1,7,8^

###### ^1^Immunology, Hospital Clinic, Barcelona; ^2^Rheumatology, Hospital Universitario Lozano Blesa, Zaragoza; ^3^Dermatology, Hospital Universitario Fundación Alcorcón, Alcorcón; ^4^Rheumatology; ^5^Nephrology, Hospital Universitario Marqués de Valdecilla, Santander; ^6^Instituto Murciano de Investigación Biosanitaria IMIB-Arrixaca, Murcia; ^7^Institut d’investigacions Biomèdiques August Pi i Sunyer; ^8^Universitat de Barcelona, Barcelona, Spain

####### **Correspondence:** Juan I. Arostegui

**Introduction:** Monoallelic *gain-of-function NLRP3* mutations are the genetic cause of the dominantly-inherited cryopyrin-associated periodic syndromes (CAPS), which represent the prototypical early-onset, interleukin-1b-mediated disease (1). Post-zygotic *NLRP3* mutations leading to somatic mosaicism have been shown as the disease-causing mechanism in a moderate, but increasing number of patients (2-3). Recent reports have also shown that somatic *NLRP3* mosaicism may appear later in life, leading to forms of the disease that started during adulthood (4-7).

**Objectives:** To describe the clinical, analytical and genetic features as well as the outcome of administered treatments in three unrelated Spanish patients with late-onset CAPS carrying somatic *NLRP3* mosaicism.

**Methods:** Clinical and analytical data, and outcome of treatments were collected from patients’ medical charts. Genetic studies were performed using both Sanger and amplicon-based deep sequencing.

**Results:** The following table summarizes the clinical data of the three enrolled patients.

All patients displayed increased counts of white blood cells, neutrophils and platelets, as well as increased plasma levels of acute-phase reactants.

Genetic studies revealed in each patient a post-zygotic *NLRP3* variant: p.Gln306His variant in patient 1 (minor allele frequency [MAF]: 5.1%), p.Ala352Thr variant in patient 2 (MAF: 18.7%) and p.Gln636Glu in patient 3 (MAF: 18.4%). All these *NLRP3* variants were classified as pathogenic and *gain-of-function* variants on the basis of their absence among healthy controls and in public databases, the damaging predictions by using different bioinformatics analyses and additional ex vivo evidences of inflammasome hyperactivation.

All three patients are being treated with anti-IL-1 drugs since several years, resulting in a successful clinical control of the disease, normalization of acute phase reactants and hematological parameters, and improvement of renal function in patient 1 who suffered from AA amyloidosis.

**Conclusion:** The results here shown add additional evidences of the relevant role of somatic *NLRP3* mosaicism as the disease-causing mechanism in patients with late onset, but otherwise typical CAPS. Due to the serious consequences that these data could have with regard to their treatments, this genetic mechanism should be seriously considered in the design of genetic analyses to be done in candidate patients.

**References**

^1^Nat Immunol 2017; 18: 832-842

^2^Arthritis Rheum 2011; 63: 3625-3632

^3^Ann Rheum Dis 2015; 74: 603-610

^4^J Allergy Clin Immunol 2015; 135: 561-564

^5^Arthritis Rheumatol 2015; 67: 2428-2436

^6^Arthritis Rheumatol 2016; 68: 3035-3041

^7^Front Immunol 2017; 8: 1410.

**Consent for publication has been obtained from patient**

Yes

**Disclosure of Interest**

None Declared

**Table 1 (abstract P1069).** See text for description

	Patient 1	Patient 2	Patient 3
Sex / Current Age (years)	Female / 63	Female / 66	Male / 67
Age at disease Onset (years)	47	55	56
Fever	Yes	No	Yes
Urticaria-like Rash	Yes	Yes	Yes
Eye Inflammation	Bilateral Uveitis	No	Conjunctivitis
Joint Inflammation	Arthralgias	Arthralgias / Arthritis	Arthralgias / Arthritis
Headache	Yes	Yes	No
Hearing Loss	Yes	No	Yes
AA-type Amyloidosis	Yes	No	No

#### P1070 The NLRP3 p.A441V mutation in cryopyrin-associated periodic syndrome pathogenesis: functional consequences, phenotype-genotype correlations and evidence for a founder effect

##### Eman Assrawi^1^, Fawwaz Awad^2^, Claire Jumeau^1^, Sylvie Odent^3^, Veronique Despert^4^, G. Cam^5^, Aleth Perdriger^6^, Camille Louvrier^1^, Laetitia Cobret^1^, Bruno Copin^1^, Phillipe Duquesnoy^1^, William Piterboth^1^, Claire Le Jeunne^7^, Sophie Georgin-Lavialle^8^, Gilles Grateau^8^, Marie Legendre^1^, Irina Giurgea^1^, Sonia Athina Karabina^1^, Serge Amselem^1^

###### ^1^Sorbonne Université, Inserm UMR_S933; ^2^Sorbonne Université,Inserm UMR_S933, Paris; ^3^Centre Hospitalier Universitaire de Rennes, Service de Génétique; ^4^Centre Hospitalier Universitaire de Rennes, Département de Médecine de L’enfant et de L’adolescent, Rennes; ^5^Centre Hospitalier de Saint-Malo, Service de Néphrologie, Saint-Malo; ^6^Centre Hospitalier Universitaire de Rennes, Service de Rhumatologie, Rennes; ^7^Hôpital Cochin; ^8^Hôpital Tenon , Service de Médecine Interne, Paris, France

####### **Correspondence:** Eman Assrawi

**Introduction:** Cryopyrin‑associated periodic syndromes (CAPS) are a group of rare monogenic autoinflammatory diseases caused by heterozygous missense gain-of-function mutations in *NLRP3* gene, coding for the NLRP3.Upon activation, NLRP3 initiates the formation of a multiprotein complex called inflammasome, which regulates proinflammatory cytokine secretion.

**Objectives:** To determine the molecular and cellular basis of autoinflammatory syndromes in two unrelated families with two clinically overlapping CAPS phenotypes; a multigenerational French family with Muckle-Wells syndrome and in a patient originating from Portugal with familial cold autoinflammatory syndrome.

**Methods:** Sanger sequencing of *NLRP3* exon 3 was performed in all accessible patients. Microsatellites analysis was used to test the intra-familial segregation of the identified variant and to look for a founder effect. Functional analyses included the study of (i) ASC speck formation in HEK293T cells (stably expressing ASC-GFP and pro-caspase1-FLAG) transiently expressing the wild-type or mutated NLRP3 protein, (ii) IL1β secretion from transfected THP1 cells, and (iii) inflammasome-related gene expression and cytokine secretion from monocytes isolated from patients in crisis (probands from the two families), related patients out of crisis, and from controls.

**Results:** The same heterozygous mutation (c.1322C>T, p.A441V) in *NLRP3* exon 3, segregating with the disease within the first family, was identified in the two families which were shown to share the same mutation-associated *NLRP3* haplotype. HEK293T cells transfected with the pNLRP3-A441V construct showed a significantly higher percentage of ASC specks, a common readout of inflammasome activation, as compared to cells transfected with pNLRP3-WT. Transfection of THP1 cells with pNLRP3-A441V led to significantly higher levels of secreted IL1β, a hallmark of inflammasome activation, as compared to cells transfected with pNLRP3-WT. Monocyte inflammasome-related gene expression and cytokine secretion profile was similar in patients out of crisis and in the healthy controls. However, the expression of the inflammasome-related genes in the two probands was different from that of patients without crisis and healthy controls. In addition, this expression pattern was found to be differentially regulated between the two probands, correlating with their phenotypic status.

**Conclusion:** These molecular and cellular findings, which indicate a founder effect in these two families, clearly demonstrate the pathogenicity of the p.A441V missense mutation in CAPS and point to the interest of studying patients’ primary cells to assess disease activity.

**Disclosure of Interest**

None Declared

#### P1071 The analysis of IL-36RA structural dynamics improves pathogenicity predictions for IL36RN variants observed in generalised pustular psoriasis

##### Camilla Davan-Wetton, Niina Karoliina Hassi, Joseph Chifung Ng, Franca Fraternali, Francesca Capon

###### King's College London, London, United Kingdom

####### **Correspondence:** Francesca Capon

**Introduction:** Generalised pustular psoriasis (GPP) is a potentially life-threatening autoinflammatory condition. While GPP is associated with mutations of the interleukin-36 receptor antagonist (IL-36Ra encoded by *IL36RN*), commonly used pathogenicity predictors cannot fully differentiate *IL36RN* disease alleles from polymorphisms.

**Objectives:** The aim of this study was to systematically assess the impact of IL-36Ra mutations on the protein three-dimensional structure, in order to identify the computational approaches that best predict *in-vitro* stability and variant pathogenicity.

**Methods:** The IL-36Ra 3D structure was derived by homology modelling. The effects of mutations were assessed using the mCSM and RAPSODY programs. Experimental validation was undertaken by western blot analysis of mutagenized constructs.

**Results:** The results of the mCSM analysis partially correlated with those obtained by western blot, particularly for well-characterised mutations like p.Leu27Pro and p.Ser113Leu. An improved correlation with the western blot data was achieved using RAPSODY, which incorporates information on protein structural dynamics. Of note, this programme also out-performed sequence- based predictors such as CADD.

**Conclusion:** Computational methods that take into account the dynamic features of the IL-36Ra protein structure show the strongest correlation with experimental data. Extending their implementation to other *IL36RN* variants (e.g. those that affect the interaction between IL-36Ra and its receptor) will improve our understanding of GPP mutations and enable the development of more accurate pathogenicity predictors to help disease diagnosis.

**Disclosure of Interest**

C. Davan-Wetton: None Declared, N. K. Hassi: None Declared, J. C. Ng: None Declared, F. Fraternali: None Declared, F. Capon Grant / Research Support from: Boheringer Ingelheim, Consultant for: AnaptysBio

#### P1072 Spondyloenchondrodysplasia and systemic lupus erythematosus: case report of two sibling

##### Bulent Kara^1^, Ayse Cefle^2^, Ozgur Kasapcopur^3^, Ayfer Sakarya Gunes^1^

###### ^1^Department of Pediatrics Division of Child Neurology; ^2^Department of Internal Medicine Division of Rheumatology, Kocaeli University Faculty of Medicine, Kocaeli; ^3^Department of Pediatric Rheumatology, Istanbul University Cerrahpasa Medical School, Istanbul, Turkey

####### **Correspondence:** Ayse Cefle

**Introduction:** Interferons (IFNs) are signalling proteins that are synthesised and released by immune host cells in response to the presence of the several pathogens. Interferonopathies comprise an expanding group of monogenic diseases characterised by disturbance of the homeostatic control of IFN-mediated immun responses. Although differing in the degree of phenotypic expression and severity, the clinical presentation of the diseases shows a considerable degree of overlap, reflecting their common pathogenetic mechanisms.

**Objectives:** Spondyloenchondrodysplasia with immune dysregulation (SPENCDI) is an immuno-osseous dysplasia combining the typical metaphyseal and vertebral bone lesions of SPENCDI and neurologic involvement.

**Methods:** 19 years old a male patient presented with arthralgia and rash in 2014. On physical examination short stature and erythematous skin rash were noted. Laboratory studies revealedproteinuria (740 mg/d), lymphopenia (1150/mm3), elevated ESR (51 mm/h), CRP (15 mg/l, N<5) and low C3 level. The C4 level was normal. ANA was homogenously positive, while the anti ds-DNA antibody was also positive. Anticardiolipin IgG and IgM and the lupus anticoagulant were negative. HBsAg, HCV and HIV were negative. IgG, IgA and IgM levels were normal. Skin bipsy revealed perivascular dematitis, while a kidney biopsy showed class II lupus nephritis. The patient was given methyl prednisolone, hydroxycholoroquine and azathioprine. On follow up, the proteinuriadecreased. However, he developed attacks of seizuresand ataxia. The neurologic examination, EEG and EMG, were normal. Cranial tomography revealed calcification of lentiform nucleus, corona radiata, frontal lobe white matter and dentate nuclei. In 2015, he presented with abdominal paine, vomiting and nausea. Abdominal CT revealed edema and inflammation of jejenum and dudenum.The steroid dose was increased. After a few months, the symptoms recurred. The patient was thought to have gastrointestinalinvolvement due to systemic lupus erythematosus (SLE) and rituximab was added to the treatment. Thereafter the patient did not have anysymptoms related toacute abdomen.

**Results:** A direct raiographic examination of the spine revealed platyspondyly. The parents were consanguineous. His sister was under follow up due to a diagnosis of juvenil onset SLE.Intracranial calcifications, spastic paraparesis, short stature, systemic lupus erythematosus and platyspondyly suggested a diagnosis of SPENCD. Next generation sequencing of the ACP5 gene showed that the patient and his sisters were homozygous for the c.155A C/p.K52T variant. Both parents were heterozygous forthis variant.

**Conclusion:** SPENCDI is a recessive genetic disease caused by homozygous or compound heterozygous mutation in the ACP5 gene on chromosome 19p13. Immune dysregulation ranges from autoimmunity to immunodeficiency. Neurologic and autoimmune manifestations have been observed in different combinations. Skeletal, nneurologic and immune phenotypes were observed between members of the same family.

**Consent for publication has been obtained from patient**

Yes

**Disclosure of Interest**

None Declared

#### P1073 ADA2 deficiency without ADA2 mutations explained by a structural homozygous variation in 22Q11.1

##### Alice Grossi^1^, Francesca Garbarino^2^, Roberta Caorsi^3^, Roberto Cusano^4^, Marta Rusmini^1^, Federica Penco^3^, Francesca Schena^3^, Rosa Anna Podda^5^, Paolo Uva^4^, Isabella Ceccherini^1^, Marco Gattorno^3^

###### ^1^UOC Genetica Medica, IRCCS Istituto Giannina Gaslini; ^2^Università degli Studi di Genova; ^3^Clinica Pediatrica Reumatologia e UOSD Centro Malattie Autoinfiammatorie-Immunodeficienze, IRCCS Istituto Giannina Gaslini, Genova; ^4^Centre for Advanced Studies Research and Development in Sardinia (CRS4), Science and Technology Park Polaris, Pula; ^5^Clinica Pediatrica,Talassemie e Malattie Rare, Ospedale Brotzu e Università degli studi di Cagliari, Cagliari, Cagliari, Italy

####### **Correspondence:** Alice Grossi

**Introduction:** Adenosine Deaminase 2 deficiency (DADA2) is an autosomal recessive autoinflammatory disease caused by loss of function mutations in the *ADA2* gene, located on chromosome 22q11.1. The clinical spectrum of the disease is heterogeneous, ranging from multisystemic inflammation with vascular and multiorgan involvement to immunodeficiency and immunedysregulation.^1^ A small proportion of patients, despite consistent phenotype and lack of ADA2 enzymatic activity, has an incomplete or negative genotype.

**Objectives:** To define the genetics underlying DADA2 in a 9 years old girl with a complete clinical phenotype and deficient enzymatic activity but without any mutation in the coding region of the *ADA2* gene.

**Methods:** Whole Genome Sequencing (WGS) was performed in the proband by TruSeq Nano DNA Library Prep kit (Illumina) and HiSeq 3000 Instrument (Illumina) with 150bp paired-end reads. Structural variants were identified with Manta.

**Results:** At the age of 3 months, the patient began to suffer from intermittent episodes of fever and livedo reticularis. At 1-year old inflammatory symptoms became persistent and the patient developed hypertension and a severe dilatative myocarditis, responsive to high doses of steroids. Anakinra was not effective, hence steroid therapy could not be suspended for years. Hypogammaglobulinemia was also present, together with recurrent upper airways infections. At the age of 6 years, following the withdrawal of steroids, the patient presented two episodes of ischemic and hemorrhagic stroke. Anti-TNF treatment (etanercept) was then started, allowing a complete control of clinical manifestations, as well as the normalization of Ig levels, without the need of any steroid treatment. The patient has been on anti-TNF as monotherapy for 4 years and is still in complete clinical remission. Whole Genome Sequencing (WGS) has revealed a homozygous tandem duplication of the genomic region encompassing exons 3 and 4 (involving 12895 bases), generated by two breakpoints in intron 2 and intron 4 respectively. This has led to a gene transcript postulated to contain 1967 instead of 1536 nucleotides, leading to a frameshift starting from codon V252 followed by a premature stop codon after 11 aminoacid residues (p.V252Gfs11*). PCR reactions properly designed to amplify the junction fragments from both the genomic DNA and the cDNA confirmed such findings.

**Conclusion:** Chromosome 22q11, where the ADA2 gene lies, is regarded as an unstable region subjected to copy number variations and other structural genomic alterations, whose occurrence is mediated by low copy repeat sequences or segmental duplications, found in association with several different Syndromes.^2^ The structural variation identified in the present patient represents the first demonstration of non-allelic homologous recombination (NAHR) occurred in DADA2, different from causative bi-allelic loss-of-function point mutations, typically found in the coding portion of the ADA2 gene. The identification of ADA2 enzymatic impairment in patients with clinical phenotypes consistent with DADA2 should lead to extensive molecular approaches, especially in the absence of any mutation at standard DNA sequencing.

**References**

1. Moens L., Hershfield M., Arts K., Aksentijevich I., Meyts I. “Human adenosine deaminase 2 deficiency: a multi-faceted inborn error of immunity” Immune Rev 2019; 287(1):62-72

2. Vergés L, Vidal F, Geán E, Alemany-Schmidt A, Oliver-Bonet M, Blanco J. “An exploratory study of predisposing genetic factors for DiGeorge/velocardiofacial syndrome” Sci Rep 2017;7:40031

**Consent for publication has been obtained from patient**

Yes

**Disclosure of Interest**

None Declared

#### P1074 Evaluation of the role of SAA1, SAA2 and SAA4 genes in AA amyloidosis

##### Claire Jumeau^1^, Camille Louvrier^1^, Eman Assrawi^1^, Fawaz Awad^1^, Laetitia Cobret^1^, Sophie Georgin-Lavialle^1,2^, Cécile Cazorla^3^, Philippe Duquesnoy^1^, Jean-Simon Rech^3^, William Piterboth^1^, Florence Dastot-Le Moal^1^, Bruno Copin^1^, David Buob^4^, Gilles Grateau^1,5^, Jean-François Bernaudin^1,6^, Marie Legendre^1^, Sonia-Athina Karabina^1^, Irina Giurgea^1^, Serge Amselem^1^

###### ^1^Sorbonne Universite, INSERM UMR_S933, Hôpital Trousseau; ^2^Hôpital Tenon, Service de Médecine Interne, Paris; ^3^Service de Médecine Interne et Infectiologie, Nouméa; ^4^Hôpital Tenon, Service d'Anatomie et Cytologie Pathologiques; ^5^Hôpital Tenon, Service de Médecine Interne, Paris; ^6^Hôpital Avicenne, Service de Pneumologie, Bobigny, France

####### **Correspondence:** Claire Jumeau

**Introduction:** AA amyloidosis is a rare inflammatory disease, mostly seen as a long term complication of chronic inflammation (ie obesity, gout). It is characterized by amyloid A (AA) deposits inside or outside the cells, affecting organ function. AA proteins originate from the cleavage of serum amyloid A proteins (SAA1, SAA2, SAA4) encoded by the corresponding genes. No molecular etiology for AA amyloidosis has been identified so far; however, a specific genotype (*SAA1.1/SAA1.1*) at the *SAA1* locus has been associated with AA amyloidosis in familial Mediterranean fever (FMF) patients of Armenian origin (OR=7). Another genotype at the *SAA1* locus (*SAA1.3/SAA1.3*) was associated with AA amyloidosis in Japanese patients with rheumatoid arthritis.

**Objectives:** gout.

This work aimed at identifying candidate genes in primary AA amyloidosis or secondary to obesity or gout.

**Methods:** *SAA1*, *SAA2* and *SAA4* genes were screened by Sanger sequencing in all patients and the genotype at the *SAA1* locus was determined in 35 independent patients: 18 presented with primary AA amyloidosis, 10 with AA amyloidosis in the context of obesity and 7 with AA amyloidosis in the context of gout.

**Results:** We initially searched for rare variants in the 3 *SAA* genes in the different groups of patients. Only one rare heterozygous variant in *SAA2* resulting in a missense variation p.(Ala79Val) was identified in one patient with primary AA amyloidosis.

The characterization of the genotypes at the *SAA1* locus showed a higher frequency (89 and 80%) of the *SAA1.1/SAA1.1* genotype in the groups of patients with primary amyloidosis and amyloidosis secondary in the context of obesity, significantly different from its theoretical frequency in healthy controls based on gnomAD database (22%) (Fisher test: p=1,3 .10^-4^ and p=0.023 respectively). However in the group of patients with AA amyloidosis in the context of gout, 71% presented the *SAA1.3/SAA1.3* genotype.

**Conclusion:** The variation in *SAA2* affects evolutionarily conserved amino-acid. This result paves the way for functional studies on AA fibril formation in order to assess its pathogenicity. The predominance of the *SAA1.1/SAA1.1* genotype among patients with primary AA amyloidosis and with amyloidosis in the context of obesity suggests that this genotype could be a risk factor for AA amyloidosis.

**Disclosure of Interest**

None Declared

#### P1075 An integrated data sharing and analysis application for systemic autoinflammatory diseases

##### Barend P. Kant^1^, K J. van der Velde^2^, Mariska K. Slofstra^2^, Joost Frenkel^3^, Andreas Weinhäusel^4^, Isabelle Touitou^5^, Dirk Föll^6^, JuanI. Aróstegui^7^, Seza Ozen^8^, Marco Gattorno^9^, Mariëlle E. van Gijn^1^, Morris A. Swertz^2^ and the INSAID Consortium

###### ^1^Department of Genetics, University Medical Center Utrecht, Utrecht; ^2^Genomics Coordination Center and Department of Genetics, University of Groningen and University Medical Center Groningen, Groningen; ^3^Department of Pediatrics, University Medical Center Utrecht, Utrecht, Netherlands; ^4^Department of Health and Environment, Austrian Institute of Technology, Vienna, Austria; ^5^Department of Medical Genetics, Rare Diseases and Personalised Medicine, Inserm, Montpellier, France; ^6^Department of Pediatric Rheumatology and Immunology, University of Münster, Münster, Germany; ^7^Department of Immunology, Hospital Clínic of Barcelona, Barcelona, Spain; ^8^Department of Pediatrics, Hacettepe University, Ankara, Turkey; ^9^Department of Pediatric Rheumatology, IRCCS G. Gaslini Institute, Genoa, Italy

####### **Correspondence:** Barend P. Kant

**Introduction:** Systemic autoinflammatory diseases (SAID) are a group of monogenic and multifactorial conditions caused by deregulation of mechanisms controlling the innate immune response. The diagnosis of patients with SAID is difficult, which can result in delayed treatment and irreversible organ damage. Patients benefit from fast molecular diagnosis, but for most patients with a clinical picture strongly resembling autoinflammatory disease, molecular analysis will not provide diagnostic confirmation. These patients are classified as undefined SAID (uSAID).

**Objectives:** The INSAID consortium aims to improve the performance of genetic diagnosis by speeding up identification of new conditions, biomarkers and causal DNA variants (in new genes). For this purpose, a central and easy to use bioinformatics platform has been set up, facilitating sharing of clinical, genetics, epigenetics, immunomics and proteomics data among the consortium partners and community-driven data analysis and interpretation.

**Methods:** Clinical data were retrieved from the Eurofever registry. Other data were produced by the consortium partners. A MOLGENIS database was set up for data sharing and analysis. MOLGENIS is a user friendly open-source web-application created to collect, manage, analyze, visualize and share large and complex biomedical datasets.

**Results:** The INSAID database is being used by the INSAID consortium. All data types are being captured using patient identifier codes as keys for data integration and analysis. New patients can be included and future data and data types can be uploaded and integrated. Consortium progress can be monitored and discussed using the database. The database’s interface is easy to operate and cluster analyses and statistical tests can be written and shared within the database. This enables all consortium partners with or without data analysis skills to query, sort, filter, analyze and visualize the available multi-omics data.

**Conclusion:** The INSAID database is set up to facilitate data sharing and to perform multi-omics analysis. This will enable diagnosing patients with currently undefined SAID, discovery of pathways involved and further elucidating disease pathogenesis, ultimately contributing to development of new or improved treatment.

**Disclosure of Interest**

None Declared

#### P1076 Comparative analysis ofreported 54 HA20 cases and 520 Japanese Behcet’s disease patients

##### Yohei Kirino, Naomi Tsuchida, Yutaro Soejima, Hideaki Nakajima

###### Department of Stem Cell and Immune Regulation, Yokohama City University Graduate School of Medicine, Yokohama, Japan

####### **Correspondence:** Yohei Kirino

**Introduction:** Phenotype of haploinsufficiency A20 (HA 20) and Behcet’s disease (BD) are reported to be similar. However, the distinction between HA 20 and BD is unknown.

**Objectives:** To clarify specific features of HA20 compared to BD.

**Methods:** The clinical features of 54 cases of HA 20 cases were examined from the literature including our 4 cases and compared with 520 Japanese BD patients followed at our facility. All BD patients met the 1987 revised diagnostic criteria of the Japanese Bebcet's Disease Eesearch Committee. The research protocol was approved by the facility review committee of Yokohama City University School of Medicine. Statistical analysis was performed using SPSS version 22 (IBM Japan, Tokyo, Japan). Category variables were analyzed using Chi-square test or Cochran-Armitage test. Continuous variables were examined using Student's t test. A p value less than 0.05 was considered to be statistically significant.

**Results:** significantly high recurrent fever (74.0%) and gastrointestinal involvement, and less eye involvement than BD. Among gastrointestinal involvement and HA20 patients, 22 (81.8 percent) 18 showed gastrointestinal ulcers including 2 cases with typical deep iliocecal ulcer seen in BD. The positive rate of HLA-B51 was lower (27.3 %) than that of BD (47.8 %), but since the majority of HA 20 has been reported by European populations whose HLA-B51 positive rate is lower than Japanese, it is uncertain whether HLA-B51 affects on HA20 phenotype or not.

**Conclusion:** Comparison of HA 20 and BD cohort revealed several important features of HA 20: early onset, familial onset, recurrent fever, gastrointestinal involvement, and less eye involvement. Patients with familial recurrent fever with early onset with BD symptoms, especially gastrointestinal disorders, should consider TNFAIP3 gene screening.

**Disclosure of Interest**

None Declared

**Table 1 (abstract P1076).** See text for description

Characteristics	HA20		BD		p	Odds ratio	95%CI	
	(n=54)	(%)	(n=520)	(%)				
Age at onset (years) (mean ± SD)	6.1 ± 6.5		36.4 ± 12.3		< 0.001	533.71	171.84	1657.65
Familial history	15 /25	60	19 /332	5.7	< 0.001	24.71	9.8	62.29
Recurrent fever	37 /50	74	39 /364	10.7	< 0.001	23.72	11.62	48.43
Oral ulcer	46 /52	88.5	518 /520	99.6	< 0.001	0.03	0.01	0.15
Genital ulcer	34 /52	65.4	372 /520	71.5	0.35	0.75	0.41	1.37
Eye involvement	5 /51	9.8	330 /520	63.5	< 0.001	0.06	0.02	0.16
Skin involvement	27 /51	52.9	461 /520	88.7	< 0.001	0.14	0.08	0.27
Gastrointestinal involvement	22 /52	40.7	78 /520	15	< 0.001	4.16	2.28	7.58

#### P1077 Novel missense mutation of TNFAIP3 gene in a family with A20 haploinsufficiency

##### Charlotte Borocco^1,2^, Guillaume Sarrabay^3,4^, Guilaine Boursier^3,4^, Solene Artru^5^, Helene Palluy^1^, Isabelle Touitou^3,4^, Isabelle Kone-Paut^1,2^

###### ^1^Pediatric Rheumatology, Bicetre University Hospital; ^2^CeReMAIA, Le Kremlin-Bicêtre; ^3^Laboratory of Rare and Autoinflammatory Genetic Diseases, Montpellier University Hospital; ^4^CeReMAIA, Montpellier; ^5^Department of Pediatric Gastroenterology, Hepatology and Nutrition, Necker Enfants Malades University Hospital, Paris, France

####### **Correspondence:** Isabelle Kone-Paut

**Introduction:** A20 haploinsufficiency syndrome is a newly described autoinflammatory disease which involves *TNFAIP3* gene. The heterozygous mutations of this gene lead to an impaired negative regulation of the NF-κB pathway. The phenotype is broad and still in process of characterization but encompasses mainly a Behçet phenotype with an unusual gastro intestinal involvement. Patients can encompass an autoinflammatory and an autoimmunity phenotype.

**Objectives:** We report a French family with 4 affected members and a new mutation.

**Methods:** Four patients over 3 generations were assessed. All the clinical and laboratory data were collected retrospectively. The genetical data were performed by new generation sequencing and Sanger sequencing.

**Results:** We identified in all the 4 patients a novel heterozygous missense mutation c.[464C>T] p. Thr155Met in the ovarian tumor domain of the *TNFAIP3* gene. This is the second missense mutation described. This mutation seems to have a variable penetration with a carrier patient presenting only psoriasis. The symptoms presented in this Caucasian family were: oral ulcers (n=3), recurrent fever (n=2), arthralgia (n=2), rectorrhagia (n=2), genital ulcers (n=1), abdominal pain (n=2), Gougerot-Sjögren syndrome (n=1), primary biliary cholangitis (n=1), purpuric rash (n=1), Asperger syndrome (n=1) and psoriasis (n=1). For the laboratory abnormalities, one patient presented a transient rheumatoid factor, one had permanent anti-nuclear antibodies associated to anti-SSA and anti-centromere antibodies and one had double negative T lymphocytes increased. One patient was treated by colchicine with a partial effect on oral ulcers.

**Conclusion:** A20 haploinsufficiency syndrome is a new cause of autoinflammatory and autoimmunity disease. As an autosomal dominant heritance disease, the penetration can be variable. We confirm the possibility of a second missense mutation in *TNFAIP3*. Futher funtional studies will be required to confirm the mecanism of pathogenecity in this family.

**Disclosure of Interest**

None Declared

#### P1078 Development and clinical application of a targeted next generation sequencing gene panel for monogenic autoinflammatory diseases of the CNS

##### Dara McCreary^1^, Ebun O. Omoyinmi^1^, Ying Hong^1^, Ciara Mulhern^1^, Charalampia Papadopoulou^2^, Muthana Al Obaidi^2^, Kshitij Mankad^3^, Kimberly Gilmour^4^, Cheryl Hemingway^5^, Paul Brogan^6^, Despina Eleftheriou^1,7^

###### ^1^Infection, Immunity and Inflammation, University College London; ^2^Paediatric Rheumatology Department, GOSH; ^3^Paediatric Neuroradiology, UCL Great Ormond Street Institute of Child Health; ^4^Paediatric Immunology Department; ^5^Paediatric Neurology Department, GOSH; ^6^Infection, Immunity and Inflammation, UCL GOSH; ^7^ARUK centre for adolescent rheumatology, University College London, London, United Kingdom

####### **Correspondence:** Dara McCreary

**Introduction:** Monogenic autoinflammatory diseases (AID) are severe lifelong systemic inflammatory disorders with dysregulated innate immunity, causing significant morbidity, mortality, and economic burden. Often these diseases present with isolated central nervous system (CNS) involvement and mimic other non-inflammatory CNS disorders. This significantly hampers the clinical management of these patients, who are subjected to expensive and often invasive diagnostic patient pathways. Securing a molecular diagnosis is of major importance in these cases for treatment, prognosis, and genetic counselling. Routine genetic screening is time-consuming, costly, and lacks sensitivity since only common disease harbouring exons of a minority of the known genes causing autoinflammation of the CNS are currently tested using Sanger sequencing. Next-generation sequencing (NGS) offers the ability to rapidly and cost-effectively screen all exons of a gene panel containing hundreds of genes. This approach has not yet been routinely introduced in the UK for these conditions.

**Objectives:** To develop and evaluate the performance of a NGS gene panel for AID with predominant CNS involvement.

**Methods:** The Agilent SureDesign tool was used to design an NGS panel targeting 256 genes, grouped into the following broad clinical phenotypes: AID; monogenic vasculitis/vasculopathy; interferonopathies; haemophagocytic lymphohistiocytosis (HLH); immunodeficiencies; metabolic diseases and other inherited white matter diseases. The targeted region includes coding exons, conserved non-coding exons, upstream promoter regions, and splice sites. Captured and indexed libraries (QXT Target Enrichment System) were sequenced as a multiplex of 16 samples on an Illumina MiSeq sequencer in paired-end mode. Positive controls for panel validation comprised 16 DNA samples from patients with confirmed mutations. We then applied the panel to test 60 prospective samples with suspected but unconfirmed monogenic autoinflammation of the CNS. Read alignment, variant calling, and annotation were performed using Agilent SureCall v3.0 software.

**Results:** In the validation stage, our targeted panel detected all known mutations in the 16 positive control samples. The panel was effective at detecting different types of variants, including rare and common single nucleotide variants (SNVs), insertion/deletions, splice-junction variants, upstream promoter region variants, and somatic mosaicism. Prospective testing of the panel in 60 patients revealed pathogenic variants (class 4 or 5) in 23 of 60 patients giving a detection rate of 38%.A definitive molecular diagnosis was established in 11/60 patients (18%). These included patients with isolated CNS involvement in the context of primary HLH (5/11), monogenic interferonopathies (2/11), AID (2/11), metabolic conditions (1/11) and immunodeficiencies (1/11).

**Conclusion:** This study demonstrates the clinical utility of a comprehensive NGS-based targeted gene panel which was both highly sensitive and specific in detecting different sequence variants of clinical significance. We are currently integrating this panel into a routine diagnostic laboratory testing strategy for monogenic autoinflammatory diseases of the CNS.

**Disclosure of Interest**

None Declared

#### P1079 Next generation sequencing analysis of an NLRP3 mutation negative CAPS cohort

##### Sonia Melo Gomes^1^, Ebun Omoyinmi^1^, Juan Arostegui^2^, Ying Hong^1^, Nigel Klein^1^, Paul Brogan^1^

###### ^1^Infection, Inflammation and Rheumatology, UCL GOS Institute of Child Health, London, United Kingdom; ^2^Immunology -CBD, Hospital Clínic, Barcelona, Spain

####### **Correspondence:** Sonia Melo Gomes

**Introduction:**

Cryopyrin Associated Periodic Syndromes (CAPS) are caused by autosomal dominant gain of function mutations in the *NLRP3* gene. However, a subset of these patients with typical clinical features and good response to anti-IL-1 treatment, have no mutation in *NLRP3* detected by conventional Sanger DNA sequencing. Somatic mosaicism may account for between 19 to 69% of these, from previous reports. Genetic heterogeneity has also been implicated in patients with CAPS-like phenotypes, with the identification of pathogenic mutations in the *NLRP12*, *PLCG2* and *NLRC4* genes (Familial Cold Autoinflammatory Syndrome subsets 2, 3 and 4, respectively).

**Objectives:**

To explore the genetic cause(s) in a paediatric cohort of mutation negative CAPS patients using Next Generation Sequencing (NGS) techniques, by assessing:

1. the rate of somatic *NLRP3* mosaicism, and
2. possible contribution of other/novel genes

**Methods:**

Patients from a single paediatric centre who met the CAPS diagnostic criteria and had no *NLRP3* mutations identified by partial (exons 3, 4 and 6) or total Sanger sequencing, were identified.

The NGS approach used in this study was Amplicon-based Deep Sequencing (ADS) for identification of *NLRP3* mosaicism; and Whole Exome Sequencing (WES) for exploration genetic heterogeneity (i.e. other causative mutations). ADS was performed on an Ion Torrent platform using the Ion Torrent PGM HiQ sequencing kit. Resulting sequences were analysed using the Amplicon Variant Analyzer software. WES was performed using the Nextera Exome Capture sequencing kit and Hiseq sequencing platforms. Exome data was analysed in the Galaxy web-based suit. The wANNOVER web-based software was used for variant annotation.

**Results:** A total of 8 patients who met the CAPS clinical diagnostic criteria and had no mutation identified by conventional Sanger sequencing were included in this study. ASD identified the presence of a somatic mutation in 3 of these patients (37.5%), with an allelic frequency varying from 3.1 to 14.5%.

The remaining patients were studied by WES. In Patient 4 a novel *NLRP3* mutation was identified in exon 5, which had been previously missed due to a restricted Sanger approach.Patient 5 had a novel *NOD2* mutation, thus redefining the diagnosis as Blau syndrome. WES findings in the other patients are summarized in the Table.

**Conclusion:**

In this study, somatic *NLRP3* mutations were identified in 3/8 (37.5%) of our cohort, and 3/8 had variants in 3 other genes, potentially of relevance to the phenotype. In particular, the identification of a *NOD2* mutation and subsequent reclassification of that patient as Blau syndrome, illustrates the limitations of current CAPS diagnostic criteria as well as potential clinicaloverlap between different autoinflammatory syndromes, reiterating the use of a wider NGS approach.At present it is unclear how the variants found in patients 6 and 7 could modulate the phenotype, therefore in 3 patients a clear genetic cause is yet to be found.

**In conclusion**, this study suggests that NGS approaches are superior to conventional sequencing for the molecular diagnosis of CAPS and CAPS like phenotypes since they can detect somatic mosaicism or alternative molecular diagnoses mimicking CAPS.

**Consent for publication has been obtained from patient**

Yes

**Disclosure of Interest**

None Declared

**Table 1 (abstract P1079).** See text for description

Patient (gender, age at testing)	CAPS phenotype	NGS	Gene	aa change	Type
1- M, 8y	CINCA	ADS	*NLRP3*	p.F566L	Mosaic 14.5%
2-M, 8y	MWS	ADS	*NLRP3*	p.E567K	Mosaic3.1%
3-M, 1y	MWS	ADS	*NLRP3*	p.G564D	Mosaic 12.5%
4 -M, 2y	CINCA	WES	*NLRP3*	p.G779V	Heterozygous
5-F, 16y	CINCA-like	WES	*NOD2*	p.D512Y	Heterozygous
6- M, 14y	MWS-like	WES	*IL36RN*	p.S113L	Heterozygous(AR)
7- M, 8y	MWS	WES	*AP1S3*	p.F4C	Heterozygous
8- F, 2y	MWS	WES	-	-	-

#### P1080 A recessive mutation in NLRC4 (A160T) causes autoinflammation and immunedysregulation, and predisposes to ulcerative colitis in heterozygous carriers

##### Leonardo O. Mendonca^1,2^, Annemarie Steiner^3^, Alessandra Pontillo^4^, Isabella Ceccherini^5^, Francesco Caroli^5^, Alice Grossi^5^, Jonas Moecking^6^, Fiona Moghaddas^6,7,8^, Marco Gattorno^9^, Seth Masters^3^

###### ^1^International Center for Autoinflammatory Diseases and Primary Immunodeficienceis, Istituto Giannina Gaslini; ^2^Immunogenetics Laboratory, Biomedical Science Institute, University of São Paulo, Sao Paulo, Brazil; ^3^Inflammation Division, The Walter and Eliza Hall Institute of Medical Research, Parkville, Australia; ^4^Immunogenetics Laboratory, Biomedical Science Institute, University of São Paulo, São Paulo, Brazil; ^5^UOC Medical Genetics, Istituto Giannina Gaslini, Genoa, Italy; ^6^Inflammation Division, The Walter and Eliza Hall Institute for Medical Research; ^7^Department of Medical Biology, The University of Melbourne; ^8^Department of Clinical Immunology and Allergy, The Royal Melbourne Hospital, Parkville, Australia; ^9^International Center for Autoinflammatory Diseases and Primary Immunodeficienceis, Istituto Giannina Gaslini, São Paulo, Brazil

####### **Correspondence:** Leonardo O. Mendonca

**Introduction:** NLRC4 associated autoinflammatory disease (NLRC4-AID) was recently described due to autosomal dominant mutations. The clinical spectrum ranges from macrophage activation syndrome with severe enterocolitis to CAPS-like disease. As opposed to mutations that trigger other inflammasomes, which result in IL-1b driven disease, IL-18 seems to be the cytokine responsible for the clinical manifestations of NLRC4-AID.

**Objectives:** Report the phenotype of a patient carrying a novel, homozygous mutation in NLRC4 (A160T). Describe in vitro evaluation that supports the pathogenicity of NLRC4 A160T in terms of inflammasome activation and IL-18 production. Provide evidence for a genetic association between heterozygous A160T and ulcerative colitis.

**Methods:** Clinical data were acquired from patients records. DNA was extracted and sequenced using standard procedures. Monocytes were isolated by adherence from peripheral blood using Ficoll-Paque gradient for IL-1, IL-6 and IL-18 measured by ELISA. Flow cytometry for quantification of ASC speck formation by TOFIE was examined with overexpressed WT or A160T NLRC4 in HEK 293T cells. THP-1 monocytes were reconstituted with WT and A160T NLRC4, then priming was performed with Pam3CSK4 at a final concentration of 100 ng/ml for 3 hours to evaluate cell death and Il-18 secretion. Genome wide assocaition of NLRC4(A160T) with ulcerative colitis was interrogated using data from the IBD exomes portal.

**Results:Case Report**- The adult patient complained since 6 months of life of recurrent episodes of systemic inflammation characterized by recurrent low grade fever, chills, oral ulcer, uveitis, arthralgia and abdominal pain followed by diarrhea with mucus and variable skin rash Low levels of IgG and CD19 were a persistent finding. High doses of corticosteroids were effective in controlling disease and anti-IL1 partially controlled symptoms. Serum cytokine levels during the use of anti-IL1 evidenced increased levels of IL-18 (285 pg/mL) compared to healthy control (median - 100 pg/mL) and undetectable level of IL-1. **Genetic analysis-** Target gene panel found an homozygous mutation in NLRC4 gene (NM_021209), exon 4, c.478G>A (p.Ala160Thr). Familial genetic segregation was done using standard Sanger Sequencing and the father, mother and a healthy brother are heterozygous for the mutation. The estimated allele frequency is of 0,09% and the frequency for the homozygous status is 0%.**In vitro studies-** Increased ASC speck formation, and IL-1b/IL-18 secretion was observed when NLRC4 (A160T) was overexpressed in human cell lines. Dominant mutations in NLRC4 (eg S171F) result in stronger inflammasome activation than A160T, which is recessively inherited in these two cases. Patient monocytes evidenced high levels of IL-1b and IL-18 secretion in response to ATP, LPS and Flagellin.**GWAS association**- The NLRC4(A160T) allele is significantly enriched in ulcerative colitis compared to healthy controls, OR 2.546 (95% 1.778-3.644), p=0.01305.

**Conclusion:** Homozygous NLRC4(A160T) causes a monogenic disease characterised by autoinflammation and immunedysregulation. This allele is pathogenic in vitro, however with reduced strength compared to dominant mutations in NLRC4. Carriers with NLRC4(A160T) are at increased risk of developing ulcerative colitis.

**Disclosure of Interest**

None Declared

#### P1081 The Australian Autoinflammatory Diseases Registry (AADRY): a streamlined approach to the genetic and immunological evaluation of monogenic autoinflammatory diseases

##### Fiona Moghaddas^1,2,3^, Jenni Harris^2^, Dunja Vekic^4^, Harapas Cassandra^2^, Dale J. Calleja^2^, Navid Adib^5^, Jonathan Akikusa^6^, Roger Allen^6^, Dan Andrews^7^, Vanessa Bryant^3,8^, Geoffrey Cains^4^, Jeffrey Chaitow^9^, Britt Christensen^10^, David Coman^11^, Matthew Cook^12^, Angela Cox^6,13^, Jo A. Douglass^1^, Peter Gowdie^6,13^, Laine Hosking^14^, Matthew F. Hunter^15^, Paul Kubler^16^, Senq J. Lee^17^, Jane Munro^6^, Samar Ojaimi^18^, William Renton^6^, Davinder Singh-Grewal^9,19^, Charlotte Slade^1,3,8^, Joanne Smart^14^, Georgina Tiller^6^, Ben Whitehead^20^, Jane Woods^4^, Philip Wu^21^, Sam Mehr^14^, Ian P. Wicks^2,3,22^, Seth L. Masters^2,3^ and AADRY

###### ^1^Clinical Immunology and Allergy, The Royal Melbourne Hospital; ^2^Inflammation Division, WEHI; ^3^Department of Medical Biology, The University of Melbourne; ^4^Dermatology Department, Liverpool Hospital; ^5^Queensland Rheumatology Services, Arthur House, Red Hill; ^6^Rheumatology Department, The Royal Children's Hospital; ^7^The John Curtin School of Medical Research, Australian National University, Canberra; ^8^Immunology Division, WEHI; ^9^Rheumatology Department, The Children’s Hospital at Westmead; ^10^Gastroenterology Department, The Royal Melbourne Hospital; ^11^Metabolic Medicine, Queensland Children’s Hospital, Brisbane; ^12^Centre for Personalised Immunology; ^13^Paediatric Rheumatology Department, Monash Children’s Hospital; ^14^Paediatric Immunology Department, The Royal Children’s Hospital; ^15^Monash Genetics, Monash Health; ^16^Department of Rheumatology, Royal Brisbane and Women's Hospital; ^17^Department of Rheumatology, Perth Children's Hospital; ^18^Department of Infection and Immunity, Monash Children’s Hospital; ^19^Rheumatology Department, The Sydney Children’s Hospital; ^20^Rheumatology Department, Queensland Children’s Hospital, Brisbane; ^21^Australian Phenomics Facility, Australian National University, Canberra; ^22^Rheumatology Department, The Royal Melbourne Hospital, Australia

####### **Correspondence:** Fiona Moghaddas

**Introduction:** Since the original publication in 1971 outlining two cases of FMF complicated by pulmonary amyloidosis, there have been only two studies looking at the status of monogenic autoinflammatory disorders (AIDs) in Australia. Clinicians caring for patients with an AID without a pathogenic mutation in known disease-causing genes do not have a streamlined approach to further genetic evaluation.

**Objectives:** The Australian Autoinflammatory Diseases Registry (AADRY) aims to provide clinicians with access to a research team to further evaluate patients without an established genetic diagnosis as well as to address limitations in the current knowledge of AIDs in Australia.

**Methods:** Patients with suspected AIDs were recruited as trios. Whole exome sequencing (WES) was performed on whole blood or saliva using the Hiseq 4000 platform and Illumina Sureselect XT V5 library. A variant list was generated using a pipeline, variant caller and filtering strategy previously described. Initial filtering of the variants proceeded by excluding synonymous and common variants, and intronic variants more than 50 base pairs from an intron-exon junction. Sequence data quality was assessed by viewing the BAM file on the Integrated genomics viewer. Variant classification was based on ACMG consensus guidelines.

**Results:** From September 2015 to June 2018, 34 index cases underwent WES. Initial curation analysis looked at autoinflammatory genes as per IUIS and ISSAID. A total of 77 variants in 34 patients were filtered with no pathogenic or likely pathogenic variants detected. The majority (82%) of variants were VUS. Nineteen trios underwent WES. Seventy-one de novo variants (mean 4 per index case), 77 AR variants (mean 4) and 253 potential CH variants (mean 13) were curated. Of the 5 families recruited with more than one symptomatic member, a likely pathogenic variant segregating with disease was found in one family. The pedigree demonstrated multiple generations symptomatic of various degrees of pustular acne and hidradenitis suppurtiva. A novel truncating variant in NCSTN (c.1485C>T) encoding nicastrin p.Glu454X segregated with disease.

**Conclusion:** AADRY has curated the WES of 34 index cases, including 19 trios. A likely pathogenic variant was discovered in one family with a highly penetrant dominantly inherited dermatological phenotype. No clear genetic diagnosis has been made in the remaining index cases, however a number of candidate novel variants are being evaluated. Further work is now underway to gather data on patients with genetically defined AIDs so that the retrospective cohort of patients with an established diagnosis is captured.

**Disclosure of Interest**

None Declared

#### P1082 Genotypic diversity observed within a large cohort of Armenian patients with late-onset familial Mediterranean fever

##### Gernot Kriegshäuser^1,2^, Hasmik Hayrapetyan^3,4^, Stepan Atoyan^3,4^, Stefan Nemeth^5^, Christian Oberkanins^5^, Tamara Sarkisian^3,4^

###### ^1^Institute of Clinical Chemistry and Laboratory Medicine, General Hospital, Steyr; ^2^Clinical Institute of Medical and Laboratory Diagnostics, Medical University, Graz, Austria; ^3^Center of Medical Genetics and Primary Health Care; ^4^Department of Medical Genetics, Yerevan State Medical University, Yerevan, Armenia; ^5^ViennaLab Diagnostics, Vienna, Austria

####### **Correspondence:** Christian Oberkanins

**Introduction:** Familial Mediterranean fever (FMF) as an autoinflammatory disease, results from mutations in the *MEFV* gene mainly with an autosomal recessive mode of inheritance. The age of onset of FMF varies, with about 60% and 90% of patients experiencing their first attack before the age of 10 and 20 years, respectively. Hence, FMF with the first attack occurring at the age of ≥ 40 years (i.e. late-onset FMF) is rare and only a few small studies have addressed this disease subset.

**Objectives:** This work aimed at investigating the molecular genetic characteristics of Armenian patients diagnosed with late-onset FMF.

**Methods:** Genomic DNA isolated from 354 Armenian late-onset FMF patients were analysed for the 12 most common *MEFV* mutations plus SAA1 isoforms 1.1, 1.3 and 1.5 using multiplex PCR and reverse-hybridisation. Mutational spectra and resulting genotypes were then matched against the clinico-demographic profiles collected for these patients.

**Results:** Of all 354 patients, 194 (54.80%) were female and 160 (45.20%) were male. The following genotypes were significantly associated with the late-onset variant: M680I/E148Q (P=0.004), M694V/E148Q (P<0.001) and V726A/V726A (P=0.001). Of note, 12/354 (3.39%) patients were found to be homozygous for the M694V mutation.

**Conclusion:** Our data suggest that late-onset FMF is more prevalent in women and is of greater genetic diversity than previously reported. Further studies including late-onset FMF patients homozygous for *MEFV* mutation M694V are ongoing and may lead to the identification of novel disease-modifying mechanisms.

**Disclosure of Interest**

None Declared

#### P1083 Genetic polymorphism of familial Mediterranean fever in Georgia - a snapshot

##### Karaman Pagava, Helen Phagava, Davit Tatoshvili

###### Tbilisi State Medical University, Tbilisi, Georgia

####### **Correspondence:** Karaman Pagava

**Introduction:** There are very few publications about FMF and MEFV mutations in Georgia.

**Objectives:** Determination of the MEFV mutations spectrum and their frequency in Georgia.

**Methods:** Review of the appropriate literature. 14 sources on the situation in Georgia published from 1981 until now, were analyzed. 8 of them included genetic data. In summary MEFV mutation was investigated in 202 clinically healthy neonates in Tbilisi and 180 patients (children and adolescents) from different regions of Georgia.

**Results:** The carrier rate of MEFV mutations in neonates was remarkable: 31/202; 15.3%, most frequently- E148Q (15/31), M680I (5/31), M694V (4/31). This could be explained by high proportion of people of Armenian ethnicity and high frequency of interethnic marriages. In patients the main mutations were: M694V (65.6%), V726A (23.3%), M6801 (20.6%). Overwhelming majority of patients had Armenian roots. This factor explained the similarity of MEFV mutations in both countries. Some difference between data in clinically healthy newborns and patients was found. Maybe the cause is the extent to which a particular mutation is expressed. Additional research in this direction is needed. Recently Georgian Society on Familial Mediterranean Fever, Autoinflammatory and Autoimmune Diseases has been established. Hopefully it will help to create more comprehensive registry of patients with these diseases and obtain new data about their genetic and clinical polymorphism.

**Conclusion:** The up-to-date information regarding MEFV genes in Georgia is presented. The awareness of FMF and the availability of appropriate testing should be promoted further in Georgia.

**Disclosure of Interest**

None Declared

#### P1084 Exploratory study of MYD88 L265P and clonal haematopoiesis in 30 patients with Schnitzler syndrome, an acquired systemic autoinflammatory disorder

##### Shelly Pathak^1^, Dorota Rowczenio^2^, Roger Owen^3^, Gina D. Doody^4^, Darren Newton^4^, Claire Taylor^4^, Catherine Cargo^3^, Philip Hawkins^2^, Karoline Krause^5^, Helen Lachmann^2^, Sinisa Savic^1^

###### ^1^Leeds Institute of Rheumatic and Musculoskeletal Medicine, University of Leeds, Leeds; ^2^National Amyloidosis Centre, University College London, London; ^3^Department of Haematology, St James’s University Hospital; ^4^Leeds Institute of Cancer and Pathology, University of Leeds, Leeds, United Kingdom; ^5^Department of Dermatology and Allergy, Universitätsmedizin Berlin, Berlin, Germany

####### **Correspondence:** Shelly Pathak

**Introduction:** Schnitzler syndrome (SchS) is rare, acquired, IL-1B mediated, autoinflammatory disorder, for which the pathogenesis is unknown.

**Objectives:**

1. Explore the concept of ‘inflamm-ageing’ by looking for evidence of clonal haematopoesis, as this phenomenon has been linked with pro-inflammatory changes of the immune system.
2. Investigate the prevalence of the MYD88 L265P mutation in SchS patients, given that 30% of SchS patients go onto develop WM.
3. To perform deep sequencing of *NLRP3* region in an additional 11 SchS patients

**Methods:** Informed consent was provided by all subjects, in accordance with the Declaration of Helsinki.

1. Exon-specific regions of 28 genes associated with MDS were amplified using the Fluidigm custom-made 48x48 access array for Illumina library preparation. The sequencing was performed on an Illumina MiSeq using an in-house analysis pipeline. Low level variants were confirmed by repeat sequencing. For the female patients, inactivation of the X chromosome (XCI) was assessed by determination of the androgen receptor (AR) locus methylation status using the HUMARA assay. The XCI ratios are reported as a percentage:<80% is considered random X-inactivation, and percentages >80% are deemed as skewed.
2. The Allele Specific Oligonucleotide-PCR (ASO-PCR) technique was used to identify patients harbouring the mutant ‘C’ allele at chromosomal location 3:38182641, leading to the downstream MYD88 Leu265Pro mutation.This PCR based assay discriminates between the WT and mutated alleles by using 3 sets of primers, followed by direct amplification producing 2 different sized products (400bp – WT; 250bp – mutated).

**Results:** Somatic mutations associated with CH were identified in 3/30 SchS patients: two had different variants predicted as variants of unknown significance (VUS) in the *TET-2* gene and one had a pathogenic mutation in the *STAG2* gene. Though the latter may be a clonal hematopoiesis of indeterminate potential (CHIP) mutation. Of the aCAPS cases one patient had two pathogenic variants in *TET-2*. As additional evidence of CH, we utilised the HUMARA assay to identify X-allelic skewing in female patients, identifying solely one patient in each cohort as ‘XCI-skewed’. MYD88 L265P variant was detected in peripheral blood (PB) DNA of 9/30 SchS patients.Further deep sequencing of the *NLRP3* gene in 11 SchS patients failed to identify somatic variants.

**Conclusion:** The rate of CH in SchS and aCAPS is similar to what would be expected for this age group. Therefore, CH does not appear to be a common abnormality underpinning these conditions. The L265P *MYD88* variant was not universally found in all SchS patients. However, having not investigated bone marrow-derived DNA we cannot categorically exclude the presence of the *MYD88* variant in mutation negative cases. Lastly, including the 21 patients we have reported previously (1), we have now excluded somatic *NLRP3* mutations in 32 SchS patients. A molecular mechanism common to all SchS patients remains elusive. It is possible that this could be a heterogeneous disorder, however given the uniform clinical characteristics, including responsiveness to IL-1 inhibition, we favour the likelihood of a common aetiology, yet to be discovered.

**Disclosure of Interest**

None Declared

#### P1085 E148Q mutation in family with familial Mediterranean fever

##### Tamara F. Sarkisian, Hasmik Hayrapetyan, Anna Yeghiazaryan, Ruzanna Karakhanyan

###### Center of Medical Genetics and Primary Health Care, Yerevan, Armenia

####### **Correspondence:** Tamara F. Sarkisian

**Introduction:** Familial Mediterranean Fever (FMF) is a chronic autosomal recessive autoinflammatory disease with very high incidence and prevalence in Armenians. First information about recurrent inflammatory syndrome or polyserositis in Armenians was reported in ancient manuscripts of XII Century. It has been confirmed that MEFV (Mediterranean Fever) gene mutations play the main role in occurrence of severity of this disorder. MEFV gene encodes pyrin, the most important regulator of immunity processes. Mutated pyrin is responsible for inflammasome formation and increased inflammatory response. It was reported in “Infever Database” (2015) that more than 310 sequence variants of MEFV gene are revealed in 10 exons of Mediterranean Fever gene. The carrier rate of MEFV gene mutations is extremely high (about one in three) in Armenians. Distribution and spectrum of MEFV gene mutations are responsible for mild and severe forms of recurrent febrile and pain attacks, periodic episodes of fever, peritonitis, pleuritic, erysipelas-like skin lesions, arthralgia. Although FMF is autosomal recessive condition, but in more than 19% of our cohort of FMF patients have been shown to exhibit the same symptoms with heterozygous genotypes. In some heterozygous cases we detected atypical clinical forms of FMF.

**Objectives:** In some FMF patients “mild” MEFV mutations, such as P369S, A744S and E148Q, are responsible for the determination of severity of attacks in FMF, along with environmental or possible other genetic factors associated with inflammatory attacks. We have identified E148Q mutation in exon 2 of MEFV gene in three individuals of three generations of one family with confirmed clinical diagnosis of FMF.

**Methods:** They were genotyped using Viennalab Reverse Hybridization Strip Assay (Austria). Genetic and clinical data of more than 36000 individuals, including FMF patients and their family members, revealed statistically significance of phenotypes and distribution ofMEFV mutations covering more than 98% of the cases.

**Results:** In this family report we present the three FMF patients, including two males and one female of three generations. In the first generation 50 y.o. female has typical clinical FMF with periodic episodes for 1-2 days duration of fever, abdominalgia, thoracalgia, arthralgia, hepato- and splenomegaly. Disease manifested in age of 16 years old. Genetic investigation identified heterozygous E148Q mutation of exon 2. Treatment with Colchicine decreased the intensity of attacks to one-two episodes annually. The son of this patient also inherited the same heterozygous E148Q mutation.The disease onset with first attacks was observed when the boy was in age of 7 years old. The recurrent fever attacks of abdominal pain, myalgia had occurred for 5-6 days. Colchicine treatment decreased the frequency of attacks to 2-4 annually. His son also inherited the same E148Q heterozygous genotype from his grandmother and father. The boy was born in 2018. The disease manifested in 8 months with fever attacks.

**Conclusion:** Overall E148Q mutation rate among healthy individuals in our population is 3.4%. In heterozygous carriers of E148Q mutation our study shows an association with typical clinical symptoms of FMF in 3% of patients, and with atypical form - in 6% of FMF patients. Exon 2 mutations (E148Q etc) is correlated with milder disease with almost no arthritis and no amyloidosis. This rare heterozygous genotype resulted in genotype-phenotype associations.

**Consent for publication has been obtained from patient**

Yes

**Disclosure of Interest**

None Declared

#### P1086 Familial Mediterranean fever: clinical significance of E148Q and P369S mutations

##### Tamara F. Sarkisian, Hasmik Hayrapetyan, Stepan Atoyan, Anna Yeghiazaryan, Gohar Shahsuvaryan

###### Center of Medical Genetics and Primary Health Care, Yerevan, Armenia

####### **Correspondence:** Tamara F. Sarkisian

**Introduction:** FMF represents a significant health care problem in Armenia, because of high prevalence of the carriers of *MEFV* gene mutations (1:3) among population. Clinical-genetic investigations of 50-90 new FMF cases are performed weekly. Personalized treatment is based on MEFV genotypes and aimed to correct the effect of colchicine therapy.

**Objectives:** Disease severity varies from mild, moderate, severe depending on the spectrum of MEFV mutations. We present the evaluation of phenotypic features of FMF patients with E148Q (exon 2) and P369S (exon 3) mutations, suggested that E148Q and P369S are the mildest mutations, and some reports have questioned their association with FMF.

**Methods:** Over 36000 individuals, including FMF patients and healthy cohort, have been referred for clinical observation and molecular-genetic detection of MEFV and SAA1 genes mutations. Reverse-hybridisation assay is performing for genetic testing using the Viennalab StripAssay (Austria). Phenotypic, genotypic, and relevant information for patients have been stored in a large database.

**Results:** the frequency of carriers of MEFV mutations among Armenians is extremely high (about 20%: 1 in 3 individuals) with FMF prevalence of up to 3%. With the exception of M694I all other common mutations were present in healthy individuals with different frequencies. E148Q and P369S mutations are more frequent for healthy individuals in comparison to affected FMF patients, suggesting their lower penetrance compared to the others, but could still contribute to the overall FMF symptoms. Colchicine treatment is required.

Among 30 individuals with E148Q/E148Q homozygous genotype 9 have typical FMF, and 3 – non-typical symptoms. Abdominal pain was seen in 77% of the patients, fever in 92.3%, pleuritis in 30.7%, arthralgia in 61.5%. Compound heterozygotes and complex cases had a higher frequency of abdominal pain, fever, arthralgia, arthritis, myalgia, and chest pain than subjects who were homozygous for E148Q, but none of these symptoms reached statistical significance. None of the mentioned patients had developed renal amyloidosis, but two with E148Q/E148Q had a family history of amyloidosis and one had rapidly progressive glomerulonephritis secondary to vasculitis, which progressed to chronic renal failure. Among heterozygous carriers of E148Q mutation 333 had typical FMF, 1336 were without any symptom.

Homozygous genotype for P369S mutation was detected in only one healthy person. Number of healthy P369S carriers was 159, and in 41 diagnosis of FMF was confirmed.

In cohort of healthy controls, we detected 10 genotypes with P369S complex alleles, 7 without clinical picture of FMF, 3 manifested mild disease, suggesting that P369S might ameliorate the phenotypic effect of exon 10 mutations. Among E148Q/P369S compound-heterozygous 115 were healthy carriers, 34 are typical and 12 non-typical FMF.

In one family we confirmed FMF in 2 generations of E148Q/P369S compound-heterozygotes: two sisters with their children.

Atypical FMF phenotypes was found in: 3 homozygotes, 90 heterozygotes with E148Q mutation, 10 heterozygotes with P369S mutation.

**Conclusion:** Genetic counselling, including modern clinical-genetic approach is recommended for disease risk estimation in families with history of FMF, assessment of efficiency of colchicine treatment, prognosis of development of complications. Using a population-based study of healthy persons and FMF patients, we provide evidence that these alleles are a disease-causing mutations and not are a benign polymorphisms among Armenians. We suggest that in some cases other factors along with MEFV genotype, such as environmental or possibly other genetic factors play role in the determination of the severity of the inflammatory attacks in FMF.

**Disclosure of Interest**

None Declared

#### P1087 Severe prognosis of late onset CAPS associated with age related clonal hematopoiesis

##### Yael Shinar^1^, Rinat Cohen^1^, Victoria Marcu^1^, Itamar Goldstein^1^, Meital Nagar^1^, Gleb Slobodin^2^, Ilan Ben-Zvi^1^, Michael Rozenbaum^2^

###### ^1^Sheba Medical Center ,Ramat Gan; ^2^Bnai Zion Medical Center , Haifa, Israel

####### **Correspondence:** Yael Shinar

**Introduction:** Sporadic cases with adult Cryopyrin Associated Periodic Syndrome (CAPS) are associated with a somatic mutation in the *NLRP3* gene, often restricted to the myeloid lineage.

**Objectives:** We investigatedif mutations in myeloprliferative genes that are known to drive age related clonal heatopoiesis (ARCH) associated with the development of CAPS in a late onset case.

**Methods:** We sequenced 26autoinflammatory and 52 myeloproliferative genes in peripheral bloodDNA ofa 56 year oldpatient with late onsetCAPS, by the Next Generation Sequencing (NGS) method. The pathogenic mutations were screened in DNA derived from FACS-sorted CD3-positive T cells, CD14-positivemonocytes and granulocytes, and from single cells.

**Results:** A somatic *NLRP3* mutation, NM_001243133.1:c.1709A>Gp.Tyr570Cys, was present in DNA purified from granulocytes or monocytes (20% *NLRP3* gene reads) but was hardly detected in the patient's DNA from T cells (1%). Two pathogenic mutations linked with myeloproliferative disorders were detected: A known frame shiftmutation in the Tet methylcytosine dioxygenase 2 gene (*TET2,* in 31% ofthegene reads), and a known amino acid substitution in the Serine/arginine-rich splicing factor 2 (*SRSF2,* in 43% of the SRSF2 reads). Both had the same lineage pattern asthe *NLRP3* mutation, yet a higher somatic mosaicism rate than the *NLRP3* mutation in peripheral blood cells (PBC). Co-occurence of the *NLRP3* mutation with one or two loss-of-function *TET2* allele(s) was demonstrated in several single myeloid cells. There was no evidence of hematologic malignancy in the bone marrow or in a CT scan of the patient. Rather, the patient developed macrophage activating syndrome (MAS) that was also the cause of death after 8 years of disease.

**Conclusion:** Our results suggest the need to evaluate sporadic late onset CAPS cases for clonality of myeloproliferative genes, with prospect of dynamic and severe disease evolution, including MAS. In the case of *TET2*deficiency mutations a proinflammatory effect through the NLRP3 inflammasome may be suggested based on mice models. Clonal autoinflammatory diseases may constitute a distinct entity within the autoinflammatory diseases, characterized by clinical complexity and imposing therapeutic challenges.

**Consent for publication has been obtained from patient**

Yes

**Disclosure of Interest**

Y. Shinar Grant / Research Support from: Novartis Israel, R. Cohen: None Declared, V. Marcu: None Declared, I. Goldstein: None Declared, M. Nagar: None Declared, G. Slobodin: None Declared, I. Ben-Zvi: None Declared, M. Rozenbaum: None Declared

#### P1088 CNV analysis from targeted NGS data: case report on a patient with a large deletion in the NLRP12 gene

##### Barbara Bangol, Kathrin Starz, Martin Ziegler, Daniel Becker, Jenny Schiller, Sophie Eilitz, Hanns-Georg Klein

###### MVZ Martinsried GmbH, Martinsried, Germany

####### **Correspondence:** Kathrin Starz

**Introduction:** Diagnostic CNV (copy number variation) analysis in molecular genetics with exon level resolution is performed mainly by hybridization techniques (e.g. multiplex ligation-dependent probe amplification). Therefore, the examinable genomic region is restricted to commercially available test kits in most cases.

**Objectives:** The amount of clinically relevant CNVs located beyond the examinable region of these kits remains elusive. Hence, a bioinformatic pipeline for the detection of CNVs from NGS sequencing data was established (MIDAS pipeline). The analysis is routinely performed for patient samples in parallel to NGS testing for single nucleotide variations and small insertions/deletions by targeted enrichment and sequencing of candidate genes.

**Methods:** DNA samples from 115 patients with suspected autoinflammatory disease were subjected to NGS panel diagnostics including the genes *ELANE*, *IL1RN*, *IL36RN*, *LPIN2*, *MEFV*, *MVK*, *NLRC4*, *NLRP3*, *NLRP12*, *NOD2*, *PSMB8*, *PSTPIP1*, *TMEM173*, *TNFRSF1A.* With the exception of *MEFV*, no commercial CNV detection kits are available for these genes. In addition to routine NGS testing, binary alignment mapping files (BAM) were further analyzed via the MIDAS CNV pipeline. Coverage of targeted regions was normalized with control samples. CNV calls were performed using CoNVaDING, ExomeDepth and XHMM. Results were further investigated if CNVs were called from two out of three callers.

**Results:** Testing in a two-year-old Polish girl presenting with recurrent fever of unknown origin and an increased susceptibility to infections revealed a heterozygous deletion of at least 321 bp comprising exon 5 of the *NLRP12* gene (rsa[GRCh37] 19q13.42(54308439_54308760x1, 54310745x2). The deletion was confirmed by quantitative PCR (qPCR) and was not present in 240 control individuals.

**Conclusion:** There are known truncating germline mutations in the *NLRP12* gene, but no larger deletions have been described in association with the rare familial cold autoinflammatory syndrome 2 (FCAS2) so far. According to Richards *et al*. (2015) the variant was classified as likely pathogenic, which supports the clinical diagnosis of FCAS2 in the patient. The case described shows that application of CNV analysis of NGS data may increase the diagnostic sensitivity for the molecular diagnostics of hereditary recurrent fever. It is a cost efficient and convenient method to screen for copy number variations beyond regions which are covered by commercially available CNV detection kits. Since very few patients with FCAS2 have been identified, data on the phenotypic presentation is limited. Interestingly, Kostik *et al*. (2018) described several patients with clinical manifestations characteristic for primary immunodeficiencies and showing increased susceptibility to infections, similar to the present case.

**Consent for publication has been obtained from patient**

Yes

**Disclosure of Interest**

None Declared

#### P1089 Population based study of frequency of FMF gene mutation carrying in Armenia

##### Artashes Tadevosyan, Tigran Avagyan, Anush Budumyan, Hasmik Hayrapetyan, Gayane Amaryan

###### Public Health and Helth Care Organization, Yerevan State Medical University, Yerevan, Armenia

####### **Correspondence:** Artashes Tadevosyan

**Introduction:** Familial Mediterranean Fever (FMF) is a hereditary autoinflammatory disorder that is very common among Armenian, Turkish, Jewish populations. However, no study was conducted before among general Armenian population on epidemiology of FMF gene mutations.

**Objectives:** To study frequency of carrying of FMF gene mutations among Armenian population.

**Methods:** Random sample of 290 unrelated persons residing in Armenia, 179 of whom were female and 111 were male were selected. Blood samples were taken from them and analyzed. Identification of FMF and SAA1 gene mutations for 12 more common types of mutations based on PCR and reverse hybridization using ViennaLab diagnostic test was performed.

**Results:** This is the first population based study of carrying of FMF gene mutations among Armenian ethnic population in Armenia. Out of 290 participants 98 persons have at least one mutation, among which 59 were female carriers and 39 - male. That makes up 33.45% of the total participants. Out of 98 carriers 87 had heterozygous mode of inheritance, while 10 had compound-heterozygous and 1 had homozygous mode of inheritance.

Rate of mutation carriers among female participants was 1 out of 3. In female group 55 had heterozygous mode of inheritance (30.73%) and 4 had compound-heterozygous mode of inheritance (2.23%). Following mutations were found in this group: M694V, M680I, V726A, E148Q, K695R, A744S, R761H, F479L and P369S Highest number of cases of identified in female group was M694V (Table1).

Rate of mutation carriers among male participants was 1 out of 3. In male group 32 had heterozygous mode of inheritance (28.83%), 6 had compound-heterozygous mode of inheritance (5.4%) and 1 had homozygous mode of inheritance (0.9%). Following mutations were found in this group: V726A, M694V, E148Q, M680I, M694I, A744S and F479L. V726A was identified 17 times in male group (Table1).

**Conclusion:** The frequency of carrying the FMF gene mutation 1:3 is highest among ever reported.

Most common type is heterozygous mode of inheritance, which constituted for 88.8% among all carriers.

The most common mutation among all carriers is M694V - 32 cases, a little less - is carriage of V726A mutation (30 cases). At the third place - is carriage of Е148Q mutation (23 cases).

Acknowledgments

This work was supported by the RA MES State Committee of Science, in the frames of the research project № SCS 15T-3D191.

**Disclosure of Interest**

None Declared

**Table 1 (abstract P1089).** Number and rate (per hundred) of registered mutations

MEFV type	Among female carriers (number)	Among male carriers (number)	Among all carriers (number)
M694V	21(11.73)	11 (9.90)	32 (11.03)
V726A	13 (7.26)	17 (15.31)	30 (10.34)
E148Q	14 (7.82)	9 (8.10)	23 (7.93)
M680I	2 (1.11)	6 (5.40)	8 (2.76)
P369S	3 (1.67)	-	3 (1.03)
F749L	3 (1.67)	1 (0.90)	4 (1.38)
A744S	2 (1.11)	1 (0.90)	3 (1.03)
K695R	3 (1.67)	-	3 (1.03)
R761H/M694I	R761H-2 (1.11)	M694I-1 (0.90)	R761H -2 (0.69)/ M694I-1 (0.34)

#### P1090 Whole genome linkage and exome sequencing analysis in a CRMO family

##### Derya Yavuz^1^, Selcuk Yuksel^2^, Eda Tahir Turanli^1^

###### ^1^Department of Molecular Biology and Genetics, Istanbul Technical University, Istanbul; ^2^Department of Pediatric Rheumatology and Pediatric Nephrology, Pamukkale University , Denizli, Turkey

####### **Correspondence:** Eda Tahir Turanli

**Introduction:** Chronic recurrent multifocal osteomyelitis (CRMO), is an autoinflammatory disorder with incidence varying between 1 – 4 per million and it is most likely due to defects in TLR4/MAPK leading to in anti- and pro-inflammatory cytokine balance (ORPHA: 324964, PMID:29080202). CRMO occurs in childhood and adolescence, characterized with recurrent inflammation and lesions that are predominantly found in long bones. Reported familial cases and associated inflammatory conditions indicate genetic predisposition in the pathogenesis.

**Objectives:** In this study, we examined a consanguineous family with 2 affected and 1 healthy siblings and their healthy parents to identify the possible causative locus or gene.

**Methods:** Whole genome SNP genotyping by using Illumina OmniExpress-24 BeadChip that targets ~700,000 SNP markers was performed on all the family members that consists of 2 affected sibs, 1 healthy sister and their healthy parents. Resulting genotyping data was used for multipoint parametric linkage analysis that was performed under recessive model with complete penetrance. One of the affected siblings was screened by whole exome genotyping for assessment of possible disease-causing rare, homozygous variants. Variants obtained after filtration of exome data was furthered verified with the linkage analyses data.

**Results:** Linkage analyses revealed 137 homozygous regions out of which 44 are longer than 200 kb. Non-synonymous and homozygous variants gathered from the exome data were further filtrated according to 1000G and ExAC databases (MAF < 0.05), revealing 11 candidate variants in *NBPF6, DNAH14, MMADHC, KIAA0232, BODIL1, PCDHGB2, PCDHGA6, ZNF316, PWWP2B, CFAB46* and *CARNS1*. Also, variants in the genes that are listed in Infevers database were checked which revealed 4 intronic variants in *NLRP3, NLRC4, PSMA3* and *PSTPIP1* genes. Among these variants, rs12786101 in *CARNS1* (c.C1145T) and rs55638726 in *NLRC4* (g.32461493_32461494insC) were chosen as candidate variants.

**Conclusion:** One of the candidate variants that might be related to CRMO phenotype is located in *CARNS1* leading to a missense mutation whereas rs55638726 in *NLRC4* is found to be located in the splice site that might lead to aberrant splicing. However, these variants were found in homozygous regions that were shared in all the family members. Identification of disease causing potential of the detected variants are still on-going.

**Disclosure of Interest**

None Declared

#### P1091 Detection of a rare variant on PSTPIP1 gene through three generations in a Turkish family with systemic autoinflammatory disease

##### Merve Ö. Önen^1^, Serdal Ugurlu^2^, Ayse Balamir^1^, Eda Tahir Turanli^1^, Huri Ozdogan^2^

###### ^1^Department of Molecular Biology and Genetics, Istanbul Technical University; ^2^Department of Rheumatology, Istanbul University Cerrahpasa, Istanbul , Turkey

####### **Correspondence:** Eda Tahir Turanli

**Introduction:** Various monogenic recurrent fever syndromes share common clinical features. Therefore differential diagnosis of systemic autoinflammatory syndromes (sAIS) is a challenge, especially in familial Mediterranean fever prevalent countries.

Here we describe a family with Turkish descent, with 3 affected members belonging to 3 successive generations (grandmother, daughter, granddaughter) with self-limited, short episodes of fever, abdominal pain, oral ulcers, pharyngitis, arthralgia/arthritis, calf pain with increased acute phase proteins which respond to colchicine prophylactic treatment. None of the patients carry pathogenic MEFV variants.

**Objectives: To identify a causative gene** responsible for the autoinflammatory phenotype transmitted in 3 successive generations, in 3 members of the same family.

**Methods:** The index patient, the grandmother, was screened with Fever panel including the coding regions and UTRs of *MEFV, MVK, TNFRSF1A, NLRP3, NOD2, CECR1, TMEM173, PSTPIP1, NLRP12, LPIN2, PLCG2, CARD14, SLC29A3, IL10RA, NLRC4*. After annotation candidate variants were selected via filtering based on frequency and functionality. Candidate variant (p.Arg228Cys) on *PSTPIP1* gene was confirmed by Sanger sequencing in grandmother, daughter, and grandchild. Analysis of the possible effect of the variant on protein and transcription levels of *PSTPIP1*, *MEFV* and *IL1b* were performed through kit based-ELISA and Q-PCR methods with the protein and cDNAs, isolated from PBMCs of our three patients and five healthy controls.

**Results:** We detected a rare variant; p.Arg228Cys on *PSTPIP1* gene, in the index patient and confirmed the variant in her daughter and grandchild. Mutations in the PSTPIP1 gene is related with PAPA (pyogenic arthritis, pyoderma gangrenosum, acne) syndrome. This rare variant is located on F-BAR domain of PSTPIP1 protein, as other PAPA-related pathogenic variants, which is important for PSTPIP1 forming functional trimeric complex with pyrin protein. This binding results in conversion of pro-IL1b to IL1b.Protein levels of PSTPIP1, MEFV and IL1b of patients (44.78 ± 17.47mg/ml , 66.65 ± 31.81 mg/ml , 201.6 ± 63.54 mg/ml respectively) were higher than healthy controls (18.62 ± 3.741 mg/ml, 21.33 ± 6.738 mg/ml, 150.5 ± 15.22 mg/ml) respectively. However, when we calculate the fold change of transcript levels according to *GAPDH* endogenous gene, an increase in patients compared to healthy controls was detected only for PSTPIP1 (0.2928 ± 0.1324, 0.2136 ± 0.068).

**Conclusion:** The systemic autoinflammatory phenotype inherited through three generations in a Turkish family was related to p.Arg228Cys, a rare variant on *PSTPIP1.* However the mutations in PSTPIP1 gene are associated with PAPA, besides the autosomal dominant transmission, the phenotype of our patients is not compatible with the classical manifestations of this syndrome. Some of these FMF-like disease phenotypes may be attributed to locus heterogeneity such that similar phenotypes may be caused by mutations in different genes of a specific pathway.

**Disclosure of Interest**

None Declared

### Dermatology and Autoinflammation

#### P1092 Long-term treatment with ustekinumab for generalized pustular psoriasis in a child with heterozygous mutation in the IL36RN and TYK2 gene

##### Troels Herlin^1^, Mette Christiansen^2^, Sofie E. Jørgensen^3^, Christian Høst^1^, Mia Glerup^1^, Jens E. Veirum^1^, Dorte A. Larsen^4^, Lars Iversen^5^, Mia Glerup^1^, Birgitte Mahler^1^, Trine H. Mogensen^6^

###### ^1^Pediatrics; ^2^Clinical Immunology, Aarhus University Hospital; ^3^Biomedicine, Aarhus University; ^4^Ophthalmology; ^5^Dermatology; ^6^Infectious diseases, Aarhus University Hospital, Aarhus , Denmark

####### **Correspondence:** Troels Herlin

**Introduction:** Recessive mutations in the *IL36RN* gene have been identified as the genetic determinants for early onset generalized pustular psoriasis which in its homozygous form results in deficiency of interleukin-36-receptor antagonist (DITRA). Non-receptor tyrosine-protein kinase Tyk2, member of the JAK family, is encoded by the *TYK2* gene, which has been identified as a psoriasis susceptibility locus.

**Objectives:** To describe the striking long-term effect of ustekinumab, a monoclonal antibody against the p40 subunit of both IL-12 and IL-23, in a girl with DITRA phenotype with heterozygous mutation in the *IL36RN* and *TYK2* gene recalcitrant for several other treatments.

**Methods:** Girl, now 5-year-old, being 1^st^ child of unrelated Danish parents, prenatally diagnosed with tetralogy of Fallot (ToF). Array CGH revealed normal karyotype. Pregnancy and delivery were uneventful. Mother has been treated for hemorrhagic proctitis and psoriasis vulgaris, father is healthy. When 3-months-old she presented with scaly psoriasiform plaques. Shortly after operation for ToF at age 4 months she had a precipitous flare of numerous pustules covering more than 80% of skin area. Skin biopsy was compatible with pustular psoriasis.

Whole exome sequencing and Sanger sequencing was performed.

**Results:** Whole exome sequencing identified a heterozygous variant in the *IL36RN* gene (c.338C>T, p.S113L) with a high CADD score of 27.8 as well as a heterozygous variant in the *TYK2* gene (c.157G>A, p.A53T), CADD score 25.1. *IL36RN* was Sanger sequenced employing DNA from keratinocyte and melanocyte cultures from skin biopsies; the S113L variant was identified in DNA but not present in mRNA.

Methotrexate treatment was initiated together with topical corticosteroids without any effect on the cutaneous lesions. On suspicion of phenotypic equivalent to DITRA, anakinra (4 mg/kg/day increased to 8 mg/kg/day) caused some improvement although never complete regression of the pustular elements. After 8 weeks anakinra treatment was shifted to infliximab 6.5 mg/kg/dose given three times with 2 weeks interval and thereafter every 4 weeks. The skin lesions, as well as the general condition, improved markedly within few days, but after 2 months she developed a marked flare of pustular skin lesions. She developed an anaphylactoid reaction towards infliximab infusion and the medication was stopped. Treatment with etanercept did not show any improvement, in fact, she developed worsening of the pustulosis. Ustekinumab (0.9 mg/kg subcutaneously every 9 weeks) was then given which had a marked and sustained effect. The treatment with ustekinumab has been well tolerated and gradually anakinra, methotrexate and topical corticosteroids could be stopped. After 1 year of treatment, ustekinumab was paused to allow vaccination for MMR. However, after 5 months intermission she flared again with numerous pustular lesions and ustekinumab treatment was restarted, again with a striking efficacy. She has now been treated with ustekinumab for more than 48 months, the last 43 months as monotherapy.

**Conclusion:** Although only homozygous mutations in the *IL36RN* gene have been associated with DITRA we believe that in our patient the combined heterozygous variants in the *IL36RN* and *TYK2* gene play an essential role in the development of early onset generalized pustular psoriasis, not unlike DITRA syndrome. In contrast to anakinra and infliximab, which only demonstrated a short-lived and partial effect, ustekinumab monotherapy, which has been well tolerated, has enabled complete long-term remission for more than 4 years.

**Consent for publication has been obtained from patient**

Yes

**Disclosure of Interest**

None Declared

### Non-monogenic SAIDs (clinical)

#### P1093 Performance of newly proposed periodic fever, aphthous stomatitis, pharyngitis and cervical adenitis (PFAPA) syndrome criteria in regions endemic for famillial Mediterranean fever (FMF)

##### Amra Adrovic, Mehmet Yildiz, Neslihan Gucuyener, Ipek Ulkersoy, Melisa Kanber, Sezgin Sahin, Oya Koker, Kenan Barut, Ozgur Kasapcopur

###### Pediatric Rheumatology, Istanbul University, Cerrahpasa Medical School, Istanbul, Turkey

####### **Correspondence:** Amra Adrovic

**Introduction:** The periodic fever, aphthous stomatitis, pharyngitis, and cervical adenitis (PFAPA) syndrome is an autoinflammatory condition characterized by regularly recurrent episodes of high fever lasting 3 to 6 accompanied by aphthosis, cervical adenitis, and pharyngitis, in the absence of upper respiratory tract infections. Diagnosis of PFAPA could be challenging since it shares common characteristics with other auto-inflammatory conditions, including familial Mediterranean fever (FMF), hyper IgD syndrome etc. A relevant number of patients with monogenic periodic fevers also meet the diagnostic criteria for PFAPA syndrome. Especially in regions endemic for FMF, it would be important to avoid useless genetic testing and to be able to identify PFAPA patients by using clinical classification criteria. Despite the high specificity, widely used Marshall’s criteria have been shown to have low specificity. An international consensus among experts has been established recently, in order to define a set of classification criteria for PFAPA syndrome with a better performance in term of sensitivity and specificity.

**Objectives:** We aimed to evaluate the performance of recently proposed PFAPA criteria, in order to assess their utility in regions endemic for FMF.

**Methods:** Patients diagnosed with PFAPA syndrome, FMF and juvenile idiopathic arthritis (JIA) were consecutively included in the study. PFAPA diagnosis has been established by Marshall’s criteria. Patients with FMF and JIA were diagnosed according to Turkish pediatric FMF criteria and ILAR criteria, respectively. Two investigators blindly evaluated all of patients for the newly proposed PFAPA criteria.

**Results:** A total of 321 PFAPA, 118 FMF and 45 JIA patients with mean age of 7.23±2.9, 14.7±3.09, 13.5±4.6 years, respectively, were included in the study. A 45 % (146/323) of PFAPA syndrome, 50% (59/118) of FMF and 58% (23/45) of JIA patients were female. We found quite high sensitivity (90%) of newly proposed PFAPA criteria: 289 out of 321 (90%) patients followed up as PFAPA syndrome fulfilled newly proposed PFAPA criteria, as well. When applied to patients diagnosed with FMF and JIA, 46 out of 118 (39%) FMF and 10 out of 45 (22%) JIA patients also fulfilled newly proposed PFAPA criteria. Specificity of recently proposed PFAPA criteria was found to be 61% and 77%, among FMF and JIA patients, respectively. Positive predictive value was 86% and 97%, negative predictive value was 69% and 50% for FMF and JIA patients, respectively.

**Conclusion:** Recently proposed PFAPA criteria have satisfactory high sensitivity. Specificity of recently proposed criteria is still under expectation in regions endemic for FMF. Multicentric studies with higher patients’ number in different regions are needed in order to provide more relevant data on performance of newly proposed PFAPA criteria.

**Disclosure of Interest**

None Declared

#### P1094 Spine involvement in patients with non-bacterial multifocal osteomyelitis

##### Dmitry Alekseev^1^, Irina Nikishina^1^, Dmitry Zubkov^1^, Aleksandr Smirnov^2^, Olga Kostareva^1^

###### ^1^Pediatric Rheumatology; ^2^Radiology, V.A. Nasonova Research Institute of Rheumatology, Moscow, Russian Federation

####### **Correspondence:** Dmitry Alekseev

**Introduction:** Non-bacterial osteomyelitis (NBO) is a rare inflammatory disease of the musculoskeletal system, which is difficult to diagnose and treat. Assumed auto-inflammatory nature of the disorder.

**Objectives:** The analysis the features and outcome of axial skeleton (AxS) involvement in patients (pts) who were observed in our department for the last 10 years with multifocal NBO.

**Results:** Among the whole group of pts with NBO (n = 37) we identified 14 pts with AxS involvement including the lesions of the vertebral bodies and sacroiliac joints; the majority were girls (n = 10, 71.4%). Age at disease onset was 10.1 years in average (from 1.4 to 13.3, Me 9.5). There are no significant differences in clinical and laboratory features between children of different sex and age. Patients underwent standard rheumatologic examination; in order to examine all localizations of the skeletal lesion, a scintigraphy and/or “whole body” MRI scan was performed; a bone biopsy was performed on 5 pts. Other possible causes of damage were excluded, especially infection and oncology.

AxS lesions were localized in different parts of the spine: cervical – in 5 pts (35.7%), thoracic - 7 (50.0%), lumbar - 3 (21.4%), sacral - 1 (7.1%). Involvement of 2 spinal regions was detected in 5 pts, 3 regions - in 1. There was a lesion of the adjacent regions: cervical and thoracic - in 3 patients; thoracic and lumbar - in 1; cervical, thoracic and lumbar - in 1. In 4 pts 3 vertebral bodies were affected, in 2 - 4, in 8 - 1, in 1 - 17; lesion of the adjacent vertebrae was revealed in 7 pts (50.0%). MRI revealed inflammatory changes, with the presence of an increase in signal intensity in STIR images. In 6 pnts - destructive damage of the vertebrae, in 4 pts – with a decrease in the height of the vertebrae.Peripheral bone lesions were presented with damage of the bones of the extremities in 6 pts, metaphysis mostly (in 3 - the proximal femur, in 2 - the knee and ankle joints), in 1 patient - the distal radius, in 1 patient – tarsal. 9 pts had chronic arthritis of the limbs joints: in 5 pts oligoarthritis with lesions of the hip and knee joints, in 2 pts with polyarthritis with lesions of the hip, knee, ankle joints. Definite evidences of the sacroiliitis was found in 12 pts (85,7%), in 7 pts (50%) the diagnosis of Juvenile ankylosing spondylitis (JAS) fulfilled to New-York modified criteria were established, including severe variant with total ankylosis of sacroiliac and joints, vertebral bodies and multiple syndesmophytes in - 4 pts. But HLA B27 antigen was present just in 5 among 14 pts. Psoriasis of the palmar-plantar localization - in 1 patient; acnae conglobata - 1. Inflammatory bowel disease, uveitis - not identified. Laboratory parameters of activity were detected in the active period of the disease in 6 pnts: ESR acceleration up to 40 mm/h, increase in CRP - up to 73 mg/l; these patients had manifestations of chronic arthritis.

Treatment included NSAIDs (all), methotrexate (n=4), sulfasalazine (n=3), bisphosphonates (n=5), TNF inhibitors (n=6). Orthopedic measures: regimen with restriction of movements; during the destruction of the vertebrae, a corset was used (n=3), an operation with the fixation of the vertebrae with metal construction (n=3).

**Conclusion:** (1) AxS involvement in pts with NBO is a prognostic unfavorable sign associated with the rapid development of an advanced stage of JAS, including the development of syndesmophytes and ankylosis of intervertebral joints.

(2) Detection of highly active, according to MRI, sacroiliitis in children requires further examination for multifocal NBO.

**Disclosure of Interest**

None Declared

#### P1095 DNASE1L3 variant in hypocomplementemic urticarial vasculitis syndrome identifies a different clinical phenotype

##### Marco Ranalli^1^, Chiara Passarelli^2^, Virginia Messia^3^, Manuela Pardeo^3^, Emanuela Sacco^3^, Antonella Insalaco^3^, Marina Vivarelli^4^, Fabrizio De Benedetti^3^, Claudia Bracaglia^3^

###### ^1^Pediatric Department, La Sapienza University of Rome; ^2^Unit of Laboratory of Medical Genetics; ^3^Division of Rheumatology; ^4^Division of Nephrology, IRCCS Ospedale Pediatrico Bambino Gesù, Rome, Italy

####### **Correspondence:** Claudia Bracaglia

**Introduction:** Hypocomplementemic urticarial vasculitis syndrome (HUVS) is a rare disease characterized by persistent urticarial lesions and hypocomplementemia associated with systemic features involving musculoskeletal, pulmonary, renal and gastrointestinal systems. Systemic lupus erythematosus (SLE) develops in >50% of patients with HUVS, although the pathogenesis is unknown.

**Objectives:** We describe 6 paediatric patients with HUVS, three of whom carry a homozygous variant of *DNASE1L3* and present a peculiar clinical phenotype.

**Methods:** A Targeted Resequencing using a panel including genes already known to be mainly associated to Interferonopathies Lupus-like (*DNASE1*, *DNASE2*, *DNASE1L3*, *TREX1*) on the Illumina NextSeq® platform was performed. All variants identified were confirmed by Sanger sequencing and, when possible, family members were tested to study the segregation of identified variants. We applied in silico studies only to variants with an allelic frequency ≤1%.

**Results:** All patients described are Caucasian and 3 of them are female. Two patients presented at onset with extended cutaneous manifestation, joints and abdominal involvement with cholecystitis. They did not develop renal or pulmonary involvement. In contrast, the other four patients presented a more severe disease. All of them developed renal involvement (from microhaematuria up to nephrotic syndrome) with renal biopsy showing mesangial glomerulonephritis in three patients and pauci-immune glomerulonephritis (ANCA negative) in one. Moreover, two of them developed also pulmonary vasculitis (Table 1). A homozygous *DNASE1L3* variant (c.290_291delCA) was identified in three of these patients. All of them were treated with glucocorticoid and dapsone at onset. Cyclophosphamide, mycophenolate mofetil and azathioprine were used in patients with renal involvement. None of them developed SLE.

**Conclusion:** HUVS is very rare disease in childhood. Approximately 50% of HUVS patients develop SLE. Genetic susceptibility to SLE is recognized and *DNASE1L3*-related SLE have been reported. Özçakar et al. have described 5 children from two families with HUVS who carry the same variant on *DNASE1L3* that we report here (1). Our patients confirm that variant in *DNASE1L3* can cause HUVS and support the hypothesis that this variant is responsible of a more severe phenotype with major organ involvement (renal and pulmonary). Patients with HUVS need to be followed very strictly for the risk to develop SLE. Presence of variant in *DNASE1L3* can identify patients with more severe disease and high risk to develop major organ involvement. These patients need more aggressive and possibly life-long immunosuppressive treatment.

**Reference**

(1) Ozçakar ZB et al. *DNASE1L3 Mutations in Hypocomplementemic Urticarial Vasculitis Syndrome*. Arthritis Rheum. 2013 Aug;65(8):2183-9.

**Disclosure of Interest**

M. Ranalli: None Declared, C. Passarelli: None Declared, V. Messia: None Declared, M. Pardeo: None Declared, E. Sacco: None Declared, A. Insalaco: None Declared, M. Vivarelli: None Declared, F. De Benedetti Grant / Research Support from: Novartis, Novimmune, Hoffmann- La Roche, SOBI, AbbVie, Pfizer, C. Bracaglia: None Declared

**Table 1 (abstract P1095).** Patients’ clinical characteristics

	Pt 1	Pt 2	Pt 3	Pt 4	Pt 5	Pt 6
Age at onset	9 y 6/12	3 y 10/12	9y 5/12	14y 3/12	3y 6/12	3y 1/12
Joints	Yes	Yes	Yes	No	Yes	Yes
Abdominal	Yes	Yes	Yes	No	Yes	Yes
Pulmonary	No	Yes	Yes	No	No	No
Renal	Yes	Yes	Yes	Yes	No	No
Antibody anti-C1q	+	+	+	+	+	+
Anti-dsDNA	absent	absent	absent	absent	absent	absent
C3 (nv 90-180 mg/dl)/C4 (nv 10-40 mg/dl)	57/7	63/6	52/2	57/7	19/1	57/1
*DNASE1L3* variant	+	+	Ongoing	+	-	-

#### P1096 A case of idiopathic recurrent pericarditis treated with anakinra but unresponsive to canakinumab

##### Francesca Ricci, Silvia Ghetti, Rossana Razza, Camilla Dallavilla, Giulia Verzura, Alessandra Manerba, Marco Cattalini

###### University of Brescia and Spedali Civili di Brescia, Pediatric Clinic, Brescia, Italy

####### **Correspondence:** Marco Cattalini

**Introduction:** Recurrent pericarditis complicates up to 30% of acute pericarditis cases. First choice initial therapy are non-steroid anti-inflammatory drugs (NSAIDS) and colchicine. Corticosteroids have been used in case of partial response to first-line therapy, allowing rapid control of inflammation but with a high rate of steroid-dependency. Quite recently Anakinra, a recombinant human interleukin-1 competitive receptor antagonist that blocks both IL-1a and b, have been demonstrated to be a valid option in such cases. When to start Anakinra, and how to taper it in case of remission are still open questions, as if other anti-IL1 treatments may also be an option.

**Objectives:** We present herein a case of recurrent pericarditis well controlled by Anakinra, that became Anakinra-depent and relapsed after switching to Canakinumab, a recombinant human interleukin-1beta competitive receptor antagonist.

**Results: Results**: in September 2008, S.S., a 14-years old boy (weight 69,8 kg, height 160 cm) was admitted in our Emergency Department complaining of three days of acute chest pain that increased in supine position, fever and dyspnoea. A chest X-ray showed heart enlargement. Past medical history revealed that in the previous eight months, S. presented three similar episodes of chest pain, that were effectively home-treated with NSAIDS. Physical examination showed fair general condition, paleness and friction rubs at the cardiac auscultation. Lab tests revealed elevated CRP (270 mcg/ml) and ESR (79 mm/h). At trans-thoracic echocardiography revealed a diffuse pericardial effusion of 300 cc. Serologic studies didn’t suggest a clue for the aetiology of episode. Treatment with naproxen (250 mg twice a day for 7 days) and prednisone (initial dosage 30 mg/die) lead to rapid control of symptoms, inflammatory markers and US findings within few days. From there on, any attempt to taper steroids, even of few mg, led to rapid relapse of symptoms, inflammatory markers, and pericardial effusion. For this reason colchicine was added to initial treatment (1,5mg once a day) with no improvement and, after 3 months from the onset, anakinra (at the dosage of 100 mg/die) was introduced, with prompt normalization of the clinical picture and US findings. Steroids were withdrawn and in the following nine years numerous attempt were made to taper Anakinra but, every time the interval between injection was extended to 4 days, SS experienced a relapse. Genetic tests for TRAPS and Familial Mediterranean Fever resulted negative. In June 2018, we tried to modify the therapy by substituting anakinra with canakinumab, in order to reduced the number of injections. Unfortunately, 48 hours after the administration of canakinumab 150 mg, the patient experienced a flare with thoracic pain and high inflammatory markers. The flare was managed by steroids (prednisone 40mg a day) and, after two weeks, a second dose of Canakinumab was administered. Even after this up-titration it was not possible to taper steroids. Therefore, after an adequate period of wash-out, Anakinra was reintroduced with rapid and stable disease control and steroid withdrawal.

**Conclusion:** our case confirms that Anakinra may be a good option for recurrent pericarditis unresponsive to first line treatment, but drug withdrawal may be difficult. Although limited to this case, it seems that canakinumab is not a valid option. One may speculate that recurrent pericarditis (at least in some cases) is an IL-a – mediated disease

**Consent for publication has been obtained from patient**

Yes

**Disclosure of Interest**

None Declared

#### P1097 Efficacy of canakinumab as first-line biologic agent in adult-onset still’s disease

##### Giulio Cavalli, Alessandro Tomelleri, Corrado Campochiaro, Elena Baldissera, Giacomo De Luca, Lorenzo Dagna

###### Unit of Immunology, Rheumatology, Allergy and Rare Diseases (UniRAR), Vita-Salute San Raffaele University, Milan, Italy

####### **Correspondence:** Giulio Cavalli

**Introduction:** Adult-onset Still’s disease (AOSD) is a rare auto-inflammatory condition characterized by fever, arthritis, skin rash, and multi-organ inflammation. The pathogenesis of AOSD is centrally mediated by the pro-inflammatory cytokine interleukin (IL)-1β; anakinra, a recombinant inhibitor of the IL‑1β receptor, has thereby become the cornerstone of biologic therapy for AOSD. More recently, a new agent blocking IL‑1β has become available, that is, the monoclonal antibody canakinumab.

**Objectives:** So far, canakinumab has been used in the treatment of AOSD following failure of multiple lines of biologic therapy, including anakinra. Here, we describe the use of canakinumab as first-line biologic agent in 4 patients with AOSD, and report the highly promising results of this treatment approach.

**Methods:** Four patients with severe, DMARD-refractory AOSD received canakinumab at the approved dose of 4 mg/kg once every 4 weeks following failure of conventional treatment with corticosteroids and methotrexate. Patient characteristics and response to therapy were recorded.

**Results:** In all patients, treatment with canakinumab led to striking and rapid clinical responses, within days of initiation. Fever and skin rash disappeared first and did not recur, followed by progressive and sustained improvement in arthritis. Inflammatory organ involvement also responded to treatment: for example, we observed resolution of pericardial inflammation in two patients, as revealed by cardiac ultrasound; another patient had hepatosplenomegaly, which also reverted to normal upon treatment. A striking reduction in serum pro-inflammatory markers C-reactive protein, erythrocyte sedimentation rate, and ferritin mirrored the efficacy on clinical manifestations. These effects amounted to a significant decrease in the modified Pouchot score for measuring disease severity (average score before canakinumab treatment: 6; after: 1.25; statistical significance of difference evaluated with Mann-Whitney: p=0.02), and allowed for tapering or discontinuation of concomitant therapy: specifically, corticosteroid treatment was completely discontinued in two patients and substantially reduced in two patients, whereas methotrexate was stopped in one patient.

**Conclusion:** In this study, first-line biologic therapy of AOSD with canakinumab resulted in rapid and marked efficacy, ultimately leading to full clinical remissions in all patients, and allowing for robust steroid-sparing effects.

**Disclosure of Interest**

None Declared

#### P1098 CARRA autoinflammatory disease network development- improving care and fostering research in systemic autoinflammatory diseases (SAIDS)

##### Julie Cherian^1^, Theresa L. Wampler Muskardin^2^, Marinka Twilt^3^, Ronald M. Laxer^4^, Jonathon S. Hausmann^5^, Cagri Yildirim-Toruner^6^, Maria J. Gutierrez^7^, Nadine Saad^8^, Grant S. Schulert^9^, Evan J. Propst^10^, Greg Licameli^11^, Akaluck Thatayatikom^12^, Gil Amarilyo^13^, Liora Harel^14^, Ian C. Michelow^15^, Peter F. Wright^16^, Yuriy Stepanovskiy^17^, Lakshmi N. Moorthy^18^, Karen Durrant^19^, Polly J. Ferguson^20^, Lori B. Tucker^21^, Fatma Dedeoglu^22^, Sivia K. Lapidus^23^ and CARRA Autoinflammatory Network Development Work Group

###### ^1^Pediatrics, Stony Brook Children’s Hospital,Stony Brook, NY; ^2^Medicine and Pediatrics, NYU School of Medicine, NY, United States; ^3^Pediatrics, Alberta Children's Hospital, Calgary, AB; ^4^Pediatrics, The Hospital for Sick Children, Toronto, ON, Canada; ^5^Pediatrics, Boston Children's Hospital, Beth Israel Deaconess Medical Center, Boston, MA; ^6^Pediatrics, Nationwide Children's Hospital, Columbus, OH; ^7^Pediatrics, Johns Hopkins University School of Medicine, Baltimore; ^8^Pediatrics, Hospital for Special Surgery, NY; ^9^Pediatrics, Cincinnati Children's Hospital, Cincinnati, OH, United States; ^10^Otolaryngology, Hospital for Sick Children, Toronto, ON, Canada; ^11^Otolaryngology, Boston Children's Hospital, Boston, MA; ^12^Pediatrics, University of Florida, Gainesville, FL, United States; ^13^Pediatrics, Schneider Hospital, Tel Aviv University; ^14^Pediatrics, Schneider Hospital,Tel Aviv University , Tel Aviv, Israel; ^15^Pediatrics, Alpert Medical School of Brown University, Providence, RI; ^16^Pediatrics, Dartmouth-Hitchcock Medical Center,NH, United States; ^17^Shupyk National Medical Academy of Postgraduate Education, Kyiv, Ukraine; ^18^Pediatrics, Rutgers/RWJ Medical School, New Brunswick, NJ; ^19^Pediatrics, Kaiser Permanente San Francisco Medical Center, San Francisco, CA; ^20^Pediatrics, University of Iowa Carver College of Medicine, Iowa City, IA, United States; ^21^Pediatrics, BC Children's Hospital, UBC Vancouver, BC, Canada; ^22^Pediatrics, Boston Children's Hospital, Boston, MA; ^23^Pediatrics, Joseph M. Sanzari Children's Hospital Hackensack Meridian Health, Hackensack, NJ, United States

####### **Correspondence:** Julie Cherian

**Introduction:**

International registries have significantly enhanced the understanding of the genetics, phenotype, prognosis, and treatment responses in Systemic Autoinflammatory Diseases (SAIDs) in the studied populations, and such understanding would be further enhanced by expanding these efforts to include a genetically heterogeneous Northern American cohort.

**Objectives:**

To explore the value of developing a Childhood Arthritis and Rheumatology Research Alliance (CARRA) Autoinflammatory Disease Network in enhancing quality of care, therapeutic monitoring, and determining outcomes in SAIDs.

**Methods:** A team within CARRA composed of pediatric rheumatologists, infectious disease physicians, immunologists, otolaryngologists, geneticists, parents/patients with autoinflammatory disorders, and members of the Autoinflammatory Alliance met in person and via teleconference to discuss the benefits of autoinflammatory disease networks from 2016 to the present. Physicians who were involved in already-established autoinflammatory disease clinics in North America shared the benefits of having such programs. A comprehensive literature search on the topic using keywords such as “EuroFever”, “pharmacosurveillance”, and “autoinflammatory registry” were reviewed. Nominal group technique was employed.

**Results:**

A consensus was reached regarding the benefits, feasibility, and intent of developing a CARRA Autoinflammatory Disease Network that will be instrumental for providing quality care for children affected by SAIDs and their families. The network will provide improvements in areas of clinical care, enhanced research, patient and family access to accurate and useful disease-related education, and facilitation of international collaborations. The group’s literature search highlighted the benefits of this approach in rare diseases in preventing diagnostic delay leading to increased morbidity and mortality, understanding the epigenetics of SAIDs that remain poorly understood, and providing an opportunity for pharmacosurveillance in a cohort of patients potentially exposed to biologics.

**Conclusion:**

The development of a CARRA Autoinflammatory Disease Network will facilitate earlier diagnoses, education, and access to expert as well as multidisciplinary quality care.Additionally, this network will create an infrastructure for future clinical and translational investigations into long-term outcomes in SAIDs, epidemiological studies, comparative effectiveness trials, and implementation of optimum standards of care for SAIDs.Given the genetically diverse populations in North America, an autoinflammatory network would facilitate collaborations with international colleagues to benefit patients worldwide.

**Disclosure of Interest**

None Declared

#### P1099 The Helios (Hacettepe University Electronic Research Forms) registry: use of biologic drugs in autoinflammatory diseases

##### Selcan Demir^1^, Ezgi D. Batu^1^, Fuat Akal^2^, Erdal Sağ^1^, Ummusen Kaya Akca^1^, Elif Arslanoğlu Aydın^3^, Emil Aliyev^3^, Kübra Yüksel^3^, Armağan Keskin^3^, Yelda Bilginer^1^, Seza Ozen^1^

###### ^1^Department of Pediatric Rheumatology, Hacettepe University, Medical Faculty; ^2^Department ofComputer Engineering, Hacettepe University; ^3^Department of Pediatrics, Hacettepe University, Medical Faculty, Ankara, Turkey

####### **Correspondence:** Selcan Demir

**Introduction:** Autoinflammatory diseases (AID) are characterized by a dysregulation of innate immunity leading to uncontrolled inflammation. The treatment in AID is critical to control the disease activity, to prevent complications, and to improve the health-related quality of life. Biologic drugs have revolutionized the treatment and outcomes in AID.

**Objectives:**Herein we aim to present the clinical characteristics of children to whom biologic drug therapy was initiated for the management of AID.

**Methods:** A web-based registry called the Helios Registry (Hacettepe univErsity eLectronIc research fOrmS) has been formed to evaluate the data of all children on biologic treatment. We have been enrolling patients since August 2018 retrospectively and prospectively. We have analyzed the data about the general characteristics of the patients, treatment, the biologic drug used, and adverse effects. Only the patients with the following diagnoses were included: systemic juvenile idiopathic arthritis (SJIA), familial Mediterranean fever (FMF), cryopyrin associated periodic syndrome (CAPS), and chronic recurrent multifocal osteomyelitis (CRMO).

**Results:** Of 60 patients included, 19 had FMF (31.7%), 24 had sJIA (40%), 10 had CAPS,(16.7%), and 7 had CRMO (11.7%). Their median age was 10.7 (2-20) years old and disease duration was 2.8 (0-6) years, at the time of biologic drug initiation. 58.3% were currently on canakinmab, 20% anakinra, 10% tocilizumab, 10% etanercept, and 1.7% adalimumab. 63.3% of our patients had previously used at least one other biologic drug. The rate of glucocorticoid use before biologic treatment was 56.6%. The median duration of glucocorticoid treatment after initiating biologic drugs was 7.4 months.56 (93%) patients achieved remission on biologic therapy. There were 15 patients (25%) who received tuberculosis prophylaxis due to positive tuberculin skin test (diameter≥10 mm) and there was no Quantiferon test positivity. Thirteen adverse events (AE) had been noted. 2 of them were serious events as anaphylaxis due to tocilizumab infusions. The rest of the adverse events were mild thrombocytopenia (n=2), varicella infection (n=1), and local side effects (n=8). The median number of the infections per year was one and there were no death or malignancy.

**Conclusion:** The most commonly prescribed biologic drugs were IL-1 inhibitors especially for patients with IL-1-mediated AID (FMF, CAPS, and SJIA). The biologic treatment in AID seems effective and reasonably safe.

Acknowledgment

This registry was supported by SOBİ

**Disclosure of Interest**

None Declared

#### P1100 Pediatric Behcet’s disease with sinus venous thrombosis: three center experience from Turkey

##### Selcan Demir^1^, Ceyhun Açarı^2^, Özge Basaran^3^, Erdal Sağ^1^, Yelda Bilginer^1^, Şevket Erbil Ünsal^2^, Seza Ozen^1^

###### ^1^Department of Pediatric Rheumatology, Hacettepe University Faculty of Medicine, Ankara; ^2^Department of Pediatric Rheumatology, Dokuz Eylül University Faculty of Medicine, İzmir; ^3^Department of Pediatric Rheumatology, Ankara Child Health and Disease Hematology Oncology Training and Research Hospital, Ankara, Turkey

####### **Correspondence:** Selcan Demir

**Introduction:** Behçet’s disease (BD) is a multisystem vascular-inflammatory disorder including the cutaneous, articular, gastrointestinal, and/or central nervous systems (CNS).Adult BD patients with CNS involvement mostly present with parenchymal form, including meningoencephalitis, headache and pleocytosis of the cerebrospinal fluid while non-parenchymal form is more common among pediatric BD patients and younger age. Non-parenchymal form usually presents with cerebral venous sinus thrombosis (CVST) and intracranial hypertension. Neurological symptoms in children and adolescents can be confused with many other disorders and may be the initial symptom of BD.

**Objectives:** To report our experiences of the juvenile Behçet’s Disease patients with cerebral venous sinus thrombosis (CVST) and to review previous studies reporting the clinical characteristics and outcomes of Juvenile Behçet’s Disease with CVS

**Methods:** The patients who met Pediatric Behçet's Disease (PEDBD) classification criteria for juvenile Behçet’s Disease from 3 referral centers in Turkey were reviewed retrospectively. Disease activity was assessed by BD current activity form (BDCAF). A systematic review of literature of all published data was conducted.

**Results:** The study group consisted of 12 juvenile BD patients with CVST. At the time of CVST diagnosis, the most common symptom was headache (%100), followed by vomiting (25%), blurred vision (16.6-7%), and disturbances in eye movements (16.7%). Six (50 %) patients presented with sinus venous thrombosis as an initial symptom. Transverse sinus was the most frequently affected sinus (9/12, 75%) followed by superior sagittal sinus (8/12, 66.6%) and sigmoid sinus (1/12, 8.3%). The median (minimum-maximum) BDCAF was 6 (5-8). Four children (33.3%) had another venous thrombosis apart from CVST. All patients received pulse methylprednisolone for three consecutive days continued with oral prednisolone. Steroid treatment was tapered and discontinued minimum in six months. Eleven patients received azathioprine concomitant to steroid treatment at the time of CVST. All the patients received anticoagulant therapy concomitantly. Only one patient had relapse. Median (min-max) follow-up period was 4 years (1-10).In the literature review, we identified nine articles, describing 35 pediatric CVST patients associated with BD. Thirty patients achieved remission, while five patients had residual neurologic deficit.

**Conclusion:** Further multicenter studies with more patients and prospective follow-up may help us to understand the whole spectrum in these patients.

**Disclosure of Interest**

None Declared

#### P1101 PFAPA- the Irish experience in a tertiary autoinflammatory clinic

##### Ana-Louise Hawke^1^, Jayne MacMahon^1^, Emma Jane MacDermott^1^, Karina Butler^2^, Ronan Leahy^3^, Patrick Gavin^2^, Orla Killeen^1^

###### ^1^Paediatric Rheumatology; ^2^Paediatric Infectious Disease; ^3^Paediatric Immunology, Our Lady's Children's Hospital, Crumlin, Dublin, Ireland

####### **Correspondence:** Ana-Louise Hawke

**Introduction:** Periodic fever, aphthous stomatitis, pharyngitis and cervical adenitis (PFAPA) syndrome is the most common autoinflammatory disorder in childhood, with multifactorial, polygenic causes postulated.

Children usually present before the age of 5 years with periodic fevers and one or more associated symptoms of stomatitis, pharyngitis and adenitis, flares usually resolving in adolescence.

Treatment options include corticosteroids for flares and colchicine, tonsillectomy and increasingly biological agents to prevent disease flare.

**Objectives:** The aim of this study was to appraise the presentation and management of children with PFAPA attending a tertiary Autoinflammatory Clinic since it’s establishment in 2016.

**Methods:** A retrospective observational chart review of all children with PFAPA, confirmed or likely, attending Autoinflammatory clinic at Our Lady’s Children’s Hospital, Crumlin Dublin from January 2016.

Data were collected on basic demographics, age at presentation, route of referral, symptoms and signs and inflammatory markers during disease episodes and non-episodes. Details of genetics analysis if performed, were included. Information was also gathered on any treatment methods used and their effect including oral corticosteroids, colchicine, biological agents and tonsillectomy. Results were analysed using Microsoft Excel.

**Results:** 13 children were identified as having PFAPA, including 2 sets of siblings. The median age of disease onset was 16 months, ranging from 4 months to 4 years of age. The route to referral was through Immunology (4 patients), Rheumatology (6 patients) and Infectious disease (3 patients) clinics. All children presented with episodic, recurrent febrile episodes with a median duration of 3-4 days and a range of associated features. (See table 1) 3 children presented with the triad of stomatitis, adenitis and pharyngitis.

Sixty nine percent of patients had documented raised inflammatory markers during a flare, with 84% having high serum amyloid A levels, the highest documented being 1220mg/l (normal <10). 69% of patients had normal inflammatory markers and 76% normal serum amyloid A documented during disease quiescence.

Genetic testing in 6 children was negative for other causes of Hereditary Autoinflammatory disorders- TRAPS FMF.

2 sisters were found to have a benign variant NLRP mutation. Serum IgD levels normal in 9 and raised in 1 patient.

11 patients were initiated with treatment that included a stat of 3-day trial of oral corticosteroids, all of whom reported some improvement in symptoms, but 2 reporting rebound flares. 11 were treated with Colchicine, 9 of whom reported some benefit. Tonsillectomy was performed in 5 patients, 3 who found benefit. Anakinra was used to treat 2 and 1 patient was commenced on Adalimumab.

**Conclusion:** This study gives an over view of the burden of disease imposed by PFAPA on an Irish population. While all the children presented with fevers, not all had the classic triad of aphthous stomatitis, pharyngitis and cervical adenitis.Raised CRP ESR were seen in the majority of patients and 84% had raised amyloid levels during disease flare. The majority of patients had prompt relief of symptoms with an initiation trial of corticosteroid. Colchicine being frequently used with some success to prevent occurrence of flares. Tonsillectomy and biological agents were also used in some resistant/severe cases.

**Disclosure of Interest**

None Declared

**Table 1 (abstract P1101).** Number of patients

Associated Symtoms:	Stomatitis		8		
	Tonsillitis/Pharyngitis		8		
	Cervical Adenitis		7		
	Lethargy		6		
	Rash		4		
	Anorexia		4		
	GI upset		12		
Periodicity of disease flare	<2 weeks	1 patient	Flare Duration	2-3 days	5 patients
	2-4 weeks	10 patients		4-5 days	7 patients
	4-6 weeks	2 patients		6-7 days	1 patient

### Systemic-onset JIA and AOSD

#### P1102 Hepatitis A virus vaccination in autoinflammatory diseases under canakinumab and tocilizumab treatment

##### Kenan Barut^1^, Amra Adrovic^1^, Sezgin Sahin^1^, Mehmet Yıldız^1^, Oya Koker^1^, Gamze Yalcin^1^, Omer Faruk Beser^2^, Bekir Kocazeybek^3^, Pelin Yuksel^3^, Ozgur Kasapcopur^1^

###### ^1^Pediatric Rheumatology, Istanbul University Cerrahpasa, Cerrahpasa Medical School; ^2^Pediatric Gastroenterology, Okmeydani Education and Training Hospital; ^3^Microbiology, Istanbul University Cerrahpasa, Cerrahpasa Medical School, Istanbul, Turkey

####### **Correspondence:** Kenan Barut

**Introduction:** Autoimmune, autoinflammatory mechanism and drugs used in treatment increase the risk of liver disease in patients with chronic rheumatic diseases. Hepatitis A vaccine is a highly effective vaccine that prevents both the formation and spread of clinical hepatitis. In childhood chronic rheumatic diseases, vaccination is of great importance. The risk of various infections increases with the immunosuppressive effect of both the disease and the drugs.Therefore, vaccination of these diseases is of high importance for the prevention of infectious diseases. Systemic juvenile idiopathic (SJİA) arthritis is a juvenile idiopathic arthritis (JİA) subtype with autoinflammatory pathogenesis. Steroid, methotrexate and anti-interleukin 1 and 6 are used for the SJİA treatment. Anti-interleukin 1 treatments are also used in the treatment of Cryopyrin-associated periodic syndromes (CAPS), which is also characterized with autoinflammatory pathogenesis.Studies on the efficacy and safety of vaccines in autoimmune and autoinflammatory diseases are limited.

**Objectives:** The aim of this study was to investigate the efficacy and safety of hepatitis A vaccine in patients with autoinflammatory disease on anti-interleukin 1 and 6 treatment.

**Methods:** This study was carried out in Pediatric Rheumatology outpatient clinic and Healthy Child clinic between June 2017 and November 2018. A total of 39 patients with autoinflammatory diseases on anti IL-1 and IL-6 therapy were initially evaluated but 25 of them were excluded due to anti-HAV IgG positivity. At the end, 24 patients withautoinflammatory diseases on anti IL-1, anti IL-6 therapyand 39 healthy participants who were seronegative for hepatitis A received two doses of the hepatitis A vaccine in a 0 and 6 month schedule. Hepatitis A virus (HAV) IgG antibodies were measured before vaccination and one month after last dose of the vaccine. Anti-HAV IgG titer as S/ CO;1.1, IU/L was considered positive and protective.

**Results:** Total 24 patients with autoinflammatory condition (13 females, 11 males) and 39 healthy controls( 18 female, 21 male) were included in the study.

Among patients with diagnosis of autoinflammatory disease, 19 were SJİA and 5 were CAPS patients. The mean age was 14.1±3.7 and 12.2±3.3 years respectively. Canakinumab was used in 15 (62.5%) and tocilizumab in 9 (37.5%) all patients. Among all SJİA patients, 10 (52.6%) were treated with canakinumab and 9(47.4%) were treated with tocilizumab. All patients with CAPS (n: 5) were using canakinumab. Among SJIA cases, 15(75%) were also using methotrexate and 14(70%) prednisolone.Anti-HAV IgG concentrations were measured one month after the last dose of hepatitis A vaccine. There was statistically significant difference between patients with autoinflammatory condition and healthy controls regarding the anti-HAV IgG titer (mean 5.3±1.5 IU/L) versus (10.5±7 IU/L) p<0.05. The rate of anti-HAV IgG seropositivity (cut-off 1.1 IU/L) in autoinflammatory disease (24/24 (100%) was significantly different comparing to healthy controls (33/39, 84.6%) (p=0.04). There was no disease flare of disease nor the adverse event detected in any patients after vaccination.

**Conclusion:** Anti-HAV IgG seroconversion was detected in patients with autoinflammatory disease on anti-IL1 and anti - IL6 therapy 1 month after the last dose of hepatitis A vaccine. The response to vaccine did not differ between healthy children and patients with autoinflammatory disease under canakinumab and tocilizumab. In this study hepatitis A vaccine was found to be safe in autoinflammatory diseases with canakinumab and tocilizumab treatment.

**Disclosure of Interest**

None Declared

#### P1103 Risk score of macrophage activation syndrome in patients with systemic juvenileidiopathic arthritis

##### Simone Carbogno^1^, Denise Pires Marafon^2^, Giulia Marucci^3^, Manuela Pardeo^3^, Antonella Insalaco^3^, Virginia Messia^3^, Emanuela Sacco^3^, Fabrizio De Benedetti^3^, Claudia Bracaglia^3^

###### ^1^Pediatric Area, University of Milan; ^2^Pediatric Unit, Fondazione IRCCS Ca’ Grande Ospedale Maggiore Policlinico, Milan; ^3^Division of Rheumatology, IRCCS Ospedale Pediatrico Bambino Gesù, Rome, Italy

####### **Correspondence:** Claudia Bracaglia

**Introduction:** Macrophage Activation Syndrome (MAS) is a severe, life-threatening, complication of rheumatic diseases in childhood, particularly of systemic Juvenile Idiopathic Arthritis (sJIA), occurring in approximately 25% of the patients with sJIA. The mortality rate of MAS is still significantly high. A score that identify sJIA patients who are at high risk to develop MAS would be useful in clinical practice. There are no parameters available to identify from onset sJIA patients with high risk to develop MAS in their disease course.

**Objectives:** To evaluate whether routine laboratory parameters at disease onset may predict the development of MAS in patients with active sJIA. To define a risk score of MAS for sJIA patients using these parameters.

**Methods:** Laboratory parameters of disease activity and severity (WBC, N, PLT, Hb, ferritin, AST, ALT, gGT, LDH, TGL, fibrinogen, D-dimer and CRP), were retrospectively evaluated in 86 sJIA patients referred to our Division of Rheumatology from 1998 to 2017 with at least one year of follow-up. Laboratory parameters were evaluated during active sJIA, without MAS, at time of hospitalization (T1) and before treatment for sJIA was started (T2). Patients were divided in two groups: group 1 (patients without history of MAS), group 2 (patients with at least one MAS episode during disease course). To calculate a MAS risk score, laboratory parameters, collected at T2, with a statistical significant difference between the two groups of patients were selected.

**Results:** Thirty-three patients, that fulfilled the 2016 classification criteria for MAS [1] at time of sampling, were excluded from the analysis. Therefore, we analysed laboratory parameters of 53 patients with sJIA, 33 of whom without history of MAS (group 1) and 20 who developed at least one episode of MAS during disease course (group 2). Levels of ferritin, AST, LDH, gGT and TGL, collected at T2, were statistically significant higher in patients with a history of MAS compared to those without a history of MAS. For each of these parameters an arbitrary cut-off was defined. In order to define the final score an arbitrary rate was attributed to each parameter. Sensitivity (Se), specificity (Sp), positive predictive value (PPV) and negative predictive value (NPV) were calculated to define the best scoring system. The scoring system with the best sensitivity was chosen (Table 1). A MAS risk score >3 identified 19 out of 20 sJIA patients with a history of MAS and 4 out of 33 sJIA patients without history of MAS.

**Conclusion:** In conclusion we developed a MAS risk score based on routine laboratory parameters that are available worldwide, that can help clinicians to identify these patients early in the disease course. A validation in a larger population is ongoing.

**Reference**

[1] Ravelli A et al. 2016 Classification Criteria for Macrophage Activation Syndrome Complicating Systemic Juvenile Idiopathic Arthritis: A European League Against Rheumatism/American College of Rheumatology/Paediatric Rheumatology International Trials Organisation Collaborative Initiative. Ann Rheum Dis. 2016 Mar;75(3):481-9.

**Disclosure of Interest**

S. Carbogno: None Declared, D. Pires Marafon: None Declared, G. Marucci: None Declared, M. Pardeo: None Declared, A. Insalaco: None Declared, V. Messia: None Declared, E. Sacco: None Declared, F. De Benedetti Grant / Research Support from: Novartis, Novimmune, Hoffmann- La Roche, SOBI, AbbVie, Pfizer, C. Bracaglia: None Declared

**Table 1 (abstract P1103).** Laboratory parameters and cut-off used to create the MAS risk scorein sJIA patients

Laboratory parameters	Cut-off	Rate
Ferritin (ng/ml)	>900	1
AST (UI/L)	>35	1
LDH (UI/l)	>550	1
gammaGT (UI/L)	>30	2
Triglycerides (mg/dl)	>150	2
Sensitivity (Se)	0.950	CI95% 0.842-0.988
Specificity (Sp)	0.879	CI95% 0.753-0.948
Positive predictive value (PPV)	0.826	CI95% 0.692-0.912
Negative predictive value (NPV)	0.967	CI95% 0.865-0.995

#### P1104 Adult onset Still’s disease complicated with haemophagocytic syndrome: successfull treatment with IVIG and Tocilizumab

##### Ayse Cefle, Fatma Tuncer, Ayten Yazici

###### Department of Internal Medicine Division of Rheumatology, Kocaeli University Faculty of Medicine, Kocaeli, Turkey

####### **Correspondence:** Ayse Cefle

**Introduction:** Adult onset Still's disease (AOSD) is anacute febrile syndrome observed in young adults. Typically, many organs are affected. Haemophagocytic syndrome is a life-threatening condition with high mortality rate. The rate of occurrence of the condition in AOSD patients is 5-10%.

**Objectives:** A 24-year-old female patient with fever, rash, joint pain having started 1.5 months ago was admitted to our hospital. In physical examination; She had hepatosplenomegaly and multiple LAPs in bilateral cervical region as well as tenderness on the left wrist and right ankle. The rash was concomittant with fever. CRP, erythrocyte sedimentation rate, white blood cell and ferritin elevation were determined. Hepatitis markers, HIV, brucella, syphilis, CMV, toxoplasma, EBV were found to be negative. The anti-nuclear antibodies, rheumatoid factor and anti-CCP were negative,

**Methods:** In the thorax CT, multiple lymph nodes were observed in the mediastinal (13 mm), bilateral hilar, axillary (15 mm largest) and bilateral lower neck. The patient's PET CT revealed increased metabolic rate in the neck, mediastinum, abdominopelvic region, mildly diffused increased metabolism in the bone marrow, and increased metabolism in the basal sections of both lungs. It was reported that lymphoproliferative disease should be included in the differential diagnosis. Right servical lymph node excisional biopsy was reported as reactive lymphoid hyperplasia. Bone marrow biopsy came as normocellular. Her bronchoscopy was normal. Thereforemalignancy and infection were excluded. Prednizolon 60 mg/d and methotrexate 15 mg/w were started.

**Results:** The patient developed pancytopenia after two weeks and exhibited an increase in her triglyceride, D-dimer, LDH and hypofibrinogenemia developed. The patient had no atypical cells in the peripheral smear. IVIG as 2g/kg was added to the treatment with the diagnosis of haemophagocytic syndrome. After IVIG, her fever reduced, leukopenia thrombocytopenia improved, her sedimentation CRP reached normal limits. IVIG was administered 3 times with an interval of four weeks. However, one month after, she was admitted to our clinic with common myalgia, subfebrile fever, sore throat, swelling of the elbow, rash on the back. Leukocytosis with neutrophil dominance, increased levels of ferritin, LDH, eryhtrocyte sedimentation rate and CRP were noted. Her methylprednisolone dose was increased and Tocilizumab treatment (8 mg/kg/4w) was begun. After three months, clinical and laboratory findings were normal.

**Conclusion:** Macrophage activation syndrome is a reactive form of haemophagocytic syndrome associated with rheumatic diseases. 71% of macrophage activation syndrome cases occur within the first month of AOSD diagnosis. Fever, cytopenia, hypertriglyceridemia, increased ferritin, LDH and liver enzymes, HSM, LAP,coagulopathy and elevation of fibrin degradation products are observed. The syndrome is characterized with haemophagocytosis in bone marrow and RES. Glucocorticoids, cyclosporine, anakinra, tocilizumab and IVIG are administered.

**Consent for publication has been obtained from patient**

Yes

**Disclosure of Interest**

None Declared

#### P1105 Drug retention rate and predictive factors of drug survival for interleukin-1 inhibitors in systemic juvenile idiopathic arthritis

##### Carla Gaggiano^1^, Jurgen Sota^2^, Antonella Insalaco^3^, Rolando Cimaz^4^, Maria Alessio^5^, Marco Cattalini^6^, Romina Gallizzi^7^, Maria Cristina Maggio^8^, Giuseppe Lopalco^9^, Francesco La Torre^10^, Claudia Fabiani^11^, Manuela Pardeo^3^, Alma Nunzia Olivieri^12^, Paolo Sfriso^13^, Carlo Salvarani^14^, Salvatore Grosso^1^, Claudia Bracaglia^3^, Fabrizio De Benedetti^3^, Donato Rigante^15^, Luca Cantarini^2^

###### ^1^Clinical Pediatrics, Department of Molecular Medicine and Development; ^2^Research Center of Systemic Autoinflammatory Diseases and Behçet's Disease Clinic, Department of Medical Sciences, Surgery and Neurosciences, University of Siena, Siena; ^3^Division of Rheumatology, Department of Pediatric Medicine, Bambino Gesù Children's Hospital, IRCCS, Rome; ^4^Rheumatology Unit, Meyer Children's Hospital, University of Florence, Florence; ^5^Department of Pediatrics, University of Naples Federico II, Naples; ^6^Pediatric Clinic, University of Brescia, Brescia; ^7^Department of Pediatrics, Azienda Ospedaliera Universitaria Policlinico “G. Martino”, University of Messina, Messina; ^8^Universitary Department “Pro.S.A.M.I.”, University of Palermo, Palermo; ^9^Rheumatology Unit, Department of Emergency and Organ Transplantation, University of Bari, Bari; ^10^Pediatric Rheumatology Section, Pediatric Oncoematology Unit, Vito Fazzi Hospital, Lecce; ^11^Ophthalmology Unit, Department of Medicine, Surgery and Neuroscience, University of Siena, Siena; ^12^Dipartimento della Donna, del Bambino e di Chirurgia Generale e Specialistica, Seconda Università degli Studi di Napoli, Naples; ^13^Rheumatology Unit, Department of Medicine, University of Padua, Padua; ^14^Rheumatology Unit, Department of Internal Medicine, Azienda Ospedaliera ASMN, Istituto di Ricovero e Cura a Carattere Scientifico, Reggio Emilia; ^15^Institute of Pediatrics, Periodic Fever Research Center, Università Cattolica Sacro Cuore, Fondazione Policlinico A. Gemelli, IRCCS, Rome, Italy

####### **Correspondence:** Carla Gaggiano

**Introduction:** The advent of biologic agents has revolutionized therapeutic approaches in systemic juvenile idiopatic arthritis (sJIA) as their introduction has been shown to modify disease course and improve overall outcomes, particularly when initiated early. Few studies have reported the drug retention rate (DRR) of biologic drugs in JIA, and none of them has specifically investigated the DRR of interleukin (IL)-1 inhibitors on sJIA.

**Objectives:** The primary aim of the study was to examine the overall DRR of IL-1 blockers in sJIA patients. Secondary aims of our study were to: (i) explore the influence of biologic line of treatment, adverse events (AEs), type of anti-IL-1 agent and the concomitant use of conventional disease modifying anti-rheumatic drugs (cDMARDs) on DRR; (ii) find eventual predictive factors associated with events leading to drug discontinuation. The corticosteroid sparing effect and the impact of disease duration and treatment delay on survival constituted ancillary aims.

**Methods:** sJIA patients – diagnosed according to the revised International League of Association for Rheumatology (ILAR) criteria – treated with anakinra (ANA) and canakinumab (CAN) were enrolled in 15 Italian tertiary referral centers. Demographic, clinical and therapeutic data collected from medical records were retrospectively collected and statistically analyzed.

**Results:** Seventy seven patients were enrolled for a total of 86 treatment courses. The cumulative retention rate of the IL-1 inhibitors at 12-, 24-, 48-, and 60-months of follow-up was 79.9, 59.5, 53.5, and 53.5%, respectively, without any statistically significant differences between ANA and CAN (p = 0.056), and between patients treated in monotherapy compared to the subgroup co-administered with conventional immunosuppressors (p = 0.058). On the contrary, significant differences were found between biologic-naive patients and those previously treated with biologic drugs (p = 0.038) and when distinguishing according to AEs occurrence (p = 0.04). In regression analysis, patients pre-treated with other biologics (HR = 3.357 [CI: 1.341–8.406], p = 0.01) and those experiencing AEs (HR = 2.970 [CI: 1.186–7.435], p = 0.020) were associated with a higher hazard ratio of IL-1 inhibitors withdrawal. The mean treatment delay was significantly higher among patients discontinuing IL-1 inhibitors (p = 0.0002).

**Conclusion:** Our findings suggest an excellent overall DRR for both ANA and CAN that might be further augmented by paying attention to AEs and employing these agents as first-line biologics in an early disease phase.

**Disclosure of Interest**

None Declared

#### P1106 Clinic-laboratory profile of macrophage activation syndrome in children with systemic juvenile idiopathic arthritis

##### Sandesh Guleria^1^, Johnson Nameirakpam^1^, Anjani Gummadi^1^, Anju Gupta^1^, Amit Rawat^1^, Prateek Bhatia^2^, Deepti Suri^1^, Surjit Singh^1^

###### ^1^Pediatric Allergy and Immunology; ^2^Pediatric Hemato-Oncology, Postgraduate Instiute of Medical Education and Research, Chandigarh, Chandigarh, India

####### **Correspondence:** Sandesh Guleria

**Introduction:** Systemic juvenile idiopathic arthritis (SJIA) is an auto-inflammatory disorder with a propensity to develop macrophage activation syndrome (MAS). MAS is an emergent, fulminant, life-threatening condition which can be fatal if not recognized early.

**Objectives:** To study the clinic-laboratory profile of MAS in children with SJIA

**Methods:** Case records of children of SJIA who had MAS, diagnosed at the Advanced Pediatrics Centre, Post Graduate Institute of Medical Education and Research, Chandigarh, during the period 2017-2018, were reviewed. Findings pertaining to history, clinical examination and laboratory investigations were recorded. Diagnosis of MAS was based on criteria given by Ravelli et al (2016).

**Results:** A total of 13 children (1-18 years) with 15 MAS events were included in the study. Two children had 2 episodes each of MAS during this period. Mean age was 6.7+ 4.8 years with male to female ratio of 1.17. Five children (38.5%) had MAS, at or during hospital admission on their first presentation. Common clinical features noted were fever (100%), pallor (100%), hepatomegaly and/or splenomegaly (73.3%), rash (46.7%), lymphadenopathy (46.7%), arthritis (33.3%), irritability (26.7%), mucocutaneous bleeding (20%) and jaundice (13.3%). In laboratory investigations (Table 1), all children had anaemia and elevated C-reactive protein (CRP). Leucopenia and leucocytosis was seen in 2 (13.3%) and 8 (53.5%) children respectively. Four (26.6%) children had thrombocytopenia (< 150 x 10^9^/L), 6 (40%) had platelet count < 181 x 10^9^/L and three children (20%) had thrombocytosis. Hypertriglyceridemia (93.3%), hypofibrinoginemia (73.3%), elevated aspartate aminotransferase (73.3%) and alanine aminotransferase (40%) were other prominent laboratory parameters. All children had high serum ferritin levels with mean of 8960.6 + 13662.12 ng/ml. Nine children had serum ferritin/ESR (ratio) of > 80. Three (20%) children died. Two children, who died, had very high serum ferritin levels (52000 ng/ml and 42845 ng/ml).

**Conclusion:** MAS is a life threatening multisystemic complication of SJIA with high mortality rate (20%). Fever, pallor, organomegaly and persistent rash are prominent clinical features of MAS. Leucopenia and thrombocytopenia may not be early features of MAS but low or normal platelets can differentiate MAS from a disease flare in which we almost always get thrombocytosis. Very high serum ferritin, Hypertriglyceridemia, hypofibroginemia and transaminitis are better clue for MAS and serum ferritin levels may be a prognostic marker.

**Disclosure of Interest**

None Declared

**Table 1 (abstract P1106).** Laboratory parameters in children of SJIA with MAS

Variables	No. of patients	Mean + SD (n=15)
Anaemia	15 (100%)	84 + 19 g/L
Leucopenia	2 (13.3 %)	14.2 + 11.7 x 10^9^/L
Elevated CRP	15 (100%)	99.5 + 63.3 mg/L
Platelet < 181 x 10^9^/L	6 (40%)	283.5 + 182.4 x 10^9^/L
Elevated AST	11 (73.3%)	136.3 + 137.3 IU/L
Elevated ALT	6 (40%)	109.6 + 101.3 IU/L
Hypofibrinogenemia (<3.6 g/L)	11 (73.3%)	323 + 189 mg/dl
Hypertriglyceridemia(>156 mg/dl)	14 (93.3%)	235.9 + 132 mg/dl
Hyperferritinemia (ng/ml)	15 (100%)	8960.6 + 13662.12

ALT, alanine aminotransferase; AST, aspartate aminotransferase;CRP, C-reactive protein; ESR, erythrocyte sedimentation rate

#### P1107 Serum soluble CD25: an useful biomarker of macrophage activation syndrome in systemic juvenile idiopathic arthritis

##### Sandesh Guleria^1^, Anju Gupta^1^, Amit Rawat^1^, Prateek Bhatia^2^, Avinash Sharma^1^, Deepti Suri^1^, Surjit Singh^1^

###### ^1^Department of Pediatrics, Division of Allergy and Immunology; ^2^Department of Pediatrics, Division of Hemato-oncology, Postgraduate Instiute of Medical Education and Research, Chandigarh, Chandigarh, India

####### **Correspondence:** Sandesh Guleria

**Introduction:** Systemic juvenile idiopathic arthritis (SJIA) is an auto-inflammatory disorder secondary to innate immune dysfunction with a propensity to develop macrophage activation syndrome (MAS), a life-threatening condition. sCD25 has been used as a sensitive biomarker for the diagnosis of Hemophagocytic lymphohistiocytosis which has similarities in clinical features and pathogenesis to MAS.

**Objectives:** To assay serum soluble CD25 in children with systemic juvenile idiopathic arthritis (SJIA) and to compare levels of sCD25 in children with inactive disease, active disease and those with macrophage activation syndrome (MAS).

**Methods:** Thisprospective study was conducted in a tertiary care referral centre in North India from January 2017 to June 2018. All patients fulfilling the International League of Associations for Rheumatology (ILAR) 2001 criteria for SJIA were eligible for enrolment. At enrolment, all patients were examined clinically for signs of disease activity. Appropriate investigations were carried out and sCD25 was analyzed by using commercially available sCD25 / IL-2R ELISA kit.

**Results:** A total of 35 children (1-18 years) with 43 events were included in the study. Mean age at enrolment in the study was 7.3+3.59 years with male to female ratio of 2.5. Based on clinical features and investigations, events were categorized into 3 groups; SJIA with inactive disease (15; 34.9%), SJIA with active disease (15; 34.9%) and SJIA with MAS (13; 30.2%). Mean sCD25 levels in the study population were 10,966.02 + 10,854.93 pg/ml. Children with inactive disease, active disease and disease with MAS had mean + SD serum sCD25 levels of 4710.6 + 1817.04 pg/ml, 7604 + 2376.24 pg/ml, and 22062.9 + 14335.97 pg/ml respectively. Although, Mean level of sCD25 in children with active disease was higher than those with inactive disease, but no significant difference could be found. sCD25 levels significantly (p value 0.0001) varied between MAS and other 2 groups. sCD25 cut off level of 10,385 pg/ml was found to have sensitivity of 100% and specificity of 96.7% in differentiating MAS in SJIA from disease flare and inactive disease. There was no correlation of sCD25 with demographic parameters, Total leucocyte count, erythrocyte sedimentation rate (ESR), C-reactive protein (CRP), platelet counts, urea, creatinine, prothrombin time, activated partial prothrombin time and fibrinogen levels. A correlation of sCD25 was found with levels of haemoglobin (p-value .01), ferritin (p-value .001), aspartate aminotransferase (AST) (p-value .000), alanine aminotransferase (ALT) (p-value .000), alkaline phosphatase (ALP) (p-value .005) and triglycerides (p-value .001). Higher sCD25 levels were found in patients who had low Hb, elevated ferritin, elevated AST, ALT, ALP and serum triglyceride levels.

**Conclusion:** sCD25 is a useful biomarker in differentiating MAS in SJIA from disease flare and inactive disease with sensitivity and specificity of 100% and 96.7% respectively at a cut off level of 10,385 pg/ml. Coupling sCD25 with other laboratory parameters may be useful for early diagnosis of MAS in SJIA.

**Disclosure of Interest**

None Declared

#### P1108 Patient with CASP1 variant with severe systemic JIA and recurrent MAS treated by dual blockade with anakinra and tocilizumab

##### Troels Herlin^1^, Sofie E. Jørgensen^2^, Mette Christiansen^3^, Sofie E. Jørgensen^2^, Christian Høst^1^, Sofie E. Jørgensen^2^, Christian Høst^1^, Mette Christiansen^3^, Christian Høst^1^, Mette Christiansen^3^, Dorte A. Larsen^4^, Mia Glerup^1^, Dorte A. Larsen^4^, Trine H. Mogensen^5^, Birgitte Mahler^1^, Trine H. Mogensen^5^, Trine H. Mogensen^5^

###### ^1^Pediatrics, Aarhus University Hospital; ^2^Biomedicine, Aarhus University; ^3^Clinical Immunology; ^4^Ophthalmology; ^5^Infectious diseases, Aarhus University Hospital, Aarhus , Denmark

####### **Correspondence:** Troels Herlin

**Introduction:** Macrophage activation syndrome (MAS) is a severe complication of rheumatic disease in childhood, particularly in systemic juvenile idiopathic arthritis (sJIA). An increasing body of evidence suggests a genetic background. Human procaspase-1 variants have been associated with febrile episodes and may contribute to inflammation via binding of receptor interacting protein kinase 2 (RIP2) and thereby activate NF-kB signaling.

**Objectives:** To describe the genetic, immunological and therapeutic aspects of recalcitrant sJIA and recurrent MAS in a patient with a variant in the *CASP1* gene.

**Methods:** A 15-year-old, previously healthy girl presented Oct 2016 with a typical clinical and biochemical picture of systemic JIA.

Based on recurrent MAS, whole exome sequencing (WES) was performed on DNA extracted from whole blood. Peripheral blood mononuclear cells (PBMCs) from patient and a healthy control were stimulated with NF-kB inducers (TNFa and LPS or left untreated). Phosphorylated IkBa, as a measure of NF-kB activation, was measured by Luminex technology. Proinflammatory cytokines (IL-6, CXCL10, MIP-1a and TNFa) were measured in the supernatants using Mesoscale U-plex multiplex assays.

**Results:** WES identified a rare heterozygous variant in the *CASP1* gene (c.482G>A, p.R161H, CADD score 32; gnomAD frequency 0.000004) inherited from her healthy father and a rather frequent heterozygousvariant in the *UNC13D* gene (c.2335G>A, p.V779M, CADD score 15.3; gnomAD frequency 0.003). Patient PBMCs demonstrated increased NF-kB activation. Upon stimulation with TNFa and LPS the PBMCs produced extremely high levels of IL-6 and CXCL10 compared to control, whereas the levels of MIP-1a was comparable to control.

Initially, she responded well to anakinra 2 mg/kg/day and corticosteroids. Three months later she flared with intense pruritus, arthritis and quotidian fever and was changed to tocilizumab. Shortly after she precipitated with the picture of MAS and ferritin rise to 26,000 μg/L, elevated LDH, high triglyceride and low fibrinogen. Bone marrow was normal, spinal fluid showed mild pleocytosis, 36 leukocytes per μL. Etoposide and dexamethasone treatment were started according to the HLH-2004 protocol. Nevertheless, she developed another two episodes of MAS attacks with ferritin peaks >66,000 μg/L and two CNS attacks with generalized seizures (no cells in spinal fluid, but MRI with widespread bilateral cortical involvement). Despite treatment with canakinumab, dexamethasone, high-dose anakinra (8 mg/kg/day), several 3-day pulses with 1 G i.v. methylprednisolone and IVIG the condition deteriorated with repeated hyperferritinemia, fever, and elevated acute phase reactants.

Based on the findings of increased expression of IL-6 in stimulated PBMCs i.v. tocilizumab 8mg/kg was added every second week to high-dose anakinra, which had a marked clinical and biochemical response. All blood tests rapidly normalized and gradual tapering to low-dose anakinra and subcutaneous tocilizumab for the next 16 months did not precipitate new flares.

**Conclusion:** To the best of our knowledge, this is the first description of a *CASP1* variant associated with sJIA and recurrent MAS. We suggest that the Arg161His contributes to the phenotype, since it is located in the catalytic domain of procaspase-1 and therefore might stabilize the ASC Pyroptosome. The observed increase in NF-kB activation, possibly explained by procaspase-1 variants inducing stronger interaction with RIP2 than wild-type procaspase-1, may have contributed to the increased expression of proinflammatory proteins, such as IL-6.

**Consent for publication has been obtained from patient**

Yes

**Disclosure of Interest**

None Declared

#### P1109 Validation and clinical application of MRP8/14 serum levels as biomarker for the diagnosis of systemic juvenile idiopathic arthritis in fever of unknown origin

##### Carolin Pretzer^1^, María Miranda-Garcia^1^, Rainer Berendes^2^, Gerd Horneff^3^, Jasmin Kuemmerle-Deschner^4^, Gerd Ganser^5^, Hans-Iko Huppertz^6^, Kirsten Minden^7^, Johannes-Peter Haas^8^, Annette Jansson^9^, Michael Borte^10^, Catharina Schuetz^11^, Prasad Oommen^12^, Michael Frosch^13^, Christoph Kessel^1^, Bernhard Schlueter^14^, Annette Richter-Unruh^15^, Helmut Wittkowski^1^, Johannes Roth^16^, Dirk Foell^1^, Dirk Holzinger^17^

###### ^1^Department of Pediatric Rheumatology and Immunology, University Hospital Children’s Muenster, Muenster; ^2^St. Marien Children's Hospital, Landshut; ^3^Asklepios Clinic Sankt Augustin, Sankt Augustin; ^4^University Children's Hospital Tuebingen,, Tuebingen; ^5^St. Josef-Stift Sendenhorst Hospital, Sendenhorst; ^6^Prof.-Hess Children's Hospital and Gesundheit Nord Klinikverbund Bremen, Bremen; ^7^Charité University Medicine and Epidemiology Unit, German Rheumatism Research Centre, Berlin; ^8^German Center for Pediatric and Adolescent Rheumatology, Garmisch-Partenkirchen; ^9^Department of Rheumatology and Immunology, Dr. von Hauner Children's Hospital, Ludwig-Maximilians-University, Munich; ^10^Paediatric Rheumatology, Immunology and Infectiology, Hospital St. Georg, Leipzig; ^11^Department of Pediatrics and Adolescents Medicine, University Hospital Ulm, Ulm; ^12^Department of Pediatric Oncology, Hematology and Clinical Immunology, University Children's Hospital, Medical Faculty, Heinrich-Heine-University, Duesseldorf; ^13^German Pediatric Pain Centre, Children's and Adolescents’ Hospital, Datteln; ^14^Center for Laboratory Medicine, University Hospital Muenster; ^15^Department of Paediatrics, University of Muenster; ^16^Institute of Immunology, University Hospital Muenster, Muenster; ^17^Department of Pediatric Hematology-Oncology, University Hospital Essen, Essen, Germany

####### **Correspondence:** Dirk Holzinger

**Introduction:** The differential diagnosis of fever of unknown origin (FUO) is a major challenge especially for differentiation of systemic-onset juvenile idiopathic arthritis (SJIA) and infectious diseases and can be supported by biomarker analysis.

**Objectives:** In a pilot study the analysis of MRP8/14 serum levels has been demonstrated as an excellent tool for the diagnosis of SJIA, allowing early differentiation with a specificity of 95%. Based on this, the analysis of MRP8/14 serum levels has been offered to pediatric rheumatologists and we aimed to validate these findings in samples from daily clinical practice and establish the analysis by a commercial enzyme-linked immunosorbent sandwich assay (ELISA) and an on-site lateral flow immunoassay (LFIA).

**Methods:** In total, 3,002 patients with inflammation of unknown origin were enrolled within 6 years. A full data set was available from 1,935 cases, including symptoms, laboratory parameters (CRP, ESR, leucocytes) and final diagnosis. These cases were divided into two cohorts: (A) For validation of the MRP8/14 test performance with the enzyme-linked immunosorbent sandwich assay (ELISA) used previously as well as a first testing of a commercial ELISA and an innovative point-of-care lateral flow immunoassay (LFIA); and (B) for validating the findings with the latter assays.

**Results:** MRP8/14 serum levels of patients with SJIA (12,100 ± 2,670 ng/ml, mean±SEM) were elevated compared to other diagnoses (including infections, vasculitis and other autoinflammatory diseases; 3,020 ± 510 ng/ml) irrespective of fever and anti-inflammatory treatment (p <0.001) in 942 samples. In the group of untreated patients with fever (n=195) MRP8/14 levels in SJIA (19,930 ± 5,260 ng/ml) were even higher compared to other diagnoses (4,730 ± 1,160 ng/ml). Sensitivity and specificity of MRP8/14 to differentiate SJIA vs. other diseases were 72% and 89% respectively (cut-off 9,160 ng/ml). The MRP8/14 levels correlated closely in the commercial ELISA (sensitivity 74%, specificity 87%) and LFIA (sensitivity 77%, specificity 88%). The performance of the commercial ELISA and the LFIA could be confirmed in an independent cohort (n=162). Overall performance was stable in both cohorts: commercial ELISA (sensitivity 79%, specificity 88%) and LFIA (sensitivity 83%, specificity 87%).

**Conclusion:** MRP8/14 serum analyses could be validated as a helpful tool for the diagnosis of SJIA in FUO in daily clinical practice. The results of the experimental assay could be confirmed with a commercial ELISA and LFIA making these tests available for routine use. The LFIA enables a quick diagnostic screening test at the point of care.

**Disclosure of Interest**

None Declared

#### P1110 Do juvenile idiopathic arthritis patients with systemic onset in the risk of low anti-vaccine antibody: the preliminary report?

##### Natalya Lubimova^1^, Olga Goleva^2^, Irina Fridman^2^, Margarita Dubko^3^, Vera Masalova^3^, Ludmila Snegireva^3^, Eugenia Isupova^3^, Ekaterina Gaidar^3^, Andrey Santimov^3^, Olga Kalashnikova^3^, Susanna Kharit^2,3^, Mikhail Kostik^3^

###### ^1^Children’s City Hospital#2 n.a. St. Mary Magdalene; ^2^Pediatric Research and Clinical Center for Infection Diseases; ^3^Saint-Petersburg State Pediatric Medical University, Saint-Petersburg, Russian Federation

####### **Correspondence:** Mikhail Kostik

**Introduction:** Patients with juvenile idiopathic arthritis with systemic onset (soJIA) may have lower protective levels of anti-vaccine antibodies due to high inflammatory activity, frequent using of the systemic corticosteroids and biologics.

**Objectives:** The aim of our study was to evaluate the protective levels of anti-vaccine antibodies in patients with soJIA compare to non-systemic ones.

**Methods:** In the present study were included data 7 soJIA and 82 non-systemic JIA patients who received scheduled vaccination before the age of 2 years and before JIA onset against measles, parotitis, hepatitis B, diphtheria and rubella. In all patients, the IgG anti-vaccine antibodies levels were detected with ELISA. In each patient, we evaluate the type of the disease, onset age, duration of JIA, treatment with corticosteroids, methotrexate, and biologics.

**Results:** We have not found differences in the anti-vaccine antibody levels in soJIA compare to non-systemic, but median levels of the ant-hepatitis B and anti-diphthreia antibody were 12 and 3 times lower, consequently (table 1). The protective levels of anti-measels antibodies was in 4 (57%) vs 47 (57%). p=0.65; anti-parotitis antibody 5(71%) vs 63 (77%), p=0.37; ant-diphtheria 2 (29%) vs 42 (51%), p=0.23; ant-hepatitis B 2 (29%) vs 46 (56%), p=0.23; anti-rubella antibody 7 (100%) vs 80 (99%), p=0.77 in soJIA compare to non-systemic JIA, relatively. The number and levels of soJIA patients with protective anti-hepatitis B and anti-diphtheria antibody were lower compares to non-systemic JIA patients (data insignificant). There was no correlation with antibody concentration and corticosteroids applying, despite soJIA more frequently received them. We have found negative correlation between levels of ant-measels antibodies and JIA duration (r = -0.36, p=0.001), JIA duration (r=-0.26, p=0.02) and treatment with biologics (r=-0.25, p=0.024). Also was detected negative correlation between anti-parotitits antibody (r=-0.25, p=0.021) and anti-diphtheria antibody levels (r=-0.25, p=0.021) with JIA duration and and anti-diphtheria antibody levels (r=-0.29, p=0.009) with methotrexate using.

**Conclusion:** patients with systemic JIA may have the risk of lower anti-vaccine antibodies, which required more close monitoring of levels of these antibodies. Further investigations needed.

**Disclosure of Interest**

None Declared

**Table 1 (abstract P1110).** The levels of anti-vaccine antibody in soJIA and non-systemic JIA patients

Parameter	soJIA (n=7)	Non-systemic JIA (n=82)	P
JIA duration, years	3.0 (1.6; 5.1)	4.9 (2.9; 8.3)	0.019
Systemic corticosteroids, n (%)	7 (100	20 (24)	0.000007
Metotrexate, n (%)	7 (100.0)	75 (91)	0.41
Biologics, n (%)	7 (100.0)	47 (57)	0.019
Anti-measels IgG, Me/ml	0.18 (0.0; 1.64)	0.17 (0.0; 0.45)	0.649
Anti-parotitis IgG, Me/ml	1.3 (0.0; 5.1)	2.7 (1.0; 5.0)	0.373
Anti-diphtheria IgG, Me/ml	0.04 (0.0; 0.2)	0.13 (0.03; 0.3)	0.231
Anti-hepatitis B IgG level, Me/ml	0.9 (0.0; 10.2)	11.6 (0.0; 41.9)	0.231
Anti-rubella IgG, Me/ml	200.0 (18.4; 200.0)	57.7 (33.4; 98.0)	0.618

#### P1111 Have patients with juvenile idiopathic arthritis patients with systemic onset particular features for monocyclic course?

##### Mikhail Kostik, Maria Rumyantseva, Eugenia Isupova, Irina Chikova, Margarita Dubko, Vera Masalova, Ludmila Snegireva, Tatyana Kornishina, Tatyana Likhacheva, Ekaterina Gaidar, Andrey Santimov, Olga Kalashnikova

###### Saint-Petersburg State Pediatric Medical University, Saint-Petersburg, Russian Federation

####### **Correspondence:** Mikhail Kostik

**Introduction:** Juvenile idiopathic arthritis with systemic onset (soJIA) may have a monocyclic, polycyclic or relapsed course. There were not known soJIA course predictors.

**Objectives:** Our study aimed to evaluate initial clinical or laboratory features of the patients with soJIA who had a monocyclic course without biologics.

**Methods:** In the present study were included data about 130 soJIA patients. After selection, we identified a subgroup of the soJIA patients (n=22) who successfully achieved remission off-biologic medication and were stable in the remission off-medication at least two years. The second group consisted of the patients who required biologics (n=83). The remained 25 patients excluded due to missing data or who did not meet the selection criteria. We evaluated routine clinical (fever, rash, hepatosplenomegaly, serositis, lymphadenopathy, MAS, joint involvement) and laboratory (WBC, PLT, Hb, ferritin, ALS, AST, LDH, GGTP, ALP, albumin, sodium, triglycerides, ESR, CRP, prothrombin, fibrinogen) soJIA features in the onset of the disease.

**Results:** Patient with monocycling course have no any differences except the ferritin level: 275 (133; 698) ng/ml vs 950 (150; 3240) ng/ml, (p=0.04), time to achievement remission 17.7 (8.2; 38.0) months vs 60.2 (36.0; 99.0) months (p=0.00002) and rare elbow involvement 4.6% vs 30.9% (p=0.01). The parameters, associated with possible monocycling course are in the table.

**Conclusion:** Patients with soJIA initially have quite similar clinical presentations independently the further clinical course. The prediction of a possible course of soJIA is a difficult problem. Patients with soJIA with the monocyclic course were younger and had less intense laboratory activity. Further investigations required.

**Disclosure of Interest**

None Declared

**Table 1 (abstract P1111).** The parameters, associated with the possible monocyclic course in soJIA patients

Parameter	AUC (95%CI)	Se	Sp	OR (95% CI)	p
CRP≤3.1 mg/dl	0.59 (0.48; 0.69)	0.53	077	3.6 (1.3; 10.3)	0.014
ESR≤ 53 mm/h	0.62 (0.51; 0.71)	0.9	0.43	6.8 (1.5; 31.3)	0.006
Ferritin ≤1340 ng/ml	0.69 (0.58; 0.79)	1.0	0.46	-	0.003
Active joints < 8	0.5 (0.4; 0.6)	0.82	0.39	4.6 (1.3; 16.8)	0.013
Onset age <7 years	0.57 (0.47; 0.66)	0.86	0.4	4.2 (1.5; 15.3)	0.022
No elbow involvement, n (%)	-	0.96	0.31	9.4 (1.2; 73.6)	0.012

#### P1112 Single center experience of biological therapy in patients with systemic juvenile idiopathic arthritis associated with macrophage activation syndrome

##### Maria I. Kaleda, Irina P. Nikishina

###### Pediatrics, V.A. Nasonova Research Institute of Rheumatology, Moscow, Russian Federation

####### **Correspondence:** Irina P. Nikishina

**Introduction:** Macrophage activation syndrome (MAS) is a severe complication of systemic juvenile idiopathic arthritis (sJIA) which associated with high risks of the multiple organ failure and mortality. The investigation of safety of Biologics (B) in patients(pts) with risk of MAS is important in real clinical practice to improve the prognosis.

**Objectives:** to analyze the results of all cases of therapy with B in pts with sJIA and history of MAS.

**Methods:** the study included all pts of single center with sJIA (in accordance to ILAR classification criteria) who developed the MAS diagnosed according to Classification Criteria for MAS 2016 before or under the treatment with B.

**Results:** We observed 102 consecutive pts with sJIA, 29 pts (28%) fulfilled the criteria of MAS (Tab.1).

A total of 37 episodes of MAS was recorded. The total number of B course in this pts was 51 (tocilizumab – 28, canakinumab – 9, anakinra – 2, rituximab – 5, infliximab – 4, etanercept – 2, abatacept – 1). 24 pts (82.7%) had episodes of MAS before starting of B (16 – at onset of sJIA). 13 episodes of MAS was observed during the B therapy (1 on anakinra, 1 - canakinumab, 11 – tocilizumab), in 5 pts among them - for the first time. 8 episodes developed due to deviation of the treatment’s schedule, 2 – associated with flare of sJIA, 2 – associated with acute respiratory infection, 1 – after surgical treatment for chronic osteomyelitis of the clavicle. First features of MAS were bright rash with itching, lower platelet counts, high triglycerides. Pts who developed MAS while were treated with B had rare episodes of fever, a tendency for serum ferritin and CRP decreasing opposite to pts who developed MAS before B, but had no other differences in clinical or laboratory features. Glucocorticoids (per os+iv), intravenous immunoglobulin and cyclosporin were used to treat MAS. Lethal outcome was in 6.7% of sJIA cases with MAS (1.9% of all sJIA pts). 25 pts continue treatment with B (tocilizumab – 19, canakinumab – 6) after the resolution of the acute manifestation of MAS with high efficacy (more than 70-90% response by ACRpedi). Two pts discontinued treatment for organizational reasons.

**Conclusion:** Pts with a history of MAS require an earlier prescription of B due to the greater disease activity. It is necessary to consider the probability of a lower frequency of fever and lower level of ferritin and CRP in case of MAS during B therapy. We didn’t observe association between treatment of B and increased risk of MAS, but risk of MAS increase in case of disorder of protocol of the treatment.

**Disclosure of Interest**

None Declared

**Table 1 (abstract P1112).** Сlinical and demographic characteristics of patients

Characteristics	All patients with sJIA (102)	Patients with sJIA and MAS (29)
Girls /boys (girls to boys ratio)	59/43 (4:3)	18/11 (5:3)
The median age at onset, months	45.5 [26.0; 69.5]	31.0 [18.0; 69.0]
The median disease duration prior to treatment with BA, months	26.5 [9.2; 62.3]	11 [3.0; 30.1]
Number of systemic manifestations	3.9 [2.7; 4.5]	4.8 [3;6]
Pts with rash, %	56	100
Number of active joints	12 [5; 25]	8 [3; 18]

### Unusual or unsolved case reports

#### P1113 Behect disease in pediatrics; a solitary sign can be enough

##### Hanan M. Abd El-Lateef

###### Pediatric Rheumatology Immunology department, Ain Shams University, Cairo, Egypt

**Introduction:** Involvement of the perineal region including vulvovaginitisin prepubertal girls is a common but quite challenging condition because it encompass a wide variety of conditions with subtle differences especially in the absence of systemic manifestations

**Objectives:** Considering autoinflammatory disorders as an important differential diagnosis in cases of perineal ulcers in prepubertal females.

**Methods:** An 8 years old prepubertal female patient who presented with recurrent painful non itchy perineal ulcerations that interfered with her normal daily activitiesand copious non foul vaginal discharge of five months duration with failure of local topical therapies and systemic antibiotics. No other symptoms were reported.

**Results:** On physical examination,patient was average built for age andvitally stable. Examination of the perineal region revealed evidence of subcentimetric scars , an irregular deep ulcer 16 cm at its widest extension with necrotic base and clean margin involving perianal region and extending to labia majorus. Systemic examination revealed no evidence of arthritis, neurological manifestations, muscloskeletal involvement , other dermatologic or mucosal lesions and no organomegaly . Ophthalmological examination was free. Gynecological consultations excluded sexual abuse. Routine Laboratory evaluation showed a within normal CBC picture , ESR 50 mm/1^st^ hr and CRP 48mg/dl. Immunological profile showed a within normal levels serum immunoglobulins assay , CD markers and negative immune markers (ANA , Anti-DNA , ANCA) . Swabs from both the ulcer base and the vaginal discharge cultures were sterile. The diagnosis of Behçet disease versus Genital Crohn's was raised .Double contrast CT pelviabdomen with barium meal follow through showed skipping lesions of edematous bowel wall highly suspicious of Crohn's disease. Anegative ASCA test and a positive HLA-B51 tip the scales and favour the diagnosis of Behçet disease. The investigations showed no involvement of cardiovascular, renal, pulmonary, urologic, joint or central nervous systems. Patient showed marked clinical improvement on Pulse methylprednisolone steroid therapy (1gm/day) for 5 days followed by full dose oral prednisone (60mg/day) but with a progressive severe relapse of the ulcerations on the early withdrawal of the oral therapy (50 mg/day) . Azathioprine was added to the full dose oral steroid therapy for three months but lesions were still full dose steroid- dependant .The last attack was associated with a large pyoderma gangrenosum-like lesion on the anterior tibialsurface of the left leg that responded markedly on pulse steroid therapy.

Infliximab (Anti-TNFα ) was added (6mg/kg/dose) at 0, 2 and 6 weeks with cautious gradual withdrawal of oral steroids . Patient showed marked clinical and laboratory improvement and was maintained on 15mg oral prednisone per day with a stable remitting course. Further close follow up is done.

**Conclusion:** Behçet disease (BD) is a relapsing inflammatory disorder with multi-system involvement , heterogeneous clinical presentations and variable degrees of vascuilitis.BD shares a considerable overlap with other autoinflammatory disorders including Crohn's disease making diagnosis occasionally challenging . Perineal ulcers is a rare presentation in the pediatric age group and being an isolated clinical presentation confirms that in pediatrics the number of symptoms may be too few to apply any classification of diagnosis to a single patient. The severity of the ulcers , the relapsing nature , failure of azathioprine as asteroid sparing therapy lead to applying TNF-α inhibitors which proved a great efficacy to maintainremission.

**Consent for publication has been obtained from patient**

Yes

**Disclosure of Interest**

None Declared

#### P1114 Childhood pyoderma gangrenosum: diagnostic challenges and managements of two cases from Libya

##### Awatif Abushhaiwia^1^, Nairuz Abushhiwa^2^, Mohammed Kamel^3^, Lailasabei^4^, Mabruka Zletni^5^, Hafsa Dawi^6^

###### ^1^Pediatric Rheumatology, Tripoli Children’s Hospital; ^2^Pathology, Tripoli Faculty of Medicine, Tripoli, Libya; ^3^Adult Rheumatology, Dr Fakhry Hospital, Alkhobar, Saudi Arabia; ^4^Community Medicine, Tripoli Faculty of Medicine; ^5^Pediatric Rheumatology, Tripoli Children’s Hospital; ^6^Dermatology, Central Tripoli Hospital, Tripoli, Libya

####### **Correspondence:** Awatif Abushhaiwia

**Introduction:** Pyoderma gangrenousm (PG) is an idiopathic Ulcerative, non-infective chronic inflammatory skin disorder of unknown etiology.The most common reported underlying diseases in pediatrics are inflammatory bowel disease^,^ followed by hematologic disorders, vasculitis, immune deficiencies and pyogenic arthritis, pyoderma gangrenosum and acne (PAPA) syndrome. More than half of the cases occur with no underlying disease. The most frequently treatment should be tailored according to the underlying etiology. It includes systemic steroids, corticosteroid sparing agents such as dapsone, cyclosporine, azathioprine and currently TNF –alpha inhibitors such as etenrecpt, adalimumab and infliximab is a promising treatment of refractory PG. Response to treatment is high with cure rates reaching 90%. A high index suspicion and a through workup are mandatory in the management of pediatrics PG.

**Objectives**: To describe the clinical presentation, laboratory testes and challenges with treatment trials in two pediatric cases.

**Methods**: clinical information was gathered from the history, which was taken from their parents after an informed consent

**Results:** for the first time we report two pediatric cases in Libya who diagnosed with refractory pyoderma gangrenousm with no associated systemic diseases except PAPA which can’t be excluded since no genetic tests available in all public hospitals in Libya, we report PG in two Libyan children of 2 year-old and 5 year-old, they presented with skin lesions beginning as a small pustule that progressed to very painful large ulcer with irregular borders. There were healed ulcers with scaring present on chest, both upper and lower extremities and abdominal wall.Laboratory testes for both cases showed an increase in white blood cell counts mainly neutrophils, anemia, increased platelets count, elevated erythrosedmintation rate, and elevated C-reactive protein.No hematological abnormalities were seen in peripheral blood smears except neutrophilic response, the red cell indices were suggestive of iron deficiency anemia.Cultures for bacteria and fungi from skin lesions, and blood cultures were repeatedly negative. Immunoglobulin assay (IgG, IgM, IgA, IgE) was normal. The antinuclear antibody, cytoplasmic antibody, and antiphospholipid antibodies, tests for hepatitis B, C and HIV were all negative. They didn’t have symptoms suggest ulcerative colitis, their colonoscopy was normal. Histopathology from their skin was consistent with pyoderma gangrenousm.

Both cases were moderate response to high dose of oral prednisolone 2mg\kg per day complicated, however by growth retardation and cushingnoid habitus and minimal response to corticosteroid sparing agent azathioprine. Anti TNF-alpha inhibitors etenrecpt, which has been available at hospital since March 2018, we used it in case 2 because he was inadequate response to azathioprine.

Both cases showed briefly remitted on azathioprine with relapsed at least 2-3 episodes per year. The ulcer lesions healed with cribriform scaring within 6-8 weeks in each episode.

**Conclusion:** Pyoderma gangrenousm has recently been included within the spectrum of auto- inflammatory diseases, which are characterized by recurrent episodes of sterile inflammation, without circulating autoantibodies and autoreactive, which can be diagnosed by genetic tests, which is unfortunately not available in public hospitals in Libya.We couldn’t either be able to start other biological treatment because of the civil war crisis in Libya.

**Disclosure of Interest**

None Declared

#### P1115 A girl with a developmental delay and Lupus-like phenotype associated with both neuroblastoma and an MDA5 gain-of-function mutation (Aicardi-Goutières Syndrome 7, AGS7)

##### Stefan Berg^1^, Dara McCreary^2^, Gunilla Drake af Hagelsrum^3^, Nina Björkander^3^, Despina Eleftheriou^2^

###### ^1^Pediatric Immunology and Rheumatology, The Queen Silvia Children's Hospital, Gothenburg, Sweden; ^2^UCL GOS Institute of Child Health, London, United Kingdom; ^3^Pediatric Neurology, The Queen Silvia Children's Hospital, Gothenburg, Sweden

####### **Correspondence:** Stefan Berg

**Introduction:** Monogenic autoinflammatory diseases (AID) with central nervous system (CNS) involvement are often difficult to diagnose and patients may be subjected to expensive and often invasive diagnostic patient pathways. Securing a molecular diagnosis is of major importance in these cases for treatment, prognosis, and genetic counselling.

We describe a 7-year-old girl of non-consanguineous Swedish/Lebanese descent with severe developmental delay. Her psychomotor development was normal until 10 months of age. At the age of 10 months she presented with myoclonus and increased muscletone of both legs. She developed a progressive encephalopathy, behavioural change and sleep disturbance during the following months. Cerebral MRI showed white matter disease with high signal on T2 weighted imaging and atrophy. CSF showed normal proteins but minor lymphocytosis, and slight increase in neurological damage markers NFL and Tau (normal GFA-p). Extensive work-up did not reveal a clear diagnosis. Cerebral CT was not performed. At the age of 20 months a neuroblastoma was detected, leading to a diagnosis of atypical opsoclonus-myoclonus syndrome (OMS) despite the absence of opsoclonus, for which she was treated with cyclophosphamide, steroid pulses, IVIG and rituximab. By this time she demonstrated severe psychomotor delay with no speech and a dyskinetic syndrome.

At the age of 6 years she developed skin rash and vasculitis-like lesions on her toes, which developed to also involved the face and upper limbs. Her ESR was increased (47 mm/h), but CRP was normal (<1 mg/L). Titres of several autoantibodies were raised (ANA, anti-dsDNA and anti-ribo-p). Lumbar puncture showed slightly increased lymphocytes (7, ref <4 x 10^6^/L), and slightly elevated levels of Tau protein (normal NFL and GFA-p), with no increase of protein and no oligoclonal bands in the face of slight increase of IgM-index. Skin biopsy demonstrated findings compatible with SLE. Treatment with MMF and monthly i.v. corticosteroid pulses were required to control her features, resulting in a marked improvement of skin rash and normalization of inflammatory markers.

**Objectives:** To use next generation sequencing to establish a genetic diagnosis in this case.

**Methods:** An NGS panel targeting 256 genes was used developed and validated as described elsewhere.

**Results:** A pathogenic variant (p.R779H, c.G2336A) was found in the *IFIH1*gene encoding MDA5 consistent with a diagnosis of an interferonopathy syndrome, Aicardi-Goutières syndrome 7 (AGS7). IFN score is pending and we are planning to start JAK inhibition

**Conclusion:** We present a child with severe developmental delay having both a classic neuroblastoma and AGS7. She presented with neurodevelopmental delay at the age of 10 months and was found to have a neuroblastoma at the age of 20 months. She was considered to have an atypical OMS even though she didn’t demonstrate opsoclonus. Her developmental delay was more severe than usually seen in OMS. The findings of lupus-like features years later made us consider another cause for her neurological delay. The genetic finding of a pathogenic variant in *IFIH1*is fully compatible with a type I interferonopathy (AGS7). The fact that she also had a neuroblastoma may also have contributed to her neurological features, although this is not clear. To our knowledge, there is no connection between AGS and neuroblastoma described so far.

**Consent for publication has been obtained from patient**

Yes

**Disclosure of Interest**

None Declared

#### P1116 Cold urticaria associated with a novel Phospholipase-Gamma-C2 (PLCƔ2) mutation

##### Hanna Bonnekoh^1,2^, Niklas AMahnke^1,2^, Jörg Scheffel^1,2^, Marcus Maurer^1,2^, Karoline Krause^1,2^

###### ^1^Department of Dermatology and Allergy; ^2^Autoinflammation Reference Center Charité (ARC2), Charité – Universitätsmedizin Berlin, corporate member of Freie Universität Berlin, Humboldt-Universität zu Berlin, and Berlin Institute of Health, Berlin, Germany

####### **Correspondence:** Hanna Bonnekoh

**Introduction:** Phospholipase-Gamma-C2 (PLCƔ2) is a member of the phospholipase C family and plays a key role in the transmembranous signaling of B-lymphocytes, natural killer cells and mast cells. Mutations in *PLCG2* were associated with rare dominantly inherited diseases characterized by a variable clinical phenotype including cold urticaria, neutrophilic dermatitis, immunodeficiency and autoimmune features or autoinflammation (PLAID/APLAID). The known mutations comprise in frame loss mutations of exon 19 and exon 20-22 and a missense mutation (p.Ser707Tyr) within the PLCG2 gene.

**Methods:** We studied four members of a three-generation family, three of whom were affected by cold-induced urticarial rash. Genetic analysis of the index patient comprised screening for autoinflammatory periodic fever syndrome genes by Sanger sequencing (*NLRP3*, *NLRP12* and *PLCG2*) and was followed by segregation analysis of further family members. We assessed clinical history and laboratory results including routine laboratory markers, immune status, autoantibodies, immunoglobulins, inflammatory markers (C-reactive protein, S100A8/9) and performed cold contact provocation testing.

**Results:** All three affected family members presented with early onset cold-induced urticarial rash since birth associated with an autosomal-dominant inheritance. The patients reported occurrence of pruritic wheals to cold contact and cold evaporation as well as systemic reactions in response to extensive cold. Direct cold contact provocation testing was negative. Treatment with antihistamines could partially reduce the cold-associated skin-symptoms. Laboratory analysis revealed elevated serum S100A8/9 as well as elevated serum IgE levels and reduced IgM levels. Genetic analysis of the index patient showed a heterozygous variant c.2054+5G>T (located in the intronic splice region 18) in *PLCG2* which was confirmed in the two other affected family members.

**Conclusion:** We identified a novel mutation variant in *PLCG2* associated with a phenotype of cold-induced urticarial rash and immune dysregulation. As the substitution is located in the intronic splice region 18, it may cause aberrant splicing and result in truncated mRNA or mRNA decay. Further studies including the identification of the amino acid sequence as well as functional analysis of the PLCƔ2 protein are necessary to clarify the impact of this novel variant.

**Disclosure of Interest**

None Declared

#### P1117 Missense variants in SERPINF1 are associated with a novel auto-inflammatory disease presenting with persistent fever and multifocal neutrophilic periostitis

##### Marta Bustaffa^1^, Marta Rusmini^2^, Marco Cattalini^3^, Sara Signa^1^, Riccardo Papa^1^, Alice Grossi^2^, Roberta Caorsi^1^, Stefano Volpi^1^, Paolo Picco^4^, Alessandro Plebani^3^, Angelo Ravelli^4^, Nicoletta Zoppi^5^, Marco Ritelli^5^, Marina Colombi^5^, Maja Di Rocco^4^, Isabella Ceccherini^2^, Marco Gattorno^1^

###### ^1^International Center for Autoinflammatory Disease and Primary Immunodeficiencies - Clinics of Pediatrics and Rhumatology; ^2^UOC of Genetics, G. Gaslini Institute, Genova; ^3^Clinica Pediatrica, Univerity of Brescia, Brescia; ^4^Clinics of Pediatrics and Rhumatology, G. Gaslini Institute, Genova; ^5^Division of Biology and Genetics, Department of Molecular and Translational Medicine, Univerity of Brescia, Brescia, Italy

####### **Correspondence:** Marta Bustaffa

**Introduction:** Caffey disease is a rare condition of early infancy characterized by hyperostotic periostitis, acute inflammation and swelling of soft tissues. It is self-limiting and usually caused by the c.3040C>T p.Arg836Cys mutation in *COL1A1*. Besides Caffey disease, *COL1A1* mutations are reported in Osteogenesis Imperfecta (OI), with a well-known pathogenic mechanism. On the contrary, a link between the molecular defects of Caffey disease and its inflammatory, self-limiting phenotype is not properly understood. Another gene associated with OI is *SERPINF1*, whereas no inflammatory phenotypes are described related to *SERPINF1* mutations.

**Objectives:** To describe a case with many common characteristics with Caffey disease, but with a more severe course, steroid-dependency and no spontaneous regression.

**Methods:** The proband is a previously healthy 11-month-old girl born from non-consanguineous parents in a community with high risk of endogamy. She presented with persistent fever, elevation of inflammatory markers along with severe pain, functional limitation and progressive swelling of all four limbs. X-ray and MRI documented an aggressive periostitis involving several long bones. Pain was not controlled despite combined antalgic therapies, requiring i.v. morphine administration. Bone biopsy revealed a neutrophilic inflammatory process consistent with acute suppurative osteomyelitis, although broad-spectrum antibiotics were ineffective and an infectious origin was excluded. 41 NGS panel and genetic analysis of *COL1A1* excluded DIRA, MKD, Majeed and Caffey disease. With suspicion of a possible auto-inflammatory condition, Anakinra was started and allowed slight clinical improvement but elevation of acute phase reactants and bone swelling persisted.

**Results:** Considering the ineffectiveness of different treatments, high dose steroid therapy was introduced with dramatic improvement, maintained even when oral prednisone was started with slow tapering. Anakinra was confirmed as a possible steroid-sparing agent. Serial X-ray displayed persistent improvement until the age of 2 years, when radiology assessment revealed a subclinical disease progression and recurrence of swelling of the left wrist occurred when steroid discontinuation was tried. In the attempt to stop the subclinical disease progression, steroid therapy was maintained to the minimal effective dose. Anakinra treatment was tapered and finally discontinued without any flare. WES revealed compound heterozygosity for 2 missense variants in *SERPINF1* (A131D and P132R), inherited from parents. Immunofluorescence analyses of patient’s dermal fibroblasts revealed disorganized collagen type I, III and V in the extracellular matrix, endorsing a possible pathogenic role of both *SERPINF1* variants. Biphosphonate treatment was considered because of osteopenic risk due to prolonged steroid treatment and its potential use in OI. 4 doses of neridronate were administered within 9 months and subsequent X-rays displayed improvement of osteopenia and stability of the dysmorphic feature of left wrist.

**Conclusion:** We describe a patient with a clinical picture characterized by fever and multifocal neutrophilic hyperostosis without spontaneous regression, presenting dramatic response to high dose corticosteroid and subsequently to bisphosphonate. We suggest that this is a new variant of Caffey disease caused by *SERPINF1* missense variants.

**Consent for publication has been obtained from patient**

Yes

**Disclosure of Interest**

None Declared

#### P1118 Late-onset SAPHO syndrome revealing chronic myelomonocytic leukemia

##### Laura Damian^1^, Simona Grad^2^, Felicia Mustatea^1^, Bianca Balan^1^, Gheorghe Cobzac^3^, Calin Bolosiu^4^, Liliana Bene^5^, Adrian Trifa^6^, Delia Dima^7^, Adriana Albu^8^

###### ^1^Rheumatology, Emergency Clinical County Hospital Cluj; ^2^Gastroenterology, “Iuliu Hatieganu” University of Medicine Cluj; ^3^Nuclear Medicine; ^4^Radiology; ^5^Immunology, Emergency Clinical County Hospital Cluj; ^6^Genetics, “Iuliu Hatieganu” University of Medicine Cluj; ^7^Hematology, “I. Chiricuta” Oncologic Institute; ^8^2nd Internal Medicine , “Iuliu Hatieganu” University of Medicine Cluj, Cluj-Napoca, Romania

####### **Correspondence:** Laura Damian

**Introduction:** Chronic multifocal osteitis (CMO) and its adult variant, SAPHO syndrome (an acronym of synovitis, acne, hyperostosis, pustulosis, osteitis) are rare polygenic bone autoinflammatory disorders. However, their radiological features are not pathognomonic, as periostitis, bone sclerosis and cysts may be found in other systemic bone diseases, including myeloid neoplasia. Acquired gain-of- function JAK2 (617F) mutations, frequently found in myeloproliferative neoplasms, are sometimes associated to bone marrow fibrosis. JAK2 inhibitors may improve marrow fibrosis in myelofibrosis. JAK inhibition was also successfully employed in a case of refractory SAPHO syndrome.

**Objectives:** To present the difficult case of a patient with the clinical picture of a late-onset, incomplete SAPHO syndrome in whom aJAK2-positive myeloproliferative syndrome was found.

**Methods:** A 57-year male patient, with no personal or familial history of acne, psoriasis or spondylarthritis, presented with cervical and anterior chest wall intense bone pain, tibio-tarsal arthritis and left plantar fasciitis.

**Results:** The laboratory revealed inflammation (ESR 88 mm/h, CRP 18 mg/dl, leukocytosis – WBC 10500 with 70% polymorphonuclear neutrophilsand 11% monocytes), with normal alkaline phosphatase, plasma protein electrophoresis,PSA, CA 19-9 and CEA neoplastic markers.HLA-B27 was negative, as well as the tests for *Chlamydia, Mycoplasma, Ureaplasma, Borrelia* and hepatitis B, C and HIV serology. Ferritin was 501 ug/l. The radiographs showed left tibial periostitis, asymmetric periarticular bone demineralization, bone cysts, asymmetric sacroiliitis and a dorsal crush fracture. Bone scintigraphy revealed cervical, dorsal sternoclavicular and tibio-tarsal enhancement, with a” bullhead” appearance considered pathognomonic for SAPHO syndrome. Abdominal ultrasound, chest radiograph and CT were unremarkable. He received NSAIDs, sulfasalazine and methotrexate, supplemented afterwards with corticosteroids, cyclosporine and alendronate, with clinical improvement, but persistence of ESR elevation and monocytosis (up to 26%). The bone marrow biopsy showed all series, hypolobulated megakaryocytes and areas of myelofibrosis. The JAK2 (V617F) mutation was positive (allele burden 1%).BCR-ABL was negative. Cytogenetic analysis of the bone marrow revealed a normal karyotype. No free immunoglobulin chains were found. The final diagnosis was chronic myelomonocytic leukemia.

**Conclusion:** A late-onset and incomplete SAPHO clinical picture, in the presence of persistently abnormal blood counts, may prompt a search for hematologic malignancies. Common putative mechanisms of osteitis and bone fibrosis may be involved, making plausible a role of JAK inhibition for osteitis therapy.

**Consent for publication has been obtained from patient**

Yes

**Disclosure of Interest**

L. Damian Grant / Research Support from: Dr. Damian has a travel grant from Pfizer., S. Grad: None Declared, F. Mustatea: None Declared, B. Balan: None Declared, G. Cobzac: None Declared, C. Bolosiu: None Declared, L. Bene: None Declared, A. Trifa: None Declared, D. Dima: None Declared, A. Albu: None Declared

#### P1119 Bloody stools and cryopyrin-associated periodic syndrome (CAPS)-- association or coincidence?

##### Mariana Correia Marques^1^, Jonathan S. Hausmann^1,2^, Edwin Anderson^1^, Fatma Dedeoglu^1^

###### ^1^Boston Children's Hospital, ^2^Beth Israel Deaconess Medical Center, Boston, United States

####### **Correspondence:** Fatma Dedeoglu

**Introduction:** NLRP3 plays a role in intestinal homeostasis and has been implicated in the development of inflammatory bowel disease (IBD). Mutations in NLRP3 also cause the autoinflammatory disease cryopyrin-associated periodic syndrome (CAPS). Despite the involvement of NLRP3 in both IBD and CAPS, there are no reports of patients with CAPS who develop colitis and other manifestations of IBD.

**Objectives:** We report on 3 patients who initially presented with bloody stools and were later diagnosed with CAPS.

**Results:** Patient 1 had a history of recurrent painful hives since she was young, triggered by weather changes, stress, illness, or sun, but not cold. At 10 years of age, she developed recurrent uveitis, which was treated with topical steroids. She also had recurrent arthralgias. In her early 20’s she was diagnosed with Crohn’s disease when she developed abdominal pain and ileitis confirmed by colonoscopy. She was treated with mesalamine as needed during ileitis flares. At around the same time, she developed episodes of painful swelling of hands and feet, in addition to daily episodes of fever, fatigue, hives, nausea, and chills, lasting several hours. She had elevated inflammatory markers. She took hydroxychloroquine with no effect. In her 30’s she was diagnosed with CAPS with mutation A441V on the NLRP3 gene. She was started on canakinumab with resolution of most of her symptoms except for her arthritis and conjunctivitis, for which she remained on methotrexate.

Patient 2 is the son of patient 1. He had occasional fevers before 1 year of age, but at 1 year of age, he started having episodic hives and fevers, often triggered by weather changes but not cold. He had recurrent episodes of bloody stools and diarrhea, which did not improve after dietary restrictions. At around age 2, he started having episodes of ankle arthritis in addition to conjunctivitis without other signs of ocular inflammation.He had normal growth and development. He did not have a colonoscopy. After his mother was diagnosed with CAPS, he was tested and found to be positive for the same NLRP3 mutation. Anakinra was started on an as-needed basis to be used during episodes with good response.

Patient 3 developed feeding intolerance at around 6 months of age, which improved during his toddler years but recurred at 4 years of age, with the development of diarrhea, vomiting, abdominal pain, bloody stools, elevated inflammatory markers, and weight loss requiring NG tube feeding. He was seen by gastroenterology, and his Prometheus IBD panel was consistent with Crohn’s disease. He was empirically treated with steroids and sulfasalazine. A colonoscopy performed post-treatment did not confirm a pathologic diagnosis. He then developed recurrent episodes of fever and arthralgias lasting 1-2 days before self-resolving. He also suffered from recurrent headaches and there was a question of developmental delays and/or ADHD. At 9 years of age, whole exome sequencing showed the low-penetrance mutation R490K on the NLRP3 gene. His asymptomatic father was also found to be positive for the same mutation. Patient 3 was then referred to rheumatology. Further history revealed he had had urticarial rashes induced by heat and cold about twice per year. His hearing testing was normal. He was treated with anakinra, followed by canakinumab, with a good response except for rare breakthrough fevers, and for persistence of the gastrointestinal symptoms, including occasional bloody stools.

**Conclusion:** Patients with CAPS may present with overlapping IBD symptoms such as bloody stools, and failure to thrive that may mask the usual CAPS features, causing delays in diagnosis.

**Consent for publication has been obtained from patient**

Yes

**Disclosure of Interest**

None Declared

#### P1120 The challenge of treating pulmonary vasculitis in Behçet’s disease: two pediatric cases and literature review

##### Selcan Demir^1^, Erdal Sağ^1^, Ümmüşen Kaya Akca^1^, Tuncay Hazırolan^2^, Yelda Bilginer^1^, Seza Ozen^1^

###### ^1^Pediatric Rheumatology; ^2^Radiology, Hacettepe Medical Faculty, Ankara, Turkey

####### **Correspondence:** Selcan Demir

**Introduction:** Behçet’s Disease (BD) is a multisystemic autoinflammatory disease and the most severe complication of BD is pulmonary artery involvement (PAI). Data regarding treatment and outcomes of pediatric patients with PAI is very limited.

**Objectives:** Herein, we report two pediatric patients with BD presenting with PAI and treated successfully with aggressive immunosuppressive treatment.

**Results: Case 1**

A 15-year-old boy was admitted to a hospital with abdominal pain and feverAn abdominal Doppler ultrasonography (USG) showed stenosis of vena cava inferior (VCI) with a thrombus. Transthoracic echocardiography (TTE) detected that the thrombus extended from VCI to the right atrium. When he started to have hemoptysis, he was referred to our hospital. TTE showed a mass in RV. The computed chest and abdominal tomography angiography (CTA) showed bilateral aneurysmatic dilatation with thrombi in the pulmonary arteries and thrombosis in vena hepatica. The pathergy test was negative and the HLA B5 was negative. According to the ICBD, the patient had been diagnosed as BD due to genital ulcers and vascular involvement. He was given pulse methylprednisolone 500 mg 3 days along and followed by oral prednisolone 1 mg/ kg/day, intravenous cyclophosphamide at a dose of 500 mg (15 mg/kg) every 3 weeks for a total of 6 cycles, and Interferon-α2a (IFN-α2a) 3 times a week. Within one month, hemoptysis and fever disappeared, and CRP and ESR values normalized.After a three-month treatment, TTE and CTA revealed that thrombi shrank significantly. The dosage of prednisolone was tapered gradually and stopped 2 years later. Immunosuppressive treatment was continued with adalimumab. The patient has been followed in remission for nearly 6 years.

**Case 2**

A fifteen-year-old boy was referred to our hospital for the evaluation of fever for over 4 months and a thrombus in his right ventricle. He had a medical history with cough, fever, intermittent hemoptysis and weight loss (14 kg) for the past 3 months. Physical examination revealed acne-like rashes over face and back, ulcers on the buccal mucosa, a 3/6 systolic ejection murmur at the left upper parasternal area.A CTA confirmed the thrombus in RV and showed bilateral multiple aneurysms along the pulmonary artery and its branches. According to ICBD, the patient was diagnosed with BD due to having aphthous ulcers, pseudofolliculitis, and vascular involvement. Iv methylprednisolone (500 mg/day) for 3 days was followed by oral prednisolone 1 mg/ kg/day, which was subsequently tapered. Iv cyclophosphamide at a dose of 500 mg was also given every 3 weeks for a total of 6 cycles, followed by oral azathioprine (AZA). Concomitant subcutaneous IFN-α2a was given two times per week for 6 months. Within two weeks, cough and fever disappeared, CRP and ESR values normalized. After 1 year the pulmonary artery aneurysm disappeared and cardiac thrombosis resolved and returned nearly normal. We have been following the patient with AZA for four years without recurrence.

**Conclusion:** We present two pediatric patients with pulmonary involvement of BD. PAI is a life-threatening condition and should be managed with more aggressive medical therapy.Early diagnosis and aggressive immunosuppressive treatment are very important in PAI. We strengthened our treatment with IFN-α2a. There is no data in the literature regarding the use of IFN-α-2a in PAI treatment along with low dose cyclophosphamide. There were no mortality or recurrences within the 6 and 4 years follow up period. An aggressive immunosuppressive therapy leads to better prognosis in this most dreadful complication of BD.

**Consent for publication has been obtained from patient**

Yes

**Disclosure of Interest**

None Declared

#### P1121 Late diagnosis of cryopyrin-associated periodic syndrome - without gene confirmation - is there any other explanation?

##### Raquel Faria^1^, António Lamas^2^, Ana Campar^1^, Graziela Carvalheiras^1^

###### ^1^Unidade de Imunologia Clínica – CHUPorto; ^2^Serviço de Medicina - CHUPorto, Porto, Portugal

####### **Correspondence:** Raquel Faria

**Introduction:** Cryopyrin-associated periodic syndrome (CAPS) is a rare and heterogenous inherited autoinflammatory disease entity: a spectrum of clinical phenotypes associated with NLRP3 mutations. It is characterized by the presence of raised inflammatory markers and typical manifestations: urticaria-like rash, cold-triggered episodes, sensorineural hearing loss, arthralgia/myalgia, chronic aseptic meningitis and skeletal abnormalities.

**Results:** A 64-year-old woman presented psychomotor agitation and a recent diagnosis of left subclavian artery thrombosis with digital ischemia. She had a long history (since adolescence) of acute severe systemic inflammatory response syndrome, early-onset sensorineural hearing loss, seronegative arthritis refractory to NSAIDs, steroids, azathioprine and leflunomide, articular deformities of the wrists, knees and feet, bilateral tibial enchondromas and frontal bossing; recurrent episodes of uveitis (anterior and posterior), resulting in blindness; recurrent headaches associated with aseptic meningitis and negative temporal artery biopsy; and chronic kidney disease, category G4/A3 (KDIGO)*.* There was no history of recurrent fever, cold-triggered episodes or rash and no family history of autoimmune or autoinflammatory disease. Markedly elevated serum amyloid A, ESR (erythrocyte sedimentation rate) and CRP (C-reactive protein). Negative autoimmune panel. Non-nephrotic proteinuria and small kidneys on ultrasound. Cerebrospinal fluid analysis revealed elevated protein and glucose levels, without associated pleocytosis. Imaging, micro and mycobacteriologic studies were negative for infectious foci. Pre-mortem genetic analysis results were only available post-mortem, with negative NLRP3 mutation findings by PCR and Sanger sequencing.

**Conclusion:** This case report highlights the challenging diagnosis of an adult patient with severe manifestations and organ failure, that fulfils the clinical picture of an autoinflammatory disease, with longstanding uncontrolled inflammation resulting in irreversible organ damage (neurological, osteoarticular, ocular, among others) and complications (prothrombotic state, e.g.). Despite negative NLRP3 mutations, alternative inflammasome mutations (e.g. NLRP12) or mosaicism (not evaluated in the analysis) could explain it. The pattern of manifestations is highly suggestive of CAPS, fulfilling the diagnosis criteria proposed by *Kuemmerle-Deschner et al.* (2016), and represents a phenotype of increased severity in the CAPS spectrum in an adult patient. Given the patient’s age and clinical course, one may hypothesize an “attenuated” phenotype, possibly associated with genetic mosaicism. The diagnostic challenge of autoinflammatory diseases complicates early diagnosis and control of inflammation, which are critical to prevent irreversible organ damage, thus becoming of vital importance the recognition of autoimmune disease patterns and phenotypes.

**Consent for publication has been obtained from patient**

Yes

**Disclosure of Interest**

None Declared

#### P1122 Late onset of tumor necrosis factor receptor associated periodic syndrome (TRAPS) due to somatic mosaicism

##### Raquel Faria^1^, Luís Magalhães^2^, Daniel Oliveira^1^, António Marinho^1^, Ana Campar^1^

###### ^1^Unidade de Imunologia Clínica – CHUPorto; ^2^Serviço de Medicina - Hospital da Arrábida, Porto, Portugal

####### **Correspondence:** Raquel Faria

**Introduction:** Somatic mosaicism has been described as one of the major mechanism for autoinflammatory syndromes with late onset in adults.

**Results:** Case description:

We describe a 57 yo previously healthy woman, that after menopause (previous 9 years) developed recurrent 5-10 days episodes of high fever (39ºC), flutuant periorbital edema, abdominal urticarial rash, arthralgia/arthritis, myalgia, abdominal pain (no vomiting, rarely diarrhea), gengival pain, occasional cervical pain and “feeling flu-ish”. The episodes became more severe and frequent, the flu-like symptoms persisted every day. Fever responded to NSAIDs, not the rest, arthritic flares appear twice a month and she was not compliant to steroids. Blood analysis documented ESR >80mm/1st hour on attacks and 50mm/1st hour between attacks, C reactive protein 170mg/dL on attacks and 70mg/dL between attacks; and serum amyloid A ~90mg/dL between flares. Extensive infectious and autoimmune work lab panel was negative. The Eurofever Classification Criteria scored 74 points to CAPS (cryopirinopathies) and 88 points for TRAPS (Tumor Necrosis Factor Receptor Associated Periodic Syndrome). A PCR and Sanger sequencing detected c.176G>A (p.Cys59Tyr) in TNFRSF1A, in 20% of the whole blood sample in EDTA (compatible with somatic mosaicism) – validated pathogenic mutation. Colchicine 1mg/day had no effect on clinical symptoms or inflammatory markers. All her symptoms remitted and C reactive protein and serum amyloid A normalized wtih anakinra 100mg/day subcutaneous. No other family member (living parents or sibling) had ever had any fever or inflammatory symptoms.

**Conclusion:** The suspicion level for monogenic autoinflammatory diseases in adults should be high as the clinical picture might be atypical and of slower progression. Genetic testing should include mosaicism screening mainly in adults and a close collaboration with attending clinical physicians is crucial for the final diagnosis.

**Consent for publication has been obtained from patient**

Yes

**Disclosure of Interest**

None Declared

#### P1123 Hyperzincaemia and hypercalprotectinemia syndrome: more than just autoinflammation?

##### Andrea Uva^1^, Claudia Bracaglia^1^, Silvia Federici^1^, Camilla Celani^1^, Manuela Pardeo^1^, Christoph Kessel^2^, Fabrizio De Benedetti^1^, Antonella Insalaco^1^

###### ^1^Division of Rheumatology, Ospedale Pediatrico Bambino Gesù, Rome, Italy; ^2^Department of Pediatric Rheumatology & Immunology, University Children’s Hospital, Muenster, Germany

####### **Correspondence:** Silvia Federici

**Introduction:** Hyperzincaemia and hypercalprotectinemia (HandH) syndrome has been described as a new rare entity characterized by recurrent infections, dermatological involvement, increased inflammatory markers, hepatosplenomegaly and anemia. Little is known about its heterogeneous presentation, pathophysiology and treatment.

**Objectives:** To describe three cases with HandH syndrome

**Methods:** Serum calprotectin (MRP8/14) was measured according to Buehlmann assay (ELISA) and plasmatic zinc by atomic absorption spectrometry

**Results:** Three patients were referred to our centre because an history characterized by recurrent episodes of skin rash, severe oral aphtosis and increased level of serum amyloid A (SAA). Patient 1 presented, since the age of ten years, with recurrent episodes of fever and rash; skin biopsy showed a picture consistent with a lymphocytic lichenoid vasculitis resembling erythema multiforme. Patient 2 presented at birth, with hemolitic anemia and thrombocytopenia. At the age of 5 she was admitted to another hospital due to EBV related hemophagocytic limphohystiocytosis (HLH). At the age of 8, she was first seen at our center because of a persistent desquamant erythematous rash with recurrent abdominal pain and recurrent arthritis. Intestinal biopsy showed small intestine inflammation (erosions in the digiunum). Patient 3 presented with recurrent episodes of fever, rash, two episodes of transient hip synovitis and muscoloskeletal pain. A bone scintigraphy was performed resulting normal. Patients 1 and 2 suffered from recurrent infections (pneumonia, otitis, skin abscesses). Immunological studies revealed in patient 2 a reduction of memory B cells and a reduced response to Toll like receptor 9 agonist. Of note, all the patients presented in their medical history at least one episode of vasculitis: patients 1 and 3 suffered from Schonlein-Henoch purpura at the age of 11 and 3 respectivelyand patient 2 had at the age of 2 years an undefined vasculitis (evaluated elsewhere). Laboratory tests showed in all patients elevated inflammatory markers, zinchemia and serum calprotectin (table)

**Conclusion:** We report three patients with high serum levels of calprotectinemia and zinch presenting with a clinical phenotype consistent with previously reported cases. The presence of vasculitis in all of the patients suggests that it may represent the first symptom of this condition. Vasculitis could lead to an increase of serum calprotectin already proposed as a marker of vascular impairment. Moreover, considering the immunological defect detected in one of our patient, we speculate that recurrent infections described in this syndrome may underline an immune-dysregulation process in which the role of zinc metabolism needs to be assessed

**Consent for publication has been obtained from patient**

Yes

**Disclosure of Interest**

None Declared

**Table 1 (abstract P1123).** See text for description

	Patient 1	Patient 2	Patient 3
Age at 1^st^ evaluation (years)	14.3	7.8	4.9
Sex	F	F	M
Clinical features	Recurrent fever, recurrent infection, vasculitis, skin rash, musculoskeletal pain	Hemolitic anemia,thrombocytopenia, HLH, vasculitis, recurrent infections, recurrent fever, arthritis, skin rash, gastrointestinal involvement	Recurrent fever, skin rash, vasculitis, arthritis, musculoskeletalpain
SAA	13-23	27,5-76,4	20
Zinchemia(mcg/dl) (n.v. 80-125)	132-143	102-233	147
Serum MRP8/14 (ng/ml) (n.v. < 2900)	22431	10383	10363

#### P1124 Inherited deficit of proteoglycan mimicking septic arthritis

##### Angelo Florio^1^, Riccardo Papa^1^, Roberta Caorsi^1^, Alessandro Consolaro^1^, Tuula Rinne^2^, Roberto Gastaldi^3^, Angelo Ravelli^1^, Marco Gattorno^1^, Paolo Picco^1^

###### ^1^Clinica Pediatrica e Reumatologia , IRCCS Istituto Giannina Gaslini, Genova, Italy; ^2^Department of Human Genetics, Radboud University Medical Center, Nijmegen, Netherlands; ^3^Clinica Pediatrica ed Endocrinologia, IRCCS Istituto Giannina Gaslini, Genova, Italy

####### **Correspondence:** Angelo Florio

**Introduction:** Aggrecanopathies are a heterogeneous group of skeletal disorders caused by *ACAN* gene mutations leading to aggrecan dysfunction: this latter is a proteoglycan with a pivotal role in extracellular matrix of the growth-plate cartilage organization. Clinically, aggrecanopathies displayed bone dysplasias, idiopathic short stature and facial dysmorphisms. Herein we report a patient with a prevalent inflammatory articular involvement, e.g. osteochondritis dissecans (OCD) mimicking a septic arthritis.

**Methods:** A 14-year-old boy displayed pain and swelling at the right elbow. Echo-scan revealed an effusion in both coronoid and olecranon recess. Acute phase reactants were negative. Since non-steroidal anti-inflammatory drugs were ineffective, the patient was admitted in our Institute, a month later clinical onset.

On physical examination, acute arthritis at right elbow was present, which appeared painful, warm, not erythematous. Laboratory test showed slight elevated inflammatory markers (C reactive protein 1.7 mg/dl, normal value <0.5). Arthrocentesis of the right elbow was performed: sterile synovial fluid was found. Magnetic resonance images of the right elbow displayed bone fragment detachment from humeral condyle with thickening of the synovium and persistent local effusion: the diagnosis of OCD was pointed out.

It is worth nothing that patient showed minor dysmorphisms (i.e. dolicocephaly, hypotelorism, arched palate and brachydactyly of the IV finger of both hands). Furthermore parents referred that the child presented a previous episode of OCD when he was 12: the symptoms resolved with non-bearwighting and non-steroidal anti-inflammatory therapy.

The patient went under regular endocrinologist follow-up for short stature since he was 8. At the age of 10 (height cm 123 – SDS 2.4) Growth Hormone (GH) stimulation tests were performed showing only partial response to insulin tolerance test (GH peak 6.27 ng/mL). Bone age was delayed of 12 months. Human recombinant GH replacement therapy was started without a significant growth-velocity improvement.

**Results:** Although our patient came to observation because of suspected elbow septic arthritis, we re-considered the diagnosis: namely, i. recurrent episodes of osteochondritis dissecans; ii.short stature poorly responsive to human recombinant GH treatment, iii. Mild facial, skeletal dysmorphisms led us to hypothesize a form of aggrecanopathy. Molecular analysis of the *ACAN* gene was performed revealing a novel missense variant c.6970T>C, p.Trp2324Arg. A similar mutation in the G3 domain (c.7249G>A) has been previously described and has been connected to aggrecanopathy.

**Conclusion:** Aggrecan, an important sub-unit of proteoglycan in cartilage, is synthetized by *ACAN* gene. Aggrecan-related bone diseases range from severe spondylo-epi-metaphyseal dysplasia to familial osteochondritis dissecans usually associated with short stature. In our experience, a patient with a novel mutation of *ACAN* gene seems to cause an atypical form of aggrecanopathy mimicking inflammatory and/or septic arthritis associated with slight short stature and bone dysmorphisms. Intra-familial molecular analysis allowed us to recognize other three subjects (mother and 2 siblings) affected only by brachydactily. Further studies need to confirm the role of this variant of *ACAN* gene as cause of inflammatory arthropathy.

**Consent for publication has been obtained from patient**

Yes

**Disclosure of Interest**

None Declared

#### P1125 LRBA deficiency in a patient with chronic arthritis

##### Şerife Gül Karadağ, Ayşe Tanatar, Mustafa Çakan, Haifize Emine Sönmez, Figen Çakmak, NurayAktay Ayaz

###### Pediatric Rheumatology, Health Sciences University Kanuni Sultan Süleyman Training and Research Hospital, Istanbul, Turkey

####### **Correspondence:** Şerife Gül Karadağ

**Introduction:** Lipopolysaccharide-responsive, beige-like anchor protein (LRBA) deficiency causes common variable immunodeficiency (CVID) disorders and autoimmunity. Patients with LRBA deficiency may present with a wide spectrum of clinical manifestations. Herein, we report a patient with LRBA deficiency associated chronic arthritis

**Results:** A 20-year-old male patient was admitted to our hospital with swelling in knees and ankles for two months. He was initially diagnosed with Evans syndrome when he was four years old. Subsequently, he was diagnosed with Crohn’s disease due to chronic diarrhea at 18-years old. He also suffered from recurrent infections since infancy. He also was followed by endocrinology department due to hypothyroidism. When he was 20-years old, he was referred to our outpatient clinic due to chronic arthritis. In laboratory work-up, he had elevated acute phase reactants and direct coombs positivity. Romatoid factor, anti-nuclear antibody and HLA-B27 were negative. Initially, he was considered to have chronic arthritis due to Crohn’s disease and sulfasalazine was started. In third months, adalimumab was administered due to the inadequate response. Immunodeficiency due to coexistence of endocrinopathy, autoimmune cytopenia and recurrent infections was suspected. Therefore, expression of LRBA protein was found to be low. Mutation in LRBA gene was evaluated with Sanger sequence. A homozygous mutation in LRBA gene was detected (c.2818dupC;p.Gln940Profs*5).Abatacept (CTLA4 fusion protein) and intravenous immunoglobulin was started. Now, he is in remission.

**Conclusion:** The association of LRBA deficiency and autoimmunity has previously described. However, the data about the association LRBA deficiency and arthritis is limited. The new finding of this rare disease might further expand the phenotypic spectrum.

**Consent for publication has been obtained from patient**

Yes

**Disclosure of Interest**

None Declared

#### P1126 Unusual case of recurrent periodic macrophage activation

##### Khulood Khawaja^1^, Tawfiq T. Hen^2^

###### ^1^Adult and Pediatric Rheumatology, Al-Mafraq Hospital; ^2^Pediatrics, Shaikh Khalifah Medical City, Abu-Dhabi, United Arab Emirates

####### **Correspondence:** Khulood Khawaja

**Introduction:** Macrophage activation syndrome (MAS) is a multisystem inflammatory disorder, can be fatal. Most commonly complicating systemic juvenile idiopathic arthritis (JIA)

**Objectives:** To discuss treatment pathway of recurrent MAS in systemic JIA when initial therapy fails.

**Methods:** Case presentation of severe and recurrent periodic MAS in a child with systemic JIA

**Results:** 12-year-old girl presented with 3 weeks history of fever, lethargy and rash.She was having fever in the evening with erythematous rash over her shoulders, face and trunk.She then developed pain in multiple joints with swelling in both knee joints.She had ultrasound scan of her knees, which confirmed effusions and synovial thickening.There had been no preceding infections or travel. Parents are consanguineous.3 siblings fit and well. No family history of significance.

Initial examination, temperature 38.3°C, she had acanthosis nigricans in axillary areas and behind her neck.No dysmorphic features.Conjunctivanormal.No hair or nail abnormality.She had faint rash over her face and shoulders.Musculoskeletal examination, pediatric gait, arm, leg and spine examination:She was struggling to hop and walk on her heels.Arms:She hadminimal effusions in her wrists with pain on extreme flexion and extension.Legs:She had effusion in both knees and ankles.Spine:Normal.TMJ:Normal. Examination of other systems unremarkable (later developed mild hepatospleenomegaly)

Initial bloods:ANA negative, double stranded DNA negative, C3 and C4 negative, ENA negative, rheumatoid factor positive.Sugar okay, thyroid function okay, hepatitis B and C serology negative, Quantiferonnegative, Renal function normal. ESR 115 and CRP 129.

USS of knees, ankles and wrists confirmed synovitis.CHAQ score 0.5, pain VAS 6, general VAS 7,

Initially treated as JIA with systemic features and was started on Methotrexate 15mg/m2 weekly, joint injections with steroids of wrists, knees and ankles then oral prednisolone reducing dose. She improved. Rash resolved. No fever but continued to complain of joint pains. She was changed to Anti TNF; etanercept (50mg weekly) and small dose of steroids . She also had inadequate response so was changed to IL-6 blocker; tocilizumab.She was well for 6 months then developed MAS presenting with fever, lethargy and feeling nauseated with raisedferritin of >10,000, cytopenia, hyperglyceremia, hypofibrogenemia and raised triglycerides. Bone marrow aspirate normal. Treatment changed to IL-1 blocker Anakinra 100mg daily. She was well on anakinra for 4 months then presented again with MAS requiring IV methyl prednisolone and IV fluids. Has had monthly attacks for 5 months (one with seizure) then every 2 weeks in the last 2 months. Cyclosporin 100mg twice daily was added to her treatment with no benefit. She also had IVIG 2g/kg with no benefit. She was assessed by Oncology and neurology; no concern of malignancy. HLH genetic testing: PRF1, UNC13D, STX11, STXBP2.,SH2D1A, XIAP (BIRC-4), RAB27A, LYST, ITK, GATA2, AP3B1, BLOC1S6, CD27, SLC7A7 all negative. Brain imaging normal. TRAPS,NLRP3 genetic testing neative. She is currently awaiting the result of whole gene sequencing. In terms of treatment, cyclophosphamide was started 375mg/m2. Had first dose. Planning3 doses every 2 weeks then 4 doses every month.

**Conclusion:** Our patient had multiplepreseentations of MAS, first following several months of treatment with tocilizumab then Anakinrathen 5 presentations monthly only responding to systemic steroids then every 2 weeks for the last 4 presentations.

No clear guidelines to treat MAS once standard therapy unsuccessful. Further work is needed to study the genetics of such patients by collaborative work amongst multiple centres.

**Consent for publication has been obtained from patient**

Yes

**Disclosure of Interest**

None Declared

#### P1127 AA amyloidosis in recipients of cadaveric derived pituitary growth hormone – potential effect of an amyloid enhancing factor?

##### Helen J. Lachmann, Dorota Rowczenio, Janet A. Gilbertson, Hadija Trojer, Islam Noor, Thirusha Lane, Julian D. Gillmore, Ashutosh D. Wechalekar, Philip N. Hawkins

###### National Amyloidosis Centre, UCL Division of Medicine and Royal Free Hospital NHS Foundation Trust, London, United Kingdom

####### **Correspondence:** Helen J. Lachmann

**Introduction:** The development of Creutzfeldt–Jakob disease (CJD) in individuals who had been treated during childhood with human cadaveric pituitary-derived growth hormone (c-hGH) was first reported in 1985 and resulted in its immediate withdrawal and replacement with recombinant protein. Subsequently amyloid-β protein (Aβ) deposits have been described in individuals with CJD and prior exposure to c-hGH. Recently misfolded Aβ peptide was identified in archived vials of human c-hGH, which induced Aβ pathology in an experimental model (Purro et a Nature 2018) supporting its role in the transmission of CJD.These observations are reminiscent of the concept of amyloid enhancing factor (AEF), in experimentally induced AA amyloidosis, in which administration of tiny amounts ofamyloidotic material is associated with massively accelerated AA amyloid deposition.

**Objectives:** To look for evidence of prior exposure to c-hGH in patients with AA amyloidosis.

**Methods:** We searched our database of 625 patients with AA amyloidosis seen between 1990 and 2017 using the key words pituitary, craniopharyngioma and growth hormone. Only patients whose records suggested exposure to GH prior to 1985 were included.

**Results:** 10 cases were identified; accounting for 1.6% of all AA amyloidosis and 8.4% of cases of AA of unknown aetiology. 8 were female, 8 were north European ethnicity and the median age at histological diagnosis of AA amyloidosis was 47.9 yrs with a median time since commencing c-hGH of 35 yrs. c-hGH was started in the 1960’s in 4 cases, the 1970’s in 4 cases and the 1980’s in 2 cases. The indications for GH were: craniopharyngioma in 4, one case each of resection of ependymona, nasal pharyngeal tumour and encephalocoele, 3 cases of unexplained hypopituatism. Chronic inflammation was assessed by serial blood tests after the diagnosis of AA amyloid; only 1 case had severe sustained inflammation with a median SAA 131 mg/L; 4 had medians SAAs of between 20 and 54 mg/L and the others had only low level inflammation with median SAA below 20mg/L. No clear cause of inflammation was identified in the cohort although 50% were obese and 3 had body mass index above 45 kg/m^2^. Genetic testing was performed in all – in no cases did this result in a diagnosis of a systemic autoinflammatory disorder. Six European patients were homozygous for SAA1.1 (66.7% compared to a reported frequency of 42.7 - 57.9%in European controls), a known predisposing factor for AA amyloidosis. One patient had a fall in SAA after bariatric surgery and one responded completely to anakinra. The other patients had no specific treatment aimed at supressing their SAA production. Five patients eventually required dialysis – 2 of them had subsequent renal transplants; median survival from diagnosis with amyloid is 132 months and 3 patients have died.

**Conclusion:** We report 10 patients with AA amyloidosis of unknown aetiology, who were exposed to c-hGH in childhood. We hypothesise that the c-hGH may have contained material that seeded conversion of healthy low concentrations of SAA into AA amyloid fibrils. It is of interest that 6 of this cohort had an additional risk factor in the form of SAA1.1 homozygosity suggesting that a combination of factors may contribute to amyloid formation in individuals who have low levels of circulating potentially amyloidogenic protein.

**Disclosure of Interest**

None Declared

#### P1128 Progressive necrotic ulcerative lesions: challenging diagnosig?

##### Angela Mauro^1^, Francesca Orlando^2^, Maria Tardi^3^, RobertoRega^3^, Martemucci Luigi^3^, Rita Sottile^3^

###### ^1^Pediatrics, Rheumatology Unit, Department of Pediatrics, “Santobono-Pausilipon” Children Hospital, Naples, Italy; ^2^ Pediatrics; ^3^Pediatrics, Rheumatology Unit, Department of Pediatrics, “Santobono-Pausilipon” Children Hospital, Naples, Naples, Italy

####### **Correspondence:** Angela Mauro

**Introduction:** Pediatric ulcers are less common compared to adult population. No data about prevalence are available. Differential diagnosis is broad, including autoinflammatory, hematologic, infectious andautoimmune causes. .

**Objectives:** We describe a case of progressive necrotic ulcerative lesions in a 7- month caucasian boy

**Methods:** A 7-month-old Caucasian boy was admitted to our Department with purpuric and telangiectatic lesions on his right hand and left wrist without fever. After one month these lesions evolved in necrotic ulceration of the skin .He was born at term to unrelated parents after a pregnancy complicated by premature contractions.At the age of 9 months he developed a large necrotic lesion (4x4 cm) on the right foot and other similar smaller lesions on the knees. He had purpuric and telangiectatic lesions on the left foot and the medial face of the left leg. Laboratory assessments were performed to exclude infectious diseases (Mycoplasma, Chlamydia, HBV, HCV, HAV, Treponema Pallidum, Rickettisa conorii, Widal-Wright, BorrelliaBurgdoferi, TORCH, EBV) The inflammatory markers (ERS, SAA, CRP), prothrombotic risk factors (PT, APTT, fibrinogen, protein S, protein C), C3, C4, immunoglobulins, antiphospholipid antibodies, ANA, ANCA, ASCA, anti-Dna, celiac disease antibodies, Quantiferon, hemoglobin electrophoresis, blood culturesand urine analysis were negative or in the normal range.The analysis of CERC1 gene was negative for the main mutaions.It was also tested CD18, blood T-lymphocyte subpopulations. cryoglobulins, interferon signature (negative) and CGH ARRAY, karyotype, and for zinc deficiency (ongoing).Skin lesion cultures were positive forProteus mirabilis andStaphylococcus aureus.Ultrasonography of lower limbs resultednegative except for arteritis of the small vessels on the right foot lesion.

Echocardiography showed interatrial communications ostium secundum type with slight left-right shunt.Abdomen Ultrasoundwas negativeChest radiography (fatta in faseacuta?):hyperinflated lungs, aincrease interstitial markingof the right and left retrocardiacfields. . Flat diaphragm. Expansive ribs.Brain MRI: negativeElectromyography: slight neurogenic problemsSkin biopsy: microscopic epidermis partly necrobiotic, focal spot parakeratosis and spongiosis with vesicles formation. In the dermis, presence of edema and erythrocyte extravasation in the papillae, small thrombosed vessels, sometimes with fibrinoid necrosis and only focal lymphocytic infiltrated, PASS-Giemsa negative staining, negative Z-Nilsen staining

**Results:** Findings consistent with livedoid vasculopathy, cryoglobulinemia and gangrenous ecthyma. For gangrenous ecthymahe started Teicoplanin, Meropenem and Fluconazole with a subsequent improvement of the skin lesions and a decrease of necrotic eschars and lesion re-epithelialization, but after 15 days he presented new purpuric lesions on his legs, right heel, and hands.

**Conclusion:** Do you have any input about this patient?

**Consent for publication has been obtained from patient**

Yes

**Disclosure of Interest**

None Declared

#### P1129 An undefined systemic autoinflammatory disease complicated by MAS or an unidentified primary HLH?

##### Roberta Naddei^1^, Francesca Orlando^1,2^, Carolina Porfito^1^, Teresa Lastella^1^, Marinabiondina Amico^1^, Maria Alessio^1^

###### ^1^Pediatric Rheumatology Unit, Mother and Child Department, University of Naples Federico II; ^2^Department of Pediatrics, Santobono-Pausilipon Children’s Hospital, Naples, Italy

####### **Correspondence:** Roberta Naddei

**Introduction:** Systemic auto-inflammatory diseases (SAIDs) are a rare group of illnesses characterized by unprovoked episodes of fever and systemic inflammation. Many patients receive a diagnosis of undefined SAID because they have clinical features not fitting a specific diagnosis or genetic tests are negative.

**Objectives:** To describe clinical presentation of a patient affected by recurrent episodes of fever characteristics resembling hemophagocytic lymphohistiocytosis.

**Methods:** Case report.

**Results:** The patient (male, age 30 months) referred to our Unit at the age of 3 months. At 1 month, he underwent a surgical repair of ventricular septal defect. At 3 months, he was admitted to a hospital for fever and maculopapular rash, with marked elevation of acute phase reactants (Protein C-reactive, PCR, 108 mg/L). Empiric antibiotic therapy was started, but no evidence of infectious diseases was disclosed.At the tenth day of fever, laboratory examinations found thrombocytosis and increase of ferritin (5034 ng/ml), lactate dehydrogenase (1059 U/l), aspartate and alanine aminotransferase (254 and 124 U/l) and triglycerides (266 mg/dl). In the suspicion of atypical Kawasaki disease, even if no coronary artery aneurysm was detected, treatment with intravenous Immunoglobulin and aspirin was started with disappearance of fever; because of persistent cutaneous rash and ferritin, triglycerides, platelets and PCR high levels, he was transferred to our Unit. On admission, physical examination revealed neck and inguinal enlarged lymph nodes. No organomegaly was detected. Laboratory investigations also revealed increased neutrophils (17830/mm^3^), normocytic anemia (hemoglobin 7,5 g/dl), hypoalbuminemia and hyponatremia. Fibrinogen levels were normal. Bone marrow aspiration revealed no signs of hemophagocytosis or cellular atypia. Intravenous methylprednisolone pulse therapy (30 mg/kg for three days) was started followed by daily oral prednisone (2 mg/kg/day), with clinical resolution and laboratory improvement. After six-months tapering, steroid therapy was withdrawn. Two months later, the patient newly present fever, rash and increase of PCR and ferritin. Anakinra (2 mg/kg/day) was started with improvement of clinical condition. Nevertheless, because of the persistent elevation of inflammatory markers, anakinra dosage was increased up to 4 mg/kg/day during the follow-up. Besides, the mother of child revealed difficulty to adhere daily subcutaneous therapy. At the age of 2 years, he presented a new episode of fever and rash with high ferritin level (2364 ng/ml); anakinra was stopped and canakinumab (4 mg/kg/4 weeks) was started with initial dramatic clinical and laboratory improvement. Fifteen days after the first injection of canakinumab, the patient newly presented fever, without rash. IL18 was raised (184000 pg/ml). Therapy with prednisone (2 mg/kg/die) was added with fever disappearance. During the follow-up, in therapy with canakinumab and prednisone, the patient never obtained normalization of inflammatory markers and fever presented two or three weeks after canakinumab injection.

So, during the last episode of fever, canakinumab was stopped and cyclosporine was added to steroid therapy. Since the introduction of cyclosporine, the patient has not presented fever and inflammatory markers have normalized.

During the follow-up, the patient underwent Next Generation Sequencing for HLH and SAID; no mutation was identified, except for a heterozygous CECR1 variant.

**Conclusion:** This patient presents recurrent hyperinflammation episodes, resembling HLH/MAS, with low response to anti-IL1 agents. Genetic tests for HLH and SAID are negative; what are we missing? Should we attempt IL18 blockade?

**Consent for publication has been obtained from patient**

Yes

**Disclosure of Interest**

None Declared

### Allied Health Professionals

#### P1130 Towards European harmonisation of healt care for patients with rare immune disorders: ERN RITA result from the registries survey

##### Riccardo Papa^1^, Andrew Cant^2^, Christoph Klein^3^, Mark Little^4^, Nico M. Wulffraat^5^, Marco Gattorno^1^, Nicolino Ruperto^1^ and on behalf of RITA ERN Council

###### ^1^Clinica Pediatrica e Reumatologia, IRCCS Istituto Giannina Gaslini, Genova, Italy; ^2^Great North Children's Hospital, & Institute for Cellular Medicine, University of Newcastle, Newcastle upon Tyne, United Kingdom; ^3^Department of Pediatrics, Ludwig-Maximilians-University Munich, Munich, Germany; ^4^Department of Nephrology and Kidney Transplantation, Beaumont Hospital, Royal College of Surgeons in Ireland, Dublin, Ireland; ^5^Department of Pediatrics, Section Pediatric Rheumatology, Wilhelmina Children's Hospital, University Medical Centrum Utrecht, Utrecht, Netherlands

####### **Correspondence:** Riccardo Papa

**Introduction:** Rare Immunodeficiency, AutoInflammatory and AutoImmune Disease (RITA) network is a European Research Network that brings together the leading centres for rare immune disorders (RID).

**Objectives:** To know the state of the art about diseases registries and research networks in Europe regarding patients with RID.

**Methods:** On April 2018 an on-line survey was sent to all 126 RITA members, of whom 45 are healthcare providers and 8 patients/family organizations. All data were collected and analysed using Excel program.

**Results:** Ninety RITA members (71%) replied. Collectively, 27 members are coordinating 25 registries, 53 members are participating in 38 registries, and 27 members knew the existence of 16 registries without participating. Only two members are not involved in any registries.

Data about 53 different registries were collected across 14 European countries. Almost 50% of registries collect data on autoimmune disorders, while others are dedicated to primary immunodeficiencies and autoinflammatory diseases (respectively 15 registries, 28%, and 12 registries, 23%). Fifteen registries (28%) enrolled patients with a single specific disorder: in particular, three registries are devoted to systemic lupus erythematosus, two registries to Kawasaki disease or Behcet disease, and single registry to juvenile dermatomyositis, juvenile systemic sclerosis, juvenile idiopathic arthritis (JIA)-related uveitis, systemic JIA, Blau syndrome, sarcoidosis, Guillain-Barre syndrome, and myasthenia gravis.

More than 55000 patients with RID are enrolled. The majority of registries (36; 68%) enrol only patients from national territories. Among the internationals, six collect data on autoimmune disorders (Pharmachild, BrainWorks, EuroMyositis, EULAR web library, UKIVAS registry and JIR cohort), five on primary immunodeficiencies (ESID, EBMT, SCETIDE, PCID and HLH registry), and three are devoted to autoinflammatory diseases (Eurofever, Infevers, and ImmunAID), despite also ESID registry and JIR cohort collect data on autoinflammatory diseases.

Data usually collected in these registries are demography, diagnosis, clinical manifestations, laboratory tests and treatment, while genetic and imaging data are less frequently reported (respectively in 38% and 9% of registries). A treatment safety profile is reported in 29 registries (55%). Collectively, fifteen biobanks are counted.

**Conclusion:** The survey highlighted the pivotal role of national and international organizations in Europe to collect and organize clinical data on immune diseases, allowing the rapidly growing knowledge on these rare disorders, creating research networks and providing significant numbers of data to support new discoveries in the field. RITA network could improve the coordination of these numerous entities, supporting initiatives of collaboration. As a first attempt, the present survey revealed that the collection of key parameters about patient safety, as well as outcome data and quality of life measures should be improved among the registries of RITA network.

**Disclosure of Interest**

None Declared

## Poster presentations – Tuesday 2 April

### Guided poster tour 2A

#### PT2A01 The French pediatric cohort of castleman disease

##### Charlotte Borocco^1,2^, Claire Ballot-Schmit^3^, Oanez Ackermann^4^, Nathalie Aladjidi^5^, Jeremie Delaleu^6^, Vannina Giacobbi-Milet^7^, Sarah Jannier^8^, Eric Jeziorski^9^, Yves Perel^5^, François Maurier^10^, Christophe Piguet^11^, Eric Oksenhendler^12,13^, Isabelle Kone-Paut^1,2,14^, Caroline Galeotti^1,2,14^

###### ^1^Pediatric Rheumatology; ^2^CEREMAIA, Bicetre Hospital, Le Kremlin-Bicêtre; ^3^General Pediatrics, Jean Minjoz Hospital, Besançon; ^4^Pediatric hepatology, Bicetre Hospital, Le Kremlin-Bicêtre; ^5^Pediatric Oncology and Hematology, Pellegrin Hospital, Bordeaux; ^6^Internal Medicine, Tenon Hospital, Paris; ^7^Pediatric Oncology and Hematology, Le Mans Hospital, Le Mans; ^8^Pediatric Oncology and Hematology, Hautepierre Hospital, Strasbourg; ^9^General Pediatrics, Arnaud de Villeneuve Hospital, Montpellier; ^10^Internal Medicine, Metz Private Hospital, Metz; ^11^Pediatric Oncology and Hematology, Limoges Hospital, Limoges; ^12^Clinical Immunology, Saint-Louis Hospital; ^13^National Reference Center for Castleman Disease, Paris; ^14^National Reference Center for Castleman Disease, Le Kremlin-Bicêtre, France

####### **Correspondence:** Caroline Galeotti

**Introduction:** Castleman disease (CD) is a very rare non-malignant lymphoproliferation of undetermined origin. CD diagnosis is difficult and often delayed because of insidious onset, low awareness and clinical heterogeneity. Two major disease phenotypes can be distinguished: unicentric CD (UCD) and multicentric CD (MCD). Diagnosis confirmation is based on histopathological findings on an involved lymph node.

**Objectives:** We attempted to survey all cases of paediatric CD identified in France so far, in order to set up a national registry aimed to improve CD early recognition, treatment and follow-up, within the context of a new reference centre (http://www.castleman.fr).

**Methods:** In 2016, we e-mailed a questionnaire to members of the French paediatric immunohaematology society, the French paediatric rheumatology society and the French Reference Centre for Castleman Disease to retrospectively collect cases of paediatric CD (first symptoms before age 18 years). Anatomopathological confirmation was mandatory.

**Results:** We identified 23 patients (12 girls and 11 boys) diagnosed with UCD (n=17) and MCD (n=6) between 1994 and 2018 in 14 centres.

The average age at first symptoms was 11.5 years for UCD and 8.3 years for MCD. The mean diagnosis delay was 8.2 months for UCD and 5.2 years for MCD.

In UCD, the initial symptoms were: isolated lymph nodes (n = 10) or lymph node associated with other symptoms (n = 7), fever was present in 3 patients.

Patients with MCD presented with fever (n=5), abdominal lymph nodes (n=5), failure to thrive (n=3), hepatomegaly and/or splenomegaly (n=3), arthralgia (n=2), abdominal pain (n=2), fatigue (n=2), facial oedema (n=1), isolated lymphadenopathy (n=1), trunk rash (n=1), vascular hepatopathy with oesophageal varicose veins (n=1), diarrhoea (n=1) or cholestasis (n=1). One patient had a Duchenne muscular dystrophy. No patients had HIV or HHV8 infection. The 6 patients met all the diagnosis criteria proposed by Fajgenbaum et al in 2017 for idiopathic MCD.

One MCD patient with recurrent fevers, had a heterozygous mutation in *MEFV* gene. Another UCD patient experienced fever episodes and pericarditis two years after surgery. Genetic tests revealed one heterozygous mutation in *IL10RA* gene (V406L) and one in *IL36RN* gene (S113L). Nevertheless, our patient did not have any psoriasis or inflammatory bowel disease.

Treatment of UCD was mainly surgical resection, steroids, and radiotherapy. Treatments of MCD were tocilizumab, rituximab, anakinra, steroids, chemotherapy and splenectomy.

Overall survival after an average of 6 years of follow-up, was 100 % for both unicentric and multicentric forms.

**Conclusion:** Paediatric CD seems still underdiagnosed with a significant diagnosis delay specially for the MCD but new international criteria will help in future. In UCD, surgery is the main treatment. New drugs are used specially for the MCD, but more studies need to be conduct in children. The global survey is better in children than adults.

**Disclosure of Interest**

None Declared

#### PT2A02 Use of whole-body magnetic resonance to identify potential diagnostic clues in children with fever of unknown origin (FUO)

##### Sara Signa^1,2^, Roberta Caorsi^1^, Giorgio Stagnaro^3^, Francesca Minoia^1^, Paolo Picco^1^, Angelo Ravelli^1,2^, GM Magnano^3^, Maria B. Damasio^3^, Marco Gattorno^1^

###### ^1^Clinics of Pediatrics and Rheumatology, G.Gaslini Institute; ^2^DINOGMI, University of Genoa; ^3^UO of Radiology, G.Gaslini Institute, Genoa, Italy

####### **Correspondence:** Sara Signa

**Introduction:** Whole-body magnetic resonance imaging (WBMRI) is a fast and accurate method to detect diseases throughout the entire body without exposure to ionizing radiation. Possible emerging applications for this technique include rheumatologic field and evaluation of fever of unknown origin (FUO).

**Objectives:** To evaluate the ability of WBMRI to identify significant potential diagnostic clue (PDC) in patients presenting a non specific inflammatory clinical picture.

**Methods:** We retrospectively collected cases of pediatric patients followed in a single pediatric rheumatology center who underwent WBMRI between January 2010 and December 2015 for the following indications: i) FUO (temperature greater than 38.3°C for more than three weeks or failure to reach diagnosis after one week of investigations), iii) recurrent fever (febrile episodesseparated by periods of normal temperature), iii) Inflammation of unknown origin, IUO (an illness of at least 3 weeks’ duration, with raised inflammatory markers and fever below 38.3°C).WBMRI studies were acquired with coronal and sagittal planes (slice thickness 5mm) with acquisition of several image sets with automatic direct image realignment after acquisition creating a whole-body scan.Sequences include short τ inversion recovery (STIR) and T1-weighted. All studies have been evaluated twice, the second time according to a predefined checklist, defined by an experienced radiologist, considering systematically single /multifocal bone lesion, bone marrow, joint effusion, soft tissues, adenopathies, parenchymal and vessels looking for PDC. We considered as a Potential Diagnostic Clue each alteration of the examined district that can potentially guide the diagnosis. Each alteration found is a PDC. We retrospectively evaluated patients’ clinical history and final diagnosis and we classified the PDCs identified during both first evaluation and re-evaluation as: Not useful (the identified PDC did not guide the diagnosis and is not coherent with the final diagnosis), consistent (the identified PDC is congruent with the patient’s final diagnosis) or diagnostic (the identification of the considered PDC strongly orient the final diagnosis).

**Results:** We collected 102 patients who underwent WBMRI; 24 (23%) of them presenting FUO, 27 (26%) presenting recurrent fever and 51 (51%) presenting persistent low grade fever . The mean age of onset symptoms was 7 years and seven months (range: 2 weeks old- 17 years and 6 months). The mean age of execution of WBMRI was 9 years (range: 5 months old- 19 years and one month). After the whole diagnostic work-out a final diagnosis was achieved in 42 patients (41%).PDCs were identified at the first evaluation in 76/102 cases (74.5%).In 22 cases (21.5%) the identified PDCs were consistent with the diagnosis, whereas in 7 cases (7%) the identified PDCs were considered diagnostic. Globally we can consider that at first evaluation PDCs were somehow contributory to the diagnosis in 29 cases (28.5%; 6 JIA, 7 systemic infections, 5 monogenic inflammatory diseases, 4 ALPS, 2 Goldbloom’s Syndrome,2 Vasculitis,1 eosinophilic fasciitis, 1 hystiocytosis, 1 neuroblastoma). Blind re-evaluation of WBMRI allowed the identification of additional PDCs in 52 patients (12 of them previously negative) . In 10 cases the PDC found after re-evaluation were consistent with the final diagnosis (2 JIA, one infectious disease, one neuroblastoma, 3 ALPS, 1 monogenic inflammatory disease, 1Takayasu arteritis, 1 Goldbloom’s syndrome).

**Conclusion:** WBMRI can be a powerful diagnostic tool in patients with FUO. A predefined checklist increase sensitivity of WBMRI in the identification of PDC.

**Disclosure of Interest**

None Declared

#### PT2A03 A biomarker profile including S100A12 as diagnostic tool for the differential diagnosis of sJIA-associated MAS vs. primary or secondary HLH

##### Christoph Kessel^1^, Ndate Fall^2^, Alexei Grom^2^, Wilco de Jager^3^, Sebastiaan Vastert^4^, Raffaele Strippoli^5^, Claudia Bracaglia^6^, Erik Sundberg^7^, Anna Horne^8^, Stephan Ehl^9^, Sandra Ammann^9^, Kai Lehmberg^10^, Fabrizio De Benedetti^6^, Helmut Wittkowski^1^, Katharina Kessel^1^, KarinBeutel^11^, Dirk Foell^1^, Dirk Holzinger^12^

###### ^1^Pediatric Rheumatology and Immunology, University Children’s Hospital, Muenster, Germany; ^2^Division of Rheumatology, Cincinnati Children’s Hospital Medical Center, Cincinnati, United States; ^3^Laboratory of Translational Immunolgy, Utrecht Medical Center; ^4^Pediatric Rheumatology and Immunology, University Medical Center Utrecht, Utrecht, Netherlands; ^5^Cellular Biotechnology and Hematology, Sapienza University of Rome; ^6^UO Reumatologia, IRCCS Ospedale Pediatrico Bambino Gesù, Rome, Italy; ^7^Paediatric Rheumatology Unit; ^8^Childhood Cancer Research Unit, Karolinska University Hospital Solna, Stockholm, Sweden; ^9^Center for Chronic Immunodeficiency, University Hospital Freiburg, Freiburg; ^10^Pediatric Hematology and Oncology, University Medical Center Hamburg Eppendorf, Hamburg; ^11^Pediatric Hematology and Oncology, Children’s Hospital, TU Munich, Munich; ^12^Pediatric Hematology-Oncology, University of Duisburg-Essen, Essen, Germany

####### **Correspondence:** Dirk Holzinger

**Introduction:** Macrophage activation syndrome (MAS) is a severe complication of autoimmune and autoinflammatory disease and is strongly associated with systemic juvenile idiopathic arthritis (sJIA).

**Objectives:** While the granulocytic protein S100A12 is highly overexpressed in sJIA, and the assessment of S100A12 serum levels helps to distinguish sJIA from other febrile illnesses, the presence of elevated levels of IFNγ, IFNγ-induced chemokines and IL-18 have recently been suggested to play a pivotal role during particularly MAS episodes. Clinically, however, MAS is strikingly similar to hemophagocytic lymphohistiocytosis (HLH) and the initial differentiation between sJIA-associated MAS and primary or secondary HLH can be challenging. In this study we set out to define a serum marker profile to support the initial diagnosis of sJIA-MAS and differentiate this from primary or secondary HLH.

**Methods:** A multiplexed bead array panel for simultaneous detection of 53 cytokines and chemokines was built based upon their suggested relevance in MAS or HLH according to respective literature. Serum samples from patients with active primary HLH (pHLH) (n=10), secondary HLH (sHLH) (n=12), clinically active or inactive sJIA-MAS (both n=11) as well as healthy controls (n=10) were subjected to this bead array assay as well as quantification of S100A12 levels by ELISA. Based on the data obtained from this discovery cohort we extracted a subset of analytes, which was validated using commercially available bead array reagents in an independent cohort.

**Results:** Following analysis of the discovery cohort, out of 54 serum analytes 14 markers were identified that significantly distinguished sJIA-MAS from HLH. These mostly distinguished between pHLH and MAS, while S100A12, IL-18 as well as the MIG/IL-18-ratio also separated between MAS and sHLH. In ROC-analyses six out of these 14 analytes separated MAS from HLH with AUC > 0.9. S100A12 (AUC=0.99), sFASL (0.98) and IL-18 (0.97) were the top-three performing markers in these analyses. The 14plex panel including S100A12 was validated in an independent cohort resulting in IL-18 (AUC=0.83), S100A12 (0.75) and sFASL (0.7) as the top-three performing markers in separating sJIA-MAS from HLH.

**Conclusion:** IL-18, S100A12 and sFASL serum levels are useful to differentiate between sJIA-associated MAS and HLH. This can be a helpful diagnostic tool to discriminate sJIA-associated MAS during early onset of the disease from primary or secondary HLH.

**Disclosure of Interest**

None Declared

#### PT2A04 Early treatment with anakinra in systemic juvenile idiopathic arthritis

##### Manuela Pardeo, Claudia Bracaglia, Anna Tulone, Antonella Insalaco, Giulia Marucci, Rebecca Nicolai, Virginia Messia, Emanuela Sacco, Fabrizio De Benedetti

###### Division of Rheumatology, IRCCS Ospedale Pediatrico Bambino Gesù, Rome, Italy

####### **Correspondence:** Manuela Pardeo

**Introduction:** Systemic juvenile idiopathic arthritis (sJIA) accounts for 10-20% of all patients with JIA. The prominent systemic clinical features, the marked elevation of inflammatory markers and the absence of autoantibodies make this disease different from other JIA forms. sJIA should be considered as a polygenic autoinflammatory disease. Interleukin 1 (IL-1) has been shown to be a major mediator of the inflammatory cascade that underlies sJIA. Treatment with anakinra has been reported to be effective in a sizable portion of patients with sJIA.

**Objectives:** To assess clinical response rate and disease course in sJIA patients treated with anakinra.To evaluated whether the response to anakinra was related to baseline variables.

**Methods:** We reviewed 56 (28 F) consecutive patients with sJIA treated with anakinra for at least 6 months in our institution. The diagnosis of sJIA was established according to the International League of Associations for Rheumatology (ILAR) classification criteria. We analyzed the effect of anakinra on fever, rash, number of active joints, erythrocyte sedimentation rate (ESR), C-reactive protein (CRP), white blood cells count, platelets count and ferritin levels. Clinically inactive disease (CID) was defined according to Wallace criteria. Clinical and laboratory data were obtained using a standard data collection form.

**Results:** The median age at the disease onset was 5.7 (IQR 2.9-10.2) years. The median time from onset to received anakinra was 1.9 (IQR 0.7-9.7) months. At baseline 52/56 (93%) of patients had fever and median number of active joints was 2 (IQR 1-4). After 6 months of treatment 39 patients (69.6%) met criteria for inactive disease. Among 56 patients 17 (30.3%) received anakinra in monotherapy and 39 (69.6%) received anakinra with glucocorticoids. There were no statistically significant differences between the two groups for demographic, clinical and laboratory features*.*13/17 (76.4%) patients treated with anakinra alone and 26/39 (66.6%) patients treated with anakinra and glucocorticoids met criteria for CID off glucocorticoids at 6 months (p=0.54). Among the 56 patients, 29 (51.7%) received anakinra within 2 months from disease onset. There were no statistically significant differences for demographic, clinical and laboratory features among patients who started anakinra in the first 2 months from disease onset compared to those that started anakinra after 2 months. At 6 months after beginning of anakinra treatment, 27/29 patients (93.1%) who started anakinra within 2 months from disease onset and 12/27 (44.4%) who started anakinra after 2 months from disease onset reached clinical inactive disease off glucocorticoids (p=0.0001). Patients who started anakinra after the first 2 months from disease onset have a significantly higher risk of non-response (OR=8.06, 95% CI: 2.03-32.0).

**Conclusion:** According with several observations, anakinra is effective in a significant proportion of patients with sJIA. A possible approach to introduce IL-1 inhibitor, with or without concomitant glucocorticoids, early in the disease course taking advantage of a “window of opportunity” has been suggested. Our observation confirms that earlier treatment with anakinra is associated with a better short-term outcome. Moreover, our results show that beginning of treatment after two months from disease onset is correlated with a high risk of non-response.

**Disclosure of Interest**

M. Pardeo: None Declared, C. Bracaglia: None Declared, A. Tulone: None Declared, A. Insalaco: None Declared, G. Marucci: None Declared, R. Nicolai: None Declared, V. Messia: None Declared, E. Sacco: None Declared, F. De Benedetti Grant / Research Support from: Novartis, Novimmune, Hoffmann- La Roche, SOBI, AbbVie, Pfizer

#### PT2A05 Antibodies to collagen V and K-Α 1 tubulin in a patient with systemic juvenile idiopathic arthritis associated lung disease

##### Elena Tronconi^1^, Thalachallour Mohanakumar^2^, Lara Danziger-Isakov^3^, Grant Schulert^1^, Alexei Grom^1^

###### ^1^Division of Rheumatology, Cincinnati Children’s Hospital Medical Center, Cincinnati; ^2^Norton Thoracic Institute Research Laboratory, St. Joseph’s Hospital and Medical Center, Phoenix; ^3^Division of Infectious Diseases, Department of Pediatrics, Cincinnati Children’s Hospital Medical Center, Cincinnati, United States

####### **Correspondence:** Elena Tronconi

**Introduction:** Lung transplantation is still characterized by high morbidity and mortality. Bronchiolitis obliterans syndrome (BOS), the clinical correlate of chronic rejection, highly impacts long-term outcomes. It has been shown that recipients can develop antibodies to self-antigens (sAgs) like collagen V (ColV) and K-α 1 tubulin (Kα1t), and this correlates with the development of BOS. ColV and Kα1t are expressed by small airway epithelial cells. The interstitial remodeling that occurs with transplantation seems to enhance ColV and Kα1t exposure. Systemic juvenile idiopathic arthritis (sJIA) is a form of juvenile arthritis characterized by various degree of arthritis and prominent systemic features. In few cases it can be associated with lung involvement, a life-threatening complication, whose exact pathogenetic mechanism is still poorly understood.

**Objectives:** We decided to explore the possible role of antibodies to lung sAgs in a single patient affected by sJIA complicated by several episodes of macrophage activation syndrome (MAS), interstitial lung disease and pulmonary alveolar proteinosis.

**Methods:** We retrospectively analyzed serum samples collected during clinical follow-up from August 2015 to February 2018. We considered positive values more than 218 ng/ml for Kα1t and 160 ng/ml for colV according to the study by Hachem *et al.* The results were matched with clinical symptoms and laboratory parameters as white blood cell counts (WBC), hemoglobin (Hb), platelets (PLT), C reactive protein (CRP) and ferritin. Furthermore, we reviewed the ongoing treatment at the time of sample collection.

**Results:** In our patient ColV antibodies fluctuated over time, 7/20 samples were positive. The correlation analysis between ColV and WBC, Hb, PLT, CRP and ferritin did not show statistical significance. However, the review of clinical charts showed that at the time of 6 out of the 7 positive samples there was fever, worsening of respiratory symptom like persistent cough, increase respiratory rate or exertional tachypnea, mild flare of sJIA or MAS. Twelve of the 13 negative samples were collected when the patient was in stable clinical conditions, only 1 sample was collected during an episode of early MAS. The trend of Kα1t antibodies did not match completely with ColV. Considering the previously reported normality range, we did not find any positive result. Therapy was a combination of steroid, cyclosporin, anakinra, canakinumab, interleukin 18-binding protein, intravenous immunoglobulins (IVIG). All the treatments, the dosage of steroid and cyclosporin as well as timing of IVIG were tailored on clinical conditions and laboratory test so it is difficult to define a clear association with ColV antibodies values.

**Conclusion:** The pathogenic mechanism of sJIA-associated lung diseases is still poorly understood. This preliminary retrospective study showed a good correlation between antibodies to ColV and clinical disease activity, in particular with MAS and worsening of respiratory symptoms. The lack of correlation with laboratory markers can be explain by the prompt modification of treatment according to the clinical condition of the patient and the retrospective nature of the study. Similar to what happens in BOS after transplantation, we hypothesize that during a flare of sJIA or an episode of MAS the inflammation and cellular damage expose sAgs, normally hidden, to the circulation, worsening and amplifying the immune response. This preliminary evidence of increased pulmonary sAgs in sJIA with lung involvement represents a possible paradigm shift in the pathophysiology of the disease although we need more evidence prospectively collected in a larger population.

**Disclosure of Interest**

None Declared

#### PT2A06 Recurrent fever, recurrent pyoderma gangrenosum-like and bacterial infections due to a novel homozygous WDR1 mutation in a child treated with allogeneic hematopoietic stem cell transplantation

##### Estibaliz Iglesias^1^, Monica Roldan^2^, Alexandru Vlagea^3^, Manuel Solís-Moruno^4^, Juan Manuel Mosquera^1^, Violeta Bitterman^1^, Joan Calzada-Hernández^1^, Laia Alsina^5^, Angela Deyà-Martinez^5^, Rocío Lara^3^, Maria Carmen Anton^3^, Susana Plaza^3^, Helios Martínez-Banaclocha^6^, María Trabazo^7^, Georgina Morón^7^, Manel Juan^3^, Jordi Yagüe^3^, Ferran Casals^8^, Isabel Badell^7^, Claudia Fortuny^9^, Asunción Vicente^10^, Jordi Anton^1^, Pablo Pelegrin^6^, Juan I. Aróstegui^3^, Anna Mensa-Vilaro^3^

###### ^1^Pediatric Rheumatology; ^2^Confocal Microscopy Unit, Hospital Sant Joan de Déu, Esplugues; ^3^Immunology, Hospital Clinic; ^4^Experimental and Health Sciences, Pompeu Fabra University, Barcelona; ^5^Clinical Immunology Unit, Hospital Sant Joan de Déu, Esplugues; ^6^Instituto Murciano de Investigación Biosanitaria IMIB-Arrixaca, Murcia; ^7^Pediatric BMT Unit, Hospital de Sant Pau; ^8^Genomics, Pompeu Fabra University, Barcelona; ^9^Pediatrics; ^10^Pediatric Dermatology , Hospital Sant Joan de Déu, Esplugues, Spain

####### **Correspondence:** Anna Mensa-Vilaro

**Introduction:** Immunodeficiency is increasingly recognized as a relevant feature in certain novel monogenic autoinflammatory diseases including the often fatal deficiency of actin-interacting protein 1 (AIP1) caused by biallelic *loss-of-function WDR1* mutations.

**Objectives:** To characterize the clinical and immunological features, genetic and molecular mechanisms and treatment outcome, underlying a child combining immunodeficiency and sterile inflammation.

**Methods:** Whole-exome sequencing (WES) was performed in the family to identify the genetic defect. Immunological evaluations and functional assays were performed to demonstrate the pathogenicity of the candidate variant.

**Results:** The patient is a 10-year-old girl born of consanguineous Pakistani parents. She was followed since the age of 5 for recurrent fever, recurrent pyoderma gangrenosum-like, upper respiratory and ocular bacterial infections, one episode of non-bacterial osteomyelitis, anemia, mild-to-moderate thrombocytopenia, neutrophilia, and increase acute-phase reactants. WES detected the novel, homozygous p.(Lys411Asn) *WDR1* variant, classified as likely pathogenic according to ACMG guidelines. Immunological investigations revealed moderate B cell lymphopenia with normal immunoglobulin levels, and normal numbers of T and NK cells. Phalloidin staining of patient’s neutrophils showed increased levels of F-actin compared with healthy subjects. Neutrophil respiratory burst was normal with fMLP stimuli, but repeatedly impaired for phagocytosis of opsonized *E. coli*. Confocal and electronic microscopy reveals aberrant morphology of neutrophils, with nuclear herniations, low cytoplasmatic granule content, and defects in chemotaxis and chemokinetics. Inflammasome-related experiments with patient’s monocytes showed lower levels of both ASC specks formation and active caspase-1 compared with healthy subjects. Finally, she has undergone HLA-identical unrelated hematopoietic stem cell transplantation (HSCT) and she is clinically well with a follow-up time of 50 days.

**Conclusion:** Our results strongly support deficiency of AIP1 as the cause of the disease observed in this patient. Allogeneic HSCT might be a curative option for this severe and rare disease.

**Consent for publication has been obtained from patient**

Yes

**Disclosure of Interest**

None Declared

### Guided poster tour 2B

#### PT2B01 Adenosine deaminase 2 deficiency: correction of the immune defects by gene therapy

##### Immacolata Brigida^1^, Matteo Zoccolillo^1,2^, Raisa Jofra Hernandez^1^, Lucia Sergi Sergi^1^, Giulia Milardi^1,3^, Federica Barzaghi^2,4^, Luigi Naldini^1,3^, Maria Pia Cicalese^1,3,4^, Alessandro Aiuti^1,3,4^, Alessandra Mortellaro^1^

###### ^1^San Raffaele Telethon Institute for Gene Therapy (SR-Tiget), IRCCS San Raffaele Scientific Institute, Milan; ^2^Tor Vergata University, Department of Systems Medicine, Rome; ^3^Vita-Salute San Raffaele University; ^4^Pediatric Immunohematology and Bone Marrow Transplantation Unit, IRCCS San Raffaele Scientific Institute, Milan, Italy

####### **Correspondence:** Alessandra Mortellaro

**Introduction:** Adenosine deaminase 2 deficiency (DADA2) is caused by loss-of-function mutations in the *ADA2* gene. Clinical features include vasculitis, ischemic strokes, intracranial hemorrhages, hematological abnormalities and immunodeficiency mainly affecting B cells. Anti-TNF therapy reduced the ischemic events, controlled vasculitis and partly restored the B-cell function. Allogeneic hematopoietic stem progenitor cells (HSPCs) transplantation attempted in some patients restored ADA2 activity and improved the inflammatory and hematological manifestations in transplanted DADA2 patients. However, the morbidity and mortality of the allogeneic HSPC transplantation remained high and unacceptable in less severe cases.

**Objectives:** Based on the observation that HSPC transplantation may be curative, we hypothesized that strategies based on genetic correction and engraftment of autologous HSPCs could provide a complete cure for DADA2.

**Methods:** CD34^+^ HSPCs isolated from healthy donors and DADA2 patients were transduced with a lentiviral vector (LV) encoding ADA2. HSPCs engraftment and differentiation were assessed *in vitro* and *in vivo* in humanized NSGW41 mice.

**Results:** Transduction of CD34^+^ HSPCs isolated from healthy donors and DADA2 patients with the ADA2-LV efficiently restored ADA2 expression *in vitro* without significant toxicity. Immunological reconstitution of ADA2-transduced CD34^+^ HSPCs was successfully achieved *in vivo* in humanized NSGW41 mice. LV-derived ADA2 expression boosted immune reconstitution of myeloid and B cells *in vivo*.

**Conclusion:** LV-mediated gene transfer restored ADA2 expression and enhanced myeloid and B-cell reconstitution. Future studies will be required to explore the safety and efficacy of HSPC gene therapy approach for DADA2.

**Disclosure of Interest**

None Declared

#### PT2B02 Analysis of the efficacy of treatment on 45 patients with deficiency of adenosine deaminase 2

##### Amanda K. Ombrello^1^, Deborah L. Stone^1^, Karyl Barron^2^, Patrycja M. Hoffmann^1^, Cornelia Cudrici^3^, Anne Jones^1^, Tina Romeo^1^, Dimana Dimitrova^4^, Amit Dotan^5^, Donna Wall^5^, Monica Thakar^6^, Jennifer Kanakry^4^, Daniel Kastner^1^

###### ^1^NHGRI; ^2^NIAID; ^3^NHLBI; ^4^NCI, NIH, Bethesda, United States; ^5^Hospital for Sick Children, Toronto, Canada; ^6^Fred Hutchinson Cancer Research Center, Seattle, United States

####### **Correspondence:** Amanda K. Ombrello

**Introduction:** Since its initial description in 2014, the deficiency of adenosine deaminase 2 (DADA2) has undergone massive phenotypic expansion. The implementation of anti-TNF agents has resulted in a significant reduction in occurrence of lacunar strokes but variable effects on the other disease manifestations. Hematopoietic stem cell transplantation (hSCT) is another potential curative treatment but, as numbers of patients transplanted rise, specific DADA2 complications have emerged**.**

**Objectives:** To analyze the efficacy of treatment modalities used in DADA2 patients in a single center, 45 patient cohort.

**Methods:** Patients with confirmed biallelic mutations in *ADA2* were enrolled and a comprehensive multi-system evaluation was undertaken to establish the extent of disease. From June 2013 forward, all patients with DADA2 were offered treatment with anti-TNF agents. Patients were followed longitudinally on an annual basis. At each visit the patients were followed by a core group of consultants with a battery of laboratory, radiologic, and pathological analyses completed. Patients with significant evidence of bone marrow involvement (pure red cell aplasia [PRCA], immune mediated neutropenia, trilineage bone marrow failure) were referred for hSCT.

**Results: Anti-TNF Analysis:** In total, 42/45 were initiated on anti-TNF agents. At the time of analysis 38/45 continued treatment (4 underwent hSCT and 3 declined treatment). 15/38 were on adalimumab, 20/38 were on etanercept, 2/38 were on golimumab and 1 was on infliximab. Prior to anti-TNF initiation, there were 66 cumulative strokes and post-anti-TNF initiation, there were 0 strokes. There was a statistically significant decrease in inflammatory burden (ESR, CRP) post-anti-TNF initiation. Pre-treatment transient elastography was abnormal in 11/34 with 6/11 normalizing post-treatment.

The inflammatory skin manifestations such as erythema nodosum and vasculitic nodules improved on treatment. There was visible improvement of livedo racemosa but little response to peripheral vascular disease/Raynaud’s phenomenon with 1 patient undergoing 3 partial digit amputations despite treatment. The patient with PRCA remained transfusion dependent. Immune mediated neutropenia was present in 10/38 pre-anti-TNF and persisted in 6/10 post-treatment. Trilineage bone marrow failure developed in 1 patient on anti-TNF.

**hSCT Analysis:** At the time of analysis 4/45 had undergone hSCT (2 for immune mediated neutropenia, 1 for combined PRCA/immune mediated neutropenia, 1 for trilineage bone marrow failure).All had been on anti-TNF agents leading up to hSCT and 1 developed trilineage bone marrow failure after 4 years of anti-TNF treatment. All received reduced intensity conditioning with varying regimens. One patient was transplanted successfully and is doing well 1 year post-transplant. Two additional patients with immune mediated neutropenia experienced graft failure due to host CD8+T lymphocyte attack requiring second transplant. Patient 4 remains hospitalized after decreasing chimerism and reemergent neutropenia necessitated donor lymphocyte infusion. Three additional patients await hSCT.

**Conclusion:** The inflammatory and neurologic manifestations of disease have a robust response to anti-TNF; hepatic and hematologic manifestations have a variable response and vascular manifestations have little response. hSCT can be curative but delayed engraftment/graft failure is a potential complication in patients with neutropenia.

**Disclosure of Interest**

None Declared

#### PT2B03 Long-term retention rate of anakinra in adult onset Still’s disease and predictive factors for treatment response

##### Antonio Vitale^1^, Giulio Cavalli^2^, Serena Colafrancesco^3^, Roberta Priori^3^, Guido Valesini^3^, Lorenza Maria Argolini^4^, Elena Baldissera^2^, Elena Bartoloni^5^, Daniele Cammelli^6^, Giovanni Canestrari^7^, Jurgen Sota^1^, Elena Cavallaro^8^, Maria Grazia Massaro^9^, Piero Ruscitti^10^, Paola Cipriani^10^, Ginevra De Marchi^8^, Salvatore De Vita^8^, Giacomo Emmi^6^, Gianfranco Ferraccioli^7^, Micol Frassi^11^, Roberto Gerli^5^, Elisa Gremese^7^, Florenzo Iannone^12^, Giovanni Lapadula^12^, Giuseppe Lopalco^12^, Raffaele Manna^9^, Alessandro Mathieu^13^, Carlomaurizio Montecucco^14^, Marta Mosca^15^, Ilaria Piazza^16^, Matteo Piga^13^, Irene Pontikaki^4^, Micol Romano^4^, Silvia Rossi^14^, Maurizio Rossini^16^, Elena Silvestri^6^, Chiara Stagnaro^15^, Rosaria Talarico^15^, Angela Tincani^11^, Ombretta Viapiana^16^, Gianfranco Vitiello^6^, Paola Galozzi^17^, Paolo Sfriso^17^, Carla Gaggiano^18^, Donato Rigante^19^, Lorenzo Dagna^2^, Roberto Giacomelli^10^, Luca Cantarini^1^ and “Working Group” of Systemic Autoinflammatory Diseases of SIR (Italian Society of Rheumatology)

###### ^1^Research Center of Systemic Autoinflammatory Diseases and Behçet’s Disease Clinic Surgery and Neurosciences, Department of Medical Sciences, Surgery and Neurosciences, University of Siena, Siena; ^2^Unit of Immunology, Rheumatology, Allergy and Rare Diseases (UnIRAR), IRCCS San Raffaele Scientific Institute, Milan; ^3^Department of Internal Medicine and Medical Specialties, Sapienza University of Rome, Rome; ^4^Division of Rheumatology, ASST Gaetano Pini, Milan; ^5^Rheumatology Unit, Department of Medicine, University of Perugia, Perugia; ^6^Department of Experimental and Clinical Medicine, University of Firenze, Firenze; ^7^Institute of Rheumatology and Affine Sciences, Division of Rheumatology, Catholic University of the Sacred Heart, Rome; ^8^Department of Medical and Biological Sciences, Rheumatology Clinic, University of Udine, Udine; ^9^Periodic Fever Research Center, Institute of Internal Medicine, Catholic University of the Sacred Heart, Fondazione Policlinico A. Gemelli, Rome; ^10^Department of Biotechnological and Applied Clinical Science, Division of Rheumatology, University of L’Aquila, L'Aquila; ^11^Rheumatology and Clinical Immunology, Spedali Civili and Department of Clinical and Experimental Sciences, University of Brescia, Brescia; ^12^Rheumatology Unit,Department of Emergency and Organ Transplantation, University of Bari, Bari; ^13^Rheumatology Unit, Department of Medical Sciences, University and AOU of Cagliari, Cagliari; ^14^Department of Rheumatology, IRCCS Policlinico San Matteo Foundation, University of Pavia, Pavia; ^15^Rheumatology Unit, Department of Clinical and Experimental Medicine, University of Pisa, Pisa; ^16^Rheumatology Unit, Department of Medicine, University of Verona, Verona; ^17^Department of Medicine DIMED, Rheumatology Unit, University of Padua, Padua; ^18^Clinical Pediatrics, Department of Molecular Medicine and Development, University of Siena, Siena; ^19^Institute of Pediatrics, Università Cattolica Sacro Cuore, Fondazione Policlinico Universitario A. Gemelli I.R.C.C.S., Rome, Italy

####### **Correspondence:** Antonio Vitale

**Introduction:** Anakinra (ANA) is an effective treatment choice in patients with adult onset Still’s disease (AOSD). Variables affecting treatment survival include loss of efficacy or adverse events, but also the decision to discontinue treatment after long-term clinical remission.

**Objectives:** Aims of this study were: i) to assess the drug retention rate (DRR) of ANA during a long-term follow-up looking for any difference related to the line of biologic treatment, the concomitant use of conventional disease modifying anti-rheumatic drugs (cDMARDs) and the different type of AOSD (systemic *versus* chronic articular); ii) to identify predictive factors of lack of efficacy, loss of efficacy and ANA withdrawal owing to long-term remission.

**Methods:** AOSD patients classified according with Yamaguchi criteria and treated with ANA were retrospectively enrolled in 18 Italian tertiary Centers. Demographic, laboratory, clinical and therapeutic data related to the start of ANA (*baseline*), the 3-month assessment and the last follow-up visit while on ANA treatment were retrospectively collected and statistically analyzed.

**Results:** One hundred and forty-one AOSD patients (48 males, 93 females) treated with ANA for a mean period of 35.96±36.05 months were enrolled. The overall DRR of ANA was 44.6% and 30.5% at the 60- and 120-month assessments, respectively, with no significant differences between: i) biologic naïve patients and those previously treated with other biologics (log-rank *p*=0.97); ii) monotherapy and concomitant use of cDMARDs (log-rank *p*=0.45); iii) systemic and chronic articular types of AOSD (log-rank *p*=0.67). No variables collected at *baseline* could predict primary inefficacy, while the number of swollen joints at baseline was significantly associated with secondary inefficacy (*p*=0.01, OR=1.194, C.I. 1.043-1.367). The typical AOSD skin rash was negatively related with ANA withdrawal owing to long-term remission (*p*=0.03, OR=0.224, C.I. 0.058-0.863).

**Conclusion:** Long-term DRR of ANA has been found excellent and is not affected by different lines of biologic treatment, concomitant use of cDMARDs, or type of AOSD. The risk of losing ANA efficacy increases along with the number of swollen joints at the start of therapy, while the typical skin rash is a negative predictor of ANA withdrawal related to sustained remission.

**Disclosure of Interest**

None Declared

#### PT2B04 The anti-inflammatory cytokine interleukin 37 is an endogenous inhibitor of trained immunity

##### Giulio Cavalli^1,2^, Mark Gresnigt^3^, Travis Nemkov^4^, Rob Arts^2^, Angelo D'Alessandro^4^, Silvia Giugliano^5^, Elan Eisenmensser^4^, Lorenzo Dagna^1^, Leo Joosten^2^, Mihai Netea^2^, Charles Dinarello^2,6^

###### ^1^Unit of Immunology, Rheumatology, Allergy and Rare Diseases (UniRAR), Vita-Salute San Raffaele University, Milan, Italy; ^2^Department of Medicine, Radboud university Medical Centre, Nijmegen, Netherlands; ^3^Microbial Pathogenicity Mechanisms, Leibniz Institute for Natural Product Research and Infection Biology, Jena, Germany; ^4^Department of Biochemistry and Molecular Genetics, University of Colorado Denver, Aurora, CO, United States; ^5^Mucosal Immunology and Microbiota Unit, Humanitas University, Rozzano, Italy; ^6^Department of Medicine, University of Colorado Denver, Aurora, CO, United States

####### **Correspondence:** Giulio Cavalli

**Introduction:** Trained immunity (TI) is a de-facto innate immune memory program induced in monocytes/macrophages by exposure to pathogens or vaccines, which evolved as a protective mechanism against infections. TI is characterized by rewiring of functional, epigenetic and metabolic programs of innate immune cells such as monocytes and macrophages, which sustain enhanced production of pro-inflammatory cytokines. Since aberrant activation of TI is implicated in inflammatory diseases, tight regulatory mechanisms are likely in place, but the mechanisms responsible for this modulation remain elusive.

**Objectives:** Scope of this study was to evaluated the role of IL-37, an anti-inflammatory cytokine that curbs inflammation as well as modulates metabolic pathways, as an endogenous regulator of trained immunity.

**Methods:** The effects of recombinant IL-37 were evaluated in a mouse model of TI induced by the administration of beta-glucan in vivo (survival to a lethal inoculum of infectious agents, production of inflammatory cytokines, recruitment of inflammatory cells at the sites of infection). Subsequently, the effects of IL-37 were evaluated ex vivo on splenic and bone marrow monocytes (production of inflammatory cytokines, metabolomic analysis of the activation status of the main pathways of cellular energy metabolism). Finally, we evaluated the association between IL-37 gene polymorphisms and the induction of TI in monocytes of healthy donorswith in vitro functional studies.

**Results:** The exogenous administration of IL-37 abrogates the pro-inflammatory effects of TI, significantly reducing the production of pro-inflammatory cytokines and the survival of experimental animals subjected to a disseminated infection model. The inhibitory effects of IL-37 on TI are also associated with reduced recruitment of neutrophils at sites of inflammation. IL-37 and TI programs have differential and opposite effects on the modulation of cellular energy metabolism of monocytes. In humans, polymorphisms in the IL-37 gene are associated with reduced activation of TI programs and reduced production of inflammatory cytokines by healthy donor monocytes.

**Conclusion:** In conclusion, IL-37 emerges as an endogenous regulator of TI, which makes this cytokine a potential therapeutic target in immune-mediated pathologies.

**Disclosure of Interest**

None Declared

#### PT2B05 Interleukin 18 gene expression by human monocytes is controlled by type I interferon

##### Emely Verweyen^1^, Dirk Holzinger^2^, Helmut Wittkowski^1^, Peter Pickkers^3^, Dirk Foell^1^, Christoph Kessel^1^

###### ^1^Pediatric Rheumatology and Immunology, University Children's Hospital, Muenster; ^2^Child and Adolescent Medicine, University Hospital, Essen, Germany; ^3^Intensive Care Medicine, Radboud University Medical Center, Nijmegen, Netherlands

####### **Correspondence:** Christoph Kessel

**Introduction:** Interleukin (IL) 18 is a member of the IL-1 cytokine family and has a pivotal role in natural killer and T cellular interferon (IFN) γ production but its precise function in inflammatory diseases as well as regulatory mechanisms underlying IL-18 expression still remain poorly understood.

**Objectives:** Both IL-1β and IL-18 are particularly associated with the autoinflammatory diseases familial mediterranean fever (FMF) or systemic juvenile idiopathic arthritis (sJIA) and IL-18 is thought to drive autoinflammation or infection associated macrophage activation syndrome (MAS). Apart from gathering evidence for the pathophysiological implications of IL-1β and IL-18, several studies have investigated the genetic control of IL-1β expression, highlighting a role for NF-κB in IL-1β transcription. Albeit there are some studies that investigated IL-18 expression by either murine or human cells, it still remains poorly understood whether and how this is regulated.

**Methods:** Cytokine expression in plasma of healthy individuals subjected to lipopolysacharide (LPS) re-challenge experiments was quantified. LPS-stimulated primary human monocytes isolated from healthy donors were analyzed for cytokine expression on gene and protein level over time as well as following various drug or recombinant cytokine treatments.

**Results:** In plasma of healthy individuals subjected to LPS re-challenge experiments we initially observed that, in contrast to TNFα, IL-6 or IL-1β, the IL-18 levels were unaffected by LPS-tolerance. Similarly, in endotoxin desensitization experiments on primary human monocytes, IL-18 gene expression was resistent to endotoxin tolerance and appeared to rather benefit from endotoxin re-challenge. When studying time kinetics of cytokine gene expression, we found that IL-1 and IL-18 followed identical secretion but completely divergent gene expression patterns, thus providing an explanation for observations on *IL18* expression in LPS re-challenge experiments. When studying triggers for the observed attenuated gene expression kinetics of IL-18, we found that both IL-18 gene and protein expression upon LPS-stimulation of primary human monocytes was not affected by other prominent inflammatory cytokines but was sensitive to interference with JAK/STAT-signaling and could be modulated by type I interferon priming of cells or STAT1 gain-of-function. While by itself type I interferon did not induce IL-18 expression, IFNβ-neutralization during LPS-stimulation blunted *IL18* transcription. Further, microtubule-destabilizing drugs, such as colchicine, completely abrogated monocytic *IL18* expression, which could be reversed by addition of type I interferons.

**Conclusion:** Our data suggest monocytic IL-18 expression to require cooperate toll-like receptor and type I interferon signaling, which has implications for IL-18 expression during viral infections and its overexpression in autoinflammation and MAS.

**Disclosure of Interest**

None Declared

#### PT2B06 Glycogen synthase kinase 3B regulates TLR3-mediated antiviral response

##### Ryeojin Ko, Soo Young Lee

###### Department of Life Science and the Research Center for Cellular Homeostasis, Ewha Womans University, Seoul, Korea, Republic Of

####### **Correspondence:** Ryeojin Ko

**Introduction:** Toll-like receptor 3 (TLR3) plays a critical role in the antiviral immune response. The protein tyrosine kinase BTK has been reported to regulate signal pathways for TLR3-mediated antiviral gene expression. However, the regulation of BTK activity in TLR3 signaling remains unclear.

**Objectives:** Here we investigate the molecular mechanisms underlying GSK3b regulation of BTK in TLR3-mediated antiviral gene expression.

**Methods:** To determine whether GSK3b regulates TLR3-mediated antiviral response, we establish the GSK3b knockdown stable macrophage cell lines.

**Results:** We demonstrate that GSK3b positively regulates TLR3 signaling via BTK activation. After poly I:C stimulation, GSK3b interacts with BTK in a time-dependent manner. Furthermore, suppression of GSK3b expression or its kinase activity significantly reduces the poly I:C-induced BTK activation and antiviral gene expression such as type I interferon (IFN) and IFN stimulated gene (ISG).

**Conclusion:** Together, our results suggest that GSK3b is critical for antiviral response by regulating TLR3 signaling and is a potential molecular therapeutic target in autoimmune disease.

**Disclosure of Interest**

None Declared

#### PT2B07 Anticytokine therapy is successful inprevention and treatment OFBCG-associated inflammatory syndrome (IS) after hematopoietic stem cell transplantation (HSCT) in patients with severe combined immunodeficiencies (SCID)

##### Alexandra Laberko^1^, Irina Shipitsina^2^, Svetlana Rodigina^2^, Sergei Blagov^2^, Daria Yukhacheva^1^, Elena Deripapa^1^, Anna Kozlova^1^, Yulia Rodina^1^, Larisa Shelikhova^2^, Dmitrii Balashov^2^, Anna Shcherbina^1^

###### ^1^Immunology; ^2^Hematopoietic Stem Cell Transplantation, National Medical Research Center of Hematology, Oncology and Immunology Named After D. Rogachev, Moscow, Moscow, Russian Federation

####### **Correspondence:** Anna Shcherbina

**Introduction:** Immune recovery IS is a well-known complication in patients with HIV-driven acquired immunodeficiency syndrome after initiation of antiretroviral therapy.It is caused by cytokine storm due to recovering CD4+ lymphocyte activation in response to persistingpathogens. We observed similar phenomena in patients with SCID after allogenic HSCT.

**Objectives:** To report BCG-related IS in a group of SCID patients.

**Methods:** 22 SCID patients, vaccinated with BCG, received allogenic haploidentical HSCT with TCRab/CD19 graft depletion in our Center between 2012 and 2018. Seven patients had symptoms of BCGitis before HSCT, at the moment of HSCT BCGitis was controlled by antimycobacterial drugs in all of them.

Post-transplant immunosuppressive therapy before November 2016 ( 11 patients) included tacrolimus, in 2 cases in combination with short course of methotrexate. From November 2016 11 patients received graft versus host disease and cytokine release syndrome prophylaxis with tocilizumab 8mg/kg days -1 , +14, + 28, abatacept 10mg/kg days -1, +5, +14, +28.All patients have been receiving from 2 to 4 antimycobacterial drugs for BCGitis prophylaxis at HSCT and in posttransplant period until full immune recovery.

**Results:** 5\22 patients developed early BCG-related IS (4/11 prior to tocilizumab introduction, and only 1/11 – with tocilizumab). Three of them presented at days +4, +6 and +12 after HSCT with sustained fever, focal BCGitis exacerbation and one additionally developed macrophage activation syndrome (MAS). All received therapy with steroids and IL-1 inhibitors, in one of them IS symptoms were controlled, but eventually all three died shortly after. One patient without signs of BCGitis before HSCT at day +38 developed IS with symptoms of systemic BCG infection and MAS. He was successfully treated with combination of etoposide and IL-6 receptor inhibitors – tocilizumab 10mg/kg. In one patient local BCGitis resolved without treatment. In most cases of early IS significant elevation of TCRgd cell level was observed.

Six patients (5/6 with tocilizumab prophylaxis ) had immune recovery associated IS 2,5 - 5,5 months after HSCT (M 4,4 months), accompanied by CD3+ lymphocytes increase in peripheral blood. Symptoms included focal BCGitis in all 6 (5 of them had no BCGitis before HSCT), fever in 3, lymphadenopathy in 3, MAS in 1. In four patients monotherapy of anakinra 7-12mg/kg/day was used with full response within 2-4 weeks. They were later switched to canakinumab 5mg/kg every 4 weeks for the next 1-3 months. In one patient IS was controlled by 2 infusions of tocilizumab 10mg/kg every 4 weeks. The patient who developed MAS received anakinra 10mg/kg in combination with steroids with full resolution of symptoms.

**Conclusion:** BCG vaccinated SCID patients are at high risk of BCG-related early IS after HSCT. Anticytokine therapy with IL6 receptor inhibitor is an effective option for prevention of early BCG-related IS. IL1 inhibitors lead to full resolution of BCG-related immune recovery IS.Evidence-based protocols for IS prevention and treatment are needed.

**Disclosure of Interest**

None Declared

#### PT2B08 Allogeneic haematopoietic stem cell transplantation (HSCT) outcome in immunedysregulation and rheumatologic patients: a single center 19 years experience

##### Sara Signa^1,2^, Andrew Gennery^1^, Sophie Hambleton^1^, Terence Flood^1^, Andrew Cant^1^, Mario Abinun^1^, Mary Slatter^1^

###### ^1^Great North Children's Hospital, Newcastle upon Tyne Hospitals NHS Foundation Trust and Primary Immunodeficiency Group, Institute of Cellular Medicine, Newcastle upon Tyne University, Newcastle upon Tyne, United Kingdom; ^2^University of Genoa and Center for Autoinflammatory Diseases and Immunodeficiencies-Clinics of Pediatrics and Rheumatology , G.Gaslini Institute, Genoa, Italy

####### **Correspondence:** Sara Signa

**Introduction:** Haematopoietic stem cell transplantation (HSCT) is a potential treatment for severe and conventional therapy-resistant immune dysregulation disorders (autoinflammatory diseases ,complex autoimmune diseases, complex rheumatologic diseases).However, it still remains the ‘last resort therapy”, particularly in pediatric age.

**Objectives:** To present the outcome of HSCT in 55 children with immune dysregulation in a single center over a 19 year period of time.

**Methods:** We retrospectively collected cases of pediatric patients who underwent allogeneic HSCTat Great North Children’s Hospital, Newcastle upon Tyne, UK between 01/1999 -06/2018 for immune dysregulation, rheumatologic and autoinflammatory disorders.Medical charts were reviewed and the following data were extracted: patient demographics, diagnosis, indication for HSCT, donor-recipient matching, conditioning regimen, and graft composition. Post-transplantation data were collected on occurrence of GVHD, infections, and relapse of initial (or occurrence of new, secondary, post-HSCT) immunedysregulation.

**Results:** We identified 55 patients who underwent allogeneic HSCT: 8 presented with a defined Rheumatologic disease (5 children JIA, 3 with systemic lupus erythematosus), 7 patients with CTLA-4 deficiency, 6 with STAT 3 gain-of-function mutation disease,6 with IPEX syndrome, 7 with autoinflammatory disorders, 3 children with ALPS, 5 with autoimmune enteropathy, 2 with STAT1 gain-of-function disease, 7 with complex autoimmuny features, and two each with RAS associated autoimmune lymphoproliferative disorder (RALD) and FADD deficiency. 41 patients presented with a monogenic disease, although only 21 were diagnosed pre- HSCT.Mean age of symptom onset was 3.5 years (range: birth-15.34 years). Mean age at HSCT was 8.5 years (range: 0.25-19.34 years). Patients underwent HSCT from 10/10 or 12/12 HLA matched unrelated donors (n 29), 9/10 HLA mismatched unrelated donor (n 5), matched sibling donor (n 14), 9/10 HLA mismatched related donor (n 3) or haploidentical TCR alpha/beta-CD19+ depleted parental donor (n 4). Most patients received reduced toxicity conditioning with fludarabine 3, treosulfan, alemtuzumab(n 27). Peripheral blood was the stem cell source in most cases (n 32). Most received cyclosporine (CsA) and mycophenolate mofetil (MMF) as GVHD prophylaxis (n 37). One did not engraft, needing a rapid second HSCT procedure. 39 patients showed evidence of viral replication (61%) , sometimes from multiple pathogens. Acute GVHD was usually mild and confined to skin (grade I/II, n=27, 49%).Severe acute GVHD occurred in 6 patients (grade III-IV GVHD -10%; 3 died) and two patients experienced extensive chronic GVHD, both deceased.With a median follow-up of 6 .67years (range, 2 months-18 years), 43 of 55 patients were alive and most are either cured and completely healthy (n=21) or significantly improved but with pre-existing end-organ damage (n=11). 19 patients experienced new autoimmune phenomena post-transplant, mostly involving thyroid or hematologic-related.Among the 12 deceased patients, one died from non-transplant related diabetic ketoacidosis, whereas five patients died of GVHD-related causes, five due to infectious causes and one due to extreme induction toxicity, giving a transplant related mortality (TRM) of 20%. Mean age of death was 11 years (range: 0.67-21 years).

**Conclusion:** Allogeneic HSCT can be an effective therapeutic option for severe refractory immune dysregulation in childhood, potentially curative. Appropriate and timely patient selection is fundamental to minimise HSCT-related complications.

**Disclosure of Interest**

S. Signa Grant / Research Support from: Research Support from: EFIS-Immunology Letters (IL) Short-Term Fellowship, A. Gennery: None Declared, S. Hambleton: None Declared, T. Flood: None Declared, A. Cant: None Declared, M. Abinun: None Declared, M. Slatter: None Declared

#### PT2B09 Global profiling and molecular characterization of the autoantibody repertoire in APECED

##### JohnM. Lindner^1^, Alexander Krämer^1^, Xavier Leber^1^, Valerie Caballero^1^, Frank Kolbinger^1^, Michail S. Lionakis^2^, Steven Holland^2^, Elisabetta Traggiai^1^

###### ^1^Novartis Institutes for BioMedical Research, Basel, Switzerland; ^2^National Institutes of Health, Bethesda, United States

####### **Correspondence:** Elisabetta Traggiai

**Introduction:** Autoimmune polyendocrinopathy candidiasis ectodermal dystrophy (APECED, also called APS-1), is a phenotypically pleiotropic disease caused by the breakdown of central T-cell tolerance due to mutation of the autoimmune regulator (AIRE) gene. The presence of autoreactive antibodies (Ab) in APECED has implications for T cell-mediated governance of B-cell tolerance. In particular, soluble factors such as cytokines and antimicrobial peptides are frequent autoantigens in APECED. To fully characterize the humoral response against these and other targets, we tested donor sera for reactivity against more than 3000 secreted proteins using customized protein microarrays.

**Results:** Applying stringent evaluation methods, we identified more than 50 reactivities enriched among 26 APECED patients relative to 19 healthy donors. We subsequently validated several targets by ELISA, then screened patient IgG memory B cells for Ab of desired specificities using recently developed high-throughput, multiplexed techniques for culture, screening, and cloning. Of particular relevance is the production of Ab against interleukin (IL)-17, given the relationship between TH17-mediated fungal control and chronic candidiasis as a common clinical feature of APECED. To understand the composition and functionality of these Ab, we cloned 15 antigen-specific IgG molecules from 3 APECED patients, and found clear patient-specific skewing toward IL-17A or IL-17F, with some Ab showing cross-reactivity. Furthermore, several also bound the heterodimeric IL-17AF in addition to their respective homodimers, and one Ab specifically bound only the heterodimer, the latter one a specificity which has not yet been described. All Ab showed extensive somatic hypermutation, had sub-nanomolar affinities, and functionally block the interaction between IL-17 and its receptor.

**Conclusion:** These features indicate a clear history of interaction with antigen-specific T helper cells, and suggest that autoantibodies in APECED can disrupt immune control, offering insights into the therapeutic control both of APECED and other human diseases.

**Disclosure of Interest**

None Declared

### Monogenic autoinflammatory diseases (clinical)

#### P2001 In vitro NLRP3 inflammasome activation assay assists diagnosis of genetically negative CAPS patients and guides anti-IL1 therapy

##### Leonardo O. Mendonca^1,2,3^, Myrthes T. Barros^2^, Tereza Robazzi^4^, Pedro F. Giavina-Bianchi^2^, Francesco Caroli^5^, Alice Grossi^5^, Jorge Kalil^2^, Fabio F. Morato Castro^2^, Isabella Ceccherini^5^, Marco Gattorno^6^, Alessandra Pontillo^3^

###### ^1^International Center for Autoinflammatory Diseases and Primary Immunodeficienceis, Istituto Giannina Gaslini, Genoa, Italy; ^2^Autoinflammatory Unit, University of São Paulo, School of Medicine; ^3^Immunogenetics Laboratory, Biomedical Science Institute, University of São Paulo, Sao Paulo; ^4^Pediatric Rheumatology Unit, School of Medicine of Bahia, Federal University of Bahia, Salvador, Brazil; ^5^UOC Medical Genetics; ^6^Center for Autoinflammatory Diseases and Primary Immunodeficienceis, Istituto Giannina Gaslini, Genoa, Italy

####### **Correspondence:** Leonardo O. Mendonca

**Introduction:** Constitutive activation of NLRP3 inflammasome and the consequent elevate production of inflammatory cytokine IL-1ß are the pathogenic mechanisms involved in CAPS syndromes. However due to the heterogeneity of clinical presentation and to the missing of genetic proving in 40% of patients, both diagnosis and therapeutic choice are often tricky.

Cryopirin/NLRP3-associated periodic syndromes (CAPS) is a group of rare autoinflammatory diseases characterized by early onset urticarial rash and periodic fever, positive acute reactants markers (CRP, SAA), arthritis and neurologic involvement, including ocular disorders and progressive deafness. The constitutive activation of NLRP3 inflammasome and the consequent elevate production of inflammatory cytokine IL-1ß are the pathogenic mechanisms involved in CAPS. Gain-of-function mutations in NLRP3 were detected in about 60% of patients. Early diagnosis and quick start of anti-IL-1 treatment are very important to prevent severe complications, however due to the heterogeneity of clinical presentation and to the missing of genetic proving in 40% of patients, both diagnosis and therapeutic choice are often trickThe constitutive activation of NLRP3 inflammasome and the consequent elevate production of inflammatory cytokine IL-1ß are the pathogenic mechanisms involved in a group of rare autoinflammatory diseases grouped as Cryopirin Associated Periodic Syndromes. Gain-of-function mutations in *NLRP3* were detected in about 60% of patients. Early diagnosis and quick start of anti-IL-1 treatment are very important to prevent severe complications, however due to the heterogeneity of clinical presentation and to the missing of genetic proving in 40% of patients, both diagnosis and therapeutic choice are often trickyThe constitutive activation of NLRP3 inflammasome and the consequent elevate production of inflammatory cytokine IL-1ß are the pathogenic mechanisms involved in a group of rare autoinflammatory diseases grouped as Cryopirin Associated Periodic Syndromes. Gain-of-function mutations in *NLRP3* were detected in about 60% of patients. Early diagnosis and quick start of anti-IL-1 treatment are very important to prevent severe complications, however due to the heterogeneity of clinical presentation and to the missing of genetic proving in 40% of patients, both diagnosis and therapeutic choice are often tricky.

**Objectives:**

Demonstrate the usefulness of an *in vitro* test for NLRP3 inflammasome activation as supportive method for CAPS diagnosis and therapeutic choice.

**Methods:** Five patients (P1-P5) with a suspect of CAPS were included in a pilot study between 2016 and 2018. All patients were genetically sequenced using gene panels for mutations in candidate genes (*NLRP3, NLPR12* and *NLRC4*). Monocytes were stimulated with LPS and ATP. IL-1ß concentration was measured in serum and culture supernatants by ELISA.

**Results:** All patients presented clinical features compatible with a CAPS. Candidate genes screening revealed a pathogenic mutation in *NLRP12* (exon 3; c.C910T; p. H304Y) in P1 and a variant of uncertain significance (VUS) in *NLRP3* (exon 4; c.A2176G; p.S726G) in P2. The other 3 patients were genetically negative. Monocytes isolated from P2, P3 and P4 produced high level of IL-1ß when challenged with LPS, and ATP did not amplify cytokine production, suggesting a defect in NLRP3 inflammasome. P1 and P5 produced normal levels of IL-1ß. According with NLRP3-IA test results, P3 and P4 were successfully treated with anti-IL1. P2 presented also an IgM monoclonal gammopathy, therefore receiving a final diagnosis of Schnitzler Syndrome (SS); colchicine ameliorated his clinical presentation. P5 (genetically negative and NLRP3-IA test negative) was successfully treated with corticosteroids.

**Conclusion:** These results demonstrate the convenience of the NLRP3-IA test in the examination of suggestive CAPS and SS patients, especially for those with negative genetic test, to (1) confirm the diagnosis and to (2) guide the therapeutic choice.

**Disclosure of Interest**

None Declared

#### P2002 Vaccination safety and coverage in a single cohort of autoinflammatory syndromes in Italy

##### Leonardo O. Mendonca, Enrica Toniolo, Stefano Volpi, Roberta Caorsi, Marta Bustaffa, Matteo D'Alessandro, Sara Signa, Marco Gattorno

###### International Center for Autoinflammatory Diseases and Primary Immunodeficienceis, Istituto Giannina Gaslini, Genoa, Italy

####### **Correspondence:** Leonardo O. Mendonca

**Introduction:** Vaccine-preventable diseases are emerging after anti-vaccine movements appear. In AID, vaccine triggered-disease is a well known phenomena for Hyper-IgD/Mevalonate-Kinase Deficiency (MKD). However, recent publications stressed out doubts in physicians and families, regarding severe flares after pneumococcus vaccine in CAPS or that PFAPA patients does not achieve sufficient and protective levels of antibodies.

**Objectives:** This work aims to evaluate the vaccination coverage of Italian Vaccination Schedule and the prevalence of adverse reactions or disease induced flare to vaccine in a single cohort of AID in Italy.

**Methods:** An anamnestic questionnaire was applied to outpatients, followed at AID Unit, Istituto Giannina Gaslini from august 2017 to august 2018. Data acquired was revised for quality of information. Data of triggers in Autoinflammatory disease from EUROFEVER registry was obtained for statistical reference.

**Results: Triggers in AID Eurofever Registry:** In august 2018 a total of 3783 patients were enrolled in EUROFEVER registry and 50,43%, 1908 were female. The average of age symptoms started was 7.04 years (standard deviation of 9,48 years, minimal of 0 and maximum of 75,92 years).The distribution among the periodic hereditary fevers were: 28,75% were FMF (n=1081); 17,66% were PFAPA (n=666); 7,85% (n=297) were CAPS; 7,16% were TRAPS (n=271) and 5,39% were MKD (n=204). Vaccine triggered disease was most common in MKD with 70%. PFAPA, TRAPS and UND presented with similar prevalence of reactions, around 20%. CAPS had 12,34% of vaccine reactions identified. FMF and inflammatory bone disorders carried 6% and 3%, respectively. Excluding other causes of reactions, and isolating just vaccine as cause, MKD still remains the top of the rank (7,14%). Just PFAPA and UND had more than 1% and CAPS, TRAPS, FMF and inflammatory bone disorders had all less than 1%. **IGG cohort:** 150 questionnaires were distributed with 70% rate of response. Quality of data was 100% for coverage and adverse reactions. Data of 105 patients could be obtained distributed as PFAPA (n=26); CAPS (n=5); TRAPS (n=6); FMF (n=14); MKD (n=8); Inflammatory Bone Disorders (CRMO and PAPA, n=4) and Undefined Inflammatory Diseases, UND, (n=41). Rate of converage was lower than 90% for Hib3 (83,11%) , all measles containing vaccine MMR/MMRV (88,9%) and for Rota C (1,85%). For DTP3, Hep3, PCV3 and IPV the rate of coverage was higher than 90% for all vaccines. A total of 13 severe/serious reactions was observed following: 5 after DTPA+IPV (1 PFPAPA; 2 TRAPS, 1 MKD and 1 UND); 2 after Hep B (TRAPS); 1 after Hib (PFAPA); 1 after P10/13 (PFAPA); 4 after MPR (1 PFAPA, 1 TRAPS, 1 MKD and 1 UND) and 1 afterHepatitis C (1 PFAPA). The general rate of severe reaction per shot was 7,7 for every 1000 shots and no severe infection, death, persistent or significant disability or life-threatening condition was observed. Just one patient, MKD, with severe/serious flare required hospitalization due to disease activity following pneumocoocal vaccine.

**Conclusion:** Vaccination in AID is a distinct and a potential public health problem. Specific recommendations for vaccination in AID are extremely necessary as well as further investigations for immunologic protection.

**Disclosure of Interest**

None Declared

#### P2003 Peripheral T regulatory lymphocytes and apoptosis expression in a large group of autoimmune, multifactorial and autoinflammatory diseases reveals specific clinical manifestations

##### Leonardo O. Mendonca^1^, Marta Bustaffa^1^, Caterina Matucci-Cerinic^1^, Ignazia Prigione^1^, Stefano Volpi^1^, Roberta Caorsi^1^, Sabrina Chiesa^1^, Francesca Schena^1^, Alice Grossi^2^, Paola Terranova^3^, Isabella Ceccherini^2^, Maurizio Miano^3^, Marco Gattorno^1^

###### ^1^International Center for Autoinflammatory Diseases and Primary Immunodeficienceis; ^2^UOC Medical Genetics; ^3^Pediatric Hematology Unit, Istituto Giannina Gaslini, Genoa, Italy

####### **Correspondence:** Leonardo O. Mendonca

**Introduction:** The application of the semantical immunological division into innate and adaptive immunity to systemic inflammatory syndromes segregates autoinflammatory from autoimmune diseases. Nonetheless a large number of conditions falls down into undefined systemic inflammatory syndromes and some share clinical characteristics with immunedysregulation syndromes such as Autoimmune Lymphoproliferative Syndromes (ALPS) characterized by activation of apoptosis pathway.

**Objectives: Characterize c**linically, immunological, molecular and therapeutically a group of patients with high expression of apoptosis pathway. Describe the main diferences expressions of regulatory T cells in a large group of autoinflammatory, autoimune and multifactorial diseases.

**Methods:** Retrospective review of all patients submitted, from 2015 to 2018, to flow citometry, for specific ALPS markers and markers of lymphocyte apoptosis: CD3+TCRαβ+CD4-CD8-(αβ-DNTCs), CD19+CD27+(Memory B), TCR αβ+ B220+, CD3CD25+/CD3HLA-DR+ RATIO. Furthermore NK cells, NKT-like cells (CD3+CD16+CD56+), B cells TCRγδ+, Treg, Tnaive and Tmemory cells , sFAS ligand, interleukin 18, interleukin 10, FAS-apoptosis were also studied. A similar flow cytometry composed of healthy Italian controls was used as reference. All clinical and laboratory results were statistically analyzed.

**Results: A total 2089 “ALPS-flow” was realized between 2015-2018. Data that could not be accessed or repeated was ruled out. Finally,** 562 flow cytometry was selected and revised. Results divided by age resulted in (number ;female ratio ; average age flow was performed): 1-12 months (9; 55,5%; 0,78); 1-5 years (174; 43,67%; 3,13); 5-10 years (150; 43,3%; 7,25) and > 10 (220 ; 62,33%; 19,5) and no difference between healthy controls were observed. High levels (>1,8) Double negative T cells (CD4-CD8-(αβ-DNTCs) were present in 38 patients carrying the diagnosis of ALPS/ALPS-like, 14 patients with PFAPA, 30 patients with undefined syndromes and in 9 patients with classical autoinflamamtory diseases. 13 patients fulfilled the four flow criteria for apoptosis (1 ALPS, 1 PFAPA, 1 AID, 6 UIS, 1 autoimmune and 1 SJIA). Clinical and immunological comparison was done between those who had complete response to sirolimus or micofenolate. 28 patients were genetically sequenced for large genetic pannels.

**Conclusion: The “alps flow” is an useful instrument to guide clinical evaluation in undefined inflammatory syndromes and can guide specific therapy. Further evaluations are necessary to understand the immunological observation of apoptosis activation in this group of patients.**

**Disclosure of Interest**

None Declared

#### P2004 Association of gene polymorphism with autoinflammatory syndromes in patients with palindromic rheumatism and intermittent hydrathrosis

##### Takako Miyamae, Yumi Tani, Takayuki Kishi, Manabu Kawamoto, Yasushi Kawaguchi, Atsuo Taniguchi, Hisashi Yamanaka

###### Institute of Rheumatology, Tokyo Women's Medical University, Tokyo, Japan

####### **Correspondence:** Takako Miyamae

**Introduction:** Palindromic rheumatism (PR) is defined as articular and para- articular inflammation that lasts from a few hours to several days, which resolves spontaneously and is associated with increase in inflammatory markers during active disease. It is characterized by recurrent joint manifestations without residual joint destruction. Intermittent hydrarthrosis (IH) is a chronic condition of unknown cause characterized by recurring, temporary episodes of fluid accumulation in the knee. The cause is unknown but allergic and auto-inflammatory mechanisms have been proposed. More recently, specific association with the Mediterranean fever gene, MEFV, has been proposed. So, with some individuals carrying gene mutations (MEFV and also TRAPS-related genes), the native immune system seems to plays a role in the development of IH.

**Objectives:** We evaluated polymorphisms of the genes responsible for AID and clinical characteristics in patients diagnosed as PR with IH.

**Methods:** Among patients who visited Institute of Rheumatology, Tokyo Women’s Medical University, those who fulfills thediagnostic criteria of PR by Gonzal-Lopez L, et al. and manifested IH were enrolled in this study. Polymorphisms of AID genes, clinical manifestations, findings of blood examinations and joint images, treatment responses, and outcome were evaluated retrospectively. Genomic DNA was isolated from the patients’ peripheral blood and a polymerase chain reaction (PCR) was used to amplify the indicated exons of 12 genes: MEFV (exons 1-10), TNFRSF1A (exons 2-4), MVK (exons 9-11), NLRP3 (exon 3-6), NOD2 (exon 4), LI1RN (exons 2-4), IL36RN (exons 2-5), PSMB8 (exons 2, 3, and 5), NALP12 (exons 3 and 9), PSTPIP1 (exons 10 and 11), TNFAIP3, and NLRC4. After cleaning the PCR products, cycle sequencing was carried out using the Big Dye® Terminator v3.1 kit and analyzed with an ABI 3130xl Prism Genetic Analyzer. Genetic polymorphisms within the above-mentioned genes were examined.

**Results:** Out of six patients (2 males and 4 females, median age: 47.1 years, median age at disease onset: 20 years, median disease duration:12 years), all manifested intermittent knee hydrathrosis lasting for several days with 1-4 weeks of attack intervals. Rheumatoid factor and anti-cyclic citrullinated peptides antibody were not detected in anyone. Knee joint X-ray showed no abnormality in all cases whereas increased synovial fluid and synovium tissue proliferation with positive vascularity by ultrasonography. Polymorphisms of AID genes were identified in all 7 cases as following; MEFV (n=4, all non-exon10), CIAS1(n=1) and TNFRSFIA (n=1). Regarding efficacy of medical treatments, although csDMARDs were not effective in all cases, colchicine applied to 4 with MEFV polymorphism was effective in all the four cases. TNF inhibitors was effective in one of two. During observation, no patients developed rheumatoid arthritis and one patient was complicated with Bechet’s disease.

**Conclusion:** Association of polymorphisms of the genes responsible for auto-inflammatory diseases should be considered in cases manifesting PR, especially IH, though systemic manifestations were not noted. Colchicine is promising medication in these conditions.

**Disclosure of Interest**

None Declared

#### P2005 Report of 65 patients with SAID in Argentina

##### Ileana Moreira, Analía G. Seminario, Agostina Llarens, Lina R. Riaño Cardozo, Lorena Regairaz, Liliana Bezrodnik

###### Immunología, Centro de Inmunología Clínica Dra. Bezrodnik, Buenos Aires, Argentina

####### **Correspondence:** Ileana Moreira

**Introduction:** Systemic auto-inflammatory diseases (SAID) can be defined as a group of immunodeficiencies marks by excessive inflammation that is predominantly mediated by increase response to known or unknown triggers by cells and molecules of the innate immune system. Most patients have recurrent systemic inflammation episodes with fever, acute phase reactants elevated and several clinical manifestations, with healthy intervals. SAIDs have, in most cases, a genetic background, with highly penetrant mutations of single genes, but some cases are polygenic with strong environmental influence that can modulate the phenotype.

**Objectives:** Report 65 Argentinian patients with different SAID.

**Methods:** Retrospective analysis of medical records of 65 patients with SAID.

**Results:** The patients have: Family mediterranean fever (FMF):22 patients (p), Hiper IgD Syndorme (HIDS): 1p. Familial Cold Autoinflammatory Syndrome (CAPS): 1p. Chronic atypical neutrophilic dermatitis with lipodystrophy (CANDLE) like: 1p. Syndrome of periodic fever, aphthous stomatitis, pharyngitis and cervical adenitis (PFAPA syndrome): 30p and 10p are unclassified.

Most of them are female and only 5/63 has a relative affected.

The median age of the first symptom was 2 yo (0-16 yo) and the median age at first immunology consult was 10 yo (0.1-48y).

The most frequent clinical manifestation was periodic fever and skin compromise. Other symptoms include: abdominal pain, arthralgia, aphthas, adenomegalies, pharyngitis, headache and chronic diarrhea.

During the fever episodes most of them had neutrophilia and elevated CRP and ESR.

Four patients have humoral compromise (2 with FMF and the ones with CAPS and Candle like).

Among the FMF, five patients have homozygous pathogenic variants and three have heterozygous compound variants. There are 14 patients with heterozygous variants, but all of them have periodic fever with several associated symptoms and also all of them present good response to oral colchicines treatment.

The CAPS patient has a heterozygous variant in NLRP12 and he is doing well with IVIG and oral budesonide. The patient with Candle-like syndrome has a poor response to steroids, anti-TNF and anti IL-1β. She has a homozygous variant in SAMD9L. After diagnosis, she received a full matched non related donor HSCT with full engraftment.

Among the patients with PFAPA syndrome, most of them have good response to oral steroids during crisis and half of them required also daily colchicine. All of them improve around 6 yo without new episodes.

None of patients have currently amyloidosis.

**Conclusion:** The symptoms between the different diseases can be similar, with predominant skin involvement, in addition to fever. All patients with heterozygous variants in MEFV gen were symptomatic and improved with the treatment. Only in 1 patient (SAMDL9) needed biological treatment, all the others had have good responses to colchicine or steroid treatment. There is a significant difference between the age of onset of the symptoms and the age of the first visit to the immunologist. We believe that the diagnosis is important because of potential implications for therapy, monitoring for the development of secondary amyloidosis, and the need for genetic counseling. Diffusion of SAID between pediatricians and clinicians would improve the age of diagnosis.

**Disclosure of Interest**

None Declared

#### P2006 Multisystemic inflammatory disease due to DNASE2 mutations: from physiopathology to new therapeutic approaches

##### Valentina Moressa^1^, Alessandra Tesser^2^, Andrea Trombetta^1^, Sergio Ghirardo^1^, Marco Bobbo^1^, Elisa Piscianz^1^, Flavio Faletra^2^, Stefano Volpi^3^, Alberto Tommasini^2^, Serena Pastore^2^, Andrea Taddio^2^

###### ^1^University of Trieste, Italy; ^2^IRCCS Burlo Garofolo, Trieste, Italy, Trieste; ^3^IRCCS Istituto G. Gaslini, Genova, Italy

####### **Correspondence:** Valentina Moressa

**Introduction:** We recently described the case of a boy with a novel interferonopathy due to DNase2 deficiency. He presented at birth a TORCH-like picture with hepatitis and cytopenias, self-healing in the following months. In his early years, he presented unexplained fever episodes, growth retardation, and poly-articular arthritis. In the subsequent years he developed lipodystrophy, skin rash and lupus pernio. The disease was refractory to several antirheumatic drugs and steroids. At the age of 15 years, DNase2 deficiency was diagnosed by exome analysis (1). Functional data showed that the disease is due to cGAS-dependent interferon inflammation, paving the way for improved treatments based on the inhibition of the hyperactive pathway.

**Objectives:** To describe how the diagnosis impacted on medical choices and overall clinical history of a patient.

**Methods:** Review of clinical sheets and analysis of interferon signature over the 4 years after genetic diagnosis.

**Results:** At the time of the genetic diagnosis, the patient complained of failure to thrive, severe poly-articular arthritis with impairment in walking ability, almost requiring a wheelchair assistance, and severe headache (NRS 9-10) requiring daily administration of major analgesics like tramadol. Treatment included: prednisone, Ramipril, lansoprazole, calcium, celecoxib, tramadole and abatacept.

After the diagnosis, a treatment with hydroxychloroquine and mepacrine was started, based on proofs of an inhibitory action of these drugs on cGAS (2). With such therapy, he was able to reduce the dosage of steroids and to improve motor capacity. To obtain a more complete control of the disease, ruxolitinib (JAK1/2 inhibitor) was added at the dosage of 10 mg, resulting in a rapid improvement of the headache and of the articular range of motion. In the subsequent six month, the lipodystrophy also showed some improvement, allowing further steroids tapering. However, he developed fatigue, tachycardia and peripheral cyanosis. Cardiologic assessment detected pulmonary arterial hypertension (PAH) and thus the boy started treatment with sildenafil, iloprost, and ambisentan.

Considering a possible adverse effect of ruxolitinib, as supported by a single case report (3), we interrupted all immunosuppressive therapies except for steroid boluses, but without any improvement. Moreover, he developed progressive cytopenia and increased interferon signature. Therefore he started again ruxolitinib at an higher dose, together with antimalarials, obtaining a marked drop of pulmonary pressure and an improvement in interferon signature, as described in published cases ofPAH (4). In few months he got well again, being able to taper steroids. Recently, ruxolitinib was switched to baricitinib, without any evident change in clinical efficacy.

**Conclusion:** The overall clinical improvement and the possibility of reducing the corticosteroid dosage suggest that treatment of our patient can be considered a precision, tailored therapy.

Nevertheless, due to the novelty and the rarity of such cases, hard is to disentangle between manifestations of the disease and the effects of treatments that are tailored to each patient without a consistent previous experience, as it is evident for orphan disease.

1. Rodero MP, et al. Nat Commun. 2017 Dec 19;8(1):2176.

2. An J, et al. The Journal of Immunology, 194(9), 4089–4093.

3. Low AT, et al. Haematologica, 100(6), e244–e245.

4. Sanchez GAM, et al. J Clin Invest. 2018 Jul 2;128(7):3041-3052.

**Consent for publication has been obtained from patient**

Yes

**Disclosure of Interest**

None Declared

#### P2007 Pregnancy and other gynecological aspects in mevalonate kinase deficiency

##### Catharina Mulders-Manders, Anna Simon

###### Radboud University Medical Center, Nijmegen, Netherlands

####### **Correspondence:** Catharina Mulders-Manders

**Introduction:** Mevalonate kinase deficiency (MKD) is one of the classic monogenic autoinflammatory diseases. Only two pregnancies in MKD patients, have been reported, both with favorable outcome. Little is known on the influence of MKD on pregnancy or other gynecological aspects.

**Objectives:** To gain further insight in pregnancy, lactation and gynaecological aspect in MKD.

**Methods:** All adult female MKD patients from the Radboud university medical center, a Dutch expertise center on MKD, were asked to fill out an online questionnaire regarding pregnancy, lactation, menses and anticonceptive use, and their influence on frequency, duration and symptoms of MKD attacks.

**Results:** *Pregnancy and lactation*

Of twenty adult female MKD patients, eleven responded to the questionnaire (55.0%). Median age was 28 years (range 21-55 years). Two patients were treated with anakinra (18.2%) and six with canakinumab (54.5%).

Four participants indicated that they had ever wanted to become pregnant, and all of them conceived spontaneously. One patient reported one pregnancy, and the other three all reported two pregnancies. Median age at first pregnancy was 27 years (range 24-30 years). During all pregnancies, patients remained attack-free without treatment. One of the four patients reported an MKD attack following delivery, and more frequent attacks in the first three months thereafter.

During pregnancy, one patient was treated because of pre-eclampsia during the first, and hypertension during her second pregnancy. Five other pregnancies were uncomplicated. All pregnancies resulted in birth of a healthy living child without congenital abnormalities.

One patient reported two periods of lactation in two children: one week in the first and 23 weeks in her second child. During lactation attack frequency was unchanged, but attacks were shorter.

*Other gynaecological aspects*

Four of the eleven patients reported more frequent MKD attacks after menarche. Two reported increased attack severity. In four patients, attacks were triggered by menstruation and during menstruation, two patients experienced longer attack duration. During the use of hormonal anticonceptive drugs, one out of eight patients experienced more frequent attacks, while four reported a decrease in frequency. Two patients reported shorter attack duration, while the other six did not notice any change in duration.

Two patients were in menopause, one of whom experienced more frequent attacks after menopause. One patient reported a shorter attack duration, while the duration increased in the other patient.

Five patients had ever experienced vaginal or vulvar ulcers.

**Conclusion:** During seven pregnancies in female MKD patients, no MKD attacks occured. In one pregnancy delivery triggered an MKD attack, and during the only episode of lactation, MKD attacks were shorter than usual. All seven children were born healthy. As our cohort size was small, it would be interesting to confirm these positive findings in a larger number of patients.

**Disclosure of Interest**

None Declared

#### P2008 Early onset sarcoidosis associated with NOD2 mutation: familial cases report

##### Roberta Naddei^1^, Francesca Orlando^1,2^, Carolina Porfito^1^, Teresa Lastella^1^, Marinabiondina Amico^1^, Maria Alessio^1^

###### ^1^Pediatric Rheumatology Unit, Mother and Child Department, University of Naples Federico II; ^2^Department of Pediatrics, Santobono-Pausilipon Children’s Hospital, Naples, Italy

####### **Correspondence:** Roberta Naddei

**Introduction:** Early onset sarcoidosis is characterized by a triad of polyarthritis, uveitis and rash. The familial cases, manifesting the classic clinical triad and an autosomal transmission pattern, have been termed Blau syndrome (BS). The mutation involved the NOD2 gene.

**Objectives:** To describe clinical features of a family with a history of uveitis, early onset arthritis and skin rash.

**Methods:** Case report.

**Results:** The propositus (patient 1) is a 7-year-old female referred to our Unit at the age of 2 years. The family history revealed a mother with rheumatoid arthritis and visual loss due to panuveitis. The clinical history disclosed that at the age of 12 and 15 months the patient had two episodes of diffused rash spontaneously resolved in two weeks. From the age of 18 months recurrent hip pain, defined as transient synovitis of the hip, was reported**.** It was treated successfully with non-steroideal antinflammatory drugs (NSAID) but pain would reappear once stopped the treatment. Clinical evaluation showed polyarticular symmetrical arthritis of wrists, elbows, knees, proximal interphalangeal joints. Polyarticular Juvenile Idiopathic Arthritis (JIA) diagnosis was made and treatment with NSAID and Methotrexate was started without significant improvement; therefore, Etanercept was added and NSAID stopped. Screening of uveitis was performed every six months. Persisting arthritis of right wrist after 7 months of therapy, ultrasound was performed showing tenosynovitis. Therefore, she underwent intra-articular injection of long-acting glucocorticoids with partial response. At the age of 4 years, she presented papuloerythematous rash, mainly on the trunk, infiltrated and confluent in plaques; skin biopsy was performed resulting consistent with granulomatous disease sarcoid-like. Suspecting BS, molecular analysis was done. At the age of 5 years, she presented scotoma and visual loss; the ocular assessment found bilateral anterior uveitis and posterior bilateral synechiae. She started systemic glucocorticoid therapy and shifted from Etanercept to Adalimumab with improvement of both ocular and articular disease. The molecular analysis of NOD2 showed missense mutation p.R334W (c.1000C<T) consistent with BS. Currently, the articular and cutaneous manifestations are controlled by the combined therapy with Adalimumab and Methotrexate; the ocular disease evolved into irido-lenticular synechiae, some mutton-fat keratic precipitates, band keropathy. She started topical treatment with improvement.

Her brother (patient 2, age 3 years) at the age of 34 months, developed boggy tenosynovitis and face papuloerythematous rash; inflammatory cellular deposits at anterior segment were disclosed at ophthalmic evaluation. Therefore, treatment with Adalimumab and Methotrexate was started.

The molecular analysis of NOD2 gene in patient 2, in a youngest asymptomatic brother and in the mother is ongoing.

**Conclusion:** Patient 1 presented typical clinical manifestations of pediatric sarcoidosis, confirmed by skin biopsy. R334W is the most common mutation reported in the international Blau registry. The presence of typical clinical manifestations, NOD2 mutation and family history led to the diagnosis of Blau Syndrome. Clinical surveillance and molecular analysis is essential in first-degree relatives of patients with Blau Syndrome in order to recognize clinical manifestations precociously and start appropriate therapy.

**Consent for publication has been obtained from patient**

Yes

**Disclosure of Interest**

None Declared

#### P2009 Extending the clinical spectrum of Blau syndrome, a case report with liver cirrhosis

##### Mohammed Nashawi^1^, Heiko Brennenstuhl^1^, Barbara Bangol^2^, Thomas Lutz^1^

###### ^1^Center for Pediatrics and Adolescent Medicine, University Hospital Heidelberg, Heidelberg; ^2^Genetic Department, Center for Human Genetics and Laboratory Diagnostics, Martinsried, Germany

####### **Correspondence:** Mohammed Nashawi

**Introduction:** Blau Syndrome is a rare auto-inflammatory disease with variants in NOD2 and an important differential diagnosis for periodic fever syndrome. Patients typically present in the first year of life with fever and a triad (skin manifestation, arthritis and uveitis). The patients could develop pan-uveitis, cardiac manifestation or even renal diseases.

**Objectives:** Our objective is to expand the differential diagnosis of periodic fever syndrome by characterizing a patient with atypical clinical features of Blau Syndrome harboring the most common known genetic variant.

**Methods:** We used standard laboratory assessment of inflammatory markers, MRI and liver biopsy. To confirm the diagnosis we used next generation sequencing to identify NOD2 variants.

**Results:** A 2-year-old male child presented to the hospital with recurrent fevers and decompensating heart failure due to arterial hypertension. He could not walk because of swelling in both ankles and additionally had swelling of the wrists. The past and the family history were unremarkable. On clinical examination, his weight and length were below the 3rd percentile. Skin examination showed erythematous exanthema on the trunk. There were also hepatosplenomegaly and a cardiac murmur present. At that time, he had been treated for heart failure and hypertension. After his medical condition was stable, further work-up had been done to look for the cause of his symptoms. CRP was massively increased. Infections and malignancy were excluded. There were no signs of immunodeficiency and no signs of uveitis. With the presented symptoms and signs the child was finally diagnosed with systemic juvenile idiopathic arthritis. Initially, he was started on Prednisolone 2 mg/kg/d. He started to get better. Methotrexate (MTX) had been also introduced and titrated until 15 mg s.c. once weekly. His symptoms improved under therapy, but he was still not in remission, with recurrent fevers, arthritis and high inflammatory markers. In this condition he was transferred to our center. Here, we started the treatment according to the following table:

However, there was no full remission, so whole body MRI was performed, which showed signs of liver cirrhosis, which was not explained by the disease and medications. Liver biopsy showed liver fibrosis with signs of inflammation and granulation formation. We did a molecular genetic analysis (fever panel) which showed a heterozygous mutation in NOD2 gene (c.1001G>A; p.Arg334Gln, Exon 4), which causes Blau Syndrome. Currently, he is responding well to the therapy with Adalimumab.

**Conclusion:** Here, we report a case of Blau Syndrome with liver cirrhosis extending the phenotypic spectrum of this rare disease. In our case the patient presented with heart failure, hypertension, arthritis, skin rash and also hepatomegaly. Episodic fever was noted. Our patient did not develop uveitis, under the treatment with MTX and the other medications which are all effective against uveitis. The recommended therapy for Blau Syndrome is to use steroid daily in addition to immunosuppressants. Different types of immunosuppressants have been used with a controversial effect, but TNF-alpha inhibitors have been shown to be most effective in treating patients with or without uveitis. Our patient showed partial response under the therapy with MTX, IL-1β antagonist and IL-6 antagonist. For the first time, under treatment with TNF-alpha inhibitor (specifically Adalimumab), he is in full clinical and laboratory remission.

**Consent for publication has been obtained from patient**

Yes

**Disclosure of Interest**

None Declared

**Table 1 (abstract P2009).** See text for description

	09/2012	01/2013	10/2015		10/2016	09/2017
Steroid	x	x	x		x	x
MTX	x	x	x		x	x
Canakinomab		x		x		
Tocilizumab			x			
Etanercept					x	
Adalimumab						x

#### P2010 Diagnosis and management of hereditary recurrent fevers: 20-year experience at a single Italian referral centre

##### Laura Obici^1^, Grazia Bossi^2^, Roberto Caporali^3^, Roberta Mussinelli^1^, Simona Casarini^1^, Sara Monti^3^, Isabella Ceccherini^4^, Francesco Caroli^4^, Antonio Vergori^2^, Eleonora Di Buduo^1^, Claudia Sforzini^1^, Marco Gattorno^5^, Giampaolo Merlini^1^

###### ^1^Amyloidosis Research and Treatment Centre; ^2^Pediatrics Unit; ^3^Rheumatology Unit, Fondazione IRCCS Policlinico San Matteo, Pavia; ^4^Medical Genetics Unit; ^5^Pediatric Rheumatology Unit, IRCCS Giannina Gaslini Institute, Genova, Italy

####### **Correspondence:** Laura Obici

**Introduction:** Disease awareness, diagnostic tools and therapeutic options for hereditary periodic fevers (HRF) have significantly improved in recent years but whether patients’ diagnostic pathway and outcome have consistently ameliorated remains to be elucidated.

**Objectives:** To investigate presentation, genotypes, clinical characteristics, natural history and response to treatment in patients with HRF referred to our centre between 1998 and 2018.

**Methods:** We conducted a retrospective study of all patients diagnosed with HRF according to genetics and/or clinical Eurofever classification criteria. All patients underwent genetic analysis and full clinical evaluation including investigation for possible amyloid disease.

**Results:** 213 patients (FMF 166, TRAPS 33, CAPS 5, MKD 9) were included, 10% of them are of non-Caucasian ancestry and 41% had a positive family history. Overall 61 genotypes were identified, with 3 novel gene variants, not reported to date. 87% of FMF patients had ³ 1 clearly pathogenic variant, the most common being p.Met694Val. 79% of TRAPS patients had a clearly pathogenic mutation, affecting a cysteine residue in 15 cases. Median age at disease onset was 12 years (range 0.5-51). Median age at diagnosis was 34 years (2-78). 79 % of patients presented in adult age, of which 35 (31 FMF and 4 TRAPS) reported symptom onset after the age of 20 (median 30, range 21-51). Median time from disease onset to diagnosis in patients referred in adult age was 25 years (0.5-61). Fever was the most common reported symptoms (in 96% of patients), followed by abdominal flares. Median number of inflammatory attacks was 8 in FMF (1-24). A chronic disease course was seen in 50% of TRAPS patients. AA amyloidosis was diagnosed in 22 patients (10.3%). Median age at AA onset was 40 years (range 12-77) with a median time from HRF onset to appearance of renal amyloidosis of 33.5 (8-56). In 19 cases (86%) the diagnosis of HRF was established after AA amyloidosis presentation. Regarding diagnostic delay, presenting features and rate of AA amyloidosis, no significant differences was observed throughout different time periods (1998-2004; 2005-2011; 2012-2018). In patients with FMF, complete response to colchicine is observed in 88%, with a median dose of 1 mg/day (range 0.5-2); partial response and/or limited tolerability was reported in 29 patients (22.7%). 7 pts were shifted to anti-IL1 agents, including two kidney transplanted patients due to AA amyloidosis, with complete response and good safety profile

**Conclusion:** Diagnostic delay hasn't significantly reduced over time in spite of increasing awareness and optimized diagnostic tools. A substantial proportion of patients is still recognized in adult age and therefore remain at high risk ofdeveloping long-term renal complications.

**Disclosure of Interest**

None Declared

#### P2011 RNF213 gene variants in patients with Takayasu arteritis or vasculopathy

##### Ebun Omoyinmi^1^, Annette Keylock^1^, Dara McCreary^1^, Sarka Fingerhutova^2^, Pavla Dolezalova^2^, Seza Ozen^3^, Sylvia Kamphuis^4^, Cheryl Hemingway^5^, Despina Eleftheriou^1^, Paul Brogan^1^

###### ^1^Infection, Inflammation and Rheumatology Section, UCL Great Ormond Street Institute of Child Health, London, United Kingdom; ^2^Paediatric Rheumatology and Autoinflammatory Diseases Unit, General University Hospital, praha, Czech Republic; ^3^Department of Paediatric Rheumatology, Hacettepe University, Ankara, Turkey; ^4^Department of Paediatric Rheumatology, Sophia Children's Hospital, Erasmus University Medical Centre, Rotterdam, Netherlands; ^5^Paediatric neuroradiology department, Great Ormond Street Hospital for Children NHS foundation Trust, London, United Kingdom

####### **Correspondence:** Ebun Omoyinmi

**Introduction:** *RNF213* (Ring Finger Protein 213) was first identified as a major susceptibility gene for moyamoya arteriopathy, which is a progressive steno-occlusive disease of the cerebral arteries and the development of abnormal basal collateral vessels. Several other studies have since reported an association of *RNF213* variants with various vasculopathies, pulmonary hypertension, coronary artery disease and stroke. *In vivo* experiments demonstrated abnormal development of brain vessels in *rnf213* knockdown zebrafish, while suppression of both arteriogenesis and angiogenesis were observed in knockout mice.Consequently, *RNF213* may not be just a susceptibility gene for moyamoya, but could also play a role in systemic vasculopathy, and be an important mimic of Takayasu arteritis (TA) of the young.

**Objectives:** To review the phenotype of vasculopathy/vasculitis in a cohort of patients referred for genetic testing where rare or novel variants in *RNF213* were identified.

**Methods:** *RNF213* variants were screened as part of a next-generation sequencing screening strategy for patients with suspected vasculitis or autoinflammation. Variants that were either novel or less than 1% in public genetic databases were considered as candidate for the purposes of this study.

**Results:** We identified 54/272 patients with at least one novel or rare *RNF213* variant. Fifteen/54 had vasculopathy or suspected vasculitis without clearly defined aetiology (Table). Amongst these cases, there were two children with TA, and a third case with familial moyamoya arteriopathy with 3 affected family members. The first case was a Turkish girl, with a novel heterozygous *RNF213* mutation p.L5003V; predicted to be damaging, who presented with hypertension at the age of 4 years, with vascular phenotype suggestive of TA, without cerebral involvement. The second TA case was a Dutch girl presenting with systemic inflammation and occlusive vasculopathy of multiple arterial beds, including renal and brain. She has 2 heterozygous variants in *RNF213*: p.P1721L and p.K4732E. The p.P1721L variant has previously been reported in cohort of patients with moyamoya arteriopathy, and is predicted to be damaging. The third case was a child from a family investigated for suspected familial moyamoya arteriopathy affecting 3 family members. The proband had a heterozygousp.D4013N *RNF213* missense mutation, previously reported in association with moyamoya. He had typical occlusive cerebral arteriopathy and collateral vessel formation. This mutation segregated with the same phenotype in the affected father and sister. Vasculopathies observed in the other cases are summarised in the Table.

**Conclusion:** These observations could support a role for mutations in *RNF213* as the cause of a monogenic but heterogeneous vasculopathy mimicking vasculitis such as TA, or stenotic vasculopathy associated with moyamoya arteriopathy.

**Disclosure of Interest**

None Declared

**Table 1 (abstract P2011).** *RNF213* variants in patients with Takayasu arteritis or vasculopathy

Patient ID	*RNF213* variants	Clinical phenotype
1	p.E803G	Behcet's disease/vasculitis
2-3	p.D1386N	Case 2; Severe congenital heart disease Case 3; Small vessel vasculitis with evolving autoimmunity
4	p.T1705K	lupus –like disease with progressive leukoencephalopathy, presumed small vessel brain disease
5	p.P1721L	Bilateral multifocal cerebral vasculopathy
6	p.P2905L	Stroke with right side focal lesion
7	p.T4155P	Ischaemic stroke
8	p.E4865K	Severe vasculopathy with multi-organ involvement including kidneys
9	p.T1705K p.I3318V	Left sided hemiplegia and progressive motor function deterioration with perivascular inflammation in brain biopsy
10	p.K4732E p.L4901F	Transient ischaemic attack (TIA)

#### P2012 Clinical and molecular features of 3 children with neonatal onset multisystem inflammatory disease: an experience from a setting where there is lack of access to anti-IL1 agents

##### Vignesh Pandiarajan^1^, Deepti Suri^2^, Sagar Bhattad^1^, Anju Gupta^1^, Amit Rawat^1^, Raphaela Goldbach-Mansky^3^, Marco Gattorno^4^, Surjit Singh^1^

###### ^1^Allergy Immunology Unit, Pediatrics; ^2^Postgraduate Institute of Medical Education and Research, Chandigarh, India; ^3^Translational Autoinflammatory Diseases Section, National Institute of Allergy and Infectious Diseases (NIAID), National Institutes of Health (NIH), Bethesda, United States; ^4^UO Pediatria II, Istituto G. Gaslini, Genoa, Italy

####### **Correspondence:** Vignesh Pandiarajan

**Introduction:** Neonatal-onset multisystem inflammatory disease (NOMID) is characterized by sterile inflammation of multiple organs due to molecular defect in *NLRP3*. Clinical manifestations usually start from early infancy and include fever, urticaria-like rash, arthropathy, and aseptic meningitis. Development of targeted therapies against interleukin-1 (IL1) has revolutionized the management of this clinical condition.

**Objectives:** To describe 3 cases of NOMID from a tertiary care centre in North India.

**Methods:** Case records of the patients followed up in our Pediatric Rheumatology Clinic were reviewed. Children with clinical features suggestive of of NOMID and proven molecular defect in *NLRP3* were included after informed consent from the parents.

The first child (P1) was symptomatic since early infancy in form of recurrent episodes of fever and urticaria-like rash. She developed frontal bossing and progressive arthropathy of knees over years, and the diagnosis was established at age of 13 years. Second child (P2) had swelling of knees and recurrent urticaria-like rash over trunk since early infancy. He developed sensorineural hearing loss, progressive enlargement of knees and contractures of both hips and knees. He developed renal amyloidosis and obstructive hydrocephalus at the age of 11 years. Radiograph of knees showed metaphyseal cupping, epiphyseal enlargement with sclerosis and prominent patella. Third child (P3) developed nephrotic syndrome at the age of 6.5 years. She received multiple immunosuppressive medications for her treatment- cyclophosphamide, tacrolimus, and mycophenolate mofetil. Renal biopsy done at the age of 10 years revealed AA amyloidosis. She had occasional urticarial rash in early infancy which went unnoticed. All three children had persistent neutrophilic leukocytosis, thrombocytosis, elevation in erythrocyte sedimentation rate (ESR), and C-reactive protein (CRP).

**Results:** Thalidomide (3 mg/kg) produced significant reduction in inflammatory burden of the first child (P1). She is now 16 years old and doing well. The second child (P2) was started on thalidomide since 4 years of age, however, no response was noted. Second (P2) and third child (P3) succumbed to renal failure secondary to amyloidosis, at the age of 13 and 11 years, respectively. Molecular defects identified in *NLRP3* in children P1, P2, and P3 were p.Asp305His, p.Thr349Ile, and p.Ala352Val, respectively.

**Conclusion:** Delay in diagnosis, lack of awareness, and lack of access to anti-IL1 agents contributed to significant morbidity and mortality in our patients. Thalidomide can be tried to reduce the inflammatory burden in patients with NOMID in settings where there is no access to anti-IL1 agents

**Consent for publication has been obtained from patient**

Yes

**Disclosure of Interest**

None Declared

#### P2013 Long-term outcomes and treatment efficacy in patients with TNF receptor-associated autoinflammatory syndrome (TRAPS): a series of 290 cases from the Eurofever/EUROTRAPS international registry

##### Riccardo Papa^1^, Thirusha Lane^2^, Taryn Youngstein^2^, Tamer Rezk^2^, Charalampia Papadopoulou^3^, Nicolino Ruperto^1^, Paul A. Brogan^3^, Philip N. Hawkins^2^, Patricia Woo^3^, Marco Gattorno^1^, Helen J. Lachmann^2^

###### ^1^Paediatric Rheumatology Clinic, IRCCS Giannina Gaslini Institute, GENOVA, Italy; ^2^National Amyloidosis Centre, Division of Medicine, Royal Free Campus, University College London; ^3^Department of Infection, Inflammation and Rheumatology, UCL Great Ormond Street Institute of Child Health, London, United Kingdom

####### **Correspondence:** Riccardo Papa

**Introduction:** Tumour necrosis factor (TNF) receptor-associated periodic syndrome (TRAPS) is one of the best known monogenic auto-inflammatory disorder resulting from an autosomal dominant variation in the TNF super family receptor 1A (*TNFRSF1A*) gene.

**Objectives:** To define best treatment approach in patients with TRAPS and effect on long-term outcomes.

**Methods:** We reviewed all data on patients with *TNFRSF1A* variants enrolled in the Eurofever/EUROTRAPS international registry.

**Results:** Data on 290 patients were available. Patients with R92Q, P46L or intronic variants (49%) displayed milder disease than 147 patients with mutations affecting other coding regions, with less frequent abdominal pain and skin rashes (P<0.01), higher efficacy rate of colchicine as maintenance treatment, and none developed AA amyloidosis. Almost 90% of patients with exon mutations required maintenance therapy. Anti-interleukin (IL) 1β drugs were the most frequently used (47 patients), with the highest efficacy rate (>90% complete response), while Etanercept was less effectively used and discontinued in 72% of patients. No patients on anti-IL1β treatment developed amyloidosis and 10 patients with amyloidosis have been successfully treated with anti IL-1 agents with preservation of native renal function in 7 and excellent long-term transplant function in 2. Nine women had a history of failure to conceive and seven had successful pregnancies without fertility treatment following complete disease control with anti-IL1β drugs. Long term safety profiles for anti IL-1 agents were excellent even in the presence of comorbidity.

**Conclusion:** Anti-IL1β drugs are the best maintenance treatment in TRAPS with potential to reverse the most serious disease complications of AA amyloidosis and infertility. The diagnosis of TRAPS should be considered very carefully in patients carrying R92Q, P46L or intronic *TNFRSF1A* variants.

**Disclosure of Interest**

None Declared

#### P2014 A child with Majeed syndrome from India

##### Pallavi Pimpale Chavan, Raju P. Khubchandani

###### Pediatric Rheumatology Clinic,Department of Pediatrics, Jaslok Hospital and Research Centre, Mumbai, India

####### **Correspondence:** Pallavi Pimpale Chavan

**Introduction:** Majeed syndrome is a very rare autosomal recessive, autoinflammatory disorder characterized by a triad of chronic recurrent multifocal osteomyelitis, congenital dyserythropoietic anemia and neutrophilic dermatosis caused by LPIN2 gene mutation. 14 mutation positive cases identified till 2017 have been reported from the Middle East.

**Objectives:** We describe our brief experience with the second family with a child afflicted with Majeed syndrome from India.

**Methods:** A 4 year old male child born of a consanguineous marriage, presented with recurrent irregular episodes of fever with irritability and failure to thrive since 5 months of age. At 9 months of age parents noticed, small skin ‘boils’ over the trunk and limbs, which recurred every 15-20 days along with the fever and stayed for 4-5 days. At 18 months, parents noticed that he refused to bear weight and walk and would cry on handling.

Gradually the episodes of painful small skin boils, limb pains which the parents could not localize and fever increased in frequency and after multiple pediatric, orthopaedic consults he was referred to us by a pediatric neurologist who had been referred the case to rule out Fabry disease. The pediatric neurologist had asked for whole exome sequencing and referred the case to us for clinical evaluation sensing a musculoskeletal rather than neurological disease.

**Results:** On examination the weight (10 kg) and height (83 cm) were below 3^rd^ centile. He had pallor, painful swelling over the left tibia, motor delay with hamstring tightness and mild bilateral knee contractures. Language and social milestones were normal. There were no skin lesions at the time of evaluation and systemic examination was normal.

Review of his past investigations revealed a persistent, microcytic hypochromic anemia (Hemoglobin 8.3-10.6 gm/dl) with raised erythrocyte sedimentation rate (54-105 mm/hr), C-reactive protein (37-98 mg/l) and platelets (4.3-8.9 x10^3^ cu mm).

His genetic mutation for LPIN 2 returned positive with homozygous missense variation in exon 17 of the LPIN2 gene resulting in amino acid substitution of cysteine to arginine at codon 736 (p.Arg736Cys;ENST00000261596). Meanwhile a bone scintigraphy done, showed increased vascularity and osseous activity in proximal shaft of left tibia with mild osseous activity at D9 and D10 vertebrae. A bone marrow examination was not performed as a conclusive mutation diagnosis was obtained.

He showed a dramatic response to Ibuprofen with abatement of fever, no skin boils and improvement of general mood and playfulness. With a view to attempt to omit NSAIDs, intravenous Pamidronate was commenced. He has received four doses of Pamidronate (cumulative dose 12 mg/kg) over one year and has gained weight (11.5kg) and height (96cm), remained afebrile with full physical activity and no new skin lesions.

**Conclusion:** Paucity of awareness amongst the medical community about these rare illnesses are responsible for diagnostic delays and complex referral pathways. With the high rates of consanguinity in several parts of our country we believe that we are seeing just the tip of the iceberg of monogenic autoinflammatory diseases. Due to non availability of Interleukin-1 inhibitors in India, which are highly effective at controlling osseous and systemic inflammation in Majeed syndrome , we have limited treatment options to offer to our patient. We have offered educational resources and genetic counselling to the family.

**Consent for publication has been obtained from patient**

Yes

**Disclosure of Interest**

None Declared

#### P2015 Spondyloenchondrodysplasia – a case from India

##### Pallavi Pimpale Chavan^1^, M Moreews^2^, Shashi Ranade^3^, A Belot^2^, Raju P. Khubchandani^1^

###### ^1^Section of Pediatric Rheumatology, Jaslok Hospital and Research Centre, Mumbai, India; ^2^Pediatric Rheumatology Clinic, National Referee centre RAISE (Rhumatism and AutoImmunity in children) & CIRI, INSERM U1111, Lyon, France, ^3^Consultant Paediatrician, Ranade Hospital, Karad, India

####### **Correspondence:** Pallavi Pimpale Chavan

**Introduction:** Spondyloenchondrodysplasia (SPENCD) is a very rare genetic immuno-osseous dysplasia characterized by skeletal anomalies (short stature, platyspondyly) and enchondromas in the long bones or pelvis. SPENCD may have a heterogeneous clinical spectrum with neurological involvement or autoimmune manifestations, such as systemic lupus erythematosus (SLE).

**Objectives:** We describe a child from India with SPENCD, who presented with features of SLE , skeletal dysplasia and enchondromas

**Methods:** A 9 year old male born of second degree consanguineous marriage presented with fever , oral ulcers, leg pain with morning stiffness since 1 month. He was worked up by his primary paediatrician for pyrexia of unknown origin and a summary of laboratory tests performed included, hemogram with hemoglobin 8.7 gm/dl, white cell count 5900 /cu mm platelet 1.85 x 10^3^ cu mm, erythrocyte sedimentation rate 100 mm/hr, antinuclear antibody 4+.Suspecting connective tissue disease child was referred to us for further evaluation.

**Results:** On further enquiry, there was past history of multiple medical visits for poor growth since infancy and a bony swelling of the right forearm leading to incomplete supination for which he was operated at age 5 years. No details were available. His milestones were appropriate for age with normal scholastic performance.His weight (16.9 kg) and height (113cm) were below the 3rd centile. On examination, he had pallor, facial rash, mucositis of the palate, generalised lymphadenopathy, triangular facies, prominent anteverted ears, prominent conjunctival blood vessels, supernumerary teeth, right elbow swelling, paucity of body hair On systemic examination abdomen was soft with hepatosplenomegaly and other systems were normal.

On further investigation his dsDNA was 4+ , Direct Coombs test was positive, C3 - 19.1 mg/dl C4 - 3.36 mg/dl, urinalysis was normal, Anti Ro and lupus anticoagulant were negative and APLA IgG was positive and APLA IgM negative. The diagnosis of SLE was confirmed.

Considering, consanguineous marriage, short stature and right elbow swelling with other subtle dysmorphisms described above, a syndromic form of lupus was suspected and a skeletal Xray analysis showed extensive areas of metaphyseal abnormalities with epiphyseal remodelling changes and platyspondyly. Hence a clinical diagnosis of Spondyloenchrondrodysplasia (SPENCD) was considered. Sanger sequencing of *ACP5* encoding the protein TRAP revealed homozygous non sense mutation (c.849T>A, p.(Tyr283stop) confirming the diagnosis of SPENCD.He was further investigated for diseases associated with SPENCD, which revealed normal thyroid function, IGF – 1, immunoglobulin levels and 2DECHO.

He was started on hydroxychloroquine (5mg/kg/day), oral prednisolone (0.6mg/kg/day), low dose aspirin, supplements and was advised sun protection. On follow up after a year, he is continued on hydroxychloroquine (5mg/kg/day), oral prednisolone (0.25mg/kg/day), low dose aspirin and supplements. He has gained weight (20.6 kg), height (114 cm), is afebrile with no arthritis, mucositis or rash.

**Conclusion:** SPENCD is an immunosseous dysplasia and a very rare cause of monogenic lupus. This, is the second case of SPENCD reported from India. The entity should be suspected in children with SLE with a consanguineous background who have short stature and bony abnormalities. We expand the phenotype here with additional dysmorphic features comprising supernumerary teeth, triangular facies, prominent anteverted ears and prominent conjunctival blood vessels not described before to the best of our knowledge.

**Consent for publication has been obtained from patient**

Yes

**Disclosure of Interest**

None Declared

#### P2016 Clinical profile of seven children with hereditary complement deficiency from a center in Mumbai, India

##### Prajakta Ranade^1^, Y.J. Crow^2^, Pallavi Pimpale Chavan^3^, L Seabra^4^, G Rice^5^, Raju P. Khubchandani^3^

###### ^1^Paediatrics, St John's Medical College Hospital, Mumbai, India; ^2^Centre for Genomic And Experimental Medicine, Institute of Genetics and Molecular Medicine, Edinburg, United Kingdom; ^3^Section of Pediatric Rheumatology, Jaslok Hospital and Research Centre, Mumbai, India; ^4^Neurogenetics and Neuroinflammation, Laboratory of Neurogenetics and Neuroinflammation, Paris, France; ^5^Division of Evolution and Genomic Science, Manchester Academic Health Science Centre, Manchester, United Kingdom

####### **Correspondence:** Pallavi Pimpale Chavan

**Introduction:** While systemic lupus erythematosus (SLE) is classically considered as a multifactorial polygenic disease, hereditary monogenic forms are well recognized. Deficiencies in the early components of the classical pathway strongly predispose to the development of SLE, of which C1q deficiency is the commonest. Children with C1q deficiency are estimated to develop SLE in 93% of cases. We report our experience with this entity.

**Objectives:** To describe the clinical and laboratory profile and follow up of a cohort of seven children with hereditary complement deficiency.

**Methods:** We reviewed our data on seven children with hereditary complement deficiency and obtained a genetic diagnosis in 5 of these 7 patients using next-generation sequencing. Informed consent was taken from the parents and the data was tabulated in the study proforma.

**Table:** 

Serial no/Sex	CSM	Onset (m)/time to diagnosis (m)	Clinical	Positive antibody profile	C3/C4/CH50	Genetics	Treatment	Follow up (m)/Outcome
1/F	N	10/7	Rash, mucositis, neuroregression	ANA Ds DNA Anti Ro	175/23/42.1	Homozygous for a c.606delA / p.Gly204Alafs*78 variant in C1QA	HCQ Sx AZT	28/Improved
2/F	Y	24/24	Rash, mucositis, arthritis, renal disease, herpes zoster	ANA Anti Ro	203/73/54	Homozygous for a c.622C>T / p.Q208* STOP variant in C1QA	HCQ,Sx,AZT,MMF	Death (at 12 years of age)
3/M	Y	21/12	Rash, mucositis, delayed development, hyperreflexia	ANA Ds DNA Anti Ro ACLA IgG	158/47.7/43	Homozygous for a c.622C>T / p.Q208* STOP variant in C1QA	HCQ,Sx,AZT	16/Improved
4/M	Y	12/24	Rash, mucositis, hyperreflexia, seizures	ANA Anti Ro	117/39/18.6	Homozygous for a c.171delT / p.Gly58Alafs*224 variant in C1QA	HCQ,Sx,AZT,MMF	41/Improved
5/F	Y	27/4	Rash, mucositis, neuroregression, hemiparesis, cerebral atrophy, hyperreflexia, hypothyroidism	ANA Anti Ro ACLA IgM Anti TPO	179/42.8/NA	Homozygous for a c.79C>T / p.Arg27* variant in C1QA	HCQ,Sx,Cyc,AZT	31/Improved
6/M	Y	40/0.5	Rash’ mucositis	ANA ACLA IgM	114/NA/NA	Afflicted sibling of Sr no 2 Not tested	HCQ,Sx	22/Improved
7/F	Y	48/36	Rash, mucositis, arthritis, proteinuria, infections (varicella, scabies, impetigo), hypothyroidism	ANA	30/NA/35.7	Demise before testing	HCQ, Sx, Cyc MMF	Death (at 12 years of age)

Abbreviations: F: Female, M: Male, CSM: Consanguinity, Y: Yes, N: No, m: months, Sx: Steroids, AZT: Azathioprine, Cyc:Cyclophosphamide, MMF: Mycophenolate

**Results:**

**Conclusion:** Based on our study and others reported, hereditary complement deficiency (notably C1Q deficiency) is the commonest form of monogenic lupus in our part of the world. Afflicted children present most commonly with rash and mucositis below 5 years of age and there is a significant delay in diagnosis due to lack of physician awareness. Neurologic features are frequently seen while renal manifestations are not. Neuroregression responds to immunosuppressive therapy with children improving on their mental, motor and speech milestones. Except in one patient major life-threatening infections have not been a challenge despite high infection rates in the community coupled with immunosuppression.

**Disclosure of Interest**

None Declared

#### P2017 Preliminary results of the Latin-American cohort of auto-inflammatory syndromes

##### Daniela G. P. Piotto^1^, Clovis A. Silva^2^, Katia Kozu^2^, Ana Luiza Cunha^3^, Flavia Patricia Santos^4^, Ana Laura Tolin^5^, Sheila K. Oliveira^6^, Simone Appenzeller^7^, Claudia S. Magalhães^8^, Marcia Bandeira^9^, Flavio Sztajnbok^10^, Carlos NobreRabelo Jr^11^, Carmen Laurade Cunto^12^, Cristina De Battagliotti^13^, Blanca Bica^14^, P Munittis^15^, Liliana Bezrodnik^16^, Marta F. Rodrigues^17^, Tereza C. Robazzi^18^, Erica N. Matos^19^, Ricardo Russo^20^, Maria Teresa Terreri^1^

###### ^1^Division of Rheumatology, Department of Pediatrics, Universidade Federal de Sao Paulo; ^2^Division of Rheumatology, Department of Pediatrics, Faculdade de Medicina da Universidade de São Paulo (FMUSP), São Paulo; ^3^Division of Rheumatology, Department of Pediatrics, Hospital Infantil João Paulo II, Belo Horizonte; ^4^Division of Rheumatology, Department of Pediatrics, Universidade Federal de Minas Gerais, São Paulo, Brazil; ^5^Division of Rheumatology and Unit of Pediatric Rheumatology - Department of Pediatrics, Hospital Notti, Mendoza, Argentina; ^6^Division of Rheumatology, Department of Pediatrics, Federal University of Rio de Janeiro, Rio de Janeiro; ^7^Rheumatology Department, Faculty of Medical Sciences, UNICAMP, Campinas; ^8^Division of Rheumatology, Department of Pediatrics, Universidade Estadual de São Paulo - Faculdade de Medicina de Botucatu, Botucatu; ^9^Division of Rheumatology, Department of Pediatrics, Hospital Pequeno Principe, Curitiba; ^10^Division of Rheumatology, Department of Pediatrics, Hospital Universitário Pedro Ernesto, Rio de Janeiro; ^11^Division of Rheumatology and Unit of Pediatric Rheumatology - Department of Pediatrics, Universidade Federal de Fortaleza, Fortaleza, Brazil; ^12^Division of Rheumatology, Department of Pediatrics, Hospital Italiano, Buenos Aires; ^13^Division of Rheumatology, Department of Pediatrics, Hospital de niños Dr Orlando Alassia, Santa Fe, Argentina; ^14^Division of Rheumatology, Department of Pediatrics, Hospital Universitário Clementino Fraga FilhoUniversidade Federal do Rio de Janeiro, Rio de Janeiro, Brazil; ^15^Division of Rheumatology and Unit of Pediatric Rheumatology - Department of Pediatrics, Hospital El Cruce, F. Varela; ^16^Servicio Inmunología HNRG., Hospital de Niños, Buenos Aires, Argentina; ^17^Division of Rheumatology and Unit of Pediatric Rheumatology - Department of Pediatrics, Universidade Federal do Rio de Janeiro, Rio de Janeiro; ^18^Division of Rheumatology and Unit of Pediatric Rheumatology - Department of Pediatrics,Universidade Federal da Bahia, Bahia; ^19^Division of Rheumatology, Department of Pediatrics,Hospital Universitário da Faculdade de Medicina da Universidade Federal de Mato Grosso do Sul, Mato Grosso do Sul, Brazil; ^20^Division of Rheumatology, Department of Pediatrics, Hospital Garrahan, Buenos Aires, Argentina

####### **Correspondence:** Daniela G. P. Piotto

**Introduction:** Auto-inflammatory syndromes (AIS) are rare and with large spectrum of clinical, genetical and laboratorial findings, which vary according to ethnic and geographical factors.

**Objectives:** There is no report evaluating AIS solely in Latin–American (LA) cohorts. Therefore, the objective of this study is to characterize AIS in a LA population.

**Methods:** Fourteen countries from all over Latin America were invited to participate in this observational and descriptive study collecting data on clinical, laboratory, genetic characteristics and treatment response of polygenic and monogenic AIS. Inclusion criteria were patients with clinical and/or genetic diagnosis of AIS. Data were prospectively collected in a web-based data bank.

**Results:** At the time of this report, 162 patients from Brazil (78.8%) and Argentina (21.2%) had been included: 52% males, 75% Caucasians; median age at symptoms onset was 2.1 years, median age at diagnosis of 6.9 years, and median disease duration was 8.5 years. The main AIS diagnosis were: PFAPA (28%), CNO (18%), FMF (16%), PGA (10%), CAPS (9%), HIDS (7%), TRAPS (5%) and PAPA (3%).The diagnosis was made clinically in 62% of patients (either they were PFAPA or CNO patients or presented a negative genetic test); 33% of patients were diagnosed by both clinical features and genetic tests and 5% of patients had no clinical but only a positive genetic test that was performed due to family history. Clinical features were consistent with the genetic abnormalities found. Genetic testing had been done in different laboratories using either Sanger sequencing and/or NGS. One of both acute phase reactants (either ESR or CRP) elevation occurred in 91% patients. The most frequently used medication were corticosteroids (66%, partial response in more than a half of patients) and colchicine (29.6%, total or partial response in half of patients). Regarding biological agents, etanercept was used in 11.1% with a partial response in most patients. IL1 blockers were prescribed in 11.1% but with a total response in the majority who used them. Of note, 51% of patients achieved remission, but 46% were still active at the time of evaluation, 2% had deceased, MAS was observed in 1% and none of them had amyloidosis.

**Conclusion:** A Latin-American registry of AIS included patients especially from South America. A clear limitation in access to genetic diagnosis was noted. Recognizing the barriers to the awareness about expansion of this group is a priority to better understand these complex diseases, especially in Central America. Some features like absence of amyloidosis and medications used in treatment differentiate our cohort from American and European ones.

**Disclosure of Interest**

None Declared

#### P2018 Chronic nonbacterial osteomyelitis: report of six cases

##### Daniela G. P. Piotto^1^, André Aihara^2^, Artur Fernandes^2^, Gabriela Balbi^1^, Claudio Len^1^, Maria Teresa Terreri^1^

###### ^1^Division of Rheumatology and Unit of Pediatric Rheumatology - Department of Pediatrics; ^2^Department of Imaging Diagnosis, Universidade Federal de Sao Paulo, São Paulo, Brazil

####### **Correspondence:** Daniela G. P. Piotto

**Introduction:** Chronic nonbacterial osteomyelitis (CNO), also known as chronic recurrent multifocal osteomyelitis (CRMO), is a rare autoinflammatory bone disorder that causes multifocal aseptic lytic lesions in bone biopsy and is characterized by periodic exacerbations and remissions, mostly affecting children and adolescents.

**Objectives:** To report and describe clinical features, laboratory and treatment of six cases of CNO.

**Methods:** Retrospective descriptive review of six children and adolescents with CNO treated at a specialized center, between 2010 and 2018.

**Results:** Four (66%) out of 6 patients were girls with mean age of 15.5 years (range 11-20.4) and mean follow-up time of 5.1 years (range 1.5-10.2). Mean age of first symptoms was 10.3 years (range 0.7–15.3) and mean age at diagnosis was 12.8 years (range 9.5-16). The most affected sites were metaphysis and diaphysis of long bones. Median number of initial bony lesions was 2.8 (range 1-7) at onset and 3.5 (range 1-8) during the follow up. Two (33.3%) patients had recurrence in clavicle, one (16.6%) in temporomandibular joint and two in mandible. One (16.6%) patient presented with fever and dyserythropoietic anemia, receiving diagnosis of Majeed syndrome.Five (83.3%) patients presented with arthralgia and bone pain, and two (33.3%) of them had acute migratory polyarthritis. All patients had increased acute phase reactants and radiographic and magnetic resonance imaging alterations.All patients received NSAIDs therapy (indomethacin) with clinical remission in half of the cases.Three (50%) patients received bisphosphonates (alendronate), and one took methotrexate associated with alendronate. All patients had good clinical response and two achieved clinical remission.

**Conclusion:** The awareness of characteristic features of CNO is important for an early diagnosis and can help avoiding unnecessary diagnostic procedures and prolonged antibiotic therapy.

**Disclosure of Interest**

None Declared

#### P2019 Familial Mediterranean fever: strong evidence for a functional effect of E148Q when combined with M694V

##### Elon Pras^1^, Ori Eyal^1^, Yael Shinar^2^, Mordechai Pras^3^

###### ^1^Institute of Human Genetics; ^2^FMF Clinic; ^3^Heller Institute of Medical Science, Sheba Medical Center, Ramat Gan, Israel

####### **Correspondence:** Elon Pras

**Introduction:** The role of E148Q in the pathogenesis of familial Mediterranean fever (FMF) is controversial. The penetrance of the M69V/M694V genotype in adult FMF patients is close to 100%. Disease penetrance in patients with the M694V/E148Q and M694V/null (single M694V mutation) genotypes is unknown. A difference in the penetrance of the latter two may indicate functionality for E148Q.

**Objectives:** To assess the penetrance of the M694V/E148Q and M694V/null genotypes in FMF

**Methods:** The study was performed on North African Jewish (NAJ) population in which FMF is highly prevalent and mutation frequencies are well known. Combined carrier rates for M694V and E148Q were calculated from three previous studies. The expected frequencies of the M69V/M694V, M694V/E148Q and M694V/null genotypes were calculated. We constructed a cohort of 107 consecutive patients with definite FMF, all of NAJ decent who came for genetic analysis. The ratio between the calculated frequencies of the 3 genotypes and the actual frequency obtained from the patient cohort was used to determine the penetrance of M694V/E148Q and M694V/null.

**Results:** In the patient cohort we found 75 patients with the M694V/M694V genotype, 15 with the M694V/E148Q an 16 with M694V/null. Pooled allele frequencies from 443 NAJ controls were 0.066 and 0.047 for M694V and E148Q, respectively.Considering 100% penetrance for adult FMF patients homozygous for M694V, calculations show a penetrance of 0.1405 and 0.00756 for M694V/E148Q and M694V/null, respectively.

**Conclusion:** The penetrance of M694V/E148Q is more than 18 times higher than M694V/null indicating an active role for E148Q when combined with M694V.

**Disclosure of Interest**

None Declared

#### P2020 Treatment of type 1 interferonopathy with Ciclosporin A and baricitinib in a 5 year old boy with heterozygous PSMB-8 mutation

##### Christoph Rietschel^1^, Eduardo Salamano^1^, Min Ae Lee-Kirsch^2^, Kay Latta^3^

###### ^1^Pediatric Rheumatology, Clementine Kinderhospital, Frankfurt/Main; ^2^Molecular Pediatrics, Klinik für Kinder- und Jugendmedizin, Universitätsklinikum Carl Gustav Carus, Dresden; ^3^General Pediatrics, Clementine Kinderhospital, Frankfurt/Main, Germany

####### **Correspondence:** Christoph Rietschel

**Introduction:** Type 1 Interferonopathy is a challenging autoinflammatory disease that can mimic different systemic diseases such as systemic Juvenile Idiopathic Arthritis. It shows a considerable clinical heterogeneity with neurological, cutaneous, arthritic and other symptoms and is commonly transmitted in an autosomal recessive manner. Different mutations have been discovered in recent years. Exceptionally, only one mutation can be found. The activation pattern of genes involved in the interferon pathway, the interferon signature, is the diagnostic hallmarkof the disease and provides an important tool for monitoring disease activity.

**Objectives:** We present this interesting case to show the clinical spectrum, diagnostic workup, possible complications and the chosen treatment modalities in this particular disease.

**Methods:** Our patient, born 03/2013, with an uneventful previous history, developed first symptoms in 09/2016. He showed persistent fever and a transient rash. From the beginning he suffered from strong, unexplained abdominal pain. His CRP values and leukocytes were only moderately elevated. He was first treated in another hospital for infection, but did not respond to broad spectrum antibiotics. He was transferred for further rheumtologic evaluation. Initial blood testing yielded only slightly elevated CrP levels but high ferritine >2000ug/l. Bone marrow aspiration showed strong granulopoiesis, no malignant infiltration or MAS. Ultrasound revealed hepatosplenomegaly, moderate serositis and cervical lymphadenopathy. Due to ongoing doubt on the diagnosis of SJIA a second bone marrow aspirate, lymph node and skin biopsy were performed, which were nonspecific. In the course of the disease the general condition deteriorated,CrP levels rose to >200mg/l and ferritine to >12.000ug/l. Liver enzymes were elevated, no cytopenia was seen. We assumed that our patient was at constant risk of full-blown MAS.

**Results:** Treatment with Anakinra was started at 2mg/kg/d and raised to 4mg/kg/d but was not effective. High dose methylprednisolonetherapy showed good clinical and laboratory response. On tapering of corticosteroids (CS) however, a rapid relapse resulted and the patient was put on tocilizumab 12mg/kg/2w and ultimately canakinumab 4mg/kg/4w, both of which were not effective. Ciclosporin A (CSA) 5mg/kg/d was added 01/17 due to incomplete control of inflammation and persistent risk of MAS. Further laboratory evaluation was obtained. IL-18 levels were above the detectable level of 20.000pg/ml. Panel diagnostic for autoinflammatory diseases showed a heterozygous mutation of unknown relevance in the PSMB-8 gene, a gene hosting several known mutations responsible for interferonopathies. Interferon signature in our patient showed a very high score, ultimately leading to the hypothesis of type 1 interfonopathy. We started a treatment with baricitinib (BAR) 05/17 and continued CSA and CS. The patient showed a rapid response, BAR was continuously raised to a dose of 4x2mg/day with complete remission. CS were tapered carefully and stopped 08/17. Ultimately CSA was tapered and stopped 09/17. Six weeks after discontinuation of CSA while on monotherapy with BAR 4x2mg/kg, a moderate clinical relapse occured, the interferon signature showing an extremely high score. Thus CSA was restarted and a course of CS was necessary to induce remission. Since 01/17 our patient is off CS and shows a complete remision with excellent tolerance of the tretment with BAR+CSA.

**Conclusion:** Treatment with Janus kinase inhibitor BAR was a breakthrough in the treatment of our patient, however, a combination with CSA was necessary to preserve complete remission.

**Consent for publication has been obtained from patient**

Yes

**Disclosure of Interest**

None Declared

#### P2021 Protein prenylation is defective in mevalonate kinase deficiency and is an accurate biomarker in peripheral blood mononuclear cells

##### Michael Rogers^1^, Julie Jurczyluk^1^, Oliver Skinner^1^, Anna Simon^2^, Sam Mehr^3^, Pravin Hissaria^4^, Rob Arts^5^, David Coman^6^, Marcia Munoz^1^

###### ^1^Bone Biology Division, Garvan Institute of Medical Research, Darlinghurst, Sydney, Australia; ^2^Department of Internal Medicine, Radboud University Medical Center, Nijmegen, Netherlands; ^3^Department of Allergy/Immunology, Royal Children's Hospital, Melbourne; ^4^Department of Clinical Immunology, Royal Adelaide Hospital, Adelaide, Australia; ^5^Department of Internal Medicine, Radboud University Medical Center, Nijmegen, Netherlands; ^6^Department of Metabolic Medicine, Queensland Children's Hospital, Brisbane, Australia

####### **Correspondence:** Michael Rogers

**Introduction:** Mevalonate kinase deficiency (MKD, which includes HIDS and mevalonic aciduria) is caused by recessive, hypomorphic mutations in the *MVK* gene encoding mevalonate kinase, a crucial enzyme of the mevalonate pathway. This biochemical pathway is required for the synthesis of isoprenoid lipids that are essential for the post-translational prenylation of proteins, particularly small GTPases such as Rab proteins and Rap1A. Blockade of the mevalonate pathway prevents the synthesis of isoprenoid lipids and results in the cytosolic accumulation of small GTPase proteins in their unprenylated form. It has long been assumed that mutations in *MVK* lead to defective protein prenylation in patients with MKD, but direct evidence for the accumulation of unprenylated proteins in patients’ cells has been lacking.

**Objectives:** To address the question whether protein prenylation is defective in MKD patients’ peripheral blood mononuclear cells (PBMCs), we optimized an *in vitro* prenylation assay that is capable of detecting very low levels of unprenylated Rab proteins in cell lysates and compared it to a western blot approach that specifically recognises the unprenylated form of Rap1A.

**Methods:** We analysed freshly-isolated PBMCs from nine individuals with genetically-confirmed MKD and compared these with PBMCs from parents who were heterozygous for *MVK* mutations, as well as PBMCs from unaffected controls and 2 individuals each with genetically-confirmed FMF, CAPS or TRAPS.

**Results:** Using the *in vitro* prenylation assay we observed a very clear accumulation of unprenylated Rab proteins in freshly-isolated PBMCs from eight MKD patients with different compound heterozygous mutations in *MVK*. In a patient homozygous for the V377I mutation the defect in Rab prenylation was milder but still detectable. Unprenylated Rap1A was also identifiable by western blotting in the MKD patients that had the greatest defect in Rab prenylation, but this western blot approach was considerably less sensitive than the *in vitro* prenylation assay. Importantly, the defect in Rab and Rap1A prenylation was absent in individuals with FMF, CAPS or TRAPS, and absent in individuals with heterozygous mutations in *MVK* or healthy volunteers.

**Conclusion:** These findings demonstrate that protein prenylation is indeed defective in individuals with MKD. Furthermore, the accumulation of unprenylated Rab proteins in PBMCs is a sensitive and specific biomarker of MKD that distinguishes this autoinflammatory disease from FMF, CAPS and TRAPS and could therefore be used to aid diagnosis.

**Disclosure of Interest**

None Declared

#### P2022 Encephalopathy in early-onset sarcoidosis: is it neurosarcoidosis or autoimmune encephalitis?

##### Nihal Sahin^1^, Sumeyra O. Cicek^1^, Aysenur P. Kisaarslan^1^, Zubeyde Gündüz^2^, Muammer H. Poyrazoglu^1^, Ruhan Düşünsel^1^

###### ^1^Pediatric Rheumatology, Erciyes University Faculty of Medicine; ^2^Pediatric Rheumatology, Acibadem Hospital, Kayseri, Turkey

####### **Correspondence:** Nihal Sahin

**Introduction:** Early onset sarcoidosis (EOS) / Blau Syndrome is NOD2 gene–associated chronic autoinflammatory disease characterized by the classic triad of skin rash, chronic symmetric arthritis, and recurrent uveitis. Pathologic investigation of organs reveals granulomatous inflammation. This disease involves many organ systems, however, neurologic involvement is rare. Cranial nerve palsy and papilledema were reported in the literature.To our knowledge, our patient is the first case with encephalitis in (EOS).

**Objectives:** We present a patient with encephalopathy in early-onset sarcoidosis.

**Methods:** Case report

The informed consent form for the publication was obtained from patient and her parents.

**Results:** A 12-year-old girl was diagnosed with juvenile idiopathic arthritis at the 3 years of age. While she was on methotrexate (MTX) and etanercept treatment, at 7 years of age, liver and kidney biopsy was performed due to high levels of transaminase and impairment of renal function tests. Granulomatous interstitial nephritis and granulomatous inflammation of the liver were detected in the biopsies, drug-induced sarcoidosis was considered in our patient, and ETA was stopped. MTX and systemic steroid were continued. Boggy synovitis and, camptodactyly were appeared on follow up. We reevaluated all clinical signs, and NOD2 gene analysis was performed 3 years later. M513T heterozygous mutation was detected. There was consanguinity, but no family history Thus, we diagnosed EOS. In the last three years, the disease activations included arthritis, interstitial lung disease, interstitial nephritis, and bilateral panuveitis. The treatment was systemic steroids on attack periods, MTX, and infliximab (INF). Lastly, she was admitted to our clinic with a headache and vomiting in June 2018. These complaints were there for two weeks and were increasing especially in the morning. She was on Methotrexate (15 mg/w sc), prednisolone (10 mg/d) and infliximab (200 mg/mt) A high fever, high blood pressure, lethargy, diplopia, and bilateral papilledema were detected on PE. Laboratory examination revealed leukocytosis, increased ESR and CRP and pyuria. E. coli was detected in the urine culture. Brain MRI and MRI angiography were normal. The patient underwent repeat lumbar punctures and opening pressures were 23 cm/H2O and 38 cm/H2O, respectively. Study of cerebrospinal fluid (CSF) revealed pleocytosis (120 leukocyte /mm3 and), the protein level of 0.35 g/l, normal chloride, and glucose level. CSF cultures were negative. Serology of viruses and Mycobacterium tuberculosis were negative. Electroencephalography (EEG) showed delta brush waves compatible with encephalitis. The pediatric neurology department considered autoimmune encephalitis. CSF limbic encephalitis antibody panel that included NMDA, AMPA1, AMPA2, CASPR2, LGI1, GABA B antibodies was performed. ANA, ANA subgroups were negative. After the urinary tract infection treatment, the fever was resolved. Intravenous immunoglobulin was given at a dose of 400 mg/kg/d for 5 days. But lethargy, diplopia, and papilledema were not resolved. Then the patient was treated with pulse intravenous methylprednisolone (1 g/kg/day for 5 days). After this treatment, his neurological findings improved rapidly. Infliximab, methotrexate, prednisolone (40 mg/d) were continued. At the last visit, neurological and ophthalmologic examinations were normal.

**Conclusion:** Early onset sarcoidosis is an autoinflammatory disease. Autoimmunity is not expected in EOS. Delta brush waves seen in EEG are frequently were detected in autoimmune encephalitis. We could not distinguish that this case is autoimmune encephalitis or neurological involvement of EOS. We wanted to present this interesting case to contribute to the literature.

**Disclosure of Interest**

None Declared

#### P2023 Risk factors of colchicine resistance in pediatric patients with familial Mediterranean fever: a single center experience

##### Nihal Sahin^1^, Aysenur P. Kisaarslan^1^, Sumeyra O. Cicek^1^, Zubeyde Gunduz^2^, Muammer H. Poyrazoglu^1^, Ruhan Düşünsel^1^

###### ^1^Pediatric Rheumatology, Erciyes University Faculty of Medicine; ^2^Pediatric Rheumatology, Acibadem Hospital, Kayseri, Turkey

####### **Correspondence:** Nihal Sahin

**Introduction:** Colchicine treatment high effectively prevents familial Mediterranean fever (FMF) attacks and secondary amyloidosis but, colchicine resistance was reported in 5 to 10% of patients receiving the highest dose tolerated. There are no validated criteria that could define colchicine resistance FMF.

**Objectives:** This study was aimed to determine the risk factors of colchicine resistance in pediatric FMF patients.

**Methods:** Two cohorts of pediatric Turkish patients followed in our pediatric rheumatology center were involved in the study. Familial Mediterranean fever was diagnosed by Turkish pediatric criteria. Group1 was determined as FMF, group2 was determined as colchicine-resistant FMF(CR-FMF).

**Results:** There were 50 patients in group 1 and 33 patients in group 2. An age, gender, consanguinity, family history of FMF, amyloidosis, and chronic renal failure, weight SDS, height SDS were not different between two groups(p>0,05). Presence of fever, peritonitis, pleuritis, arthritis, prolonged febrile myalgia, erysipelas like rash, headache, constipation, dysmenorrhea in attack periods and hepatosplenomegaly were not different between two groups (p>0,05). The myalgia and diarrhea in attacks period, chronic arthritis, and homozygous Exon 10 mutation were detected high in CR-FMF group p<0,05). In CR-FMF, the age of presence of symptoms, and age of diagnosis were detected lower than FMF groups(p<0,05). The colchicine compliance was same rate between the two groups. The number of attacks, international severity scoring system for familial Mediterranean fever (ISSF) scores, PRAS scores, CRP, ESR were more high in CR-FMF after at least 6 months colchicine treatments. All of the parameters were evaluated with multivariate logistic regression analysis. The presence of diarrhea, ISSF and PRAS scores were detected independent risk factors (OR 136,950, 95% CI: 1,128-16537,589, p=0,045; OR 8,313, 95%CI: 1,267-54,552, p=0,027; OR 3,170, 95%CI: 1,072-9,372, p=0,037, respectively).

**Conclusion:** This study will develop a formula to predict colchicine resistance. Early diagnosis of chronic inflammation and treatment can be kept from FMF complications.

**Disclosure of Interest**

None Declared

#### P2024 Distinctive features of familial Mediterranean fever with juvenile spondyloarthritis patients from familial Mediterranean fever and juvenile spondyloarthritis patients

##### Ayşenur Paç Kısaarslan^1^, Nihal Şahin^1^, Sümeyra Özdemir Çiçek^1^, Zübeyde Gündüz^2^, Muammer H. Poyrazoğlu^1^, Ruhan Düşünsel^1^

###### ^1^Pediatric Rheumatology, Erciyes University Faculty of Medicine; ^2^Pediatric Rheumatology, Acıbadem Hastanesi, Kayseri, Turkey

####### **Correspondence:** Nihal Şahin

**Introduction:** Musculoskeletal involvement of familial Mediterranean fever(FMF) extends from acute arthritis to juvenile spondyloarthritis(jSpA).

**Objectives:** Our aim was to identify distinctive features of FMF with jSpA patients from FMF and jSpA patients.

**Methods:** Three cohorts of pediatric Turkish patients followed in our pediatric rheumatology center were involved in the study. Familial Mediterranean fever was diagnosed by Turkish pediatric criteria. Juvenile spondyloarthritis was diagnosed by ILAR criteria. These two groups did not have other inflammatory conditions and chronic diseases. FMF patients who disclosed jSpA findings later andjSpA patients who disclosed FMF findings later were determined as FMF+jSpA group. Findings of FMF+jSpA were compared with FMF and jSpA groups.

**Results:** Thirty-two patients were in FMF+jSpA group, 50 patients were in FMF group, and 54 patients were in jSpA group. Ages of patients were 16,6(10,9-21,3), 13,7(6,8-21,5), and 14,0(7,0-16) in FMF+jSpA, FMF, and jSpA, respectively. Patients with FMF+jSpA had less fever and peritonitis in attack periods than FMF patients(p<0,05). They had more myalgia, cronic arthritis, high ESR levels on remission, and high disease activity score( p<0,05). In FMF patients, FMF50 response was reached more than FMF+jSpA patients after colchicine treatment(p<0,05). Two groups had the same rate of at least one Exon 10 mutationsin MEFV gene (87,4% to 88%, respectively). Other FMF attack findings, proteinuria and amyloidosis were non significant differences in two groups(p>0,05).

Patients with FMF+ jSpA had less small joint involvement, sacroiliac tenderness, HLA-B27 positivity, jSpADA score than jSpA patients(p<0,05). Plantar faciitis and JADI-E score were high in FMF+ jSpA group than jSpA (p<0,05). Other jSpA findings, uveitis and sacroiliitis detected by MRI were same rate in two groups. Non steroidal antiinflammatory drug usage was high in patients with FMF+ jSpA more than jSpA. Usage of steroid, DMARDs, and biologic DMARDs(except for IL-1 antagonist) were at the same rate in two groups.

**Conclusion:** Articular involvement of familial Mediterranean fever especially in M694V carriage may be associated with jSpA. Patients with FMF+ jSpA have more subclinical inflammation. Because the disease damage index is high in this group, diagnose and treatment should be made on time.

**Disclosure of Interest**

None Declared

#### P2025 Type 1 interferonopathy presenting with fever, fatigue, chronic urticaria, arthritis, elevated liver enzymes and hyperferritinemia in a 13-year-old girl – an important differential diagnosis to systemic juvenile idiopathic arthritis

##### Eduardo Salamano^1^, Min Ae Lee-Kirsch^2^, Christoph Rietschel^1^

###### ^1^Rheumatology, Clementine Children Hospital, Frankfurt; ^2^Immunology, Universitätsklinikum Carl Gustav Carus, Dresden, Germany

####### **Correspondence:** Eduardo Salamano

**Introduction:** Type 1 interferonopathies are a group of heterogenous diseases which are characterized by a dysregulation of the type 1 interferon axis and as a result an inadequate Type 1 interferon activation causing autoinflammation and autoimmunity. While type 1 interferonopathies are genetic disorders the underlying mutation is not always known. The variety of symptoms and the abundance of possible differential diagnosis often lead to difficulties in recognizing the disease and thus delay an effective therapy. Janus kinase inhibitors have been shown to yield promising results in the treatment of these rare immunologic conditions.

**Objectives:** We report on a previously healthy 13-year-old german girl presenting with recurrent and persistent fever, chronic itching urticaria-like rash and fatigue. Laboratory results showed strongly elevated liver enzymes, LDH and ferritin with only mild signs of inflammation. During the clinical course she developed joint pain with arthritis of both wrists and leukopenia. After exhaustive diagnostic workup including bone marrow biopsy steroid treatment was given and effective for fever and arthritis with normalization of liver enzymes and ferritin while urticaria-like skin lesions and pruritus only slowly improved. Due to steroid dependence IL-1-blockade with anakinra was started but only partially effective.

**Results:** Results for HLA-B27, ANA, ENA, anti-dsDNA, ANCA, AMA, LKM1, immunoglobulin A/G/M/E, ACE, Transglutaminase-AK-IgA/IgG, C1q, C2, C3, C4, CH50 were normal. S100-A8/A9 was only moderately elevated. Possible infectious causes were ruled out. A liver biopsy was performed with the result of slight non-specific inflammation. Ultimately molecular genetic panel diagnostic for known rare autoinflammatory conditions was performed and showed no mutations.

With progression of symptoms and high steroid burden further treatment options were evaluated. Due to the resemblance of some disease aspects with the clinical picture of interferonopathies we ordered a type 1 interferon signature showing a very strong activation. Type 1 interferonopathy was thus suspected and treatment with the Janus kinase inhibitor baricitinib was initiated, which lead to an immediate improvement of all symptoms and a regression of liver enzymes, ferritin and inflammatory markers. A control of the type 1 interferon signature 4 weeks after start of the treatment presented a normalization of the interferon activity confirming the suspected differential Diagnosis of a Type-1-Interferonopathie. Two months into treatment the patient is currently healthy and shows very good tolerance to the medication with no side effect to this point.

**Conclusion:** Type 1 interferonopathies are autoinflammatory diseases with a very heterogenous clinical manifestation. Symptoms can include every organic system, most common are recurrent fever, cutaneous and neurological manifestations. They should be considered as a differential diagnosis to systemic autoinflammatory diseases like systemic juvenile arthritis. In this case we present a girl with possible type 1 interferonopathy with no relevant medical antecedents or familial history of autoinflammation. While extensive genetic testing in this girl yielded no known genetic cause of the disease, evaluation of the type 1 interferon signature led to a specific treatment with a Janus kinase inhibitor, which ultimately showed a rapid improvement with complete remission. Considering the short duration of treatment to this point, the further disease course needs to be monitored carefully.

**Consent for publication has been obtained from patient**

Yes

**Disclosure of Interest**

None Declared

#### P2026 Cryopyrin-associated periodic syndromes in adults: diagnosis, therapy in clinical practice

##### Svetlana Salugina, Evgeny Fedorov, Tatjana Dubinina, Svetlana Palshina

###### Nassonova Research Institute of Rheumatology, Moscow, Russian Federation

####### **Correspondence:** Svetlana Salugina

**Introduction:** Cryopyrin-associated periodic syndromes (CAPS) – a group of rare monogenic hereditary autoinflammatory diseases (AIDs). CAPS is clinically characterized by fever, urticarial skin rash, musculoskeletal features, eye manifestations, neurological symptoms, hearing loss, acute phase markers. There is a high risk of amyloidosis, renal failure. Age of onset may be different. The diagnosis is sometimes delayed for many years. IL-1 inhibition (anakinra, canakinumab) is indicated for the whole spectrum of CAPS (FCAS, MWS, CINCA/NOMID)

**Objectives:** To present clinical, laboratory and demographic characteristics and therapy of patients with CAPS in the practice of an adult rheumatologist

**Methods:** The study included 11 inpatients or outpatients with CAPS, who were on examination in Federal center of Rheumatology (10 women with MWS, 1 boy with CINCA/NOMID), aged from 18 to 60 years. All conducted standard rheumatological examination, including ESR, CRP.

**Results:** The age of the onset was 0-51 years, in 10 of 11 in childhood. Inflammatory attacks in 7 pts started in the first year of life, 1 patient – in the age of 51 years. Delay in diagnosis varied from 1 to 44 years, 1 - 6 years in 2 pts, 15-17 years in 4 pts, above30 years in 4 pts. The fever was in all pts, urticarial skin rash in 9 (81,8%), musculo-skeletal manifestations in 11 (mostly benign oligoarthritis, 4 –polyarthritis). 1 patient with polyarthritis had comorbid pathology - rheumatoid arthritis. Eye manifestations (conjunctivitis, corneal dystrophy, uveitis) had 9 (81,8%), sensorineural hearing loss – 6 (54,5%). 2 pts had abdominal pain, nausea, vomiting, 4 – headache. Duringthedisease all pts had increased ESR, CRP. Among our pts there were 5 family cases. Mutations in the NLRP3 gene in heterozygous state were revealed in all patients except one boy with CINCA/NOMID. 6 pts before diagnosis received corticosteroids, treatment withIL-1 inhibitors was initiated in 7 pts (canakinumab -6, anakinra-1) with a complete or partial response in all pts.

**Conclusion:** In the practice of an adult rheumatologist there are patients with recurrent fevers and other manifestations of systemic inflammation, among which there may be patients with CAPS. These patients need timely diagnosis and appointment of targeted therapy. Most patients had childhood - onset. It is also very important to take into account the presence of periodic fevers in the family history. The adult patients have big problems with initiating or continuing treatment with IL-1 inhibitors

**Disclosure of Interest**

None Declared

#### P2027 Pulmonary screening of FMF patients during periodic follow-up: clinical and pathogenetic considerations

##### Anna Sargsyan

###### Internal Medicine, YSMU, Yerevan, Armenia

**Introduction:** The most prominent pulmonary manifestations of FMF are chest attacks due to pleuritis. It occurs in 45% of patients, sometimes as a sole manifestation of disease. However, in Armenians pleuritis is more prevalent than arthritis- 84% in 3 series of total 335 FMF patients^1^.

**Objectives:** To reveal the spectrum of possible pulmonary manifestations in FMF patients and explore correlations with inflammatory biomarkers.

**Methods:** Pulmonary screening of a random group of 155 Armenian patients with FMF who had pleuritic chest attacks. Pulmonary function test (PFT) by spirometry- FEV_1_(L), FEV_1_%predicted, FVC(L), FVC% predicted, FEV_1_/FVC%; TTE for systolic pulmonary artery pressure (sPAP), chest x-ray and HRCT-scan. C-reactive protein (CRP) by immunoturbidimetry, SAA by ELISA, and capillary blood gases (PO2, PCO2, SpO2) were measured. All tests were done in attack free period. Patients were followed prospectively for 5 years.

**Results:** Comparison of PFT volumes revealed statistically significant differences in FMF-amyloidosis patient group vs FMF group (Mann-Whitney-U test, mean±SD):FEV1%pred 71.9±39 vs 71.8±13.3, p=0.046; FVC%pred 69.5±13.3 vs 74.4±10.7, p=0,033; FEV1/FVC% 77.39±9.5 vs 85.2±8.1,p<0.000. We observed 16 patients with pneumonia (lobar and interstitial), in 5 patients pneumonia had recurrent or persistent course. Pulmonary hypertension (sPAP>35mmHg) was diagnosed in 14 patients. 1 patient had plate atelectasis of right lower lobe during chest attack, 2 patients bronchial asthma episodes, 2 bronchitis; 1 polyarteritis nodosa, 1Henoch-Schonlein purpura, 1 livedoid vasculopathy and 1Raynaud disease.

Lab tests were as following in FMF-amyloidosis group vs FMF group (mean±SD):CRP 17.74±13.74mg/L vs 11,88±13.79 and SAA 33±66.6mg/L vs 5.25±4,45 p<0,000; PO_2_ 74±11.36mmHg, PCO_2_ 35.3±4.5mmHg, SpO_2_ 90.1±10.26% vs PO_2_ 83.6±8.95, PCO_2_ 39.4±3.6, SpO_2_ 94.6±3.38 p<0.000.

**Conclusion:** Clinically overt heart and lung diseases in FMF result from extrarenal amyloid deposition^2^. Pulmonary hypertension due to amyloidosis is rare. Pulmonary hemorrhage and infiltrates highly possible as FMF associates with vasculitis. Atelectasis during chest attacks; ARDS due to pneumonia are described^3^.

We demonstrated that FMF patients with amyloidosis have hypoxia and it may have an additive pathogenetic effect for pulmonary complications. We have shown *for the first time* that patients without renal amyloidosis may develop pulmonary hypertension. These findings raising an intriguing question about pathogenetic mechanisms. We assume that the respiratory symptoms in FMF result from continuous low-grade inflammation. This conclusion drawn from the significantly raised levels of inflammatory biomarkers, CRP and SAA. The present study is the first to consider clinically relevant confounders. Pleural adhesions were frequent radiologic findings (38%). We reveal impaired pulmonary function and gas exchange in patients with amyloidosis. CRP but not SAA well correlates with PO_2_, SpO_2_, and FEV_1_/FVC in FMF-amyloidosis group. Moreover, we reveal correlations between CRP and PO_2_ in FMF patients group without amyloidosis. While pleural adhesions, atelectasis and pulmonary infiltrates lead to restriction, obstruction causes hypoxia. A physician, taking care of FMF patient with chest attack, can face various pulmonary manifestations and should aware of their origin.

1 Ben-Chetrit E, Touitou I. FMF in the world. Arthritis Rheum 2009;61(10):1447-53

2 Lidar M et al. Thoracic and lung involvement in FMF. Clin Chest Med 2002;23:505-11

3 Arai Y et al. FMF mutations in a patient with recurrent episodes of ARDS. Clin Immunol 2013;147,58-60.

**Disclosure of Interest**

None Declared

**Table 1 (abstract P2027).** Correlations (Spearman's) between PFT volumes, blood gases and CRP level

	CRP	
	FMF-amyloidosis, n=75	FMF, n=80
FEV_1_	-0.063	-0.100
FVC	0.139	0.168
FEV_1_/FVC	-0,408**	-0.076
PO_2_	-0,301*	-0,329*
PCO_2_	-0,022	-0.037
SpO_2_	-0,317*	-0.131

*p<0.01, **p<0.001

#### P2028 Identification of novel loss-of-function mutations in two independent patients with deficiency of adenosine deaminase 2

##### Oskar Schnappauf, Natalia Sampaio Moura, Qing Zhou, Natalie Deuitch, Daniel Kastner, Ivona Aksentijevich

###### National Human Genome Research Institute (NHGRI), National Institutes of Health (NIH), Bethesda, United States

####### **Correspondence:** Oskar Schnappauf

**Introduction:** Deficiency of adenosine deaminase 2 (DADA2) is an autosomal recessive disorder that manifests with fever, rash, hypocellular bone marrow, early-onset vasculitis, and propensity to ischemic and hemorrhagic stroke. Over 60 pathogenic mutations, mostly missense variants, have been identified to date.

**Objectives:** The current study aimed to identify novel cryptic loss-of-function mutations in the *ADA2* gene in two unrelated families.

**Methods:** A variety of molecular and biochemical methods were used to identify novel pathogenic variants in the *ADA2* gene and to determine the effect of these variants on the RNA and protein expression. The utilized methods include Sanger sequencing, multiplex ligation-dependent probe amplification analysis (MLPA), quantitative RT-PCR (qRT-PCR), long range PCR and long read sequencing, RNA sequencing, and ADA2 enzyme activity assays.

**Results:** The first index patient is a 5-year-old female who was born to a consanguineous Pakistani family and presented with features consistent with Diamond-Blackfan anemia (DBA). Sequencing analysis did not identify pathogenic mutations in DBA-associated genes and a chromosomal microarray did not detect duplications or deletions. Interestingly, several regions of homozygosity were identified in the proband, one of which comprises the *ADA2* gene locus. Sanger sequencing of *ADA2* did not identify a pathogenic variant in the patient. Multiplex ligation-dependent probe amplification analysis (MLPA) identified a homozygous duplication of a region comprising exon 7 of *ADA2* in the proband, her affected sister, her affected father, and a heterozygous duplication of this region in the proband’s mother. Subsequent qRT-PCR experiments confirmed a complete loss of RNA-expression in the individuals homozygous for the duplication. Reduced ADA2 enzyme activity corroborated the qRT-PCR findings. Long-range PCR and long-read sequencing analysis were performed to localize the breakpoints of the duplication.

The second index patient is a 17-year-old female who presented with a history of ischemic strokes, livedo rashes and vasculitis. She was found to be heterozygous for a known pathogenic variant in *ADA2* (c.1358A>G, p.Y453C). This variant was inherited from the proband’s father and is also present in her three at the time unaffected siblings. The ADA2 enzyme activity assay revealed that the proband and two of her siblings have low to absent ADA2 enzyme activity. Her parents, as well as a fourth sibling, exhibited ADA2 enzyme levels in the carrier range. Haplotype analyses revealed that the three children with low ADA2 activity inherited the same allele from their mother while the fourth sibling inherited the unaffected maternal allele. WGS of the mother and the 3 affected children identified a novel canonical splice site variant (ADA2: c.-47+2T>C) in an alternative ADA2 transcript (NM_001282225.1) present in all four individuals. Sanger sequencing analysis confirmed the segregation of this variant with the disease. This variant is absent from population databases and predicted to effect splicing. RNA expression analysis showed that the affected individuals express only 50% of the normal *ADA2* mRNA and subsequent cDNA sequencing confirmed that only the allele carrying the pathogenic c.1358A>G, p.Y453C variant is expressed.

**Conclusion:** The identification of two novel cryptic mutations in *ADA2* that were not detected by Sanger sequencing, suggests the incorporation of additional methods in the diagnosis of DADA2. ADA2 enzyme activity testing should be utilized to identify individuals with absent or almost absent enzyme activity even if Sanger sequencing was negative. Subsequently, WGS and MLPA analyses can be applied to identify cryptic single nucleotide variants and CNVs, respectively.

**Consent for publication has been obtained from patient**

Yes

**Disclosure of Interest**

None Declared

#### P2029 Humoral immune compromise with autoinflammatory disease due to mutation in NLRP12

##### Analia G. Seminario, María S. Caldirola, Ileana Moreira, Lorena Regairaz, Liliana Bezrodnik

###### Immunology, Centro de Inmunología Clínica Dra Bezrodnik, Buenos Aires, Argentina

####### **Correspondence:** Analia G. Seminario

**Introduction:** The hereditary periodic fevers, compromise a group of mendelian autoinflammatory disorders characterized by recurrent episodes of fever and systemic inflammation, sometimes complicated with amyloidosis

**Objectives:** Report a case of periodic fever syndrome due to NLRP12 mutation, with humoral immune compromise.

**Methods:** Retrospective analysis of a clinical history of a patient with primary immunodeficiency with humoral compromise and autoinflammatory syndrome

**Results:** Boy 8y.o. with recurrent severe fever episodes, since 1y.o. The crises were like bacteremia with severe abdominal pain, arthralgia and rash with high levels of CRP and ESR. In skin biopsy, it was observed neutrophilic vasculitis. He suffered several bronchospasm sinusitis and acute otitis media. IgG and IgA low levels, not response to pneumococcal antobodies and low post switched memory B cells. No improvement with antibiotic prophylaxis. Due to its humoral immune defect, he started intravenous gammaglobulin (IVIG) 800mg/kg/dose. He presented in the evolution coxsackie B type3 in stool, negative in CFS, needing high doses of weekly IVIG with good response and no relapse until today. As our patient presented recurrent infections, with hypogammaglobulinemia and autoinflammatory syndrome we studied ADA activity (NORMAL). Molecular studies have been done, heterozygous mutation in NLRP12, was found. The infections improved but severe episodes of abdominal pain and does not get better with colchicine treatment.Today his treatment is IVIG, and oral budesonide. An endoscopy study to rule out amyloidosis is pending.

**Conclusion:**We want to present a patient with humoral defect and autoinflammatory diseases, due to NLRP12 mutations and demonstrate the crucial role of NLRP12 in inflammatory signaling pathways.

**Consent for publication has been obtained from patient**

Yes

**Disclosure of Interest**

None Declared

#### P2030 Genetic screening in patients with undifferentiated periodic fever syndrome

##### Ferhat Demir^1^, Ozlem Akgun Dogan^2^, Yasemin Kendir Demirkol^2^, Kubra Ermis Tekkus^3^, Sezin Canbek^3^, Nuray Aktay Ayaz^4^, Levent Doganay^3^, Betul Sozeri^1^

###### ^1^Pediatric Rheumatology; ^2^Pediatric Genetic; ^3^Genomic Laboratory (GLAB), Health Directorate of Istanbul, University of Health Sciences, Istanbul, Umraniye Training and Research Hospital; ^4^Pediatric Rheumatology, University of Health Sciences, Istanbul, Kanuni Sultan Suleyman Training and Research Hospital, Istanbul, Turkey

####### **Correspondence:** Betul Sozeri

**Introduction:** Autoinflammatory diseases (AID) are a group of hereditary diseases characterised by inflammation periods accompanied with clinical findings such as fever, skin rash, lymphadenopathy, abdominal pain, musculoskeletal symptoms, and with sign of inflammation in the blood. Each disease has own typical clinical findings and they are associated with mutations in specific genes such as in MEFV gene in familial Mediterranean fever (FMF), MVK gene in hyperimmunglobulin D syndrome (HIDS), TNFRSF1A gene in tumor necrosis factor-alpha receptor associated periodic fever syndrome (TRAPS) and NLRP3 gene in cryopyrin associated periodic fever syndrome (CAPS) . Also in some patients with periodic fever syndrome (PFS), clinical signs of these diseases can be seen but no mutation can be detected in the related genes. There are also patients exhibit the incomplete phenotype of a disease or overlap signs of more than one AID. The diagnosis of these undifferentiated patients have difficult and may not be possible by a single target gene analysis. Screening of the periodic fever syndrome (PFS) panel including various AID genes may be beneficial to define the atypical cases. Molecular genetics has an important role for lead to diagnosis in these patients.

**Objectives:** The aim of this study was to investigate the genotypic diagnosis in patients with non-characteristic PFS findings for any AID.

**Methods:** This is a prospective study and conducted between June 2016 and December 2018. Next-generation sequencing (NGS) analysis was performed by using “Fever and AutoInflammatory Syndrome panel: Panel by Sophia Genetics” including 8 genes (MEFV, MVK, NLRP3, NLRP12, TNFRSF1A, TNFRSF11A, LPIN2 and PSTPIP1) in 30 patients with undifferentiated PFS. Clinical features and genetic results were evaluated together and final diagnoses were determined.

**Results:** Thirty patients included in the study did not have typical clinical features for any of the eight monogenic diseases in the PFS panel. In the result of the genetic screening; disease-causing mutation was found in MEFV gene in 12 patient, in NLRP3 gene in four patient, in NLRP12 gene in two patient and in MVK gene in one patient. Also, genetic variants of uncertain significance (VUS) in different genes were shown in five patient. No mutation was detected in remaining six patient. The final diagnosis was made by both phenotypic and genotypic data. 12 patients were diagnosed with FMF, four were FCAS, two were FCAS2, one was TRAPS and one was HIDS. Patients with negative genetic screening or had mutation as VUS, were followed as undifferentiated PFS.

**Conclusion:** Autoinflammatory diseases may not always be appear with typical clinical findings of related disease. In such patients, target gene sequencing and detection of underlying disease can be challenging. Our study has shown that the NGS analysis may help to determined the diagnosis in patients with non-characteristic PFS findings for any AID.

**Disclosure of Interest**

None Declared

#### P2031 Deficiency of ADA2 from childhood to adult; the same mutation in a family

##### Betul Sozeri^1^, Gozde Ercan^2^, Ozlem Akgun Dogan^3^, Jale Yıldız^4^, Ferhat Demir^1^, Levent Doğanay^4^

###### ^1^Pediatric Rheumatology; ^2^Pediatrics; ^3^Pediatric Genetics; ^4^Genomic Laboratory (GLAB), Health Directorate of Istanbul, University of Health Sciences, Istanbul, Umraniye Training and Research Hospital, Istanbul, Turkey

####### **Correspondence:** Betul Sozeri

**Introduction:** The deficiency of adenosine deaminase 2 (DADA2) has recently been defined as a monogenetic autosomal recessive autoinflammatory disease. It mainly characterized by high fever, livedoid racemosa, early onset stroke, and mild immunodeficiency, clinically polyarteritis nodosa (PAN) like symptoms. Wide spectrum of severity in phenotype as well as in the age of onset has been reported in the literature. This phenotypic variability is also found in our clinical practice even in patients with the same mutation.

**Objectives:** We present a case series of nine members of same family. p.Gly47Arg mutation in CECR1 gene was detected in homozygous state in five patients who have moderate to severe clinical findings, and in heterozygous state in four members with mild non-specific symptoms in the family.

**Methods:** Genomic DNA was extracted from peripheral blood sample of the index case and the next generation sequencing was performed on Illumina MiSeq (v1.9) platform using the Periodic Fever Panel. Bioinformaticanalyses were performed by using SophiaDDM software. A previously reported missensec.139G>A change in *CECR1*, which leads the glysine to arginine substitution at position 139, was detected in homozygous state in the index case. This variation were searched in the parents and siblings as well as the symptomatic cousins, by Sanger sequencing. Five homozygos and four heterozygos variations in *CECR1* were detected.

**Results:** Fever, skin manifestations and neurological system involvement was observed in the index case and three of which were homozygos mutations but in the one, none of these was observed. A mild to severe involvement of neurological system and skin manifestations was seen more severe in homozygous mutant patients with early onset disease even resulted with necrosis. Gastrointestinal system involvement was only observed in index case and the late-onset patient with homozygous mutation. Arthritis was detected in three of patients with homozygous mutations. The age of onset of the disease is quite different from each other, even in individuals with the same mutation. In the light of these results, we suggest that early age of onset in the disease is associated with serious clinical findings and poor outcome

**Conclusion:** With this report, we aimed to further delineate the phenotype and demonstrate variability both in the clinical findings and in the age of onset in five patients with the same mutation from the same family. As we know, there are any family cases presented with DADA2 almost in all members have been reported yet.

**Disclosure of Interest**

None Declared

#### P2032 Autoinflammatory diseases in Ukraine

##### Yuriy Stepanovskyy^1^, Yaryna Boyko^2^

###### ^1^Pediatric Infectious Diseases and Pediatric Immunology, Shupyk National Medical Academy of Postgraduate Education, Kyiv; ^2^Rheumatology, Western Ukrainian Specialized Pediatric Medical Centre, Lviv, Ukraine

####### **Correspondence:** Yuriy Stepanovskyy

**Introduction:** First case of monogenic autoinflammatory disease (CINCA/NOMID) was diagnosed in Ukraine in 2001. Since, the number of patients with autoinflammatory diseases (AIDs) was slowly growing up. More than 90% of patients were diagnosed and were under surveillance in two centers: Kyiv city pediatric center of clinical immunology and Western Ukrainian Specialized Pediatric Medical Centre.

**Objectives:** The purpose of this study is to describe current situation with AIDs in Ukraine and its problems, and advances.

**Methods:** We reviewed the charts of patients with autoinflammatory diseases from 2 centers where they were followed up. Among them, 7 patients with monogenic AIDs (genetically confirmed), 5 with strong clinical and laboratory phenotype of AIDs and 90 patients with PFAPA-syndrome.

**Results:** We analyzed known patients with AIDs in Ukraine. Spectrum of patients is presented it the Tab. 1. Delay with diagnosis of monogenic AIDs was 2 to 9 years, and 1,5 years for PFAPA patients. The most common burdens on the way to diagnosis were: very low awareness of physicians about AIDs, absence of accessible biologic treatment and absence of genetic tests.Actually, all patient’s genetic tests were done in foreign laboratories. In 2018 pediatric immunology community, initiated introduction of IL-1 driven diseases with anakinra with government support.

**Conclusion:** Awareness about autoinflammatory disorders is still poor in Ukraine.Access to treatment with anakinra in Ukraine opened new opportunities to moving forward in the field of autoinflammatory diseases. Searching ways to facilitate access to genetic diagnostic is always a question to be resolved.

**Disclosure of Interest**

None Declared

**Table 1 (abstract P2032).** See text for description

Patient	Sex	Disease type	Genetic test
1, 2	F	FMF	confirmed & not done
3	M	HIDS	confirmed
4, 5, 6	F, F, M	CINCA/NOMID	confirmed
7	F	DADA2	confirmed
8	F	Cold-induced AID	not done
9	F	TRAPS-like phenotype	not done
10	F	AID, undefined	new mutation was found, under investigation at NIH
11	F	AID, undefined	WGS, mutations not found
12	F	NRLC4-MAS like illness	Panel sequencing for 15 genes including *NLRC4*-negative result

#### P2033 The spectrum of autoinflammatory syndromes at postgraduate institute of medical education and research, Chandigarh, India

##### Deepti Suri^1^, Amit Rawat^1^, Anju Gupta^1^, P. Vignesh^1^, Ankur Jindal^1^, Sagar Bhattad^1^, Marco Gattorno^2^, Adriana A de Jesus^3^, Raphaela Goldbach-Mansky^3^, Surjit Singh^1^

###### ^1^Department of Pediatrics, Advanced Pediatrics Centre, Postgraduate Insitute of Medical Education and Research, Chandigarh, India; ^2^Center for Autoinflammatory Diseases and Immunodeficiencies, G. Gaslini Institute for Children, Genoa, Italy; ^3^Translational Autoinflammatory Disease Section, NIAID, NIH, Bethesda, Maryland, United States

####### **Correspondence:** Deepti Suri

**Introduction:** This paper presents clinical and molecular data of patients with auto-inflammatory diseases (AIDs) at Postgraduate Institute of Medical Education and Research, Chandigarh, India.

**Objectives:** To highlight the clinical and molecular spectrum of AIDs at our centre.

**Methods:** A review of case records of patients with AIDs attending the Pediatric Immune Deficiency in the last 7 years.

**Results:** A total of 35 children with AIDs were identified and their profile is summarized in the Table. Among the periodic fevers group, Periodic Fever, Aphthous Stomatitis, Pharyngitis and Adenitis (PFAPA) was diagnosed in 8 patients. Three patients had Cryopyrin-Associated Periodic Syndrome (CAPS) while 2patients hadTumor Necrosis Factor Associated Periodic Fever syndrome (TRAPS). A child novel *PSTPIP 1* gene mutation with Pyogenic Arthritis, Pyoderma gangrenosum, and Acne (PAPA) was diagnosed. Ten children werefound to have chronic recurrent multifocal osteomyelitis. The first ever Indian child with Deficiency of IL-1 Receptor Antagonist (DIRA) had presented with lytic bone lesions in early infancy and has been reported. Another infant suspected to have DIRA responded to glucocorticoid therapy with radiological improvement, however, molecular confirmation is awaited.Eight children had Blau syndrome with*NOD 2* gene mutations. There were considerable delays in diagnosis and difficulties in treatment due to non-availability of anti- IL-1 blocking drugs(Anakinra or Canakinumab) in India. Two children with NOMID/CAPS died due to renal amyloidosis.

**Conclusion:** Delay in diagnosis and lack of availability of drugs for treatment of AIDs are difficulties in managing patients in India.

**Disclosure of Interest**

None Declared

**Table 1 (abstract P2033).** See text for description

Patient Profile	Diagnosis	Prominent clinical features	Age at Diagnosis (years)	Treatment	Outcome
NOMID S,13 years F	*NLRP3 C.913G>C, p.D305H* (de novo mutation)	Fever, rash in infancy, bony overgrowth, headache Amyloidosis, misdiagnosed as JIA,	13 years	Thalidomide	Doing well Symptomatically better
NOMID K,11years M	NLRP3 p.Thr349Ile Parents negative (de novo mutation). Mosaicism	Crippling arthritis, Bed bound, amyloidosis	11 years	Prednisolone	Died due to refractory renal failure secondary to amyloidosis
K,8 years F	*CAPS* *NLRP3* mutation	Diagnosed as steroid resistant nephrotic syndrome	8 years	Prednisolone	Died
TRAPS (n=2) M,4 years, F	*TNFRSF1A* p. Cys43Tyr Transmitted from father	Periodic fevers, subcutaneous swellings, rash, periorbital edema Father symptomatic	4 years	Etanercept	Doing well on etanercept
KD,35 years, F	*TNFRSF1A* p.Pro301His (Unreported VUS)	Fever, conjunctivitis, Pustular psoriasis with high Inflammatory markers	6 years	Prenisolone cyclosporine	Partial control Intermittent flares of skin and fever
DIRA (n=1) B/O V, 2months, F^1^	*IL1RN* deletion, at chr2_hg19_113,865,011 and chr2_hg19_113,887,227 homozygous 22,216bp deletion spans the first four exons of IL1RN Parents carrier for same mutation (NM_173843)	Infant with multifocal osteitis, few pustules,	-	Anakinra*	Doing well
S, 1.5 months, M	Result awaited	Multifocal osteitis involving humerus, clavicle, ribs	1.5months	Prednisolone for 6 months	Doing well
PAPA (n=1) M, 8 years F	*PSTPIP1 gene* p.Thr 68Met	Pyoderma, abscess, colitis, fever	4 years	Prednisolone, Infliximab	Died
APLAID (n=1) M, 3 years, F	*PLCG2* exon 22 c.2393A>G p.Asn798Ser Heterozygous	Joint swelling, rash and bloody diarrhoea	6 years	Prednisolone	Doing well Intermittent skin flares

*Therapy courtesy compassionate use supply NIH

#### P2034 Patient with FMF presented by isolated myositis

##### Sema N. Taskin, Aysenur Kisaarslan, Sümeyra Ozdemir Çiçek, Nihal Sahin, Muammer Hakan Poyrazoğlu, Ruhan Dusunsel

###### Pediatric Rheumatology, Erciyes University Medical Faculty, Kayseri, Turkey

####### **Correspondence:** Sema N. Taskin

**Introduction:** Familial Mediterranean fever (FMF) is a genetic disease characterized by recurrent febrile episodes and mostly by the inflammation of serous membranes. We presented our case whom presented with acute myositis and was diagnosed as FMF.

**Results:** A six year and 5 mounth old girl complained of severe pain in her right leg and a gait while walking for a period of one week. It was learned from the history that the right ankle swelled and hiperemic macular rash were determined on lateral malleol area three years ago and the acute phase reactants were elevated at that time.There was no consanguinity and family history of autoinflammatory diseases. Physical examination of the patient revealed swelling, redness, thenderness, heat increase of on the calf muscles and limitation of extension of the knee joint. White blood cells, C-reactive protein (CRP), and eritrocyte sedimantation rate (ESR) were eleveated, muscle enzymes were normal levels.In the USG, the right popliteal fossa fat tissue was prominent and inflamed, and the neural structures were thick and edematous.Magnetic Resonance imaging of the thighs and right leg posterolateral muscles was consistent with myositis. After NSAID treatment, the clinic improved. However, the high CRP and ESR persisted for six mounths. There was no mutation on TRAPS gene. When the M694V homozygous mutation was detected in the FMF genetics of the patient, Familial Mediterranean Fever was diagnosed and colchicine treatment was started. Other autoinflammatory diseases genes could not studied. Acute phase reactans of the patient regressed after the treatment. The patient experienced arthritis attack at once for two years follow up.

**Conclusion:** The patients who carried M694V homozygous mutations may presented nonclassical findings of FMF.

**Consent for publication has been obtained from patient**

Yes

**Disclosure of Interest**

None Declared

#### P2035 Interstitial lung disease in a newborn affected by mevalonic aciduria

##### Sofia Torreggiani^1,2^, Carlo Pietrasanta^3,4^, Francesca Minoia^1^, Giovanni Filocamo^1^, Andrea Ronchi^3^, Stefano Volpi^5^, Roberta Caorsi^5^, Marco Gattorno^5^, Francesco Caroli^6^, Alice Grossi^6^, Isabella Ceccherini^6^, Lorenza Pugni^3^, Fabio Mosca^3,4^

###### ^1^Pediatria Media Intensità di Cura, Fondazione IRCCS Ca’ Granda Ospedale Maggiore Policlinico, Milan; ^2^University of Milan; ^3^NICU Fondazione IRCCS Ca’ Granda Ospedale Maggiore Policlinico, Milan; ^4^University of Milan, Department of Clinical Sciences and Community Health, Milan; ^5^Clinica Pediatrica e Reumatologia; ^6^UOC Genetica Medica, Istituto Giannina Gaslini, Genova, Italy

####### **Correspondence:** Sofia Torreggiani

**Introduction:** Mevalonic aciduria (MA) is the most severe phenotype of mevalonate-kinase deficiency (MKD), with a onset in early infancy and poor prognosis. MA diagnosis may be challenging in the neonatal period given its rarity and its unspecific symptoms that frequently recall those of other neonatal diseases. To our knowledge, interstial lung involvement has never been described as onset feature in a newborn with MKD.

**Objectives:** We report the case of a newborn affected by MKD characterized by interstitial lung disease.

**Methods:** The patient underwent laboratory and radiology evaluation as clinically indicated. Direct Sanger sequencing was used to screen the 10 exons of the MVK gene.

**Results:** A female neonate born at term from consanguineous parents was referred to our hospital at 16 days of life (DOL) for mild hypotonia and persistent raised inflammatory markers despite antibiotic therapy. Infectious work-up was negative for both viral and bacterial infections. Chest x-ray revealed bilateral perihilar peribronchial thickening. Electroencephalography (EEG) reported moderate diffuse anomalies of background activity without major abnormalities. On DOL 20 the first episode of fever was recorded. Due to worsening tachypnea and persistent abnormal chest x-ray, a pulmonary CT scan was performed and showed diffuse ground-glass bilateral infiltrates consistent with alveolar-interstitial lung disease. On DOL 22 a palpable maculo-papular skin rash appeared on feet and hands, vanishing spontaneously 24 hours later. Bone marrow examination and levels of perforins, neuron-specific enolase and urinary catabolites of catecholamines were normal. A total body MRI was normal except for a mild cerebellar hypoplasia and the known interstitial lung disease. The patient kept presenting hypotonia, relapsing episodes of fever and skin rashes, developed anemia requiring blood transfusions and failure to thrive became evident. Type-I IFN signature was negative. A genetic test was requested, as well as quantification of urinary levels of mevalonic acid, which were markedly above the normal range. Direct Sanger sequencing allowed to detect a homozygous c.709A>T missense mutation in the exon 8 of the MVK gene, coding for a protein substitution p.T237S already classified as pathogenic in the INFEVERS database (http://fmf.igh.cnrs.fr/ISSAID/infevers/) and therefore consistent with the diagnosis of MKD. Both parents and her sister were found to be heterozygous carriers of the same mutation. On DOL 38 treatment with anakinra was started, with prompt regression of fever and skin rash, decrease in inflammatory markers, increase in reticulocytes count and weight gain. Hypotonia improved but persisted. The patient was discharged from hospital on DOL 56 in good clinical conditions, with acute phase reactants within the normal range and mild hypotonia. She is now 4 months old, still on anakinra treatment without adverse events.

**Conclusion:** Autoinflammatory diseases in the neonatal period are a diagnostic challenge. Clinical suspicion is crucial in order to perform specific laboratory and genetic testing and start appropriate treatment. Interstitial lung involvement may be present in MKD and, together with increased inflammatory markers, could be the first manifestation of the disease.

**Consent for publication has been obtained from patient**

Yes

**Disclosure of Interest**

None Declared

#### P2036 Familial Mediterranean fever related damage assessed by auto-inflammatory disease damage index (ADDI) and associated factors with damage

##### Hakan Babaoglu^1^, Berkan Armagan^2^, Erdal Bodakci^3^, Timucin Kasifoglu^3^, Hasan Satis^1^, Nuh Atas^1^, Alper Sari^2^, Gozde K. Yardimci^2^, Nazife S. Y. Bilge^3^, Reyhan B. Salman^1^, Levent Kilic^2^, Mehmet A. Ozturk^1^, Seminur Haznedaroglu^1^, Berna Goker^1^, Umut Kalyoncu^2^, Abdurrahman Tufan^1^

###### ^1^Department of Internal Medicine, Division of Rheumatology, Gazi University Faculty of Medicine; ^2^Department of Internal Medicine, Division of Rheumatology, Hacettepe University Faculty of Medicine, Ankara; ^3^Department of Internal Medicine, Division of Rheumatology, Eskisehir Osmangazi University Faculty of Medicine, Eskişehir, Turkey

####### **Correspondence:** Abdurrahman Tufan

**Introduction:** Familial Mediterranean Fever (FMF) is the most frequent auto-inflammatory disease caused by MEFV gene mutations. Although FMF is characterized by intermittent inflammatory attacks some patients exert chronic persistent inflammation that can result in damage of multiple organs. Available reports investigated only specific components of damage such as amyloidosis. All possible organ targets of damage have not been entirely evaluated before. Such as Disease severity index which is emerged especially for FMF do not cover entire damage domains related to FMF. Recently, a new scoring system (Auto-inflammatory disease damage index) was developed and validated for autoinflammatory diseases.

**Objectives:** to investigate damage accrual caused by FMF and associated features with damage.

**Methods:** All patients recruited from FMF in Central Anatolia (FiCA) cohort, which is a duplication disabled, internal and externally controlled, cross-sectional, multicenter accessible web-based cohort. This study is comprising 970 adult patients (mean age 35.3 ±12.1 years, 61.5% female). Demographic data, FMF disease characteristics, co-morbid conditions, disease complications were meticulously questioned and laboratory features and genotype data (if available) were recruited from patient files. FMF caused damage was assessed by auto-inflammatory disease damage index (ADDI) which is recently validated. Association between damage and demographic, disease and treatment characteristics were analyzed.

**Results:** Proportions of FMF manifestations were fever 83.1%, peritonitis 91.5%, pleuritis 47.9%, arthritis 43.3% and skin rash 26.2%. Dominant attack types were fever in 6.2%, serositis in 65.7%, musculoskeletal in 16.8% and all types of attacks were common in rest of patients. MEFV mutations were available in 814 subjects and 75.9% of these subjects were harboring M694V mutation (42.5% homozygous for M694V). Among all 63.1% patients were well responded to colchicine and 8.8% were non-responders. Median ADDI score was 1 (min 0-max 11). Most common FMF related damages were observed in musculoskeletal, reproductive and kidney domains. Chronic musculoskeletal pain was present in 49%, joint deformity in 2.9%, infertility in 6.6%, amenorrhea in 3.9%, proteinuria in 6.9%, amyloidosis in 5.9% and renal failure in 3.7% of the patients. 411 (%42.3) of patients had no damage accrual. M694V homozygous mutation, male gender and colchicine nonresponse were found to be the independent predictors of damage.

**Conclusion:** M694V homozygous mutation, colchicine non-response and male gender are predictors of damage and effective therapeutic interventions must be undertaken to prevent from damage in these patients.

**Disclosure of Interest**

None Declared

#### P2037 Familial Mediterranean fever associated inflammatory disorders

##### Nuh Ataş^1^, Berkan Armagan^2^, Erdal Bodakci^3^, Timucin Kasifoglu^3^, Hasan Satis^1^, Alper Sari^2^, Nazife S. Y. Bilge^3^, Hakan Babaoglu^1^, Gözde K. Yardımcı^2^, Reyhan B. Salman^1^, Levent Kilic^2^, Mehmet A. Ozturk^1^, Seminur Haznedaroglu^1^, Berna Goker^1^, Umut Kalyoncu^2^, Abdurrahman Tufan^1^

###### ^1^Department of Internal Medicine, Division of Rheumatology, Gazi University Faculty of Medicine; ^2^Department of Internal Medicine, Division of Rheumatology, Hacettepe University Faculty of Medicine, Ankara; ^3^Department of Internal Medicine, Division of Rheumatology, Eskisehir Osmangazi University Faculty of Medicine, Eskisehir, Turkey

####### **Correspondence:** Abdurrahman Tufan

**Introduction:** Familial Mediterranean fever (FMF) is an autosomal recessive hereditary autoinflammatory disease, characterized by recurrent self-limiting attacks of fever, serositis, arthritis and erysipelas-like skin eruption. Beside these, a substantial number of patients manifest with variety of inflammatory diseases probably due to predisposing effects of disrupted immune pathways in FMF. There is no systematic study investigating these conditions in FMF.

**Objectives:** The aim of this study is to determine prevalence of inflammatory conditions observed in patients with FMF and to define new potential inflammatory disorders.

**Methods:** All patients recruited from FMF in Central Anatolia (FiCA) cohort, comprising 970 (mean age 35.3±12 years, 61.5% female) adult subjects. All patients fulfilled Tel Hashomer criteria. Demographic data, FMF disease characteristics, co-morbid conditions, disease complications were meticulously questioned and laboratory features and genotype data (if available) were recruited from patient files. Data on inflammatory conditions were derived from patient interviews and hospital records.

**Results:** Inflammatory diseases were detected in 205 (21.1%) patients. Most common inflammatory disease was spondyloarthritis. Psoriasis, Henoch-Schönlein purpura, Behçet’s and inflammatory bowel diseases were other remarkable relevant disorders. Contact dermatitis and c*ryptogenic organizing pneumonia* were two newly defined entities in our cohort seem to be associated with FMF.

**Conclusion:** FMF is an inflammatory disease with short-lived/ self-limiting attacks. However, our results suggest that FMF is more than our apprehension which can also manifest with chronic conditions. In case of consistent symptoms, other potential FMF associated inflammatory diseases must be considered. Elaboration of pathogenic pathways linking these diseases to FMF warrant further investigations.

**Disclosure of Interest**

None Declared

#### P2038 Work productivity and activity impairment in familial Mediterranean fever patients

##### Erdem Suticen^1^, Nuh Atas^2^, Hakan Babaoglu^2^, Hasan Satis^2^, Reyhan B. Salman^2^, Ozkan Varan^2^, Mehmet A. Ozturk^2^, Seminur Haznedaroglu^2^, Berna Goker^2^, Abdurrahman Tufan^2^

###### ^1^Department of Internal Medicine; ^2^Department of Internal Medicine, Division of Rheumatology, Gazi University Faculty of Medicine, Ankara, Turkey

####### **Correspondence:** Abdurrahman Tufan

**Introduction:** Familial Mediterranean fever (FMF) is the most common monogenic autoinflammatory disease characterized with febrile attacks of serositis and arthritis. Although FMF can be observed at any age, it commonly affects children and young adults who are at most productive ages of the life. FMF has significant effects on work productivity and working life of patients as their quality of life.

**Objectives:** The aim of this study is to investigate work productivity and impairment in patients with FMF.

**Methods:** This study included 111 consecutive FMF patients who followed at the outpatient rheumatology clinic of Gazi University Hospitals. Demographic characteristics, the type of attacks, clinical and treatment features of FMF and work related data in last three months were recorded. Work productivity and activity impairment (WPAI) questionnaire was used for evaluation of the work productivity. Ten point visual analog scale was used for the determination of patient reported global disease activity assesment.

**Results:** The mean age of patients were 32.72±8.65 years and 54.1% were males. The proportion of FMF manifestations were peritonitis 87.4%, fever 83.8%, arthritis 75.7%, pleuritis 62.4 % and skin rash 32.4%. Sixty-five (58%) patients were on paid jobs. Absenteeism were evident in 12 of 65 subjects (18.4%). The median total lost days due to disease in last three months was 1 (range, 0-30) day. The median ratio of absenteeism was 22.4% (range, 2-100). The median presenteeism was 3 on a 0-10 point visual analog scale (range, 0-10). Disase activity assessed by patient global assesment was correlated with presenteeism (r=0.42, p<0.001) and absentteism (r=0.22, p=0.08).

**Conclusion:** Results of our study suggest that FMF is associated with work impairment which is more frequently observed as presenteeism (work productivity). FMF disease activity is remarkably associated with impairment in work productivity.

**Disclosure of Interest**

None Declared

#### P2039 Human phenotype ontology for systemic autoinflammatory disorders

##### Marielle Van Gijn^1^, Julia Pazmandi^2,3^, Paul Brogan^4^, Marco Gattorno^5^, Paul van Daele^6^, Kaan Boztug^2,3^, Peter Robinson^7^, Matthias Haimel^2,3^, Anna Simon^8^

###### ^1^Genetics, University Medical Center Utrecht, Utrecht, Netherlands; ^2^Ludwig Boltzmann Institute for Rare and Undiagnosed Diseases; ^3^CeMM Research Center for Molecular Medicine of the Austrian Academy of Sciences, Vienna, Austria; ^4^Infection Inflammation and Rheumatology Section, University College London Great Ormond Street Institute of Child Health, London, United Kingdom; ^5^Department of Pediatric Rheumatology, IRCCS G. Gaslini Institute, Genoa, Italy; ^6^Deparment of Internal medicine and department of Immunology, Erasmus MC, Rotterdam, Netherlands; ^7^The Jackson Laboratory for Genomic Medicine, Farmington, CT, United States; ^8^Department of Internal Medicine, Radboudumc Expertise Ccenter for Immunodeficiency and Autoinflammation, Radboud University Medical Center, Nijmegen, Netherlands

####### **Correspondence:** Marielle Van Gijn

**Introduction:** Systemic autoinflammatory diseases (SAID) are highly heterogeneous monogenic or multifactorial conditions whose onset is secondary to deregulation of mechanisms controlling the immune innate response. Molecular analysis is able to provide a definitive diagnosis in some patients with inherited SAID, but the results can be inconclusive. Bioinformatics can facilitate matching of phenotypically similar patients to enable gene discovery and the detection of new biomarkers. However, to allow efficient data exchange between clinicians and laboratories standardized clinical descriptions are necessary. Data structures such as the Human Phenotype Ontology (HPO) and disease ontologies such as the OrphaNet Rare Disease Ontology (ORDO) aim to provide standardized vocabularies of phenotypic abnormalities in human diseases, and can be used for this purpose. However, there are still crucial gaps in HPO and ORDO for SAID.

**Objectives:** A directed re-evaluation and correction of SAID-specific HPO and ORDO terms and ontology structures, as well as addition of new terms to enableuse of HPO in clinical practice.

**Methods:** In a 2-day workshop geneticists, medical doctors and bioinformaticians started to systematically re-evaluateSAID HPO and ORDO ontology terms, and re-annotate diseases. To continue this process, a working group was formed that will use text-mining from references from an international textbook on autoinflammation, and the Eurofever dataset to further optimizeand re-evaluate SAID HPO.

**Results:** So far, we have collected changes regarding the classification of autoinflammatory diseases to be submitted to ORDO. These include new disease terms such as “Autoinflammatory diseases without immunodeficiency”; and “NLRP3-associated autoinflammatory disease”, an umbrella term encompassing Muckle-Wells syndrome, CINCA, and Familial cold urticaria syndrome. So far, we have revised six sub-branches of the HPO to include a more accurate representation of autoinflammation-associated phenotypes, such as “increased inflammatory response”, “abnormality of temperature” and “tissue ischemia”. Moreover, we have successfully re-annotated 13 diseases entities with updated HPO terms.

**Conclusion:** Accurate metadata are increasingly needed to describe clinical information not only in a genetic manner, but also phenotypically. With the continued systematic re-evaluation of HPO for SAID, we expect to (i) standardize patient characterization so that clinicians/researchers can characterize patients in a language-independent manner; (ii) allow for efficient data exchange between clinicians, laboratories and centers; (iii) facilitate matching phenotypically-similar patients to enable gene discovery.

**Disclosure of Interest**

None Declared

#### P2040 Diagnosis and stratification of familial Mediterranean fever by a simple functional assay

##### Hanne Van Gorp^1^, Linyang Huang^1^, Pedro Saavedra^1^, Tomoko Asaoka^1^, Andy Wullaert^1^, Benson Ogunjimi^2^, Vito Sabato^2^, Joost Frenkel^3^, Fabrizio De Benedetti^4^, Joke Dehoorne^1^, Filomeen Haerynck^1^, Giuseppe Calamita^5^, Piero Portincasa^5^, Mohamed Lamkanfi^1^

###### ^1^Ghent University, Ghent; ^2^University of Antwerp, Antwerp, Belgium; ^3^University Medical Center Utrecht, Utrecht, Netherlands; ^4^Bambino Gesù Children’s Hospital, Rome; ^5^University of Bari, Bari, Italy

####### **Correspondence:** Andy Wullaert

**Introduction:** Familial Mediterranean Fever (FMF) is the most common monogenic autoinflammatory disease (AID) worldwide. The disease is caused by mutations in the *MEFV* gene encoding the inflammasome sensor Pyrin.

**Objectives:** Diagnosis of FMF is complicated by overlap in symptoms with other AIDs. Around 340 *MEFV* variants have been reported without clear genotype-phenotype links, thereby complicating interpretation of genetic testing. Therefore, we aimed to develop a functional test to shed more light on the deleterious effect of specific *MEFV* variants of the disease, and to aid in a more straightforward diagnosis.

**Methods:** In a prior study,^1^ we highlighted that in contrast to wild type Pyrin that requires microtubules to activate the inflammasome signaling pathway, inflammasome signaling by FMF-associated Pyrin variants is independent of microtubules. We used this differential microtubule requirement as a functional assay for diagnosing and stratifying FMF patients.

**Results:** Overall, our data nicely corroborate the recent consensus pathogenicity classification, though also provide potential new insights for specific variants. The assay is highly specific since related disorders (pyogenic arthritis, pyoderma gangrenosum, and acne (PAPA) and mevalonate kinase deficiency (MKD)) involving Pyrin inflammasome signaling are reliably segregated from FMF. The assay can be performed using purified peripheral blood mononuclear cells (PBMC) as well as a small volume of whole blood, providing a robust and versatile tool that paves the way for further research deciphering the molecular pathophysiological mechanisms underlying FMF, and, most importantly, diagnosis of FMF in the clinic.

**Conclusion:** To our knowledge, this microtubule dependency functional assay is the first one allowing diagnosis of FMF that enables functional/immunological screening of the disease among clinically overlapping auto-inflammatory patients and thus may contribute to timely FMF diagnosis and commencement of therapy.

**Reference**

1Van Gorp, H. *et al.* Familial Mediterranean fever mutations lift the obligatory requirement for microtubules in Pyrin inflammasome activation. *Proc Natl Acad Sci U S A* **113**, 14384-14389, doi:10.1073/pnas.1613156113 (2016).

**Disclosure of Interest**

None Declared

#### P2041 Evaluation of coexisting diseases in children with familial Mediterranean fever

##### Mehmet Yildiz, Amra Adrovic, Emre Tasdemir, Khanim Baba-Zada, Muhammed Aydin, Oya Koker, Sezgin Sahin, Kenan Barut, Ozgur Kasapcopur

###### Pediatric Rheumatology, Istanbul University, Cerrahpasa Medical School, Istanbul, Turkey

####### **Correspondence:** Mehmet Yildiz

**Introduction:** Familial Mediterranean Fever (FMF) is most common periodic fever syndrome in childhood. It is characterized by fever attacks, abdominal pain lasting between 6 - 72 hours, serositis and erysipelas like erythema. Since FMF is inherited in autosomal recessive manner, it has higher frequency in populations that have higher rate of consanguineous marriages. As it is a lifelong chronic disorder, it is important to understand its clinical course for preventive medicine. Despite the higher incidence of variety of disorders shown studies among adult FMF patient, there is not enough data from pediatric populations.

**Objectives:** The objective of the study is to evaluate the comorbid disorders in a large pediatric familial Mediterranean fever cohort.

**Methods:** Six hundred and eighty-six children with FMF were interviewed by the same investigator between October 2018 and January 2019 in our pediatric rheumatology department. Demographic features and MEFV gene mutations were recorded from patients’ charts. Patients and/or their parents were asked about characteristics of their fever episodes, presence of arthralgia, arthritis, abdominal pain, chest pain in the course of fever attack and coexistence of any other disease confirmed by a physician.

**Results:** Female-male ratio of our cohort was 0,85. Mean age of the patients was 12,8 ± 7,4 years. Mean age of patients at disease onset and at the time of diagnosis was 4,38 ± 3,46 years and 6,55 ± 3,67 years, respectively. Sixty-five and a half percent of patients had family history of FMF. Consanguineous marriage rate was 29,8 %. Most common MEFV mutations were: M694V homozygotes (18,8%), M694V heterozygotes (17,7%), R202Q heterozygotes (13,1%), R202Q homozygotes (5,5%) and V726A heterozygotes (4,9%), respectively. Resistance to colchicine treatment was present in 43 (6,2 %) patients. Number of patients that underwent tonsillectomy were 9 (1,3 %) and number of patients that had appendectomy was 11 (1,6 %). Detected coexisting diseases are listed in **Table 1**.

**Conclusion:** In this study, we have reported increased frequency of juvenile idiopathic arthritis, asthma- reactive airway disease, Henoch Schonlein purpura, uveitis, inflammatory bowel disease, PFAPA syndrome and acute rheumatic fever in a large pediatric FMF cohort. Those findings consistent with data from literature. It is important to be alert about these diseases that may develop in patients with FMF for preventing them from the potential morbidities.

**Disclosure of Interest**

None Declared

**Table 1 (abstract P2041).** Detected coexisting diseases in patients group

	n(%)
Juvenile Idiopathic Arthritis	40(5,8)
-·Systemic	4 (0,5)
- Oligoarticular	17(2,4)
- Polyarticular	5(0,7)
- Enthesitis Related Arthritis	1(0,1)
- Juvenile Spondylitis	13(1,8)
Asthma/ Reactive Airway Disease	26(3,7)
Henoch Schonlein Purpura	18(2,6)
Uveitis	10(1,4)
Inflammatory bowel disease	9(1,3)
Periodic fever, aphthous stomatitis, pharyngitis, cervical adenitis (PFAPA)	7(1)
Acute Rheumatic Fever	7(1)
Migraine	7(1)
Allergic Rhinitis	6(0,8)

#### P2042 Results of international Delphi survey on the investigation and management of first-degree relatives of those with deficiency of ADA2

##### Taryn A.-B. Youngstein^1^, Eugene P. Chambers^2^, on behalf of DADA2 Foundation, Helen J. Lachmann^1^, and DADA2 Delphi Study Participants

###### ^1^National Amyloidosis Centre, National Amyloidosis Centre, UCL Division of Medicine, London, London, United Kingdom; ^2^Vanderbilt University Medical Centre, Vanderbilt University, Nashville, United States

####### **Correspondence:** Taryn A.-B. Youngstein

**Introduction:** DADA2 is thought to be a recessive disease that is phenotypically heterogenous in its presentation, even within families with the same mutation.However, there have been numerous reports of disease in true heterozygotes.

**Objectives:** As part of a broader E-Delphi study to establish consensus on the diagnosis, investigations and management of those with DADA2, we explored current opinion and practice with respect to first-degree relatives of those with DADA2.

**Methods:** A Delphi method over two rounds of consensus building was used. Invitation email to participate in the study was sent to all published authors on DADA2 and physicians and scientists known to the DADA2 Foundation, a patient advocacy group supporting research into DADA2.Consensus was defined as > 80% agreement.

**Results:** 69 respondents contributed to Round 1 and 53 respondents in Round 2. 75% of respondents were paediatricians, the rest adult physicians, a nurse specialist and basic scientists.

88% of respondents would investigate asymptomatic first-degree relatives with genetic testing.39% would refer formally for genetic counselling and 6% have counsellors in their clinic consultations.

62.8% of respondents believe that true heterozygotes can have DADA2, and screen for low ADA2 enzyme activity to make the diagnosis (66%).Treatment (anti-TNF) would only be started in those who were symptomatic (defined by stroke (80%), digital gangrene (64.6%), livedoid rash (49.2%) and cytopaenias (43.8%)). If asymptomatic, 65% would follow-up carriers (heterozygote or homozygote), and 13.6% would only follow-up those who also had low ADA2 activity.

74.5% screened for hypertension in carriers, 64.7% would treat borderline hypertension.89.7% would not anti-coagulate asymptomatic carriers, 50.7% would not give anti-platelet therapy.Statins would only be given if indicated by raised fasting lipids.50% perform follow-up investigations such as testing inflammatory markers, 73.9% do not perform any surveillance imaging (such as MRI Brain) in asymptomatic carriers. There was a wide range of duration of follow-up visits, from monthly to annually, with 19.9% only reviewing if symptoms evolved.

**Conclusion:** There is consensus, after two rounds, that first-degree relatives should be screened for mutations in the ADA2 gene.Asymptomatic carrier status is recognised with homozygotes and compound heterozygous.A majority also recognise a true heterozygote disease state.The measurement of serum ADA2 activity levels is believed to be helpful inrisk stratification but investigations and treatment are currently largely guided by symptoms.

Measures are not in place to control traditional cardiovascular risk factors beyond those employed in the general population.These data raise many questions and suggest the need for an international case registry and a standardised approach to the investigation of first-degree relatives.

**Disclosure of Interest**

None Declared

### Non-monogenic SAIDs (clinical)

#### P2043 We need more tests for discrimination non-bacterial osteomyelitis form tuberculosis in early stages

##### Mikhail Kostik^1^, Olga Kopchak^2^, Alexey Maletin^3^, Vyacheslav Zorin^3^, Alexander Mushkin^3^

###### ^1^Saint-Petersburg State Pediatric Medical University, Saint-Petersburg; ^2^Kirov’s Regional Children’s Hospital, Kirov; ^3^Science Research Institute of Phthisiopulmonology, Saint-Petersburg, Russian Federation

####### **Correspondence:** Mikhail Kostik

**Introduction:** Non-bacterial osteomyelitis (NBО) and tuberculous osteitis (osteomyelitis, TBO) are two primarily chronic inflammatory bone diseases with similar clinical and radiological findings, but different in etiology, pathogenesis, treatment, and outcomes.

**Objectives:** The aim of the study is to determinate clinical and laboratory features which could discriminate the NBO and TBO.

**Methods:** The following criteria for inclusion in the retrospective-prospective study: i) patient’s age under 18 years old; ii) bone lesions, confirmed by any radiological methods (X-ray films or CT or MRI); iii) inflammatory bone process confirmed by morphology study; iv) bone lesions biopsy followed by bacteriological and morphology research. In the cases of multifocal NBO, the biopsy had to be done at least from one bone focus; v) positive cultural (MBT complex isolates) or molecular genetics (PCR) or GEEN-expert studies from bone lesion site biopsy or operation material for TBO group. Exclusion criteria: i) positive culture for non-MBT mycobacteriosis from bone lesion site biopsy or operation material for TBO; ii) the negative cultural or PCR tests in the cases of granulomatous osteomyelitis which could be suspected on TB or for patients who had received at least two courses of antibacterial drugs. The study included 124 patients - 91 with NBO and 33 with TBO. All patients underwent a routine blood test: WBC, platelets, ESR, C-reactive protein (CRP) and hemoglobin levels. We compare our results with criteria created by A. Jansson et al. (2009, 2011) and Roderick et al. (2016).

**Results:** Logistic regression analysis performed by including the 15 independent variables that presented ahigh level of statistical significance in the univariate analyses. The choice of the variables in this model performed according to the clinical plausibility for a diagnosis of TBO. Only number of foci > 1.0 (p=0.00002), WBC≤11.0 (p=0.004), bands ≤ 120.0 х 10^6^/l (p=0.002), lymphocytes ≤ 52 % (p=0.0005) and CRP> 0.2 mg/l (p=0.003) remained independent risk factors that distinguished NBO from TBO patients. The R2 of the Nagelkerke test was 0.66. The diagnostic rule formulates according to sensitivity, specificity and DOR analysis: criteria allowing to differentiate non-bacterial osteomyelitis from TB osteomyelitis are negative bone microbiota tests (sensitivity – 100.0%, specificity – 100.0%) – major discriminative criteria or at least four from the five additional criteria. These criteria could be useful at the stages when histological and bacteriological results have not yet ready or if it is impossible to do they. Applying of NBO diagnostic tests may misdiagnose TBO as NBO (table).

**Conclusion:** We offer to use our criteria only as additional diagnostic set in combination with all previously created tests. Further investigations required for the creation of more sensitive and specific tests. It is necessary to have high suspicion about TBO and biopsy needed at least in all monofocal cases.

This work supported by the Russian Foundation for Basic Research (grant № 18-515-57001).

**Disclosure of Interest**

None Declared

**Table 1 (abstract P2043).** Diagnostic power, sensitivity, and specificity of NBO diagnostic sets

The set of criteria	The maximal possible number of patients with TBO, who may be false-diagnosed as NBO, n (%)	RR (95%CI)	Se	Sp
Jansson, 2009	29/33 (87.9)	0.14 (0.06-0.35)	0.12	0.12
Jansson 2011	7/33 (21.2)	3.7 (1.9-7.3)	0.79	0.79
Roderick, 2016	5/33 (15.2)	5.6 (2.5-12.7)	0.85	0.85
Our criteria	For NBO			
Major criteria	0 (100.0)	na	1.0	1.0
≥2 minor criteria	32/91 (35.2)	1.84 (1.3-2.5)	0.65	0.65
≥3 minor criteria	11/91 (12.1)	7.3 (4.2-12.7)	0.88	0.88
≥4 minor criteria	0/91 (0.0)	na	1.0	1.0

#### P2044 The features and distribution of chronic non-bacterial osteomyelitis in Russian Federation

##### Mikhail Kostik^1^, Maria Makhova^1^, Vyacheslav Zorin^2^, Evgeny Suspitsin^1,3^, Eugenia Isupova^1^, Shamai Magomedova^4,5^, Inna Kostik^6^, Alexander Mushkin^2^

###### ^1^Saint-Petersburg State Pediatric Medical University; ^2^Science research Institute of Phthisiopulmonology; ^3^N.N. Petrov Institute of Oncology, Saint-Petersburg; ^4^Republican Children's Clinical Hospital; ^5^Dagestan State Medical Academy, Makhachkala; ^6^Children's Rehabilitation Center “Detskye Duny”, Saint-Petersburg, Russian Federation

####### **Correspondence:** Mikhail Kostik

**Introduction:** Data about the incidence and prevalence of chronic non-bacterial osteomyelitis (CNO) in Russia is scarce.

**Objectives:** Our study aimed to evaluate the clinical features and prevalence of CNO in Russia.

**Methods:** The diagnosis of CNO made with criteria, proposed by Jansson (2007, 2009), after the exclusion of other causes of bone disease. Our cohort consists of three main subtypes: i) early-onset (<5 years) CNO (n=17); ii) CNO, associated (n=20) and iii) not associated (n=59) with rheumatic diseases (RD).

**Results:** The patients with early-onset (<5 years) CNO characterized by 1) early onset; 2) all children were initially diagnosed as having tuberculosis (TB) due to bone morphology findings (granulomatous, e.g., tuberculosis-like inflammation), but had a negative TB culture test; 3) initial treatment with combination of 3-4 anti-MBT drugs during 1-2 years was ineffective, patient continued to form new inflammatory bone foci; 4) patients had more severe clinical and radiological signs of disease, compared to others and 5) all patients have North Caucasus origin (area with high prevalence of consanguinity). These patients have earlier onset age, more foci number, a high incidence of symptomatic arthritis, femur and foot involvement (table 1).

**Conclusion:** We have found the unique regional subtype of CNO in North Caucasus region with at least nine times higher prevalence. We have not yet seen similar patients in other nationalities of Russia.

This work supported by the Russian Foundation for Basic Research (grant № 18-515-57001)

**Disclosure of Interest**

None Declared

**Table 1 (abstract P2044).** Characteristic of clinical and laboratory features of patients with CNO

Parameter	EO-CNO (n=17)	“CNO w/o RD” (n=59)	CNO with RD (n=20)	p_1_
Onset age, years	3.0 (2.1-4.8)	7.3 (2.8-11.7)	10.3 (8.2-12.2)	**0.0009**
Gender, females	8 (47.1)	27 (45.8)	14 (70.0)	0.17
Fever at onset	9/16 (56.3)	23 (39.0)	5 (25.0)	0.16
Foci number	5.0 (1.5-6.0)	3.0 (1.0-4.0)	2.0 (1.0-6.0)	**0.048**
Symptomatic arthritis	15/16 (93.8)	33 (55.9)	17 (85.0)	**0.003**
North Caucasus origin	17 (100.0)	0 (0.0)	0 (0.0)	**<0.00001**
Granulematosus inflammation (tuberculosis-like)	17 (100.0)	0 (0.0)	0 (0.0)	**<0.00001**
Prevalence of CNO	1: 55,000	1:450,000	1:1,375,000	**<0.00001**

#### P2045 Kawasaki disease shock syndrome: clinical characteristics and possible use of IL-6, IL-10 and IFN-gamma as biomarkers for early recognition

##### Meiping Lu^1^, Yandie Li^2^, Qi Zheng^2^

###### ^1^Zhejiang University; ^2^Children’s Hospital of Zhejiang University, Hangzhou, China

####### **Correspondence:** Meiping Lu

**Introduction:** As an acute febrile and inflammatory disease, Kawasaki disease (KD) could develop Kawasaki disease shock syndrome (KDSS) sometimes. However its pathogenesis was still not well known.

**Objectives:** This study was to learn more about the clinical features and evaluate the role of cytokines in the pathogenesis of KDSS.

**Methods:** All 27 patients with KDSS who were hospitalized at our hospital from Jan 2014 to Oct 2017 were enrolled in our study retrospectively. Clinical features, laboratory evaluations including white blood cell (WBC) count, C-reactive protein (CRP), Erythrocyte Sedimentation Rate (ESR), procalcitonin (PCT), liver and kidney function, blood coagulation function, echocardiographic data, cytokine levels and outcomes were collected. Cytokines IL-2, IL-4, IL-6, IL-10, TNF-α and IFN-γ in serum were assayed using flow cytometric bead array.

**Results:** 2203 patients with KD were seen in our hospital during the study period. Of those, 27 (1.23%) met our inclusive criteria of KDSS. The patients with KDSS were older age (43.41±31.42 vs 28.81±21.51,p<0.05), longer duration of fever(10.63±5.12 vs 6.98±2.45, p<0.05), higher WBC count, neutrophils, CRP, ESR, PCT and D-dimer, and lower hemoglobin and albumin, more severe hyponatremia and hypokalemia, more refractory to IVIG therapy, more coronary artery abnormalities (CAAs), aseptic meningitis, and longer duration of hospitalization than patients with KD. There was higher level of serum IL-6, IL-10, TNF-α and IFN-γ in patients with KDSS than patients with KD. ROC curves showed that 66.7 pg/ml of IL-6, 20.85 pg/ml of IL-10 and 8.35 pg/ml of IFN-γ had sensitivity and specificity for indentifying KDSS as 85.2% and 62.8 %; 66.7% and 83.7%; 74.1% and 74.4 % respectively. No fatality was recorded in this series.

**Conclusion:** Increased production of IL-6, IL-10, TNF-α and IFN-γ cytokines may pay a key role in the pathogenesis of KDSS. KDSS were more prone to developing CAA and IVIG non-responsiveness. IL-6 above 66.7 pg/ml, IL-10 above 20.85 pg/ml, and IFN-γ above 8.35 pg/ml suggested higher risk for KDSS.

**Disclosure of Interest**

None Declared

#### P2046 Outcomes following tonsillectomy in patients with periodic fever, aphthous stomatitis, pharyngitis, and adenitis (PFAPA) syndrome

##### Kalpana Manthiram^1^, Sivia Lapidus^2^, Karyl Barron^3^, Amanda Ombrello^1^, Daniel Kastner^1^, Kathryn Edwards^4^

###### ^1^National Human Genome Research Institute, NIH, Bethesda; ^2^Hackensack University Medical Center, Hackensack; ^3^National Institute of Allergy and Infectious Diseases, NIH, Bethesda; ^4^Vanderbilt University School of Medicine, Nashville, United States

####### **Correspondence:** Kalpana Manthiram

**Introduction:** Tonsillectomy has been reported to induce complete resolution of fever episodes in patients with periodic fever, aphthous stomatitis, pharyngitis, and adenitis (PFAPA) syndrome. However, predictors of lack of resolution and management of continued episodes after tonsillectomy are not well understood.

**Objectives:** To determine the response to tonsillectomy in subjects with PFAPA, clincial features associated with lack of response to tonsillectomy, and subsequent management in subjects who did not respond to tonsillectomy.

**Methods:** Patients from Vanderbilt Children’s Hospital and the National Institutes of Health (NIH) who met the original diagnostic criteria for PFAPA and underwent tonsillectomy were enrolled and interviewed about their symptoms before and after tonsillectomy. Clinical features of patients who had complete and incomplete resolution of symptoms after tonsillectomy were compared with the chi-square test.

**Results:** Sixty-four patients (41 from Vanderbilt and 23 from NIH) were followed for an average of 61 months after tonsillectomy. In total, 48% of participants reported complete resolution of PFAPA episodes (63% had full resolution at Vanderbilt and 22% at NIH), while 6% subsequently had only one or two more PFAPA episodes, 22% had a reduction in severity or frequency of episodes, 17% had a period of remission ranging from 12 months to 7 years with recurrence of stereotypical episodes, and 6% had no change in episodes following tonsillectomy. The table shows the percentage of patients with and without each feature during flares who had complete resolution of episodes after tonsillectomy; episode shortening with corticosteroids and absence of abdominal pain and limb pain were predictive of complete resolution. In the multivariable model, episode shortening with corticosteroids (aOR=12.5, p=0.04) and lack of limb pain (aOR=0.11, p=0.001) remained significant predictors of complete response to tonsillectomy. Twenty patients (61% or 20/33) with continued febrile episodes after tonsillectomy required medical intervention. In these patients, 7 out of 12 (58%) had resolution of each episode with one dose of corticosteroid, and 63% (5/8) had resolution of each episode with one dose of anakinra while 37% (3/8) required multiple doses of anakinra during each flare. After tonsillectomy, 86% of patients (6/7) who took cimetidine prophylaxis had improvement in the severity or frequency of flares compared to 44% (4/9) of those who took colchicine prophylaxis.

**Conclusion:** Most patients with PFAPA had an improvement in the frequency and severity of symptoms following tonsillectomy. Differences in outcome at Vanderbilt and NIH likely reflect referral bias as patients seen at NIH are more likely to be refractory to conventional therapies. Patients with abdominal pain, limb pain and lack of episode resolution with steroids were less likely to respond completely to tonsillectomy. These patients may have lymphoid tissue outside the palatine tonsils that triggers PFAPA episodes or may have alternate mechanisms of disease. Cimetidine was an effective prophylactic agent in patients with episodes following tonsillectomy, indicating that further study of the effect of cimetidine on myeloid and lymphoid cells is needed.

**Disclosure of Interest**

None Declared

**Table 1 (abstract P2046).** See text for description

Characteristic	% without feature who had complete resolution after tonsillectomy	% with feature with complete resolution after tonsillectomy	P value
Aphthous ulcer	53.3%	46.9%	0.67
Pharyngitis	40.0%	49.2%	1.00
Lymphadenopathy	63.6%	45.3%	0.27
Headache	45.5%	51.6%	0.62
Abdominal pain	**60.6%**	**35.5%**	**0.04**
Vomiting	51.3%	44.0%	0.57
Limb pain (arthralgia or myalgia)	**70.6%**	**23.3%**	**<0.001**
Episode shortens with steroid	**12.5%**	**53.5%**	**0.03**

#### P2047 Relationship between MEFV gene and clinical findings of chronic recurrent multifocal osteomyelitis

##### Sümeyra Özdemir Çiçek^1^, Nihal Şahin^1^, Zehra F. Karaman^2^, Sema N. Taşkın^1^, Ayşenur Paç Kısaarslan^1^, Zübeyde Gündüz^3^, Muammer H. Poyrazoğlu^1^, Ruhan Düşünsel^1^

###### ^1^Pediatric Rheumatology; ^2^Pediatric Radiology, Erciyes University Medical Faculty; ^3^Pediatric Rheumatology, Acıbadem Hastanesi, Kayseri, Turkey

####### **Correspondence:** Sümeyra Özdemir Çiçek

**Introduction:** Chronic recurrent multifocal osteomyelitis (CRMO) is an inflammatory disorder that characterized byrecurrent flares and remissions due to sterile bone inflammation. CRMO may be accompanied by different autoinflammatory diseases.

**Objectives:** To determine CRMO patients’ features, involvement patterns, clinical course and treatment followed in our pediatric rheumatology clinic.

**Methods:** Seventeen patients who were diagnosed of CRMO and followed in our clinic between 2012-2018 were analyzed retrospectively.

**Results:** The mean age of symptoms onset was 10,41 (±3,79 ) years, and the mean age at diagnosis was 11,36 (±3,68) years. Between time of symptoms onset and diagnosis was 5 months (min-max:1,5-55), The disease duration was 25,82 (±17,26 ) months. Ten (58,8%) patients were male. The lesions were evaluated by regional MRI on 8 patients, whole body MRI on 9 patients, and skeletal scintigraphy on 9 patients. Bone biopsy was performed in 6/17 (35,29%) patients. The most affected areas were femur (70,58%), tibia (58,82%) and vertebra (47,05%). A total number of lesions were 110 during the disease course. The mean number of affected areas was 6,47 (±4,04 ) per patient. Three of the patients had skin signs and five of patients had aphthous stomatitis. Six (35,29%) patients had concomitant familial Mediterranean fever (FMF) and four of them had M694V homozygous mutations. Compared to those who carried MEFV mutation and not carriers, time of symptomes onset, disease duration, number of affected areas, clinical and radiological remission, sacroiliitis, C-reactive protein and erithrocyte sedimentation rate at the beginning of disease were not significant differencies statistically. The treatments were NSAI drugs on 16 (94,11%), colchicine on 7 (41,17%), DMARDs on 8 (47,05%), and biologic DMARDs on 6(35,29%) patients. Six of the patients were receiving colchicine treatment due to fmf, and one of patients due to recurrent oral aphthae.

**Conclusion:** Previous studies have shown that CRMO is associated with various inflammatory diseases. CRMO patients should be evaluated for MEFV gene carriage.

**Disclosure of Interest**

None Declared

#### P2048 Behçet’s disease with pseudotumor cerebri and cerebral venous sinus thrombosis: a case report

##### Sümeyra Özdemir Çiçek^1^, Nihal Şahin^1^, Sema N. Taşkın^1^, Ayşenur Paç Kısaarslan^1^, Zübeyde Gündüz^2^, Muammer H. Poyrazoğlu^1^, Ruhan Düşünsel^1^

###### ^1^Pediatric Rheumatology, Erciyes University Medical Faculty; ^2^Pediatric Rheumatology, Acıbadem Hastanesi, Kayseri, Turkey

####### **Correspondence:** Sümeyra Özdemir Çiçek

**Introduction:** Behçet’s disease (BD) is a vasculitis that can affect arteries or veins of any size. BD manifests with the clinical triad of aphthous stomatitis, genital ulceration and uveitis. Neurologic involvement may be parenchymal or nonparenchymal. The non parenchymal involvement is usually a benign hypertension with papilledema and is often due to venous sinus thrombosis. Here we present a BD patient admitted with pseudotumor cerebri and appeared venous sinus thrombosissix months later on his follow up.

**Objectives:** A case report

**Methods:** a case report

**Results:** A 16-year-old boy with BD for 3-years admitted redness of eye, blurred vision, progressive headache for seven days and nausea, vomiting for two days. He was on colchicine theraphy for three years. On physical examination, he had oral and genital ulcers. On the eye excamination he had not uveitis or papilledema. He had sixth cranial nerve palsy on the right eye. Cranial MRI showed pseudotumor cerebri signs .There were not any parencimal lesions or venous thrombosis.The cerebrospinal fluid pressure was detected 40 cmH_2_O. Protein, glucose, and cell levels of CSF were normal. A lumbar puncture performed to reduce intracranial pressure. Corticosteroid and acetazolamide treatment were started. His clinical findings improved with this theraphy, his oral and genital ulcers healed. After being discharged he didn’t come his routine controls and didn’t use his medications. Five mounths later he presented with headache and blurred vision. His eye excamination revealed papilledema and he had bilateral nasal scotoma. MR angiography revealed irregularity on bilateral carotis interna and branches, and MR venography revealed occlusions on left transvers, sigmoidal and right transvers sinuses. Enoxaparin, steroid and azathioprin treatment was started. Three months later control MR venography showed improvement in venous sinuses thromosis but his increased intracranial pressure didn’t improve. İnfliximab treatment was initiated and follow-up continued.

**Conclusion:** The most common neurologic manifestation is cerebral venous sinus thrombosis in children with BD. Pseudotumor cerebri is one of the Behçet’s disaese’s neurologic involvement that needed rapidly invention. It occurs with or without venous sinus thrombosis

**Consent for publication has been obtained from patient**

Yes

**Disclosure of Interest**

None Declared

#### P2049 The use of next generation sequencing panel in undifferentiated autoinflammatory diseases identify a separate subset of colchicine-responder recurrent fevers distinct from PFAPA syndrome

##### Riccardo Papa^1^, Marta Rusmini^2^, Stefano Volpi^1^, Roberta Caorsi^1^, Paolo Picco^1^, Alice Grossi^2^, Francesco Caroli^2^, Francesca Bovis^3^, Valeria Musso^1^, Laura Obici^4^, Cinza Castana^5^, Angelo Ravelli^1^, Marielle E. Van Gijn^6^, Isabella Ceccherini^2^, Marco Gattorno^1^

###### ^1^Pediatric Rheumatology Clinic; ^2^Medical Genetics Unit, IRCCS Giannina Gaslini Institute; ^3^Department of Health Sciences (DISSAL), University of Genoa, Genova; ^4^Amyloidosis Research and Treatment Centre, Biotechnology Research Laboratories, IRCCS Fondazione Policlinico San Matteo, Pavia; ^5^Pediatric Clinic, ARNAS Civico Di Cristina Benfratelli, Palermo, Italy; ^6^Department of Medical Genetics, University Medical Center Utrecht, Utrecht, Netherlands

####### **Correspondence:** Riccardo Papa

**Introduction:** The number of monogenic innate immune system disorders classified as systemic autoinflammatory diseases (SAID) has increased during the recent years. However, more than 70% of patients with clinical manifestations of SAID not achieved a molecular diagnosis, thus getting into the so-called undifferentiated or undefined SAID (uSAID).

**Objectives:** The aim of the present study is to characterize a subgroup of patients with recurrent fever episodes distinct to PFAPA that turned out to be negative to a large 41-gene NGS panel.

**Methods:** We designed an NGS panel including 41 genes clustered in seven subpanels. The clinical characteristics of genetically negative patients with recurrent fevers were compared to different PFAPA cohort reported in the literature.

**Results:** Fifty patients were enrolled in the study. 34 patients (72%) displayed recurrent fevers and sixteen presented a chronic inflammatory disease course. A total of 100 gene variants were found (mean 2 per patient; range 0-6). Mutations with a sure or possible diagnostic impact were detected in five patients (10%). Differently from PFAPA syndrome (table), genetically negative patients with recurrent fevers presented episodes that lasted on averange of six days (P<0.0001). Abdominal pain and limb pain were the most common symptoms. The classic PFAPA triad (pharingotonsillitis, apthousis and cervical lymphadenopathy) was less frequently reported (P<0.0001) while skin rash and arthritis were more frequent (P<0.0001). Eighteen patients were exclusively treated with steroid on demand with a high response rate (94%). In 18 patients, colchicine treatment was used with an overall complete or partial response of 78%.

**Conclusion:** Even with a low molecular diagnostics rate, an NGS-based approach is able to provide a final diagnosis in a proportion of uSAID patients. It also allows the identification of a subgroup of genetically negative patients with recurrent fever responding to steroid on demand and colchicine.

**Disclosure of Interest**

None Declared

**Table 1 (abstract P2049).** Clinical features of patients with undifferentiated recurrent fevers compared to PFAPA syndrome

Clinical features	Our cohort (34 patients)	Thomas et al. (82 patients)	Hofer et al. (301 patients)	Batu et al. (131 patients)	Pehlivan et al. (359 patients)	P value
Mean duration of episodes (days; ±SD)	5.9 ± 1.4	NR	NR	NR	4.0 ± 3.1	<.0001
Median interval between episodes (weeks; ±SD)	3.0 ± 0.3	NR	NR	NR	3.3 ± 1.5	NS
Abdominal pain	17 (50)	40 (49)	136 (45)	60 (46)	102 (29)	NS
Arthritis	7 (21)	NR	8 (3)	NR	7 (0)	<.0001
Skin rash	11 (32)	7 (9)	38 (13)	7 (5)	NR	<.0001
Pharyngotonsillitis	13 (38)	59 (72)	271 (90)	126 (96)	359 (100)	<.0001
Cervical lymphadenopathy	6 (18)	72 (88)	236 (78)	70 (53)	215 (60)	<.0001
Oral aphthosis	13 (38)	57 (70)	171 (57)	56 (43)	317 (88)	<.0001
Response to colchicine	14/18 (78)	0/1 (0)	NR	5/11 (45)	24/45 (53)	NS

Values are number of patient (%) when not specified. NR = not reported. NS = not significant. P values were assessed using Chi square test or T-test as appropriate. If significant interactions were determined, a post-hoc test for multiple comparison was performed

#### P2050 Epidemiology and clinical features of PFAPA in Western Sweden

##### Karin Rydenman^1,2^, Hanna Fjeld^3^, Anna Karlsson^4^, Stefan Berg^1,5^, Anders Fasth^1,5^, Per Wekell^1,2^

###### ^1^Department of Pediatrics, Institute of Clinical Sciences, Sahlgrenska Academy, University of Gothenburg, Gothenburg; ^2^NU Hospital Group, Uddevalla; ^3^Örebro University, Örebro; ^4^Department of Rheumatology and Inflammation Research, Institute of Medicine, Sahlgrenska Academy, University of Gothenburg; ^5^Queen Silvia Children's Hospital, Gothenburg, Sweden

####### **Correspondence:** Karin Rydenman

**Introduction:** Periodic fever, aphtous stomatitis, pharyngitis and cervical adenitis (PFAPA) syndrome is generally seen as the most common autoinflammatory disease, but the epidemiology of the disease is largely unknown. In a Norwegian study, the annual incidence was calculated to 2.3/10000 children <5 years of age (1). In Sweden and other parts of the world, the incidence has not been studied.

**Objectives:** To estimate the annual incidence and describe the clinical features of PFAPA in Western Sweden.

**Methods:** Children <18 years of age diagnosed with PFAPA during the years 2006-2017 and living in the area of Western Sweden served by the two hospitals NU Hospital Group and Queen Silvia Children’s Hospital were included retrospectively. Patients were identified through search for relevant diagnostic codes in the comprehensive electronic medical record (Melior, Cerner Sweden) and data was obtained through review of case records. For estimation of incidence, patients with onset of symptoms 1^st^ of Jan 2006 to 31^st^ of Dec 2016 were included. Population data for the study area during this period of time was retrieved from Statistics Sweden (2).

**Results:** In our study, a total of 236 patients diagnosed with PFAPA were identified. Of these, 122 (52%) were girls and 112 (48%) were boys (no data on 2 patients). Frequency of typical symptoms during fever episodes is depicted in table 1. Data on CRP during episodes was obtainable for 80 patients, with a median value of 75 (range 12-358) mg/L. Data on CRP between episodes was obtainable for 96 patients and all of these patients except two had a CRP <5 mg/L. The remaining two had a CRP of 7 and 17 mg/L, respectively. Treatment was symptomatic in most patients with acetaminophen and/or ibuprofen. 120 patients received betamethasone to abort single febrile episodes, however patients were advised to use the drug only on rare occasions due to concerns of side-effects. Tonsillectomy was performed on 73 patients.

For calculation of incidence, 216 patients with onset of symptoms 1^st^ of Jan 2006 to 31^st^ of Dec 2016 were identified and of these 171 had disease onset <5 years of age. The mean annual incidence was estimated to 0.8/10000 for children <18 years of age and 2.3/10000 children <5 years of age respectively.

**Conclusion:** The mean annual incidence of PFAPA (2.3/10000 children <5 years of age) was in the same range as estimated by Forsvoll et al (1). Our study also suggests an annual incidence of 0.8/10000 for PFAPA in children up to 18 years of age. Clinical features were similar to those seen in the Eurofever registry (3). However, there was a slight female predominance among patients in this study in contrast to previous studies. Tonsillectomy was a common treatment option in this cohort.

1. Forsvoll J, Kristoffersen EK, Oymar K. Incidence, clinical characteristics and outcome in Norwegian children with periodic fever, aphthous stomatitis, pharyngitis and cervical adenitis syndrome; a population-based study. Acta Paediatr. 2013;102(2):187-92.

2. Statistics Sweden. Retrieved 14th of Jan 2019 from http://pxwebb2017.vgregion.se/PXWeb/sq/618a266b-d273-4923-8f86-01f6c7bb1a84

3. Hofer M, Pillet P, Cochard MM, Berg S, Krol P, Kone-Paut I, et al. International periodic fever, aphthous stomatitis, pharyngitis, cervical adenitis syndrome cohort: description of distinct phenotypes in 301 patients. Rheumatology (Oxford). 2014;53(6):1125-9.

**Disclosure of Interest**

None Declared

**Table 1 (abstract P2050).** Frequency of typical symptoms during PFAPA episodes (% of patients)

Symptom	Always	Sometimes	Never	Data missing	Total
Pharyngitis	151 (64)	64 (27)	2 (0.8)	19 (8)	236
Aphthous stomatitis	22 (9)	83 (35)	57 (24)	74 (31)	236
Cervical adenitis	114 (48)	62 (26)	8 (3)	52 (22)	236

#### P2051 Observations in a patient with PFAPA-like disease associated with erysipelas-like erythema and R202Q variant in MEFV

##### Per Wekell^1,2^, Mia Olsson^3^, Peter Söderkvist^4^, Anna Karlsson^5^, Stefan Berg^2^, Anders Fasth^2^

###### ^1^Department Pediatrics, NU-Hospital Group, Uddevalla; ^2^Department of Pediatrics, Institute of Clinical Sciences, Sahlgrenska Academy, University of Gothenburg, Gothenburg; ^3^Department of Medicine, Center for Molecular Medicine, Karolinska Institute, Stockholm; ^4^Department of Clinical and Experimental Medicine, Linköping University, Linköping; ^5^Department of Rheumatology and Inflammation Research, Institute of Medicine, Gothenburg, Sweden

####### **Correspondence:** Per Wekell

**Introduction:** The genetic predisposition to develop periodic fever, aphthous stomatitis, pharyngitis, and cervical adenitis (PFAPA) syndrome is not known, but variants in NLRP3 inflammasome proteins such as NLRP3 and CARD8 has been proposed to contribute. PFAPA has also been associated with an increased frequency of variants in the gene for familial Mediterranean fever (*MEFV*) indicating that such variants may modify the disease expression in PFAPA.

**Objectives:** To investigate if there is a genetic basis for erysipelas-like erythema (ELE) presented in a child with a PFAPA-like picture.

**Methods:** Genetic variants in the classical periodic fever genes *MVK*, *MEFV*, *TNFRSF1A*, *NLRP3* and *NLRP12* were analyzed.

**Results:** We report, a 9-year-old girl of Scandinavian origin with a PFAPA-like presentation and an ELE considered to be pathognomonic for FMF. No established disease-causing variants was found in the classical periodic fever genes depicted above. The patient was heterozygous for several single nucleotide polymorphisms (SNPs): S52N (rs7957619) in MVK, R202Q (rs224222) in MEFV and Q703K/Q705K (rs35829419) in NLRP3.

**Conclusion:**

If we consider PFAPA a complex disease, its etiology would be a combination of rare and common genetic factors providing a propensity for disease in response to (unknown) environmental trigger(s). In the present patient, the two low-penetrance variants Q703K and R202Q, could provide such a combination that contributes to her typical and atypical clinical PFAPA features in response to a, e.g., tonsillar trigger.

Furthermore, the patient’s heterozygosity for R202Q with a PFAPA-like presentation including ELE, support the concept that MEFV variants in general and R202Q in particular may have pathophysiological effects as disease modifier(s) in PFAPA.

**Consent for publication has been obtained from patient**

Yes

**Disclosure of Interest**

None Declared

#### P2052 YAO syndrome versus familial Mediterranean fever

##### Qingping Yao^1^, Peter Gorevic^2^

###### ^1^Medicine, Stony Brook University, Stony Brook; ^2^Medicine, Mount Sinai Hospital, NY, United States

####### **Correspondence:** Qingping Yao

**Introduction:** Autoinflammatory diseases include monogenic periodic fever syndromes and genetically complex diseases. Familial Mediterranean fever (FMF) is a prototypic monogenic periodic fever syndrome. Yao syndrome (YAOS, OMIM 617321), formerly termed NOD2-asssociated autoinflammatory disease, is considered to be polygenic disease. YAOS is characterized by periodic fever, dermatitis, arthritis, and swelling of the distal extremities, as well as gastrointestinal and sicca-like symptoms and linked to certain *NOD2* variants. These diseases share the overlapping clinical phenotypes, which may cause diagnostic dilemma in the absence of genetic testing.

**Objectives:** To illustrate the similarities and differences between YAOS and FMF.

**Methods:** A case is exemplified coupled with the literature review.

**Results:** A 66-year-old Caucasian female presented with recurrent episodes of high fever, abdominal pain, rash and arthralgia for three years. Typically, the patient experienced flu-like symptoms followed by fever of 101 to 103 ^0^F with chills lasting up to 36 hours. Abdominal pain was generalized with non-bloody diarrhea often lasting for four days and occasionally several months. She reported a nonitchy patchy erythema on her face and chest, lasting up to two weeks with each episode (**Figure 1**). She also had pain and swelling in her knees and ankles, diffuse myalgia/stiffness, and mouth burning sensation. Her febrile episodes led to hospitalizations three times. The disease flares were initially concerned for FMF in the absence of MEFV mutations and decreased with colchicine 0.6mg twice daily, which was eventually discontinued because of lack of efficacy and side effects. Complete blood counts, complete metabolic panel, and urinalysis were essentially normal except occasional leukocytosis, thrombocytosis and eosinophilia. C-reactive protein was 12.7(<5 pg/ml), and erythrocyte sedimentation rate and serum amyloid A were normal. Extensive serologic markers were negative for systemic autoimmune diseases. Testing for food allergy, serum IgE and tryptase level was normal. Other immunoglobulins were normal except IgA 31(81-463 mg/dl). A thorough workup for infectious etiology was negative.Gastrointestinal workup, including DQ2/DQ8 testing for celiac disease, computed tomography and endoscopy, was essentially normal without inflammatory bowel disease. Cytokine levels in plasma were normal for IL-1, IL-6, TNFα and IL-5. She also had fibromyalgia and rhinitis/ asthma. Maternal and paternal families both originated from Czechoslovakia, and there was no known history of periodic fever syndromes. She was allergic to a sulfa antibiotic. Physical examination was positive for multiple tender spots. Next generation sequencing for periodic fever syndrome 6-gene panel (MEFV, NLRP3, TNFRSF1A, MVK, NLRP12 and NOD2) was only positive for heterozygous NOD2 IVS8^+158^ and R702W. In this well-studied case, the clinical phenotype and genotype *NOD2* IVS8^+158^/R702W are consistent with YAOS. The diagnostic criteria for YAOS include the characteristic phenotype, specific NOD2 variants, and rigorous exclusion of overlapping disease entities. One of the major differential diagnoses is FMF since YAOS and FMF share some clinical phenotypes; however, they are distinct in clinical manifestations otherwise and genotypes (**Table1**).

**Conclusion:** Given the shared phenotypes and that the current diagnostic criteria for FMF remain clinical, molecular genetic testing to cover both MEFV and NOD2 may be imperative for distinguishing these two diseases. It may be particularly true for those patients who present with atypical phenotype for FMF and/or poor response to colchicine. To prove this hypothesis, further study is warranted to unveil cases of YAOS in the disease population with unspecified autoinflammatory features.

**Consent for publication has been obtained from patient**

Yes

**Disclosure of Interest**

None Declared

#### P2053 May some of the MEFV gene variants cause PFAPA syndrome like symptoms?

##### Amra Adrovic, Mehmet Yildiz, Ipek Ulkersoy, Neslihan Gucuyener, Oya Koker, Sezgin Sahin, Kenan Barut, Ozgur Kasapcopur

###### Pediatric Rheumatology, Istanbul University, Cerrahpasa Medical School, Istanbul, Turkey

####### **Correspondence:** Mehmet Yildiz

**Introduction:** PFAPA syndrome is characterized by periodic fever, aphthous stomatitis, pharyngitis and cervical adenitis. As there is not any gold standard diagnostic test and diagnosis usually depends on clinical diagnostic criteria, sometimes it can be difficult to distinguish this clinical entity from the other periodic fever syndromes. Especially in regions endemic for FMF, it could be challenging to identify PFAPA patients due to many common disease characteristics.

**Objectives:** The objective of the study is to evaluate the PFAPA patients’ *MEFV* gene variation frequencies (if it was performed) and relations between detected variants and clinical manifestations in a large cohort of pediatric PFAPA patients.

**Methods:** Nine hundred and thirty-seven patients that were recorded to our database as PFAPA syndrome were evaluated. Firstly, demographic features and *MEFV* gene variations (if it was performed) were recorded from the patients’ database. Then, patients were reached by phone and asked about characteristics of their fever episodes, presence of acute phase reactant elevation, pharyngitis/cryptic tonsillitis, aphthous stomatitis, cervical lymphadenopathy, arthralgia, arthritis, abdominal pain, headache, nausea or vomiting, chest pain, diarrhea, skin changes, myalgia and conjunctivitis in the course of fever attack, if they had tonsillectomy, ifthey were attack-free after tonsillectomy and if they had clinical response to steroid or colchicine.

**Results:** There were 937 PFAPA patients in our database. *MEFV* gene analysis was performed in 407 (%43) of PFAPA patients and 305 of them had at least one mutation. Most common *MEFV* mutations of patients were: R202Q heterozygotes, M694V heterozygotes, E148Q heterozygotes and P369S heterozygotes, respectively. 45.8% of detected mutations were located in exon 2, 40,3% of them were located in exon 10 and 13,9% of them were located in exon 3 of the *MEFV* gene. Patients were divided into five groups according to their mutations: those with exon 2 mutations, with exon 3 mutation, with exon 10 mutation, those that didn’t have mutation and patients whose *MEFV* mutation analysis hasn’t been performed. Groups were compared according to clinical features. There were significant differences between groups according to presence of pharyngitis/cryptic tonsillitis, arthralgia, abdominal pain, myalgia and tonsillectomy history (**Table 1**).

**Conclusion:** In this study, we reported increased frequency of *MEFV* mutations in a large PFAPA patients cohort.Frequency differences of clinical features between groups suggest that some of the *MEFV* gene mutations may modify phenotype ofPFAPA syndrome. Furthermore, underlying *MEFV* gene mutations possibly lead toPFAPA like clinical presentation in FMF patients. Another remarkable finding of this study is the relatively high P369S mutation rates in patients with PFAPA syndrome. Further studies are needed to investigate the relationship between this mutation and PFAPA disease.

**Disclosure of Interest**

None Declared

**Table 1 (abstract P2053).** Comparison of patients according to their mutations’ location and clinical features

	Exon 2	Exon 3	Exon 10	No Mutation	MEFV study not performed	p
Pharyngitis						
*Yes*	21(87,5)	9(40,9)	45(80,4)	28 (87,5)	168(87,5)	**<0,05**
*No*	3(12,5)	13(59,1)	11(19,6)	4(12,5)	24(12,5)	
Aphthous stomatitis/Cryptic tonsillitis						
*Yes*	15(62,5)	8(36,4)	21(37,5)	17 (53,1)	102(53,1)	0,11
*No*	9(37,5)	14(63,6)	35(62,5)	15(46,9)	90(46,9)	
Cervical lymphadenopathy						
*Yes*	10(41,7)	8(36,4)	21(37,5)	14(56,3)	77(40,1)	0,97
*No*	14(58,3)	14(63,6)	35(62,5)	18(43,7)	115(59,9)	
Arthralgia						
*Yes*	5(20,8)	11(50)	22(39,3)	19(59,4)	66(34,5)	**0,02**
*No*	19(79,2)	11(50)	34(60,7)	13(40,6)	126(65,5)	
Arthritis						
*Yes*	1(4,2)	3(13,6)	1(1,8)	2(6,3)	11(5,7)	0,35
*No*	23(95,8)	19(86,4)	55(98,2)	30(93,7)	181(94,3)	
Abdominal Pain						
*Yes*	17(70,8)	12(54,5)	27(48,2)	16(50)	56(70,8)	**<0,05**
*No*	7(29,2)	10(45,5)	29(51,8)	16(50)	136(29,2)	
Myalgia						
*Yes*	0(0)	8(36,4)	9(16,1)	7(21,9)	24(12,5)	**0,006**
*No*	24(100)	14(63,6)	47(83,9)	25(78,1)	168(87,5)	
Tonsillectomy						
*Yes*	14(58,3)	5(22,7)	21(38,9)	20(62,5)	109(57,1)	**0,005**
*No*	10(41,7)	17(77,3)	33(66,1)	12(37,5)	82(42,9)	
Steroid Response						
*Yes*	17(94,4)	8(88,9)	35(89,7)	20(90,9)	139(89,7)	0,97
*No*	1(5,6)	1(11,1)	4(10,3)	2(9,1)	16(10,3)	

### Non-monogenic SAIDs (basic science)

#### P2054 Next generation sequencing analysis of familial haemophagocytic lymphohistiocytosis related genes in macrophage activation syndrome and secondary HLH

##### Chiara Passarelli^1^, Manuela Pardeo^2^, Ivan Caiello^2^, Elisa Pisaneschi^1^, Antonella Insalaco^2^, Francesca Minoia^3^, Andrea Taddio^4^, Francesco Licciardi^5^, Antonio Novelli^1^, Fabrizio De Benedetti^2^, Claudia Bracaglia^2^

###### ^1^Unit of Laboratory of Medical Genetics; ^2^Division of Rheumatology, IRCCS Ospedale Pediatrico Bambino Gesù, Rome; ^3^Reumatologia Pediatrica, IRCCS Istituto Giannina Gaslini, Genoa; ^4^Institute for Maternal and Child Health, IRCCS “Burlo Garofolo”, University of Trieste, Trieste; ^5^SCDU Pediatria II, Immunoreumatologia, Ospedale Pediatrico Regina Margherita, Turin, Italy

####### **Correspondence:** Claudia Bracaglia

**Introduction:** Macrophage activation syndrome (MAS), a severe complication of paediatric rheumatic disease, is currently classified among the secondary forms of HLH (sHLH). Primary HLH (pHLH) are caused by mutation of genes coding for proteins involved in cytotoxic functions. Mice carrying heterozygous mutations in more than 1 pHLH gene carry a higher risk to develop HLH following viral infection, suggesting that accumulation of partial genetic defects may be relevant in HLH.

**Objectives:** To analyse, with next generation sequencing (NGS), genes involved in pHLH in MAS in the context of different rheumatic diseases and in sHLH.

**Methods:** A targeted resequencing was performed on all patients using a panel including the 7 principal HLH-related genes (*PRF1*, *UNC13d*, *STX11*, *STXBP2*, *Rab27a*, *XIAP*, *SH2D1A*) on MiSeq® and NextSeq550® platforms (Illumina, San Diego, CA). All variants identified were confirmed by Sanger. We took into account variants with an allelic frequency in the global population up to 5% in the dbSNP and Ensembl databases.

**Results:** We analysed 125 patients: 47 MAS, (39 developed this complication in the context of systemic Juvenile Idiopathic Arthritis (sJIA), and 8 in the context of different rheumatic diseases), 32 sHLH, 22 sJIA (without history of MAS) and 24 with different autoinflammatory diseases (AID). sJIA and AID patients were used as control groups. We identified at least 1 heterozygous variant in one of the pHLH genes in 41 patients with MAS or sHLH, with a detection rate of 52%, (45% of MAS and 62% of sHLH patients). More than one variant was identified in 37% patients from both groups, with 19% of both MAS and sHLH patients carrying polygenic variants. The frequency of variants was lower in AID patients compared to the other groups of patients but the difference was significant only comparing AID patients with sHLH who carry one variant (*p*=0.029). The frequency of variants between the 39 sJIA patients with history of at least one episode of MAS and the 22 sJIA patients without history of MAS was similar (41% versus 54%). The distribution of variants among the genes in the group of patients was comparable. *PRF1* and *UNC13d* were the most involved genes in both MAS and sHLH patients, and A91V and R928C were the most common variants identified, respectively. The A91V variant in *PRF1* gene was identified in 19% of both MAS and sHLH patients, while this variant was present, respectively, in only 5% of sJIA and 4% of AID patients. The R928C variant in *UNC13d* gene was identified in 32% of MAS, 18% of sHLH, in 9% of sJIA and 17% of AID patients. Considering the patients clinical characteristics, relapse, CNS involvement, ICU admission and death, we observed that three of the 6 sHLH patients (50%) carrying multiple variants had recurrent episodes of HLH and 2 of them (33%) presented a severe disease with exitus.

**Conclusion:** Monoallelic variants in pHLH-related genes are more frequent in MAS, sHLH and sJIA and less in AID patients, suggesting different molecular mechanisms involved in the diseases. Re-occurrence and severity of disease seem to be more frequent and more severe in patients who carry mutations in two genes in sHLH group. These data may support a polygenic model of sHLH.

**Disclosure of Interest**

C. Passarelli: None Declared, M. Pardeo: None Declared, I. Caiello: None Declared, E. Pisaneschi: None Declared, A. Insalaco: None Declared, F. Minoia: None Declared, A. Taddio: None Declared, F. Licciardi: None Declared, A. Novelli: None Declared, F. De Benedetti Grant / Research Support from: Novartis, Novimmune, Hoffmann- La Roche, SOBI, AbbVie, Pfizer, C. Bracaglia: None Declared

#### P2055 Evaluation of the new classification criteria for PFAPA syndrome

##### Fabio Crimi^1^, Véronique Hentgen^2^, Glory Dingulu^2^, Isabelle Koné-Paut^3^, Sophie Georgin-Lavialle^4^, Pascal Pillet^5^, Michaël Hofer^1^

###### ^1^CHUV, Lausanne, Switzerland; ^2^CH de Versailles, Le Chesnay; ^3^CHU de Bicêtre, Le Kremlin Bicêtre; ^4^Hôpital Tenon, Paris; ^5^CHU de Bordeaux, Bordeaux, France

####### **Correspondence:** Michaël Hofer

**Introduction:** Modified Marshall criteria used for PFAPA syndrome (Periodic Fever, Adenitis, Pharyngitis, Aphtous stomatitis) have never been validated and are little used by the experts because the symptoms of monogenic fevers often overlap with PFAPA ones. A previous study realized a new set of classification criteria based on an international survey and a consensus conference in Genoa in 2018. The aim of our study is to evaluate the performance of the new criteria in a real-life setting.

**Objectives:** Evaluate and test the performance of the Genoa 2018 classification criteria for PFAPA syndrome

**Methods:** This is a multicentric, prospective and descriptive cohort study, through the recurrent fever module of the JIRcohorte platform. 417 patients diagnosed with PFAPA (187), monogenic fever syndromes (167) or unclassified recurrent fever syndrome (UPF=63) from Swiss and French centers were enrolled in the study. The new classification criteria were applied to this cohort and we calculated their performance. We then analyzed which of the criteria performed the less well.

**Results:** 114/187 (61%) PFAPA patients met the new criteria, as well as 20/230 non-PFAPA patients (FMF: 3, MKD: 4, UPF: 13); 73 PFAPA patients did not meet the criteria. We calculated a specificity of 91.3% and a sensitivity of 60.9%. The least satisfied criterion among PFAPA patients not meeting the criteria was “absence of skin rash”. By removing this criterion, the sensitivity improved (81.2%), but the specificity decreased slightly to 86%.

**Conclusion:** Genoa 2017 classification criteria for PFAPA syndrome showed a good specificity but an insufficient sensitivity. Excluding the less satisfied criterion, the set reaches a sensitivity and specificity around 80% which could be a fair compromise for PFAPA classification criteria. Our study highlights the difficulty in establishing classification criteria due to the lack of gold standard for PFAPA diagnosis.

**Disclosure of Interest**

None Declared

#### P2056 Human (pro)caspase-1 variants influence cell death and mechanical properties of human monocytes/macrophages

##### Felix Schulze^1^, Franz Kapplusch^2^, Sabrina Rabe-Matschewsky^1^, Susanne Russ^1^, Maik Herbig^3^, Ursula Range^4^, Angela Rösen-Wolff^1^, Stefan Winkler^1^, Christian M. Hedrich^2,5^, Jochen Guck^3^, Sigrun R. Hofmann^1^

###### ^1^Pediatrics, Universitätsklinikum C.G. Carus, TU Dresden, Dresden, Germany; ^2^Department of Women's & Children's Health, Institute of Translational Medicine, University of Liverpool, Liverpool, United Kingdom; ^3^Biotechnology Center for Molecular and Cellular Bioengineering, TU Dresden; ^4^Institute for Medical Informatics and Biometry, Medizinische Fakultät C.G. Carus, TU Dresden, Dresden, Germany; ^5^Paediatric Rheumatology, Alder Hey Children's NHS Foundation Trust Hospital, Liverpool, United Kingdom

####### **Correspondence:** Sigrun R. Hofmann

**Introduction:** Procaspase-1 (*CASP1*) variants trigger febrile episodes and systemic sterile inflammation in a subset of patients with autoinflammatory diseases despite altered enzymatic activity of procaspase-1 and reduced IL-1β release. These on the first view contradictory observations of reduced IL-1β expression and systemic inflammation could at least partially be explained by increased NF-κB activation through prolonged interaction of *CASP1* variants with receptor interacting protein kinase 2 (RIP2). However, little is known about the exact molecular basics how *CASP1* variants initialize other pro-inflammatory signaling pathways.

**Objectives:** Therefore, we aimed to characterize how *CASP1* variants influence cell death mechanisms, cell proliferation, phagocytosis of macrophages and mechanical properties of monocytes/macrophages.

**Methods:** Establishing an *in vitro* model of a virally transduced human monocytic cell line (THP-1) with shRNA knock-down of endogenous procaspase-1, expressing either wild type or enzymatically inactive procaspase-1 fusion-reporter proteins we analyzed subcellular distribution, inflammasome complex formation, the induction of pyroptosis after NLRP3-inflammasome stimulation using fluorescence and confocal microscopy, life-time imaging and Western blot. Proliferation and phagocytosis was assessed by using the IncuCyte® live-cell analysis system in combination with pH sensitive probes (e.g. pHrodo E. coli microparticle). Real time deformability cytometry (RT-DC) was assessed to define cell mechanical properties of monocytes/macrophages, which could be altered by *CASP1* variants and then contribute to disease pathogenesis (by influencing cell migration).

**Results:** Macrophages with *CASP1* variants strongly influenced formation of cytosolic macromolecular complexes (pyroptosomes/specks). The p.C285A and p.R240Q variants show increased pyroptosome size, which could contribute to the amplification of inflammatory signals if specks are released to the extracellular space. The p.L265S variant additionally showed disturbed nuclear localization. Proliferation and phagocytosis of monocytes/macrophages was not significantly influenced by *CASP1* variants, but RT-DC revealed monocytic shape changes and deformation by *CASP1* variants compared to procaspase-1 wildtype.

**Conclusion:** These findings reveal abnormal pyroptosome formation, impaired nuclear localization and changes in monocyte cell shape and deformation as further mechanisms, how *CASP1* variants could trigger increased inflammation despite reduced IL-1β release. Further investigations are needed to confirm these data in vivo.

**Disclosure of Interest**

None Declared

#### P2057 Patient with NOD2, NLRP1 and C9 variants with recurrent fever attacks, infectious susceptibility and osteomyelitic lesions indicative of NAID?

##### Christian Høst^1^, Mette Christiansen^2^, Sofie E. Jørgensen^3^, Troels Herlin^1^, Mia Glerup^1^, Birgitte Mahler^1^, Trine Mogensen^4^

###### ^1^Pediatrics; ^2^Clinical Immunology, Aarhus University Hospital, Aarhus N; ^3^Biomedicine, Aarhus University, Aarhus C; ^4^Infectious diseases, Aarhus University Hospital, Aarhus N, Denmark

####### **Correspondence:** Christian Høst

**Introduction:** Variants in the Nucleotide-binding Oligomerization Domain-containing protein 2 (NOD2) have been associated with a variety of immunological diseases, such as Crohns disease, sarcoidosis and the rare monogenic autoinflammatory disease Blau syndrome(BS). Recently, NOD2 associated inflammatory disease (NAID) was described as a disease sharing clinical resemblance to BS. While BS seems to arise from gain of function mutations in the NOD2 gene, little is known of the NOD2 variants commonly associated with NAID.

**Objectives:** To describe the genetic, immunological and therapeutic aspects of recurrent fever episodes in a patient with variants in the NOD2, NLRP1, and C9 genes.

**Methods:** Oct 2016 a 12-year-old girl presented with recurrent fever, bone pain, arthralgia, and elevated ESR and CRP. MRI revealed osteomyelitic lesions in both tibiae showing unspecific inflammatory changes on biopsy. Cultures were negative. Whole exome sequencing (WES) was performed on DNA extracted from whole blood. Peripheral blood mononuclear cells (PBMCs) from the patient and healthy controls were stimulated with NF-kB inducers (TNFα and LPS), a NOD2 ligand (MDP), or left untreated. Phosphorylated IkBα, as a measure of NF-kB activation, was measured by Luminex technology. Functional complement analysis was performed to ascertain the role of the C9 variant.

**Results:** WES identified three heterozygous variants in the *NOD2* gene (c.140C>T, p.S47L, CADD score (CS) 21.5, gnomAD frequency (GF) 0.0002;c.3019dupC, p.L.1007fs*2, CS35.0, GF 0.015; and c.2798+158C>T (IVS8^+158^), CS 0, GF 0.10), a heterozygous variant in the *NLRP1* gene (c.923G>A, p.R308Q, CS 0.01, GF 0.0013), and a rare heterozygousvariant in the *C9* gene (c.214C>T, p.Q72*, CS 36.0; GF 0.000007).

Patient PBMCs demonstrated normal NF-kB activation. Upon stimulation, phosphorylated IkBα increased, but comparably to control levels, and was unaffected by MDP. Cytokine analyses are pending.Activation of lectin pathway was 0%, and plasma MBL <100μg/L.

Initially, CNO was suspected and bisphosphonate treatment was initiated. Recurrent fever episodes of 4-8 weeks duration questioned the diagnosis. Nevertheless, adalimumab was initiated 40 mg e.o. week, but due to poor response, substituted with anakinra 2 mg/kg/day after half a year. On this treatment she suffered a new and long attack of daily fever for 8 consecutive weeks with abdominal pain and markedly raised ESR and CRP levels. PET-CT and new MRI remained unremarkable. During fever episodes she continued to develop minor skin and mucosal infections with herpes simplex virus and staphylococci, but despite relevant antibiotic treatment fever episodes continued. Renewed literature review on NOD2 variants raised the suspicion of the recently described NAID, also known as Yao Syndrome. It is characterized by recurrent fever episodes, weight loss, fatigue, polyarthralgia/polyarthritis, and often increased acute phase laboratory findings. Genetically, one or more variants in the NOD2 gene are typically found.

**Conclusion:** We believe our patient has NAID due to the presence of two NOD2 variants IVS8^+158^ and 1007fs commonly associated with this condition. We speculate that a defective NOD2 dependent phagocytosis leads to bacterial persistence and secondary inflammatory changes. Additionally, the variants in NLRP1 and C9 might contribute to her overall hyperinflammation and infectious susceptibility. Pending cytokine analyses might further clarify whether these immune responses act through inflammasome-dependent or -independent cellular pathways.

**Consent for publication has been obtained from patient**

Yes

**Disclosure of Interest**

None Declared

#### P2058 The cytokines profiles in chronic non-bacterial osteomyeilitis non-systemic juvenile idiopathic arthritis and healthy controls

##### Mikhail Kostik^1^, Daria Kozlova^2,3^, Dmitry Vasiliev^2,3^, Olga Karlina^2,4^, Maria Makhova^1^, Lubov Sorokina^1^, Vyacheslav Zorin^5^, Eugenia Isupova^1^, Shamai Magomedova^6,7^, Inna Kostik^8^, Alexander Mushkin^5^

###### ^1^Saint-Petersburg State Pediatric Medical University; ^2^LLC “Scientific and Production Company “Abris+”; ^3^Sechenov Institute of Evolutionary Physiology and Biochemistry RAS; ^4^Saint-Petersburg State Institute of Technology; ^5^Science research Institute of Phthisiopulmonology, Saint-Petersburg; ^6^Republican Children’s Clinical Hospital; ^7^Dagestan State Medical Academy, Makhachkala; ^8^Children's Rehabilitation Center “Detskye Duny”, Saint-Petersburg, Russian Federation

####### **Correspondence:** Mikhail Kostik

**Introduction:** Chronic non-bacterial osteomyelitis (CNO) is an immune-mediated disease associated with cytokine dysbalance.

**Objectives:** The aim of our study was to evaluate the cytokines levels in CNO and compare to other immune-mediated disease and healthy controls.

**Methods:** The diagnosis of CNO made with criteria, proposed by Jansson (2007, 2009), after the exclusion of other causes of bone disease. We included 35 patients with NBO, 18 patients with non-systemic juvenile idiopathic arthritis (JIA) and five healthy controls (HC). We evaluated plasma levels of 14-3-3 protein, calprotectin, and interleukine-6 in 3 groups by the ELISA. Statistical analysis was carried out with Statistica 10.0 software. We utilized descriptive statistics (Me; IQR), Mann-Whitney and Wilcoxon tests, Pierson's and Spearmen’s correlation analysis. We used the Bonferroni’s correction, so significant level was <0.017 for group comparison.

**Results:** We have found differences in the proinflammatory biomarkers between CNO, JIA, and HC. Patients with NBO had higher levels of 14-3-3- protein, calprotectin and interleukin-6 compare to HC, but lower levels of 14-3-3 protein and interleukine-6 then in JIA patients (table 1).

We have found positive correlation between 14-3-3- protein and ESR (r=0.41, p=0.04 for whole group and r=0.52’ p=0.047 for NBO), and negative correlation with multifocal involvement (r=-0.45, p=0.016) and hemoglobin (r=-.072, p=0.028). There were not found differences in the studied biomarkers in CNO patients depend on age, onset age, type of bone inflammation (granulomatosis vs non-granulomatosis), fever, spine involvement and type of treatment.

**Conclusion:** Patients with CNO had proinflammatory activity but less then JIA patients, further investigations required for finding new more precise biomarkers.

This work supported by the Russian Foundation for Basic Research (grant № 18-515-57001)

**Disclosure of Interest**

None Declared

**Table 1 (abstract P2058).** Comparison of the cytokine levels between CNO, JIA, and HC

Parameter	CNO (n=35)	JIA (n=18)	HC (n=5)	р	р_1_	р_2_	р_3_
ESR, mm/h	25.0 (8.0-43.0)	9.0 (3.0-14.0)	5.0 (2.0-5.0)	0.007	0.018	0.008	0.174
CRP, mg/l	10.1 (6.0-40.6)	1.75 (0.7-5.6)	0.5 (0.2-0.5)	0.0001	0.007	0.0001	0.024
14-3-3 protein, ng/ml	19.9 (18.3-27.1)	51.9 (39.7-60.0)	2.4 (1.5-5.1)	0.00001	0.0000001	0.000003	0.00006
Calprotectin, ng/ml	5.9 (5.2-6.7)	3.95 (3.1-14.3)	0.75 (0.68-0.84)	0.004	0.122	0.000003	0.00006
Interleukin-6, ng/ml	45.5 (40.0-51.0)	445.1 (69.3-530.5)	4.0 (3.2-4.9)	0.00001	0.0000001	0.000003	0.00006

p – all groups, р_1_ CNO vs JIA; р_2_NBO and HC; р_3_ JIA vs HC

#### P2059 Genetic analysis of the patients with early-onset chronic non-bacterial osteomyelitis from north Caucasian regions of Russia

##### Mikhail Kostik^1^, Maria Makhova^1^, Evgeny Suspitsin^1,2^, Anna Sokolenko^1,2^, Vyacheslav Zorin^3^, Eugenia Isupova^1^, Shamai Magomedova^4,5^, Inna Kostik^6^, Hiroshi Takayanagi^7^, Alexander Mushkin^3^, Evgeny Imyanitov^1,2^

###### ^1^Saint-Petersburg State Pediatric Medical University; ^2^N.N. Petrov Institute of Oncology; ^3^Science research Institute of Phthisiopulmonology, Saint-Petersburg; ^4^Republican Children's Clinical Hospital; ^5^Dagestan State Medical Academy, Makhachkala; ^6^Children’s Rehabilitation Center “Detskye Duny”, Saint-Petersburg, Russian Federation; ^7^Tokyo University, Tokyo, Japan

####### **Correspondence:** Mikhail Kostik

**Introduction:** Chronic non-bacterial osteomyelitis (CNO) is a heterogenic group of immune-mediated inflammatory bone diseases with unclear pathogenesis. At present, only a few genes associated with this condition have identified.

**Objectives:** The aim of the study was to evaluate the spectrum of mutations in genes associated with primary immunodeficiency syndromes (PIDs) and autoinflammatory diseases (AIDs) in the cohort of patients with early-onset CNO from North Caucasus (Dagestan and Chechnya).

**Methods:** We selected a subgroup of the CNO patients (n=22) having the following features: 1) early disease onset (<5 years); 2) all children were initially diagnosed as having tuberculosis (TB) due to bone morphology findings (granulomatosis, e.g. tuberculosis-like inflammation), but had negative TB culture test; 3) initial treatment with a combination of 3-4 anti-MBT drugs during 1-2 years was ineffective, and the patient continued to develop new inflammatory bone foci; 4) patients had very severe clinical and radiological signs of disease; 5) all patients were from areas with traditionally high prevalence of consanguinity. Targeted next-generation sequencing (NGS) analysis of 302 genes related to PIDs and AIDs was performed.

**Results:** Rare variants of PID genes were detected in 7/22 (32%) patients. Mutations affecting the genes previously associated with CNO were found only in two patients: one of them carried heterozygous variant IL1RN c.170G>T (p.C57F), and another had IL1RN c.512T>C (p.V171A). No mutation of LPIN2 was revealed. Other detected variants included one pathogenic MEFV p.M694V mutation in the heterozygous state and some VUS in CD40LG, NLRP12, CR2, NLRP3, IL12B, PLCG2, SH3BP2, CARD14, IRF8, CASP10, and NFKB1A genes.

**Conclusion:** Mutations in known genes were detected only in a minor fraction of CNO patients from Dagestan and Chechnya.

This work supported by the Russian Foundation for Basic Research (grant № 18-515-57001) and by Japan Medical Research Foundation (grant № 18jmrf001).

**Disclosure of Interest**

None Declared

#### P2060 Proteomic profile of peripheral blood mononuclear cells in Behçet’s disease

##### Asli Kirectepe Aydin^1^, Yesim Ozguler^2^, Didar Ucar^3^, Emire Seyahi^2^, Hasan Yazici^2^, Eda Tahir Turanli^1,4^

###### ^1^Molecular Biology-Genetics & Biotechnology Department, Dr Orhan Öcalgiray Molecular Biology-Biotechnology & Genetics Research Centre, Graduate School of Science, Engineering & Technology, Istanbul Technical University; ^2^Division of Rheumatology, Department of Internal Medicine, Cerrahpasa Medical School; ^3^Division of Ophthalmology, Department of Surgical Medicine, Cerrahpasa Medical School, Istanbul University – Cerrahpasa; ^4^Department of Molecular Biology & Genetics, Faculty of Science Literature, Istanbul Technical University, Istanbul, Turkey

####### **Correspondence:** Eda Tahir Turanli

**Introduction:** Behçet’s disease (BD) is a systemic inflammatory disorder with unknown etiology. Although HLA-B51 has been previously described as the most associated allel with BD, presence of BD patients without the B51 allel (42.8%), healthy individuals with HLA-B51 allel (18.1%) were reported. Also, various genetic associations with BD revealed through several genome-wide association and linkage analysis indicate that genetic and environmental factors may play role in BD pathogenesis.

**Objectives:** Investigation of proteome profile is crucial to elucidate disease pathogenesis and related molecular pathways especially in phenotypes like BD with complex genetics and clinical heterogeneity. In this study, we aimed to identify differentially expressed proteins in BD and related pathways through proteomic analyses performed on peripheral blood mononuclear cell (PBMC) samples.

**Methods:** Study groups were composed of active BD (N=33), inactive BD (N=26), and healthy controls (N=28). Isolated PBMC protein samples from each group were pooled and then separated using 2D-DIGE. Protein spots with at least 2 fold-changes or statistical significance in terms of spot density were compared among the groups, and identified by MALDI-TOF-MS. Bioinformatic pathway analyses were carried out through KEGG, PANTHER, and STRING databases. Western blot analyses were performed for WDR1, PGK1, and calreticulin to confirm 2D-DIGE results.

**Results:** A total of 369 protein spots were detected by 2D-DIGE. A number of differentially expressed spots were found: 115 for active vs inactive BD, 118 for active BD vs healthy controls, 129 for inactive BD vs healthy controls. Among them, the strongest 45 spots were further analyzed through MALDI-TOF-MS. Following differentially expressed proteins were determined as the final candidates: calreticulin, WD repeat-containing protein-1 (WDR1), Fructose-bisphosphate aldolase-C, ficolin-1, fibrinogen alpha chain, fibrinogen beta chain, filamin-A, FUSE-binding protein-1, phosphoglycerate kinase-1 (PGK1), stathmin, vinculin, hnRNP-M, HSPA8, myosin light polypeptide-6, talin-1, and tropomyosin alpha-3 chain. Western blot analyses of WDR1 and PGK1 confirmed that WDR1 is decreased (1.3 fold), whereas PGK1 is increased (1.5 fold) in BD patients, although the differences were not statistically significant. However, although decreased calreticulin level was observed in BD patients compared to healthy controls by 2D-DIGE analysis, we detected increased calreticulin level in BD patients by western blot analysis.

**Conclusion:** We identified proteins related to glycolysis, coagulation, ER stress, and cytoskeleton by 2D-DIGE analysis. Although none of these proteins have been previously implicated in BD, lower levels of stathmin, WDR1, and calreticulin having roles in inflammation was revealed in BD patients. Also increased PGK1 level was observed in BD patients, which is a glycolytic enzyme and previously suggested to have a role in rheumatoid arthritis. The expression of proteins involved in ER protein processing (calreticulin, HSPA8, GRP78-BiP) were down-regulated in BD patients compared to healthy controls, which hints the involvement of ER stress in BD and will be investigated in more detail.

**Disclosure of Interest**

None Declared

#### P2061 IL1RAP as a candidate gene for autosomal dominant Behçet’s disease

##### Gamze Turan^1^, Ilker Karacan^1^, Serdal Ugurlu^2^, Selcuk Dasdemir^3^, Huri Ozdogan^2^, Aslihan Tolun^4^, Eda Tahir Turanli^1^

###### ^1^Department of Molecular Biology and Genetics, Istanbul Technical University; ^2^Department of Rheumatology, Istanbul University Cerrahpasa; ^3^Department of Medical Genetics, Istanbul University; ^4^Department of Molecular Biology and Genetics, Bogazici University , Istanbul, Turkey

####### **Correspondence:** Eda Tahir Turanli

**Introduction:** Behçet’s Disease (BD) is a chronic inflammatory disorder with unknown aetiology. Higher prevalence along the Silk Route and familial cases suggest a genetic contribution to its pathogenesis. We present a Turkish family diagnosed with BD involving the father and three of the four children, all with disease onset before age 16. All affected individuals meet the International Study Group criteria for BD. Oral/ genital ulcers and ostiofolliculitis were shared by all probands. Posterior uveitis and deep vein thrombosis were diagnosed in the father while recurrent arthritis was prominent in the affected children.

**Objectives:** This study aimed to identify the putative gene responsible for autosomal dominant BD.

**Methods:**Exome sequencing was performed for unaffected mother and the four affected family members. Heterozygous, rare (MAF <0.005) and exonic/splicing variants were selected from among the shared variants, and those shared also with mother were excluded. Candidate variants that were chosen after analysis via bioinformatics tools were tested by Sanger sequencing of aforementioned members along with an unaffected and an undiagnosed sibling. Those variants were also screened in 152 healthy controls with high resolution melting (HRM) method. To evaluate the effect of the strongest candidate gene to autoinflammation, levels of IL1β isolated from PBMC cells in four healthy controls and an affected member in exacerbation period were compared by ELISA method.

**Results:** Exome analysis showed that patients did not carry variants in *TNFAIP3* which is the known gene for autosomal dominant BD. Six rare, heterozygous variants which were shared among four affected members but absent in unaffected mother were selected as initial candidates. Three of those variants were eliminated because they were present in a healthy sib. The remaining three prominent candidate variantsc.35A>T (p.D12V) *in NAGK* (rs150821125), c.1490C>G (p.A497G) in *SLC9A2*and c.11T>G (p.L4R) in *IL1RAP* (rs200782803) were absent in control samples. Furthermore, variants in *SLC9A2*and *IL1RAP* were not reported in gnomAD, 1000G, ExAC and ESP6500 databases. They are predicted as deleterious by computational prediction tool such as Mutationtaster, SIFT. Due to its involvement in IL1 signaling, *IL1RAP* stood out as the most prominentcandidate.No significant difference in IL1β levels were observed between the affected member and the healthy controls (165.4 μg/ml vs 165.4 μg/ml respectively).

**Conclusion:** Our results suggest that in this family the most relevant candidate isc.11T>G (p.L4R) in IL1RAP.This is the first report suggesting a possible association of *IL1RAP* with autosomal dominant BD. Further studies are needed to better define *IL1RAP* effect in BD pathogenesis.

**Disclosure of Interest**

None Declared

### Systemic-onset JIA and AOSD

#### P2062 Canakinumab in systemic juvenile idiopathic arthritis: clinical inactive disease rate and safety initalian patients

##### Manuela Pardeo^1^, Claudia Bracaglia^1^, Anna Lucia Piscitelli^1^, Arianna De Matteis^2^, Jessica Tibaldi^3^, Maria Alessio^2^, Achille Marino^4^, Giovanni Conti^5^, Maria Cristina Maggio^6^, Clotilde Alizzi^6^, Francesco Licciardi^7^, Alma Nunzia Olivieri^8^, Giovanni Filocamo^9^, Francesca Orlando^2^, Silvana Martino^7^, Rolando Cimaz^4^, Angelo Ravelli^3^, Fabrizio De Benedetti^1^

###### ^1^Division of Rheumatology, IRCCS Ospedale Pediatrico Bambino Gesù, Rome; ^2^Department of Pediatrics, Rheumatology Unit, University Federico II, Naples; ^3^Department of Paediatric Rheumatology, University of Genova and Giannina Gaslini Institut, Genoa; ^4^Department of Neurosciences, Psychology, Drug Research and Child Health, Rheumatology Unit, Meyer Children's Hospital, University of Florence, Florence; ^5^Unit of Pediatric Nephrology and Rheumatology, University of Messina, Messina; ^6^University Department Pro.Sa.M.I. “G. D'Alessandro”, University of Palermo, Palermo; ^7^Department of Pediatrics and Infectious Diseases, University of Turin, Turin; ^8^Department of Woman, Child, and General and Specialistic Surgery, Second University of Studies of Naples, Naples; ^9^Intermediate Pediatric Care Unit, Fondazione IRCCS Ca’ Granda Ospedale Maggiore Policlinico, Milan, Italy

####### **Correspondence:** Manuela Pardeo

**Introduction:** Systemic juvenile idiopathic arthritis (sJIA) is a polygenic autoinflammatory disease. The pathophysiology is still unclear, it is now well known that innate immune mechanisms play a central role with overproduction of inflammatory cytokines. The increased knowledge on the role of these cytokines has provided a change in the natural history of the disease with the introduction of the targeted treatments. Remarkable results has been observed with canakinumab, an anti-interleukin-1β monoclonal antibody, in two clinical trials but little information are available in real life.

**Objectives:** To evaluate clinical inactive disease rate and safety of canakinumab in Italian patients with sJIA.

**Methods:** We have collected retrospectively clinical and laboratory data of patients with sJIA treated with canakinumab in 9 Italian Paediatric Rheumatology centers. Clinically inactive disease (CID) at 6 months was defined according to Wallace criteria.We analyzed the effect of canakinumab on fever, rash, number of actives joints, erythrocyte sedimentation rate (ESR), C-reactive protein (CRP) and physician’s global assessment of disease activity score. Clinical and laboratory data were obtained using a standard data collection form.

**Results:** Forty seven patients (26 F) were included in the analyses**.** The median age (range) at the diagnosis and at the beginning of treatment with canakinumab was 7.6 (1-14.7) and 10.2 (1.7-22.2) years, respectively. Twenty seven patients (57.4%) had been previously treated with other biologic agents (18 with anakinra, 1 with tocilizumab, 6 with both and 2 with etanercept), withdrawn for inefficacy in 15/27 (55.5%). Thirty patients (63.8%) were receiving concomitant treatment with glucocorticoids at the median dose (range) of 0.69 (0.02-2.75) mg/kg/die. Thirty nine out of 47 patients had > 6 months of follow-up. Among these 39 patients, 27 (69.2%) achieved CID at 6 months and 5/27 (18.5%) were still on glucocorticoids. Of the 30 patients who received concomitant glucocorticoids at baseline, 24 achieved 6 months of follow-up and 12 (50%) of these were able to withdraw glucocorticoids. Minor adverse events were reported in 5/30 (16.6%) patients:upper respiratory tract infections in 4 and transient injection site reaction in 1. No cases of macrophage activation syndrome was reported.

**Conclusion:** Our results provide initial real world evidence of the efficacy of treatment with canakinumab in patients with sJIA. In our study the percentage of patients who reached CID at 6 months is slightly higher (69.2%) than reported at the end (from 3 months to one year) of the 2 published randomized trials (60%) (1). No serious adverse events were recorded in our population.

**Reference**

(1) N. Ruperto *et al. Two randomized trials of Canakinumab in systemic juvenile idiopathic arthritis.*N Engl J Med, 367 (2012), pp. 2396-2406.

**Disclosure of Interest**

M. Pardeo: None Declared, C. Bracaglia: None Declared, A. L. Piscitelli: None Declared, A. De Matteis: None Declared, J. Tibaldi: None Declared, M. Alessio: None Declared, A. Marino: None Declared, G. Conti: None Declared, M. C. Maggio: None Declared, C. Alizzi: None Declared, F. Licciardi: None Declared, A. N. Olivieri: None Declared, G. Filocamo: None Declared, F. Orlando: None Declared, S. Martino: None Declared, R. Cimaz: None Declared, A. Ravelli Grant / Research Support from: Angelini, AbbVie, Bristol-Myers Squibb, Johnson & Johnson, Novartis, Pfizer, Reckitt-Benkiser, and Roche,Consultant for: Angelini, AbbVie, Bristol-Myers Squibb, Johnson & Johnson, Novartis, Pfizer, Reckitt-Benkiser, and Roche, F. De Benedetti Grant / Research Support from: Novartis, Novimmune, Hoffmann- La Roche, SOBI, AbbVie, Pfizer

#### P2063 Whole blood phosphorylated STAT1 levels in patients with active macrophage activation syndrome and secondary hemophagocytic lymphohistiocytosis

##### Antonia Pascarella, Claudia Bracaglia, Emiliano Marasco, Ivan Caiello, Gian Marco Moneta, Luciapia Farina, Giulia Marucci, Alessia Arduini, Manuela Pardeo, Fabrizio De Benedetti, Giusi Prencipe

###### Division of Rheumatology, Ospedale Pediatrico Bambino Gesù, Rome, Italy

####### **Correspondence:** Antonia Pascarella

**Introduction:** A large body of evidence demonstrates the pivotal role of interferon gamma (IFNγ) in the pathogenesis of secondary hemophagocytic lymphohistiocytosis (sHLH) and macrophage activation syndrome (MAS). IFNγ is a key endogenous activator of macrophages and exerts its biological activities by phosphorylation of the transcription factor Signal transducer and activator of transcription 1 (STAT1).

**Objectives:** In this study, we aimed to investigate whether the phosphorylation status of STAT1 in whole blood cells represents a good biomarker for the identification of patients at early stages of MAS/ sHLH.

**Methods:** Whole blood samples from patients with suspected untreated MAS/sHLH (n=7) and suspected treated (glucocorticoids) MAS/sHLH (n=9) were collected prospectively. As controls, whole blood samples from patients with active systemic Juvenile Idiopathic Arthritis (sJIA) without MAS at sampling (n=6) and healthy subjects (HS, n=7) were used. Fresh whole blood cells were left unstimulated or stimulated with different concentrations of IFNγ (0.01, 0.1, 1, 10 ng/ml) for 10 minutes. The intracellular phosphorylation levels of Tyrosine (701) STAT1 (pSTAT1) were evaluated by flow cytometry. Results have been expressed as Delta mean fluorescence intensity (MFI), calculated by subtracting the MFI of cells stained with isotype control antibody (Ab) from that stained with anti-pSTAT1 Ab. Anti CD3, CD14 and CD16 staining was performed to discriminate the monocyte, neutrophil, natural killer- and T- cell subpopulations.

**Results:** In both treated and untreated MAS/sHLH patients, flow cytometric analyses showed no significant differences in pSTAT1 levels in unstimulated monocyte, neutrophil, natural killer and T cell subpopulations, compared to sJIA and healthy subjects. Interestingly, we found that, compared to sJIA and healthy subjects, in patients with untreated MAS/sHLH, pSTAT1 levels were significantly higher in monocytes (p<0,01 Vs HS, p<0,05 Vs sJIA and p<0,01 Vs HS, p<0,05 Vs sJIA, for stimulation with 1 and 10 ng/ml of IFNg respectively) and neutrophils (p<0,05 Vs HS, p<0,05 Vs sJIA and p<0,01 Vs HS, p<0,01 Vs sJIA) stimulated with the higher concentrations of IFNγ (1 and 10 ng/ml). In contrast, we did not find differences in the levels of pSTAT1 observed in stimulated monocytes and neutrophils from treated MAS/sHLH patients or those observed in cells from active sJIA and healthy subjects.

**Conclusion:** Our results demonstrate that the combined evaluation of pSTAT1 levels by flow cytometry in monocytes and neutrophils stimulated with high doses of IFNγ show high levels of pSTAT1 and might contribute to the identification of patients at early stages of MAS/sHLH. In addition, our results further support the involvement of IFNγ in the development of the diseases, as suggested by the increased phosphorylated STAT1 levels exclusively in patients with active MAS/sHLH and not in patients with active sJIA.

**Disclosure of Interest**

None Declared

#### P2064 Whole blood cells from patients with systemic juvenile idiopathic arthritis (SJIA) in clinical inactive disease displayed a dysregulated response to TLR-4 stimulation

##### Antonia Pascarella, Manuela Pardeo, Ivan Caiello, Claudia Bracaglia, Marianna N. Rossi, Giulia Marucci, Emanuela Sacco, Fabrizio De Benedetti, Giusi Prencipe

###### Bambino Gesù Children’s Hospital, Rome, Italy

####### **Correspondence:** Giusi Prencipe

**Introduction:** Systemic juvenile idiopathic arthritis (sJIA) is a polygenic autoinflammatory disease. Innate immune mechanisms appear to play a central role in the pathogenesis of the disease. Nevertheless, a better understanding of the pathophysiology of sJIA is still needed to identify patients responsive to IL-1 or IL-6 targeted therapies.

**Objectives:** In this study, we evaluated the production of IL-1β, IL-6 and TNF-α by fresh whole blood cells isolated from sJIA patients in disease remission, after stimulation with the TLR-4 ligand lipopolysaccharide (LPS), and we investigated whether sJIA patients that respond or not-respond to treatment with the IL-1 receptor antagonistanakinra show a different response.

**Methods:** We collected fresh whole blood samples from sJIA patients during clinical inactive disease (inactive sJIA, n=19) and sJIA patients during active disease (active sJIA n=4). Active and inactive disease was defined at time of sampling. As controls, fresh whole blood samples from healthy subjects (HS, n=10) were used. Whole blood cells were left unstimulated or stimulated with 10ug/mL of LPS for 24 hours. Cytokine levels (IL-1β, IL-6 and TNF-α) released in the supernatants were measured by ELISA. Response to anakinra was defined as achievement of clinical inactive disease off glucocorticoids at 6 months after initiation of anakinra treatment.

**Results:** We found that LPS-stimulated cells from inactive sJIA patients released significantly higher amounts of all the inflammatory cytokines tested, compared to HS (p<0.01). In addition, cells from inactive sJIA patients produced significantly higher levels of IL-1β also compared to active sJIA patients (p<0.05). When we divided inactive sJIA patients in two groups (responders and non-responders), depending on the clinical response to anakinra treatment, we observed that in both groups of patients IL-1β, IL-6 and TNF-α levels were significantly higher than those observed in HS. In addition, we found that LPS-stimulated whole blood cells from non-responder inactive sJIA patients released significantly higher levels of IL-1β and TNF-α compared to responder inactive sJIA patients.

**Conclusion:** Our preliminary results show a dysregulated production of inflammatory cytokines by whole blood cells from sJIA patients in remission disease, when stimulated with a TLR-4 agonist.

**Disclosure of Interest**

None Declared

#### P2065 Anakinra drug retention rate and predictive factors of drug survival in systemic juvenile idiopathic arthritis and adult onset Still’s disease

##### Jurgen Sota^1^, Donato Rigante^2^, Antonella Insalaco^3^, Paolo Sfriso^4^, Salvatore de Vita^5^, Rolando Cimaz^6^, Giuseppe Lopalco^7^, Giacomo Emmi^8^, Francesco La Torre^9^, Claudia Fabiani^10^, Alma N. Olivieri^11^, Marco Cattalini^12^, Daniele Cammelli^13^, Romina Gallizzi^14^, Maria Alessio^15^, Raffaele Manna^16^, Ombretta Viapiana^17^, Micol Frassi^18^, Armin Maier^19^, Carlo Salvarani^20^, Rosaria Talarico^21^, Roberta Priori^22^, Maria C. Maggio^23^, Manuela Pardeo^3^, Carla Gaggiano^24^, Salvatore Grosso^24^, Fabrizio de Benedetti^25^, Antonio Vitale^26^, Luca Cantarini^26^

###### ^1^Research Center of Systemic Autoinflammatory Diseases and Behçet's Disease Clinic, Department of Medical Sciences, Surgery and Neurosciences,, University of Siena, Siena; ^2^Institute of Pediatrics , Fondazione Policlinico A. Gemelli IRCCS; ^3^Division of Rheumatology, Department of Pediatric Medicine, Bambino Gesù Children's Hospital IRCCS, Rome; ^4^Rheumatology Unit, Department of Medicine, University of Padua, Padua; ^5^Department of Medical and Biological Sciences, Rheumatology Clinic, Univeristy of Udine, Udine; ^6^Rheumatology Unit, Meyer Children's Hospital, University of Florence, Florence; ^7^Department of Emergency and Organ Transplantation-Rheumatology Unit, University of Bari, Bari; ^8^Department of Experimental and Clinical Medicine, University of Florence, Florence; ^9^Pediatric Rheumatology Section, Pediatric Oncoematology Unit, Vito Fazzi Hospital, Lecce; ^10^Ophthalmology Unit, Department of Medicine, Surgery and Neuroscience, University of Siena, Siena; ^11^Dipartimento della Donna, del Bambino e di Chirurgia Generale e Specialistica, Seconda Università degli Studi of Naples, Naples; ^12^Pediatric Clinic, University of Brescia and Spedali Civili di Brescia, Brescia; ^13^Experimental and Clinical Medicine Department, University of Florence, Florence; ^14^Department of Pediatrics, Azienda G. Martino, University of Messina, Messina; ^15^Department of Pediatrics, University Federico II, Naples; ^16^Periodic Fever Research Center, Università Cattolica Sacro Cuore, Rome; ^17^Rheumatology Section, Department of Medicine, University of Verona, Verona; ^18^Rheumatology and Clinical Immunology Unit, Department of Clinical and Experimental Sciences, University of Brescia and Spedali Civili di Brescia, Brescia; ^19^Struttura Semplice di Reumatologia, Ospedale di Bolzano, Bolzano; ^20^Rheumatology Unit, Department of Internal Medicine, Azienda Ospedaliera ASMN IRCCS,, Reggio Emilia; ^21^Rheumatology Unit, Department of Clinical and Experimental Medicine, University of Pisa, Pisa; ^22^Department of Internal Medicine and Medical Specialties, Rheumatology Unit, Sapienza University of Rome, Rome; ^23^Universitary Department “Pro.S.A.M.I.”, University of Palermo, Palermo; ^24^Clinical Pediatrics, Department of Molecular Medicine and Development, University of Siena, Siena; ^25^Division of Rheumatology, Department of Pediatric Medicine, Bambino Gesù Children's Hospital, IRCCS, Rome; ^26^Research Center of Systemic Autoinflammatory Diseases and Behçet's Disease Clinic, Department of Medical Sciences, Surgery and Neurosciences,University of Siena, Siena, Italy

####### **Correspondence:** Jurgen Sota

**Introduction:** Only a few studies have reported the long-term efficacy of interleukin (IL)-1 inhibition in systemic juvenile idiopathic arthritis (sJIA) and adult onset Still’s disease (AOSD). We herein describe Anakinra (ANA) effectiveness expressed in terms of drug retention rate (DRR) and evaluate predictive factors of drug survival in sJIA and ASOD patients.

**Objectives:** Examine the overall DRR of ANA in sJIA and AOSD patients. Explore the influence of biologic line of treatment, and the concomitant use of disease modifying anti-rheumatic drugs (cDMARDs) on DRR in the whole sample and stratified according to the disease thereafter; find eventual predictive factors associated with events leading to drug discontinuation. The corticosteroid (CS)- and cDMARDS-sparing effect, the impact of treatment delay on survival and the record of safety profile constituted ancillary aims.

**Methods:** Medical records from 61 sJIA and 76 AOSD patients treated with ANA in 24 Italian tertiary referral centers were retrospectively reviewed.

**Results:** The cumulative retention rate of ANA at 12-, 24-, 48- and 60-months of follow-up was 74.3%, 62.9%, 49.4% and 49.4% respectively, without any significant differences between sJIA and AOSD patients (p=0.164), and between patients treated in monotherapy compared to the subgroup co-administered with conventional cDMARDs (p=0.473). On the other hand, a significant difference in DRR was found between biologic-naive patients and those previously treated with biologic drugs (p=0.009), which persisted even after adjusting for pathology (p=0.013). In regression analysis, patients experiencing adverse events (AEs) (HR=3.029 [C.I. 1.750-5.242], p<0.0001) and those previously treated with other biotechnologic agents (HR=1.818 [C.I. 1.007-3.282], p=0.047) were associated with a higher hazard ratio of ANA discontinuation. The median treatment delay was significantly higher among patients discontinuing ANA (p<0.0001). A significant CS- (p=0.033) and cDMARDs-sparing effect (p<0.0001) was also recorded. Less than one third of our cohort developed AEs and 85% were deemed mild in nature, with 70% involving the skin.

**Conclusion:** Our findings display an overall excellent DRR of ANA on the long run for both sJIA and AOSD that may be further optimized by closely monitoring patient’s safety issues and employing this IL-1 inhibitor as a first-line biologic as early as possible. Moreover, ANA allowed a significant drug-sparing effect while showing a good safety profile.

**Disclosure of Interest**

None Declared

#### P2066 Predictors of effectiveness of anakinra in systemic juvenile idiopathic arthritis

##### Jessica Tibaldi^1^, Bendetta Saccomanno^1^, Francesca Minoia^2^, Francesca Bagnasco^3^, Angela Pistorio^3^, Andreessa Guariento^4^, Roberta Caorsi^3^, Alesandro Consolaro^1^, Marco Gattorno^3^, Angelo Ravelli^1^

###### ^1^Istituto G. Gaslini/Università degli Studi di Genova, Genoa; ^2^Fondazione IRCCS Ca’ Granda, Ospedale, Milan; ^3^Istituto G. Gaslini, Genoa, Italy; ^4^Instituto de Criança – FMUSP, Rio de Janeiro, Brazil

####### **Correspondence:** Jessica Tibaldi

**Introduction:**Systemic juvenilei diopathicarthritis (sJIA) is the most severe and a rather distinct subtype of JIA. It is common viewthatsJIAis the most severe form of childhood arthritis and the most difficult to treat. Recently the use of interleukin(IL)-1 antagonists has led to a significant improvement of the disease’s long-term evolution and has confirmed this cytochin’s key-role in the pathogenesis of sJIA. A number of potential predictors of the therapeutic effectiveness of IL-1 inhibitors have been reported, which include less severe joint disease and increased white blood cell count, shorter disease duration, older age at disease onset and use of IL-1 blockade as first-line therapy. However, because the experience gained so far is still limited, there is a need of further data to better characterize the profile of sJIA patients who are more susceptible to respond to IL-1 blockade.

**Objectives:** To seek predictors of therapeutic response to the interleukin (IL)-1 inhibitor anakinra in children with systemic-onset juvenile idiopathic arthritis (sJIA).

**Methods:** The clinical charts of all patients with sJIA who were newly treated with anakinra at our center between 2004 and 2017 were reviewed retrospectively. Predictors included baseline demographic, clinical, and laboratory variables as well as previous or concomitant therapies. The effectiveness of anakinra was assessed at 1 year after treatment start. Complete clinical response (CCR) was defined as absence of fever, physician’s global assessment ≤1, count of active joints ≤1, negative C-reactive protein, and ≥75% reduction of corticosteroid dose. According to the intention-to-treat principle, patients who had anakinra discontinued before 1 year for any reasons other than disease remission were classified as nonresponders. Statistics included univariate and multivariable analyses.

**Results:** Of the 62 patients included in the study, 24 (39%) met the criteria for CCR at 1 year, whereas 38 (61%) did not. On multivariable analysis, independent correlations with achievement of CCR were identified for shorter disease duration, lower active joint count, higher ferritin level, and greater activity of systemic manifestations. The area under the curve of the model was 0.83.

**Conclusion:** Our findings help to delineate the clinical profile of patients with sJIA who are more likely to benefit from IL-1 blockade. They also underscore the need for studies aimed at examining the therapeutic role of early IL-1 inhibition and to identify biomarkers predicting response to either IL-1 or IL-6 antagonists.

**Disclosure of Interest**

J. Tibaldi: None Declared, B. Saccomanno: None Declared, F. Minoia: None Declared, F. Bagnasco: None Declared, A. Pistorio: None Declared, A. Guariento: None Declared, R. Caorsi: None Declared, A. Consolaro: None Declared, M. Gattorno Consultant for: SOBI,Speaker Bureau of: SOBI, A. Ravelli: None Declared

**Table 1 (abstract P2066).** Best-fitting model obtained through logistic regression procedures

Baseline explanatory variable	OR (95% CI)	P^£^
Diseaseduration ≤ 3.9 years	6.78 (1.30-35.27)	0.012
Active joint count ≤ 10	8.25 (1.26-53.91)	0.012
Ferritin > 444 ng/dL	4.75 (1.16-19.50)	0.020
Systemicmanifestationscore > 3	6.44 (1.38-24.62)	0.007

#### P2067 Canakinumab treatment in adult-onset Still’s disease: case series

##### Serdal Ugurlu^1^, Gul Guzelant^1^, Berna Yurttas^1^, Bilgesu Ergezen^1^, Ediz Dalkilic^2^, Timucin Kasifoglu^3^, Burcu Yagiz^2^, Huri Ozdogan^1^

###### ^1^Istanbul University, Cerrahpasa Medical Faculty, Department of Internal Medicine, Division of Rhematology, Cerrahpaşa Medical Faculty, Istanbul; ^2^Uludag University, Medical Faculty, Department of Internal Medicine, Division of Rhematology, Uludag Medical Faculty, Bursa; ^3^Osmangazi University, Medical Faculty, Department of Internal Medicine, Division of Rheumatology, Osmangazi Medical Faculty, Eskisehir, Turkey

####### **Correspondence:** Serdal Ugurlu

**Introduction:** In Adult-onset Still’s disease (AOSD), cases refractory to typical DMARDs, Canakinumab (an anti-IL-1ß monoclonal antibody) has been reported to be effective in a limited number of refractory cases (1).

**Objectives:** The aim of this retrospective study was to represent AOSD patients treated with Canakinumab in 3 centers.

**Methods:** The follow up data of 11 AOSD patients (8 female, 3 male), who were followed up in outpatient clinics of 3 tertiary centers were reviewed retrospectively. The initial characteristics and follow up findings were reported.

**Results:** The mean timespan between the initial diagnosis and Canakinumab treatment 43.2 ± 28 months (mean ± SD). Before the onset of Canakinumab therapy, all patients were exposed to methotrexate, 1 to leflunomide, 9 to Tocilizumab and 8 to Anakinra. As for the biologic agents, 3 patients were also treated beforehand with Infliximab, 2 with Adalimumab, 3 with Etanercept and 2 with Rituximab. Canakinumab therapy was initiated in all patients with the indication of refractory disease under other medications, except for the one in whom neutropenia became evident under anakinra. The mean number of Canakinumab injections was 11,8 ± 6. The mean follow-up period of patients treated with Canakinumab was 42.2 ± 31 months. Five of 8 patients are still being treated with Canakinumab of 150 mg/month, one of 300 mg/month and two of 150 mg/every 2 months. One patient had a single injection and was fully controlled. The mean ferritin measure of 9 patients was reduced from 1292.3 ± 1530 ng/ml to 218.1 ± 327.1 ng/ml following the Canakinumab therapy (p=0.056). The mean of patient-reported global visual analogue scale (PG-VAS) scores was reduced from 7.9 ± 2.4 to 1.4 ± 2 with Canakinumab (p<0.0001). Mean Erythrocyte sedimentation rate (ESR) was reduced from 51 ± 39.4 to 18.8 ± 21.6 with the help of Canakinumab therapy (p = 0.036). Seven patients are still on prednisolone at a maximum dose of 10 mg/day. The indication of therapy termination in the remaining 1 patient was the diagnosis of tuberculosis at 9th month of the treatment despite isoniazid prophylaxis. The patient was also treated with multiple biological agents beforehand, therefore it is not easy to conclude that treatment with Canakinumab induces tuberculosis flares.

**Conclusion:** Canakinumab treatment seems to be effective in refractory AOSD patients who were previously treated with various agents. We state that an IL-1 blocking agent, Canakinumab is a relatively safe and effective alternative in managing refractory AOSD cases. On the other hand, randomized controlled trials are needed to further investigate the role of Canakinumab in these cases as well as its use as the first choice of biologic agents.

**REFERENCE**

1-Kontzias A, Efthimiou P. The use of Canakinumab, a novel IL-1β long-acting inhibitor, in refractory adult-onset Still's disease. Semin Arthritis Rheum. 2012;42(2):201-5.

**Disclosure of Interest**

None Declared

**Table 1 (abstract P2067).** See text for description

	Before Canakinumab	After Canakinumab	p
Ferritin value (mean ± SD) (ng/ml)	1292.3 ± 1530	218.1 ± 327.1	0.056
Patient-reported global visual analogue scale (PG-VAS)	7.9 ± 2.4	1.4 ± 2	<0.0001
Mean Erythrocyte sedimentation rate (ESR)	51 ± 39.4	18.8 ± 21.6	0.036

### Therapy of systemic auto-inflammatory diseases

#### P2068 Microbiota transplant to control inflammation in a NLRC4-related disease patient with recurrent hemophagocytic lymphohistiocytosis (HLH)

##### Claudia Bracaglia^1^, Giulia Marucci^1^, Federica Del Chierico^2^, Alessandra Russo^2^, Manuela Pardeo^1^, Antonella Insalaco^1^, Giusi Prencipe^1^, Ivan Caiello^1^, Sarka Fingerhutova^3^, Pavla Dolezalova^3^, Fabrizio De Benedetti^1^, Lorenza Putignani^2,4^

###### ^1^Division of Rheumatology; ^2^Unit of Human Microbiome, IRCCS Ospedale Pediatrico Bambino Gesù, Rome, Italy; ^3^Paediatric Rheumatology and Autoinflammatory Diseases Unit, General University Hospital, Prague, Czech Republic; ^4^Unit of Parasitology, IRCCS Ospedale Pediatrico Bambino Gesù, Rome, Italy

####### **Correspondence:** Claudia Bracaglia

**Introduction:** The NLRC4 inflammasome activation is essential in host defence, particularly against enteric pathogens. Gain-of-function mutations in *NLRC4* are associated with a distinct autoinflammatory syndrome characterized by enterocolitis and recurrent HLH.

**Objectives:** To report safety and efficacy of faecal microbiota transplantation (FMT) in a patient carrying a *de novo* missense mutations in *NLRC4*, with gut inflammation and gut colonization by multi-drug resistant (MDR) pathogens.

**Methods:** Subject and donors were screened for potential FMT according to Cammarota *et al*. (Gut, 2017) with modifications introduced by Ospedale Pediatrico Bambino Gesù Committee for paediatric FMT procedures. Culture-based and NGS-based microbiota profiling were performed to address MDR persistence and microbial associated communities coupled to inflammatory profiling. After wide donor screening, a universal adult donor was selected and infusion performed twice by colonoscopy from fresh and frozen faeces, respectively. A one year FMT follow up was ensured to address clinical outcomes.

**Results:** A Caucasian 16 months old boy presented, from 1 month of life, with recurrent HLH, diarrhoea and vasculitic skin lesions, secondary to a de novo missense mutation in *NLRC4* (I343N). His disease was not controlled despite treatment with repeated high dose of intravenous methylprednisolone pulses and chronic daily glucocorticoid therapy, cyclosporine-A (5 mg/kg) and anakinra (ranging from 5 to 25 mg/kg/day). Because of recurrent HLH episodes, the patient was treated with emapalumab (anti-IFNgamma antibody) for three months, with HLH control. However, the patient presented persistent diarrhoea with gut inflammation and colonization by MDR pathogens (*Enterobacter cloacae* and *Enterococcus faecalis*), leading by translocation to systemic infections and, hence, HLH reactivation. Moreover, he developed severe intestinal meteorism with subsequent paralytic-ileus that required ileostomy. Based on the hypothesis that his intestinal dysbiosis concurred in the persistent gut inflammation related to his underlying disease, he received a first FMT with initial clinical improvement. Therefore, ileal anastomosis was performed with recanalization, and a second FMT was performed a month later. The patient did not develop any complication. The stool cultures no longer showed the presence of enteropathogenic germs and since the last FMT (12 months of follow-up) he did not present any symptom of gut inflammation or HLH reactivations.

**Conclusion:** *NLRC4*-related disease is typically characterized by enterocolitis and *NLRC4*, highly expressed in intestinal macrophages, is critical for pathogen restriction by phagocytes in the gut lamina propria. The chronic gut inflammation of *NLRC4*-patients is a stimulus for systemic inflammation. Moreover, pathogens present in the gut can trigger HLH episodes. A presence of a selected microbiota from appropriate donor can decrease gut inflammation. FMT may represent a valid and safe therapeutic option in *NLRC4*-patients in order to reduce chronic inflammatory *stimuli*.

**Consent for publication has been obtained from patient**

Yes

**Disclosure of Interest**

C. Bracaglia: None Declared, G. Marucci: None Declared, F. Del Chierico: None Declared, A. Russo: None Declared, M. Pardeo: None Declared, A. Insalaco: None Declared, G. Prencipe: None Declared, I. Caiello: None Declared, S. Fingerhutova: None Declared, P. Dolezalova: None Declared, F. De Benedetti Grant / Research Support from: Novartis, Novimmune, Hoffmann- La Roche, SOBI, AbbVie, Pfizer, L. Putignani: None Declared

#### P2069 Emapalumab, an anti-interferon gamma monoclonal antibody in two patients with NLRC4- related disease and severe hemophagocytic lymphohistiocytosis (HLH)

##### Claudia Bracaglia^1^, Giusi Prencipe^1^, Antonella Insalaco^1^, Ivan Caiello^1^, Giulia Marucci^1^, Manuela Pardeo^1^, Raffaele Pecoraro^2^, Pavla Dolezalova^3^, Sarka Fingerhutova^3^, Maria Ballabio^4^, Cristina de Min^4^, Fabrizio De Benedetti^1^

###### ^1^Division of Rheumatology, IRCCS Ospedale Pediatrico Bambino Gesù; ^2^Pediatric Department, La Sapienza University of Rome, Rome, Italy; ^3^Paediatric Rheumatology and Autoinflammatory Diseases Unit, General University Hospital, Prague, Czech Republic; ^4^Novimmune, S.A., Geneva, Switzerland

####### **Correspondence:** Claudia Bracaglia

**Introduction:** Interferon gamma (IFNγ) plays a pathogenic role in primary and secondary HLH. An ongoing phase 2/3 trial with emapalumab in primary HLH provides encouraging preliminary data and a pilot trial in MAS in the context of sJIA has just been initiated. Gain-of-function mutations in NLRC4 are associated with a distinct autoinflammatory syndrome, with recurrent HLH.

**Objectives:** To report safety and efficacy of emapalumab treatment in two patients carrying de novo missense mutations in NLRC4, with severe early onset HLH.

**Methods:** Cytokine levels were measured by multiplex assay and by specific ELISAs and expression of IFNγ in freshly isolated PBMCs by cytometry.

**Results: Pt 1.** Caucasian male, presented, at age 20 days, fever and rash and progressively developed clinical and laboratory features of HLH leading to multi-organ failure. A de novo missense mutation in NLRC4 (T337N) was found. High-dose glucocorticoids and cyclosporine-A (CyA) led only to partial improvement. A sepsis triggered HLH reactivation. Emapalumab was started (compassionate use) on background of dexamethasone (13.6 mg/m2) and CyA. After 3 months, the child was discharged in excellent conditions (prednisone 0.3 mg/kg). Infections resolved during treatment with emapalumab. After 7 months of emapalumab treatment, all therapies, including emapalumab, were discontinued, without clinical or laboratory signs of HLH reactivation. **Pt 2.** This is 16 months old Caucasian boy with recurrent HLH and vasculitic skin lesions, since 1 month of life, secondary to a de novo missense mutation in NLRC4 (I343N). His disease was not controlled despite treatment with repeated methylprednisolone pulses and chronic daily glucocorticoid therapy, CyA (5 mg/kg) and anakinra (ranging from 5 to 25 mg/kg/day). When anakinra was withdrawn prior to start emapalumab he immediately developed high-grade fever, skin rash with vasculitic lesions and diarrhoea with laboratory features of HLH. Emapalumab was started (compassionate use) on background of methylprednisolone and CyA with rapid resolution of fever and improvement in biochemical parameters.During emapalumab treatment the patient resolved his initial HLH flare and presented two HLH episodes of mild intensity controlled with moderate intensification of glucocorticoid therapy. These episodes were triggered by systemic infections caused by pathogens translocated from the gut. His diarrhoea persisted with low grade inflammation; emapalumab was eventually withdrawn after 3 months. His subsequent course was characterized by additional mild episodes of MAS. In both patients increased production of IFNγ was demonstrated by high levels of CXCL9 (pt.1: 5670 pg/ml, pt.2: 3310 pg/ml), a chemokine induced specifically by IFNγ, by increased IFNγ expression in NK cells and CD8T cells, and by presence of high levels of total IFNγ bound to circulating emapalumab.

**Conclusion:** In both patients with HLH secondary to NLRC4-related disease, treatment with emapalumab was well tolerated, no safety concerned emerged, normalization of all HLH clinical and laboratory abnormalities was achieved. Pt. 1 showed no disease reactivation even in the absence of treatments. In pt. 2 IFNγ neutralization has provided control of HLH, while his underlying disease and, in particular, gut inflammation and gut colonization by MDR pathogens remained unchanged.

**Consent for publication has been obtained from patient**

Yes

**Disclosure of Interest**

C. Bracaglia: None Declared, G. Prencipe: None Declared, A. Insalaco: None Declared, I. Caiello: None Declared, G. Marucci: None Declared, M. Pardeo: None Declared, R. Pecoraro: None Declared, P. Dolezalova: None Declared, S. Fingerhutova: None Declared, M. Ballabio Employee of: Novimmune S.A., C. de Min Employee of: Novimmune S.A., F. De Benedetti Grant / Research Support from: Novartis, Novimmune, Hoffmann- La Roche, SOBI, AbbVie, Pfizer

#### P2070 IL-1 blockade in paediatric recurrent pericarditis: a multicentric retrospective study of the Italian cohort

##### Roberta Caorsi, Antonella Insalaco, Chiara Longo, Giorgia Martini, Marco Cattalini, Rita Consolini, Giovanni Filocamo, Alessandro Rimini, Silvia Federici, Camilla Celani, Maria Cristina Maggio, Micol Romano, Barbara Teruzzi, Andrea Taddio, Angela Miniaci, Silvana Martino, Francesco Latorre, Alessandro De Fanti, Giulio Cavalli, Berbara Bigucci, Antonio Brucato, Fabrizio De Benedetti, Marco Gattorno and The Italian study group on anti-IL-1 drugs’ efficacy in recurrent pericarditis

###### Center of Autoinflammatory Diseases and Immunodeficiencies, Department of Pediatrics and Rheumatology, Istituto G. Gaslini, Genova, Italy

####### **Correspondence:** Roberta Caorsi

**Introduction:** Acute pericarditis is an inflammatory condition causing the occurrence of pericardial effusion. In a third of patients, the disease is recurrent. First line treatment of idiopathic pericarditis consists in non-steroidal anti-inflammatory drugs (NSAIDs) and colchicine; glucocorticoids represent the second line treatment in resistant or intolerant cases. A recent clinical trial has enlightened the effectiveness of anakinra in adults and paediatric patients with colchicine-resistant recurrent pericarditis.

**Objectives:** To describe the clinical characteristics and response to treatment in a cohort of paediatric patients with recurrent pericarditis treated with IL inhibitors.

**Methods:** paediatric patients with recurrent pericarditis followed at 19 Italian centers of paediatric rheumatology or cardiology and treated with IL1 inhibitors were included in the study. Demographic, clinical and response to treatment data were retrospectively collected.

**Results:** 55 patients were included in the study. The mean age at onset of the first episode of pericarditis was 12.53 years. The mean number of relapses of pericarditis before the beginning of treatment with IL1 inhibitors was of 3.4 (range 1 -10). 53 out of 55 patients had previously received treatment with NSAIDS and 44 with colchicine. 44 patients received steroidal treatment: 2 of them displayed a steroidal-resistance and 39 steroidal-dependence with reoccurrence of the symptoms following any attempt to reduce or withdraw this treatment. Anakinra (mean dosage of 1.67 mg/kg/day) was used as first Il-1 inhibitor in 54 of the 55 patients, Canakinumab (150 mg every 4 weeks) in one. 53 out of 54 patients treated with anakinra displayed a complete clinical response to treatment within a mean of 2 days (range 1-7): NSAIDs, glucocorticoids and colchicine were withdraw in 25 out of 26, 31 out of 35 and 15 out of 32 patients respectively. 50 of 54 patients displayed a complete response; among these, three were switched to Canakinumab, 17 patients continued treatment at the same dosage while in 30 patients a reduction of treatment was attempted. 12 patients presented, during anakinra tapering, a disease flare, promptly resolved after an increasing of the dosage. The remaining 18 patients did not present any flare despite the reduction of the drug. Anakinra was withdrawn in 16 patients, with recurrence of the symptoms in 11. 5 patients were treated with Canakinumab: 1 as first anti-IL1 drug, 4 were switched from anakinra (two for poor compliance, one for side effects and one for incomplete control of the disease). 2 out of five patients had a complete control of the diseases, 2 patients discontinued the treatment because of inefficacy and 1 patient required low dose of glucocorticoids to control the disease. At last follow-up 34 patients were on anakinra, 7 on anakinra and colchicine, 2 on canakinumab, 1 oncanakinumab plus colchicine and NSAIDs. In 9 patients all treatments were withdrawn for complete control of the disease.

**Conclusion:** This study confirm the effectiveness of IL-1 blockade in paediatric patients with recurrent pericarditis. However most of the patients require prolonged treatment to maintain clinical remission. Moreover, in our cohort of patients the rate of response was higher for anakinra then for canakinumab, suggesting a possible role of IL1α in the pathogenesis of this condition.

**Disclosure of Interest**

None Declared

#### P2071 Efficacy and safety of anakinra in the treatment of inflammatory heart failure in myocarditis

##### Giacomo De Luca, Corrado Campochiaro, Giulio Cavalli, Silvia Sartorelli, Lorenzo Dagna

###### Unit of Immunology, Rheumatology, Allergy and Rare Diseases, IRCCS San Raffaele Hospital, Vita-Salute San Raffaele University, Milan, Italy

####### **Correspondence:** Giacomo De Luca

**Introduction:** Virus-negative myocarditis (VNM) is a severe, inflammatory heart disease with a poor prognosis, and is a leading cause of inflammatory dilated cardiomyopathy(i-DCM). Therapies are limited. Preliminary data indicate that interleukin-1 (IL-1) plays a key role in the initiation and maintainance of the inflammatory heart response, sustaining an auto-inflammatory cycle [1,2].

**Objectives:** to evaluate the efficacy and safety of anakinra (ANK) in improving Left Ventricular Ejection Fraction (LVEF) on transthoracic echocardiography (TTE) and other cardiovascular parameters in patients with VNM.

**Methods:** Biopsy-proven VNM patients were enrolled and treated with ANK 100 mg daily subcutaneously. All patients also received treatment with the maximum tolerated dose of any beta blockers and ACE-inhibitors, according to current guidelines. At baseline and 8±4 weeks after ANK therapy initiation, all patients underwent a full evaluation with assessment of their functional status (New York Heart Association [NYHA]), measurement of high-sensitive troponin T (hs-TnT) and NT-proBNP serum levels, electrocardiography (ECG), 24h-ECG Holter, TTE and cardiac magnetic resonance (CMR). Any myocarditis-related complication, cardiovascular deaths and adverse events (AEs) were recorded during follow-up. Continuous variables were assessed with the Wilcoxon signed-rank test for non-parametric tests and a p value<0.05 was considered statistically significant.

**Results:** eleven patients with EBM-proven myocarditis were enrolled and treated with ANK. Nine patients received ANK as first line therapy, and in 5 cases ANK was used as monotherapy; ANK was combined with prednisone (mean dose 31,7±16,7 mg daily) in 6 patients, 5 of them were concomitantly treated with azathioprine. Demographic and baseline clinical characteristics of our cohort are summarized in table 1. Mean LV-EF on TTE at baseline was 38,7%±19,6, with comparable findings on CMR (36,45%±18,0), and 8 patients (72.7%) had a depressed LV-EF(<55%). At baseline, mean levels of hs-TnT and NT-proBNP were 150,0 ± 153,9 ng/L and 6968,8 ± 10788,4 pg/ml, respectively. Hs-TnT and NT-proBNP levels were elevated in 10 (90.9%) and 9 patients (81.8% respectively. At 8 weeks, LV-EF improved in 10 patients (90.9%). The LV-EF increase was >10% in 5 patients (45.5%) and between 5-10% in 5 cases (45.5%); only 1 patient showed a <10% LV-EF decrease. Mean LV-EF at the end of follow-up improved to 49,4%±10,8 (p=0.059). When evaluating the 8 patients with baseline reduced LV-EF, the LV-EF improvement was statistically significant (baseline 29,25% ±12,9; after ANK 45.2% ±9.2, p=0.025). The LV-EF amelioration was paralleled by clinical improvements in all patients, since the majority of them (90.9%) were in NYHA class I-II at the end of follow-up(vs 27.3% at baseline). Consistently, hs-TnT declined after 8 weeks (64,6±100,7 ng/L,p=0,028), and a similar trend was observed for NT-proBNP, even though this not reach statistical significance(2582,6±5048,1 pg/ml,p=0,06). We did not observe any myocarditis-related death or complications, nor any ANK-related AEs.

**Conclusion:** Our pilot study supports the efficacy and safety of anakinra in the treatment of inflammatory heart failure in VNM and provides the first clinical evidence to support the therapeutic blockade of IL-1 in myocarditis.

**Disclosure of Interest**

None Declared

**Table 1 (abstract P2071).** See text for description

Females/males, n	5/6
Mean age, years	46,2±12,2
Clinical onset, n(%)	
*Heart failure (LV-EF<55%)*	8(72.7)
*Chest pain*	2(18.2)
*Dyspnea*	1(9.1)
NYHA class III-IV, n(%)	8(72.7)
EBM classification*, n(%)	
*i-DCM*	6(54.5)
*Acute/active/chronic VNM*	3(27.3)/1(9.1)/1(9.1)

* according to histologic and immunohistochemical criteria ^*[3-4]*^

#### P2072 Efficacy, safety and cost-effectiveness of a vial-sharing programme for canakinumab treatment for paediatric patients with cryopyrin-associated periodic syndrome

##### Abdulkadir A. Elmi^1^, Karen Wynne^2^, Iek L. Cheng^2^, Despina Eleftheriou^2^, Helen J. Lachmann^3^, Philip N. Hawkins^3^, Paul Brogan^1,2^

###### ^1^Infection Inflammation and Rheumatology Section, University College London Institute of Child Health; ^2^Department of Paediatric Rheumatology, Great Ormond Street Hospital NHS Foundation Trust; ^3^National Amyloidosis Centre, UCL Centre for Amyloidosis and Acute Phase Proteins, London, United Kingdom

####### **Correspondence:** Abdulkadir A. Elmi

**Introduction:** Cryopyrin-associated periodic syndrome (CAPS) is a rare autoinflammatory disease, caused by gain of function mutation in *NLRP3* resulting in excess production of interleukin-1 (IL-1). Canakinumab is a human monoclonal antibody against IL-1β, licensed for the treatment of CAPS**.**

**Objectives:** To describe the feasibility and cost-effectiveness of a canakinumab vial-sharing programme for paediatric patients with CAPS.

**Methods:** Retrospective case series and clinical service description of a national specially commissioned CAPS clinic at Great Ormond Street Hospital (GOSH). Efficacy was assessed using the CAPS disease activity score (DAS) and serum amyloid A protein (SAA). Adverse events were collected to determine safety. The number of canakinumab vials saved were considered when investigating the cost-effectiveness of vial-sharing.

**Results:** Nineteen/20 (95%) of our paediatric patients achieved minimally active clinical disease activity with canakinumab monotherapy; and 75% achieved both minimally active clinical disease and serological remission using a pre-specified definition based on the CAPS DAS and SAA level. Canakinumab was well tolerated, with only one child developing an infection requiring hospitalisation during the study.Canakinumab vial sharing resulted in 117 vials of canakinumab saved over a 24-month period, equating to a direct drug-related cost saving of £1,385,821, and a conservative estimated 5-year cost-saving of £3,464,552.50.

**Conclusion:** We provide further evidence for the efficacy and safety of canakinumab in children with CAPS, and highlight the cost-effectiveness of a vial-sharing programme for this high cost medicine. We suggest that this could have important implications for the delivery of other high cost medicines used in paediatric practice.

**Disclosure of Interest**

None Declared

#### P2073 Anti-IL-1 therapies in patients with familial Mediterranean fever related AA-amyloidosis

##### Serdal Ugurlu^1^, Bilgesu Ergezen^2^, Oguzhan Selvi^1^, Bugra H. Egeli^1^, Huri Ozdogan^2^

###### ^1^Division of Rheumatology, Department of Internal Medicine, Cerrahpasa Medical Faculty; ^2^University of Istanbul - Cerrahpasa, Istanbul, Turkey

####### **Correspondence:** Bilgesu Ergezen

**Introduction:** The most devastating complication of Familial Mediterranean Fever (FMF) is secondary AA amyloidosis and is still a problem in many cases. Efforts have been paid to prevent AA amyloidosis or to control an already formed amyloidosis in order to prevent organ function loss, particularly renal insufficiency. There are emerging therapies in FMF related amyloidosis.

**Objectives:** We aimed to evaluate the role of anti-IL-1 regimens, anakinra and canakinumab regarding their efficacy and safety in FMF related amyloidosis.

**Methods:** In this single center study, 44 patients diagnosed with FMF and had a histologically proven diagnosis of amyloidosis, were exposed to Anakinra or canakinumab. Patients who received anti IL-1 therapy for at least 3 months were evaluated for treatment response. All of the patients recruited in this study received colchicine and are either resistant or intolerant. Patients were questioned with regards to FMF related characteristics and comorbidities. MEFV gene status, laboratory measures, side events and therapy outcomes were tested and followed.

**Results:** Four patients were excluded because they received treatment less than 3 months. Among remaining 40 patients, anakinra was switched to canakinumab due to allergic reactions or insufficient response in 15 patients. There are 16 patients still on anakinra and 10 with canakinumab after a mean of 25.92±11.31 months. The patients were divided into two subgroups with regards to their initial creatinine levels: Group 1 creatinine levels below 1.5 mg/dL and Group 2 above 1.5 mg/dL. The analysis of clinical outcome is summarized in Table 1. The decrease in proteinuria is more modest (p=0.01) in Group 1 in comparison with Group 2 (p<0.01). The decrease in the mean patient global assessment scores was significant for both groups. It decreased from 6.82 to 1.2 in Group 1 and from 6 to 2.5 in Group 2. At the time of this review 8 patients were still on hemodialysis. Three patients had renal transplantation. The only side effect observed was injection-site reaction in patients receiving anakinra (n=3). Patients on canakinumab developed pneumonia, flare of psoriasis, and lichen planus one each.

**Conclusion:** We suggest that anti-IL-1 therapies are effective and tolerable in FMF related amyloidosis by controlling the acute phase response, as well as improving renal function and quality of life. These agents are particularly effective when administered during the early stages of renal disease. The anti-IL-1 agents are well tolerated and the related side effects are generally reversible.

**Disclosure of Interest**

None Declared

**Table 1 (abstract P2073).** The parameters of renal functions and acute phase reactants in patients with initial creatinine level below 1.5mg/dL (Group 1) and above 1.5mg/dL (Group 2)

	Before treatment	After treatment	p	Before treatment	After treatment	p	Before treatment	After treatment	p
Group 1	Anakinra(n= 19)			Canakinumab (n=10)			All anti IL-1 (n=23)		
Creatinine (mg/dl)	0,9 ± 0,28	0,9 ± 0,44	<0,01	1,35 ± 1,57	1,78 ± 1,93	0,01	0,8 ± 0,25	1,3 ± 1,5	<0,01
Proteinuria (mg/day)	2686 ± 3257	2328 ± 4458	<0,01	6000 ± 5550	3730 ± 5030	<0,01	2780 ± 3323	1365 ± 2365	<0,01
ESR(0-20 mm/h)	43,5 ± 31,3	19,3 ± 26,4	<0,01	51,22 ± 33,54	17,1 ± 31,2	<0,01	44,8 ± 31,1	17,6 ± 31,1	<0,01
Group 2	Anakinra(n= 13)			Canakinumab (n=9)			All anti IL-1 (n=17)		
Creatinine (mg/dl)	3,3 ± 2,4	3,4 ± 2,4	<0,01	3,1 ± 2,4	3,2 ± 2,5	<0,01	3,54 ± 2,62	3,5 ± 2,59	<0,01
Proteinuria (mg/day)	5482 ± 8040	3651 ±4635	0,01	7670 ± 7600	7585 ± 6830	0,01	5347 ± 7377	3130 ± 4764	0.01
ESR (0-20 mm/h)	56,8 ± 35,1	31,3 ± 17,8	<0,01	51,22 ± 33,54	34,55 ± 27,19	<0,01	56,8 ± 34,6	38,8 ± 27,6	<0,01

#### P2074 Anti-interleukin-1 prescription in adult patients with familial Mediterranean fever: a real-life study from the French national reference center

##### Antoine Fayand^1^, Léa Savey^1^, Véronique Hentgen^2^, Pierre Quartier^3^, Claude Bachmeyer^1^, Joris Galland^1^, Serge Amselem^4^, Camille Louvrier^4^, Irina Giurgea^4^, Gilles Grateau^1^, Sophie Georgin-Lavialle^1^ and French National Reference Center for Autoinflammatory Diseases

###### ^1^Internal Medicine, AP-HP, Tenon Hospital, PARIS; ^2^General Pediatry, CH Versailles, André Mignot, Versailles; ^3^Pediatric Rheumatology, AP-HP, Necker Hospital; ^4^Medical Genetics, AP-HP, Trousseau Hospital, PARIS, France

####### **Correspondence:** Sophie Georgin-Lavialle

**Introduction:** Familial Mediterranean fever (FMF) is the most common monogenic auto-inflammatory disease in the world. Long-term treatment of the disease is based on daily intake of colchicine, which has proven its effectiveness in the occurrence of acute attacks and AA amyloidosis. In cases of poor tolerance, ineffectiveness or contraindication, anti-interleukin-1 (IL-1) biotherapies have been effectively tested.

**Objectives:** To report on the experience of using IL-1 biotherapies in real life among FMF patients followed in the French National Reference Centre (NRC).

**Methods:** All French adult FMF records were reviewed; for those receiving or having received IL1 biotherapy, the all medical file was available.

**Results:** Among the 603 FMF patients, 3.5% (n=21) were included (12 women/9 men); 81% displayed M694V homozygous *MEFV* mutation and 13 had AA amyloidosis. Anti-IL1 biotherapy used were: anakinra (n=20) and canakinumab (n=2)0. Colchicine was maintained in 18 patients at a median dose of 1 mg/d.

The prescription of IL1 biotherapy was justified by:

1/ AA amyloidosis responsible for renal failure that did not allow the colchicine dose to be increased: n=13 (2.2% of the overall FMF population);

2/ Insufficient control with colchicine: n=4 (0.7%)

3/ Coexistence of an inflammatory or neoplastic disease participating in the poor control of FMF: n=4 (0.7%);

3/ In one patient, renal failure on AA amyloidosis and colchicine resistance coexisted.

In all our patients, the use of anti-IL1 has allowed clinical and biological control of their disease. There was no evidence of poor tolerance of anti-IL1 except for reactions at injection sites for anakinra and weight gain.

**Conclusion:** In our clinical experience, the first cause of prescription of anti-IL1 biotherapy is the existence of AA amyloidosis with renal failure, which corresponds to more than half of the indications and 2.2% of FMF patients followed on our CNR. Colchicine resistance is lower than expected, 1% of our patients, compared to 5 and 10% that were recently published. This can be explained by potential “false resistances” to be eliminated as a priority: either poor compliance or insufficient dosage. Finally, in the case of true resistance to colchicine, our patients displayed another inflammatory disease associated to FMF.

In conclusion, situations constituting an indication for IL1 biotherapies in FMF are rare (3.5% of patients) and their prescription requires great vigilance on the part of the clinician, which is why they should always be the subject of a collegial discussion, after having eliminated all organic causes of FMF deregulation.

**Disclosure of Interest**

A. Fayand: None Declared, L. Savey: None Declared, V. Hentgen: None Declared, P. Quartier: None Declared, C. Bachmeyer: None Declared, J. Galland: None Declared, S. Amselem: None Declared, C. Louvrier: None Declared, I. Giurgea: None Declared, G. Grateau: None Declared, S. Georgin-Lavialle Consultant for: SOBI and NOVARTIS

#### P2075 Effectiveness of anti-IL6 drug (Tocilizumab) in pulmonary alveolar proteinosis associated to severe persistent systemic inflammation: a case report

##### Ilaria Gueli^1^, Roberta Caorsi^1^, Oliviero Sacco^2^, Donata Girosi^2^, Gian Michele Magnano^3^, Angelo Ravelli^1^, Marco Gattorno^1^, PaoloPicco^1^

###### ^1^Rheumatology Unit; ^2^Pulmonology Unit; ^3^Radiology Unit, Giannina Gaslini Institute, Genoa, Italy

####### **Correspondence:** Ilaria Gueli

**Introduction:** Pulmonary alveolar proteinosis (PAP) consists in accumulation of pulmonary surfactants in the alveolar space. It is supposed to be caused by reduced number and/or impaired function of alveolar macrophages, with resultant reduced clearance of surfactant. It could be an autoimmune, a congenital or a secondary disease associated to a very large spectrum of pathologies and has recently been described as an extremely rare but potentially fatal complication of systemic juvenile idiopathic arthritis (sJIA). We describe a case of a very severe and persistent systemic inflammation complicated by PAP who had a dramatic recovery and also an improvement of lung injury, under anti IL6-treatment with Tocilizumab.

**Results:** Case report: A 14-year-old boy has been affected by a very severe systemic inflammation (flares of fever, generalized lymph-nodes enlargement, rash, fugacious small joints arthritis, increase of blood acute phase reactants), with features consistent with sJIA, since the age of 11. Once malignancies were ruled out (bone marrow examination; lymph-node biopsy; total body PET), systemic steroid was started (prednisone 1mg/kg/die) with only a transient benefit, as he relapsed during the progressive tapering. Among the tried treatments used in association with steroids (Cyclosporine; Micophenolate Mofetil; Anakinra), only high-dose Canakinumab (300 mg/4 weeks) lead to a complete disease control. In the following months steroid tapering was performed with disease flares at low steroids doses. Due to parental decision, Canakinumab was discontinued and alternative medicine was followed for at least nine months. During this period the patient presented a very severe inflammatory relapse, which was complicated by pulmonary manifestations, such as dry cough and dyspnea. Radiological images (chest-TC), functional test (DLCO with moderate impairment of predicted value, 49%; spirometry with mild restrictive alteration) and, finally, lung biopsy, lead to the diagnosis of PAP. Anti-IL6 drug (Tocilizumab, 10mg/kg), was started with dramatic response both on systemic inflammation and on pulmonary impairment (cough resolution, no dyspnea, DLCO with mild impairment of predicted value, 70%). Maintenance therapy with Tocilizumab led to long-term clinical stability.

**Conclusion:** Secondary PAP could be a severe complication of persistent and uncontrolled systemic inflammation and may respond to treatment of the underlying disease. Both IL-1 and IL-6 inhibitors are effective in systemic inflammation diseases such as sJIA; though, their possible direct causative role in the occurrence of PAP has been proposed. Anyway, the case we presented showed anti IL-6 treatment (Tocilizumab) being effective either on systemic inflammation and on lung impairment, leading to a long-term control of such a severe respiratory complication. In conclusion anti IL-6 treatment could be an effective choice in severe lung complications of systemic inflammatory diseases, such as PAP.

**Consent for publication has been obtained from patient**

Yes

**Disclosure of Interest**

None Declared

#### P2076 Is fibrodysplasia ossificans progressiva an interleukin-1 driven auto-inflammatory syndrome?

##### Ruby Haviv^1^, Veronica Moshe^1^, Noa Rabinowicz^1^, Giusi Prencipe^2^, Fabrizio De Benedetti^2^, Yosef Uziel^1^

###### ^1^Pediatric Rheumatology Unit, Department of Pediatrics, Meir Medical Center and Sackler School of Medicine (Tel-Aviv University), Kfar Saba, Israel; ^2^Division of Rheumatology, Bambino Gesù Children’s Hospital IRCCS, Rome, Italy

####### **Correspondence:** Ruby Haviv

**Introduction:** *Fibrodysplasia ossificans progressiva* (FOP) is a very rare condition. It is the most catastrophic form of heterotopic ossification, caused by ongoing intra-cellular signaling through the bone morphogenic protein (BMP) pathway, which results in chondrocyte formation and complete ossification of soft tissues. The *ACVR1/ALK2* gene produces the Activin 1 receptor, which serves as the heterodimer of the BMP type I receptor. The typical R206H activating mutation occurs within the highly conserved glycine-serine region of the cytoplasmic domain, adjacent to a protein kinase domain.

Painful, soft tissue swellings usually appear by the age of 3-4 years, but the typical, bilateral deformity of the large toes can be noted at birth. Average life expectancy is 45 years. Morbidity and mortality are commonly related to ankylosis of the temporomandibular joints and thoracic insufficiency syndrome.

Currently, no treatment has proven effective. Some use anti-leukotrienes, non-steroidal anti-inflammatory drugs, and other agents, as prophylaxis. When swelling appears, corticosteroids, along with bisphosphonates, are usually used. Anti-TNFa agents were reported unsuccessful.

The recurrent paroxysmal appearance of local tender swellings, with erythematous skin, which partially react to anti-inflammatory agents, accompanied by elevated inflammatory markers during flares, suggest that FOP might be an auto-inflammatory disease. We hypothesized that treatment with anti-interleukin-1 (IL-1) agents helps ameliorating progression of this devastating disease, and report our experience.

**Objectives:** To try lowering the rate of FOP paroxysms, and/or limit symptoms and residual lesions, with anti-IL-1 agents in one patient with FOP.

**Methods:** Patient’s clinical data and blood IL-1b levels were analyzed to characterize the effects of anti-IL-1 treatments in ameliorating the natural progression of FOP

**Results:** A 13.5-year-old Arab male was diagnosed with FOP, clinically and genetically (the typical R206H mutation was found). Treatment with high-dose corticosteroids, pamidronate infusions, and daily celecoxib and monteleukast did not affect the course of the disease, and new swellings, lasting 5-7 days, continued to appear, at an approximate rate of one new swelling every 8 days. Anakinra treatment was commenced (100mg/day, as the patient weighed more than 100 kg). After marked improvement was noted during a 2-month period, with no side effects, he was switched to monthly canakinumab (300mg/dose) for 5 months. Markedly lower rate of flare-ups was documented (a new swelling every 22-25 days), with shorter duration (2-3 days). The swellings were smaller and easier to control, with shorter courses of methylprednisolone during flares. Treatment was withdrawn, but renewed after 6.5 weeks, due to appearance of a large swelling below his left scapula. High plasma levels of IL-1b were found during this flare (18.17-21.52pg/ml). In contrast, the 3 previous blood samples, obtained during treatment with anakinra or canakinumab, showed undetectable IL-1b levels (<0.125pg/ml), (Figure).

**Conclusion:** This case may suggest, that FOP flares are mediated by IL-1, and that FOP may be included under the umbrella of auto-inflammatory syndromes. Anti-IL-1 agents might have a role in ameliorating the natural progression of FOP. Further exploration and international experience with additional FOP patients are needed.

**Consent for publication has been obtained from patient**

Yes

**Disclosure of Interest**

None Declared

#### P2077 Kawakinra: a phase IIA multicenter trial to assess the efficacy, and safety of anakinra in patients with intravenous immunoglobulin-resistant Kawasaki disease

##### Isabelle Koné-Paut^1^, Stephanie Tellier^2^, Karine Brochard^2^, Virginie Lambert^3^, Corinne Guitton^1^, Alexandre Belot^4^, Perrine Dusser^1^, Linda Rossi-Semerano^1^, Isabelle Marie^1^, Gregory Allain^5^, Helene Agostini^5^, Celine Piedvache^5^

###### ^1^Pediatric Rheumatology, APHP, Bicetre Hospital, Le Kremlin Bicêtre; ^2^Pediatrics, CHU Purpan, Toulouse; ^3^Pediatric Cardiology, Montsouris Institute, Paris; ^4^Pediatric Rheumatology, CHU Lyon, Lyon; ^5^Clinical Research Unit, APHP, Bicetre Hospital, Le Kremlin Bicêtre, France

####### **Correspondence:** Isabelle Koné-Paut

**Introduction:** The development of more potent therapeutic approaches of KD is an urgent need because intravenous immunoglobulin (IVIg) treatment is not effective in 20% of patients, increasing the risk of coronary dilatations/ aneurysms. The combination of genetic and transcriptomic data revealed the key role of interleukin-1 (IL-1) signaling in KD vasculitis and mouse model of KD has shown that anakinra (IL1RA: IL-1R1receptor antagonist) could prevent the development of vascular aneurysms.

**Objectives:** To assess as primary objective, the efficacy of anakinra in patients with KD who fail to respond to at least one infusion of 2g/kg of IVIg

To assess as secondary objectives, its safety and tolerability and its effect on disease activity, systemic inflammation and coronary lesions.

**Methods:** A 45-day, phase IIa proof of concept study open labeled with anakinra dose escalation, with a target of at least 12 patients completing the study Eligible patients had KD according to the AHA criteria, duration of fever ≤ 14 days, and were ≥ 3 months and 5 Kg. They had persistent (or relapsing) fever (≥38°C) within 48h of the last IVIG infusion, had not received other alternative treatment including steroids, and had no others exclusion criteria. After informed consent, they received a starting dose of 2mg/kg *(patients <10kg and/or <8 months: 4mg/kg)* of anakinra, which could be increased every 24h of 2mg/kg until a maximum of 6mg/kg *(patients <10kg and/or <8 months: 8mg/kg)*, in case of persistent fever. Anakinra treatment duration was 15 days. Outcome measures were fever, KD symptoms, blood inflammation and cardiac echography. Total study duration was 45 days. Clinical trials: NCT02390596. The study is supported by a grant from the French ministry of health, APHP, national PHRC 2014. IRB approval was obtained and all patients (parents) gave informed consent.

**Results:** 18 patients were screened and 16 were included and 13 have completed the study. Anakinra was started in 16 patients (14 boys, 2 girls) at a median age 2 years (3 months to 6 years) and at a median of 9.5 days after the onset of fever. 4 patients escaped early for SAE, and 1 had sJIA final diagnosis. The maximum dose of anakinra was 6mg/kg in 6 patients, 4mg/kg in 6, and 2mg/kg in 4. Mean PGA decreased from 7.80 (4-10) to 1.2 (0-3) at D14. Median temperature was 37.6°C (36.7-39.7) at day 3 and 37.2°C at D7 (36.7- 37.9)). Median CRP was 135mg/L at screening and decreased to 9.5 mg/L at D7. 8/14 evaluated patients had coronary dilatation (Z score max ≥2.5mm) at inclusion, 5/14 at D14 and 3/14 at D45. 3/14 patients who increased Z score at D14 decreased it at J45. We observed 3 severe adverse events (SAE) where treatment was discontinued: anakinra overdose, MAS in a patient evolving to sJIA and increase of coronary dilatation. Others AE included cytolytic hepatitis (2 patients), hypereosinophilia (1), injection site reaction (1) and pancreatitis (1) without treatment discontinuation.

**Conclusion:** We have realized the first experimental trial assessing IL-1 blockade in severe refractory KD. 15 day-duration of anakinra treatment, given early in the course of IVIG-resistant KD, was rapidly effective on KD symptoms, biologic inflammation and coronary dilatations in almost all patients, with a good tolerability. This study calls for further investigation of IL1 blockade in KD.

**Disclosure of Interest**

I. Koné-Paut Grant / Research Support from: SOBI,Consultant for: SOBI, S. Tellier: None Declared, K. Brochard: None Declared, V. Lambert: None Declared, C. Guitton: None Declared, A. Belot: None Declared, P. Dusser: None Declared, L. Rossi-Semerano: None Declared, I. Marie: None Declared, G. Allain: None Declared, H. Agostini: None Declared, C. Piedvache: None Declared

#### P2078 Continuous effectiveness of canakinumab treatment in Schnitzler’s syndrome: a 4-year open-label multi-center study

##### Karoline Krause^1^, Hanna Bonnekoh^1^, André Ellrich^1^, Athanasios Tsianakas^2^, Nicola Wagner^3^, Jörg Fischer^4^, Marcus Maurer^1^

###### ^1^Charite - Universitaetsmedizin Berlin, Berlin; ^2^Universitätsklinikum Münster, Münster; ^3^Klinikum Erlangen, Erlangen; ^4^Universitätsklinik Tübingen, Tübingen, Germany

####### **Correspondence:** Karoline Krause

**Introduction:** Schnitzler’s syndrome (SchS) is a prototype adult-onset autoinflammatory disease characterized by urticarial exanthema and monoclonal gammopathy combined with systemic inflammatory symptoms. We have previously reported the efficacy of canakinumab treatment in a 4-month randomized placebo-controlled multi-center study involving 20 patients with SchS.

**Methods:** All patients who successfully completed the initial 4-month trial were eligible to enter an open-label study extension for a maximum period of 4 years. Patients received canakinumab 150 mg or 300 mg injections as needed based on individual disease activity and response to treatment within the first 4 months. Efficacy was monitored by changes in disease activity (physician global assessment [PGA; range 0-20]), inflammation markers (C-reactive protein [CRP], serum amyloid A [SAA]) and quality of life (DLQI, SF-36). Safety assessment included adverse event reporting and routine clinical and laboratory assessments.

**Results:** A total number of 17 patients entered the open-label extension of the study. Of these, 15 patients completed the whole study period of 4 years.

During this study, continuous effectiveness of canakinumab treatment was demonstrated by significantly lower PGA scores as compared to baseline scores throughout the study. The clinical response was mirrored by normalized inflammation markers CRP and SAA in most patients and stabilized quality of life as determined by DLQI and SF-36. Relapse of symptoms was rare and occurred after canakinumab interruption or infections.

During the open-label extension of the study, seven serious adverse events (SAEs) were noted. These included infections such as pneumonia and sepsis in two elderly patients, one of them with fatal outcome. Also, one patient developed plasmocytoma and left the study.

**Conclusion:** In this long-term open-label study, canakinumab treatment provided continuous symptom control in most patients. Canakinumab may be considered a therapeutic option in these patients but requires close monitoring for potential infections.

**Disclosure of Interest**

K. Krause Grant / Research Support from: Novartis, Roche,Speaker Bureau of: Novartis, Roche, SOBI, H. Bonnekoh: None Declared, A. Ellrich: None Declared, A. Tsianakas: None Declared, N. Wagner: None Declared, J. Fischer: None Declared, M. Maurer: None Declared

#### P2079 Long-term effectiveness of canakinumab in patients with monogenic periodic fever syndromes – first interim analysis of the CAPS subgroup

##### Jasmin B. Kuemmerle-Deschner^1^, Norbert Blank^2^, Michael Borte^3^, Ivan Foeldvari ^4^, Gerd Horneff^5^, Prasad Oommen^6^, Catharina Schuetz^7^, Frank Weller-Heinemann^8^, Julia Weber-Arden^9^, Tilmann Kallinich^10^

###### ^1^Pediatrics, University Hospital Tuebingen, Tuebingen; ^2^Rheumatology, University Hospital Heidelberg, Heidelberg; ^3^ImmnoDeficiencyCenter, Hospital St. Georg, Leipzig; ^4^Center for Pediatric and Adolescence Rheumatology, Hamburg; ^5^Asklepios Clinic, Sankt Augustin; ^6^Pediatrics, University Hospital Duesseldorf, Duesseldorf; ^7^Pediatric Immunology, University Hospital Carl Gustav Carus, Dresden; ^8^Pediatric Rheumatology, Prof. Hess Kinderklink, Bremen; ^9^Novartis Pharma GmbH, Nuremberg; ^10^Pediatrics, Charité University Hospital, Berlin, Germany

####### **Correspondence:** Jasmin B. Kuemmerle-Deschner

**Introduction:** Treatment options for autoinflammatory diseases include anti-interleukin (IL)-1 therapies and IL1 inhibitors since the IL-1 pathway is crucial in immune dysregulation in patients with monogenic periodic fever syndromes.

**Objectives:** The aim is to gain further insights from routine clinical practice with respect to long-term effectiveness and safety of canakinumab (CAN) in pediatric and adult patients with CAPS (cryopyrin-associated periodic syndrome, including Muckle-Wells syndrome [MWS], familial cold autoinflammatory syndrome [FCAS], neonatal onset multisystem inflammatory disease [NOMID]/chronic infantile neurological cutaneous and articular syndrome [CINCA]), FMF (familial Mediterranean fever**)**, TRAPS (tumor necrosis factor receptor-associated periodic syndrome) and HIDS/MKD (hyperimmunoglobulinemia D syndrome/mevalonate kinase deficiency).

**Methods:** RELIANCE is a prospective, non-interventional, multi-center, observationalstudy based in Germany with a 3-year follow-up enrolling pediatric (age ≥2 years) and adult patients with clinically confirmed diagnoses of CAPS, FMF, TRAPS and HIDS/MKD who routinely received CAN. 6-monthly clinical assessment and evaluation of patient-reported outcomes is aligned with routine clinic visits. The endpoints will assess the effectiveness and safety of CAN under standard clinical practice conditions.

**Results:** The interim analysis includes 52 CAPS patients with prior long-term CAN treatment (43.1% females) enrolled by September 2018. 44.2% of the patients participated in the β-Confident studypreviously. The mean age was 20.7 years (4.0–79.0 years) at baseline and the mean duration of prior CAN treatment was 5.4 years (0.0–11.0 years). 40 patients (76.9%) were diagnosed with MWS, the other patients had FCAS (2), NOMID/CINCA (7) or atypical CAPS (1). CAPS disease activity was determined by physicians’ and patients’ assessment of disease activity at baseline and 6 months (table 1) demonstrating sustained remission in patients receiving long-term CAN treatment. Serious adverse events were reported for 2 patients including a case of tonsillitis and a preterm birth at week 31.

**Conclusion:** TheRELIANCE study longitudinally monitors the effectiveness of CAN in patients with monogenic periodic fever syndromes. An initial interim analysis including the CAPS subgroup which had prior CAN treatment showed that CAN is an effective and safe treatment in those patients.

**Disclosure of Interest**

J. Kuemmerle-Deschner Grant / Research Support from: Novartis, SOBI,Consultant for: Novartis, SOBI, N. Blank Grant / Research Support from: Novartis, SOBI,Consultant for: Novartis, SOBI, M. Borte Grant / Research Support from: Novartis, Pfizer, Shire, I. Foeldvari Grant / Research Support from: Novartis,Consultant for: Novartis, G. Horneff Grant / Research Support from: Abbvie, Chugai, Roche, Pfizer, MSD, Novartis,Speaker Bureau of: Chugai, Novartis, BMS, P. Oommen Grant / Research Support from: Novartis, C. Schuetz Grant / Research Support from: Novartis, F. Weller-Heinemann Grant / Research Support from: Novartis, J. Weber-Arden Employee of: Novartis, T. Kallinich Grant / Research Support from: Novartis

**Table 1 (abstract P2079).** Disease activity by physicians’ and patients’ assessment

	Baseline, all patients, N=52	Baseline, analysis cohort, N=31	Month 6, analysis cohort, N=31
Disease activity by physicians’ assessment: absent, mild/moderate, severe, % of patients			
Urticarial skin rash	71.4, 20.4,4.1	67.7, 19.4, 6.5	74.2, 25.8, 0.0
Arthralgia	67.3, 30.6, 2.0	67.7, 32.3, 0.0	61.3, 29.0, 9.7
Myalgia	91.8, 6.1, 0.0	87.1, 9.7, 0.0	83.9, 16.1, 0.0
Conjunctivitis	71.4, 26.5, 2.0	64.5, 32.3, 3.2	77.4, 12.9, 6.5
Headaches	69.4, 22.4, 6.1	64.5, 22.6, 9.7	61.3, 19.4, 19.4
Abdominal pain	81.6, 14.3, 4.1	83.9, 9.7, 6.5	67.7, 22.6, 9.7
Patients’ assessment			
Mean disease activity, visual analog scale (VAS) 0–10	2.1	2.0	1.8
Mean Fatigue, VAS 0–10	2.6	2.5	2.7
Impairment of social life, %	52.6	54.5	24.0

#### P2080 The effectiveness of anti-TNF therapy in association of familial Mediterranean fever and ankylosing spondylitis

##### Sevgi Atar, Baris Colak, Omer Kuru

###### Departmant of Physical Medicine and Rehabilitation Division of Rheumatology, University of Health Sciences, Istanbul Okmeydani Research and Training Hospital , Istanbul, Turkey

####### **Correspondence:** Omer Kuru

**Introduction:**

Familial Mediterranean Fever (FMF) among most of Mediterranean Mediterranean peoples, especially it’s a disease that is seen between Turkish, Arab, Spanish Jews and Armenians, with increased acute phase reactants, acute inflammation of serous membranes such as recurrent peritoneum, pleural and synovium, fever and self-limiting attacks. It is an otosomal recessive disease. The interval between attacks is unclear, and it is difficult to predict the attacks.

As with different attacks, different symptoms may be seen in different patients. The disease occurs for the first time in childhood or young adulthood. The onset of the disease after the fourth decade is very rare. Although some studies have reported that men are more frequent (M / F: 1.5-2 / 1) by the Turkish FMF study group showed that similar rates in both sexes (M / F: 1,2 / 1).

Ankylosing spondylitis (AS) is a chronic rheumatic disease primarily affects axial skeleton, characterised by inflammatory back pain and stiffness in the morning. AS is associated with FMF was related to gene mutations. It is also thought to increase the prevalence of ankylosing spondylitis or spondyloarthritis in FMF patients.

**Objectives:**

In this study, we aimed to discuss the improvement in clinical and laboratory findings of anti-TNF treatment in the perspective of literature with the association of FMF and AS, prolonged fever and serositis attacks in spite of high doses of colchicine.

**Methods:**

Case:

46-year-old female patient had abdominal pain, diarrhea, nausea and vomiting for 5 days in last 2 years. She had not fever attacks, concomitant joint pains and low back pain. Colchicum dispert used 1.5 mg / day. There was no alcohol and no smoking. Her past medical history revealed that she had familial acute fever and her sister passed on hypertension and thyroid disease. Except for erythrocyte sedimentation rate and high C-reactive protein levels, no additional features were detected in laboratory tests. Anti-TNF (etanercept) treatment was started.

**Results:**

Patient had no episode during 1st month and 3rd month control. Erythrocyte sedimentation rate and C-reactive protein values ​​were found to be regressed.

**Conclusion:**

In our case, we achieved clinical improvement with anti-TNF in acute attacks of FMF. We believe that this treatment efficacy should be investigated with larger prospective studies.

**Key words:** anti-TNF therapy, familial mediterranean fever

**Consent for publication has been obtained from patient**

Yes

**Disclosure of Interest**

None Declared

#### P2081 Desensitization to anakinra in refractory recurrent pericarditis

##### C. Longo^1^, S. Signa^1^, M. D'Alessandro^1^, M. Bustaffa^1^, R. Consolini^2^, M. Tosca^3^, L.O. Mendonça^4^, A. Ravelli^1,5^, R. Caorsi^5^, M. Gattorno^5^

###### ^1^Department of Pediatrics, Università degli studi di Genova, Genoa; ^2^Department of Pediatrics, Università degli studi di Pisa, Pisa; ^3^U.O. Pneumologia, G. Gaslini Institute, Genoa, Italy; ^4^Clinical Immunology and Allergy Department, HC-FMUSP, Sao Paulo, Brazil; ^5^Clinica Pediatrica e Reumatologia, G. Gaslini Institute, Genoa, Italy

####### **Correspondence:** C. Longo

**Introduction:** Recurrent pericarditis (RP) is a possible complication of acute pericarditis (15–30%). It can be idiopathic or it can occur after a pericardial procedure (post-pericardiotomic). First line treatment consists of a combination of high-doses of NSAIDs with colchicine; corticosteroids represent the second line in resistant or intolerant cases. Different biologics and immunosoppressant have been used as third line treatment, with variable responses: by now the most promising results have been obtained with anakinra, enlightening the possible role of IL-1 in the pathogenesis of this condition.

**Objectives:** To describe the clinical course and the outcome of desensitization procedure to anakinra in a patient with steroid-dependent pericarditis, who withdrawn anakinra for adverse reactions and did not respond to IL-1β blockade with canakinumab.

**Methods:** A 9-years old girl started to complain with recurrent pericarditis at the age of 6 years old, after surgical correction of an atrial septal defect. NSAIDs were not effective and colchicine was withdrawn for intolerance; oral steroids were started with good response, then gradually tapered, with prompt relapse after the steroid discontinuation, requiring pericardiocentesis. The child showed a steroid-dependent course of the disease, with several relapses after tapering attempts. In March 2016, after 5 relapses, anakinra (2 mg/kg/day) was started with a fast and complete clinical response; however, it was discontinued after 2 weeks for the appearance of severe local side effects. She was firstly evaluated in our Center in April 2016; in July 2016 therapy with canakinumab 150 mg (4 mg/kg) every 4 weeks was started. She experienced four relapses during this treatment (July 2016 -December 2017), following every attempt to reduce steroidal dosage, despite the modification of the schedule (4 mg/kg every three weeks) and the reintroduction of colchicine. Due to inadequate response, canakinumab was withdrawn and, in light of the effectiveness previously demonstrated by anakinra, it was decided, after an allergologic consultation, to attempt to reintroduce this therapy, performing a process of desensitization as reported by Mendonca et al (J clin immunology, 2017)**.**

**Results:** In January 2018 desensitization to anakinra was started. The patient received five to three consecutive injections per day of gradually increasing anakinra doses and dilutions from days 1 to 9. Each injection was spaced by 15 minutes intervals, raising the dose at each step. On Day 2, due to the appearance of skin reactions at injection site, it was decided to enlarge the interval between the injections (30 minutes) and increase the dilution, restarting desensitization protocol. The full target dosage (80 mg/day; 2mg/kg/day) at standard dilution (divided in 4 different administrations) was reached on Day 8. Since Day 11 anakinra was administrated twice a day, once a day after one month. Antihistaminic and steroids were administrated during all the desensitization process, then discontinued, without recurrence of both skin reactions and disease manifestations. In June 2018 low-dose colchicine was progressively tapered and finally discontinued.

**Conclusion:** Desensitization process to anakinra allowed to achieve full control of the disease in a patient with severe refractory recurrent pericarditis, not responding neither to first line treatment nor to IL-1β blockade and becoming steroids-dependant. Even further data are required, the divergent response to the two antagonists of IL-1 could suggest a prominent role of IL-1α in the pathogenesis of this disease.

**Consent for publication has been obtained from patient**

Yes

**Disclosure of Interest**

None Declared

#### P2082 One decade real life experience with anti-IL1 therapy in monogenic and multifactorial autoinflammatory diseases

##### Leonardo O. Mendonca, Andressa Guariento, Roberta Caorsi, Sara Signa, Ilaria Gueli, Angelo Ravelli, Marco Gattorno

###### Pediatric Rheumatology Clinic, Istituto Giannina Gaslini, Genoa, Italy

####### **Correspondence:** Leonardo O. Mendonca

**Introduction:** The immunological concept of autoinflammation made possible genetic dissection of inflammation, development of new cytokine target therapies and catalogue the a.k.a. Autoinflammatory Diseases (AID). However, in 50% or more, no causative gene can be found going down to Undefined Inflammatory Syndromes (UIS). The identification of the pathogenic mechanisms related to most of the monogenic and multifactorial auto inflammatory diseases prompted the evidence of IL-1 as a crucial target for treatment.Moreover,the success of these treatments in the most frequent autoinflammatory diseases, suggested their off-label use in many other rare conditions, including in the undifferentiated AID.

**Objectives:** This paper aims to retrospectively report 10 years experience with different IL-1 blocking agents in monogenic and non monogenic disorders in a single, pediatric reference center in Italy.

**Methods:** All patients followed by the Pediatrics and Rheumatology Unit ogf Gaslini Institutewho start an anti-IL1 treatment , from 2005 to 2018 were retrospectively reviewed. Patient’s descriptive characteristics were reported as mean, standard deviation, minimal and maximum values. The variables associated with response in the univariate analysis (p<0.20) were included in the multivariate model.

**Results:** From 2005, 108 consecutive patients receive at least one anti-IL-1 treatment, namely Anakinra or Canakinumab. Among monogenic AID (60,1%), 18% were CAPS, 16% TRAPS, 13% Hyper IgD/MKD, 8% PAPA, 1% DADA2. Anti-IL1 was also used in many multifactorial conditions, mainly SJIA (n=51), undefined SAID (n=18), and recurrent recurent idiopathic pericarditis (n=6) and one patient with SAPHO syndrome. Among the monogenic forms, CAPS, TRAPS and Mevalonate Kinase Deficiency, that started on anakinra (n=19) 73,7% (n=14) achieved a complete or partial control of their condition. Switching to canakinumab (n=12) or starting direct with canakinumab (n=9) resulted in 85% of disease control. UIS presented high rate of response to anakinra, 75% versus 52% of SJIA. Biologic drug could be discontinued in 6 patients after clinical remission. Just one patient, with severe phenotype of MVK did not achieve clinical control with anakinra and was successfully submitted to HSCT. In the univariate model, using p value <0,20 , statically difference could be obtained with the following characteristics: symptoms started<1 year, presence of chest pain, macrophage activation syndrome, pericarditis, periodic fever, poliartrhitis and psoriasis. Using the multivariate model, just periodic fever remained as a positive predictor of response to anti-IL1 and the presence of poliartrhitis remained as a negative predictor.

**Conclusion:** Different anti-IL1 strategies can be used to disease control for the monogenic forms of AID as well as for undefined AID.

**Disclosure of Interest**

None Declared

#### P2083 A prospective outcome of all 17 consecutive pediatric patients with chronic nonbacterial osteomyelitis treated with intravenous pamidronate and followed at a single center over 15 years

##### Paivi Miettunen^1^, Chloe M. Stephenson^1^, Seamus L. Stephenson^1^, XIng-Chang Wei^2^

###### ^1^Pediatrics; ^2^Radiology, University of Calgary, Calgary, Canada

####### **Correspondence:** Paivi Miettunen

**Introduction:**Intravenous pamidronate ( IV-PAM) has been reported to be effective in pediatric patients with severe chronic nonbacterial osteomyelitis (CNO)in short term. Little is known about longterm outcome in CNO after IV-PAM.

**Objectives:** To describe a consecutive series of pediatric CNO (pCNO) patients who were prospectively followed after treatment with IV-PAM between 2003-2018 at a single center regarding: 1) The effect of IV-PAM on pain and Whole Body Magnetic Resonance Imaging (WBMRI) documented inflammation initially and after flare; 2) Relapse rate and; 3) Spinal CNO outcome and4) Urine N-telopeptide/creatinine ratio (UNtx/Cr, a product of collagen 1 breakdown) correlation with active CNO.

**Methods: Patients:** All pCNOpatients (age at diagnosis <18 years) with WBMRI confirmedactive disease who required at least one dose of IV-PAM between 2003-2018 were included.

**IV-PAM dosing:** 0.5 mg/kg (maximum 30 mg) for the first dose and 1 mg/kg (maximum 60 mg) for all subsequent doses (maximum annual dose 11.5 mg/kg). The initial IV-PAM was administered once monthly x 9; with potential flares IV-PAM was given until resolution of pain and/or resolution/stabilization of CNO lesions on imaging (max 9 doses/year).

**Pain response:** Visual analogue scale for pain (VAS) with “0” indicating no pain and “10” maximum pain was administered at baseline, at each IV-PAM treatment, and at suspected flare.

**Imaging:**WBMRI was administered before 1^st^ IV-PAM, at 6 and 12 months, at suspected flare and after retreatment. CNO resolution was documented as complete (CR) if there was 95% improvement in the WBMRI signal, as partial resolution (PR < 95 % improvement), or as no response. Spinal x-rays were performed at baseline and then yearly.

**UNtx/Cr** was measured at baseline, after each IV-PAM and with suspected flare.

**Results:** 17 patients (9F, 8M) were included. The median [range] age at CNO diagnosis was 10.3[4-15] years, and at first IV-PAM 11.6[4-20] years. The median [range] follow-up was 9.2[1-15] years. Six patients had unifocal CNO, (n=1 femur, 1 radius, 3 mandible, 1 clavicle), 4 had spinal CNO (3 with extraspinal sites, 2 with baseline vertebral fractures) and remaining 6 had multifocal non-spinal CNO. VAS was uniformly 10/10 at baseline and decreased to 0-1/10 within first month after IV-PAM. CR was achieved by all at 6 months, which persisted at 12 months. Four patients, 3 unifocal and one multifocal, had no further flares. Twelve patients had WBMRI confirmed flare at previously active sites at 9-36 months and 11 received 1-9 further doses of IV-PAM. With flare, VAS ranged from 4-9/10 and decreased to 0-3/10 within first month after re-initiation of IV-PAM.On final WBMRI, 12/17 (70%) had CR and 5/17 (30%) stable mild increased signal but no clinical symptoms. No further spinal compression fractures occurred. One patient required a third course of IV-PAM. Regarding UNtx/Cr, each patient hadappropriate reduction after first IV-PAM. No patients flared whileUNtx/Cr remained suppressed. Three patients developed arthritis and one acne. At last follow-up, 10/17 (59%) patients were on no medications, 4/17 (24%) required prn Naproxen for CNO and 3/17 (18%) were on Naproxen and/or disease modifying medications for arthritis.

**Conclusion:**Long-term follow-up ofpCNO patients treated with IV-PAM confirms that while severalpatients eventually flare, the flares are less painful and have an excellent response to IV-PAM retreatment. No further spinal fractures occurred . No flares occurred while UNtx/Cr remained suppressed, suggesting a role of osteoclasts in CNO. Further prospective multicenter studies are now required to define the long-term clinical and imaging response to IV-PAM.

**Disclosure of Interest**

None Declared

#### P2084 Efficacy of anakinra in the treatment of recurrent idiopathic pericarditis in pediatric population: experience in a tertiary hospital

##### Sara Murias^1^, Luis García-Guereta^2^, Rosa Alcobendas^1^, Diana Salas^2^, Clara Udaondo^1^, Pablo Fernandez^1^, Catarina Fervenza^1^, Agustin Remesal^1^

###### ^1^Pediatric Rheumatology; ^2^Pediatric Cardiology, University Hospital La Paz, Madrid, Spain

####### **Correspondence:** Sara Murias

**Introduction:** Although there is growing evidence on the efficacy of anakinra in the treatment of recurrent pericarditis, its use in pediatric patients is still very limited.

**Objectives:** Our aim is to review the experience of our center in the use of this therapeutic option in children with recurrent pericarditis who did not respond to classical therapy.

**Methods:** Descriptive study of a series of cases by collecting data through the clinical history.

**Results:** 7 patients were included (6 girls, 1 boy). All of them presented recurrent symptomatic pericarditis with poor outcome despite several treatments (ibuprofene, colchicine, steroids).

All had a quick response to treatment with complete resolution of systemic and cardiologic symptoms, associated to normalization of laboratory parameters thus allowing steroids withdrawal.

Two out of the three patients in whom anakinra was eventually discontinued required reintroduction due to recurrences. One remains asymptomatic after two years without treatment.

Six patients are still on anakinra (variable dosage), with absence of flares and with no significant side effects.

**Conclusion:** Anakinra is very useful for the treatment of recurrent pericarditis in pediatric patients. The good response observed through this sample supports the autoinflammatory etiopathogenesis of some cases of recurrent pericarditis.

The duration of treatment needs to be prolonged, and its withdrawal may involve flares that quickly respond to ANK reintroduction.

**Disclosure of Interest**

None Declared

**Table 1 (abstract P2084).** Clinical characteristics of 6 girls and 1 boy with recurrent pericarditis and complete response to anakinra

	Pacient 1	Pacient 2	Pacient 3	Pacient 4	Pacient 5	Pacient 6	Pacient 7
Age at onset (years)	9	11	3	12	15	17	1.2
Time from onset to first ANK use	9	24	12	24	2	2	6
Former therapies	Ibuprofene Colchicine Prednisone	Ibuprofene Dexametasone Colchicine	Ibuprofene Prednisone Colchicine	Ibuprofene Prednisone Colchicine	Ibuprofene Prednisone Colchicine	Ibuprofene Prednisone Colchicine	Indometacine Colchicine Prednisolone
Symptoms associated to pericarditis*	Fever Asthenia Pleuritis Peritonitis	Fever Asthenia Skin rash Pleuritis	Fever Abdominal pain	Pachypleuritis	Fever Asthenia	Febricula Pleuritis	Hepatomegaly
CRP (mg/L)*	104	250	162	350	56	150	16
Related disease	No	SoJIA and MAS	No	FMF (mutations M694l and R202Q)	No	Renal trasplantation due to vasculitis	No
CRP (mg/L)**	0.54	0.8	1.63	0.59	2.43	48	Not done yet
Number offlares after starting ANK	0	1 flare, 30 days after ANK withdrawal	0	1 flare, 35 days after voluntarily stopping ANK	0	0	0
Current treatment	None (ANK withdrawn: september 2015)	ANK 100 mg /day	ANK 75 mg every 72 hours	ANK 100 mg/day	ANK 75 mg every 72 hours	ANK 100 mg/day	ANK 50 mg/day

*Maximum values during a typical flare

**First laboratory examinations performed after starting ANK

(ANK: Anakinra. CRP: C-Reactive Protein. SoJIA: Systemic onset Juvenile Idiopathic Arthritis. MAS: Macrophage Activation Syndrome. FMF: Familial Mediterranean Fever)

#### P2085 Clinical manifestations and treatment of idiopathic recurrent aseptic meningitis (IRAM)

##### Michele Nehrebecky^1^, David Beck^1^, Amanda Ombrello^1^, Ariane Soldatos^2^, Bryan Smith^2^, Patrycja M Hoffman^1^, Lauren Reoma^2^, Avindra Nath^2^, Karyl S. Barron^3^, Anne Jones^1^, Ivona Aksentijevich^1^, William A. Gahl^1^, Camilo Toro^1^, Daniel L. Kastner^1^

###### ^1^NIH/NHGRI; ^2^NIH/NINDS; ^3^NIH/NIAID, Bethesda, United States

####### **Correspondence:** Michele Nehrebecky

**Introduction:** Idiopathic Recurrent Aseptic Meningitis (IRAM) is an autoinflammatory syndrome characterized by non-infectious episodic fevers, malaise and meningeal inflammation associated with cerebral spinal fluid (CSF) pleocytosis, punctuated by symptom-free intervals.

**Objectives:** We aim to characterize clinical, imaging, laboratory and molecular features of a cohort of patients with IRAM, learn about pathophysiological mechanisms and share our experience with anakinra as a therapeutic option.

**Methods:** A single center cohort of 12 patients with features of IRAM were evaluated from the National Institutes of Health’s (NIH) Clinical Center in Bethesda, Maryland. Ages ranged from 12-74 years (5 females, 7 males) with majority adult onset.Duration of symptoms prior to NIH evaluation ranged from 4 months to 29 years. The most common clinical features were recurrent periodic fevers, fatigue, headache, decreased appetite, sensorineural hearing loss (SNHL), CSF pleocytosis, elevated inflammatory markers and resolution of symptoms between episodes. Frequency of episodes varied from 1 to approximately 50 episodes per year.A multidisciplinary evaluation including infectious disease studies, whole exome sequencing (WES), cranial magnetic resonance imaging, lumbar puncture and audiologic assessment were performed to further characterize the phenotype and pathophysiology of the disorder.Symptomatic treatment with the IL-1 receptor antagonist anakinra at a starting daily dose of 100mg subcutaneously was attempted in six patients.

**Results:** Whole exome sequencing was performed in all 12 subjects.Diagnoses were confirmed in 2/12 patients (1 with a pathogenic germline *NLRP3* mutation and one subsequently found to have CNS cysticercosis via detailed infectious studies)

Cranial MRI imaging revealed meningeal enhancement in 2 of the 10 subjects.5 of the 10 subjects had elevated ESR and CRP.Lumbar punctures (performed during acute episodes in 10) revealed evidence of CSF lymphocytic pleocytosis. PCR for herpes family viruses was negative in all.Audiology assessment was performed on 6 of the 10 subjects and revealed SNHL in all evaluated.

For the six patients who received anakinra, dose escalation was required for three up to 500mg per day.Clinical improvement as defined by reduced frequency and severity of episodes was reported by four of the six subjects.

**Conclusion:** Patients with IRAM may represent a molecularly heterogeneous group of patients with episodic systemic and intrathecal innate immune system activation including patients with germline *NLRP3* mutation.Preliminary observations on the response to anakinra supports a role of innate immune activation in this disorder and a potential role of IL-1 inhibition of the management of this condition.

**Disclosure of Interest**

None Declared

#### P2086 Autoinflammatory syndrome caused by mutations in TRNT1: successful treatment of two cases with etanercept

##### Francesca Orlando^1,2^, Roberta Naddei^2^, Carlo Maria Gallinoro^2^, Daniela Melis^2^, Maria Alessio^2^

###### ^1^Department of Pediatrics, Santobono-Pausilipon Children’s Hospital; ^2^Department of Mother and Child, University of Naples Federico II, Naples, Italy

####### **Correspondence:** Francesca Orlando

**Introduction:** *TRNT1* is a nuclear gene encoding a ubiquitous enzyme (CCA-adding tRNA nucleotidyl transferase enzyme) necessary for aminoacylation of both mitochondrial and cytosolic tRNA. *TRNT1* mutations are associated to heterogeneous phenotypes and systemic involvement of variable severity and progression.

**Objectives:** To characterise clinical features, laboratory assessment and treatment strategies of two patients affected by an autoinflammatory syndrome caused by *TRNT1* mutations.

**Methods:** The patient 1 (P1) is a twenty years old female. From the first month of life she experienced recurrent fever associated to painful cutaneous lesions, edema of the hands, raised inflammatory markers and anemia. The symptoms were moderately controlled by corticosteroid therapy. One of these episodes was characterized by severe anemia (Hb 6.6 g/dl), requiring blood transfusion. At the age of 8 months she was found to have mild hypogammaglobulinemia, but no other alterations were found on immunological investigations. She showed normalization of the immunoglobulin levels at the age of 22 months. The physical examination found facial dysmorphisms, brittle hair, developmental delay and microcephaly. Splenomegaly was reported by abdomen ultrasound. Isolated Growth Hormone Deficiency (GHD) was diagnosticated at the age of 3 years and it was treated with recombinant human GH (rhGH) for the next 4 years, without improvement. At the age of 8 years she was diagnosed with bilateral sensorineural hearing loss.At the age of 10 she was diagnosticated with posterior subcapsular cataract, probably related to chronic steroid treatment. The patient 2 (P2) is a ten years old female. From four months of age she suffered from recurrent fever, associated from the age of 11 months to painful cutaneous lesions. Laboratory assessment showed elevated serum inflammatory markers, mild anaemia, hypogammaglobulinemia and reduced levels of B-cells. Growing up, she showed failure to thrive, developmental delay, facial dysmorphisms, brittle, microcephaly, hepato-splenomegaly and swelling of the right ankle. At the age of two she was diagnosticated with sensorineural hearing loss and bilateral cataract. At the age of 5 she started therapy with rhGH for GHD.

**Results:** Suspecting Chronic Infantile Neurologic Cutaneous and Articular (CINCA) syndrome, even though negativity of molecular analysis of CIAS1, they started treatment with anti-IL1 (Anakinra) at the age of 9 (P1) and 2 (P2). Due to the partial response, P1 shifted to anti-TNFa therapy (Etanercept) at the age of 11 with significant improvement. P2 showed nonresponse to Anakinra so she shifted to Etanercept after four months. Whole exome sequencing was performed resulting in two mutation of TNRT1: P1 c.608G>A (p.Arg203Lys), c.1246A>G (p.Lys416Glu); P2 c.938delT (p.Leu313fs), c.1246A>G (p.Lys416Glu).During the 9 years follow-up the patients underwent six-month clinical and laboratory assessment, showing normalization of inflammatory index and resolution of recurrent fever and associated symptoms.

**Conclusion:** To date, 34 patients with *TRNT1* mutations related disease have been reported, presenting significant clinical heterogeneity. In this study we extend the cohort of patients and we report a new mutation (c.938delT).It was recently reported that treatment with TNF inhibitors has been efficacious in four patients, including three patients treated with Etanercept for 3 years.We report the longest follow-up in these patients treated with Etanercept, confirming efficacy and safety of that treatment.

**Consent for publication has been obtained from patient**

Yes

**Disclosure of Interest**

None Declared

#### P2087 Does colchicine really cause leukopenia?

##### Erdal Sag^1^, Yagmur Bayindir^2^, Aydin Adiguzel^2^, Selcan Demir^1^, Yelda Bilginer^1^, Selin Aytac^3^, Seza Ozen^1^

###### ^1^Division of Pediatric Rheumatology; ^2^Department of Pediatrics, Faculty of Medicine, Hacettepe University, Ankara, Turkey; ^3^Division of Pediatric Hematology, Department of Pediatrics, Faculty of Medicine, Hacettepe University, Ankara, Turkey

####### **Correspondence:** Seza Ozen

**Introduction:** FMF is the most common monogenic autoinflammatory disease and colchicine has been used as the drug of choice for the treatment since 1972. It is a safe drug when it is used in the suggested dosage however it is associated with certain side effects. Some case reports suggest that colchicine can cause leukopenia even with the lower doses.

**Objectives:** In this study, we aimed to investigate the bone marrow side effects of colchicine in patients who are treated with normal routine dose (0.5-2mg/day) for FMF.

**Methods:** 213 consecutive pediatric FMF patients between July-September 2018 who were followed at Hacettepe University Department of Pediatric Rheumatology and treated with colchicine for at least 6 months were included in this study. Clinical characteristics, demographics, and laboratory findings were reviewed retrospectively from medical charts and electronic records of the patients. In patients with leukopenia, the time interval from the treatment onset and time interval to full recovery were also recorded.

Patients were diagnosed as FMF according to Yalcinkaya-Ozen criteria, and the treatment started as 0.5 mg/day <5 years, 1 mg/day 5-10 years and 1.5 mg/day >10 years old. Max colchicine dose was 2 mg/day. Patients were followed at 2^nd^week of treatment for acute side effects of colchicine, at 3 months and then every 6 months. GraphPad 6.0 was used to evaluate the statistical analysis. Proportions, medians, minimum and maximum values were used where appropriate to present the descriptive analyses.

**Results:** 213 pediatric FMF patients were included to the study group of whom 52.3% of them were female. The mean age at the disease onset and the start of the colchicine was 5.74±4.07 years. The median follow-up duration was 70 months ranged from 8 months to 16.9 years.175 (82.2%) of the patients had recurrent fever, 165 (77.5%) of them had abdominal pain, 89 (42%) of them had arthritis and 17 of them (8%) had chest pain at the time of diagnosis.

Twenty-three (10.8%) out of 213 FMF patients had leukopenia during the colchicine treatment. Among these 23 patients, 1 of them had moderate lymphopenia (500-1000/mm^3^), 2 had moderate neutropenia (500-1000/mm^3^), 7 had mild neutropenia (1000-1500/mm^3^) and 2 patients had both mild lymphopenia and neutropenia. The median interval between the onset of the treatment and the leukopenia onset was 63 months (range 3-179 months). At the time of leukopenia onset, median colchicine doses per weight (0.026 vs 0.027 mg/kg/day p:0.96) were comparable in patients with and without leukopenia. There were no other significant differences in terms of demographics, clinical features, mutation types and colchicine formulations. Lymphocyte count (1760.87±512.34 vs 2264.89±924.04; p<0.001) PMNL count (1673.91±574.63 vs 4172.34±3074.77; p<0.001) and platelet count (249304±42568 vs 282399±77571; p<0.001) were also lower in this group as expected.

Colchicine doses were lowered due to this adverse event. In all patients, leukopenia was transient and completely resolved in median 146.0 (IQR 70.5-192) days. There was no increased rate of infections observed in these patients with leukopenia compared to other patients.

**Conclusion:** Colchicine caused leukopenia in 10.8% of the patients in our cohort however only 3 patients had moderate cytopenia and none of them had severe leukopenia. All of the cases were fully reversible after dose adjustment and there were no clinical consequences.Leukocyte counts should be assessed every 6 months in patients on colchicine.

**Disclosure of Interest**

None Declared

#### P2088 Mycophenolate mofetil (MMF) in defined and undefined interferonopathies

##### Carmela Gerarda Luana Raffaele, Gianmarco Moneta, Silvia Federici, Manuela Pardeo, Claudia Bracaglia, Fabrizio De Benedetti, Antonella Insalaco

###### Division of Rheumatology, IRCCS, Ospedale Pediatrico Bambino Gesù, Rome, Italy

####### **Correspondence:** Carmela Gerarda Luana Raffaele

**Introduction:** Type I interferonopathies are genetic disorders characterized by an up-regulation of type I interferon (IFN) activity. An increased expression of type I IFN regulated genes, IFN signature (IS), is described in these conditions. They are characterized by autoinflammation and varying degrees of autoimmunity or immunodeficiency. Some patients with a phenotype strongly suggestive for type I interferonopathy with a high IS, do not show any mutations in known type I interpheronopaties related genes, being classified as undefined

**Objectives:** To evaluate the effect of mycophenolate mofetil (MMF) in patients with defined and undefined type I interferonopathy

**Methods:** 7 patients with type I interferonopathy followed at a Pediatric Rheumatology center, were included. Leucocyte (WBC) and platelet count, hemoglobin (Hb), CRP, ESR, serum amyloid A (SAA), autoantibodies, complement levels and IS, were assessed before and after 5-7 months of MMF treatment (600 mg/m^2^ BID). IS was determined by qPCR (high scores >1.42)

**Results:** Patient 1 and 2 presented respectively with SAVI and Aicardi-Goutieres syndrome. The others were classified as undefined. In patient 1(table), treated with JAK1/2 inhibitor, MMF was followed by decrease of inflammatory markers and resolution of lung involvement; in patient 2 to an increase in Hb and normalization of inflammatory markers. Among patients with an undefined phenotype the addition of MMF to low dose prednisone led to complete resolution of inflammation. A reduction of the antinuclear antibodies titre was observed in patients 2, 3 and 7 and a normalization of complement level in patients 3, 4 and 7. Patient 5 had normal inflammatory markers already before the beginning of MMF possibly due to previously administered immunosuppressive treatment. Despite all patients presented a significant improvement of clinical picture after 5 months of MMF treatment, the IS decreased only in 4/7 patients while it remain stable or increased in the others

**Conclusion:** Our data suggest that mycophenolate mofetil might be an effective therapy in patients with type I interferonopathy leading to improvement of clinical and laboratory features thus allowing a glucocorticoid sparing

**Consent for publication has been obtained from patient**

Yes

**Disclosure of Interest**

None Declared

**Table 1 (abstract P2088).** See text for description

	Pt 1	Pt 2	Pt 3	Pt 4	Pt 5	Pt 6	Pt 7
Disease	SAVI (lung and skin involvement, systemic inflammation)	Aicardi Goutieres (neurological involvement, rash, fever)	Undefined (epilepsy, arthritis,skin involvement)	Undefined (recurrent fever, hypertransaminasemia, hemophagocytic lymphohistiocytosis)	Undefined (hemiplegia)	Undefined (epilepsy, arthritis,skin involvement)	Undefined (recurrent fever, lymphadenopathy, hypogammaglobulinemia
Clinical improvment after MMF	Resolution of lung involvement	Resolution of fever and rash	Resolution of rash, arthritis, improvement of seizures	Resolution of fever, inflammation and hypertransaminasemia	Improvement of motility	Resolution of arthritis and skin involvement	Resolution of fever and limphadenopathy
Gender	F	F	M	M	M	F	M
Age at onset (yrs)	3 (days)	2	4	7	4	6	16
WBC/ mmc^3^*	16 /7,4	8,0/8,4	9,8/8,5	1,7/8,4	4,4/8,2	4,4/5,2	6,6/5,9
Hb g/dl *	7,7/8,9	8,5/12,3	12,9/12,8	9,7/13	12,1/10,7	12/12,9	11,5/15,1
PLT/mmc^3^ *	950 /766	680/320	194/181	56/161	358/421	381/390	218/323
ESR (mm) *	44/40	49/9	38/10	7/6	2/5	24/6	3/3
CRP (mg/dl) *	1,22/0,73	3,13/0,06	5,55/0,32	<0,05/<0,05	<0,05/<0,05	1,15/<0,05	8,34/0,98
SAA (mg/dl)*	95,8/3,62	5,37/2,82	188/8,11	-/1,89	<0,84/-	4,19-0,78	/
C3 (90-180mg/dl)*	154/134	122/108	81/92	83/118	203/170	127/108	53/133
C4 (10-40 mg/dl)*	22/22	10/24	6/8	16/27	38/28	24/14	15/41
Antinuclear antibody*	Absent	1:2560/1:160	1:10.240/1:1280	Absent	Absent	1: 2560 /1:320	Absent
ENA*	pANCA:40/1:20	Absent	Anti dsDNA 1:1280/1: 160	Absent	Absent	Anti dsDNA 1:320/1:40	Absent
IFN signature*	43,2/18,3	42,6/79,8	54,73/64,6	8,66/18,19	40,6/5,95	171,5/13,12	14,34/2,24

* Lab value pre/post MMF therapy

#### P2089 Successful IL-17 targeted therapy in a patient with severe Blau syndrome and novel NOD2 mutation

##### Nikolaus Rieber^1^, Maximilian Steinhauser^1^, Matthias Klopfer^2^, Sabine Pietzsch^3^, Peter Strotmann^1^, Harald Engelhardt^3^, Uta Behrends^1^, Rainer Berendes^3^, Stefan Burdach^1^

###### ^1^Department of Pediatrics, Kinderklinik Muenchen Schwabing, München-Klinik Schwabing und Klinikum rechts der Isar, Technical University of Munich; ^2^Department of Ophthalmology, Technical University of Munich, Munich; ^3^Kinderklinik St. Marien Landshut , Landshut, Germany

####### **Correspondence:** Nikolaus Rieber

**Introduction:** Blau syndrome is a monogenic disease resulting from mutations in the pattern recognition receptor NOD2, and is phenotypically characterized by the classical triad of granulomatous polyarthritis, dermatitis and uveitis. However, extra-triad symptoms have increasingly been reported including, but not limited to, vasculitis, granulomatous glomerular and interstitial nephritis, interstitial lung disease, arterial hypertension, pericarditis, and hepatic granulomata.^1^ Therapy of Blau syndrome is challenging with following medications being reported with varying responses: steroids, methotrexate, TNF-blocker, Il-1 and IL-6 targeted therapies.

**Objectives:** To describe the case of a Blau syndrome patient refractory to conventional therapies benefiting from IL-17 targeted therapy.

**Results:** The female patient of African origin presented at the age of six months with recurrent high fever episodes for days to weeks, polyarthritis, erythematous and ichthyosiform skin manifestations, severe panuveitis, granulomatous liver disease, kidney disease with tubulopathy and severe arterial hypertension. Laboratory evaluation revealed highly elevated inflammation parameters (C-reactive protein (CRP), erythrocyte sedimentation rate (ESR), Serum Amyloid A (SAA)). Liver biopsy showed granulomatous liver disease. Broad infectious disease work-up including tuberculosis was negative. The family history was unremarkable regarding inflammatory or immunological diseases. The suspicion of Blau syndrome was made and genetic analyses revealed a novel *de novo* mutation in the NOD2 Gene (c.[1807C>A]; Histidin_603_> Asparagin) confirming the diagnosis Blau syndrome. The patient was consecutively treated with each high doses of steroids, IL-1 receptor antagonist (anakinra up to 10mg/kg/d) and anti-TNF antibodies (allergic reaction to 3^rd^ dose of infliximab, adalimumab up to 5mg/kg weekly) in addition to maintenance doses of steroids, none of which with sustained success. Therefore a hitherto novel therapeutic approach was initiated with an anti-IL-6 therapy (tocilizumab; up to 20mg/kg every 2 weeks) which yielded a good, yet only contemporary response for up to 6 months. As significant amounts of IL-17 have been identified in granuloma biopsies of Blau syndrome patients² we decided to start a novel therapy targeting the Il-23 / IL-17 pathway. We first used the anti- IL-12 / IL-23 antibody ustekinumab (up to 6mg/kg every 3 weeks) which led to striking clinical and laboratory response for up to 6 months but then lost effectiveness. Therefore we switched to the anti-IL-17A antibody secukinumab (up to 17mg/kg every 4 weeks) leading to substantial therapeutic response again. The patient has now been successfully treated with this therapy for 10 months together with a maintenance dose of prednisolone (0,2 mg/kg/d), electrolyte substitutions due to her tubulopathy and anti-hypertensive therapy. No substantial side effects have been observed so far. Further observation is needed for long term evaluation of the anti-IL-17 therapy response in this single patient.

**Conclusion:** This case report might encourage the use and report of high-dose IL-17 targeted therapies in further refractory Blau syndrome patients. The repeated secondary loss of effectiveness towards anti-IL-6 antibody tocilizumab and anti- IL-12 / IL-23 antibody ustekinumab after several months of successful treatment might be related to anti-drug antibody formation or the specific pathophysiology of Blau syndrome.

^1^ Wouters et al., Pediatric Rheumatology 2014 ² Jannsen et al., J Allergy Clin Immunol 2012

**Consent for publication has been obtained from patient**

Yes

**Disclosure of Interest**

None Declared

#### P2090 Recurrent fevers associated with neurodevelopmental disorders and their treatment with anakinra

##### Tina M. Romeo, Amanda K. Ombrello, Karyl Barron, Anne Jones, Seth Berger, Ann C. Smith, Daniel L. Kastner, Deborah L. Stone

###### National Institues of Health/NHGRI, Bethesda, United States

####### **Correspondence:** Tina M. Romeo

**Introduction:** Rubenstein-Taybi syndrome, a multiple congenital anomaly syndrome characterized by intellectual disability and dysmorphic facial features, is usually associated with mutations in the transcriptional coactivator CREB-binding protein.^1^ Mutations in the transcriptional factor, DEAF1, have been associated with *DEAF1*-associated neurodevelopmental disorder (DAND), characterized by intellectual disability, speech impairment and motor delay.^2^We describe the presence of a recurrent fever syndrome in a child with Rubenstein-Taybi syndrome and another with DAND.

We evaluated a child with Rubenstein-Taybi syndrome with recurrent fevers associated with grunting.During a severe flare, he had been found to have small pericardial and pleural effusions. He did not respond to colchicine and was treated frequently with prednisone. A second child was diagnosed with DAND and recurrent fevers that exacerbated his seizure disorder. Both children had increased inflammatory markers at the time of their fevers.

**Objectives:** To find a steroid-sparing treatment for these children.

**Methods:**Both children were treated with anakinra at the first sign of symptoms related to a fever flare.

**Results:** Both children responded rapidly with improvement in fevers, inflammatory markers, and other associated symptoms (grunting, poor appetite, diarrhea, and behavioral issues).

**Conclusion:** Anakinra may be effective in treating fevers and inflammation in children with syndromes caused by mutations in genes for transcription factors or activators. These children have dysregulation of multiple genes, including some plausibly involved in the innate immune system.

**Disclosure of Interest**

None Declared

#### P2091 Hematopoietic stem cell transplantation (HSCT) successfully curedtwo patients with PSTPIP1-associated autoinflammatory diseases (PAMI)

##### Alexandra Laberko^1^, Daria Yukhacheva^1^, Irina Shipitsina^2^, Maria Dunaykina^2^, Anna Kozlova^1^, Vasilii Burlakov^1^, Irina Mersiyanova^3^, Yulia Rodina^1^, Elena Raykina^3^, Dmitrii Balashov^2^, Larisa Shelikhova^2^, Anna Shcherbina^1^

###### ^1^Immunology; ^2^Hematopoietic Stem Cell Transplantation; ^3^Laboratory of Molecular Biology, National Medical Research Center of Hematology, Oncology and Immunology named after D. Rogachev, Moscow, Moscow, Russian Federation

####### **Correspondence:** Anna Shcherbina

**Introduction:** HSCT isan accepted curative treatment for diseases caused by genetic defects of immune system. Yet, it’s not as widely used in autoinflammatory syndromes since some of them affect not only hematopoietic, but also other cell types. PAMI caused by certain mutations of *PSTPIP1* gene andmanifests mainly withhematologic abnormalities (that can be life-threatening) and autoinflammation with or without purulent features.PAMI treatment is challenging as IL-1 inhibitors can alleviate the inflammatory symptoms but not hematologic features.Hence HSCT seems a logical treatment optionin PAMI.

**Objectives:** To describe treatment results of two PAMI patients.

**Methods:** The girl born in 2005 (P1) and the boy born in 2016 (P2) from unrelated families were diagnosed with PAMI based on detection of p.E250K mutation of*PSTPIP1*. TCR alfa/beta/CD19 depletion of the transplant and myeloablative preparative regimen with treosulfan 36-42g/m2, fludarabin 150mg/m2, thiotepa 10mg/kg were used forHSCT. Pre- and post-transplant immunosuppression in P1 consisted of tocilizumab 10mg/kg day -1, abatacept 10mg/kg days -1, +7, +14, +28 and bortezomib 1,3mg/m2 days -5, -2, +2, +5, in P2 thymoglobulin 5mg/kg and abatacept 10mg/kg days -1, +7, +28, both received rituximab 100mg/m2 day -1.

**Results:** Both patients had high inflammatory activity, fever episodes and cytopenia from the first year of life. P1was treated withIVIG, mycophenolate mofetil andrapamicin, with partialeffect, but later developedmyelodisplastic syndrome. P2 was treated with infliximab with partial effect, yet developed severe vasculitis and hemophagocytic lymphohistiocytosis and received treatment according to the HLH2004 protocol. Allogenic HSCT from matched unrelated donor in P1 andhaploidentical related donor in P2was performed at the age of 12 years (P1) and 18 months (P2). There were no severe posttransplant complications in both patients. Currently the patients are15 (P1) and 9 (P2) months after HSCT. Both patients have predominantly donor chimerism, good immune function, no inflammatory activity or other signs of PAMI.

**Conclusion:** PAMI syndrome can be successfully treated with HSCT. Indications for HSCT in other autoinflammatory syndromes have to be yet evaluated.

**Disclosure of Interest**

None Declared

#### P2092 Response to JAK inhibition in two children with haploinsufficiency of A20 (HA20) caused by truncating mutations in the ZNF4 domain

##### Deborah L. Stone, Amanda K. Ombrello, Karyl Barron, Tina Romeo, Natalia Sampaio Moura, Ivona Aksentijevich, Daniel L. Kastner

###### National Institutes of Health, U.S., Bethesda, United States

####### **Correspondence:** Deborah L. Stone

**Introduction:** Haploinsufficiency of A20(HA20) is an early onset autoinflammatory syndrome. The A20(TNFAIP3) protein is an important inhibitor of the NFκB signaling pathway, and individuals with HA20 frequently have increased inflammatory markers, fevers, oral and genital ulcers, and inflammation of the joints and eyes.^1^A20 has also been found to be a critical hepatoprotective factor ^2^ , and some individuals have presented with liver inflammation and fibrosis. Most mutations in A20 occur in the ovarian tumor (OTU) domain, but we follow two patients with heterozygous, *de novo* ZnF4 domain mutations, Thr604Argfs*93 and Leu626Valfs*45. The teenage girl with Thr604Argfs*93 had frequent and severe oral, genital and perirectal ulcers that required her to take frequent courses of prednisone and did not respond to etanercept, adalimumab or anakinra.The younger child with Leu626Valfs*45 had chronically elevated transaminases, decreased platelet count, increased prothrombin time and stage 3 liver fibrosis.

**Objectives:** To find an effective treatment for individuals with HA20 who have not responded to anti-IL1 and anti-TNF therapy or who have severe disease with liver involvement.

**Methods:** Because A20 was found to act as a regulator of STAT1^3^, JAK inhibition was proposed as a treatment. Gene expression testing of interferon related genes was performed before and after treatment. Tofacitinib was prescribed at a dose of 5 mg twice daily for the older girl and 2.5 mg twice daily for the younger one.

**Results:** Gene expression testing of interferon related genes showed upregulation in both girls before treatment. The older girl was started on tofacitinib and noted a great improvement with many fewer oral ulcers even when her dose of tofacitinib was reduced to once daily. Repeat gene expression testing post-initiation of therapy showed a reduction in expression of interferon related genes. After approximately 3 months of taking tofacitinib, the younger girl’s liver fibroscan (transient elastography) measurement had decreased from 13.5 Kpa, suggestive of advanced liver disease, to 8.7 Kpa (normal ≤7 Kpa). Her transaminases appeared to be slowly decreasing with a progressive increase in her platelet count.

**Conclusion:** Treatment with JAK inhibitors has benefited two children with HA20 who have truncating mutations in the Zn4 domain, demonstrating that HA20 is an interferonopathy in these patients. Further research may determine whether these results are generalizable to other individuals with HA20. Gene expression testing and treatment with JAK inhibitors should be considered in patients with HA20.

^**1**^ Zhou Q, Wang H, Schwartz DM, Stoffels M, Park YH, Zhang Y, et al. Loss-of-function mutations in TNAIP3 leading to A20 haploinsufficiency cause an early-onset autoinflammatory disease. Nat Genet 2016 Jan; 48(1):67-73. PMID 26642243

^2^ Catrysse L, Farhang Ghahremani M, Vereecke L, Youssef SA, McGuire C, Sze M, et al. A20 prevents chronic liver inflammation and cancer by protecting hepatocytes from death. Cell Death Dis 2016 Jun; 7(6): e2250. PMID 27253414

^3^ De Wilde K, Martens A, Lambrecht S, Jacques P, Drennan MB, Debusschere K, et al. Ann Rheum Dis 2017 Mar; 76(3): 585-592. PMID 27551052

**Disclosure of Interest**

None Declared

#### P2093 Squalene synthase inhibitors to regulate the inflammation in mevalonate kinase deficiency

##### Annalisa Marcuzzi^1^, Elisa Piscianz^1^, Erica Valencic^2^, Alessandra Tesser^2^, Andrea Taddio^1,2^, Alberto Tommasini^2^

###### ^1^University of Trieste; ^2^IRCCS Burlo Garofolo Trieste, Trieste, Italy

####### **Correspondence:** Alessandra Tesser

**Introduction:** Mevalonate Kinase Deficiency (MKD) is a rare inborn disease belonging to the family of periodic fever syndromes. MKD phenotype is characterized by multiorgan inflammation, which may involve the brain in the most severe cases. Anti-IL-1 therapies can contrast almost completely systemic inflammation, but it is uncertain whether they can prevent neural inflammation. Since it is believed that shortage of mevalonate derived isoprenoids plays a major role in inflammation, biochemical treatments modulating the mevalonate pathway may contribute to improve the cure of the disease.

**Objectives:** We hypothesize that repurposing of the squalene synthase inhibitor Lapaquistat could represent a promising therapeutic approach by increasing the accumulation of squalene isoprenoids with antinflammatory properties.

**Methods:** MKD shows different degrees of disease severity due to the residual mevalonate kinase (MK) activity, ranging from autoinflammatory hyper immunoglobulinemia D and periodic fever syndrome, with a 1-8% residual MK activity, to mevalonic aciduria in which MK activity is undetectable.

To mimic the spectrum of the biochemical defect of the mevalonate pathway, a consolidated MKD-model was used to test the anti-inflammatory potential of the Lapaquistat. Briefly, a murine monocyte/macrophage cell line was treated with increasing concentration of alendronate (25/50/100 μM) to obtain a biochemical block of the mevalonate pathway. At the same time, cells were stimulated with different concentration of Lapaquistat (10/25/50 μM). After 24 hours, cells were treated with bacterial stimuli (lipopolysaccharide) to mimic a generic inflammatory stimulus.

Cell viability, programmed cell death, morphology and cytokine release were the experimental readouts.

Cell viability was measured by means of impedance assay: the system allows to quantify cell proliferation, changes in morphology and the quality of cell adhesion to the plastic wells. These data were confirmed by flow cytometric programmed cell death assays. Cells morphology was assessed by electronic microscopy, and in particular the evaluation was focused to discriminate the mitochondrial and membranes size and shape.

Finally, after stimulation, culture supernatants were collected to analyse the cytokine concentration by means of Multiplex Assay, with particular reference to IL-1β, IL-6 and TNF-α.

**Results:** Lapaquistat was able to preserve the cellular morphology in MKD-cellular model, as assesses by impedance analysis. However, treatment with Lapaquistat didn’t reduce LPS-induced apoptosis.

As for the cytokine profile, as expected, the production of inflammatory cytokines IL-1β, IL-6 and TNF-α significantly increased following treatment with LPS in cells treated with alendronate in a dose-dependent manner. At low doses of alendronate, Lapaquistat was able to reduce the release of these cytokines, contrasting inflammation, with a significative effect on IL-6.

The morphological-qualitative analysis showed that Lapaquistat was able to preserve the membranes, to reactivate the correct process of mitophagy and to prevent the formation of toxic lipid aggregates, but only at lower concentrations of alendronate.

**Conclusion:** Based on these results, we could hypothesize an efficacy of Lapaquistat as a complementary treatment in MKD, to be added to currently available antinflammatory treatments to improve the control of inflammation. Moreover, it is possible that Lapaquistat alone could have a sufficient antinflammatory effects in subjects with milder MK defect. However, further studies in animal models are needed to evaluate whether the benefits of increasing pre-squalene isoprenoids overcame the costs of a reduction of post-squalene sterols in vivo, which might affect in other ways the cell function.

**Disclosure of Interest**

None Declared

#### P2094 IL1Β blockade by canakinumab leads into remission recurrent pericarditis associated with the MEFV variant P.GLU148GLN (E148 Q)

##### Maria Tsinti^1^, Maria Zamanakou^2^, Vasiliki Dermentzoglou^1^, Elena Tsitsami^1^

###### ^1^Pediatric Rheumatology Unit, First Department of Pediatrics, School Of Medicine, University Of Athens, Children’s Hospital “Aghia Sofia”, Greece, Athens; ^2^CeMIA, Larissa, Greece

####### **Correspondence:** Maria Tsinti

**Introduction:** Increasing evidence suggests that idiopathic Recurrent Pericarditis (RP) results from inflammasome activation. The autoinflammatory (AIF) origin is strongly supported by the efficacy of treatments typically applied in Familial Mediterrenean Fever (FMF) and other AIF diseases; colchicine and IL1 blockade. RP is met in TRAPS patients and represents the rarest manifestation of polyserositis of FMF. A minority of FMF patients suffer from RP as the sole manifestation. The effecticeness of treatment of RP refractory to colchicine with IL1 blockade by anakinra has been shown in control trials. The efficacy of IL1β blockade by canakinumab in RP has been scarcely described.

**Objectives:** To indicate the effectiveness of canakinumab in treating RPin a patient carrying the E148 Q *MEFV* variant, along with other AIF genes variants.

**Methods:** Case presentation

**Results:** A 12-year-old overweight girl was hospitalized for 15 days with fever; 39,5^o^C-2 spikes daily, myalgias, arthralgias, headache, hepatosplenoomegaly and pericarditis (chest pain, dyspnea, tachypnea ST elevation in ECGand moderate pericardial effusion on US; cardiomegaly& bilateral pleuritic effusion on X-Ray. Laboratory findings revealed leukocytosis-WBC=14400/uL, neu 80%, mild anemia-Hb=8,5 g/dl, elevation of acute phase reactants-CRP=184 mg/l, SAA=900 mg/l, ESR=85mm/hr, ferritin=558μg/L fibrinogen=445mg%, d-dimmers= 29μg/ml), mildly elevated γGT, normal cardiac enzymes and abnormal clotting studies (PT=19,2 sec, APTT=32,4 sec, INR=1,7). Infections were excluded and medical history was negative for drug administration, recurrent fevers or rheumatic disease manifestations. Serology revealed absent Antinuclear Autoandibodies, normal C3&C4 and elevated IgA . Ibuprofen was ineffective. Prednisolone (PDN) 60 mg daily was administered with immediate response. Two months later during steroid tapering pericarditis recurred with similar manifestations. PDN dose was increased to max. Colchicine 2 mg/d was initiated. Analysis for monogenic AIF diseases by a custom NGS protocol including 25 genes (mean coverage >92%) was performed. Common polymorphisms-UCSC Common SNPs- with no reported disease associations in the ClinVar database were excluded. Pathogenicity of variations was predicted by bioinformnatic analysis using SIFT and PolyPhen tools, in comparison to their European frequency (ExAc database). Detected mutations were cross-referenced with the International Society for Systemic AIF Disease (Infevers) and ClinVAr databases.The E148Q Variant of Uncertain Significance-VOUS-on the *MEFV* gene, along with a benign variant; p.Ala313Thr and a VOUS; p.Gly192Val of the *TMEM172* gene and a VOUS; p.Ala313Gly of the *PLCG2* gene were detected (reported according to the HGVS and the guidelines for the genetic diagnosis of hereditary recurrent fevers). E148Q as the only *MEFV* variant cannot support the diagnosis of FMF, due to its high frequency in the general population; however it has been detected in patients with typical FMF phenotype as the sole variant. Thus, RP was considered a manifestation of FMF in this patient. A 2nd relapse of pericarditis occurred in 2 months during PDN tapering. Colchicine was increased to 3 mg/d and PDN to 30 mg/d. After 3 months while on 7,5 mg PDN pericarditis relapsed with milder manifestations. PDN was increased to 15 mg, canakinumab, 150 mg, monthly was initiated and colchicine dose was adjusted to 2 mg/d. In 3 months steroids were interrupted. The disease remains into remission for 8 months under canakinumab.

**Conclusion: Conclusion**IL-1β blockade by canakinumab is effective and safe in recurrent pericarditis associated with polymorphisms in AIF genes.

**Consent for publication has been obtained from patient**

Yes

**Disclosure of Interest**

None Declared

#### P2095 Anakinra treatment in recurrent pericarditis: single center experience

##### Zeynep Toker Dincer, Osman Corbali, Serdal Ugurlu, Huri Ozdogan

###### Division of Rheumatology, Department of Internal Medicine, Cerrahpasa Medical Faculty, University of Istanbul - Cerrahpasa, Istanbul, Turkey

####### **Correspondence:** Serdal Ugurlu

**Introduction:** Recurrent pericarditis (RP), however the etiology is unknown in the majority, may be observed in autoinflammatory diseases such as familial Mediterranean fever (FMF) and tumor necrosis factor receptor-1 associated periodic syndrome (TRAPS). Colchicine has long been used to treat pericarditis related to FMF as well as patients with idiopathic recurrent pericarditis (IRP) (1). Alternative treatments have been reported for cases with colchicine resistant RP.

**Objectives:** The aim is to present our data regarding anakinra treatment in recurrent pericarditis either related to FMF or idiopathic, who are resistant to colchicine.

**Methods:** Patients who had recieved anakinra with a diagnosis of recurrent pericarditis either idiopathic or secondary to FMF followed in our autoinflammatory disease center between 2014-2018 are evaluated retrospectively. From patients’ files, demographic and clinical features, response to other treatment approaches such as NSAID, corticosteroid, colchicine, were evaluated. All patients have been genetically screened for monogenic autoinflammatory diseases (MEFV, TRAPS, MVK, NLRP3, NOD2). Patients who had at least 3 attacks were administered anakinra 100 mg/day. Therapeutic efficacy, as well as side effect profile of anakinra is also assessed.

**Results:** There were 5 patients (3 male and 2 female) with the diagnosis of RP, 1 was related to FMF and 4 were idiopathic. The mean age of the group was 28±8 (range 20-40). All patients diagnosed with IRP were negative for autoinflammatory genetic screening, while a MEFV variant (K695R het.) was detected in the FMF patient. Median duration of follow-up was 30 months (range 11-129). In table 1, demographic and clinical features are given. The median number of recurrence was 6 before anakinra treatment. No episode of pericarditis was observed in any of the patients after the initiation of anakinra. The response to anakinra persisted even after the dose was reduced to 100 mg/alternate day in 3 patients, however in 2, recurrence of pericarditis was observed and anakinra was escalated to initial dose. It was possible to discontinue corticosteroid treatment in all patients. Currently all patients continue anakinra treatment. No side effect including injection site reaction, has been observed by now.

**Conclusion:** Anakinra seems to be a safe and effective treatment approach for colchicine resistant recurrent pericarditis. However recurrence may occur during dose tapering.

**Reference**

1) Adler Y, et al. Colchicine treatment for recurrent pericarditis. A decade of experience. Circulation. 1998 2;97(21):2183-5.

**Consent for publication has been obtained from patient**

Yes

**Disclosure of Interest**

None Declared

**Table 1 (abstract P2095).** Demographic features and treatment response during anakinra therapy

Patient ID #	Age	Sex	Diagnosis	Duration of pericarditisfollow-up (mo)	Prior medications	Number of recurrences before anakinra	Anakinra treatment duration (mo)	Time to corticosteroid discontinuation (mo)	Number of recurrences after daily dose of anakinra
1*	23	M	IRP	129	Colchicine, NSAIDs, CS, HCQ	6	52	9	No
2	32	F	IRP	128	Colchicine, NSAIDs	7	4	NA	No
3*	40	F	IRP	21	Colchicine, CS	6	8	1	No
4	20	M	IRP	11	Colchicine, CS	3	8	2	No
5	25	M	FMF	30	Colchicine, CS	5	15	1	No

F: Female, M: Male, CS: Corticosteroid, HCQ: Hydroxychloroquine, NA: Not applicable

*Dose tapering was unsuccessful in these patients

### Immunedysregulation

#### P2096 Inaugural hemophagocytic syndrome in autoimmune diseases: a case series

##### Laura Damian^1^, Manuela Sfichi^2^, Mihaela Lupse^3^, Simona Cocu^4^, Laura Urian^5^, Cristina Pamfil^6^, Mihnea Zdrenghea^5^, Ina Kacso^7^, Simona Rednic^6^, Anca Bojan^5^

###### ^1^Rheumatology; ^2^Immunology, Emergency Clinical County Hospital Cluj; ^3^Infectious Diseases; ^4^Intensive Care; ^5^Hematology; ^6^Rheumatology; ^7^Nephrology, “Iuliu Hatieganu” University of Medicine Cluj, Cluj-Napoca, Romania

####### **Correspondence:** Laura Damian

**Introduction:** Hemophagocytic syndrome, lymphohistiocytosis or macrophage activation syndrome (MAS) is a severe, potentially lethal disease with unspecific features often mimicking sepsis. It may be triggered by infections or by the autoimmune disease itself.

**Objectives:** To describea series of MAS cases heralding systemic autoimmune diseases.

**Methods:** We retrospectively reviewed the patients’ records and charts in our tertiary referral units, in the last 5 years. The MAS cases in patients with a previously known autoimmune diagnosis were excluded. MAS was diagnosed according to the HLH-2004 criteria. Genetic tests were not performed.

**Results:** MAS was found to herald systemic diseases in 8 cases (7F, 1M, median age 46.2 years). The autoimmune diseases were: systemic lupus erythematosus (3 cases- all with anti-Ro+ and no anti-dsDNA, in 2 overlap with Sjogren’s syndrome), inflammatory idiopathic myopathy (2 cases), seronegative rheumatoid arthritis, systemic pyoderma gangrenosum and polyarteritis nodosa (1 case each). In all cases, prolonged fever and cytopenia in at least 2 lines were the presenting symptoms. Three cases required intensive care and life support; plasma exchange was employed in a case of thrombotic microangiopathy and immunoglobulins (1 g/kg) were given in the other two cases. Hiperferritinemia was noted in all cases, with large variations from 510 IU to over 10000 UI. Low NK counts and/or percentage (by flow cytometry BD FACS) were found in 5 of the 6 cases tested.

**Conclusion:** MAS may reveal the presence of autoimmune diseases, even when infectious triggers are proven. Clinical awareness, a great index of suspicion and a fast multidisciplinary approach in a referral center improve the prognosis.

**Consent for publication has been obtained from patient**

Yes

**Disclosure of Interest**

None Declared

**Table 1 (abstract P2096).** See text for description

No	S	Age	Trigger	Disease	Therapy	Outcome	Observations
1.	F	72	Resp. Virus	IIM	life support (ICU); MP pulses; cyclosporine; immunoglobulins	survival	ARF; hypoC4 Atypical rash
2.	F	56	Acute cholecystitis	PG	Life support (ICU); immunoglobulins; MP, CyA	survival	
3.	F	58	viral	RA	MP, CyA, MTX	survival	
4.	F	46	?	SLE/SSj (Ro52)	MP, CyA	survival	Evolved to Systemic sclerosis
5.	F	49	Q fever	SLE/SSj (Ro52) cryoglobulinemia	Life support (ICU); plasma exchange; MP, CyA	survival	TMHA; hypoC3, C4
6.	M	23	?	PAN	MP, CyA	survival	
7.	F	25	?	SLE	MP, CyA	survival	
8.	F	57	Resp. virus	inflammatory idiopathic myopathy	MP pulses; CyA, then Aza	survival	Atypical rash

Legend: ARF- acute respiratory failure, Aza- azathioprine, CyA- cyclosporin A,ICU- intensive care unit, IIM= inflammatory idiopathic myopathy, MP- methylprednisolone, PAN- polyarteritis nodosa, PG- pyoderma gangrenosum (systemic), RA-rheumatoid arthritis, SLE- systemic lupus erythematosus, SjS- Sjogren’s syndrome, TMHA- thrombotic microangiopathy

#### P2097 Assessing the mutation detection rate and genotype-phenotype correlation with a large haemato-immunological NGS panel: crossing the borders among different subspecialties

##### Alice Grossi^1^, Maurizio Miano^2^, Stefano Volpi^3^, Roberta Caorsi^3^, Francesca Fioredda^2^, Marina Lanciotti^2^, Enrico Capelli^2^, Carlo Dufour^2^, Marco Gattorno^3^, Isabella Ceccherini^1^

###### ^1^UOC Genetica Medica; ^2^UOC Ematologia; ^3^Clinica Pediatrica Reumatologia e UOSD Centro Malattie Autoinfiammatorie-Immunodeficienze, IRCCS Istituto Giannina Gaslini, Genova, Italy

####### **Correspondence:** Alice Grossi

**Introduction:** Next Generation Sequencing (NGS) has driven the rapid increase in the number of recognizable inborn errors of immune system development and/or function often hampered by the wide heterogeneity of the many genetically diverse but phenotypically overlapping diseases. NGS has also led to the discovery of new genes implicated in well-defined biological pathways, revisiting frequencies and broadening their phenotypic spectrum.

**Objectives:** Identification of the genetic causes of already known and unknown immune-mediated diseases with immunedysregulation; improvement of our knowledge about clinically overlapping phenotypes through the genetic characterization of the corresponding patients; optimization of the diagnostic work-up in order to administer disease specific treatments to patients.

**Methods:** A total of 150 patients, selected based on a clinical history highly evocative for immune-dysregulation (peripheral and/or central cytopenia and/or lymphoprolipheration and/or autoimmunity or autoinflammation) and/or immunodeficiency were submitted to 3 custom gene panels progressively larger and sequenced through the Ion Personal Genome Machine (PGM™) System. Based on the clinical phenotype, frequency and impact on the protein, variants were selected and validate by standard Sanger sequencing.

**Results:** Table 1 reports technical results for the 3 gene sets. The mutation detection rates for these panels were 2/30 (6%), 15/51 (29%), and 16/69 (23%), respectively, for a total of 35 pathogenic variants that correlate with the respective clinical phenotypes. The patients could be classified in: ALPS/ALPS-like (97, 26 of whichdiagnosed), cytopenia (33, 4 diagnosed), undefined autoinfiammatory (12, 2 diagnosed) and suspected immunodeficiency (8, 1 diagnosed). Moreover, variants of unknown significance (VUS) in potentially causative genes were also found in additional 14 patients (6/30; 4/51; 4/69). A number of variants, either pathogenic, likely pathogenic, or VUS have been detected in some cases at genes unexpected on the basis of the phenotypes, among which PRKCD, PIK3CD, IL7R, NCF1, TNFRSF13C, CASP8, thus confirming wide heterogeneity in the phenotypic spectrum associated with diseases sharing haemato-immune-rheumatological features. Conversely, several clinically similar cases did not reveal any relevant mutation, thus reflecting a genetic heterogeneity that is far from being disclosed yet.

**Conclusion:** The NGS approach has demonstrated excellent performances in the 1) evaluation of large genes and mutation detection, 2) overall timeliness of the gene panels, relying on continuous literature updates, and 3) identification of unexpected phenotypes for well-defined monogenic disease and definition of different disease clinical entities characterized by overlapping phenotypes. By contrast, due to the remarkable variability in clinical presentations, defining the appropriate list of genes for a given phenotype represents one key difficulty in the design of these panels. In the near future we will have to focus on the functional study of the many variants, especially VUS, that have emerged in a massive study like ours.

**Disclosure of Interest**

None Declared

**Table 1 (abstract P2097).** See text for description

	GENE PANEL 1	GENE PANEL 2	GENE PANEL 3
Library_capture technology	Ampliseq_ThermoFisher	Haloplex HS_Agilent	Haloplex_Agilent
#genes	58	146	315
Target size	209,54 kbp	341,073 kpb	769,995 kpb
Target bases analyzable (coverage)	197,35kbp (94,18%)	337,24kbp (98,88%)	750,99kbp (97,53%)
#patients analyzed	30	51	69
Mean total variant called/filtered	127/4	641/14	1413/25

#### P2098 Vitamin D status & its therapeutic role in pediatric patients with idiopathic nephrotic syndrome

##### Dina E. Sallam

###### Paediatric, Ain Shams University hospital, Cairo, Egypt

**Introduction:** Idiopathic nephrotic syndrome (INS) is the most common form of childhood nephrotic syndrome, the hallmark of the diseases still remains unknown, however; strong evidence suggests that it has an immune pathogenesis involving T-cells and its released cytokines including IFN-g. Vitamin D is a multifunctional hormone that exerts its biological activities mainly through the vitamin D receptor (VDR) and has an anti-inflammatory and immunomodulatory effect on many immunological disorders.

**Objectives:** This study aimed at detecting the changes in serum level of vitamin D in children with idiopathic steroid sensitive nephrotic syndrome at diagnosis, early remission and at full remission together with studying the effect of vitamin D intake and other factors on achieving early remission and studying serum level of interferon gamma (IFN-g) as a marker of immunoregulatory effect of vitamin D.

**Methods:** This study included twenty five newly diagnosed children with idiopathic steroid sensitive nephrotic syndrome and was randomly categorized into two groups; both received oral prednisolone at diagnosed; meanwhile, only group 2 received vitamin D injection. Vitamin D statuses were detected at initial diagnosis, early remission and at full remission together with the serum level of IFN -g.Follow up was made over a period of 28 days.

**Results:** Study results revealed a deficiency of 25 (OH) D_3_ in our patients with INS, which improved markedly with remission especially if combined with vitamin D injection, while serum levels of 1, 25 (OH)_2_ D_3_ were normal from the start. Serum levels of IFN-g were at lower levels which increased with remission; meanwhile its level did not increase significantly in vitamin D supplemented group. Dyslipidemia improved with vitamin D injection. Interestingly, the most determinant factor for achieving early remission was the younger of our patients at diagnosis.

**Conclusion:** deficiency of 25 (OH) D_3_ is common among children with INS and improves with remission which emphasizes the effect of proteinuria and loss of vitamin D binding protein on serum level of 25 (OH) D_3_. On the other hand, active vitamin D (1, 25 (OH)_2_ D_3_) does not seem to be affected. Vitamin D has no significant effect on serum IFN-g. Both vitamin D and IFN-g do not appear to have significant role in the immunopathogenesis of nephrotic syndrome. Screening for vitamin D deficiency in children with INS and administration of therapeutic doses of vitamin D will help remission. Vitamin D supplementation has a protective lowering effect on serum cholesterol.

**Disclosure of Interest**

None Declared

#### P2099 Immune reconstitution in autoinflammatory CANDLE like syndrome due to SAMD9L mutation

##### Analia G. Seminario^1^, María S. Caldirola^1^, Ileana Moreira^1^, Valeria Regairaz^1^, Marco Gattorno^2^, Raphaela Goldbach-Mansky^3^, Liliana Bezrodnik^1^

###### ^1^Immunology, Centro de Inmunología Clínica Dra Bezrodnik, Buenos Aires, Argentina; ^2^Rheumatology, IRCCS Istituto Giannina Gaslini, Genova, Italy; ^3^Translational Autoinflammatory Disease Studies, NIH/NIAID/LCID, Bethesda, United States

####### **Correspondence:** Analia G. Seminario

**Introduction:** Autoinflammatory diseases are inborn errors of the innate immune system. They present with uncontrolled systemic and organ specific inflammation, like infections, that generally associate inflammation of the skin and others organs since neonatal period and raised inflammatory markers in blood. Some may not have fever. In recent years, new mutations were discovered, as causes of primary immunodeficiencies. SAMD9L expression is high in B and NK lymphocytes, moderate in T cells, monocytes, neutrophils, lung and muscle and low in skin, liver, heart and kidney tissues. Missense mutation in SAMD9L can cause, an Early-Onset Immune- Dysregulation Syndrome of Neutrophilic Panniculitis, Interstitial Lung Disease and Cytopenias.

**Objectives:** Report clinical case and immune reconstitution 3 years after bone marrow transplant in a patient with severe neonatal autoinflammatory syndrome due to SAMD9L mutation, with innate and adaptive immune compromise.

**Methods:** Retrospective analysis of a clinical history and immune reconstitution in a patient with a new primary disease of immune dysregulation.

**Results:** 3 years old girl, with chronic and generalized pustulosis since birth. At 2 months, persists with severe skin ulcers, no fever but high levels of CRP:78.5 mg/dl and ERS:68 mm^3^/h. In the immunological lab: hypogammaglobulinemia and almost absent B cell counts. Because of that she began treatment with intravenous gammaglobulin and corticosteroid, in spite of it she presented several skin exacerbations. So anti-TNF α (0.8 mg/dose weekly) treatment was added. She could improve skin lesions and almost normalized CRP but at fifth dose, she presented low platelets, white cells count and visceromegaly with respiratory failure because of Pneumocity Jiroveci and skin erythema and adenopathy at BCG site, needing mechanical ventilation, antibiotic, corticosteroid, cyclosporine and tuberculostatics drug because: positive Mycobacterium bovis in blood. After this treatment she presented partial response with marrow aplasia and she received canakinumab (ant IL-1β) 1 mg/kg/dose. No mutation in ADA2, TNFRSF1A, CECR1 and NLRP3 was found. Given the severe compromise, new studies were performed: Altered score for interferonopathies and mutation in SAMD9L protein was found by WES. Two months later, she was clinically stable. In 2016 she received unrelated HSCT (10/10). Nowadays she presents clinically well, no infections, only has mild rash in skin. No cytopenias, normal immunoglobulins, with B cell engraftment 15% (18,3 %B memory cells, 10,3% post switched memory cells and 3,2 % B transitional cells) Cd4 naïve T cells: 29,8% - 169 mm^3^, Normal NK compartment and T cells proliferations and normalized serum proinflammatory cytokines, with good evolution up to today and stopped GvHD treatment, only continue with weekly subcutaneous gammaglobulin

**Conclusion:** Severe autoinflammatory syndrome represent a challenge for clinical immunologists. HSCT was a curative treatment for our patient, who presented with a severe neonatal autoinflammation due to an immunodeficiency that affect innate and adaptive immune response

**Consent for publication has been obtained from patient**

Yes

**Disclosure of Interest**

None Declared

#### P2100 FASL gene mutation in a child with autoimmune lymphoproliferative syndrome

##### Madhubala Sharma^1^, Amit Rawat^1^, Deepti Suri^1^, Vignesh Pandiarajan^1^, Koon W. Chan^2^, Yu L. Lau^2^, Surjit Singh^1^

###### ^1^Allergy amd Immunology Department, Post Graduate Institute of Medical Education and Research, Chandigarh, India; ^2^Department of Pediatrics and Adolescent Medicine, Queen Mary Hospital, Hong Kong, Hong Kong

####### **Correspondence:** Madhubala Sharma

**Introduction:** Autoimmune lymphoproliferative syndrome (ALPS) is characterized by immune dysregulation due to a defect in lymphocyte apoptosis. The clinical manifestations may be noted in multiple family members and include lymphadenopathy, splenomegaly, increased risk of lymphoma, and autoimmune disease, which typically involves hematopoietic cell lines manifesting as multilineage cytopenias. Since the disease was first characterized in the early 1990s, there have been many advances in the diagnosis and management of this syndrome. The inherited genetic defect of many ALPS patients has involved (FAS) pathway signaling proteins.

**Objectives:** To demonstrate genetic variation in a case of autoimmune lymphoproliferative syndrome.

**Methods:** This case describes a 22-month-old female symptomatic from 2.5 months of life. She had severe pallor, received a blood transfusion (TORCH infections panel- negative). At 5.5 months she had anemia and ecchymoses over extremities and splenomegaly, which Improved with oral prednisolone, however, she developed relapse during tapering of oral prednisolone doses with cervical and axillary lymphadenopathy, hepatosplenomegaly- spleen 6 cm below left costal margin.

There was no significant family history. Her elder male sibling is 9 years old, he is alive and healthy.

**Results:** Following Investigations were made for her at 2.5 months and at 22 months:

I. 2.5 months

1. Hemoglobin- 3.3 g/dl, white cell counts- 39,600/cu.mm, differentials- P14 L58 M26 E12, platelet counts- 40,000/cu.mm, reticulocyte counts- 11.6%, LDH values- 306 IU, peripheral smear- anemia, thrombocytopenia with lymphocytosis

2. Bone marrow- erythroid hyperplasia with increased lymphocytes

3. CD3 cell counts- 13,376/cu.mm, CD4 cell counts- 10,883/cu.mm, CD8 cell counts- 7,332/cu.mm, B cells- 6000/cu.mm, Double negative T cells- 23.9%

II. 22 months

IgG- >2509 mg% (310-1380)

IgA- 266 mg% (50-220)

IgM- 285 mg% (40-229)

Direct Coombs test- positive for C3d

Double negative T cells- 7.6% of all CD3+TCRαβ+ cells

Antinuclear antibody- negative

Antiphospholipid antibodies- negative

Flow cytometric Annexin V assay: effective apoptosis in the case was 9.8% (REDUCED) compared to 41.2% in control

Sequencing of the FAS ligand gene identified a homozygous nonsense mutation in exon 1, c.343C>T, p.R115X. Father and mother both are heterozygous carrier the same mutation.

**Conclusion:** FASL gene mutations account for < 5% of all ALPS patients. The identification of a nonsense mutation in the FASL gene confirms the diagnosis. However, serum FASL levels could not be estimated in the child or the parents.

**Consent for publication has been obtained from patient**

Yes

**Disclosure of Interest**

None Declared

#### P2101 Characteristics of the immunological status in patients with systemic and articular form of juvenile idiopathic arthritis

##### Anna Y. Spivakovskaya, Yuri Spivakovskiy, Yuri Chernenkov

###### Depertment of Hospitality Pediatrics, Saratov State Medical University, Saratov, Russian Federation

####### **Correspondence:** Anna Y. Spivakovskaya

**Introduction:** Juvenile idiopathic arthritis (JIA) brings together a diverse group of chronic joint diseases. JIA is one of the most serious and potentially disabling pathologies in pediatric rheumatology. The high frequency of occurrence, the tendency to the progression of the pathological process, the possibility of systemic manifestations with the involvement of internal organs, dictates the need for a more thorough and comprehensive examination of patient's data. Identification of the features of the immunological reaction in patients with various forms of the course of JIA has not lost its relevance.

**Objectives:** To compare changes in cytokine (IL-1β, IL-6, INF-γ, IL-4) and chemokine (MCP-1) profiles in patients with systemic and articular JIA.

**Methods:** The study involved 96 patients, ranging in age from 1,1 years to 16,11 years (mean age 9,8 ± 4,2 years). A group of children with a verified diagnosis of JIA consisted of 76 patients with a medicamentally supported inactive disease stage and a remission stage. Of these, 20 patients with the systemic variant of the disease and 56 children with the articular form of JIA (30 patients with oligoarticular and 26 with the polyarticular variant of JIA). The comparison group consisted of relatively healthy children (n = 20). The concentration of interleukins and chemokine in the serum of patients was determined by enzyme immunoassay (ELISA) using ready-made commercial detection kits - IL-1β, IL-4, IL-6, INF-γ and chemokine MCP-1 from the company “Vector-Best” (Novosibirsk, Russia). The study was performed on a “Lazurite” automated immunoassay analyzer (Dynex Technologies Inc., USA).

**Results:** The main changes in cytokine status in the examined patients were associated with an increase in serum concentrations of IL-1β, IL-6 and INF-γ in relation to the change in these parameters in volunteers from the comparison group. The serum IL-1β concentration in the articular form of the disease was 1,1-1,7 times higher than in patients with systemic JIA. The level of IL-6 in systemic arthritis was 1,2-1,6 times higher than in the articular form of JIA. If the concentration of INF-γ in oligoarthritis was only 1,3 times higher than in the control group, then in polyarthritis and systemic arthritis it was 2,6-2,7 times higher, respectively. The course of JIA characterized an increase in the level of chemokine MCP-1 in all variants of the disease by an average of 1,5-1,9 times relative to the comparison group. Changes in the level of MCP-1 correlated with the concentration of IL-1β and IL-6 (r = 0,6; p <0,05) in polyarthritis and with IL-6 (r = 0,5; p <0,05) in systemic version of JIA.

**Conclusion:** The revealed fluctuations in immunological parameters in various variants of JIA did not go beyond the general trends of the current pathological process. At the same time, a number of features have been established that characterize various forms of JIA. Investigation of the MСP-1 chemokine reaction makes it possible to increase the informativeness of the JIA verification algorithm.

**Disclosure of Interest**

None Declared

### Unusual or unsolved case reports

#### P2102 Early onset Behcets disease in an Indian family: is it related to A20 haploinsufficiency?

##### Vignesh Pandiarajan, Deepti Suri, Amit Rawat, Anju Gupta, Surjit Singh

###### Allergy Immunology Unit, Pediatrics, Postgraduate Institute of Medical Education and Research, Chandigarh, India

####### **Correspondence:** Vignesh Pandiarajan

**Introduction:** A20 haploinsufficiency due to defect in *TNFAIP3* has been recently described as a monogenic cause for early-onset Behcets disease. Characteristic features include presence of recurrent oral, genital and gastrointestinal ulcerations, fever, and presence of multiple autoantibodies.

**Objectives:** To describe a child with early-onset Behcets disease who was identified to have a defect in *TNFAIP3*

**Methods:** A 6-year-old girl was symptomatic since 6 months of age in form of recurrent oral ulcers, fever, and genital ulcers. She had elevation in erythrocyte sedimentation rate (ESR) (40 mm at 60 min) and C-reactive protein (CRP) (32 mg/L). HLA B51 and HLA B27 were positive by polymerase chain reaction (PCR). Oral ulcers had shown a partial response to colchicine therapy. She developed abdominal pain and gastrointestinal bleeding at the age of 4 years. Colonoscopy revealed increased vascularity in terminal ileum and biopsy revealed non-specific inflammation. Azathioprine was used to control the bowel symptoms. She developed headache and blurring of vision at age of 5.5 years and fundus revealed papilloedema. MRI Brain revealed features of type 2 Arnold Chiari malformation. Antinuclear antibodies and anti-neutrophil cytoplasmic antibodies were negative by indirect immunofluorescence. Her younger brother had recurrent oral ulcerations from the age of 8 months.

**Results:** Genomic DNA was extracted and was subjected to Next Generation Sequencing using an exome panel for known autoinflammatory disorders. A heterozygous missense variation in exon 7 of the ***TNFAIP3*** gene (c.1504C>T**;** p.Arg502Trp) was detected. The variant has not been reported in the 1000 genomes database and *in silico* predictions are possibly damaging by PolyPhen-2 (HumDiv), and damaging by SIFT, LRT and Mutation Taster2. The mutation is validated later by Sanger sequencing.

**Conclusion:** We describe a case of early-onset Behcets Disease from an Indian family probably related to a novel heterozygous *TNFAIP3* defect, however, functional validation of the genotype is warranted to conclusively prove the association. Identification of underlying genetic defect could pave way for future development of targeted therapies for abrogation of inflammation.

**Consent for publication has been obtained from patient**

Yes

**Disclosure of Interest**

None Declared

#### P2103 Two types of systemic amyloidosis in a single patient

##### Riccardo Papa^1^, Janet A. Gilbertson^2^, Nigel Rendell^2^, Ashutosh D. Wechalekar^2^, Julian D. Gillmore^2^, Philip N. Hawkins^2^, Helen J. Lachmann^2^

###### ^1^Clinica Pediatrica e Reumatologia, IRCCS Istituto Giannina Gaslini, Genova, Italy; ^2^National Amyloidosis Centre, University College London Medical School, Royal Free Hospital Campus, London, United Kingdom

####### **Correspondence:** Riccardo Papa

**Introduction:** The systemic amyloidoses are a group of rare diseases, in which extracellular deposition of a variety of proteins in an abnormal fibrillar confirmation results in life-threatening organ dysfunction. Acquired and hereditary amyloidoses differ in their precursor proteins and predilection for specific organ involvement. Two types of fibril have been reported in a single organ, and can complicate the same disease, but two separate types of systemic amyloidosis have not previously been described in a single patient.

**Results:** We report a woman of Sudanese origin who presented aged 31 with dysuria and haematuria. She was found to have an estimated Glomerular Filtration Rate of 38 ml/min and no proteinuria, and a renal biopsy demonstrated AA amyloid deposition. An I^123^ labelled SAP scan demonstrated a small amount of amyloid confined to the kidneys. She had no overt underlying inflammatory disease, an infectious diseases work up, including blood borne viruses, was negative and serial measurement of serum amyloid A protein showed no significant elevation with a median of 5 mg/L. Retrospective molecular analysis of the *MEFV* and *TNFRSF1A* genes, underlying Familial Mediterranean Fever and the Tumor Necrosis Factor Receptor Associated Periodic Syndrome, revealed the heterozygosity for *MEFV* E148Q and *TNFRSF1A* P46L common sequence variants of unknown significance. Interpretation of the significance polymorphisms in two fever genes remains contentious but given her persistently normal acute phase reactants they were felt to be of no clinical relevance. Homozygosis for SAA1.1 allele of the *SAA1* gene is a recognised risk factor for AA amyloidosis, but testing showed she was heterozygous. Management was blood pressure control only, and her inflammatory markers and renal function remained stable until she was lost to follow up 3 years later.

Thirteen years after her renal biopsy she represented in end stage renal failure with a history of weight loss, deranged liver function tests and marked easy bleeding. Further investigation demonstrated well controlled C reactive and serum amyloid A proteins, and an IgG lambda M-band with no serum free light chain bias. A bone marrow demonstrated 7% neoplastic plasma cells and was complicated by a retroperitoneal bleed. An SAP scan now showed a large amyloid load with amyloid deposition in the liver and spleen obscuring the kidneys.Review of the bone marrow and a duodenal biopsy demonstrated amyloid deposition which was AL (lambda) type by both immunohistochemistry and proteomic analysis after laser dissection and mass spectroscopy. Review of the original biopsy confirmed AA type amyloid by both immunohistochemistry and proteomics. Six-cycle chemotherapy for AL amyloidosis was administered with complete clonal response. She remained on dialysis and died four years later of a cerebrovascular accident.

**Conclusion:** This is the first reported case of two types of amyloidosis developing consecutively in a single individual. The underlying inflammatory driver of her AA amyloidosis was never identified and given that she had migrated some years earlier from Africa, previous chronic infection that has resolved or responded to non-disclosed prior treatment was thought to be the most likely cause. Whether the subsequent development of AL amyloidosis was pure chance remains unclear. Theoretically chronic inflammation/infection may drive generation of oligoclonal bands with the potential for monoclonal breakthrough. Whether her AA amyloid deposits played a role by providing a template for deposition of subsequent AL amyloidosis derived from an entirely separate precursor protein is also unknown although this theoretically possible and has been shown in reverse in mice models.

**Consent for publication has been obtained from patient**

Yes

**Disclosure of Interest**

None Declared

#### P2104 MAFB not seems to regulate serum C1Q concentration in humans

##### Riccardo Papa^1^, Annalisa Madeo^2^, Stefano Volpi^1^, Roberta Caorsi^1^, Giancarlo Barbano^3^, Marina Botto^4^, Andrea Superti-Furga^5^, Marco Gattorno^1^, Maja Di Rocco^2^, Paolo Picco^1^

###### ^1^Clinica Pediatrica e Reumatologia; ^2^Malattie Rare; ^3^Nefrologia e Trapianto di Rene, IRCCS Istituto Giannina Gaslini, Genova, Italy; ^4^Faculty of Medicine, Imperial College London, London, United Kingdom; ^5^Division of Genetic Medicine, Centre Hospitalier Universitaire Vaudois, Lausanne, Switzerland

####### **Correspondence:** Riccardo Papa

**Introduction:** MafB-deficient mice develop systemic lupus erythematous-like autoimmune disease by inhibited C1q production and efferocytosis. MafB-deficient humans develop multicentric carpotarsal osteolysis syndrome (MCTO), frequently followed by progressive nephropathy.

**Objectives:** To investigate a possible role of C1q deficiency in patients with MCTO.

**Results:** Three patients with MCTO were enrolled (table 1). The patient A presented recurrent urinary tract infections since 18 months of life for a third-degree vesicoureteral reflux. Despite correction surgery, he displayed proteinuria after one year, and kidney biopsy did not revealed significant lesions. After two years, he displayed bilateral tarsal malformations requiring orthopedic surgery. At the age of 6, a pathological fracture of the right wrist occurred, and local X-ray revealed osteolysis of carpal bones. The patient B was treated with intra-articular steroid injection and subcutaneous methotrexate for one year without benefit because of a suspected juvenile idiopathic arthritis of the right wrist. After one year, he developed proteinuria and kidney biopsy revealed focal segmental glomerular lesions with tubular fibrosis and renal vasa sub-intimal delamination. C3 and C4 positive mesangial deposits were also present. The patient C was sent to our observation for a suspected juvenile idiopathic arthritis of the right wrist. No inflammatory signs were present at the ultrasound examination. Sanger sequencing of the *MAFB* gene revealed in all the patients a novel heterozygous mutation. Although specific variants have not been reported before, the nature and position of the variants associated with the clinical features of the patients strongly argued in favor of their pathogenic role. The serum C1q concentration was normal in all the patients.

**Conclusion:** MCTO-associated progressive nephropathy is an orphan disease with dramatic impact on the patient life who usually require kidney transplantation in the adulthood. For that reason, despite ACE-inhibitor could reduce progression of kidney dysfunction, retarding the onset of end state kidney disease, other treatments should be used to permit a sufficient lifelong kidney function in patients with the well-known structural kidney damage related to MafB-deficiency. Furthermore, a slight proteinuria reduction in a patient with MCTO treated with cyclosporine has been reported. However, despite further studies are necessary to exclude a role of complement system in the progressive nephropathy of patients with MCTO, our study seems to exclude a MafB-dependent regulation of complement component C1q production in humans.

**Consent for publication has been obtained from patient**

Yes

**Disclosure of Interest**

None Declared

**Table 1 (abstract P2104).** Main features of three patients with multicentric carpotarsal osteolysis syndrome

	Patient A	Patient B	Patient C
Age (years); gender; country of origin	14; M; Croatia	16; M; Italy	11; F; Russia
*MAFB* mutation	c.184A>G, p.Thr62Ala	c.172A>G, p.Thr58Ala	c.208T>C, p.Ser70Pro
Bone lesions onset (age; years)	5	8	8
Presentation	Bilateral	Monolateral	Monolateral
Site	Tarsus	Carpus	Carpus
Current state (except carpotarsal joints)	Elbows and knees contractures	Knees contractures	None
Nephropathy onset (age; years)	3	9	None
Current state	Proteinuria (720mg/24h)	Proteinuria (1.3g/24h)	Normal kidney function
Serum C1q concentration	174 mg/l	312 mg/l	176 mg/l

#### P2105 Sibship with uveitis and sensorineural hearing loss

##### Pallavi Pimpale Chavan^1^, Mayur Morekar^2^, Raju P. Khubchandani^1^

###### ^1^Section of Pediatric Rheumatology, SRCC Children’s Hospital; ^2^Consultant Eye Surgeon, Bombay Hospital & Medical Research Centre, Mumbai, India

####### **Correspondence:** Pallavi Pimpale Chavan

**Introduction:** We hope to use this forum to guide us in the diagnosis for this family.

**Objectives:** We describe a family from India with three siblings having uveitis and sensorineural hearing loss.

**Methods:** A 16 years male born of a non consanguineous marriage, sixth by birth order was referred to us by an ophthalmologist for bilateral anterior non granulomatous uveitis with band shaped keratopathy. There was no history of any constitutional symptoms or apparent hearing loss.

On further enquiry and on reviewing records, there was a significant history of other two female siblings suffering with uveitis and gradually progressive sensorineural hearing loss while other 3 siblings (2 females and 1 male) were well.

**Results:** On examination, he had conjunctival congestion with no syndromic features. Systemic examination was normal. His complete blood picture, erythrocyte sedimentation rate, urine routine, urine for beta 2 microglobulin, mantoux test and chest X ray were all normal. Due to a positive family history of hearing loss with uveitis, his Brainstem Evoked Response Audiometry (BERA) was done which was suggestive of mild sensorineural hearing loss.

He was started on topical steroids along with oral methotrexate (10.7mg/m^2^). Gradually his topical steroids were tapered and stopped while oral methotrexate (9.7mg/m^2^) was continued. His repeat BERA after a year has not shown any further deterioration and no other systems or organs have been involved.

**Conclusion:** Literature search for this unique sibship with uveitis and sensorineural hearing loss pointed us towards tubulointerstitial nephritis, uveitis, hearing loss and vestibular failure (TINU) with atypical Cogan's overlap syndrome (Brogan.K et al) but our three siblings screened negative for renal disease. We are awaiting further genetic analysis. (Courtesy – Dr Daniel Kastner and Dr Ivona Aksentijevich)

**Consent for publication has been obtained from patient**

Yes

**Disclosure of Interest**

None Declared

#### P2106 An unsolved case including granulomatous panuveitis from India

##### Pallavi Pimpale Chavan^1^, Mihir Kothari^2^, Raju P. Khubchandani^1^

###### ^1^Section of Pediatric Rheumatology, Jaslok Hospital and Research Centre; ^2^Pediatric Ophthalmologist, Jyotirmay eye clinic for children and binocular vision laboratory, Mumbai, India

####### **Correspondence:** Pallavi Pimpale Chavan

**Introduction:** We hope to use this forum to guide us in the diagnosis and management of this child.

**Objectives:** We describe a child from India, who presented with bilateral congenital glaucoma, recurrent granulomatous uveitis with episodes of fever, drowsiness, rash and arthritis.

**Methods:** A 5.6 years old male born of a second degree consanguineous marriage presented at birth with watery eyes and was diagnosed with bilateral congenital glaucoma at 2 months of age. Since 8 months of age, he had episodes of fever with recurrent granulomatous uveitis and ‘boil’ like rash on his face since 1 year of age. Gradually the frequency of fever spikes increased to 2-3 per month with drowsiness and increase in rash. At 2 years of age, he developed swelling of the right knee.

There is a notable family history of vertically transmitted sensorineural deafness in paternal grandfather, mother and elder sibling with arthritis in mother and maternal uncle.

**Results:** When he presented to us, on examination, he had failure to thrive, delayed milestones, poor vision, hearing loss, erysipelas like rash on the face and limbs with hepatosplenomegaly and arthritis of the right knee. His investigations were suggestive of anaemia (5.1 - 9.4 g/dl), normal leucocyte counts with monocytosis (max 13%), intermittent thrombocytopenia(30000/cmm) with raisedESR (14-124mm/hr) and CRP(13-368 mg/l). He had hypertriglyceridemia with mild ferritin elevation and (1:40) 1+ ANA.

His Immunoglobulin profile, Bone marrow, skin and conjunctival biopsy were all normal. Computed Tomography (CT) brain didnot show any intracranial calcification. Mutation studies were sent for BLAU and CAPS, which were negative. His interferon signals were negative.

To date he has received oral corticosteroids, methotrexate, colchicine and thalidomide (as canakinumab and anakinra are not available in our country). After starting thalidomide, in the last 10 months his frequency of fever, rash, arthritis and uveitis has reduced.

He is regularly taking topical drugs and following up with the ophthalmologist and has eventually developed cataract, retinal detachment, left eye corneal decompensation, pthisis bulbi (all sequelae of the panuveitis).We propose to start adalimumab for him.

**Conclusion:** We have achieved quiescence of disease in this child but do not have a diagnosis and are awaiting further genetic analysis. (Courtesy – Dr Daniel Kastner and Dr Ivona Aksentijevich)

**Consent for publication has been obtained from patient**

Yes

**Disclosure of Interest**

None Declared

#### P2107 An unsolved case: is this a Candle-like syndrome?

##### Alessia Pin^1^, Alessandra Tesser^2^, Flavio Faletra^2^, Alberto Tommasini^2^, Serena Pastore^2^, Andrea Taddio^1,2^

###### ^1^Department of Medicine, Surgery and Health Sciences, University of Trieste, Italy; ^2^Institute for Maternal and Child Health IRCCS Burlo Garofolo, Trieste, Italy, Trieste, Italy

####### **Correspondence:** Alessia Pin

**Introduction:** Chronic Atypical Neutrophilic Dermatosis with Lipodystrophy and Elevated Temperature (CANDLE) syndrome is a complex autoinflammatory disorders arising from inborn defects in immunoproteasome. Several genes can be involved and cases with digenic inheritance have been described. However, many cases remain without the identification of a specific genetic defect. A positive interferon signature is typically found in patients and may serve as a diagnostic clue.

**Objectives:** To describe clinical and genetic features in a girl with CANDLE and in her relatives with a variety of different rheumatologic complaints.

**Methods:** We performed Whole Exome Sequencing (WES) on 10 family members. Moreover, we assessed RNA-seq on three sample from the proband, collected during acute phases of disease (samples positive to class I interferon signature (IS)) (1), and her parents. Results of RNA sequencing (RNA-seq) were compared with specimens from healthy controls.

Differentially expressed genes (DEGs) were filtered by fold change > 2 and padj < 0.05. DEGs enrichment were performed using different R packages, such as pathfindR.

**Results:** We describe the case of a 20 years old girl with clinical and biological data supportive of CANDLE syndrome. At the age of 3 years, she started presenting a clinical picture reminiscent of amyopathic dermatomyositis, with skin rash, lipodystrophy, subcutaneous panniculitis nodules, and more recently with chilblains, skin ulcerations, polyarticular arthritis and alopecia.

Her pedigree includes several relatives with rheumatic disorders, but none has a clinical picture as complex and severe as our patient.

This girl was found to have a strongly positive class I IS in peripheral blood cells. After several unsuccessful therapeutic attempts with antirheumatic drugs and biologics, the girl showed a dramatic clinical response to the JAK inhibitors tofacitinbib.

IS resulted positive also in 4 of her relatives, three of whom presented also rheumatologic symptoms. Conversely, one uncle of the girl was affected with rheumatologic symptoms but had negative IS. The pedigree may suggest a complex pattern of inheritance, likely with a major dominant disorder, whose expression can be modulated by multigenic and/or environmental factors.

WES failed to detect significant genetic variants in proteasome components. However, RNA-seq revealed a profile of differentially expressed IFN-regulated genes similar to that reported by Anja Brehm et al. in subjects with CANDLE/PRAAS (2).

**Conclusion:** Our results suggest that our family may present a multigenic form of CANDLE, with a complete clinical picture only in the proband, whilst other relatives may only present partial or incomplete forms of the disease.

1.Rice GI, et al. Assessment of Type I Interferon Signaling in Pediatric Inflammatory Disease. J Clin Immunol. 2017 Feb;37(2):123-132. doi: 10.1007/s10875-016-0359-1. Epub 2016 Dec 9.

2.Brehm A, et al. Additive loss-of- function proteasome subunit mutations in patients with CANDLE/PRAAS promote type I IFN production. J Clin Invest. 2016 Feb;126(2):795. doi: 10.1172/JCI86020. Epub 2016 Feb 1.

**Consent for publication has been obtained from patient**

Yes

**Disclosure of Interest**

None Declared

#### P2108 A case of sting-associated vasculopathy with onset in infancy (SAVI) without skin involvement

##### Carmela Gerarda Luana Raffaele, Virginia Messia, Manuela Pardeo, Camilla Celani, Silvia Federici, Gianmarco Moneta, Ivan Caiello, Claudia Bracaglia, Fabrizio De Benedetti, Antonella Insalaco

###### Division of Rheumatology, IRCCS, Ospedale Pediatrico Bambino Gesù, Rome, Italy

####### **Correspondence:** Carmela Gerarda Luana Raffaele

**Introduction:** STING-associated vasculopathy with onset in infancy (SAVI) is a newly described type I interferonopathy caused by autosomal dominant gain-of-function mutations in Transmembrane Protein 173 (TMEM173) gene. It is characterized by early-onset systemic inflammation with increased levels of acute phase reactants, severe cutaneous vasculopathy leading to extensive tissue loss and severe interstitial lung disease (ILD)

**Objectives:** to describe an atypical clinical phenotype

**Methods:** A 4 year-old young girl presented since the first months of life with recurrent fevers, arthralgia, persistent cough and failure to thrive (below the 5th percentiles). She never showed skin rash. At the age of 3, she was admitted to our hospital for persistent fever and arthritis of left knee and right ankle. She started ibuprofen without any benefit. Laboratory tests showed persistently elevated CRP and serum amyloid A levels, persistent microcytic anemia and increased IgG levels. Highly positive antinuclear antibody (title 1:640, homogeneous pattern) with positive p-ANCA was detected. Immunological and cytogenetic studies performed on bone marrow were normal. Both sweat and genetic test for cystic fibrosis were negative. She started low-dose glucocorticoids with incomplete response, persistent cough and polyarthritis in small joints. Chest X-ray and CT scan showed a severe interstitial lung disease (ILD) with bronchiectasis and small nodules. Extensive investigations for bacteria, virus and fungi and cytological tests on blood and bronchoalveolar lavage were negative. A lung biopsy revealed desquamative interstitial pneumonia. High-dose methylprednisolone, subcutaneous methotrexate and rituximab were started. Whole blood from patient and healthy donors (HD) (n = 10) were collected into PAXgene tubes. The type I IFN signature was defined by the expression levels of six IFN-regulated genes (IFI27, IFI44L, IFIT1, ISG15, RSAD2, SIGLEC1), measured by quantitative polymerase chain reaction (qPCR). As previously described by Rice GI, the median fold change of each IFN-induced gene, when compared with the median of the combined HD, was used to calculate the type I IFN score. The mean type IFN score of the HD plus two SD was calculated; we considered as positive a type IFN score higher of 1,42

**Results:** Because of a persistent inflammatory state, ILD and polyarthritis, an autoinflammatory syndrome with lung involvement was suspected (COPA and SAVI Syndromes) The sequencing of coatomer associated protein subunit alpha (COPα) gene showed no mutations while the molecular analysis of the TMEM173 gene by NGS showed the presence of c.463G>A (p.Val155Met) variant in heterozygotic status. Whole-blood gene expression studies demonstrated increased IFN signature (182,2), consistent with the values reported in SAVI patients and in other interferonopathies already described. Elevated levels of CXCL-10 (>1000 pg/ml) were also found

**Conclusion:** The clinical picture of SAVI syndrome is characterized by early-onset (in the first month of life) cutaneous vasculitis, recurrent fevers, ILD, and systemic inflammation. Our patient never developed skin rash unlike the cases described in literature. Therefore, the lack of typical skin lesions should not exclude this clinical suspicion since the respiratory and systemic inflammatory components of the disease may predominate. In the context of early age of onset, ILD, failure to thrive and recurrent fevers, SAVI must be considered as a differential diagnosis. A better understanding of the genotype–phenotype correlation is needed to improve SAVI management and prognosis.

**Consent for publication has been obtained from patient**

Yes

**Disclosure of Interest**

None Declared

#### P2109 A boy with severe phenotype of immune deficiency and relapsing hyperinflammationcured with a hematopoietic stemcell transplantation

##### Kim Ramme^1^, Yenan Bryceson^2^, Mikael Sundin^1^, Sofie Vonlanthen^3^, Anna Carin Horne^4^

###### ^1^Pediatric Hemathology, Karolinska Hospital; ^2^Department of Medicine, Karolinska Institutet; ^3^Molecular Biology; ^4^Department of Paediatric Rheumatology, Karolinska Hospital, Stockholm, Sweden

####### **Correspondence:** Kim Ramme

**Introduction:** Patients with a clinical picture of immune deficiency combined with severe inflammation present significant challenges in diagnosis and treatment.

**Objectives:** Description of the phenotype and treatment of a patient with immune deficiency combined with severe hyperinflammation.

**Methods:** We have investigated a boy of non-consanguineous parents of Middle East origin, with extended immunologic and genetic studies..

**Results:** The boy was born after an uncomplicated pregnancy at 36+6 wks.At two wks of age he developed problems moving his right leg. Osteomyelitis was suspected but never confirmed. At admission he had an unspecific skin rash, pancytopenia and very high inflammatory markers He then developed periodic fever, near-fatal episodes of enterocolitis and reoccurent macrophage activation syndrome (MAS). The boy also suffered different suspected and confirmed infections (Enterococcus coli and Enterococcus faecalis) requiring broad-spectrum antibiotics and ICU admission.The immunological work-up initially showed very high levels ofIL-18 (55,000) but normal levels of IFN-gamma. It also showed showed initially low peripheral blood T- and B-cell as well as NK-cell numbers. T-cells and NK-cells were gradually improving when the hyperinflammation was under control, however the B-cells remained low.Both the CD4+ and CD8+ lymphocytes showed a normal response to stimulation with mitogen and superantigen with FASCIA. Clinical features suggested hemophagocytic lymphohistiocytosis (HLH) or NLRC4 which led to a rapid examination of functional studies and the most common genes for these syndromes, which were absent. Whole genome sequencing was performed and analyzed for genes linked to primary immune deficiencies and autoinflammatory conditions, but no genetic alterations linked to the phenotype were found. Treating the reoccuring infections and the hyperinflammation was a balancing act. Supportive care included regular transfusions of erythrocytes and platelelts as well as total parenteral nutrition.Steroid pulses were initially given to reduce hyperinflammation, followed by peroral dexamethasone and then high-dose Anakinra(10 mg/kg/day).As the dose was lowered hyperinflammation reoccured. Unfortunately neutropenia, a side effect of Anakinra, developed. This was controlled by the addition of colony stimuli factor. The hyperinflammation slowly improved (over months), although an infection triggered a further life-threatning relapse. Due to his severe phenotype a hematopoietic stem cell transplantation (HSCT) was considered necessary, with cord blood as the stem cell source. His condition was initially stable with a complete donor-chimerism, but he later developed a severe thrombocytopenia secondary to immune suppression. Consequently, the immune suppression was stopped at approximately eight months following transplantation, with a marked improvement of the thrombocytes. Unfortunately however he then developed suspected severe GVHD of the skin and a myopathy. This is currently being treated with methotrexate and Etanercept.

**Conclusion:** During recent years the progress in immunological phenotyping and genetic investigations have improved tremendously, requiring close collaboration between physicians, clinical immunologists and molecular biologists. However, there are still patients where a specific genetic diagnosis cannot be established, making the choice of treatment difficult. In this case a boy with a severe phenotype of immune deficiency and relapsing hyperinflammation was cured with an HSCT.

**Consent for publication has been obtained from patient**

Yes

**Disclosure of Interest**

None Declared

#### P2110 Two genes and one phenotype: same therapy?

##### Silvia Ricci^1^, Ilaria Pagnini^1^, Elena Sieni^2^, Chiara Azzari^1^, Martina Cortimiglia^1^, Giusi Mangone^1^, Lorenzo Lodi^1^, Elisa Calistri^1^

###### ^1^Pediatric Immunology, AOU Meyer- University of Florence, Florence, Italy; ^2^Paediatric Haematology/Oncology, AOU Meyer- University of Florence, Florence, Italy

####### **Correspondence:** Silvia Ricci

**Introduction:** We report two cases of 2-years old twins with periodic fever. They were born at 36 weeks of gestational age after pregnancy with donor oocytes in-vitro fertilization (IVF). The patient came to our attention at 1 year of life due to recurrent episodes characterized by high fever (39-40°C) for 3-4 days, associated with bilateral cervical lymphadenopathy, diarrhea, not constantly morbilliform rash. Laboratory findings, during these episodes, included leukocytosis, thrombocytopenia, anemia, and elevated C-reactive protein. Our patients were generally well between attacks. Regarding their past clinical history: at first months of life they underwent to intervention foranal fistula, discovered on neonatal period. They suffered also from recurrent wheezing since the introduction in the nursery. They presented optimal growth and good psychomotor development.

**Results:** We performed immunological workup that evidenced elevated levels of immunoglobulin D (P1: 150 mg/L and P2 250mg/L, normal values 5-120mg/L) and serum amyloid A (P1 124 mg/L and P2 89 mg/L; normal values < 6.4 mg/L), normal immune-phenotyping, with normal lymphocyte T and NK function. We performed analysis of the MVK gene and we identified two known^1^missense compound heterozygous mutations: c.803T > C (p.I268T) and c.1129G > A (p.V377I). In particular, p.I268Tis described in homozygosity in a patient who died at 4.5 months of age. Moreover, other authors, demonstrated in fibroblast-derived lysates of patients with the same mutation, markedly reduced or absent mevalonate kinase activity and in vitro studies they demonstrated dramatically reduced enzyme activity of the mutant protein. These data indicate that the p.I268T mutation markedly affects enzyme stability and activity^2-4^. In 2016, the same genotype p.V377I /p.I268T was described in 25 patients of a cohort of 114 patients (21%) from the Eurofever Register^5^. The study demonstrated statistically significance between patients with p.V377I/p.I268T versus all other genotypes for amyloidosis developing (p 0.049).

At the same time, for the history of perinatal anal fistula and recurrent inflammatory attacks, despite no complete criteria for HLH (Hemophagocytic lymphohistiocytosis), knowns FHL (Familial Hemophagocytic Lymphohistiocytosis) genes and SH2D1A and XIAP genes were sequenced and a known hemizygous mutation c.1408A>T (p.T470S) was revealed on XIAP gene (chromosome X). Evidently, it was no possible to demonstrate hereditary of this mutation, because maternal DNA was not available. This mutation is a known missense mutation with very low frequency (reported with frequency <1% in HGMD) and previously reported as causative of XLP2^6^(X-linked lymphoproliferative disease 2) and very early onset Crohn’s disease (VEOIBD)^7^. Cytofluorimetric and western blotting analysis showed good expression of XIAP protein in our patients. No others functional analysis was performed, until now. After initial treatment with nonsteroidal anti-inflammatory drugs and steroids given during attacks, therapy was switched to colchicine without clinical success.

**Conclusion:** On the basis of frequent association between their MVK genotype and amyloidosis (16% of patients)^5^, and persistent high levels of serum amyloid A (mean values: P1 261mg/L and P2 120mg/L) we decide to start with IL-1 inhibitor (Anakinra) that based on limited literature data, appeared to improve symptoms and could induce complete remission in patients with MKD. Moreover, studies are ongoing to test the response to IL-1 inhibitor in lymphoproliferative disease. The coexistence of mutations of different autoinflammatory syndromes in our patients has been reported very rarely, and its implications are not totally clear, but they include atypical clinical manifestations with a variable response to treatment.

**Consent for publication has been obtained from patient**

Yes

**Disclosure of Interest**

None Declared

#### P2111 Autoinflammatory phenotype as a mask of oncological pathology

##### Evgeny Fedorov, Svetlana Salugina

###### Nassonova Research Institute of Rheumatology, Moscow, Russian Federation

####### **Correspondence:** Svetlana Salugina

**Introduction:** The interest of the professional community in auto-inflammatory diseases (AID) is growing worldwide. Diagnosis of AID began to be made more often. It must be remembered that AID is a diagnosis of exclusion of other conditions with a similar clinical picture, including oncological pathology.

**Objectives:** To present the observation of oncological disease in a child with suspected AID.

**Methods:** Clinicalcase of a 5-year-old girl who entered the Federalrheumatological center in April 2018 to exclude AID. Complaints at admission were periodic fever 38.5 lasting 6-7 days, abdominal pain, increased posterior cervical lymph nodes, sometimes tonsillitis attacks, arthralgia, weakness, fatigue. Patient was examined according to the standard program of the patient with systemic juvenile arthritis (soJIA). Infectious diseases were excluded. X-ray examination and radioisotope skeleton scintigraphy, bone marrow biopsy, molecular genetic analysis using automatic sequencing for mutations in the NLRP3 and TNFRSF1A genes in hot points were performed.

**Results:** The family history of AID is not burdened. The patient is sick from 4 years (from February 2017). In the onset of disease - febrile fever, pain in the cervical spine, cervical lymphadenopathy. After 2 months arthralgia of the knee, synovitis of the hip, claudication appeared. On examination, it was an increase of acute phase markers, anemia. At the initial examination infections, tuberculosis were excluded, on abdominal CT - an increase in para-aortic lymph nodes, radioisotope scintigraphy of the skeleton without pathologicalfeatures. According to bone marrow biopsy, there is no data for hematologic disease. Diagnosis: soJIA. Treatment: NSAIDs, against the background of which the pain in the joints stopped. From the 5th month of the disease was in the form of monthly febrile fevers, lasting 6-7 days, accompanied by hyperemia of the pharynx and raid on the tonsils, spotty rashes, enlarged cervical lymph nodes, abdominal pain, pain in the knee. ESR 59 mm/h, Hb-96 g /l, CRP-43.8 mg/l, WBC-7.3х10^9^/l . During hospitalization in the rheumatological center in April 2018 there were febrile fever, weight loss, intoxication (weakness, lethargy, moodiness), generalized lymphadenopathy, liver enlargement, arthritis of the left hip joint, Hb 92 g /l, ESR 48 mm/h, CRP 30 mg/l. There were no pathogenic mutations in the NLRP3 and TNFRSF1A genes. On the X-ray of the pelvis and scintigraphy - multiple lesions in the bones. Sent for re-examination by an oncologist. The increase in the level of the tumor marker of a neuron-specific enolase detected during additional examination, the results of the femur biopsy confirmed the diagnosis: low differentiated neuroblastoma without primary focus, 4 stages with multiple lesions in bones and para-aortic lymph nodes

**Conclusion:** The diagnosis of AID should be considered only after the exclusion of other, more common and severe pathologies. In clinical practice physicians who areworking withpts with an auto-inflammatory phenotype should be aware of possibility of oncological disease.

**Consent for publication has been obtained from patient**

Yes

**Disclosure of Interest**

None Declared

#### P2112 Coexistence of familial Mediterranean fever with celiac disease in armenian patient (case report)

##### Hasmik Sargsyan

###### Pediatrics N2, Yerevan State Medical University, Yerevan, Armenia

**Introduction:** Familial Mediterranean Fever (FMF) is autosome-recessive inherited disorder, particularly frequent around the Mediterranean basin. FMF shows a marked ethnic distribution; it is most frequently observed in Armenian, Jewish, Turkish and Arabic communities. In general, FMF is characterized by recurrent episodes of fever, associated with serositis. Celiac disease (CD) known as gluten enteropathy is immune-mediated inflammatory disease of the small intestine caused by gluten sensitivity in genetically predisposed people. The most common symptoms of CD are chronic diarrhea, anorexia, weight loss, abdominal distention and pain. However, both of the diseases share some common clinical features and sometimes, both of the diseases are associated with many inflammatory and autoimmune diseases. It is necessary to mention, that there are some cases of FMF with CD in the literature.

**Objectives:** We present FMF patient coexistence of CD. Despite the high level of FMF in Armenia, this is the first case of these two disorders.

**Methods:** Clinical and laboratory findings are presented.

**Results:** 14 years old boy admitted to the “Arabkir” Medical Center with complains of: fever, recurrent abdominal pain, chest pain, arthritis of knee and ankle, sometimes nausea, vomiting, irregular stool. Manifestation of disease began from the four years old. The attack was 3-5 times per month each lasting typically for 3-4 days. There is a family history of FMF: sister and brother have FMF (with M694V/M694V mutation). Physical examination revealed M-46, P-168, BMI-16,31, paleness, low muscular tone, enlargement of the spleen.Some investigations as a IgA, IgM, IgG, C3, C4, ASLO, RF, PT, serological tests for viral or bacterial infections were negative, kidney and liver tests also without any abnormalities. The inflammation markers were positive (CRP 176.11(N<5mg/dl), ESR 25mm/h, ferritin 101.27(30-300ng/ml). Chest X-ray revealed exudates in the costophrenic angle from the two sites, EchoCG – aseptic pericarditis, ultrasound of abdomen – splenomegaly and fluid in abdomen. The vitaminD were checked. Specific markers of CD (T-TG IgA 92.6(N<10), HLA DQ2, HLA DQ8, EMA IgA) was positive. Duodenum biopsy was revealed Marsh Type III. Genetic investigation of MEFV discovered M694V/M694V mutation. We established the diagnosis of FMF and CD at the same time rule out other diseases (endocrine, autoimmune, ets.). It was started the treatment with colchicine 1mg/day and gluten-free diet.

**Conclusion:** This is a family case of FMF, one of the member has a CD.It is necessary to continue investigations the siblings in the field of CD. Not excluded, that should be revealed some factors which can confirm familial form of CD in this family. However, for the understanding relationship between CD and FMF should be further studies with more patients for a providing precise interpretation.

**Consent for publication has been obtained from patient**

Yes

**Disclosure of Interest**

None Declared

#### P2113 Inflammation arising on combined genetic background characterized by simultaneous occurrence of NOD2 and IFNGR1 mutations

##### Anna Sediva^1^, Zuzana Parackova^1^, Markéta Bloomfield^1^, Adam Klocperk^1^, Irena Zentsova^1^, Michael Svaton^2^, Eva Froňková^2^

###### ^1^Department of Immunology; ^2^Department of Pediatric Hematooncology, Motolm University Hospital, 2nd School of Medicine, Charles University, Prague, Czech Republic, Prague, Czech Republic

####### **Correspondence:** Anna Sediva

**Introduction:** New technologies such as NGS enable elucidation of genetic cause in patients with primary immunodeficiencies with complex, atypical or ambiguous presentation. Here we present a patient with a complicated clinical picture characterized by severe bcgitis in the neonatal and infant age, followed later by manifestations of autoinflammation characterized by generalized skin lesions, lymphoproliferation, recurrent erythema nodosum, joint involvement and bone lesions.

**Objectives:** NGS and testing of immune function in an effort to precise diagnosis and set personalized therapy.

**Methods:** NGS was performed by Illumina-based technology, functional assays included complex immune profiling, analysis of signalling pathways and cytokine response after stimulation by IFNg (due to IFNGR1) and muramyl dipeptide (MDP) (in a context with NOD2 mutation). To verify the effect of the therapy functional tests were repeated after initiation of Methotrexate treatment.

**Results:** In the patient, mutations in IFNGR1 and NOD2/CARD15 genes were detected simultaneously bearing an increased susceptibility to mycobacterial infections and NOD2 associated Blau syndrom. The patient’s cellular phenotype correlated with known features of partial IFNGR1 deficiency, a marked overexpression of surface IFNGR1, decreased IFNγ-induced STAT1 phosphorylation, overproduction of IFNγ by T cells upon stimulation, significantly decreased IL-12p70 production by PBMCs upon IFNγ and LPS co-stimulation and a reduction of expresion of STAT1-inducible genes, *CXCL10* and *IRF1*. The functional property of the *NOD2* mutation indicated an enhanced basal NFκB activation, MAPK phosphorylation and expression of transcription factors c-Jun and c-Fos. The stimulation with MDP resulted in further increase of NFκB activation, but only minimal increase in MAPK phosphorylation.

Interestingly, treatment with methotrexate alone almost completely resolved the inflammatory symptoms. This was paralleled with suppression of untreated and MDP-induced NFκB activation, MAPK activation and MDP-induced and simultaneous IFNγ and MDP-induced production of cytokines.

**Conclusion:** We present here the patient with a dominant autoinflammatory phenotype consistent with NOD2 related Blau syndrome, influenced by a simultaneous immune deficiency based on IFNGR1 mutation. Based on the results of functional testing we hypothesize that both mutations exhibit certain mutual alteration. While the NOD2-derived proinflammatory bias may decrease the susceptibility to mycobacterial infections associated with IFNGR1 deficiency, the strongly Th1-polarized immune system may accentuate the inflammatory responses via IFNγ-driven priming of NOD2-mutated cells, reflecting in the patient’s severe granulomatous disease. We also show significat effect of methotrexate chosen to influence granulomatous inflammation in a situation when antiTNF treatment could not be used due to an increased susceptibility to Mycobacteria.

**Disclosure of Interest**

None Declared

#### P2114 Relapsing-remitting systemic and neurological symptoms in Griscelli disease

##### Alessandra Tozzo^1^, Nicoletta Milani^1^, Gianluca Marucci^2^, Luisa Chiapparini^3^, Alessandra Erbetta^3^, Elena Sieni^4^, Nardo Nardocci^1^

###### ^1^Neuropsichiatria Infantile; ^2^Anatomia Patologica; ^3^UOC Neuroradiologia, Fondazione IRCCS Istituto Neurologico Besta; ^4^Oncoematologia Pediatrica, Azienda Ospedaliero-Universitaria A.Meyer, Milano, Italy

####### **Correspondence:** Alessandra Tozzo

**Introduction:** We present the case of a 3 years old Romanian healthy boy who developed a relapsing-remitting multifocal encephalomyelopathy and episodic pancytopenia, hepatosplenomegaly and fever.

**Objectives:** Our patient had a physiological and a remote pathological anamnesis mute up to 3 years of age.

He was the first-born of non consanguineus parents.

He appeared as an healthy child with very light hairand skin. After some day of a flu-like syndrome,he showed systemic symptoms (fever, pancytopenia, hepatosplenomegaly) and then developed neurological symptoms consisting of focal epilepsy, cerebellar signs, right hemiparesis.

Systemic and neurological symptoms were responsive to steroids.

After a short period of well being, he had two more episodes of worsening of neurological symptoms. A few months later, during steroid and antiepileptic therapy, he developeda rightnon-convulsive status epilepticusand then a contralateral one.

**Methods:** Neurological symptoms werein temporal correlation with the appearance ofmultifocal extensive lesions of the white matter in brain andcervical cord, with enhancement.

The CSF investigations showed a mild protein elevation. Brain biopsy was compatible with non-specific findings suggestive of inflammatory pathology. We made further investigations in order to exclude infective, metabolic, autoimmune or autoinflammatory diseases . The strong diagnostic suspicion in the context of pathologies that could secondarily be associated with hemophagocytic lymphohistiocytosis induced a deepening in specific immunodeficiencies.

**Results:** We identified a mutation in Rab27a in homozygosity responsible for the type II Griscelli syndrome.

**Conclusion:** Griscelli Syndrome Type 2 (GS-2) is caused by mutations in *RAB27A* gene and is associated with immunological dysfunction without primary neurological impairment. The cytotoxic defect caused by RAB27A mutations is responsible for triggering the hemophagocytic syndrome characterized by acute onset of uncontrolled T-lymphocyte and macrophage activation. Neurological symptoms in GS-2 may be due to infiltration of brain by the activated hematopoietic cells (1). A relapsing- remitting course is not typical for GS-2. We can’t demonstrate a sure hemophagocytic syndrome (ferritin was always normal) but we had episodic pancytopenia, hepatosplenomegaly and fever responsive to steroid. Unfortunately, although the genetic defect was identified, the child's general condition was too compromised to carry out a stem cell transplant and medical therapy proved ineffective; after a long period of medicaltreatment the patient died. This case showes the importance to not avoid to consider rare conditions even with atypical manifestations.

**Reference**

(1) Intractable Rare Dis Res. 2017 Feb; 6(1): 76–79. “Griscelli syndrome subtype 2 with hemophagocytic lympho-histiocytosis: A case report and review of literature”. Priyanka Minocha, Richa Choudhary, Anika Agrawal, Sadasivan Sitaraman

**Consent for publication has been obtained from patient**

Yes

**Disclosure of Interest**

None Declared

#### P2115 Deficiency of adenosine deaminase 2: the importance of a diagnosis before irreversible damage

##### Elena Tronconi^1^, Francesca Conti^1^, Duccio M. Cordelli^2^, Stefano Volpi^3^, Marco Gattorno^3^, Andrea Pession^1^, Angela Miniaci^1^

###### ^1^Department of Pediatrics; ^2^Child Neurology and Psychiatry Unit, Pediatric Department, Sant'Orsola-Malpighi Hospital, University of Bologna, Bologna; ^3^Clinica Pediatria e Reumatologia, Istituto Giannina Gaslini, Genova, Italy

####### **Correspondence:** Elena Tronconi

**Introduction:** We present the case of a 15 years old girl who was admitted to our hospital for acute lower limb flaccid paralysis.

**Results:** Physical examination revealed marked and diffuse livedo reticularis. Neurologic exam showed peripheral deficit of the VII cranial nerve, motor activity at lower limbs was absent with sensitive deficit. She underwent a brain and spinal magnetic resonance imaging (MRI) showing multiple areas of signal abnormalities (T2 hyperintensity) in both thalamus and basal ganglia, small area in the spinal cord at C2-C3, at D10-D11 and L1 levels; these images were related to vascular damage. Blood exams showed high blood counts and high inflammatory markers (PCR 16.76 mg/dl). Coagulation was normal, antiphospholipid and antinuclear antibodies were negative, immunological work-up showed normal immunoglobulins level, T and B phenotype. She started intravenous steroid and received high dose intravenous immunoglobulin in 4 days. The constellation of findings led to clinical suspicion for adenosine deaminase 2 (ADA2) deficiency (DADA2), which was confirmed by low plasma ADA2 activity. For the presence of microhematuria, worsening proteinuria and hypertension, vascular ultrasound was performed with no evidence of renal artery stenosis. She underwent renal biopsy negative for glomerulonephritis, renal vasculitis and amyloidosis. Following the diagnosis of DADA2, she was initiated on etanercept 50 mg once weekly and she was able to switch from intravenous to oral prednisone and to start its tapering with normalization of inflammatory markers and improvement of skin rash. She underwent an intensive daily rehabilitation training with improvement in the trunk stability but without recovery of the leg paralysis. The girl is the second of 3 children, born from consanguineous parents (first grade cousin) of Italian origin. By the age of 1 she started suffering from fever, arthritis, pericarditis with elevation of inflammatory markers and was diagnosed with systemic juvenile idiopathic arthritis (sJIA). At that time, she already presented livedo reticularis and chronic disease anemia. She was treated with short term oral steroid first and later with methotrexate with remission of fever and articular symptoms. She presented speech delay. At the age of 2 presented a convulsive crisis. Polysomnography and brain MRI were negative. One year later she presented a convulsive status epilepticus, brain MRI was repeated and resulted negative. She was started on topiramate with no further crisis, treatment was stopped 2 years later. When she was 9, she was admitted for pancytopenia and she was diagnosed of Leishmania infection. The two siblings of the girl were tested for plasma ADA2 activity and both resulted deficient. The boy is a 12 years old boy with mild development delay and a previous diagnosis of sJIA at the age of 5. He was treated with steroid and Methotrexate with remission. The older sister is now 19 years old and she had an episode of erythema nodosum. Genetic testing of all the family members is still pending. In 2014, two independent groups, Zhou et al. and Navon-Elkan et al., described the first 34 patients with disease-associated mutations in ADA2. During the past 4 years, over 170 new patients of many ancestries have been reported in the literature showing a highly variable phenotype.

**Conclusion:** Given the important morbidity, especially in consanguineous families, a high index of suspicion is needed for early diagnosis and precocious intervention, before persistent neurologic damage has been established. Our report describes for the first time a new neurological presentation as acute transverse myelitis in a patient with DADA2.

**Consent for publication has been obtained from patient**

Yes

**Disclosure of Interest**

None Declared

#### P2116 The case study of undifferentiated autoinflammatory disease treated with anakinra

##### Marina Visnevska^1^, Anija Meiere^1^, Zane Davidsone^1^, Ruta Santere^2^, Valda Stanevica^1^

###### ^1^Rīga Stradiņš University; ^2^Children's Clinical University Hospital, Riga, Latvia

####### **Correspondence:** Marina Visnevska

**Introduction:** Patients with symptoms commonly found in autoinflammatory disorders may not fit a specific diagnosis, either because their clinical phenotype is nondiagnostic or genetic tests are negative. The term undifferentiated systemic autoinflammatory disorder (uSAID) has been used to describe such cases (*Harrison et al*., 2016). Recognizing uSAID requires a high degree of clinical suspicion and remains a diagnosis of exclusion with suboptimal treatment.

**Objectives:** To report an unsolved case of uSAID with successful anti-IL-1 treatment.

**Methods:** We report a case of a 10-year-old boy, presented in Children’s Clinical University Hospital in Riga, Latvia in December 2013 with fever lasting for 3 weeks and knee joint pain.

The boy was born full term from physiological pregnancy and had no family history of autoinflammatory disease or consanguinity. In medical history he had surgical treatment of cryptorchidism, frequent pneumonia, bronchitis episodes and asthma diagnosis in later childhood. At the age of 7 he experienced an episode of bloody diarrhoea. Since the age of 8 he had recurrent acute otitis media.

In December 2013, while in hospital, ultrasound showed bilateral suprapatellar bursitis. Temperature elevations were not observed. The patient was treated as reactive arthritis with NSAIDs. In January 2014 he developed recurrent episodes with fever up to 40°C every 3-4 days. Laboratory tests showed CRP 10 mg/L, ESR 73 mm/h, negative autoimmune profile and normal results of bone marrow biopsy (BMBx). The examinations and clinical findings suggested systemic JIA (> 1 joint involved, fever > 2 weeks, hepatosplenomegaly).

Fever and knee joint pain disappeared at first with pulse corticosteroid (CS) treatment. Until July 2016, he received treatment with 6 CS pulses, low-dose oral CS daily and methotrexate (MTX).However, with CS dose reduction subfebrility and erythema nodosum-like (EN-like) lesions appeared periodically. Skin biopsy showed non-specific changes.

During July and August 2017, the boy had febrile temperature every other day when CS were not given and experienced aphthous stomatitis (possibly MTX-related). The patient became CS dependent. He had recurrent elevated temperature, fatigue, headache, abdominal pain, EN-like lesions.Secondary osteoporosis with vertebral compression fractures was diagnosed. Repeated skin biopsy showed undifferentiated panniculitis. Weber-Christian disease was excluded. Laboratory tests showed Serum Amyloid A 138 mg/L (N <6.4).The diagnosis of Still’s disease was reconsidered. Genetic tests for 50 autoinflammatory genes and NOD2 gene were negative. Urinalysis showed normal mevalonic acid level. Treatment with Azathioprine 2 mg/kg and Plaquenil 200 mg once daily was started without significant response. In December he had a fever with CRP 99 mg/L, without signs of infection and CRP normalization without a/b therapy, with an episode of EN-like eruptions with non-specific panniculitis in histologic examination. BMBx remained normal. Cyclosporin A 3 mg/kg was initiated and condition improved slightly. In May 2018 Anakinra 100 mg/day was started, with good response: no recurrent episodes of fever, inflammatory marker values normalized, EN-like lesions appeared periodically.

**Results:** The boy presented with uSAID, with a time of 4 years to diagnosis, with the initial diagnosis of systemic JIA. The results of genetic testing were negative. The disease was only partially controlled by or unresponsive to DMARDs and steroid treatment. Good response was achieved with IL-1 blocker.

**Conclusion:** 1. USAID should be considered in the differential diagnostics of systemic JIA.

2. Genetic testing must be performed in patients presented with a range of nonspecific autoinflammatory symptoms.

3. We conclude that Anakinra seems a feasible treatment option for uSAID.

**Consent for publication has been obtained from patient**

Yes

**Disclosure of Interest**

None Declared

#### P2117 Etanercept in the treatment of familial Mediterranean fever: a case report

##### Yeli̇z Zahi̇roglu

###### Rheumatology, Health Sciences University, Samsun Education and Research Hospital, ılkadım, Turkey

**Introduction:** Familial Mediterranean Fever (FMF) is an autoinflammatory disease characterized by recurrent fever,peritonitis,pleuritis or arthritis attacks.The main treatment of FMF is daily oral colchicine treatment. There is strong evidence that colchicine inhibits both acute inflammatory attacks of FMF and systemic amyloidosis.We aimed to report the response of a 2-year-old patient with colchicine-resistant FMF to the treatment of Etanercept.

**Objectives:** A two-year-old girl complained of recurrent fever one year ago. R202Q heterozygote positivity was determined in the gene analysis and colchicine 0.5mg / day was started with the diagnosis of FMF. Although the patient received this treatment regularly, she had fever and abdominal pain attacks for the last 4 months and her attacks started to increase day by day. She lost appetite and weight loss. The older brother had FMF in his family history. Physical Examination: height: 89,5cm, weight: 11,5kg, Fever: 38 degrees, abdominal sensitivity was present, there was no arthritis and rash. In laboratory tests; Sedimentation: 31mm/ h, C-reactive protein: 65mg / L, Fibrinogen: 4.27g / L. The patient was treated with colchicine 1mg / day, ibuprofen 200mg / day and prednisolone 5mg / day. Four weeks later, the patient complained of abdominal pain, fever, loss of appetite and weight loss. The frequency of attacks was not decreased.

**Methods:** The patient who was treated with subcutaneous Etanercept at 0.8mg / kg / week with the diagnosis of colchicine-resistant FMF, had no abdominal attacks and fever episodes.

**Results:** After 6 weeks there was no attack and sedimentation, CRP and fibrinogen levels decreased to normal limits.

**Conclusion:** There are no more alternatives for patients who do not respond to colchicine or cannot tolerate the drug.Considering cytokine patterns; Anti -TNF agents may be an alternative in the treatment of FMF.

**Consent for publication has been obtained from patient**

Yes

**Disclosure of Interest**

None Declared

### New monogenic diseases

#### P2118 A case of neonatal-onset proteasome-associated autoinflammation and immunodeficiency disease resembling but distinct from Nakajo-Nishimura syndrome

##### Noriko Kinjo^1^, Satoru Hamada^1^, Koichi Nakanishi^1^, Hiroyuki Mishima^2^, Akira Kinoshita^2^, Koh-Ichiro Yoshiura^2^, Tsunehiro Mizushima^3^, Jun Hamazaki^4^, Shigeo Murata^4^, Hiroaki Hemmi^5^, Tsuneyasu Kaisho^5^, Nobuo Kanazawa^6^

###### ^1^Department of Pediatrics, University of the Ryukyus., Nishihara-cho, Okinawa; ^2^Department of Human Genetics, Atomic Bomb Disease Institute, Nagasaki University, Nagasaki; ^3^Picobiology Institute, Graduate School of Life Science, University of Hyogo; ^4^Laboratory of Protein Metabolism, Graduate School of Pharmaceutical Sciences, University of Tokyo, Tokyo; ^5^Department of Immunology, Institute for Advanced Medicine, Wakayama Medical University; ^6^Department of Dermatology, Wakayama Medical University, Wakayama, Japan

####### **Correspondence:** Noriko Kinjo

**Introduction:** We report a Japanese boy with a distinct neonatal-onset autoinflammatory phenotype. Exudative erythemas developed on his face and extremities 2 weeks after birth. At 1 month of age, the patient developed fever with elevated serum C-reactive protein and hepatic amino transferase levels. A febrile convulsion with basal ganglia calcification appeared at 4 months. At 7 months of age, severe pulmonary arterial hypertension was diagnosed. He periodically developed erythema and myositis with elevated serum creatine kinase level. Anti-nuclear antibodies were negative. His symptoms were improved with corticosteroid but recurred periodically. Portal hypertension with hepatosplenomegaly was diagnosed at 6 years of age. Although Nakajo-Nishimura syndrome (NNS) was suspected, lipomuscular atrophy has never been developed. This case was once reported in 8^th^ISSAID, but we additionally present the case with the recent disease course and new findings, especially the aspects of immunodeficiency.

**Objectives:** To identify the responsive gene mutation(s) in this patient and to clarify his phenotypic difference from NNS.

**Methods:** Sequencing a panel of genes causing known autoinflammatory syndromes and whole exome sequencing of the patient and his parents were performed using the next generation sequencer. Expression of proteasomeal subunits and ubiquitinated protein was analyzed by western blotting and immunostaining. Proteasomal activities were also analyzed. Serum TREC/KREC levels were analyzed by qRT-PCR.

**Results:** No significant mutation had been detected in a panel of genes causing autoinflammatory syndromes including *PSMB8*, while whole exome sequencing of the patient and his parents identified a novel de novo heterozygous *PSMB9* mutation in the patient. Among proteasome-associated genes, a rare polymorphism in *PSMD9* has additionally been detected in the patient and his father. By analysis of the patient-derived cells, disrupted assembly and maturation of the immunoproteasome similar to NNS, but relatively intact catalytic activities without apparent accumulation of ubiquitinated proteins in contrast to NNS, were observed. Blood analysis showed not only increase of monocytes but also decrease of B cells. A TREC level as well as a KREC level decrease significantly. Notably, serum IgG levels were decreased (less than 500 mg/dL) and IgG supplementation was required every week and efficient as treatment. These facts suggest that humoral immunity was severely impaired.

**Conclusion:** Thus, our case manifested not only autoinflammation but also immunodeficiency, which is resembling but distinct from NNS. This implicates a novel category of autoinflammatory disease, which we may propose proteasome-associated autoinflammation and immunodeficiency disease (PRAID).

**Consent for publication has been obtained from patient**

Yes

**Disclosure of Interest**

None Declared

#### P2119 Proteasome-associated autoinflammatory syndrome 2 (PRAAS2) in a five months oldboy

##### Anna Kozlova, Ekaterina Viktorova, Viktoria Zaharova, Dmitriy Konovalov, Aleksandra Terentieva, Vasiliy Burlakov, Elena Raikina, Natalia Kuzmenko, Julia Rodina, Alexsey Maschan, Anna Shcherbina

###### Immunology, National Medical Research Center of Pediatric Hematology, Oncology and Immunology named after Dmitry Rogachev , Moscow, Russian Federation

####### **Correspondence:** Anna Kozlova

**Introduction:** PRAAS2 is a monogenic autoinflammatory syndrome caused by autosomal dominant mutations in *POMP* gene. It was first reported in 2018 as a disorder with early onset severe inflammatory neutrophilic dermatitis, autoimmunity and variable immunodeficiency. Only 3 patients have been reported to date

**Objectives:** To describe a case ofPRAAS2 ina 5-months-old Russian boy

**Methods:** Genetic defect was identified by whole exome sequencing (TruSeq DNA Exome kit, Illumina). Mutation was confirmed by Sanger sequencing in the patient, was absent in both parents

**Results:** Five-months old boy from healthy non-consanguineous parents was referred to our Center due to the multisystem inflammatory condition.Since the first days of life the patient suffered from vasculitis-like rash with centralnecrosis, recurrent fever, hepatosplenomegaly, thrombocytopenia, high inflammatory markers (leukocytosis, elevated CRP, hypergammaglobylinemia). Skin biopsy revealed non-specific changes including dermal fibrosis, subepidermal and perivascular chronic inflammatory, mostly histiocytic, infiltration. Later he developedbilateral pneumonia, purulent otitis, enterocolitis. Upon admission to our Center he also fulfilled criteria forhemophagocytic lymphohystiocytosis.The patient was treated with broad-spectrum antimicrobials, high-dose steroids,tocilizumab and anakinra, with resolution of cytopenia, but otherwise no effect. The patient’s conditiondeteriorated rapidly, he developed hepatitis with coagulopathy, intracranial and lunghemorrhage, respiratory failureand died from progressive multiorgan failure. Heterozygous de novo c.335delT, p.I112fs mutation in *POMP* gene was identified post mortem.This mutation was not previously reported andpredicted to be deleterious

**Conclusion:** Heterozygous mutations in the same position334-335 was previously described in patient with less severe course ofPRAAS2.Severity of the disease varies in the few patients described, even within the same family.Steroids, IL6 and IL1-blocking anti-cytokine agents were not successful in our patient. Treatment strategy for PRAAS2 is yet to be elucidated

**Consent for publication has been obtained from patient**

Yes

**Disclosure of Interest**

None Declared

#### P2120 A case of novel identified proteasome-related autoinflammation and immunodeficiency syndrome caused by PSMB9 mutation

##### Hidenori Ohnishi^1^, Shinsuke Kataoka^2^, Hideki Muramatsu^2^, Emi Kadoi^3^, Nobuo Kanazawa^4^, Satoshi Okada^5^, Yoshitaka Honda^6^, Kazushi Izawa^6^, Ryuta Nishikomori^6^, Takeshi Taketani^7^, Jun Hamazaki^8^, Shigeo Murata^8^, Yoshiyuki Takahashi^2^, Toshiyuki Fukao^9^

###### ^1^Pediatrics, Graduate School of Medicine, Gifu University, Gifu; ^2^Pediatrics, Nagoya University Graduate School of Medicine, Nagoya; ^3^Pediatrics, Gifu City Hospital, Gifu; ^4^Dermatology, Wakayama Medical University, Wakayama; ^5^Pediatrics, Hiroshima University Graduate School of Biomedical & Health Sciences, Hiroshima; ^6^Pediatrics, Kyoto University Hospital, Kyoto; ^7^Pediatrics, Shimane University Faculty of Medicine, Izumo; ^8^Laboratory of Protein Metabolism, Graduate School of Pharmaceutical Science, University of Tokyo, Tokyo; ^9^Pediatrics, Gifu University Graduate School of Medicine, Gifu, Japan

####### **Correspondence:** Hidenori Ohnishi

**Introduction:** Recently, type I interferonopathy was categorized with autoinflammatory syndrome. Several responsible genes of type I interferonopathy including not only the 7 causative genes of Aicardi-Goutieres syndrome but also *TMEM173*(STING) and the genes associated with proteasome such as *PSMB8*had been reported. Additionally, patients with heterozygous truncated mutation of *POMP*had both type I interferonopathy and combined immunodeficiency. More recently, it was shown that the model mice with STING-gain-of-function mutation had combined innate and adaptive immunodeficiency and virus-induced pulmonary fibrosis. In this situation, we found a patient with a mutation of PSMB9 (also called β1i), which is the major component of immunoproteasome.

**Objectives:** In this study, we present the clinical symptoms and the results of in vitro analysis of a novel identified patient who showed the very early onset severe inflammatory phenotype caused by *PSMB9*mutation in Japan.

**Methods:** The patient is one-month-old male infant. He showed the characteristic phenotypes which included severe skin eruption like chilblain, myositis, hepatitis, pneumonia, pulmonary hypertension, pancytopenia, the increase of inflammatory reactants, the elevated serum ferritin, the increase of protein concentration of the central spinal fluid (CSF), and intracranial calcification. The detailed immunological patterns were evaluated and the gene panel including the known autoinflammatory syndrome was analyzed using next-generation gene sequencer. The trio-based whole exome sequence was also performed as the additional analysis. His dermal fibroblast was established, and the immunoproteasome activity, immunoproteasome assembly analyzed by western blotting of fractionated protein and the cytokine production level were analyzed. The reporter gene activity was analyzed using the dual-luciferase reporter assay system.

**Results:** By the ex vivo analysis using the patient’s peripheral blood, several abnormal patterns were detected. In the lymphocyte subset analysis, CD8, γδT and NK cells were decreased. NK cell activity was also decreased. IFN-α was increased in his serum and CSF. IFN signature of his peripheral blood was also enhanced. Interestingly, in his serum polyoma virus was detected continuously. The gene panel analysis revealed the de novo mutation of *PSMB9*. This mutation was located on the dimer interface between PSMB9 proteins in the complex structure of immunoproteasome. No other known pathogenic gene was identified by whole exome analysis. Furthermore, the retain of premature form of PSMB9 protein, the accumulation of UMP1 and the decreased activity of IFN-γ induced 20S proteasome were shown using his dermal fibroblast. The decrease of production level of IL-6 from TNF-α stimulated his fibroblast was shown. The decreased NF-κB reporter gene activity was shown in the *PSMB9*mutation transfected HEK293T cells.

**Conclusion:** The identified genotype is same with the previously reported mutation in 8th ISSAID by Kinjo N et al. and their clinical phenotypes were quite similar. Thus, we hypothesized that this *PSMB9*mutation caused the early onset immunodeficiency leading the virus infection and this might trigger the type I interferonopathy. We proposed that this disease is designated as “Proteasome-associated autoinflammation and immunodeficiency disease (PRAID)” including POMP and PSMB9 mutation-related diseases.

**Consent for publication has been obtained from patient**

Yes

**Disclosure of Interest**

None Declared

#### P2121 ‘Non-self’ mutation: double-stranded RNA elicits antiviral pathogenic response in a drosophila model of expanded CAG repeat neurodegenerative diseases

##### Rob Richards^1^, Saumya Samaraweera^1^, Andrew Scott^1^, Dani Webber^1^, David Harvey^1^, Olivia Mecinger^1^, Louise O'Keefe^1^, Jen Cropley^2^, Paul Young^2^, Joshua Ho^2^, Cath Suter^2^

###### ^1^Molecular and Biomedical Sciences, The University of Adelaide, Adelaide; ^2^Victor Chang Cardiac Research Institute, Sydney, Australia

####### **Correspondence:** Rob Richards

**Introduction:** Inflammation is activated prior to symptoms in neurodegenerative diseases, providing a plausible pathogenic mechanism. Indeed genetic and pharmacological ablation studies in animal models of several neurodegenerative diseases demonstrate that inflammation is required for pathology.

**Objectives:** However, while there is growing evidence that inflammation-mediated pathology may be the common mechanism underlying neurodegenerative diseases, including those due to dominantly inherited expanded repeats, the proximal causal agent is unknown.

**Methods:** We have utilized Drosophila genetics to explore the ability of various forms of expanded repeat RNA to cause pathology and genetically dissect the responsible pathway.

**Results:** Expanded CAG.CUG repeat double stranded RNA causes inflammation-mediated pathology when expressed in *Drosophila*. Repeat dsRNA is recognised by *Dicer2* as a foreign or ‘non-self’ molecule triggering both antiviral RNA and RNAi pathways. Neither of the RNAi pathway cofactors *R2D2* nor *loquacious* are necessary, indicating antiviral activation. RNA modification enables avoidance of recognition as ‘non-self’ by the innate inflammatory surveillance system. Human ADAR1 edits RNA conferring ‘self’ status and when co-expressed with expanded CAG.CUG dsRNA in *Drosophila* the pathology is lost. Cricket Paralysis Virus protein CrPV-1A is a known antagonist of *Argonaute2* in *Drosophila* antiviral defence. CrPV-1A co-expression also rescues pathogenesis, confirming anti-viral-RNA response.

**Conclusion:** Repeat expansion mutation therefore confers ‘non-self’ recognition of endogenous RNA, thereby providing a proximal, autoinflammatory trigger for expanded repeat neurodegenerative diseases.

**Disclosure of Interest**

None Declared

### I have a possible gene or a strange phenotype. Looking for a second case

#### P2122 A family with SAVI-like vasculopathy, arthritis and cold-induced inflammatory urticarial rash

##### Ester Conversano^1^, Elisa Piscianz^1^, Erica Valencic^2^, Alessandra Tesser^2^, Alberto Tommasini^2^, Serena Pastore^2^, Valentina Moressa^3^, Alessia Omenetti^4^, Bianca Lattanzi^4^, Giulia Gortani^2^, Andrea Taddio^1,5^

###### ^1^University of Trieste; ^2^Institute for Maternal and Child Health IRCCS Burlo Garofolo; ^3^University of Trieste, Department of Medicine, Surgery and Health Sciences, Trieste; ^4^Pediatric Division, Ospedali Riuniti ‘Salesi Children’s Hospital’, Ancona, Italy, Ancona; ^5^Institute for Maternal and Child Health IRCCS Burlo Garofolo, Trieste, Italy, Trieste, Italy

####### **Correspondence:** Ester Conversano

**Introduction:** Awareness of autoinflammatory disorders is allowing the detection of families with phenotypes reminiscent of known monogenic defects but lacking genetic confirmation. We describe three patients from a family of Italian ancestry with autoinflammatory phenotype. Exome sequencing didn’t allow the detection of any mutation in genes associated with autoinflammatory syndromes but held complex results supportive of a possible digenic disorder.

**Objectives:** Describe clinical and genetic features of the family, and to hypothesize treatments targeted to mechanisms likely involved in the disease pathogenesis.

**Methods:** Whole exome sequencing was performed in patient 1, 2, 3 and mother of patient 1. Mutations found were analysed by Sanger sequencing. IFN signature and flow-cytometric assessment of TBK1 phosphorylation was analysed in all the family.

**Results:** Patient (pt) 1, a 2 years old girl, presented urticarial rash since 2 months of age, sometimes aggravated by cold. No history of recurrent fever or abnormal physical and neurological development was reported. Pt 2, her 40 years old father, presented analogues cutaneous lesion associated by sporadic episodes of polyarthritis starting later in childhood compared to his daughter, sometimes treated with prednisone with partial remission of symptoms. Pt 3, the 42 years old uncle, presented episodic fever of unknown origin associated to polyarthritis and urticarial starting during childhood. Corticosteroid therapy did not prevent a severe progression of the disease characterized by cutaneous ulcers and small articulation deforming polyarthritis evolving to erosion of the nasal bridge, deformity of hands and fingers, auto amputation of toes. All three patients present evidence of baseline systemic inflammation with elevations of the erythrocyte sedimentation rate and C-reactive protein. A twin brother and the mother were completely asymptomatic. Whole exome sequencing was performed in pt 1, 2, 3 and mother of pt 1. Two mutations in two candidate genes were found in pt 1, 2 and 3: *ZC3H12D* and *MB21D1*. The first is a new heterozygous c.C564A variation in the *ZC3H12D* gene (stop-gain mutation), coding for a Zn-finger protein involved in the IL-6 mRNA degradation. The second one is a heterozygous c.A948G variation in the *MB21D1* gene, encoding the cytosolic DNA sensor cGAS, one of the main trigger of type I interferon (IFN) production. This variant is already described but no frequency data are reported and is predicted as damaging by different bioinformatic tools. These two mutations were afterwards analysed in twin brother of patient 1 by Sanger sequencing, revealing the presence of only the heterozygous c.C564A variation in *ZC3H12D*. None of the two mutation were found in mother. Except for mother, all components of the family showed an intermediate interferon signature, suggesting a possible involvement of the type I IFN inflammation. Despite the role of the two candidate genes, and the result of IFN signature, the flow-cytometric assessment of the TBK1 kinase phosphorylation didn’t reveal a significant increase of the IFN pathway activation, even after LPS stimulation.

**Conclusion:** This family shows a clinical phenotype of variable severity, not definitely explained by genetic mutation found. The fact that the uncle phenotype is similar to SAVI syndrome is coherent with the possible involvement of dysregulated cGAS pathway in the pathogenesis of inflammation. Further analysis are required to clarify the role of candidate genes to provide a suitable pharmacological approach.

**Consent for publication has been obtained from patient**

Yes

**Disclosure of Interest**

None Declared

**Table 1 (abstract P2122).** Summary of family members: age, sex, results of exome sequencing (mutations) and functional assays

Patient	Age	Sex	Mutation	State of mutations	Interferon Signature	Lymphocyte pTBK1 LPS (NS)	Monocyte pTBK1 LPS (NS)
1	2	F	ZC3H12D:c.C564A MB21D1:c.A948G	Het	Intermediate	3.8(3.9)	1.7(1)
2	42	M	ZC3H12D:c.C564A MB21D1:c.A948G	Het	Intermediate	3.7(4.7)	3.2(2.7)
3	40	M	ZC3H12D:c.C564A MB21D1:c.A948G	Het	Intermediate	-	-
Twin brother pt1	2	M	ZC3H12D:c.C564A	Het	Intermediate	4.2(3.4)	1.2(0.9)
Mother pt1	37	F	-	-	Negative	6.7(3.7)	4(1.4)

#### P2123 New ATM variants and a rare association of juvenile idiopathic arthritis and ataxia-telangiectasia: coincidence or relationship?

##### Nastasia Kifer^1^, Todor Arsov^2,3^, Mario Sestan^1^, Marijan Frkovic^1^, Carola G. Vinuesa^2,3^, Marija Jelusic^1^

###### ^1^Department of Paediatrics, Division of Rheumatology and Immunology, University Hospital Centre Zagreb, University of Zagreb School of Medicine, Zagreb, Croatia; ^2^Centre for Personalised Imuunology (CPI), Australian National University, Canberra, Australia; ^3^China-Australia Centre for Personalised Immunology (CACPI), Australian National University; Renji Hospital, Jiao Tong University, Shanghai, China

####### **Correspondence:** Nastasia Kifer

**Introduction:** Ataxia-telangiectasia (A-T) is an autosomal recessive disorder caused by biallelic pathogenic variants in *ATM* (ataxia telangiectasia mutated) gene. A-T has a variable presentation, including cerebellar ataxia, telangiectasia, gradually developing cerebellar degeneration, immunodeficiency, predilection for cancer occurrence and radiation sensitivity. Juvenile idiopathic arthritis (JIA) is an autoimmune disorder, believed to be of a polygenic nature, encompassing all arthritis of unknown aetiology occurring in children under the age of 16.

**Objectives:** We present a patient diagnosed with a rare association of JIA and A-T which with identified two likely pathogenic *ATM* variants.

**Methods:** We obtained all patient’s records from the Department of Paediatrics, UHC Zagreb. Genomic analysis was done using singleton whole exome sequencing (WES).

**Results:** Our patient is a male with suspected ataxia telangiectasia and JIA. His older sister suffers from cerebral palsy (due to complications of premature birth). He was diagnosed with ataxia at 3 years of age. Brain MRI performed at the age of 15 years revealed pontocerebellar hypoplasia and laboratory testing showed selective IgA deficiency, decrease in proportion of lymphocytes and progressive elevation of alpha fetoprotein concentration. Genetic testing excluded Friedrich ataxia and spinocerebellar ataxia. Cytogenetics analysis detected mosaic translocation (7;14) suggestive of chromosomal instability. These findings were clinically suggestive of A-T.

Regarding the JIA, the patient first noticed swelling of the fifth finger of the right foot at 13 years of age. Two years later the metacarpophalangeal joint of the thumb of his left hand started to swell and finally his left knee. MRI revealed chronic effusion, oedema and a popliteal cyst in the left knee. MRI of the right foot detected erosive changes followed with the expansion of the soft tissue and bone. Ultrasound of hands, left knee and feet confirmed chronic inflammatory changes. Based on these features the patient was diagnosed with JIA and treated with nonsteroidal anti-inflammatory drugs (NSAIDs). Due to the chronic character of JIA and having in mind the concomitant disease, low doses of methotrexate were initiated alongside NSAIDs, after which a stable improvement followed.

Genomic analysis using WES identified two likely pathogenic *ATM* variants: NM_000051.3 (ATM_v001): c.8305T>C, p.(W2769R) and c.5293C>T, p.(Q1765Ter) confirming the A-T diagnosis. Analysis of the familial segregation of the identified variants is underway to ascertain the phase of the variants and identify heterozygous relatives at increased risk of cancer.

**Conclusion:** To the best of our knowledge, this is the second reported case of an association between JIA and typical A-T in the literature. A compelling phenomenon is that the only prior described case was identified in the same geographical region. Further analysis of these case with the rare association between A-T and JIA may help us explore and understand the possible role of the ATM gene in development of JIA.

**Consent for publication has been obtained from patient**

Yes

**Disclosure of Interest**

None Declared

#### P2124 A case of drug-induced undifferentiated connective tissue disease in a 2 years old girl as a manifestation of munchausen syndrome by proxy

##### Tetiana Kovalchuk

###### Department of Pediatrics # 2, Ivan Horbachevsky Teropil State Medical University, Ternopil, Ukraine

**Introduction:** Munchausen syndrome by proxy (MSBP) is a mental health problem in which a caregiver makes up or causes an illness or injury in a person under his or her care, such as a child. Because vulnerable people are the victims, MSBP is a form of child abuse.

**Objectives:** The purpose of the study was to analyze the case of undifferentiated connective tissue disease and probable autoinflammatory disease as a manifestation of MSBP.

**Methods:** A 2-years-old girl was referred to Pediatric Hospital for symptoms of increase in body temperature to 39-40º C, recurrent lower back pain, lethargy, general weakness. A baby was from the first pregnancy. The second pregnancy of the mother ended in premature stillbirth of the twins under unknown circumstances.

**Results:** Physical examination confirmed daily episodes of resistant to antipyretics febrile fever which were accompanied by severe lower back pain and hepatosplenomegaly. Anemia, leukocytosis, high rates of ESR (up to 149 mm/hour), C-reactive protein (up to 267,9 mg/l) and procalcitonin (up to 269,5 ng/ml) were recorded. Slightly increased Ig M and circulating immune complexes were noted in the serum of blood without violations of the cellular immunity. CT of abdomen just confirmed hepatosplenomegaly and mesenterial limfadenopathy. No other lymph nodes have been enlarged. Results of trepanobiopsy allowed to exclude the diagnosis of leukemia. A girl received antibiotics of different groups, nonsteroidal anti-inflammatory drugs and detoxification therapy in relation to an intoxication syndrome of unknown etiology. 6 weeks later were arose positive alarm symptoms, and appendectomy was performed. Catarrhal appendicitis was diagnosed postoperatively. Further daily episodes of high fever up to 39-40°C and lower back pain were observed with symptoms of transient synovitis of the right hip joint. Undifferentiated connective tissue disease and utoinflammatory disease were suspected. A short-term effect of pulse corticosteroid therapy with methylprednisolone was noted. Methotrexate therapy was not accompanied by an improvement of girl’s condition. The results of immunohistochemical study of the skin and kidneys confirmed mesangial proliferative glomerulonephritis with a weak tubule-interstitial component and secondary microvasculitis. In dynamics, oliguria, erythrocyturia, and unstable edema of the face and pubic area were increased on the background of normal rates of urea and creatinine in the blood serum. Methotrexate was replaced by cyclophosphamide. Episodes of hyperthermia last 5-6 days, after the normal body temperature is noted 1-2 days. Given the lack of positive dynamics, cyclophosphamide has been discontinued. Negotiations with European clinics for genetical diagnosis of autoinflammatory disease in this girl were conducted. Three months colchicine therapy didn’t produce any positive effect. After 9 months from the debut of disease a group of doctors documented urinary catheter tie with thread that was the reason of blocked urine flow. During the search policemen seized a blank ampoule of pyrogenal from the mother. Pyrogenal is an immunomodulator which contains the bacterial lipopolysaccharide and enhances the functional activity of the cellular and humoral immune response (produced by the Russian Federation only). Side effects of the drug are dose-dependent and accompanied by high fever, lower back pain, and extremely increased inflammatory blood parameters. From the moment of mother isolation from the child, girl’s condition has improved, all symptoms of the disease have disappeared till now.

**Conclusion:** The diagnostic search for recidive fever is extremely difficult even for a highly qualified doctor. In the search for rare diseases accompanied by a persistent fever, a doctor should always remember about MSBP.

**Consent for publication has been obtained from patient**

Yes

**Disclosure of Interest**

T. Kovalchuk Conflict with: noneShareholder of: none,Grant / Research Support from: none,Consultant for: none,Employee of: none,Paid Instructor at: none,Speaker Bureau of:none

#### P2125 Severe large vessels vasculitis with toe gangrene in a young child

##### Caterina Matucci Cerinic, Roberta Caorsi, Stefano Volpi, Silvia Rosina, Riccardo Papa, Paolo Pietro Picco, Angelo Ravelli, Marco Gattorno

###### Clinica Pediatrica e Reumatologica, Istituto Giannina Gaslini, Genova, Italy

####### **Correspondence:** Caterina Matucci Cerinic

**Introduction:** Takayasu arteritis is a granulomatous large vessel vasculitis involving mainly the aorta and its major branches. It is rare in childhood, with only few anecdotal cases in the first years of life reported in the literature.

**Objectives:** To describe, in a 2-year-old boy, a peculiar clinical phenotype characterized by systemic inflammation, sialoadenitis and large vessels vasculitis, complicated by digital ischemia.

**Results:** A 23 month-old boy was admitted for persistent fever associated with cervical and submandibular swelling, with no response to antibiotics. The minor salivary gland and lymph-node biopsies showed a chronic sclerosing sialoadenitis and a reactive follicular hyperplasia, respectively. The laboratory work up showed a neutrophilic leukocytosis and elevation of acute phase reactants. A broad work-up for infectious diseases was negative, as chest x-ray, whole body STIR-MRI and bone marrow evaluation. Abdominal US identified a slight splenomegaly. All the immunologic investigations were negative except for a B CD20 lymphocyte expansion and an elevation of plasmatic IgG4. The double negative T lymphocytes were negative, as the FAS-mediated apoptosis test. A 41-gene NGS panel for autoinflammatory diseases was negative.

The patient was treated with i.v. metilprednisolone (2 mg/kg), with prompt clinical response and resolution of acute phase reactants, but reappearance at tapering. In light of the non-specific clinical and immunological findings and the age of the patient, treatment with Mophetil Mycophenolate (600 mg/mq twice a day) was started with good response. After 3 months, the patient started to complain with pain of the right foot and after few days presented with cyanosis of the anterior part of the right foot and an ulcer of the first toe, associated to severe systemic hypertension and elevation of acute phase reactants. The Doppler US revealed a reduced patency of the common iliac arteries, with a wall thickening of the common right iliac artery, a discontinuous aspect of the femoral and posterior tibial right arteries and of the subrenal abdominal aorta.

The angioCT and angioMRI showed a thickening of the abdominal aorta, the common right iliac artery, and the distal part of the brachial right artery.

Full dose iloprost, heparine and metylprednisolone pulses were started, together with cyclophosphamide (4 mg/kg/day) with quick amelioration of the pain; however, the ischemia evolved to the entire first toe and the third phalanx of the third and fourth toe.

In the hypothesis of a large vessel vasculitis, treatment with rituximab (375 mg/m2 x 4 weeks) was added, with complete control of systemic inflammation and without a further evolution of the ischemia. When the clinical picture was stabilized, the patient underwent the amputation of the first toe and of the third phalanges of the third and fourth toe.

When the maximal cumulative dose of cyclophosphamide was reached (200 mg/kg) , the patient was switched to azathioprine (3 mg/kg) and the steroids progressively tapered, with good control of the disease.

**Conclusion:** In this case, we present a large vessels vasculitis similar to Takayasu arteritis associated to toe gangrene, that is an atypical manifestation of large vessels vasculitis, in a young child.

The peculiar clinical picture and the age at diseases onset may suggest a monogenic origin. Whole exome sequencing is actually in progress in the patient and the parents.

**Consent for publication has been obtained from patient**

Yes

**Disclosure of Interest**

None Declared

#### P2126 Is this disease related to NLRP3 or MEFV or both?

##### Ayşenur Paç Kısaarslan^1^, Nihal Şahin^1^, Sümeyra Özdemir Çiçek^1^, Afig Berdeli^2^, Zübeyde Gündüz^3^, Hakan Poyrazoğlu^1^, Ruhan Düşünsel^1^

###### ^1^Pediatric Rheumatology, Erciyes University School of Medicine, Kayseri; ^2^Molecular Genetic, Ege University Faculty of Medicine, İzmir; ^3^Pediatric Rheumatology, Acıbadem University School Of Medicine, Kayseri, Turkey

####### **Correspondence:** Ayşenur Paç Kısaarslan

**Introduction:** Cryopyrin-associated periodic syndrome **(**CAPS) is a broad definition for a group of auto-inflammatory disorders caused by activating mutations in the *NLRP3* gene. CAPS involved three disorderscharacterized by blood neutrophilia, fever and tissue-specific inflammation in the skin, joints and conjunctiva. Thus far, more than 200 mutations in the *NLRP3* gene associated with CAPS have been documented in the INFEVER database.

**Objectives:** We presented a patient carried both NLRP3 and MEFV mutations.

**Methods:** Eight year-old female patient was admitted our rheumatology clinic with fever, vomiting, and abdominal pain attacks lasting 4 days for 2 years. An aphthous stomatitis, rash, conjoctivitis, and arthritis were not accompained. The attack ferquency was 4 times in last 6 months. Acute phase rectactans were very high in attack periods. There was consaguinity, but no family history.

On the attack period, the patient was reevaluated. There werenopathologic sings of infectious and rheumatologic diseases. High level of white blood cell(WBC), C-reactive protein(CRP), and eritrocyte sedimantation rate(ESR) were detected. Blood and urine cultures were negative. Genetic sample for autoinflammatory diseases was sent , colchicine 1 mg/d was started. After 20 days, she was admitted withfever, arthritis, systolic murmur, high CRPESR and antistreptolysin-O. An ecocardiographic evaluation showed mitral valve regurgitation. Patient was diagnosed acute rheumatic fever, and started steroid, enalapril, and furosemid treatments. While patient on steroid treatment, all complaints was regressed, and decreased levels of CRP,ESR (APR) . After steroidstopped, attacks of arthritis repeated accompanied by high APR. Complaints of abdominal pain and vomiting decreased on this period. Autoinflammatory disease panel was analysed on NLRP3,NLRC4, IL1RN, PSMB8, TNFRSF1A, MVK, PSTPIP1, MEFV, NOD2, PLCG2, NLRP1, CARD8, NLRP12, CARD14, CECR1, TNFRSF11A, TMEM173, TNFAIP3, LACC1, OTULIN, IL23R, TREX1, IL12RB1, PRF1, DNASE1, UNC13D, STX11, STXBP2, USP43, RIPK1 genes.

**Results:** As the result**,** p.Ile313Val(c.937A>G) (Heterozygos)on NLRP3 gene,p.Pro369Ser(c.1105C>T) (Heterozygos) on exon 3, andp.Arg408Gln (c.1223G>A) (Heterozygos) on exon 3 mutations on MEFV gene were detected. Patient still receivesmaximum dose colchicine, nonsteroidal antiinflammatory drug, and enalapril treatments. Her ophthalmologic and hearing examinations were normal.

**Conclusion:** The Ile313Val mutation is determined as “symptomatic for MAGIC Syndrome” in infevers database.Mouth and genital ulcers with inflamed cartilage (MAGIC) syndrome is a disease that fulfilled criteria for diagnosis of Behcet's disease (BD) and relapsing polychondritis (RP).Our patient had not oral or genital aphthae and polychondritis.We do not know whether our patients clinical findings related to NLRP3 or MEFV mutations or both.

**Consent for publication has been obtained from patient**

Yes

**Disclosure of Interest**

None Declared

#### P2127 M1184V mutation in NLRP1: is that polymorphism or pathogenic mutation?

##### Nihal Sahin^1^, Sumeyra O. Cicek^1^, Afig Berdeli^2^, Aysenur P. Kisaarslan^1^, Zubeyde Gündüz^3^, Muammer H. Poyrazoglu^1^, Ruhan Düşünsel^1^

###### ^1^Pediatric Rheumatology, Erciyes University Faculty of Medicine, Kayseri; ^2^Molecular Genetics, Ege University Faculty of Medicine, Izmir; ^3^Pediatric Rheumatology, Acıbadem Hospital, Kayseri, Turkey

####### **Correspondence:** Nihal Sahin

**Introduction:** Monogenic autoinflammatory disorders are a heterogeneous group of conditions characterized by innate immune dysregulation that typically present in early childhood with fever, rashes, and disease-specific patterns of organ inflammation. The genetic mutations leading to autoinflammatory phenotypes affect pathways the innate immune responses. Nuclear localization leucine-rich-repeat protein 1 (NLRP1) play role regulator of the innate immune system. The NLRP1 inflammasome promotes caspase-1, resulting in IL-1β secretion.

**Objectives:** We present a patient with a periodic fever who was detected the NLRP1 gene mutation.

**Methods:** The genetic analysis was performed at Ege University, Department of Molecular Genetics. The informed consent form for the publication was obtained from patient and her parents.

**Results:** A 7-year-old girl was admitted with recurrent fever to our rheumatology clinic. Her complaints firstly started in 4-year-old and recurred with a monthly. There were enuresis and dysuria in some of the fever periods and she was treated as urinary tract infection. But there were no positive urine cultures at any time. Urinary ultrasonography and renal cortical scintigraphy were normal. In 5-year-old, tonsillectomy and adenoidectomy were performed because of presented tonsillitis in fever attacks and repeated after two years. After these surgeries, the fever was not present in 3 months. But then periodic fever was present again. She had pneumonia at once. In apart of these fever periods, she had the normal physical examination findings. Any rash, arthritis, lymphadenitis, and periorbital edema were not detected. There was no complaint of abdominal pain, diarrhea or myalgia in attacks. She has no remarkable family history. In laboratory examinations in fever attacks, complete blood count and urine analysis were normal, CRP, ESR and serum amyloid A were increased, throat culture was negative. Level of immunoglobulin G, A, M, E was normal but immunoglobulin D was increased. The increased AFRs was seen in the attack-free period also. We considered that she had an autoinflammatory disease and gave colchicine. After colchicine treatment, AFRs normalized, but the fever attacks continued. The comprehensive genetic panel was performed for autoinflammatory diseases. This genetic panel included NLRP3, NLRC4, IL1RN, PSMB8, TNFRSF1A, MVK, PSTPIP1, MEFV, NOD2, PLCG2, NLRP1, CARD8, NLRP12, CARD14, CECR1, TNFRSF11A, TMEM173, TNFAIP3, LACC1, OTULIN, IL23R, TREX1, IL12RB1, PRF1, DNASE1, UNC13D, STX11, STXBP2, USP43, RIPK1. M1184V mutation was detected in the NLRP1 gene.

**Conclusion:** M1184V is a polymorphism in NLRP1. In one study was shown that M1184V exhibited caspase-1 activation and IL-1β secretion. However, this polymorphism was presented in 25% of the population without causing any pathological manifestation. Therefore, we do not know whether M1184V mutation is polymorphism or a pathogenic mutation.

**Disclosure of Interest**

None Declared

#### P2128 Periodic fever syndrome with novel TRNT1 variant - possible cause of TRNT1 deficiency or just an incidental finding?

##### Mario Sestan^1^, Todor Arsov^2,3^, Nastasia Kifer^1^, Marijan Frkovic^1^, Carola G. Vinuesa^2,3^, Marija Jelusic^1^

###### ^1^Department of Paediatrics, University Hospital Centre Zagreb, University of Zagreb School of Medicine, Zagreb, Croatia; ^2^Centre for Personalised Imuunology (CPI), Australian National University, Canberra, Australia; ^3^China-Australia Centre for Personalised Immunology (CACPI), Australian National University, Canberra, Australia; Renji Hospital, Jiao Tong University, Shanghai, China

####### **Correspondence:** Mario Sestan

**Introduction:** Biallelic pathogenic variants in *TRNT1* gene, coding for the enzyme transfer RNA nucleotidyltransferase 1, cause *TRNT1* deficiency with various clinical manifestations of autoinflammation, autoimmunity and immunodeficiency, including the syndrome of congenital sideroblastic anaemia with immunodeficiency, periodic fevers and developmental delay (SIFD).

**Objectives:** Authors present a case of a child with recurrent fevers and epilepsy with *de novo* heterozygous mutation in *TRNT1* gene of uncertain role in disease pathogenesis, successfully treated with TNFα inhibitor.

**Methods:** Retrospective analysis of patient medical data and genomic testing using whole exome sequencing (WES).

**Results:** Three months old female infant was referred to our Department with right-sided hemiconvulsions and dysrhythmic EEG changes treated with valproic acid. Subsequently, she had seizures on several occasions successfully treated with diazepam. Since 11 months of age, the child has had periodic protracted febrile episodes. Seven months after the onset of fever, she developed intermittent erythema accompanied with swelling of both hands, feet, knees and face. Subsequently, episodes of fever recurred in an irregular rhythm. The patient has been showing signs of progressive fatigue and severe facial erythema unrelated to febrility. Her physical exam was unremarkable apart from occasional facial erythema. Laboratory findings were normal, except for the long-lasting microcytic anemia (the lowest hemoglobin was 85 g/L) and elevated C-reactive protein and erythrocyte sedimentation rate during the febrile episodes. Comprehensive laboratory, microbiological and immunological examination failed to establish underlying diagnosis. Bone marrow examinations were normal. The frequent and prolonged febrile episodes were successfully treated with short-term methylprednisolone, however this treatment failed to prevent the occurrence of new episodes. To ameliorate fevers, TNFα inhibitor (etanercept) was consequently introduced in therapy at 5 years of age. Over the four month period after the initiation of TNFα inhibitor, the child had no further febrile episodes or seizures and there was an improvement in appetite and general condition. It is planned to reduce the dose of antiepileptics.

Genomic testing using trio WES identified a *de novo* heterozygous *TRNT1* variant, NM_182916.2 (TRNT1_v001): c.448C>T, p.(R150C), predicted to be pathogenic with high CADD score. Previous cases of *TRNT1* deficiency have been described with bialellic *TRNT1* pathogenic variants and analyses are underway to elucidate whether this finding could establish the underlying diagnosis. At present we hypothesize that this could be a case of unidentified second intronic *TRNT1* variant or that this *TRNT1* variant could have a dominant negative effect. In this respect, it should be noted that in our patient we have identified some but not other features of SIFD.

**Conclusion:** Clinical manifestations associated with *TRNT1* deficiency have a broad spectrum with significant clinical heterogeneity, which makes it difficult for rapid clinical recognition. Although the WES is as a powerful tool for the diagnosis of clinically unrecognized genetic conditions, there are still unresolved cases that pose a challenge in routine clinical practice.

**Consent for publication has been obtained from patient**

Yes

**Disclosure of Interest**

None Declared

#### P2129 A novel autoinflammatory and lymphoproliferative syndrome associated with PIM1 mutations

##### Giovanna Ferrara^1^, Silvio Polizzi^2^, Erica Valencic^3^, Annalisa Chiocchietti^4^, Josef Vuch^3^, Alessia Pin^1^, Elisa Piscianz^1^, Diego Vozzi^3^, Serena Pastore^3^, Paola Tomietto^5^, Andrea Taddio^3^, Flavio Faletra^3^, Umberto Dianzani^4^, Alberto Tommasini^3^

###### ^1^University of Trieste, Trieste; ^2^Meyer Children's Hospital, Firenze; ^3^IRCCS Burlo Garofolo, Trieste; ^4^Universita’ del Piemonte Orientale, Novara; ^5^Azienda Sanitaria Universitaria Integrata Trieste, Cattinara Teaching Hospital, Trieste, Italy

####### **Correspondence:** Andrea Taddio

**Introduction:** Whole exome sequencing (WES) can allow genetic diagnosis in subjects with long lasting clinical stories not supportive of any well defined disorder.

A 35-year-old man was referred to ophthalmologist’s evaluation for blurry vision in his left eye. The fundus examination showed choroidal lesions in both eyes. His past medical history was relevant for recurrent episodes of fever and skin rashes, inflammatory lesions of the osteoarticular-muscular system, one episode of aseptic meningitis, an intracranial granuloma and two episodes of anterior uveitis. In adulthood, his medical history included episodes of fever with urticaria and subcutaneous nodules. The histological examination of two skin lesions showed a reactive angioendotheliomatosis and a leukocytoclastic vasculitis. Spleen biopsy revealed the finding of non-caseating granulomas. Brain TC founded multiple lytic and sclerotic skull lesions. He was diagnosed with atypical sarcoidosis and started oral steroid and methotrexate.

Laboratory data showed persistent elevated erythrocyte sedimentation rate, strong positive C-reactive protein, polyclonal gammopathy and elevated serum amyloid A levels.

**Objectives:** To describe functional and genetic data supporting the role of *PIM1* mutation in a man with early onset multisystemic inflammation and lymphoproliferation.

**Methods:** WES analysis was performed to search for a monogenic cause underlying the disease. Flow-cytometry was carried out to evaluate Pim1 expression, BAD phosphorylation (target of Pim1 kinase) and the effect of PIM inhibitor (PIM447) on cell viability. RNAseq was performed on primary fibroblasts from the patient and from healthy donors. The mutated gene was cloned in a vector to perform further functional studies.

**Results:** WES analysis revealed the de novo heterozygous missense variation c.C1132A (p.H378N) in *PIM1* gene, never described in on-line database and predicted as damaging. Preliminary functional investigations showed that expression of Pim1 in patient’s fibroblasts was comparable to those of healthy controls while phosphorylation of BAD protein was higher in cells from the patient. Moreover, cell from the patient displayed a lower sensitivity to PIM447. RNAseq showed an altered expression profile in genes involved in the extracellular matrix organization. Our data revealed an increased expression of some collagen and matrix metalloproteinases genes, which are related to invasion and migration processes, already known to be impaired in several cancer types, and a decreased expression in genes responsible for anti-tumorigenic activity.

**Conclusion:** *PIM1* is an oncogene that encodes a protein kinase and, indeed, somatic gain of function mutations can be found in cancers. Also preliminary data obtained from our patient suggest a gain of function effect of the p.H378N variant.

The lymphoproliferation may be sustained by the anti-apoptotic action of phosphorylated BAD. Recent data correlated hyperactive Pim1 in tumors with high degree of inflammatory infiltration accompanied by IL6 expression. A role of this cytokine also in our patient was coherent with a good clinical response to a treatment with IL-6 inhibitor.

Although these results are supportive of a role of mutated Pim1 in the observed phenotype, we cannot still claim that this is causative of a novel syndrome. The detection of further cases and functional studies on cells transfected with mutated *PIM1* will help shedding more light on this disorder.

**Consent for publication has been obtained from patient**

Yes

**Disclosure of Interest**

None Declared

